# Design and Preclinical Characterization Program toward Asundexian (BAY 2433334), an Oral Factor XIa Inhibitor for the Prevention and Treatment of Thromboembolic Disorders

**DOI:** 10.1021/acs.jmedchem.3c00795

**Published:** 2023-09-05

**Authors:** Susanne Roehrig, Jens Ackerstaff, Eloísa Jiménez Núñez, Henrik Teller, Pascal Ellerbrock, Katharina Meier, Stefan Heitmeier, Adrian Tersteegen, Jan Stampfuss, Dieter Lang, Karl-Heinz Schlemmer, Martina Schaefer, Kersten M. Gericke, Tom Kinzel, Daniel Meibom, Martina Schmidt, Christoph Gerdes, Markus Follmann, Alexander Hillisch

**Affiliations:** Pharmaceuticals, Research and Development, Bayer AG, 42133 Wuppertal, Germany

## Abstract

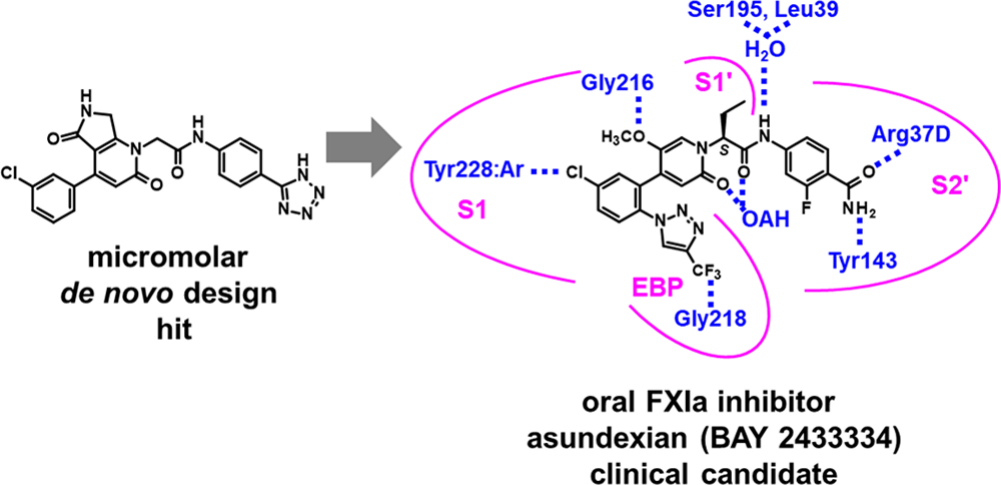

Activated coagulation factor XI (FXIa) is a highly attractive antithrombotic target as it contributes to the development and progression of thrombosis but is thought to play only a minor role in hemostasis so that its inhibition may allow for decoupling of antithrombotic efficacy and bleeding time prolongation. Herein, we report our major efforts to identify an orally bioavailable, reversible FXIa inhibitor. Using a protein structure-based *de novo* design approach, we identified a novel micromolar hit with attractive physicochemical properties. During lead modification, a critical problem was balancing potency and absorption by focusing on the most important interactions of the lead series with FXIa while simultaneously seeking to improve metabolic stability and the cytochrome P450 interaction profile. In clinical trials, the resulting compound from our extensive research program, asundexian (BAY 2433334), proved to possess the desired DMPK properties for once-daily oral dosing, and even more importantly, the initial pharmacological hypothesis was confirmed.

## Introduction

The formation of a network of fibrin fibers and activated platelets is a key physiological mechanism to prevent massive bleedings at sites of vessel wall leakage.^1^ On the other hand, overly excessive fibrin generation may result in the formation of vessel-occluding thrombi, which can cause ischemic damages to the surrounding tissues.^2^ Such events can potentially lead to heart attacks, strokes, or pulmonary embolisms, all of which are ranked high as causes of death or disability and increased healthcare costs.^3^ Therefore, thrombus prevention and resolution remain of key importance for patients and societies.^4^

Since the introduction of novel antithrombotics in the early 2000s, mainly platelet aggregation inhibiting P2Y_12_ antagonists^5^ and direct oral anticoagulants (DOACs),^6^ significant therapeutic advances have been made. The DOACs target the final common pathway of the coagulation system via inhibition of either factor Xa (rivaroxaban, apixaban, and edoxaban) or thrombin (dabigatran). While blocking any kind of fibrin formation by addressing a target after the convergence of the intrinsic and extrinsic cascade is very efficacious, the antithrombotic efficacy of these inhibitors is linked to an increased risk of bleeding, and the identification of “sweet spots” with positive benefit/risk ratios has been a major goal in clinical trials. Nevertheless, especially in many high-risk patients, this bleeding risk is a major concern.

Recently, another serine protease in the coagulation cascade, factor XIa (FXIa),^7^ has emerged as an attractive target, because it may allow for a more selective reduction of fibrin generation: FXI is activated via FXIIa after contact activation or by thrombin in the course of the positive feedback loop for thrombin amplification, but it is not involved in the tissue factor pathway, which is key for fibrin production to cover a vessel wall leakage. Thus, this form of “selective coagulation modulation”^8^ by FXIa inhibition may allow for a decoupling of antithrombotic efficacy and bleeding risk and offer the opportunity for better antithrombotic protection to a much broader patient population. Several lines of evidence support this notion: (1) Individuals with FXI deficiency have a reduced risk for venous thrombosis^9^ or stroke^10^ and very rarely experience any abnormal bleeding events, which are not related to FXI levels. (2) Knock-out mice are protected from thrombosis and have not revealed any increase in bleeding risk versus wild-type animals.^11^ (3) Experiments in animal models in multiple species with irreversible or reversible inhibitors to achieve reduced FXI synthesis or FXI(a) activity have demonstrated strong anticoagulant effects without an impact on bleeding times.^12^ (4) Most importantly, currently, several approaches to inhibit FXI synthesis or FXIa activity are in clinical phases and in these early phases have demonstrated antithrombotic efficacy with less bleedings compared to anticoagulant standard of care or similar bleeding rates compared to placebo.

With regard to parenteral treatment, antisense approaches to reduce FXI synthesis, such as with ISIS 416858 or fesomersen,^13^ and FXI- or FXIa-targeting monoclonal antibodies, such as osocimab,^14^ abelacimab,^15^ or xisomab,^16^ are in Phase 2 clinical evaluation.

Orally bioavailable, reversible FXIa inhibitors might be particularly desirable for chronic treatment, but the research aimed at identifying such small molecule FXIa inhibitors has been challenging despite significant and lengthy efforts. Recently, clinical Phase 2 data^17−20^ have been presented for two orally administered candidates, milvexian^21^ (BMS-986177 or JNJ-70033093) and asundexian^8^ (BAY 2433334, **80**).

In this study, we describe our drug discovery efforts, resulting in the identification of asundexian (BAY 2433334, **80**), a direct, potent, selective, and reversible small molecule FXIa inhibitor for oral application, which is currently being investigated in clinical Phase 3 trials.

## Results and Discussion

To establish a starting point for a program toward orally bioavailable, reversible FXIa inhibitors, we performed several high-throughput screening campaigns using the Bayer AG in-house proprietary compound library, which contained up to 4.3 million compounds. However, a viable hit was not identified.^22^ We therefore pursued a protein structure-based *de novo* design approach with the aim of generating a novel starting point for lead modification toward a reversible, active site FXIa inhibitor. Two structurally distinct compound series discovered by Bristol Myers Squibb were known as FXIa inhibitors at the start of our drug discovery program, a charged 4-aminomethyl-*trans*-cyclohexyl-bearing series and a neutral series containing chloroaryl and 3-aminoindazole moieties. We studied both series in detail to understand their interaction with FXIa.

### Exploration of Distinct Chemical Classes of FXIa Inhibitors: Zwitterionic/Charged FXIa Inhibitors

**Figure 1:**
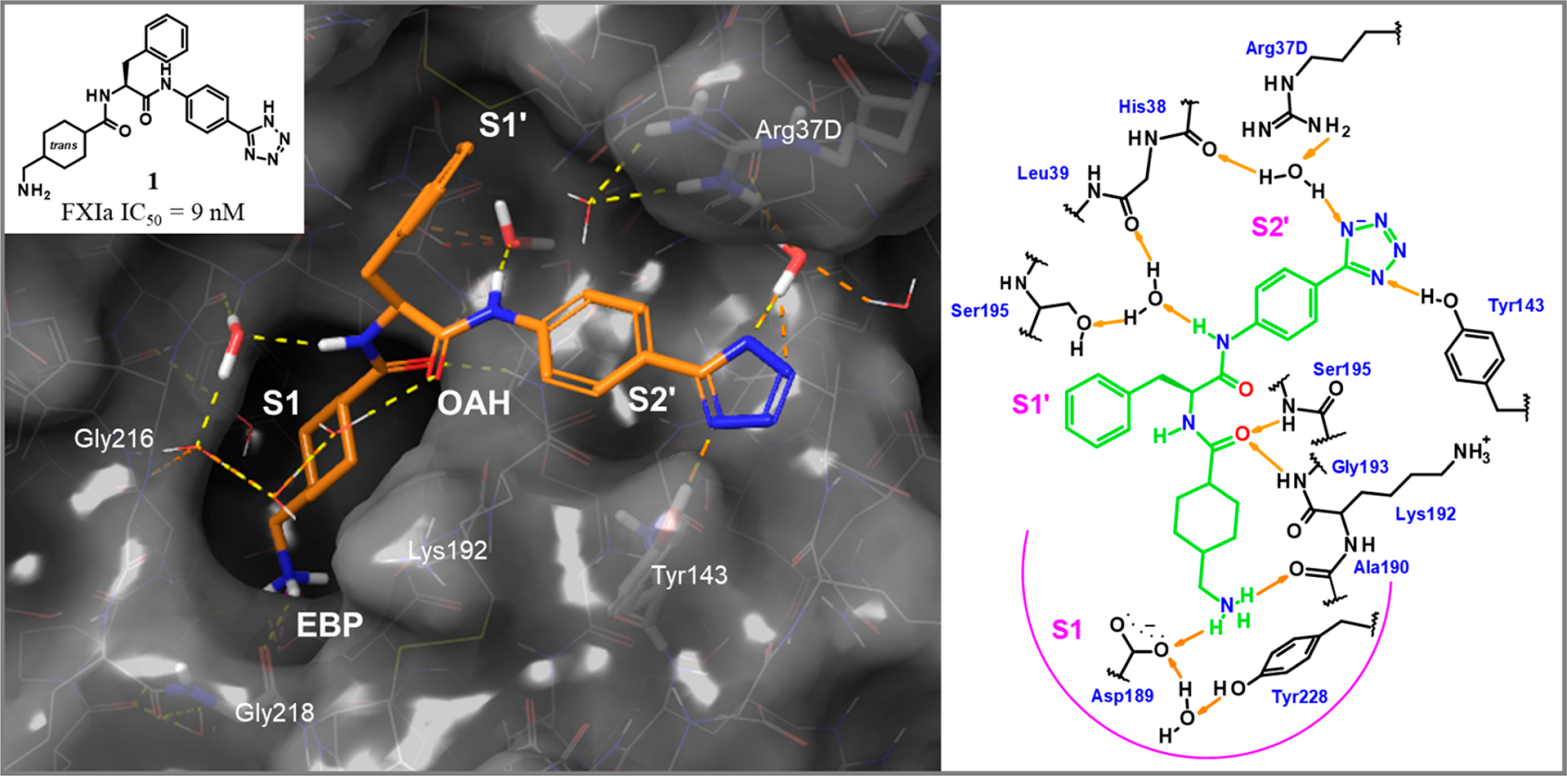
X-ray cocrystal structure of compound **1** in complex with human FXIa (PDB code 8BO4) and the corresponding 2D sketch. S1, S1′, and S2′ pockets, the oxyanion hole (OAH), and the ester binding pocket (EBP) are displayed.

To explore FXIa inhibitors, we synthesized tetrazole **1** (inspired by chemical matter published by Bristol Myers Squibb in 2007^23^) which showed good potency (FXIa IC_50_ = 9 nM, Figure 1). A docking experiment of this compound in the published X-ray structure of FXIa^24^ indicated that the 4-aminomethyl-*trans*-cyclohexyl residue would occupy the S1 pocket. The tetrazole moiety appeared to point toward the S2′ pocket. An X-ray cocrystal structure of tetrazole **1** in complex with human FXIa then revealed the binding mode with the key hydrogen bonds and hydrophobic contacts (Figure 1). The 4-aminomethyl-*trans*-cyclohexyl moiety was indeed found to be located in the S1 pocket where its primary amine forms a direct ionic interaction with the carboxylate of Asp189 and a hydrogen bond with the backbone carbonyl of Gly218. The S1 pocket is the only deep pocket of the FXIa active site, and ligand **1** extends from the S1 pocket into the S1′ and S2′ pockets. We hypothesized that this orientation in the binding mode toward the prime sites might facilitate the required selectivity versus other serine proteases such as FXa and thrombin, as potent inhibitors of the latter use the more druggable pockets of their nonprime site (S1 and S2; S4).^25^ The cyclohexyl-bound amide carbonyl oxygen forms two hydrogen bonds with the protein backbone forming the oxyanion hole (OAH in Figure 1). The ester binding pocket (EBP) is not occupied with substituents of **1**. Located in the S2′ pocket, the tetrazole moiety of **1** forms a hydrogen bond with the hydroxyl group of Tyr143 and a water-mediated interaction with the carbonyl group of His38. The docking binding mode experiment did not indicate the potential of this interaction since Lys192 was kept rigid in that particular X-ray structure. In the S1′ pocket, the phenyl ring of the benzyl side chain participates in a hydrophobic contact with the Cys42-Cys58 disulfide bridge.

However, the low membrane permeability observed for tetrazole **1** made it an unfavorable starting point for our program. We speculated that the rigid molecular structure, which prevents close intramolecular contacts between the counter charged functional groups, had a negative effect on permeation (driven by separated charged moieties).

### Neutral FXIa Inhibitors

**Figure 2:**
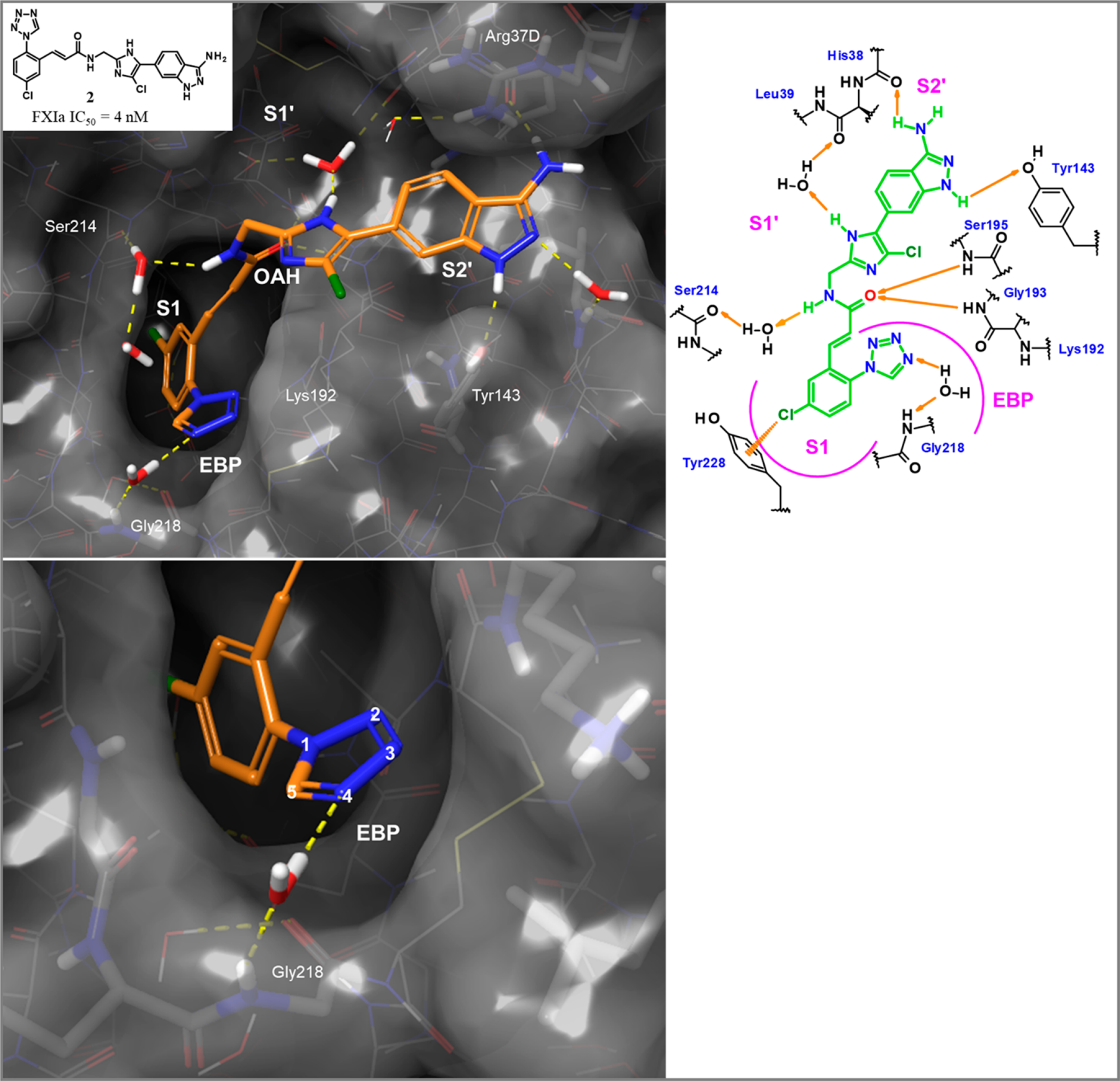
X-ray cocrystal structure of compound **2**(28) in complex with human FXIa (PDB code 8BO6) and the corresponding 2D sketch. The magnification shows the S1 site occupied by the chloroaryl residue and the interactions of the neutral tetrazole in the EBP site.

Neutral P1 groups such as chloroaryl, chlorothienyl, or methoxyaryl have been successfully exploited in the discovery of oral inhibitors of thrombin^26^ and FXa.^27^ In our experience, whether serine proteases of the coagulation cascade (e.g., thrombin, FVIIa, FIXa, FXa, and FXIa) can be addressed by neutral P1 groups depends mainly on the amino acid at position 190. Thrombin and FXa possess an Ala190, flanking Asp189 at the bottom of the S1 pocket and offering no interaction sites with bulk water. Ligands with neutral, lipophilic P1 groups can expel the deeply buried water and release it into bulk solvents, which is associated with a huge gain in entropy. In contrast, serine proteases such as FVIIa and FIXa with Ser190 offer hydrogen bond acceptor and donor sites to the bulk water and directly bind that water molecule. Lipophilic residues alone are not sufficient to release the more tightly bound solvent molecules, and ligands require rather basic P1 groups for potent inhibition. As FXIa belongs to the Ala190 “class”, with an otherwise completely conserved S1 pocket compared to thrombin or FXa, we proposed at the start of our project that neutral P1 groups such as chloroaryl should also work in the design of our new FXIa inhibitors. This proposal was supported by compound **2** (FXIa IC_50_ = 4 nM) disclosed by Bristol Myers Squibb in 2008.^28^ The binding mode of this compound could be modeled based on the cocrystal structure of **1**, using a docking approach. Based on these data, we hypothesized that the chloroaryl residue would make similar contacts to Tyr228 as in homologous serine proteases and occupy the S1 pocket. In theory, the 3-aminoindazole moiety can be regarded as cyclized benzamidine and could have functioned as the P1 residue as well. Serine protease inhibitors carrying this P1 motive are known.^29^ The X-ray cocrystal structure of ligand **2** in complex with human FXIa showed that a basic P1 binder as in **1** can be replaced by a nonbasic chloroaryl moiety. The chlorine substituent forms an edge-to-face interaction with Tyr228 at the bottom of the S1 pocket (Figure 2).

### Hit Finding

**Figure 3:**
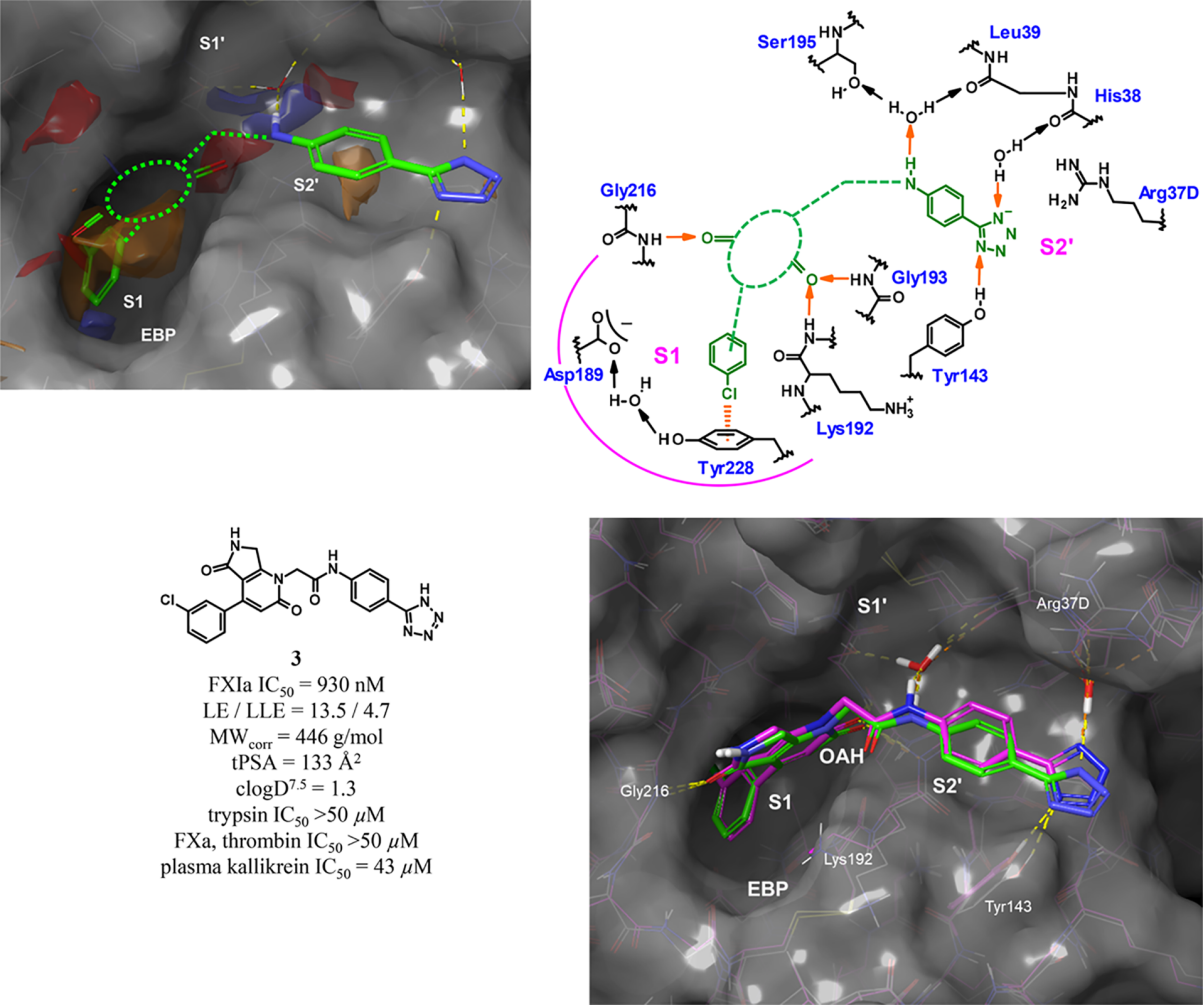
Structure-based *de novo* design concept leading to hit **3** and comparison of the docked compound (magenta, pre-synthesis) and the X-ray cocrystal structure in complex with human FXIa (green, PDB code 8BO5) after experimental verification.

In 2010, based on our in-house X-ray cocrystal structure of compound **1** in complex with human FXIa (Figure 1), and the shape and properties of the FXIa binding pocket, we defined for our program the most important interactions which an oral FXIa inhibitor should fulfill: (1) most importantly, the S1 pocket should be occupied by a lipophilic aromatic moiety (preferably a chloroaryl substituent), (2) the oxyanion hole (OAH) should be occupied by a carbonyl group being able to form one or two strong hydrogen bonds with the backbone NH groups of Lys192 and Gly193, (3) the solvent-exposed backbone NH of Gly216 at the rim of the S1 pocket should be contacted by a strong hydrogen bond acceptor, (4) a trapped water molecule (between Leu39 and Ser195) functioning as an indirect hydrogen bond acceptor on the protein side should be contacted by a strong hydrogen bond donor, such as an NH group, on the ligand side, and (5) a lipophilic/aromatic spacer carrying a neutral or acidic functional group that is capable of accepting two hydrogen bonds (from Tyr143 and another trapped water) should be present (Figure 3). Since this design concept involved a relatively high number of polar functional groups, we aimed to connect them with primarily lipophilic spacers.

We then *de novo* designed a novel chemotype that we hypothesized would allow for all of these interactions. The design was based on the previously undescribed idea of incorporating the central pharmacophoric elements into one heteroaromatic core, allowing decoration with substituents carrying the above-noted functional groups. Various different aromatic cores were enumerated, docked into the active site, and energy-minimized. Besides the steric and electrostatic complementarity, synthetic accessibility of a similar core that was previously used in an unrelated, internal Bayer AG project^30^ played a role in the final selection for the synthesis of the first compound. This structure-based *de novo* design approach resulted in hit **3** with micromolar potency in FXIa inhibition and favorable selectivity versus other serine proteases such as thrombin, FXa, and especially important, trypsin (all IC_50_ values >50 μM). The subsequent comparison of the (pre-synthesis) docked complex versus the X-ray cocrystal structure of ligand **3** in complex with human FXIa confirmed our design concept (Figure 3). Since **3** already had favorable physicochemical properties, we had fortunately achieved a promising hit with a novel chemotype with the synthesis of just one compound, which we could then use as the starting point for further modification.

### Lead Finding

**Figure 4:**
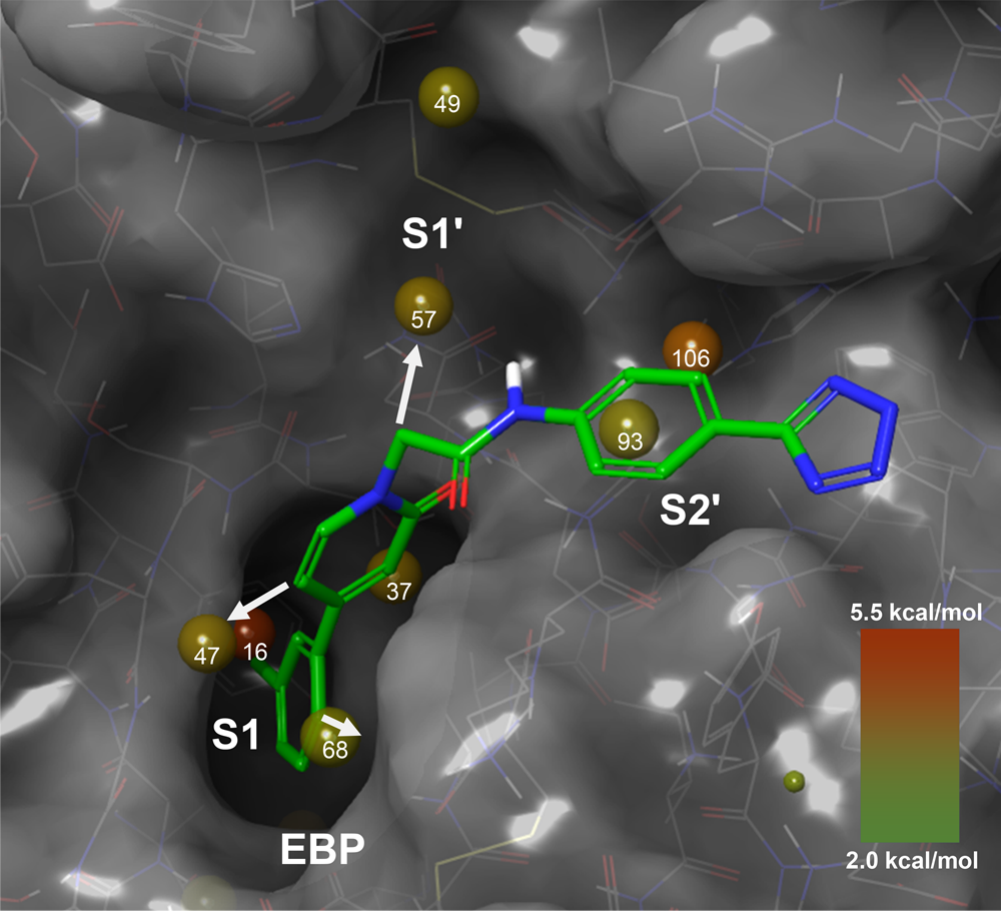
WaterMap hydration sites in the substrate binding pocket of FXIa (PDB code 8BO5). Sites are color- and size-coded with regard to the modeled hydration free energy (high Δ*G*, reddish; low Δ*G* greenish). The depicted compound in green is the basic framework of the lead series; the white arrows show the possibilities for substitutions to increase interactions with FXIa.

The subsequent lead finding was, among other aspects, guided by consequent considerations of physicochemical properties like clogD^7.5^,^31^ corrected molecular weight (MW_corr_),^32^ topological polar surface area (tPSA),^33^ ligand efficiency (LE)^34^ (with MW_corr_), and ligand lipophilicity efficiency (LLE)^35^ (with clogD^7.5^), as well as being design-driven by WaterMap,^36^ a molecular dynamics approach characterizing solvent structure and energetics within a ligand binding pocket. The system interaction energy and excess entropy for each water molecule in the substrate binding pocket are calculated using inhomogeneous solvation theory.^37^ In principle, the less energetically (enthalpically and/or entropically) favorable expelled water molecules are, the more favorable is their contribution to the binding free energy if replaced. As shown in Figure 4, the energetically most favorable hydration sites with the color-coded modeled highest free energy (Δ*G*) upon dis- or replacement are shown (for detailed values, see Figure S1, Supporting Information). The analysis indicated that the energetically least favored water molecules reside in the S1 pocket (site 16, Δ*G* = 5.2 kcal/mol and site 37, Δ*G* = 3.2 kcal/mol), followed by the S2′ pocket (site 106, Δ*G* = 4.0 kcal/mol) and the S1′ pocket (site 57, Δ*G* = 3.1 kcal/mol). Other important hydration sites are located at the rim of the S1 pocket (site 47, Δ*G* = 3.0 kcal/mol), the distant part of the S1′ pocket (site 49, Δ*G* = 2.8 kcal/mol), and in the EBP (site 68, Δ*G* = 2.6 kcal/mol). Our internal analysis led us to the conclusion that further hydration sites lie between the EBP and the S2′ site and the S3′ site but cannot be addressed with ligands of molecular weight compatible with good absorption properties. It is worth noting that in FXIa, the S2 pocket is so shallow that it cannot be detected by this computational/protein structural analysis as such.

Compound **3** displaces water molecules corresponding to sites 16, 37, 106, 93, and 47. Aiming to improve the potency of our hit **3**, we first concentrated on adding additional substituents to displace energetically unfavorable water molecules in the EBP and S1′ pocket. The aforementioned WaterMap calculations helped to guide our structural modifications.

We hypothesized that an additional ortho substitution at the P1 chloroaryl moiety could further improve potency by filling the EBP (and thus displacing an energetically unfavorable water molecule, site 68) and by conformationally preorganizing the dihedral angle between the pyridinone and chloroaryl ring. We proposed that this angle, which was derived from the X-ray complex of FXIa with ligand **3**, should ideally be approximately −66° to fit the S1 pocket with a minimal entropy loss upon binding. To facilitate synthetic access, we first considered a series of simplified nonannulated pyridinones. We aimed to explore the EBP with relatively small, not too polar substituents, composed of one or two non-hydrogen atoms. As a quick decision criterion, we looked at the difference of the dihedral angle between the pyridinone and P1 aryl moiety in the active conformation (energy-minimized in the FXIa binding site) and the unbound ligand in water and the steric filling of the EBP. We hypothesized that the smaller this difference, the more preformed the compounds would be, having a lower entropy penalty upon binding. Energy calculations were carried out with the OPLS2.0 force field^38^ and implicit continuum solvent model for water. Virtual derivatives were enumerated, docked, and prioritized. Cyano as a substituent was clearly favored as it showed an excellent fit in the EBP and a relatively flat torsion profile with a still not ideal energy minimum at around −43° in the unbound energy-minimized state. Several derivatives (like compounds **5**–**7**) were synthesized and, indeed, an *ortho*-cyano group turned out to be a local optimum among smaller ortho substituents (). The cocrystal structure (data not shown) for compound **7** in complex with human FXIa revealed a torsion angle of 68° of the P1 phenylpyridinone moiety, with only Lys192 giving way to the larger CN group in comparison to **3** and enlarging the EBP.

**Table 1:**
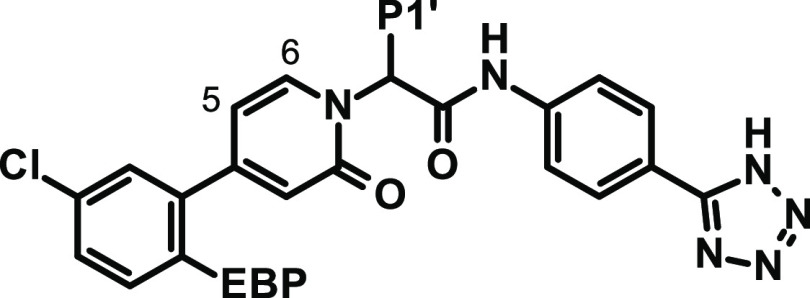

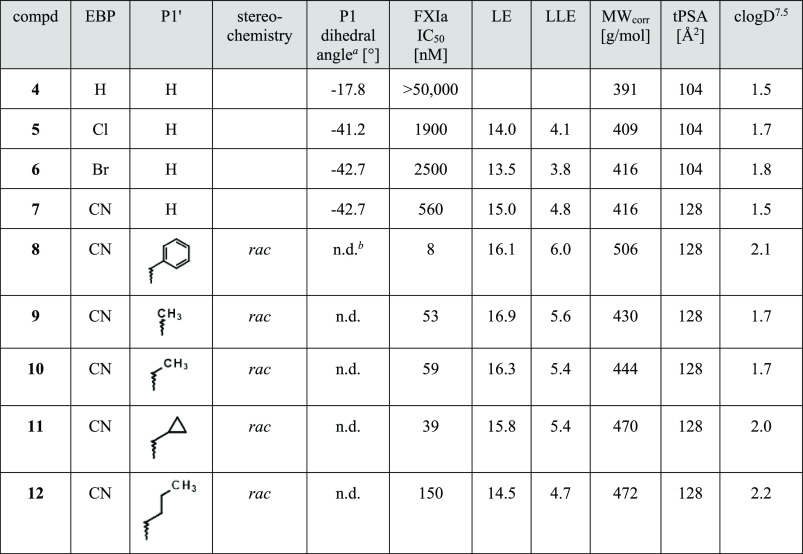
Modification of EBP and P1′ Substitution

a The P1 dihedral angle from force field calculations with implicit water is shown to monitor the progress in adjusting an ideal angle around 66° derived from the X-ray complex of FXIa with ligand **3**.

b n.d.: not determined.

The inactivity (>50 μM) of the unsubstituted derivative **4** is interesting and shows the importance of conformational preformation and rigidification in that series. Had this derivative, fulfilling 80% (4 out of 5) of the pharmacophoric requirements (Figure 3, see *de novo* strategy outlined above), been synthesized and tested first, without recognizing the importance of the dihedral angle (P1 phenyl/pyridinone, −18°), namely, the presence of at least one appropriate *ortho* substituent and the conformational prearrangement, the project would have taken a different turn.

Following the guidance from the WaterMap calculations and a superimposition of the chemically distinct compound **1**, we speculated that a larger lipophilic substituent protruding toward the S1′ pocket would displace energetically unfavorable water molecules and lead to an increase in binding affinity. The first derivative designed to explore this pocket was compound **8** carrying an additional benzyl group. The observed FXIa IC_50_ value of 8 nM for the racemate exceeded our expectations (Table 1). Parts of the 70-fold affinity increase (compared to unsubstituted **7**) were speculated to arise from the rigidification of the flexible hinge in the middle part of the molecule. The additional stereocenter leads to a productive conformational prearrangement. We hypothesized from this analysis that a single methyl group (P1′ substituent, see Table 1) would also lead to a significant potency gain without occupying the S1′ pocket. The said methyl derivative **9** indeed displayed a strong increase in IC_50_ (53 nM) compared to **7**. This was an important finding since with two derivatives, the potency of the compound class was increased by more than 10-fold with a minimal increase in molecular weight and lipophilicity. Additional derivatives (like compounds **10** and **11**) with a sterically more demanding P1′ residue showed a general trend of increasing affinity with increasing size, which is compatible with the water displacement theory. The *n*-butyl derivative **12** does not obey that general trend, which may be explained by higher flexibility and a relatively high gain in entropy upon binding as a potential reason for the somewhat lower measured binding affinity.

The next step in the design of novel FXIa inhibitors would be the combination of the annulated cyclopentanone (cf. compound **3**) and the *ortho*-cyano P1 moiety. However, since the cyclopentanone moiety (especially the pyridinone C6 substituent) was hypothesized to lead to an intramolecular steric clash with a P1′ substituent that was considered necessary to achieve high potency, such a structural modification was deprioritized at that point in time.^39^

Instead, we chose to further explore the nonannulated pyridinone as the central core and to address the important interaction with the backbone Gly216:NH with a single substituent at the C5 position. We were looking for C5 substituents that lead to a dihedral angle of around −66° and ideally act as a hydrogen bond acceptor to form strong interactions with Gly216. For P2′, we changed the acidic function from tetrazole to carboxylate. From structurally related matched pairs, we could see the trend that Caco-2 permeation was better here. Combinations of various C5 substituents with different EBP residues were enumerated, characterized, and ranked in silico. Compound **15** with a C5-chloro substituent, allowing for a favorable dihedral angle of −59.8°, was prepared and led to 6-fold improved potency (FXIa IC_50_ = 27 nM) compared to C5-unsubstituted compound **13** (Table 2). This was especially promising as the C5-chloro substituent would not form a hydrogen bond and the potency increase was primarily arising from the conformational prearrangement compared to the C5-unsubstituted compound. Based on our work, we anticipated that C5-methoxy substitution would be highly beneficial, as we designed it to not only adopt a more favorable dihedral angle (energy minimum of unbound compound at −54°) but also form an additional hydrogen bond with the backbone NH of Gly216. Indeed, the resulting compound **19** showed a 29-fold improved potency (FXIa IC_50_ = 5.9 nM, racemic mixture) relative to **13**. We observed that sterically more demanding EBP substituents than cyano, such as trifluoromethyl (**18**) versus difluoromethyl (**17**) and trifluoromethoxy (**21**) versus difluoromethoxy (**20**), can result in significantly less potent compounds (Table 2), emphasizing the importance of choosing the right combination of both EBP and C5-pyridinone substitution.

**Table 2:**
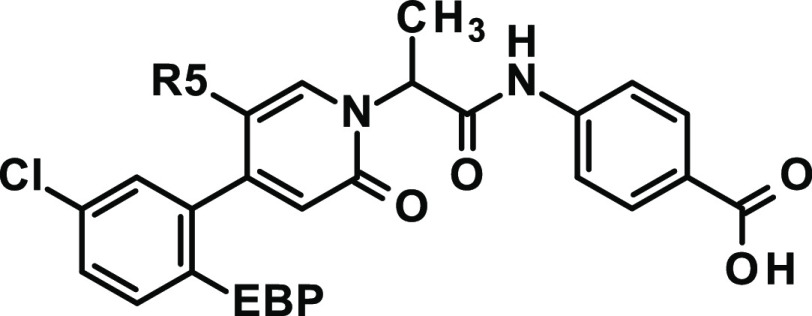
Modification of EBP and C5 Substitution (R5)

compd	EBP	R5	stereochemistry	P1 dihedral angle [°]	FXIa IC_50_ [nM]	LE	LLE	MW_corr_ [g/mol]	tPSA [Å^2^]	clogD^7.5^
**13**	CN	H	*rac*	–42.7	170	16.7	5.2	406	111	1.6
**14**	CN	F	*rac*	–51.4	74	17.4	5.5	410	111	1.7
**15**	CN	Cl	*rac*	–59.8	27	17.9	5.8	424	111	1.8
**16**	CN	CN	*rac*	–57.3	42	17.1	5.8	431	134	1.6
**17**	CHF_2_	CN	*rac*	–61.6	43	17.2	5.6	428	111	1.8
**18**	CF_3_	CN	*rac*	–127.2	470	14.6	4.4	432	111	2.0
**19**	CN	OCH_3_	*rac*	–53.6	5.9	18.9	6.6	436	120	1.6
**20**	OCHF_2_	OCH_3_	*rac*	–46.2	10	17.8	6.2	449	105	1.8
**21**	OCF_3_	OCH_3_	*rac*	–46.0	46	16.2	5.2	453	105	2.1
**22**	CN	CHF_2_	*rac*	–64.8	630	14.5	4.4	428	111	1.8
**23**	CN	CF_3_	*rac*	–120.6	3500	12.6	3.6	432	111	1.9

Enantiomerically pure compound **24** already showed good potency (FXIa IC_50_ = 2 nM, Table 3) and excellent properties such as low molecular weight and balanced lipophilicity (clogD^7.5^ = 1.6). However, the compound had insufficient anticoagulant activity in human plasma (aPTT EC_150_ = 10.8 μM, the inhibitor concentration needed for 1.5-fold prolongation of plasma clotting time).

**Table 3:**
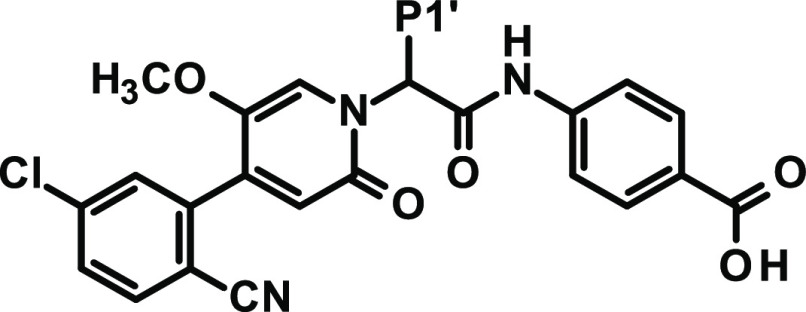

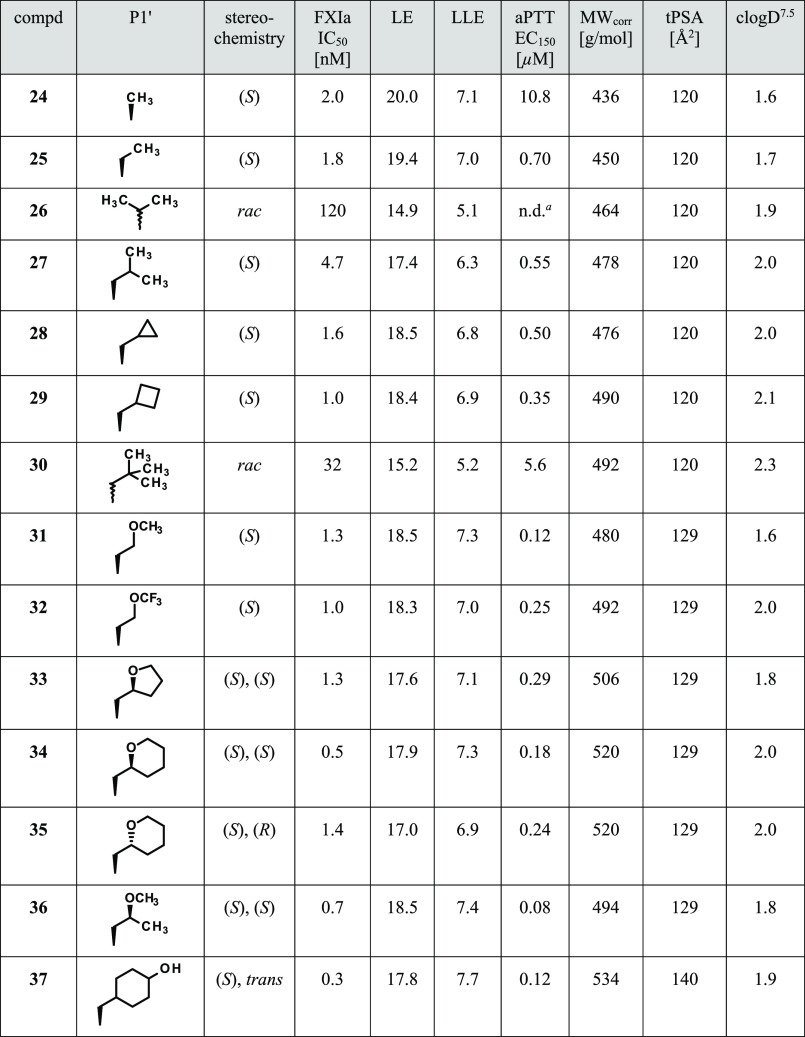
Modification of P1′ Substitution

a n.d.: not determined.

Modification of the P1′ group from methyl to ethyl led to our lead **25** with good potency (FXIa IC_50_ = 1.8 nM) as well as a 15-fold improved anticoagulant activity in human plasma (aPTT EC_150_ = 0.70 μM). Lead **25** had a favorable selectivity profile (e.g., on trypsin, IC_50_ > 50 μM) and showed convincing physicochemical properties (MW_corr_, clogD^7.5^, tPSA) along with good oral absorption in rats (bioavailability of 67%, see Table 7).

### Initial Lead Modification

**Figure 5:**
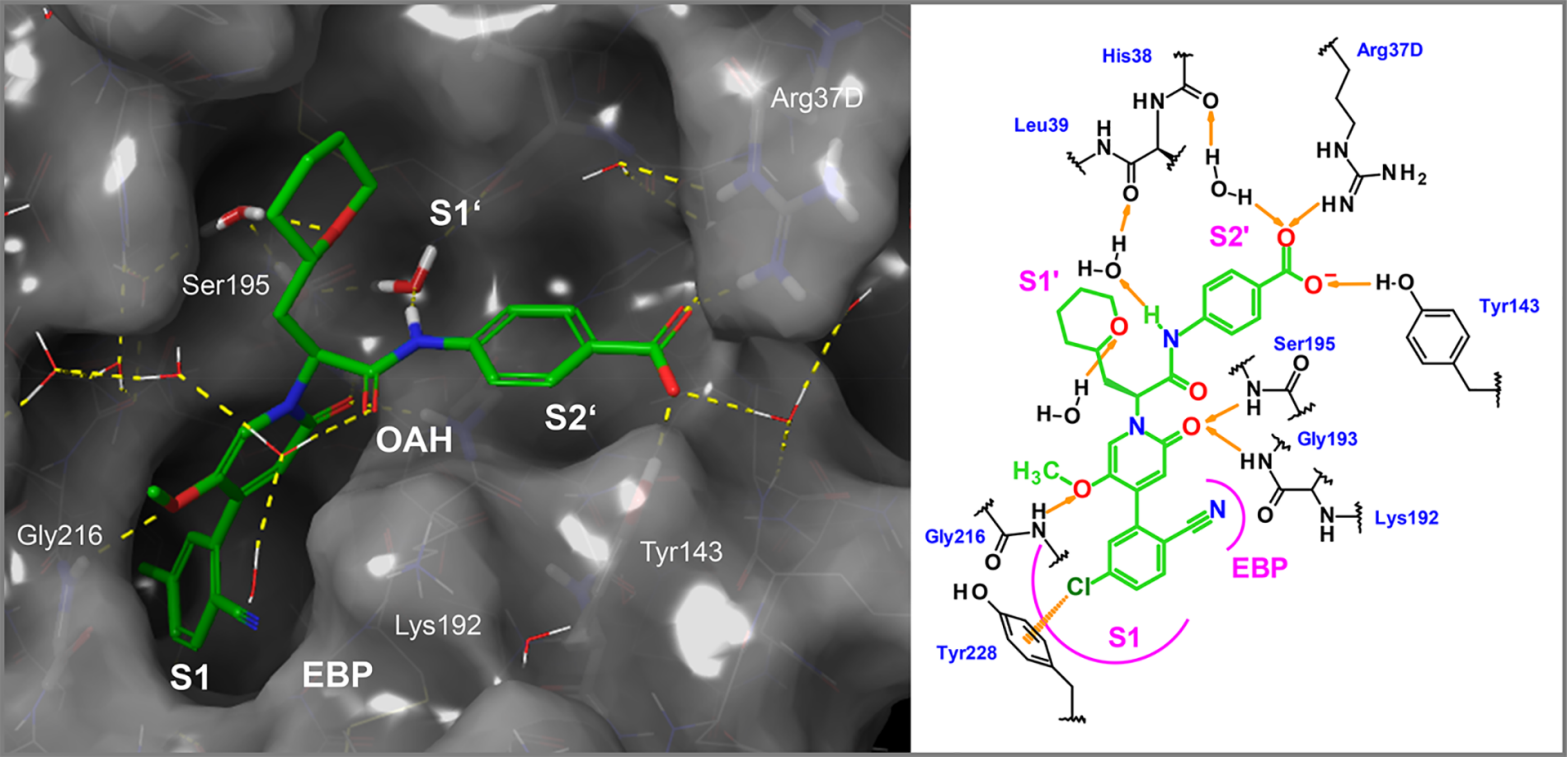
X-ray cocrystal structure of compound **34** in complex with human FXIa (PDB code 8BO7) and the corresponding 2D sketch.

The goal of the initial lead modification was to increase potency, specifically, to increase the anticoagulant activity of the compound series significantly, without changing the favorable physicochemical properties excessively. Following the WaterMap calculations (Figure 4) and our initial design of substituents in the S1′ pocket, we explored suitable P1′ groups in more depth (Table 3). Direct branching by extending the P1′ ethyl group to isopropyl was unfavorable (compound **26**). Both FXIa potency and anticoagulant activity improved going from isopropylmethyl (**27**) to cyclopropylmethyl (**28**) and then to cyclobutylmethyl (**29**). Additional terminal branching like *tert*-butylmethyl (**30**) resulted in loss of potency. We observed a gain in anticoagulant activity upon the introduction of a methoxyethyl substituent, namely, compound **31** (human aPTT EC_150_ = 0.12 μM). Based on the limited potency previously obtained for the P1′ *n*-butyl derivative **12** (Table 1), this was surprising and appeared to result from an additional hydrogen bond interaction of the ether oxygen with a water molecule, held in place by Ser195. This hydrogen bond was subsequently observed in a cocrystal structure with one of the later described derivatives (**34**). By integrating the P1′ methoxyethyl into a five- or six-membered aliphatic ring, we focused our activities on cyclic ethers due to their balanced physicochemical properties and potentially improved solubility compared to, for example, a benzyl substituent. This resulted in highly potent tetrahydropyran **34** (FXIa IC_50_ = 0.5 nM, human aPTT EC_150_ = 0.18 μM). From the X-ray cocrystal structure of **34** in complex with human FXIa, the absolute stereochemistry of both chiral centers was determined to be the (*S*)-configuration. The additional interaction between a water molecule, binding to the hydroxy functions of Ser195 and the ether oxygen of the P1′ tetrahydropyran, is nicely revealed (Figure 5). Molecular-dynamics-based relative binding free energy calculations later confirmed the choice of (*S*)-tetrahydropyran within the typically observed error range (calculated Δ*G* = −12.21 ± 0.21 kcal/mol versus experimental Δ*G* = −12.77 kcal/mol). Further efforts to reduce the number of carbon atoms resulted in the design of methyl-substituted **36** [preferred isomer: (*S*)-Me, FXIa IC_50_ = 0.7 nM, human aPTT EC_150_ = 0.08 μM] which can be regarded as a seco-pyran derivative. The P1′ 4-hydroxycyclohexylmethyl residue was designed to replace an energetically unfavorable water molecule (site 49, Figure 4) and form an additional hydrogen bond with the backbone carbonyl oxygen of Cys58, and derivative **37** marked one of the most potent compounds (FXIa IC_50_ = 0.3 nM, human aPTT EC_150_ = 0.12 μM) but lacked Caco-2 permeation and in vivo metabolic stability. Also, in binding free energy calculations, **37** showed high potency with a calculated Δ*G* of −13.69 ± 0.30 kcal/mol (experimental Δ*G* = −13.07 kcal/mol).

### Characterization of Carboxylic Acid **34**

At that point in the project, carboxylic acid **34** was regarded as the best compound overall and was profiled in depth: **34** inhibited human FXIa directly, reversibly, potently, and selectively with an IC_50_ of 0.5 nM in buffer. Plasma kallikrein, the protease closest related to FXIa, was inhibited with an IC_50_ of 4.9 nM. Compound **34** showed selectivity for FXIa inhibition versus other proteases, especially of the hemostatic system, including thrombin, FXa, FVIIa, FIXa, plasmin, tissue plasminogen activator, and trypsin (all inhibited at a more than 1000-fold lower potency). While the 50% clotting time prolongation (EC_150_) in the aPTT assay during the compound screening phase was 0.18 μM (final assay concentration as described in the Supporting Information, corresponding to 0.54 μM in plasma), the in-depth characterization with repeated measurements yielded an EC_150_ of 0.25 μM in human plasma. In plasma samples of various other species, compound **34** prolonged the clotting time as well, resulting in an aPTT EC_150_ of 2.8 μM (rabbit), 0.28 μM (dog), and 0.25 μM (pig). However, no clotting time prolongations were observed in mouse or rat plasma (aPTT EC_150_ > 30 μM).

The effect of **34** on thrombus formation in vivo was evaluated in ferrous-chloride-induced injury models at the rabbit carotid artery or jugular vein. When administered prophylactically by intravenous injection, **34** reduced the thrombus weight dose-dependently in both models without interfering with hemostasis, which was checked simultaneously by measuring the ear bleeding time (see Figure 8A–E). Ex vivo experiments in plasma of the respective animals demonstrated a close correlation between the aPTT and the thrombus weight reduction. Oral administration of a solution of 10 or 20 mg/kg **34** in a PEG/ethanol/water vehicle to rabbits resulted in 16 and 39% reduction of thrombus weight, respectively, in a ferrous chloride-induced injury model (see Figure 8F).

The P2′ carboxylic acid moiety turned out to be favorable for a noncritical drug–drug interaction (DDI) profile with respect to inhibition and induction of CYP3A4. Despite this carboxylic acid, we observed glucuronidation as only a minor pathway in human hepatocytes, relatively high free fractions (fraction unbound, *f*_u_) in animal species (13–15%) and still moderate *f*_u_ in man (5%), and moreover, moderate to low in vivo clearance (CL) and moderate oral bioavailability (53%/55%) in rats and dogs (see Tables 7 and 8).

With additional favorable results in preclinical safety studies, **34** was chosen for evaluation in a first-in-human study. In this trial, however, **34** turned out to have lower oral exposure and a shorter dominant half-life in humans than expected. Based on data from animal studies, we apparently had underestimated the more complex absorption behavior of **34** in humans and the extent of transport via P-gp and OATP, most probably due to the carboxylic acid moiety.

### Modification of P2′ Substitution

We further evaluated a broad range of compounds with P2′ carboxylic acids, benzoic acids, and several other derivatives (e.g., acids **38**–**40**, Table 4), but it became evident to us from additional in vivo PK experiments that nonacidic P2′ moieties displayed longer half-lives and lower clearances than acidic P2′ moieties and therefore could be more promising for improving the PK profile in humans.

**Table 4:**
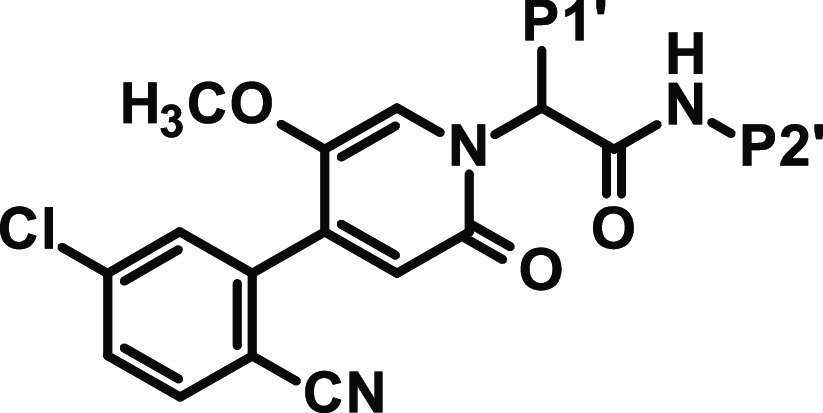

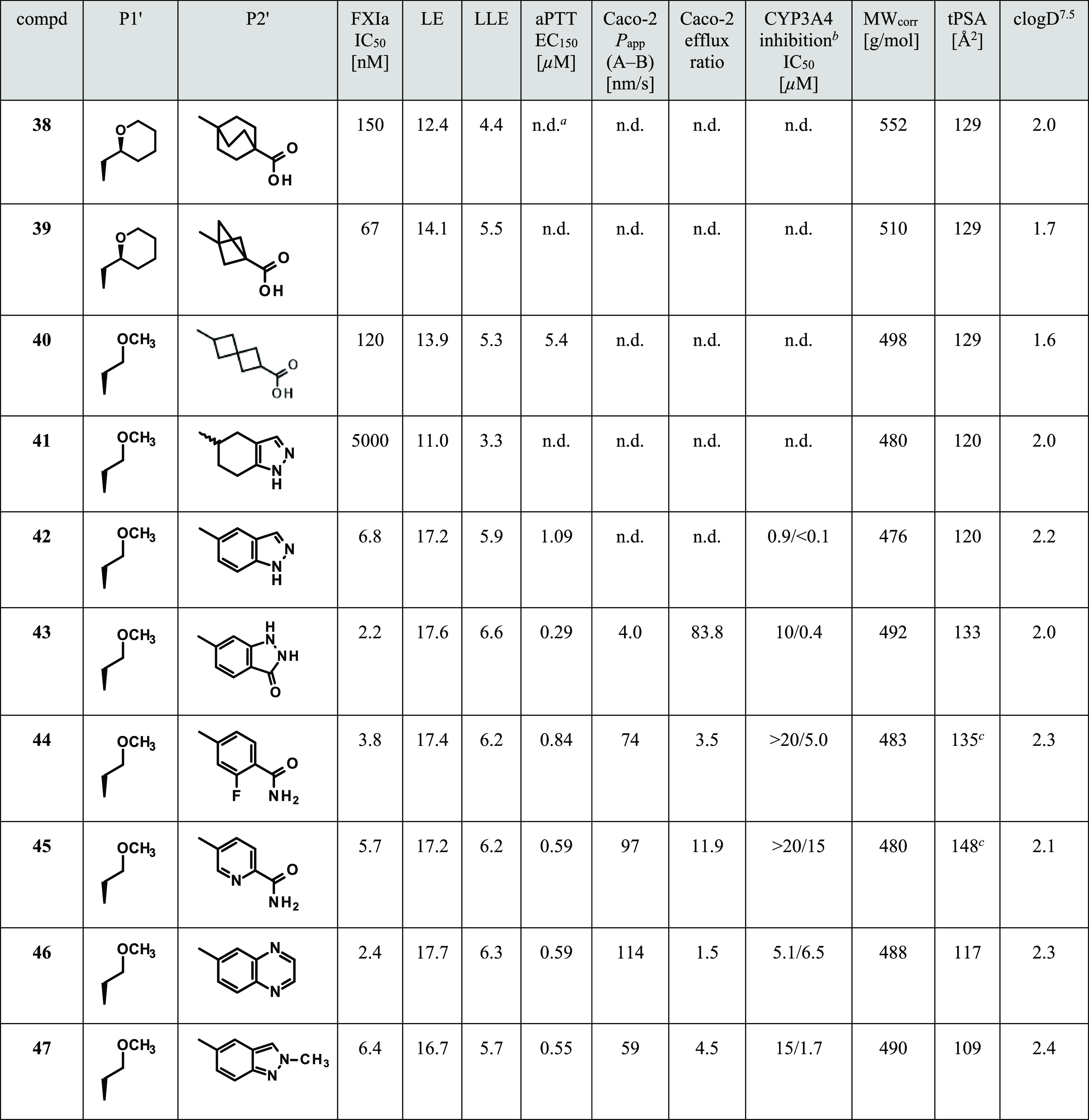
Modification of P2′ Substitution

a n.d.: not determined.

b CYP3A4 inhibition: IC_50_ without preincubation/IC_50_ after 30 min preincubation.

c tPSA value does not reflect the actual polar surface area as the algorithm simply sums up the polar surface of precalculated functional groups and does not take into account intramolecular hydrogen bonds.

We did not identify a nonaromatic P2′ group leading to compounds with sufficient potency (e.g., **41** is less potent than **42**, Table 4). Moreover, the NH of the central amide bond is essential for the water-mediated interaction with Leu39 (Figure 5) so that, for example, cyclization to circumvent a secondary amide was not an option. Therefore, we had to stick with the resulting secondary anilide structure having the potential to be cleaved by metabolism with liberation of an aniline derivative. To avoid potentially genotoxic metabolites, only Ames negative anilines (like 4-aminobenzoic acid) qualified for use, and we evaluated those by GLP Ames screening studies. Any P2′ group had to fulfill the complex interaction pattern (addressing His38, Arg37D, and Tyr143 concurrently) which is necessary as anchor in the S2′ pocket for high FXIa affinity but further restricts the selection pool of aromatic amines substantially.

With these prerequisites in mind, we evaluated a range of nonacidic P2′ groups. The selection of P2′ groups for synthesis was supported by binding free energy calculations using FEP+.^40^ Compared to the corresponding carboxylic acids, the nonacidic P2′ derivatives were overall less potent in FXIa inhibition and showed less anticoagulant activity. This is rationalized by the missing ionic interaction of the acidic moiety with Arg37D. Nonacidic P2′ groups leading to more potent compounds often required several hydrogen bond donors/acceptors resulting in compounds with low Caco-2 permeation and insufficient exposure after oral administration, correlating with high tPSA values. Moreover, many of these derivatives showed critical inhibition of CYP isoforms. These overall issues hold true for many derivatives with different P1′ groups, and a few examples comparable with carboxylic acid **31** are shown in Table 4.

Dihydroindazolone **43** was designed to interact via its aromatic NH group with the His38 backbone carbonyl and replace a bridging structural water always observed for the P2′ benzoic acids. It was hypothesized that the dihydroindazolone carbonyl function would accept a hydrogen bond from the hydroxy function of Tyr143. Compound **43** displayed good potency but, matching its high tPSA value of 133 Å^2^, it showed almost no permeation in the Caco-2 assay and a very high efflux ratio. To try to improve permeation, we aimed for carbamoyl substituents where one hydrogen of the NH_2_ functionality could be involved in an intramolecular hydrogen bond. Carbamoyl derivatives **44** and **45** were indeed reasonably permeable, as the *ortho*-fluoro group or *ortho*-pyridinyl nitrogen apparently ended up masking one of the highly polar carbamoyl hydrogens. Quinoxalinyl amide **46** and substituted indazol-5-yl amide **47** with reasonable potency showed more favorable Caco-2 permeation due to lower tPSA values. However, CYP3A4 inhibition and moreover time-dependent inhibition (TDI) emerged as further parameters for closer consideration in the course of our project. For many nonacidic P2′ derivatives, we still observed high in vivo CL in rat, unfortunately not closely correlating with in vitro CL in rat hepatocytes.

### Reassessment of P1′ Substitution

To try to improve metabolic stability, we investigated a diverse set of derivatives with different P1′ substituents more closely for their in vitro metabolism in rat and human hepatocytes. The P1′ residue turned out to be the main soft spot for metabolism for many compounds. Therefore, our amended strategy to achieve higher metabolic stability was to keep P1′ groups rather small and minimize the number of sites for metabolic attack; for example, P1′ groups like ethyl were preferred.

### Reassessment of EBP Substitution

Derived from in vivo pharmacology studies, our potency target for a single stereoisomer was inhibition of FXIa activity with a low single-digit nanomolar IC_50_ and prolongation of plasma clotting time in the aPTT assay with an EC_150_ of less than 0.5 μM in the final volume of the assay (as described in the Experimental Section). As potency was lost by abandoning charged P2′ carboxylic acids and extended P1′ alkyl ether groups, we needed to identify other options to regain potency. Essentially, the only pocket left for further experimentation was the EBP. The WaterMap analysis (Figure 4) showed an energetically unfavorable water molecule (site 68) that could be displaced by larger substituents to gain potency. In addition, previously described compounds having a five-membered heteroaromatic ring as EBP moiety^41,42^ have shown to be highly potent. Especially, an uncharged N-substituted tetrazole led to highly potent compounds. However, in our series, such derivatives were associated with low permeability and unfavorably high efflux ratios in the Caco-2 assay, explainable by the increased tPSA values (e.g., see compounds **54** and **59**, Table 5).

**Table 5:**
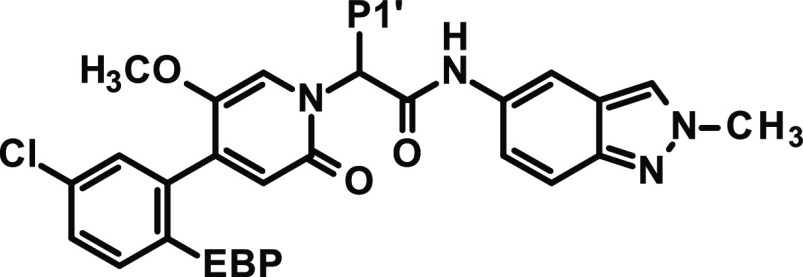

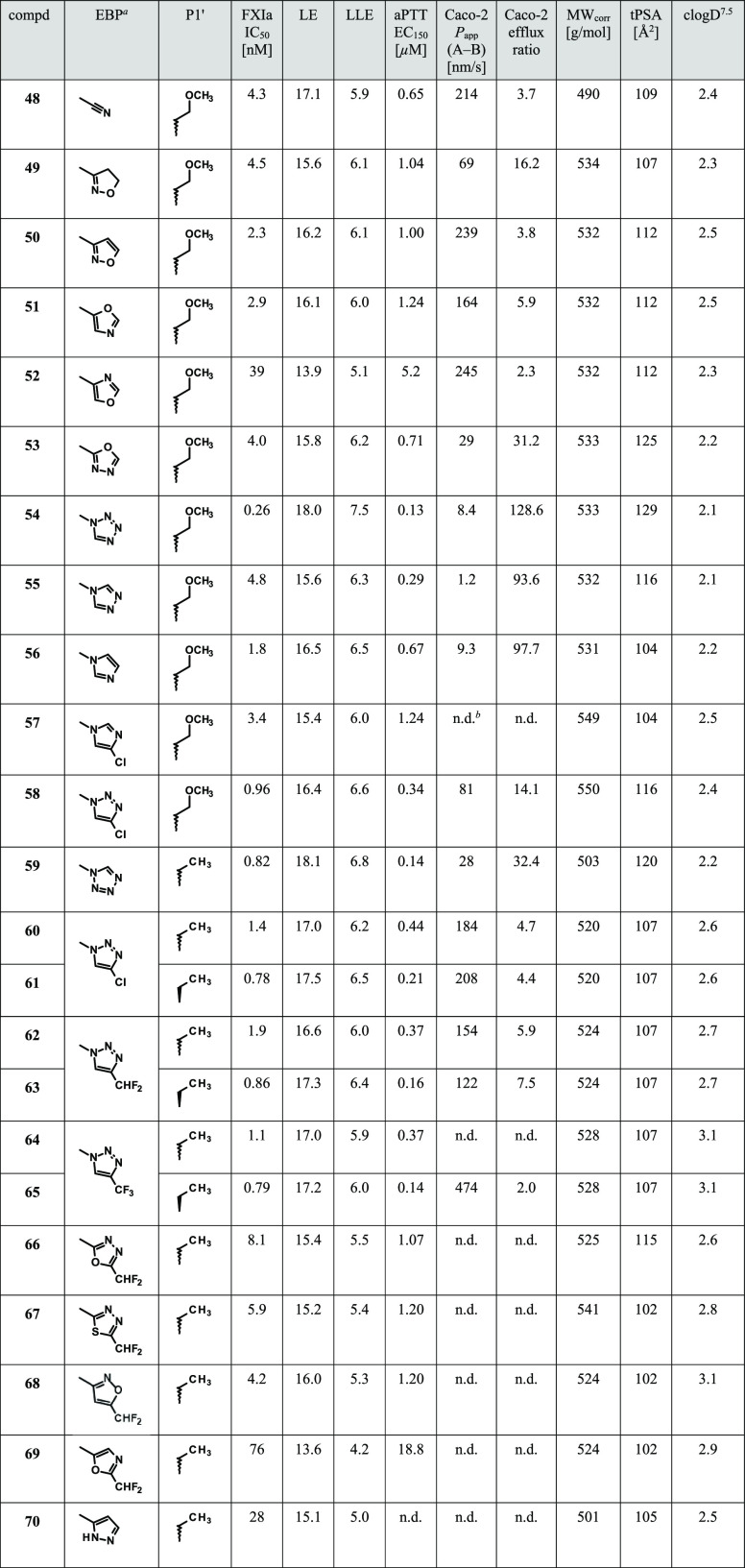
Modification of EBP Substitution

a The heteroaromatic EBP substituents are drawn corresponding to the supposed orientation with FXIa, see Figure 2.

b n.d.: not determined.

Therefore, we evaluated a set of five-membered heteroaryl and heterocyclyl rings for EBP substitution. Figure 2 shows the details of the interaction of an EBP tetrazole residue with FXIa. The aromatic carbon (C5) points toward a small groove at the rim of the S1 pocket formed by Gly216, Glu217, and Gly218. N4 forms a water-mediated hydrogen bond with the Gly218:NH. N2 and N3 are surrounded by the aliphatic carbon side chain of Lys192. We aimed at exploring the role of the aromatic carbon (C5) and hydrogen bonding features of N4, with further substitution at position 4 to increase interactions and directly contact Gly218:NH via hydrogen bonds. We wanted to keep the tPSA low for increased permeation and to try to improve the interactions with FXIa. Results for a small set of compounds (with P1′ a methoxyethyl or ethyl residue and P2′ methylindazolyl as a standard) are shown in Table 5. Prioritization and selection of residues for synthesis were supported by relative binding free energy (FEP+) calculations using the OPLS2.1 force field^43^ (see the Supporting Information).

In a comparison of cyano-substituted compound **48** with 4,5-dihydro-1,2-oxazol-3-yl derivative **49** and 1,2-oxazol-3-yl derivative **50**, only the latter led to slightly increased potency.

The importance of the water-mediated hydrogen bond (tetrazole N4, see Figure 2) is further supported by the two regioisomeric oxazoles **51** and **52**. The nitrogen at (tetrazole)-position 4 is a significantly better hydrogen bond acceptor than oxygen. In addition, the polar isolated nitrogen at position 2 (Figure 2) is detrimental for binding as it is forced into a primarily lipophilic environment (Lys192 side-chain carbons). Both aspects together explain the 13-fold difference in potency between these two isomers.

1,3,4-Oxadiazol-2-yl derivative **53** is less potent than the corresponding oxazole **51**, forcing a nitrogen into a lipophilicity preferring surrounding (tetrazole C5, Figure 2). Imidazole **56** which possesses the minimum EBP pharmacophore features (five-membered heterocycle with one strong hydrogen bond accepting atom at position 4, see Figure 2) combined high potency with low tPSA. However, this compound exhibited low Caco-2 permeability and a high efflux ratio, and therefore was not pursued further. 1,2,4-Triazol-4-yl derivative **55** again lost affinity due to the lower hydrogen bonding acceptor strength of the two neighboring nitrogens (due to the interaction of the nitrogen lone pairs) in the aromatic ring.

Besides tetrazole, the best results regarding potency and anticoagulant activity were achieved with 1,2,3-triazol-1-yl groups with an electron-withdrawing substituent, such as chloro, difluoromethyl, or trifluoromethyl. The difluoro- or trifluoromethyl substituent is able to displace the structural water near the EBP observed in the complex of **2** with FXIa (see Figure 2) and form a hydrogen bond with one of the fluorine atoms and the backbone Gly218:NH. In contrast to their corresponding tetrazole, triazole derivatives **60**, **62**, and **64** showed significantly higher Caco-2 permeation and lower efflux ratios. 1,3,4-Oxadiazol-2-yl, 1,3,4-thiadiazol-2-yl, and 1,2-oxazol-3-yl groups with electron-withdrawing substitution (**66**–**68**) resulted in compounds with reasonable potency but insufficient anticoagulant activity (human aPTT EC_150_ > 1 μM), albeit as racemic mixtures. 1,3-Oxazol-5-yl derivative **69** is 26-fold less potent than the corresponding unsubstituted **51**. We hypothesized that the additional difluoromethyl substituent forms a hydrogen bond to Gly218:NH, thereby forcing the polar isolated aromatic nitrogen into a primarily lipophilic environment. In contrast, compound **51** can adopt a 180° flipped orientation presenting the nitrogen to a water-mediated hydrogen bond contact (see position N4 in the corresponding tetrazole, Figure 2). The corresponding oxadiazole derivative **66** is again more potent than **69**, apparently because the hydrogen bond acceptor strength and polarity of both aromatic nitrogens is reduced due their direct vicinity within the five-membered heterocycle. A similar effect is observed for the pair **57** and its corresponding triazole **58**, rendering the latter by over a factor of three more potent.

One hypothesis, that an acidic hydrogen (CH or NH) at position 5 (tetrazole C5, Figure 2) could lead to the formation of a direct hydrogen bond with the backbone carbonyl of Gly218 and thus an increased affinity, was disproved with compound **70**. The tetrazole C5 surrounding is best occupied with nonpolar ligand atoms.

We concluded from our study of the EBP moiety that none of the prepared alternative five-membered heteroaryl derivatives showed overall more convincing properties than the compounds with a 1,2,3-triazol-1-yl EBP residue.

### Reassessment of P2′ Substitution

Having identified chloro-, trifluoromethyl-, or difluoromethyl-substituted triazole as preferred EBP options and small alkyl substituents like methyl or ethyl as favorable P1′ options, we next returned to our preferred set of P2′ substitutions (Table 6).

**Table 6:**
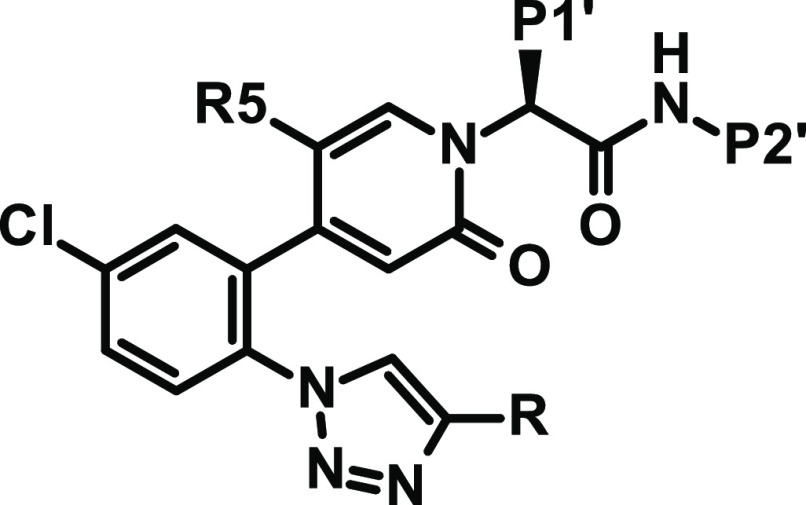

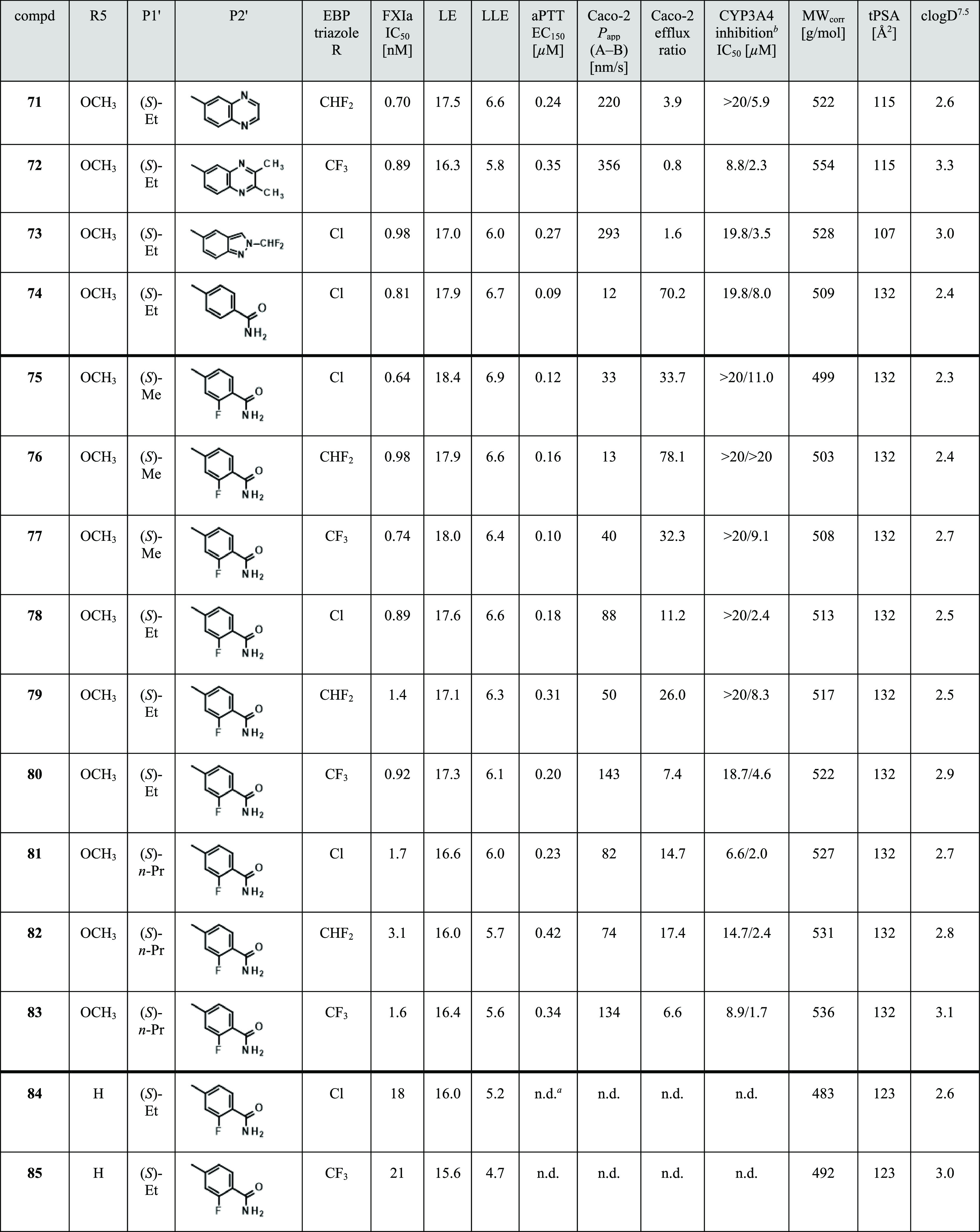
Candidate Selection

a n.d.: not determined.

b CYP3A4 inhibition: IC_50_ without preincubation/IC_50_ after 30 min preincubation.

Quinoxalinyl amide **71** is highly potent, with an FXIa IC_50_ of 0.7 nM and human aPTT EC_150_ of 0.24 μM. As the quinoxaline moiety is associated with a rather low increase in tPSA, **71** showed favorable Caco-2 permeation [*P*_app_(A–B) = 220 nm/s, tPSA = 115 Å^2^]. Coincubation with **71** did not affect CYP3A4 activity up to the highest test concentration (IC_50_ > 20 μM), but preincubation (30 min) of **71** with NADPH-supplemented recombinant CYP3A4 revealed an increase in the inhibitory potency of **71** toward CYP3A4 (IC_50_ = 5.9 μM), indicating time-dependent inhibition (TDI). However, relevant TDI of CYP3A4 activity in human hepatocytes was not observed. After intravenous administration in rat, **71** already showed a reasonable in vivo CL (Table 7). Aiming to further improve metabolic stability, we investigated the metabolism of **71** in hepatocytes of different species and learned that in human and monkey hepatocytes, additional metabolism by aldehyde oxidase (which shows low or no expression in rat and dog) occurred. Although we introduced additional substitution at the quinoxalinyl moiety to block metabolism by aldehyde oxidase (e.g., compound **72**), a compound with improved in vivo CL could not be identified. Moreover, genotoxicity studies revealed that quinoxalin-6-amine, a potential metabolite of amide **71**, is Ames positive.

**Table 7:** In Vivo Pharmacokinetic Properties of Selected Compounds in Rats (0.3 mg/kg iv bolus and 1.0 mg/kg po)

compd	AUC_norm_^iv^ [kg·h/L]	CL [L/h/kg]	*V*_ss_ [L/kg]	MRT_iv_ [h]	AUC_norm_^po^ [kg·h/L]	*F*^a^ [%]	*f*_u_ [%]
							rat/human/rabbit
**25**	1.3	0.79	1.1	2.1	0.87	67	9.1/4.5/8.6
**34**	1.0	0.98	1.8	1.9	0.53	53	15/5.2/14
**71**	1.5	0.67	0.98	1.5	0.67	42	n.d.^b^
**75**	n.d.	n.d.	n.d.	n.d.	0.46	n.d.	n.d.
**76**	n.d.	n.d.	n.d.	n.d.	0.45	n.d.	n.d.
**77**	n.d.	n.d.	n.d.	n.d.	0.84	n.d.	n.d.
**78**	2.0	0.51	0.83	1.6	1.1	53	2.2/7.0/16
**79**	n.d.	n.d.	n.d.	n.d.	1.3	n.d.	n.d.
**80**	2.2	0.46	0.76	2.5	1.3	60	2.4/6.4/14
**81**	1.7	0.58	1.0	1.8	0.56	33	n.d.
**82**	1.2	0.83	1.5	1.8	0.72	60	n.d.
**83**	2.2	0.45	1.4	3.1	0.87	40	n.d.

a Oral bioavailability.

b n.d.: not determined.

Difluoromethyl-substituted indazole **73** is also highly potent (FXIa IC_50_ = 0.98 nM, human aPTT EC_150_ = 0.27 μM) with favorable Caco-2 permeation [*P*_app_(A–B) = 293 nm/s, tPSA = 107 Å^2^]. Again, we observed a hint of TDI after preincubation of **73** with NADPH-supplemented recombinant CYP3A4 (IC_50_ = 3.5 μM) but in this case, irreversible and strong TDI of CYP3A4 activity in human hepatocytes was observed. Moreover, as 2-methyl-2*H*-indazol-5-amine turned out to be Ames positive, such indazoles as P2′ moieties were discontinued.

Three more fragments as options for P2′ substitution, 5-aminopyridine-2-carboxamide, 4-aminobenzamide, and 4-amino-2-fluorobenzamide, were studied for their genotoxicity profile and the latter two proved to be Ames negative.

Compared to its des-fluoro analogue **74** in the Caco-2 assay, compound **78** showed a significantly higher *P*_app_(A–B) value (88 vs 12 nm/s) and lower efflux ratio (11 vs 70). This difference could also be confirmed by rat in vivo exposure studies using oral administration, emphasizing the favorable impact of this *ortho*-fluoro substitution by masking one *N*-hydrogen of the carbamoyl group due to the formation of an intramolecular hydrogen bond.

### Candidate Selection

**Figure 6:**
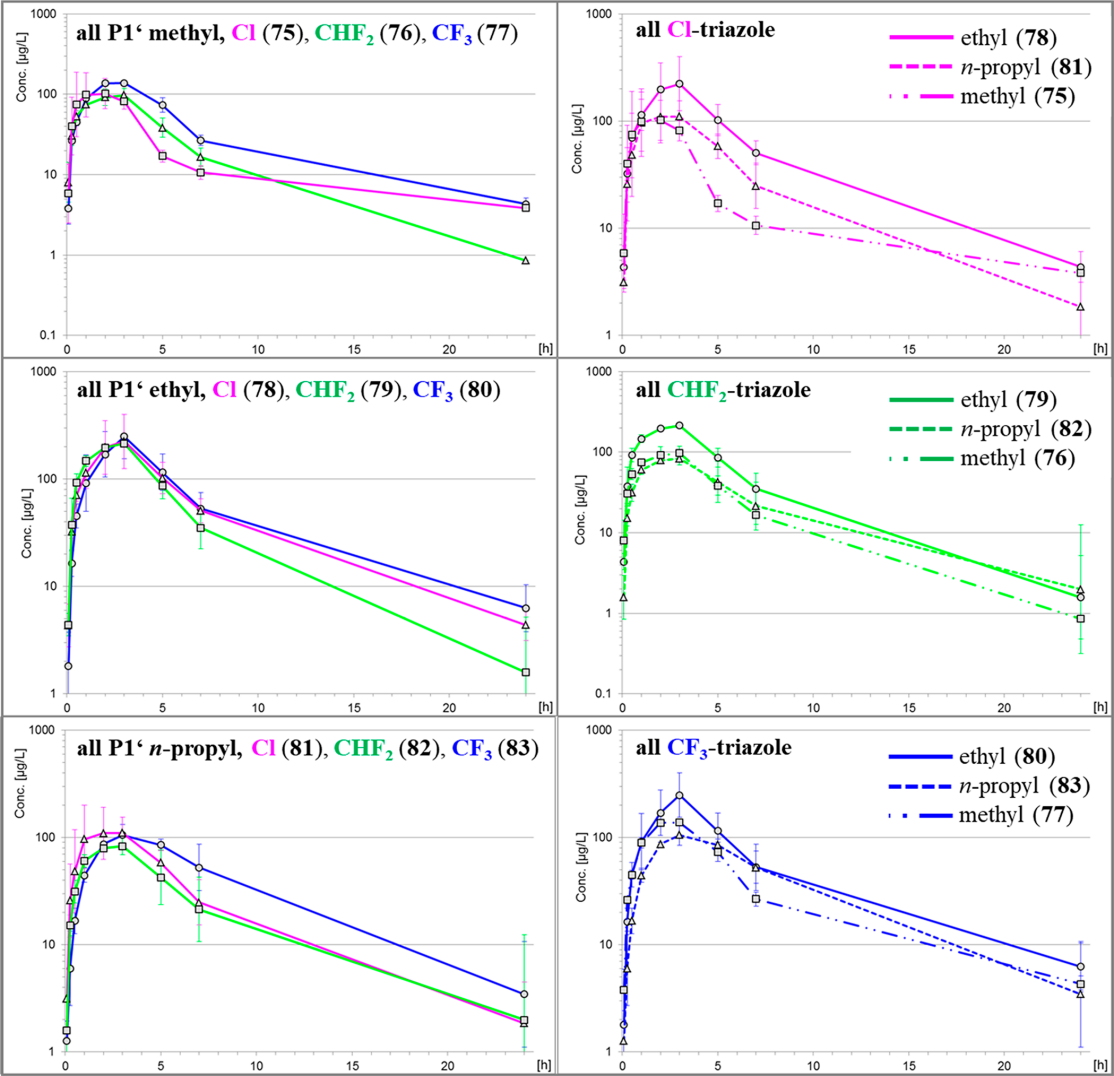
Concentration-to-time graphs for compounds **75**–**83** after oral administration in rats (see Table 7).

In the final modification cycle, we studied a 3 times 3 matrix of compounds as shortlist (single enantiomers **75**–**83**, Table 6), having chloro-, difluoromethyl-, or trifluoromethyl-substituted triazole as EBP group, methyl, ethyl, or *n*-propyl as P1′ group, and 2-fluorobenzamide as P2′ group. On the one hand, P1′ methyl derivatives were more potent FXIa inhibitors than their P1′ *n*-propyl counterparts. On the other hand, the P1′ *n*-propyl derivatives were favored due to their higher Caco-2 permeation and significantly lower efflux ratios compared to the P1′ methyl derivatives. Overall, the P1′ ethyl derivatives displayed properties somewhat in between. Furthermore, initial data on inhibition and induction of CYP isoforms for the P1′ methyl and ethyl derivatives did not reveal such a difference allowing for prioritization. As our main goal was to identify an FXIa inhibitor with high oral bioavailability and a human dose allowing for once-daily dosing, we focused on comparing all nine derivatives on the basis of their PK profiles in rats. Evaluation of the concentration-to-time graphs and the overall exposure (AUC_norm_) after oral administration (Figure 6) revealed that the P1′ ethyl substitution was superior to methyl and propyl for each triazole substitution. Based on the concentration-to-time graphs of each P1′ group, in trend, trifluoromethyl substitution at the triazole was more favorable than the other two substitutions. Focusing then on the P1′ ethyl set, difluoromethyl-substituted **79** was least potent and showed a significantly higher efflux ratio. Finally, it was decided to evaluate compound **80** (BAY 2433334, asundexian) in more depth and, after having gained favorable preclinical safety data, asundexian was moved into clinical Phase 1 studies.

At this point, a remark on the importance of the C5 methoxy group of our compound series is pertinent. Omitting the C5 methoxy group at the pyridinone core as for **84** and **85** led to a 20- to 23-fold drop in FXIa inhibitory potency compared to the corresponding **78** and **80** (Table 6), emphasizing the significant impact of this C5 methoxy group, a design element already introduced early on in the project during lead finding.

### Characterization of the Clinical Candidate Asundexian (**80**)

**Figure 7:**
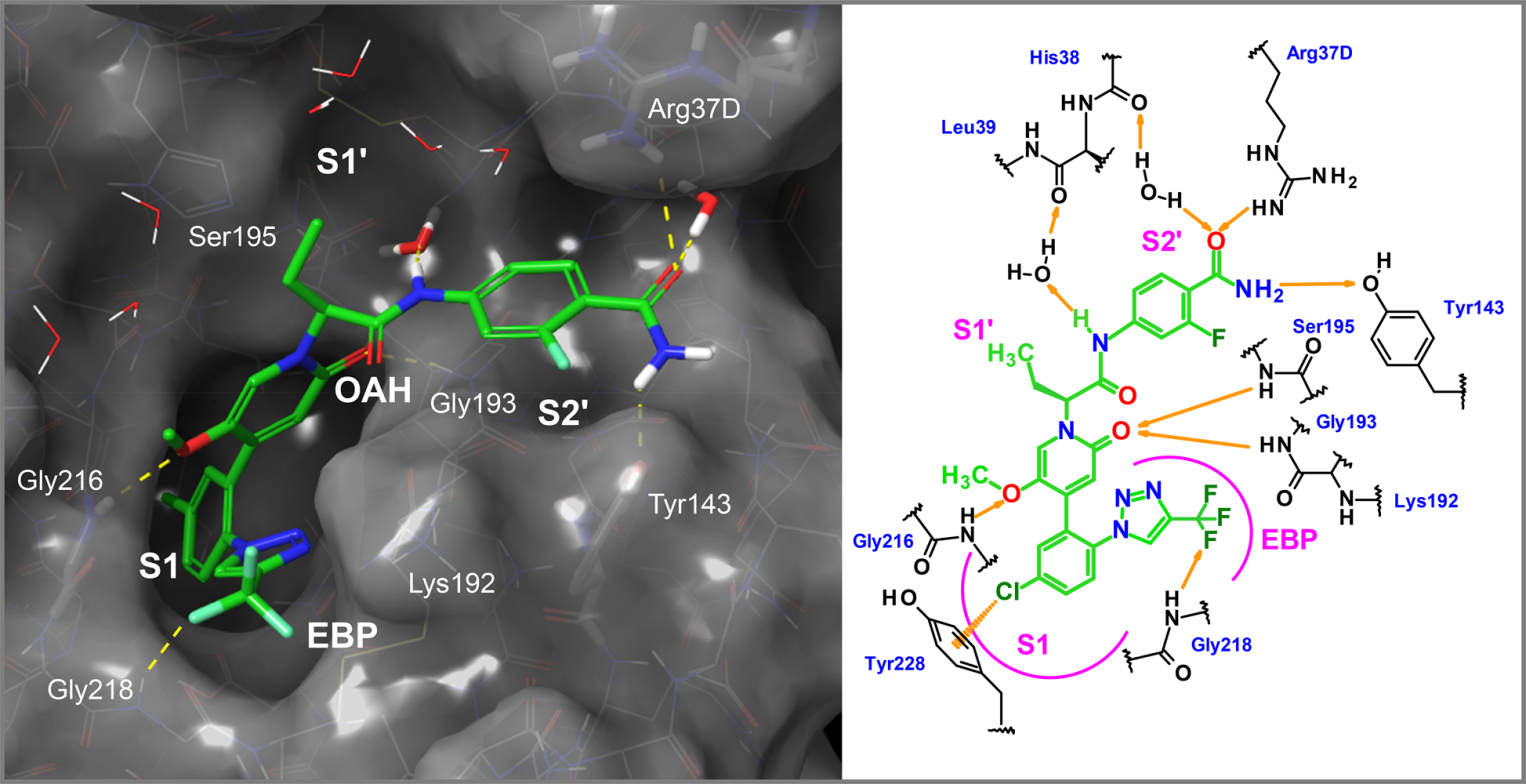
X-ray cocrystal structure of asundexian (**80**) in complex with human FXIa (PDB code 8BO3) and the corresponding 2D sketch.

Asundexian was cocrystallized with human FXIa (Figure 7). Special binding features include the formation of a *π*-cation interaction of the P1 chloroaryl with Tyr228 displacing an energetically unfavorable water molecule, a water-mediated hydrogen bond with the central amide NH, a hydrogen bond between one of the protons of the terminal P2′ amide function and the phenolic group of Tyr143, a hydrogen bond between the said amide carbonyl function and Arg37D and a water-mediated hydrogen bond to the backbone oxygen of His414, and a hydrogen bond between one of the fluorine residues of the substituted triazole and the Gly218 backbone NH. The central ethyl group points toward the S1′ pocket and undergoes lipophilic contacts. Strong hydrogen bonds are observed between the methoxy oxygen and Gly216 and for the pyridinone carbonyl group in the OAH (Gly193, Ser195).

The in-depth evaluation^8^ of asundexian revealed a potent and reversible inhibition of FXIa in buffer (IC_50_ = 1.0 nM) and after contact activation in human plasma (IC_50_ = 0.14 μM). Asundexian inhibited the closest homologue of FXIa, human plasma kallikrein, with IC_50_ values of 6.7 nM in buffer and 1.23 μM in human plasma. This potential reduction of both enzyme activities after contact activation may have intriguing features and is currently under further pharmacological evaluation. Asundexian showed favorable selectivity of greater than 1000-fold versus serine proteases associated with the hemostatic system, including FVIIa, FIXa, FXa, FXIIa, thrombin, urokinase, tissue plasminogen activator, activated protein C, or plasmin, and other proteases of potential importance related to the oral administration route, such as trypsin, chymotrypsin, and caldecrin (chymotrypsin c).

After asundexian was added to human plasma, the clotting time in the aPTT assay was prolonged with an EC_150_ of 0.20 μM (calculated in the final assay volume of 150 μL) and of 0.61 μM when calculated for the concentration in plasma (50 μL, see the Supporting Information for details). While the aPTT was prolonged in rabbit, dog, pig, and guinea pig plasma samples as well, with EC_150_ values of 4.5, 4.8, 1.5, and 6.4 μM, respectively, no prolongation of clotting time was observed in plasma samples from the mouse or rat (EC_150_ > 30 μM).

**Figure 8:**
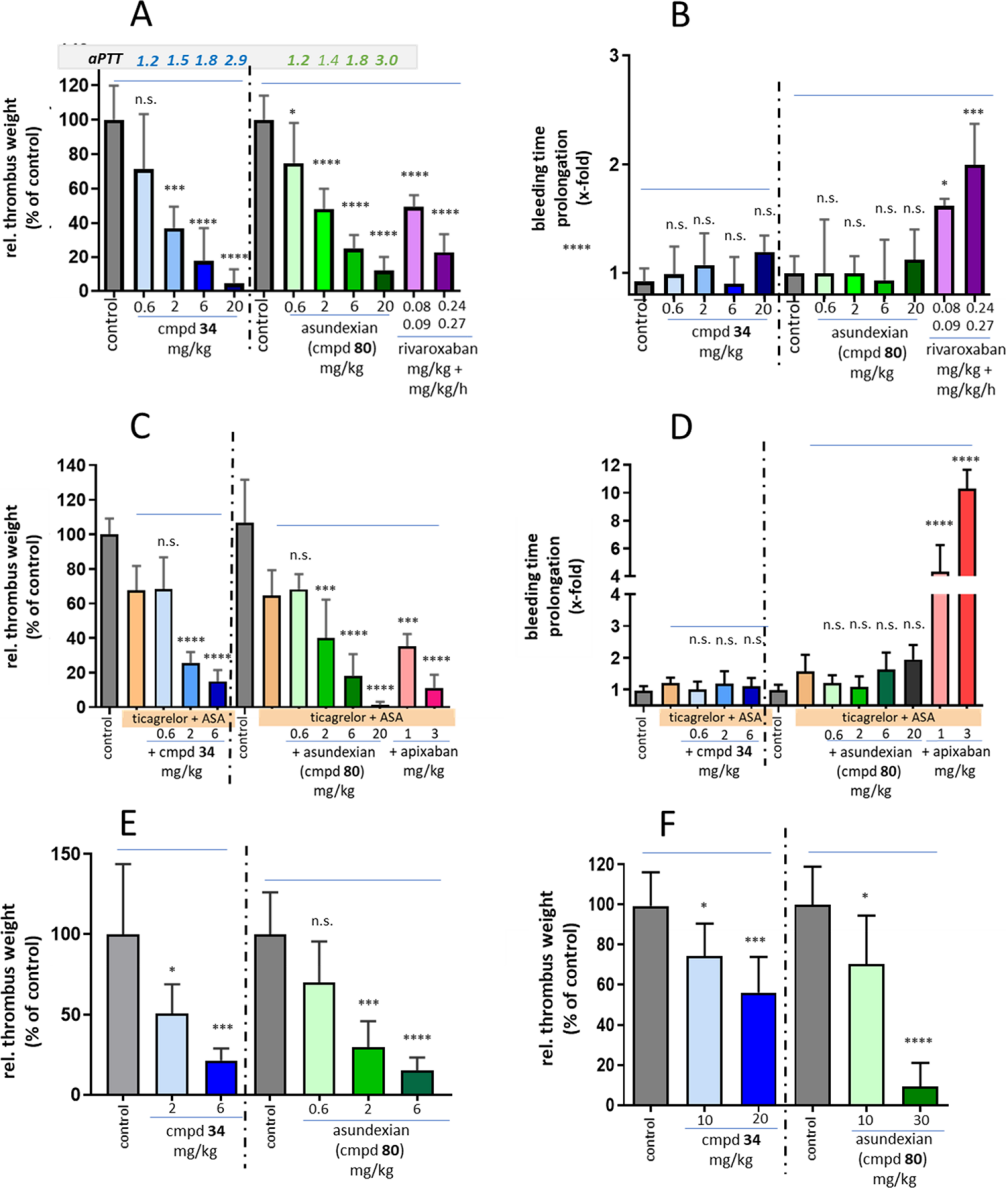
Antithrombotic effects and impact on hemostasis of compound **34** and asundexian (**80**) in vivo in rabbit models: (A) dose-dependent thrombus weight reduction after intravenously administered **34** (blue), asundexian (green), and rivaroxaban (magenta) vs control (gray) following FeCl_2_-induced damage to the carotid artery (arterial thrombosis model) with aPTT prolongation factors given for each dose group, and (B) simultaneously determined impact on bleeding time prolongation after a defined ear incision; (C and D) antithrombotic effects and impact on bleeding time prolongation compared to apixaban (red bars) in the models described in (A) and (B), with the additional presence of aspirin (ASA) and ticagrelor; (E) dose-dependent thrombus weight reduction following FeCl_2_-induced damage to the jugular vein (venous thrombosis model); (F) dose-dependent impact of orally administered compound **34** and asundexian on the thrombus weight compared to control in the model as described in (A); *n* = 4–8, mean + SD, *P < 0.05, **P < 0.01, ***P < 0.001, ****P < 0.0001.

In vivo, the antithrombotic effect of asundexian was determined in various thrombosis models. After FeCl_2_-induced injury of the rabbit carotid artery, the compound reduced the thrombus weight dose-dependently versus control animals when given intravenously in a prophylactic mode up to almost completeness at the highest doses, with an ED_50_ of 380 mg/L (Figure 8A). In the simultaneously conducted ear bleeding time measurements, no impact was observed in any of the asundexian groups (Figure 8B). This profile of strong antithrombotic efficacy without increasing the bleeding time was confirmed when asundexian was coadministered with antiplatelet drugs (aspirin and ticagrelor, Figure 8C,D) and are supported by studies in venous vessels (FeCl_2_-induced injury of the rabbit jugular vein, Figure 8E) and on artificial surfaces in arteriovenous shunt models (data not shown). When administered orally to rabbits at doses of 10 and 30 mg/kg in a PEG/ethanol/water solution, asundexian reduced the thrombus weight by 30 and 91%, respectively (Figure 8F).

One parameter which was critical for some earlier compounds was reversible and time-dependent CYP inhibition. Coincubation of asundexian did not affect CYP1A2, CYP2A6, CYP2B6, CYP2C19, CYP2E1, CYP2J2, and CYP3A4 activities up to the highest test concentration (IC_50_ > 41 μM). Weak inhibitory potential on CYP2C8 (IC_50_ = 3.6 μM), CYP2C9 (IC_50_ = 17 μM), CYP1A1 (IC_50_ = 13 μM), and CYP2D6 (IC_50_ = 19 μM) was observed. Furthermore, after preincubation (30 min) of asundexian with NADPH-supplemented human liver microsomes, a slight increase in the inhibitory potency of asundexian was only shown toward CYP3A4 (IC_50_ = 17 μM). This was further investigated in human hepatocytes, and relevant TDI of CYP3A4 activity was not observed.

In Caco-2 cells, asundexian showed a high permeability [*P*_app_(A–B) = 143 nm/s] and a moderate efflux ratio of 7. The fraction unbound of asundexian in plasma was determined using the equilibrium dialysis method with ^3^H-labeled drug substance and is moderate in rat (2.4%), monkey (6.2%), and human (6.4%) plasma and is high in dog (10%) and rabbit (14%) plasma.

After intravenous (bolus, 0.3 mg/kg) and oral (1.0 mg/kg) administration to male Wistar rats and female beagle dogs, asundexian showed low CL (0.46 and 0.19 L/h/kg), high volume of distribution (0.76 and 1.80 L/kg), and moderate to high bioavailability (60 and 97%) (Tables 7 and 8).

**Table 8:** In Vivo Pharmacokinetic Properties of Selected Compounds in Dogs (0.3 mg/kg iv infusion and 1.0 mg/kg po)

compd	AUC_norm_^iv^ [kg·h/L]	CL [L/h/kg]	*V*_ss_ [L/kg]	MRT_iv_ [h]	AUC_norm_^po^ [kg·h/L]	*F^a^* [%]	*f*_u_ [%]
							dog
**25**	7.7	0.13	0.55	4.2	3.9	51	8.5
**34**	2.1	0.48	1.40	2.9	1.9	55	13
**80**	5.3	0.19	1.80	9.4	5.1	97	10

### First Results on Clinical Studies with Asundexian (**80**)

In the Phase 1 program, no clinically relevant bleeding events or impact on bleeding times was observed. Asundexian showed a consistent PK/PD relationship, with dose-dependent changes in pharmacodynamic parameters such as anticoagulant activity (aPTT) and FXIa inhibitory activity, while bleeding times were consistent across all dose cohorts and similar to those of the placebo. In single dose and multi-dose studies on human pharmacokinetics, asundexian showed a dose-proportional increase in exposure with high oral bioavailability, and after administration of immediate release tablets, a geometric mean elimination half-life of about 14–17 h.^44−46^ Therefore, asundexian is a promising clinical candidate as a once-daily oral anticoagulant and has been investigated for its efficacy and safety in three Phase 2 dose finding studies, namely, PACIFIC-AF^18^ (NCT04218266) in patients with atrial fibrillation, PACIFIC-STROKE^19^ (NCT04304508) in patients with a noncardioembolic stroke, and PACIFIC-AMI^20^ (NCT04304534) in patients with acute myocardial infarction. Currently, the OCEANIC program is ongoing with two Phase 3 studies investigating the efficacy and safety of asundexian in the prevention of stroke events in patients with atrial fibrillation as well as in patients with a noncardioembolic ischemic stroke or high-risk transient ischemic attack, involving up to 30,000 patients (OCEANIC-AF and OCEANIC-STROKE). The U.S. Food and Drug Administration (FDA) has granted Fast Track designation for asundexian as a potential treatment for secondary prevention in patients with a noncardioembolic ischemic stroke.

### Chemistry

**Scheme 1:**
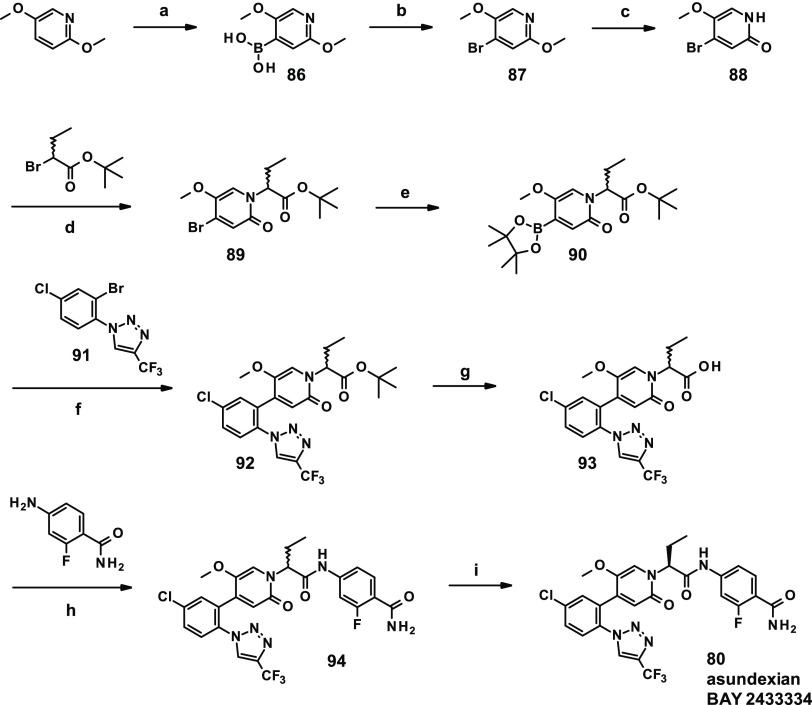
Synthesis of Asundexian (BAY 2433334, **80**) Reagents and conditions: (a) (i) LDA, THF, (ii) B(O*i*-Pr)_3_, −78 °C → RT, (iii) aq. HCl, 72%; (b) CuBr_2_, MeOH/H_2_O, 100 °C/microwave, 65%; (c) pyridine hydrobromide, DMF, 100 °C, 69%; (d) *tert*-butyl 2-bromobutanoate, K_2_CO_3_, DMF, 50 °C, 53%; (e) bis(pinacolato)diboron, Pd(dppf)Cl_2_-DCM complex, KOAc, 1,4-dioxane, 80 °C; (f) Pd(dppf)Cl_2_-DCM complex, K_2_CO_3_, 1,4-dioxane, 80 °C, 59% for two steps; (g) 4 M HCl/1,4-dioxane, RT, 99%; (h) T3P (50% in ethyl acetate), pyridine, 40 °C, 84%; (i) enantiomer separation.

The synthesis route of asundexian (**80**) used during the research phase comprises eight linear steps and a chiral separation as the last step (Scheme 1). Starting from commercially available 2,5-dimethoxypyridine, deprotonation with LDA, addition of triisopropyl borate, and subsequent ester hydrolysis resulted in boronic acid **86** in 72% yield. Boronic acid **86** was converted into bromide **87** in the presence of copper(II) bromide in 65% yield. As direct bromination of 2,5-dimethoxypyridine resulted in undesired regioisomeric mixtures, this two-step approach was used to obtain the desired bromide regioisomer. The methoxy group adjacent to the pyridyl nitrogen was selectively deprotected with pyridine hydrobromide to give **88** in 69% yield. Alkylation of **88** with *tert*-butyl 2-bromobutanoate afforded racemate **89** in 53% yield. Best to use in this step is the *tert*-butyl ester, as the methyl or ethyl esters are too prone to saponification due to activation by the pyridinone substitution. The benzyl ester also works, and both the *tert*-butyl and benzyl ester can be already deprotected under basic conditions (e.g., with lithium hydroxide). Pinacol boronic ester formation (→ **90**) with subsequent Suzuki coupling of 1-(2-bromo-4-chlorophenyl)-4-(trifluoromethyl)-1*H*-1,2,3-triazole (**91**)^47^ resulted in compound **92** in 59% yield for the two steps. After ester deprotection, acid **93** was coupled with 4-amino-2-fluorobenzamide in the presence of propylphosphonic anhydride (T3P) to yield compound **94** in 84% yield. A last step, chiral separation of racemate **94** resulted in eutomer **80** (asundexian).

## Conclusions

Using protein structure-based *de novo* design, we identified a novel micromolar hit as FXIa inhibitor with attractive physicochemical properties. The main parameters we attempted to improve upon were potency, permeability, metabolic stability, and cytochrome P450 interaction profile. A careful design approach focused on understanding the compound’s conformational behavior and introducing functional groups that (1) undergo strong interactions with the target protein, (2) rigidify the structure to offer the FXIa preformed conformations, exactly fitting in the active site without losing entropy upon binding, and (3) keep the polar surface low. Only a focus on the most important hydrogen bond donor and acceptor interactions enabled a balanced combination of potency and absorption.

The first compound that reached clinical trials was a highly potent inhibitor with a carboxylic acid moiety. The inclusion of that moiety solved many problems with cytochrome P450 interaction but the compound ultimately suffered from low oral exposure and a short half-life in humans. Active transport issues were suspected to be related to the acidic moiety.

In a second attempt, the carboxylic acid moiety was replaced by carboxamides capable of forming an intramolecular hydrogen bond. The challenge was to regain potency and to simultaneously attempt to improve the metabolic stability and cytochrome P450 interaction profile on top of high potency and permeability. Several learning cycles were needed to identify the most promising substituents for addressing each of the protease pockets, S1, EBP, S1′, and S2′, within one compound. The resulting compound from our extensive research program, asundexian (BAY 2433334, **80**), combines high potency and selectivity with excellent oral bioavailability, a long half-life, and a favorable CYP interaction profile. Asundexian shows antithrombotic efficacy in arterial and venous thrombosis models in prevention and intervention settings, without increasing bleeding. Completed clinical Phase 1 trials^45,46^ and the PACIFIC Phase 2 program (PACIFIC-AF,^18^ PACIFIC-STROKE,^19^ PACIFIC-AMI^20^) on asundexian have confirmed the desired DMPK properties and the initial pharmacological hypothesis. The OCEANIC Phase 3 studies to investigate asundexian in patients with atrial fibrillation (OCEANIC-AF) or after stroke (OCEANIC-STROKE) have been initiated. The U.S. FDA has granted Fast Track designation for asundexian as a potential treatment for secondary prevention in patients with a noncardioembolic ischemic stroke.

## Experimental Section

We describe the synthesis of compound **80** (asundexian, BAY 2433334) here in detail, referring to a route used during the research phase. Meanwhile, a process synthesis has also been disclosed.^48^ The synthesis of all other derivatives is described in the Supporting Information.

### General Procedures

All commercial reagents and catalysts were used as provided by the commercial supplier without purification. Solvents for synthesis, extraction, and chromatography were of reagent grade and used as received. Moisture-sensitive reactions were carried out under an atmosphere of argon, and anhydrous solvents were used as provided by the commercial supplier. Preparative normal-phase flash chromatography was performed using Biotage Isolera chromatography systems with Biotage silica cartridges or silica gel 60 (230–400 mesh) in combination with glass columns/frits. Preparative reversed-phase (RP) chromatography was performed on 125/250 mm × 20/30/40 mm HPLC columns packed with YMC gel ODS-AQ S-5/15 μm and UV detection. Gradients or isocratic mixtures used as eluents are indicated. All compounds tested in biological assays were of ≥95% purity, as determined by HPLC, LC/MS, or NMR data.

^1^H NMR and ^13^C NMR spectra were recorded at RT with Bruker Avance spectrometers. Chemical shifts (δ) are reported in ppm relative to TMS as an internal standard. The descriptions of the coupling patterns of ^1^H NMR signals are based on the optical appearance of the signals and do not necessarily reflect the physically correct interpretation. In general, the chemical shift information refers to the center of the signal. In the case of multiplets, intervals are given.

Analytical mass spectrometry was performed on HPLC/MS (Waters, Agilent, Thermo Fisher) or GC/MS (Waters, Agilent, Thermo Fisher) systems using Waters Time-of-Flight, Waters/Micromass Single Quadrupole, or Thermo Fisher Scientific Orbitrap mass spectrometers. Ionization methods were electrospray ionization (ESI) positive/negative or electron ionization (EI). LC/MS and GC/MS analyses were performed using the respective method 1–3 or 21, as noted.

Method 1. Instrument: Waters Acquity SQD UPLC system; column: Waters Acquity UPLC HSS T3 C18 1.8 μm, 50 mm × 1.0 mm; eluent A: water + 0.025% formic acid, eluent B: acetonitrile + 0.025% formic acid; gradient: 0.0 min 10% B → 1.2 min 95% B → 2.0 min 95% B; temperature: 50 °C; flow rate: 0.40 mL/min; UV detection: 210–400 nm.

Method 2. Instrument: Micromass Quattro Premier MS with Waters Acquity UPLC; column: Thermo Hypersil GOLD 1.9 μm, 50 mm × 1 mm; eluent A: 1 L water + 0.5 mL 50% formic acid, eluent B: 1 L acetonitrile + 0.5 mL 50% formic acid; gradient: 0.0 min 97% A → 0.5 min 97% A → 3.2 min 5% A → 4.0 min 5% A; temperature: 50 °C; flow rate: 0.3 mL/min; UV detection: 210 nm.

Method 3. Instrument: Thermo Scientific FT-MS with Thermo Scientific UltiMate 3000 UHPLC; column: Waters HSS T3 C18 1.8 μm, 75 mm × 2.1 mm; eluent A: water + 0.01% formic acid; eluent B: acetonitrile + 0.01% formic acid; gradient: 0.0 min 10% B → 2.5 min 95% B → 3.5 min 95% B; temperature: 50 °C; flow rate: 0.90 mL/min; UV detection: 210–400 nm.

Method 21. Instrument: Waters TOF MS with Waters Acquity I-Class UPLC; column: Waters Acquity UPLC HSS T3, 1.8 μm, 150 mm × 2.1 mm; eluent A: 1 L water + 0.100 mL 99% trifluoroacetic acid, eluent B: 1 L acetonitrile + 0.100 mL 99% trifluoroacetic acid; gradient: 0.0 min 5% B → 1 min 5% B → 13 min 95% B → 15 min 95% B; temperature: 50 °C; flow rate: 0.60 mL/min; UV detection: 210 nm.

Assessment of optical rotation [α] was performed using an Anton Paar polarimeter MCP2000 with parameters (wavelength, temperature, optical pathway, solvent, and concentration) as indicated.

### 2,5-Dimethoxypyridin-4-ylboronic Acid (**86**)

Lithium diisopropylamide (2 M in tetrahydrofuran, 49.71 mL, 99.43 mmol, 1.2 equiv) was added under an argon atmosphere at −78 °C to a solution of 2,5-dimethoxypyridine (11.53 g, 82.86 mmol) in tetrahydrofuran (260 mL). The mixture was stirred at −78 °C for 2 h, followed by the quick addition of triisopropyl borate (38.82 mL, 168.20 mmol, 2.03 equiv). The reaction mixture was maintained at −78 °C for a further 2 h and then slowly (!) thawed to RT overnight. After addition of water, tetrahydrofuran was removed under reduced pressure, and the aqueous phase was extracted with ethyl acetate. The aqueous phase was acidified with aqueous hydrochloric acid solution (2 N), resulting in the formation of a precipitate which was collected by filtration, washed with water, and dried in vacuo to give a first batch of **86**. Yield: 9.53 g (61% of theory). The filtrate was extracted with ethyl acetate. The combined organic phases were dried over sodium sulfate, filtered, concentrated under reduced pressure, and dried in vacuo to give a second batch of **86**. Yield: 2.01 g (85% purity, 11% of theory). LC/MS (method 1): *t*_R_ = 0.47 min, MS (ESIpos): *m*/*z* = 184 [M + H]^+^; ^1^H NMR (400 MHz, DMSO-*d*_6_): δ [ppm] = 8.15 (s, 2H), 7.79 (s, 1H), 6.76 (s, 1H), 3.78 (s, 3H), 3.77 (s, 3H).

### 4-Bromo-2,5-dimethoxypyridine (**87**)

A mixture of 2,5-dimethoxypyridin-4-ylboronic acid (**86**) (2.25 g, 12.05 mmol) and copper(II) bromide (4.04 g, 18.08 mmol, 1.5 equiv) in methanol (24 mL) and water (24 mL) was irradiated in a microwave oven at 100 °C for 60 min. After reaching RT, the precipitate was collected by filtration, washed with water, mixed with methanol (600 mL), and stirred at 65 °C for 1 h, then the mixture was filtered. After the residue was dissolved in dichloromethane, the solution was washed with diluted ammonia solution, dried over sodium sulfate, filtered, concentrated under reduced pressure, and dried in vacuo to give **87**. Yield: 1.71 g (65% of theory). LC/MS (method 2): *t*_R_ = 2.12 min, MS (ESIpos): *m*/*z* = 218 [M + H]^+^; ^1^H NMR (400 MHz, DMSO-*d*_6_): δ [ppm] = 7.93 (s, 1H), 7.16 (s, 1H), 3.87 (s, 3H), 3.80 (s, 3H).

### 4-Bromo-5-methoxypyridin-2(1*H*)-one (**88**)

Pyridine hydrobromide (6.59 g, 411.78 mmol, 20 equiv) was added to a solution of 4-bromo-2,5-dimethoxypyridine (**87**) (4.88 g, 20.59 mmol) in *N*,*N*-dimethylformamide (180 mL). The mixture was stirred at 100 °C for 3 h and concentrated under reduced pressure. The residue was mixed with water (50 mL) and the solution cooled in an ice bath for 30 min. The formed precipitate was collected by filtration, washed with water, and dried in vacuo to give **88**. Yield: 2.94 g (69% of theory). LC/MS (method 2): *t*_R_ = 1.25 min, MS (ESIpos): *m*/*z* = 204 [M + H]^+^; ^1^H NMR (400 MHz, DMSO-*d*_6_): δ [ppm] = 11.43 (br s, 1H), 7.19 (s, 1H), 6.77 (s, 1H), 3.71 (s, 3H).

### *tert*-Butyl 2-(4-Bromo-5-methoxy-2-oxopyridin-1(2*H*)-yl)butanoate (Racemate **89**)

Racemic *tert*-butyl 2-bromobutanoate (66.27 g, 297.12 mmol, 1.2 equiv) was added under an argon atmosphere at RT to a mixture of 4-bromo-5-methoxypyridin-2(1*H*)-one (**88**) (50.50 g, 247.51 mmol) and potassium carbonate (85.52 g, 618.78 mmol) in *N*,*N*-dimethylformamide (884 mL). The reaction mixture was stirred at 50 °C for 2 h, mixed with brine and ethyl acetate, and extracted with ethyl acetate. The combined organic phases were washed with brine, dried, and concentrated under reduced pressure. The residue was purified by flash silica gel chromatography (petroleum ether/ethyl acetate 8:2) to give **89**. Yield: 45.70 g (53% of theory). LC/MS (method 3): *t*_R_ = 1.80 min, MS (ESIpos): *m*/*z* = 346 [M + H]^+^; ^1^H NMR (400 MHz, DMSO-*d*_6_): δ [ppm] = 7.36 (s, 1H), 6.86 (s, 1H), 4.93 (dd, *J* = 9.3 Hz, 6.2 Hz, 1H), 3.73 (s, 3H), 2.13–2.01 (m, 2H), 1.38 (s, 9H), 0.80 (t, *J* = 7.4 Hz, 3H).

### *tert-*Butyl 2-[5-Methoxy-2-oxo-4-(4,4,5,5-tetramethyl-1,3,2-dioxaborolan-2-yl)pyridin-1(2*H*)-yl]butanoate (Racemate **90**)

A suspension of *tert*-butyl 2-(4-bromo-5-methoxy-2-oxopyridin-1(2*H*)-yl)butanoate (racemate **89**) (5.00 g, 14.44 mmol), bis(pinacolato)diboron (4.03 g, 15.89 mmol, 1.1 equiv), and potassium acetate (4.25 g, 43.32 mmol, 3.0 equiv) in 1,4-dioxane (105 mL) was flushed with argon for 5 min, followed by addition of [1,1′-bis(diphenylphosphino)ferrocene]dichloropalladium-dichloromethane complex (354 mg, 0.43 mmol). The reaction mixture was stirred at 80 °C for 1.5 h, cooled to RT, and filtered through Celite, and the filter residue was washed with ethyl acetate. The combined filtrates were concentrated under reduced pressure and dried in vacuo to give **90** which was used without further purification. Yield: 10.36 g. LC/MS (method 3): *t*_R_ = 1.22 min, MS (ESIpos): *m*/*z* = 312 [M + H]^+^ [boronic acid fragment].

### 1-(2-Bromo-4-chlorophenyl)-4-(trifluoromethyl)-1*H*-1,2,3-triazole (**91**)

In a three-neck flask (equipped with an empty balloon to trap excess gas and avoid pressure build up; however, it remained empty during the reaction), copper(I) oxide (690 mg, 4.8 mmol) was added at RT to a solution of 1-azido-2-bromo-4-chlorobenzene (10.4 g, 44.7 mmol) in acetonitrile (600 mL). Trifluoropropyne (5 g cylinder) was bubbled gently through the solution at RT for 10–15 min until the cylinder was empty. After capping the flask and stirring for 3 d, approximately 80% conversion into the desired product was observed. Further trifluoropropyne (1 g from a second 5 g cylinder) was bubbled gently through the solution. The solution was stirred overnight and then concentrated under reduced pressure. The residue was taken up in a mixture of *n*-heptane/dichloromethane (1:1) and filtered through a plug of silica gel. The residue was crystallized from *n*-heptane to give a first batch of **91** (9.5 g). Precipitation from the mother liquor gave a second batch of **91** (0.9 g). The batches were combined. Yield: 10.4 g (71% of theory). LC/MS (method 3): *t*_R_ = 2.04 min, MS (ESIpos): *m*/*z* = 326 [M + H]^+^; ^1^H NMR (400 MHz, DMSO-*d*_6_): δ [ppm] = 9.42 (s, 1H), 8.17 (d, *J* = 2.2 Hz, 1H), 7.85 (d, *J* = 8.6 Hz, 1H), 7.78 (dd, *J* = 8.6 Hz, 2.2 Hz, 1H).

### *tert*-Butyl 2-[4-{5-Chloro-2-[4-(trifluoromethyl)-1*H*-1,2,3-triazol-1-yl]phenyl}-5-methoxy-2-oxopyridin-1(2*H*)-yl]butanoate (Racemate **92**)

A mixture of *tert*-butyl 2-[5-methoxy-2-oxo-4-(4,4,5,5-tetramethyl-1,3,2-dioxaborolan-2-yl)pyridin-1(2*H*)-yl]butanoate (racemate **90**) (5.67 g, 53% purity, 7.66 mmol), 1-(2-bromo-4-chlorophenyl)-4-(trifluoromethyl)-1*H*-1,2,3-triazole (**91**) (2.50 g, 7.66 mmol, 1.0 equiv), and potassium carbonate (3.17 g, 22.97 mmol, 3.0 equiv) in 1,4-dioxane (78 mL) was flushed with argon for 5 min, followed by addition of [1,1′-bis(diphenylphosphino)ferrocene]dichloropalladium-dichloromethane complex (375 mg, 0.46 mmol, 0.06 equiv). The reaction mixture was stirred at 80 °C overnight, cooled to RT, and filtered through Celite, and the filter residue was washed with dichloromethane and acetonitrile. The combined filtrates were concentrated, and the residue was purified by flash silica gel chromatography (cyclohexane/ethyl acetate gradient) to give **92**. Yield: 2.32 g (59% of theory). LC/MS (method 3): *t*_R_ = 2.14 min, MS (ESIpos): *m*/*z* = 513 [M + H]^+^; ^1^H NMR (400 MHz, DMSO-*d*_6_): δ [ppm] = 9.13 (s, 1H), 7.82 (s, 2H), 7.78 (s, 1H), 7.03 (s, 1H), 6.48 (s, 1H), 4.94–4.86 (m, 1H), 3.22 (s, 3H), 2.09–1.97 (m, 2H), 1.38 (s, 9H), 0.74 (t, *J* = 7.4 Hz, 3H).

### 2-[4-{5-Chloro-2-[4-(trifluoromethyl)-1*H*-1,2,3-triazol-1-yl]phenyl}-5-methoxy-2-oxopyridin-1(2*H*)-yl]butanoic Acid (Racemate **93**)

A solution of *tert*-butyl 2-[4-{5-chloro-2-[4-(trifluoromethyl)-1*H*-1,2,3-triazol-1-yl]phenyl}-5-methoxy-2-oxopyridin-1(2*H*)-yl]butanoate (racemate **92**) (355 mg, 83% purity, 0.57 mmol) in hydrogen chloride solution (4 M in 1,4-dioxane, 8.3 mL) was stirred at RT overnight and then concentrated. The residue was purified by preparative RP-HPLC (acetonitrile/water with 0.1% formic acid gradient) to give **93**. Yield: 260 mg (99% of theory). LC/MS (method 1): *t*_R_ = 0.90 min, MS (ESIpos): *m*/*z* = 457 [M + H]^+^; ^1^H NMR (400 MHz, DMSO-*d*_6_): δ [ppm] = 12.90 (br s, 1H), 9.10 (s, 1H), 7.86–7.76 (m, 3H), 7.07 (s, 1H), 6.48 (s, 1H), 5.00 (br s, 1H), 3.20 (s, 3H), 2.14–2.00 (m, 2H), 0.71 (t, *J* = 7.4 Hz, 3H).

### 4-{2-[4-{5-Chloro-2-[4-(trifluoromethyl)-1*H*-1,2,3-triazol-1-yl]phenyl}-5-methoxy-2-oxopyridin-1(2*H*)-yl]butanamido}-2-fluorobenzamide (Racemate **94**)

Propylphosphonic anhydride (T3P, 50% solution in ethyl acetate, 1.52 mL, 2.56 mmol, 1.6 equiv) was added under an argon atmosphere at RT to a solution of 2-[4-{5-chloro-2-[4-(trifluoromethyl)-1*H*-1,2,3-triazol-1-yl]phenyl}-5-methoxy-2-oxopyridin-1(2*H*)-yl]butanoic acid (racemate **93**) (1.00 g, 73% purity, 1.60 mmol) in pyridine (13.1 mL). The reaction mixture was heated to 40 °C, mixed with 4-amino-2-fluorobenzamide (0.32 g, 2.08 mmol, 1.3 equiv), stirred for an additional 15 min at 40 °C, and then immediately concentrated under reduced pressure. The residue was taken up in acetonitrile (10 mL), acidified with aqueous hydrochloric acid solution (1 M, 3 mL), and purified by preparative RP-HPLC chromatography (acetonitrile/water with 0.1% formic acid gradient) to give **94**. Yield: 794 mg (84% of theory). LC/MS (method 1): *t*_R_ = 0.92 min, MS (ESIpos): *m*/*z* = 593 [M + H]^+^; ^1^H NMR (400 MHz, DMSO-*d*_6_): δ [ppm] = 10.76 (br s, 1H), 9.13 (s, 1H), 7.86–7.80 (m, 2H), 7.79–7.77 (m, 1H), 7.69 (t, *J* = 8.6 Hz, 1H), 7.66–7.61 (m, 1H), 7.56–7.49 (m, 2H), 7.37 (dd, *J* = 8.6 Hz, 1.8 Hz, 1H), 7.13 (s, 1H), 6.53 (s, 1H), 5.55–5.49 (m, 1H), 3.26 (s, 3H), 2.14–2.02 (m, 2H), 0.79 (t, *J* = 7.2 Hz, 3H).

### 4-{(2*S*)-2-[4-{5-Chloro-2-[4-(trifluoromethyl)-1*H*-1,2,3-triazol-1-yl]phenyl}-5-methoxy-2-oxopyridin-1(2*H*)-yl]butanamido}-2-fluorobenzamide, Alternatively: (4*S*)-2^4^-Chloro-4-ethyl-7^3^-fluoro-3^5^-methoxy-3^2^,5-dioxo-1^4^-(trifluoromethyl)-3^2^*H*-6-aza-3(4,1)-pyridina-1(1)-[1,2,3]triazola-2(1,2),7(1)-dibenzenaheptaphane-7^4^-carboxamide (Eutomer 80, Asundexian, BAY 2433334)

Enantiomer separation of 793 mg of 4-{2-[4-{5-chloro-2-[4-(trifluoromethyl)-1*H*-1,2,3-triazol-1-yl]phenyl}-5-methoxy-2-oxopyridin-1(2*H*)-yl]butanamido}-2-fluorobenzamide (racemate **94**) resulted in 328 mg of distomer (chiral HPLC: *t*_R_ = 1.26 min, >99% ee) and 295 mg of eutomer **80** (chiral HPLC: *t*_R_ = 1.97 min, >99% ee). Separation method: SFC: column: Chiralpak AD-H 5 μm, 250 mm × 20 mm; mobile phase: 80% carbon dioxide/20% ethanol; temperature: 40 °C; flow rate: 80 mL/min; UV detection: 210 nm. Analytical method: SFC: column: Daicel AD-H 3 μm, 100 mm × 4.6 mm; mobile phase: 75% carbon dioxide/25% ethanol; flow rate: 3 mL/min; UV detection: 210 nm. [α]_D_^20^ = −61.6 (*c* 0.36, methanol). LC/MS (method 1): *t*_R_ = 0.92 min, MS (ESIpos): *m*/*z* = 593 [M + H]^+^; LC/MS (method 21): *t*_R_ = 7.95 min, HRMS (ESIpos): *m*/*z* = 593.1249 [M + H]^+^; ^1^H NMR (500 MHz, DMSO-*d*_6_): δ [ppm] = 10.79 (br s, 1H), 9.14 (s, 1H), 7.88–7.81 (m, 2H), 7.79 (d, *J* = 2.0 Hz, 1H), 7.70 (t, *J* = 8.5 Hz, 1H), 7.67–7.63 (m, 1H), 7.56 (s, 1H), 7.54 (br s, 1H), 7.38 (dd, *J* = 8.5 Hz, 1.5 Hz, 1H), 7.15 (s, 1H), 6.55 (s, 1H), 5.60–5.48 (m, 1H), 3.27 (s, 3H), 2.17–2.03 (m, 2H), 0.80 (t, *J* = 7.1 Hz, 3H); ^13^C NMR (126 MHz, DMSO-*d*_6_): δ [ppm] = 168.72 (s), 164.39 (s), 159.57 (d, *J* = 248.2 Hz), 159.07 (s), 142.08 (d, *J* = 11.9 Hz), 141.24 (s), 138.99 (s), 136.50 (q, *J* = 38.6 Hz), 135.01 (s), 133.17 (s), 132.53 (s), 131.06 (d, *J* = 3.7 Hz), 130.52 (s), 130.01 (s), 127.40 (s), 126.98 (d, *J* = 2.8 Hz), 120.67 (s), 120.52 (q, *J* = 267.5 Hz), 117.88 (d, *J* = 13.8 Hz), 116.58 (br d, *J* = 5.5 Hz), 114.68 (d, *J* = 1.8 Hz), 106.13 (d, *J* = 28.5 Hz), 58.97 (br d, *J* = 7.8 Hz), 56.05 (s), 23.67 (s), 9.92 (s).

## Supporting Information Available

General methods; crystallographic data for compounds **1**–**3**, **34**, and asundexian (**80)**; in vitro, ex vivo, and in vivo pharmacology assays, and DMPK assays; ^1^H NMR and LC/MS data of intermediates **86**–**94**; ^1^H NMR, ^13^C NMR, LC/MS, and chiral HPLC data of asundexian (**80**); synthesis of compounds **3**–**79**, **81**–**85**, **355**, and **362**; LC/MS data of compounds **1**–**85**, **355**, and **362** (PDF)
Molecular formula string spreadsheet of compounds **1**–**85**, **355**, and **362** with FXIa IC_50_ values (CSV)

## Accession Codes

The coordinates and structure factors have been deposited to the Protein Data Bank (PDB) with accession codes, 8BO4 (**1**), 8BO6 (**2**), 8BO5 (**3**), 8BO7 (**34**), and 8BO3 (**80**, asundexian). Authors will release the atomic coordinates upon article publication.

## Abbreviations Used

aPTT: activated partial thromboplastin time
AUC_norm_: area under the curve normalized
Caco-2: colon carcinoma cell line
CL: clearance
clogD^7.5^: calculated logarithm of distribution coefficient at pH 7.5
CYP3A4: cytochrome P450 3A4
DOACs: direct oral anticoagulants
dppf: 1,1′-bis(diphenylphosphino)ferrocene
EBP: ester binding pocket
FEP: free-energy perturbation
*f*_u_: fraction unbound
FVIIa: factor VIIa
FIXa: factor IXa
FXa: factor Xa
FXIa: factor XIa
LE: ligand efficiency
LLE: ligand lipophilicity efficiency
MRT: mean residence time
MW_corr_: corrected molecular weight
OAH: oxyanion hole
OATP: organic anion-transporting polypeptides
P1, P2, P1′, P2′: substrate residues of proteinases
*P*_app_: apparent permeability coefficient
P-gp: permeability glycoprotein
S1, S2, S4, S1′, S2′ pockets: substrate binding pockets of proteinases
SFC: supercritical fluid chromatography
SD: standard deviation
T3P: propylphosphonic anhydride
TDI: time-dependent inhibition
tPSA: topological polar surface area
*V*_ss_: steady-state volume of distribution.
