# Supplementary material for: Design and Preclinical Characterization Program toward Asundexian (BAY 2433334), an Oral Factor XIa Inhibitor for the Prevention and Treatment of Thromboembolic Disorders
Source: J Med Chem. 2023 Sep 5;66(17):12203–24. doi: 10.1021/acs.jmedchem.3c00795 (PMC10510402; doi:10.1021/acs.jmedchem.3c00795)
Supplement: Supplementary file 1 — jm3c00795_si_001.pdf [file jm3c00795_si_001.pdf]

# Supporting Information

# Design and Preclinical Characterization Program Towards Asundexian (BAY 2433334), an Oral Factor XIa Inhibitor for the Prevention and Treatment of Thromboembolic Disorders

Susanne Roehrig,* Jens Ackerstaff, Eloísa Jiménez Núñez, Henrik Teller, Pascal Ellerbrock, Katharina Meier, Stefan Heitmeier, Adrian Tersteegen, Jan Stampfuss, Dieter Lang, Karl-Heinz Schlemmer, Martina Schaefer, Kersten M. Gericke, Tom Kinzel, Daniel Meibom, Martina Schmidt, Christoph Gerdes, Markus Follmann, and Alexander Hillisch

Bayer AG, Pharmaceuticals, Research and Development, 42133 Wuppertal, Germany; susanne.roehrig@bayer.com

**CONTENT**

(A) General methods, crystallographic data for compounds **1–3**, **34**, and asundexian (**80**), *in vitro*, *ex vivo*, and *in vivo* pharmacology assays, and DMPK assays;

(B) $^{1}$H NMR and LC/MS data of intermediates **86–94** (Scheme 1), and $^{1}$H NMR, $^{13}$C NMR, LC/MS, and chiral HPLC data of asundexian (**80**);

(C) Synthesis of compounds **3–79**, **81–85**, **355**, and **362**;

(D) LC/MS data of compounds **1–85**, **355**, and **362**;

(E) Molecular Formula String Spreadsheet of compounds **1–85**, **355**, and **362** with FXIa $IC_{50}$ values.

**(A) General Methods, Cystallographic Data for Compounds 1, 2, 3, 34, and Asundexian (80), *In Vitro, Ex Vivo,* and *In Vivo* Pharmacology Assays, and DMPK Assays.**

**Druggability Assessment.** For the modeling of oral druggability, the SiteMap[1] program was applied. Fifteen site points were required to detect a ligand binding pocket. The standard grid for site points was used, together with the more restrictive hydrophobicity definition. The Dscore was used to compare oral druggability of different serine proteases. X-ray structures were prepared using Schrödinger's Maestro protein preparation routine.[2] WaterMap calculations were performed using the same X-ray structures.[3,4]

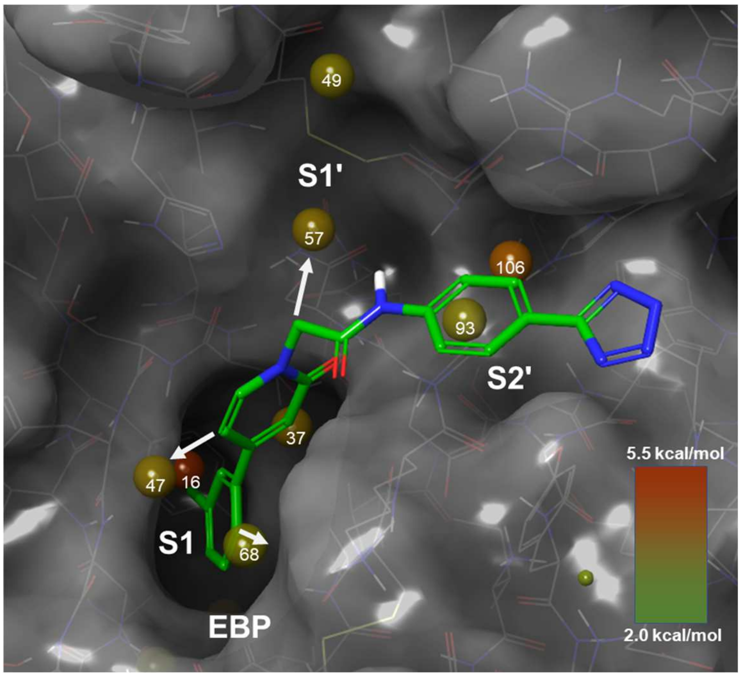


| Site | Occupancy | Overlap | dH | -TdS | dG | #HB(WW) | #HB(PW) |
|---|---|---|---|---|---|---|---|
| 16 | 0.96 | 0.58 | 1.59 | 3.65 | 5.24 | 1.03 | 1.13 |
| 106 | 0.30 | 1.00 | 3.09 | 0.91 | 4.00 | 1.27 | 0.89 |
| 37 | 0.66 | 1.00 | 0.60 | 2.56 | 3.16 | 1.64 | 0.94 |
| 57 | 0.52 | 0.00 | 1.52 | 1.53 | 3.05 | 1.82 | 0.26 |
| 47 | 0.60 | 0.00 | 1.23 | 1.78 | 3.01 | 1.99 | 0.39 |
| 49 | 0.58 | 0.00 | 0.69 | 2.06 | 2.75 | 1.53 | 1.63 |
| 93 | 0.32 | 1.00 | 1.81 | 0.89 | 2.70 | 2.17 | 0.00 |
| 68 | 0.45 | 1.00 | 1.34 | 1.22 | 2.56 | 1.87 | 0.07 |

**Figure S1.** WaterMap hydration sites in the substrate binding pocket of FXIa.

**Physicochemical Property Estimation.** Lipophilicity of compounds was estimated using $clogD^{7.5}$.[5] Polar surface area was characterized with the tPSA[6] descriptor. The corrected molecular weight ($MW_{corr}$)[7] was used to quantify molecule size. $MW_{corr}$ applies appropriate weight corrections for molecules with halogens because these atoms have an unproportionally low volume compared to their atomic weight. Thus, it is a surrogate parameter for the molecular volume and related to diffusion rates.

The ligand efficiency (LE) was calculated as $LE = pIC_{50} \times MW_{corr}^{-1} \times 1000$, and the ligand lipophilicity efficiency (LLE) was calculated as $LLE = pIC_{50} - clogD^{7.5}$.

**Docking and Energy Minimization in Factor XIa.** Glide SP Version 3.5[8] (Schrödinger LLC) was used to dock compounds into prepared FXIa X-ray structures. The X-ray structure of human FXIa in complex with ligand **2** was used for the majority of the docking studies. The ligand was placed in the center of a 22 Å box to calculate the interaction grid. The van der Waals scaling factor was set to 0.8 and the partial charge cutoff to 0.15. The ligands were docked flexibly and nonplanar amide bonds were penalized. 10000 poses per docking run were sampled. The complex with the most probable binding pose after manual inspection of top-ranked poses was energy-minimized using the OPLS2.0[9] force field (dielectric constant 1.0, constant dielectric, solvent water, Polak–Ribière conjugate gradient, convergence threshold 0.5) in MacroModel (Schrödinger LLC). The same settings were used for the analyses of the P1 dihedral angle. Compounds (unbound in water) were energy-minimized to the next local minimum in implicit water solvent.

**Relative Binding Free Energy Calculations.** Experimental $IC_{50}$ values were converted into $\Delta G$ values using $\Delta G = RT\ln K_i$ with $K_i \approx IC_{50}$. The in-house X-ray cocrystal structure of compound **34** in complex with human FXIa was prepared using the Protein Preparation Wizard[10] (Schrödinger Release 2015-1[11]) with missing side chains being filled using Prime.[12] Ligands were manually built based on the X-ray coordinates of cocrystallized **34** using Maestro (Release 2015-1). Binding free energy calculations were performed using the FEP/REST algorithm[13] (Release 2015-1) and the OPLS2.1 force field. The Force Field Builder was used to calculate missing ligand torsional parameters (Release 2015-1). The simulation time per lambda window was 20 ns with 12 lambda windows per perturbation. All simulations were run in the NPT ensemble. $\Delta G$ values were calculated from $\Delta\Delta G$ values.

**Prioritization and selection of EBP residues for synthesis, supported by relative binding free energy (FEP+) calculations using the OPLS2.1 force field.** FEP+ calculations were

performed for EBP analogues of the P2' carboxyphenyl amide instead of the substituted indazol-5-yl amide. For better comparison to the experimental data presented in this study, we recalculated relative binding free energies for the EBP substituents listed in Table 5 with (*S*)-methoxyethyl as P1' substituent. Figure 6 shows the corresponding correlation between calculated and experimental binding affinities. With a mean unsigned error (MUE) of 1.02 ± 0.26 kcal/mol, the accuracy is slightly lower than the average accuracy observed for all calculations performed during the project but can still be considered representative. Note that here, we could only compare to experimental values for racemic mixtures. The observed outlier at $\Delta G_{calc}$ = -8.69 ± 0.27 kcal/mol corresponds to imidazole derivative **56** and can be associated with a force field error. Using a later version of the force field (OPLS4[14]) in 2022, the value improved to $\Delta G_{calc}$ = -10.76 ± 0.16 kcal/mol.

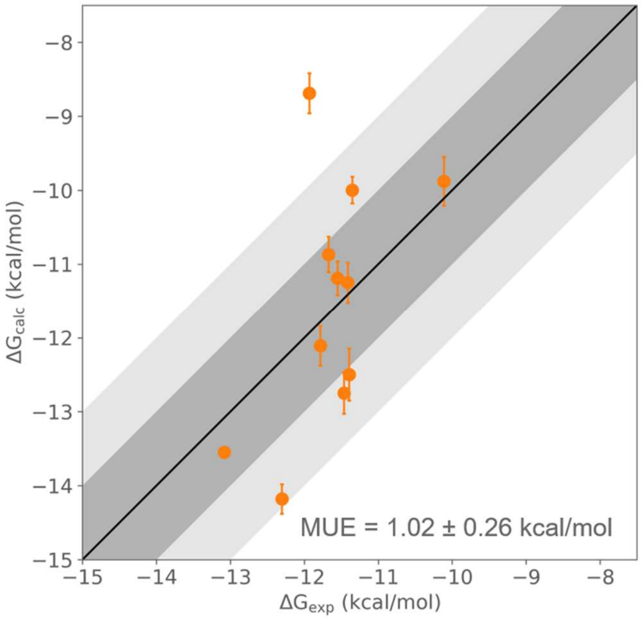


**Figure S2.** Calculated vs experimental relative binding free energies for EBP substituents listed in Table 5 with (*S*)-methoxyethyl as P1’ substituent. Dark gray and light gray areas mark 1 and 2 kcal/mol error ranges, respectively. The outlier in the upper left part of the correlation plot can be explained by a force field error.

**X-ray Crystallography.** Cocrystal structures of compounds **1**, **2**, and **3** in complex with human FXIa were obtained by Proteros Biostructures GmbH. Crystallographic data are listed in Table S1.

**Crystallization and Complex Formation of 34 and Asundexian (80).** FXIa was purchased from Proteros Biostructures GmbH. The protein was concentrated to ~30 mg/mL in 20 mM Tris/HCl buffer at pH 7.5 and 75 mM NaCl solution and was incubated with ~2 mM of inhibitor for ~1 h on ice. Prior cocrystallization, the solution was ultracentrifugated for ~2 min. Cocrystallization was performed using vapor diffusion in hanging drops, using equal volumes of protein solution and reservoir solution (0.1 M citrate buffer, pH 4.5, 20–30% PEG 4000) at RT. Seeding was performed using a cat whisker. In brief, one crystal of FXIa was transferred into 50 $\mu$L of reservoir solution in a seed bead tube. The tube was vortexed for ~90 s. The cat whisker was briefly dipped into the seed solution and pulled through the crystallization drop (streak seeding). After 1–3 d, crystals grew along the streak seeding line.

**Data Collection, Data Processing, and Data Refinement.** For data collection of asundexian (**80**), a crystal was plunged into a solution containing 25% glycerol and was mounted at 100 K on a Rigaku 007 diffractometer equipped with a Pilatus photon-counting detector. Data were processed using the software HKL3000.[15] For compound **34**, the frozen crystal was sent to beamline P14 in Hamburg, Germany and data were processed using XDS.[16] Structure solution was performed using the program PHASER[17] with a known in-house structure. Refinement was performed using the program REFMAC5.[18] The ligand was generated using the program PRODRG[19] and docked into the electron density within the program COOT.[20] Data collection and refinement statistics are summarized in Table S1.

**Table S1. Data Collection and Refinement Statistics** (values in brackets refer to the highest resolution shell)

| **Compound number** | **1** | **2** | **3** | **34** | **80 (asundexian)** |
|---|---|---|---|---|---|
| PDB ID | 8BO4 | 8BO6 | 8BO5 | 8BO7 | 8BO3 |
| **Data collection and processing** | | | | | |
| Wavelength [Å] | 1.000 | 1.000 | 0.97887 | 0.9762 | 1.54178 |
| Space group (no.) | *P*2(1)2(1)2(1) | *P*2(1)2(1)2(1) | *P*2(1)2(1)2(1) | *P*2(1)2(1)2(1) | *P*2(1)2(1)2(1) |
| Unit cell parameters, *a*, *b*, *c* [Å] | 59.30<br>59.45<br>68.15 | 59.12<br>59.63<br>67.05 | 58.76<br>59.74<br>66.93 | 59.76<br>60.67<br>67.47 | 58.19<br>59.92<br>66.82 |
| Resolution limit [Å] | 44.81–1.75 (1.79–1.75) | 44.56–1.25 (1.28–1.25) | 44.59–1.70 (1.75–1.70) | 42.57–1.25 (1.30–1.25) | 44.61–1.84 (1.92–1.84) |
| No. of reflections | 97829 (6633) | 518455 (37579) | 181634 (10011) | 904273 (95622) | 56426 (4266) |
| No. of unique reflections | 24732 (1678) | 65835 (4814) | 26387 (2026) | 68020 (7341) | 20404 (1939) |
| Multiplicity | 3.9 (4.0) | 7.9 (7.8) | 6.9 (4.9) | 13.3 (13.0) | 2.8 (2.2) |
| $I/\sigma(I)$ | 19.02 (3.10) | 23.09 (2.75) | 15.76 (2.42) | 17.1 (2.5) | 13.2 (1.8) |
| $R_{meas}$ [%] | 4.8 (51.4) | 4.9 (82.5) | 8.2 (74.2) | 8.1 (114.0) | 8.2 (57.4) |
| CC(1/2) | 99.9 (89.3) | 100 (82.4) | 99.8 (75.2) | 99.9 (78.5) | 99.7 (72.3) |
| Completeness [%] | 98.5 (92.7) | 99.4 (99.4) | 99.3 (93.7) | 99.3 (98.2) | 98.3 (94.9) |
| **Refinement** | | | | | |
| $R_{work}/R_{free}$ [%] | 16.20/18.92 | 11.61/14.07 | 15.39/17.36 | 11.72/15.70 | 16.96/20.78 |
| RMSD bond length [Å] | 0.010 | 0.013 | 0.010 | 0.015 | 0.015 |
| RMSD bond angles [deg] | 1.58 | 1.66 | 1.54 | 1.81 | 1.86 |
| *B* factors [$Å^2$] | 29.80 | 20.38 | 29.10 | 19.07 | 25.49 |
| Ramachandran favored (%) | 98.2 | 98.3 | 98.3 | 97.9 | 98.3 |
| Ramachandran allowed (%) | 1.8 | 1.7 | 1.7 | 2.1 | 1.7 |
| Ramachandran outliers (%) | 0.0 | 0.0 | 0.0 | 0.0 | 0.0 |

**Solubility.** The aqueous solubility screening during lead modification was performed from a DMSO stock solution (10 $\mu$L, 50 mg/mL), which was equilibrated with phosphate-buffered saline (PBS, 960 $\mu$L) at pH 6.5 for 24 h at RT. After ultracentrifugation, the supernatant was analyzed by LC/MS, using DMSO solution-based calibration. Alternatively, the solid compound was suspended in PBS buffer and stirred for 24 h at RT. Ultracentrifugation provided a supernatant, which was analyzed by HPLC (UV absorption), based on calibration curves of the test compound using DMSO solutions.

**Pharmacology.**

**Inhibition of Human FXIa and Selectivity Versus Other Serine Proteases.** The potency of test compounds against human FXIa activity in buffer was analyzed in biochemical assays based on the fluorometric detection of aminomethylcoumarine (AMC) cleaved from a synthetic peptidic substrate by FXIa. Test compounds were diluted in dimethylsulfoxide to a final assay concentration of 0–50 $\mu$M. Human FXIa (Kordia, Leiden, The Netherlands) and the FXIa substrate Boc-Glu(OBzl)-Ala-Arg-AMC (Bachem I-1575) were diluted in buffer solution (pH 7.4) consisting of 50 mM Tris/HCl, 100 mM NaCl, 5 mM $CaCl_2$, and 0.1% bovine serum albumin to a final concentration of 0.15 nM and 5 $\mu$M, respectively. Diluted test compound or DMSO (1 $\mu$L) in assay buffer (20 $\mu$L), diluted FXIa solution (20 $\mu$L), and substrate solution (20 $\mu$L) were added to 384-well microtiter plates (Greiner Bio-One, Frickenhausen, Germany). Fluorescence (excitation 360 nm, emission 460 nm) was measured using a microtiter plate fluorescence reader (Safire II, Tecan, Männedorf, Switzerland) for 30 min at RT. The concentration of test compounds producing 50% inhibition of FXIa activity ($IC_{50}$) was determined *via* a nonlinear logistic regression model. $K_i \approx IC_{50}$ applies as $IC_{50}$ values were determined at substrate concentrations much lower than the substrate $K_M$.

The selectivity of test compounds versus other serine proteases, mostly of the hemostatic system, was evaluated in buffer in biochemical assays based on the fluorometric detection of AMC released from specific substrates for respective proteases as described above. The active proteases or zymogens, typically purified from human plasma, and corresponding substrates are commercially available. All enzymes and substrates were diluted in assay buffer (50 mM Tris/HCl, pH 7.4, 100 mM NaCl, 5 mM $CaCl_2$, 0.1% bovine serum albumin), unless otherwise specified. Serine protease assays were comprised of the following enzymes and substrates (final assay concentrations are given): thrombin (Kordia; 0.02 nM), Boc-Asp(OBzl)-Pro-Arg-AMC (Bachem I-1560; 5 $\mu$M); factor VIIa (Kordia; 0.5 pM) + tissue factor (HemosIL, Bedford, MA; RecombiPlasTin 2G; 1:1000), substrate mix: Boc-Ile-Glu-Gly-Arg-AMC (Bachem I-1100;

50 $\mu$M) + factor X (Kordia; 0.07 U/mL); factor IXa (Kordia; 0.08 U/mL, 8.8 nM), substrate mix: Boc-Ile-Glu-Gly-Arg-AMC (Bachem I-1100; 50 $\mu$M) + factor X (Kordia; 0.07 U/mL, 12 nM) [Factor IXa was diluted in 'lipid buffer' instead of assay buffer. Preparation of 'lipid buffer': Evaporate the solvent from a lipid solution of 1,2-dioleoyl-sn-glycero-3-[phospho-L-serine] (20 mg/mL in chloroform, Avanti Polar Lipids) under a nitrogen flow in a beaker. Prepare 0.2 mg/mL 1,2-dioleoyl-sn-glycero-3-[phospho-L-serine] + 0.6 mg/mL 1,2-dioleoyl-sn-glycero-3-phosphocholine (100 mg/mL in water, Sigma) by addition of buffer (50 mM Tris/HCl, pH7.4, 100 mM NaCl) and stir slowly at 4 °C overnight using a magnetic stirrer. Store 'lipid buffer' at 4 °C.]; factor Xa (Kordia; 1.3 nM), Boc-Ile-Glu-Gly-Arg-AMC (Bachem I-1100; 5 $\mu$M); factor XIIa (Loxo; 10 nM), H-Pro-Phe-Arg-AMC (Bachem I-1295; 5 $\mu$M); plasma kallikrein (Kordia; 0.2 nM), H-Pro-Phe-Arg-AMC (Bachem I-1295; 5 $\mu$M); plasmin (Kordia; 0.1 $\mu$g/mL, 1.2 nM), MeOSuc-Ala-Phe-Lys-AMC (Bachem I-1275; 50 $\mu$M); urokinase (uPA, Kordia; 0.7 nM), Z-Gly-Gly-Arg-AMC (Bachem I-1140; 5 $\mu$M); tissue plasminogen activator (tPA, Loxo; 1 nM), $CH_3SO_2$-D-Phe-Gly-Arg-AMC (Pentapharm 091-06; 5 $\mu$M); activated protein C (Kordia; 10 nM), Boc-Leu-Ser-Thr-Arg (Sigma B4636; 50 $\mu$M); trypsin (Sigma; 0.042 U/mL), Boc-Ile-Glu-Gly-Arg-AMC (Bachem I-1100; 5 $\mu$M); chymotrypsin from human pancreas (Merck, 0.1 nM), *N*-succinyl-Leu-Leu-Val-Tyr-AMC (Merck S6510; 50 $\mu$M); caldecrin (chymotrypsin C, Cell Sciences; 0.13 nM), *N*-succinyl-Leu-Leu-Val-Tyr-AMC (Merck S6510; 50 $\mu$M).

***In Vitro* and *Ex Vivo* Effect of Compounds in Plasma on the Clotting Time after Contact Activation (aPTT Assay).** Citrated plasma samples were either obtained from Octapharma (Langenfeld, Germany) in the case of human plasma, or prepared from different animal species by drawing whole blood into citrate solution and subsequent centrifugation. Solutions of the compounds in DMSO or DMSO alone were added to the plasma samples to yield a 1:50 DMSO/plasma mixture and a concentration range of the compound in the final assay volume of 150 $\mu$L of 0–37.5 $\mu$M. After a 3 min incubation at 37 °C, 50 $\mu$L aliquots were incubated for another 3 min with 50 $\mu$L of an aPTT reagent (C.K. Prest, Diagnostica Stago, Asnières sur Seine Cedex, France). The coagulation process was initiated by addition of 50 $\mu$L of 0.025 M $CaCl_2$, and the clotting time was measured by an automated coagulometer (AMAX 200, Trinity Biotech, Lemgo, Germany). The compound concentrations in the final assay volume of 150 $\mu$L which produce a 1.5-fold increase in the clotting time are reported as the $EC_{150}$ in the compound modification phase (mean of two measurements). For the in-depth evaluation of compound **34** and asundexian (**80**), the procedure was repeated eightfold. In order to be able to compare the

effects to other plasma assays with a different final assay volume, the $EC_{150}$ values of compound **34** and asundexian (**80**) in plasma (50 $\mu$L; 1/3 of the final assay concentration) were calculated as well. For *ex vivo* measurements of the aPTT prolongation in animal experiments, the assay was conducted as described above without addition of any compound or DMSO.

***In Vivo* Models of Thrombosis ($FeCl_2$ Injury) and Bleeding Time in Rabbits.** The antithrombotic effects of compound **34** and asundexian (**80**) were assessed in rabbit arterial thrombosis models in preventive settings. In addition, the impact on the bleeding time after a standardized incision at a rabbit ear was determined simultaneously (in the same animal at the same time). Several series of experiments were performed: First, various intravenous and oral regimens of asundexian were compared with intravenous rivaroxaban in an arterial thrombosis model. Another series investigated the impact of oral administration and a combination of intravenous asundexian plus dual antiplatelet treatment [DAPT; aspirin (acetylsalicylic acid) and ticagrelor] versus DAPT alone or apixaban plus DAPT in the same model. In addition, the impact of the compounds in a venous thrombosis model was investigated.

*Antithrombotic Effect after $FeCl_2$-Induced Injury of the Carotid Artery and Intravenous or Oral Administration of Compounds Alone or in the Presence of Aspirin and Ticagrelor, in Combination with an Ear Bleeding Time Model.*

Anesthesia was induced and maintained in male New Zealand white rabbits (2.7–3.0 kg) with ketamine and xylazine (intramuscular bolus of 40 mg/kg + 5 mg/kg and 800 mg + 80 mg in 12 mL with an infusion rate of 5 mL/h *via* an ear vein, respectively). The femoral artery and vein were exposed and cannulated for arterial blood sampling and intravenous drug administration, respectively. Thrombosis was induced 30 min after treatment by exposing the carotid artery to $FeCl_2$ (13% in water; Merck, Darmstadt, Germany) on a filter paper for 5 min. Thirty minutes after application of $FeCl_2$, the injured artery was excised, and the thrombus removed for weighing.

Simultaneously to thrombus generation, ear bleeding was initiated by making a standardized 5 mm skin cut using a scalpel blade parallel to the external vein of the ear, avoiding direct injury of a visible vessel. Blood was removed gently by swabbing with a filter paper at 10-second intervals. Bleeding time was recorded as the time at which bleeding stopped.

At the start of the thrombosis experiment, blood was drawn and collected in 2.9 mL citrated tubes (Sarstedt, Nümbrecht, Germany). Plasma was obtained by centrifugation at 4000 g for 10 min at 4 °C and was stored at -20 °C. The aPTT was measured.

*Antithrombotic Effect after $FeCl_2$-Induced Injury of the Jugular Vein and Intravenous Administration of Compounds.*

Anesthesia was induced and maintained in male New Zealand white rabbits (2.7–3.0 kg) using the same regimen as described above. Thrombosis was induced 30 min after intravenous treatment with of compound **34** or asundexian by exposing the jugular vein to $FeCl_2$ (13% in water, Merck) on a filter paper for 5 min. After an additional 30 min, the injured part of the vein was excised, and the thrombus removed for weighing. The effect of drug treatment on ear bleeding was assessed as described above.

*Statistical Methods.* Descriptive statistics were prepared for all variables after confirmation of normality (*in vitro*: mean ± standard error of the mean [SEM] or standard deviation [SD]; *in vivo*: median with 25th and 75th percentiles). Differences in thrombus weights and in bleeding times between asundexian and comparators were analyzed by Kruskal–Wallis analysis followed by Dunn's multiple comparison tests. Graphs were prepared and all statistical analyses were performed using GraphPad Prism software and adjusted alpha errors of $P < 0.05$ were considered statistically significant.

**Drug Metabolism and Pharmacokinetics.**

***In Vitro* Metabolic Stability in Hepatocytes.** Incubations with hepatocytes were performed at 37 °C and pH 7.4 in a total volume of 1 mL using a modified Microlab Star robotic system (Hamilton) or Janus robotic system (PerkinElmer). The incubation mixtures contained $1 \times 10^6$ cells/mL (corrected for cell viability, determined by microscopy after staining with trypan blue), 1 $\mu$M substrate, and Williams' medium E (Sigma). The final acetonitrile concentration was ≤1%. Aliquots of 70 $\mu$L were withdrawn from the incubation mixture after 2, 10, 20, 30, 50, 70, and 90 min and dispensed in a 96-well plate containing 200 $\mu$L acetonitrile to stop the reaction. After centrifugation, the supernatants were analyzed by LC/MS/MS (AB Sciex Triple Quad 5500 system). The calculation of *in vitro* clearance values from half-life data of the compounds, reflecting substrate depletion, was performed using the following equation:

$$CL'_{intrinsic} = \frac{0.693}{t_{½\ in\ vitro}} \times \frac{M_{liver,spec} \times N_{hepatocytes,spec} \times V_{incub}}{N_{hepatocytes,incub}}$$

where $M_{liver,spec}$ is the body weight normalized species-specific liver weight (human: 21 g/kg, rat: 32 g/kg), $N_{hepatocytes,spec}$ is the liver weight normalized number of hepatocytes in the liver ($110 \times 10^6$/g for all species), $N_{hepatocytes,incub}$ is the number of hepatocytes used in the incubation experiment, and $V_{incub}$ is the total volume in the incubation experiment.

The blood clearance was estimated using the nonrestricted (without considering protein binding in blood and incubation mixture) well-stirred model:

$$CL_{blood,well\,stirred} = \frac{Q_H \times CL'_{intrinsic}}{Q_H + CL'_{intrinsic}}$$

where $Q_H$ is the body weight normalized species-specific liver blood flow (human: 1.3 L/h/kg, rat: 4.2 L/h/kg).

The maximum possible bioavailability ($F_{max}$) was calculated using the formula:

$$F_{max,\ well\,stirred} = \left(1 - \frac{CL_{blood,well\,stirred}}{Q_H}\right) \times 100\%$$

**Permeability and Efflux in Caco-2 Cells.** Caco-2 cells (obtained from the Deutsche Sammlung für Mikroorganismen und Zellkulturen, DSMZ) were cultured in 24-well Transwell plates for 15 or 16 d. Tests were carried out using a Hamilton robot. The density of the cell monolayers was ensured by measuring the lucifer yellow permeability. The test compounds were dissolved in DMSO and then diluted with assay buffer to a concentration of 2 $\mu$M (final DMSO concentration 1%). The permeability was examined in both directions by addition of the substance solutions to either the apical or basolateral compartment. The covered plates were incubated at 37 °C for 2 h. The concentrations in the two compartments were determined by LC/MS/MS and the $P_{app}$ values were calculated according to literature.[21] The efflux ratio describes the ratio of $P_{app}$ (B–A) / $P_{app}$ (A–B).

**CYP Inhibition Potential.** The inhibitory potency of test compounds was assessed *in vitro* by means of formation of metabolites from standard probes mediated by CYP isoforms based on assay conditions described in the literature.[22] To investigate time-dependent inhibition, preincubation experiments were performed.[23] Stock solutions of test compounds were prepared in acetonitrile (concentration = 20 mM).

Coincubation: 4 $\mu$L of the stock solution of the test compound was added to phosphate buffer (196 $\mu$L, 50 mM, pH 7.4) containing 1 mM EDTA. This mixture was sequentially diluted on a Starlet Workstation (Hamilton, Munich, Germany). After addition of 80 $\mu$L phosphate buffer (50 mM, pH 7.4) containing human liver microsomes and the respective substrate, the mixture was prewarmed (5–6 min). Reactions were initiated by addition of 20 $\mu$L phosphate buffer (50 mM, pH 7.4) containing NADP, glucose-6-phosphate, and glucose-6-phosphate dehydrogenase. Overall, mixtures contained human liver microsomes (pooled, XTreme200, XenoTech LLC, Lenexa, Kansas, USA) at protein concentrations defined in Table S2,

NADPH-regenerating system [1 mM NADP, 5 mM glucose-6-phosphate, glucose-6-phosphate dehydrogenase (1.5 U/mL)], 1 mM EDTA, test compound at six different concentrations (20, 10, 5, 2.5, 1.3, 0.63 $\mu$M), probe substrate at concentrations defined in Table S2 close to reaction $K_M$ values, and phosphate buffer (50 mM, pH 7.4) in a total volume of 200 $\mu$L. Incubations were performed on a Starlet Workstation (Hamilton, Munich, Germany) in 96-well plates (microtiter plate, 96-well polystyrene, nontreated; Corning, Wiesbaden, Germany) at 37 °C. Stock solutions of probe substrates were prepared in water (amodiaquine, 2 mM; diclofenac, 4 mM; dextromethorphan 5 mM; midazolam, 2.5 mM) or water/acetonitrile 1:1 (v/v, phenacetin, 45 mM). Substrates (with 1% acetonitrile) in the absence of test compound were incubated as reference in parallel (sextuple). Known direct-acting and metabolism-dependent inhibitors of the respective enzymes ('standard inhibitors') were included as positive controls, most of which appear on the FDA list of recommended or accepted *in vitro* inhibitors. Total acetonitrile concentration was 1%. Reactions were stopped by the addition of 100 $\mu$L acetonitrile containing the respective internal standard. Precipitated proteins were removed by centrifugation of the well plate (3000 rpm, 10 min) and supernatants were combined and analyzed by LC/MS/MS.

Preincubation (CYP3A4): Test compound at six different concentrations (20, 10, 5, 2.5, 1.3, 0.63 $\mu$M) was preincubated with human liver microsomes and the NADPH-regenerating system in the absence of the probe substrate midazolam for 30 min at 37 °C. Following preincubation, midazolam was added for the coincubations and the reactions were allowed to proceed. The known metabolism-dependent inhibitor mibefradil served as positive control. Total acetonitrile concentration was 1%. Reactions were terminated by the addition of 100 $\mu$L acetonitrile containing internal standard and the samples were prepared for LC/MS/MS analysis as described above.

Workup procedure: The main incubations were stopped with acetonitrile (about 33% final concentration) containing internal standard and cooled at 4–10 °C until analysis. Prior to analysis, the samples were centrifuged at 3000 rpm for 10 min to precipitate protein. From the supernatants, an aliquot of usually 10 $\mu$L was subjected to LC/MS/MS analysis.

**Table S2. CYP Incubation Conditions, Standards, and CYP Probes**

| CYP isoform | Substrate, concentration | Protein concentration (human liver microsomes) | Incubation time | ISTD | Standard inhibitor |
|---|---|---|---|---|---|
| 1A1 | Granisetron, 2.5 µM | 1 mg/ L (rec. hCYP1A1) | 15 min | 7'-Hydroxy[$^2H_3$]granisetron | 7-Hydroxyflavone |
| 1A2 | Phenacetin, 45 $\mu$M | 50 mg/L | 15 min | [$^2H_4$]Acetaminophen | Furafylline |
| 2A6 | Coumarin, 2 µM | 20 mg/L | 10 min | [$^2H_5$]Umbelliferone | Tranylcypromine |
| 2B6 | Bupropion, 100 µM | 250 mg/L | 30 min | [$^2H_6$]Hydroxybupropine | Ticlopidine |
| 2C8 | Amodiaquine, 2 $\mu$M | 9 mg/L | 15 min | [$^2H_5$]Desethylamodiaquine | Montelukast |
| 2C9 | Diclofenac, 4 $\mu$M | 9 mg/L | 15 min | 4'-Hydroxy-[$^{13}C_6$]diclofenac | Sulfaphenazole |
| 2C19 | (S)-Mephenytoin, 50 µM | 500 mg/L | 30 min | 4'-Hydroxy[$^2H_3$]mephenytoin | (-)-N-3-Benzylphenobarbital |
| 2D6 | Dextromethorphan, 5 $\mu$M | 50 mg/L | 15 min | [$^2H_3$]Dextromethorphan | Fluoxetine |
| 2E1 | Chlorzoxazone, 50 µM | 130 mg/L | 15 min | 6-Hydroxy[$^{13}C_6$]chlorzoxazone | Methylpyrazole |
| 2J2 | Ebastine, 2 µM | 0.22 mg/L (rec. hCYP2J2) | 15 min | 4'-Hydroxy-[$^{13}C_6$]diclofenac | HET0016 |
| 3A4 | Midazolam, 2.5 $\mu$M | 60 mg/L | 10 min | [$^{13}C_6$]1-hydroxymidazolam | Ketoconazole |

***In Vivo* Pharmacokinetic Studies.** The respective test substances were administered to animals as a bolus injection, by infusion, or *via* oral administration. The formulation for intravenous administration of the test substances was plasma/DMSO (rats) or polyethylene glycol/ethanol/water (dogs). The formulation for oral administration was polyethylene glycol/ethanol/water or solutol/ethanol/water or other formulations as appropriate (e.g., water, tylose, self-emulsifying drug dispering systems). The administration volume was 2–10 mL/kg (rats) or 0.5–5 mL/kg (dogs).

Blood samples were removed from the test animals into sodium EDTA (or other anticoagulant)-containing tubes: in the case of bolus administration, blood samples were usually taken at 0.083, 0.167, 0.25, 0.283, 0.333, 0.5, 0.75, 1, 2, 3, 5, 7, and 24 h after administration of the test substance. Other time points could be chosen as appropriate. After removal, the blood samples were centrifuged at 1280 *g* for 10 min. The supernatant (plasma) was taken off and either directly processed further or frozen for later sample preparation. For sample preparation, 50 $\mu$L plasma was mixed with 250 $\mu$L acetonitrile (also containing the internal standard ISTD for later analytical determination) and then allowed to stand at RT for 5 min. The mixture was then centrifuged at 16000 *g* for 3 min. The supernatant was taken off and 500 $\mu$L of a buffer suitable for the mobile phase was added. The samples were then examined by LC/MS/MS analysis [e.g., LC: Gemini 5 $\mu$M C18 110A 50 mm × 3 mm (or 150 mm × 3 mm) column (Phenomenex), MS: API 5500 or API 6500 system (Sciex, Canada)] to determine the concentration of the test substance in the individual samples.

The pharmacokinetic parameters were calculated by noncompartmental analysis (NCA). The algorithms for calculating the parameters are based on rules published in general textbooks on pharmacokinetics.

**Plasma Protein Binding.** The separation of protein bound and free (unbound) test substance was performed by dialysis across a semipermeable membrane with a pore size of 12–14 kDa. The free drug diffuses through the membrane into the buffer side until equilibrium is reached. The concentration of unbound drug can then be derived by measuring its concentration in the plasma and the buffer after a sufficient dialysis time.

Dialysis apparatus: 96-well dialysis apparatus, Teflon (HTDialysis, Gales Ferry, USA); dialysis membrane: regenerated cellulose membrane, pore size 12–14 kDa (HTDialysis, Gales Ferry, USA); dialysis buffer: Dulbecco's 20 mM PBS buffer, pH 7.4 (Sigma D8537); incubator: Heraeus BB 6060 with 7% carbon dioxide aeration (Kendro, Langenselbold, Germany); laboratory shaker: IKA VXR Vibrax (Janke & Kunkel, IKA Labortechnik, Staufen, Germany). Plasma- or protein-containing buffer solutions (150 µL) adjusted to pH 7.4 (either by thawing the plasma in an incubator with 7% $CO_2$ aeration or by exposing the plasma to a carbogen stream for 2 min) were dialyzed against buffer (150 µL) for 6 h at 37 °C. The concentration in the plasma (C) and the dialysis buffer ($C_u$) were determined *via* LC/MS/MS. These values were used for the calculation of the unbound fraction. The amount of organic solvent added to the plasma did not exceed 1% of the total incubation volume.

Before the study was started, the recovery of a low drug concentration added to PBS from the dialysis cells (including membrane) was investigated. Generally, a recovery ≥90% of the actual concentration in the assay was accepted.

The free (unbound) fraction was calculated according to:

$$f_u = \frac{C_u}{C} \cdot 100\,[\%]$$

The fraction of drug bound to the plasma proteins $f_b$ was calculated according to:

$$f_b = \frac{C_b}{C} \cdot 100\,[\%]$$

where $C_b = C - C_u$ concentration bound to proteins, C = total concentration,
$C_u$ = concentration in dialysate. No correction was made for volume shift (below 10%).

**(B) $^1$H NMR and LC/MS Data of Intermediates 86-94 (Scheme 1), and $^1$H NMR, $^{13}$C NMR, LC/MS, and Chiral HPLC Data of Asundexian (80).**

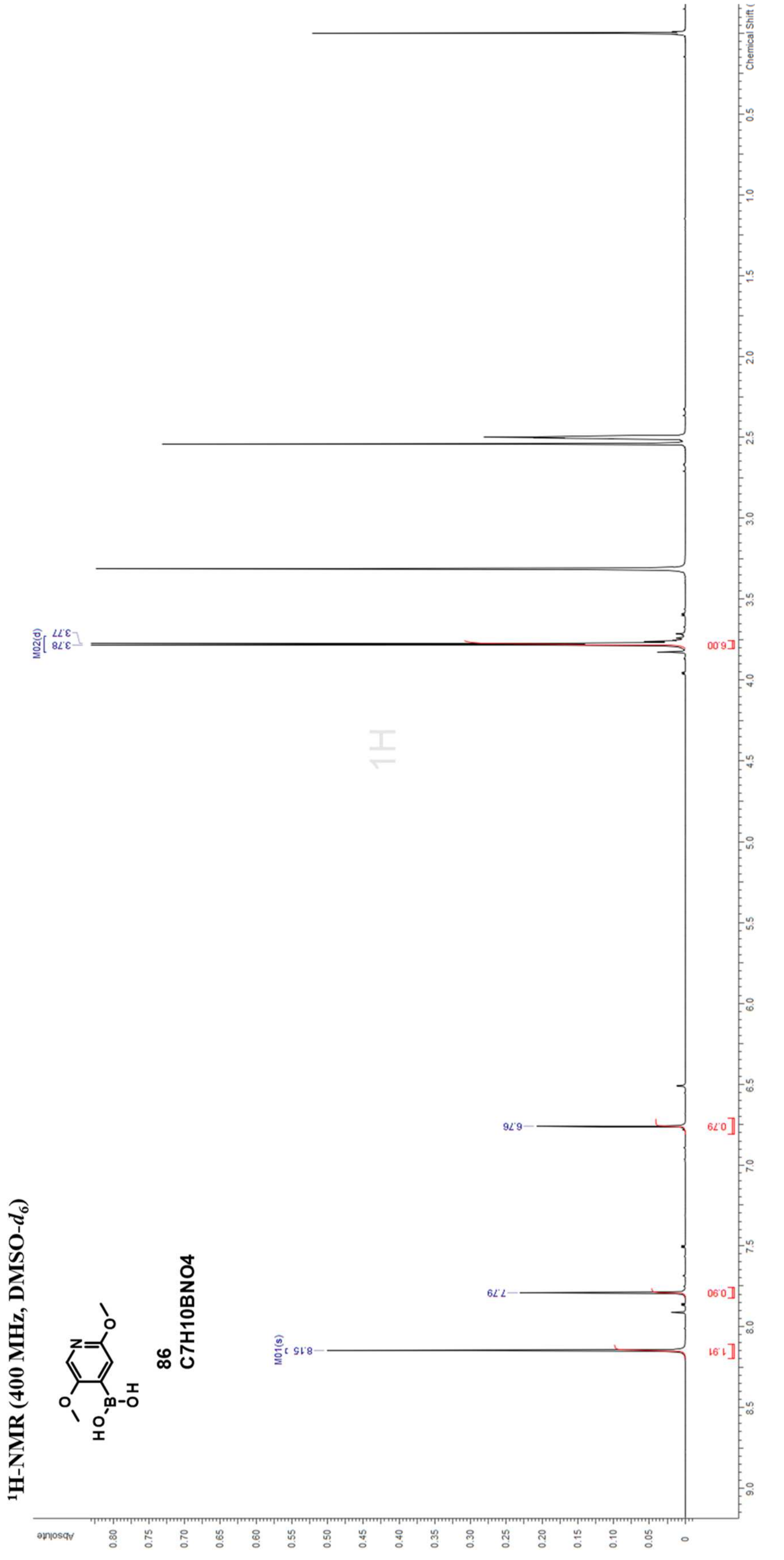

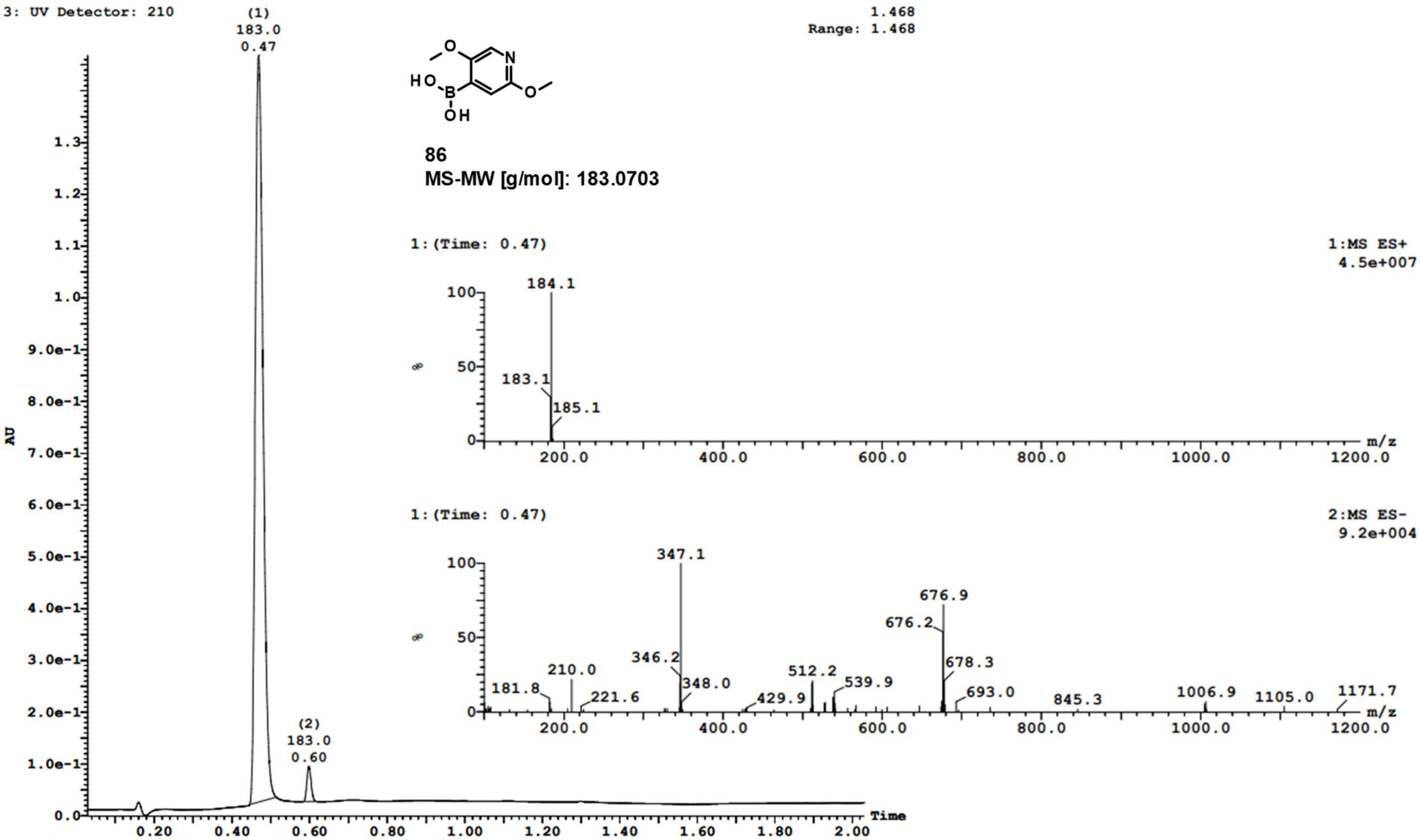


**86**
**MS-MW [g/mol]: 183.0703**

| Peak Number | Time | AreaAbs | Area %Total | Mass Found |
|---|---|---|---|---|
| 1 | 0.47 | 33441 | 97.48 | 183.00 |
| 2 | 0.60 | 866 | 2.52 | 183.00 |

$^1$H-NMR (400 MHz, DMSO-$d_6$)

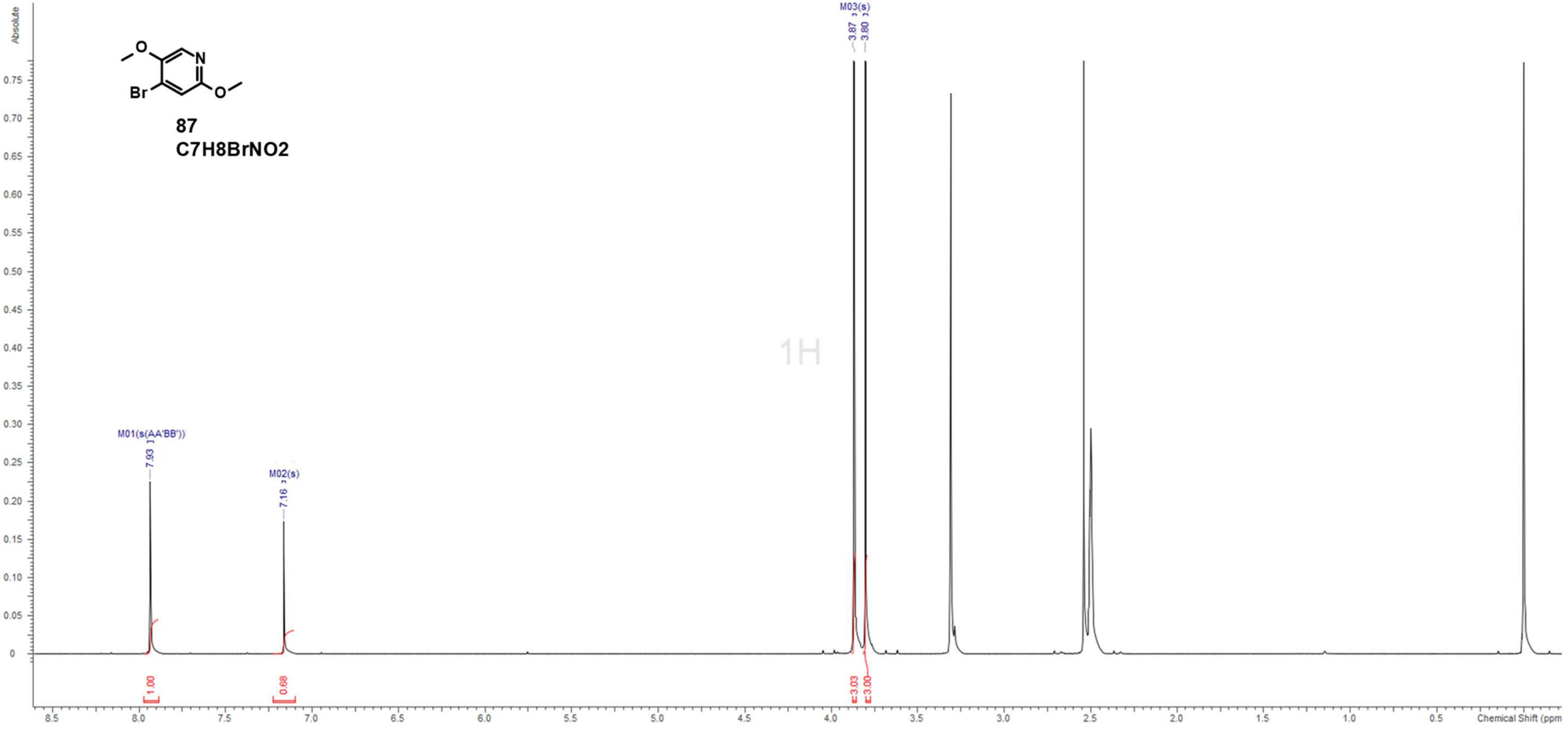

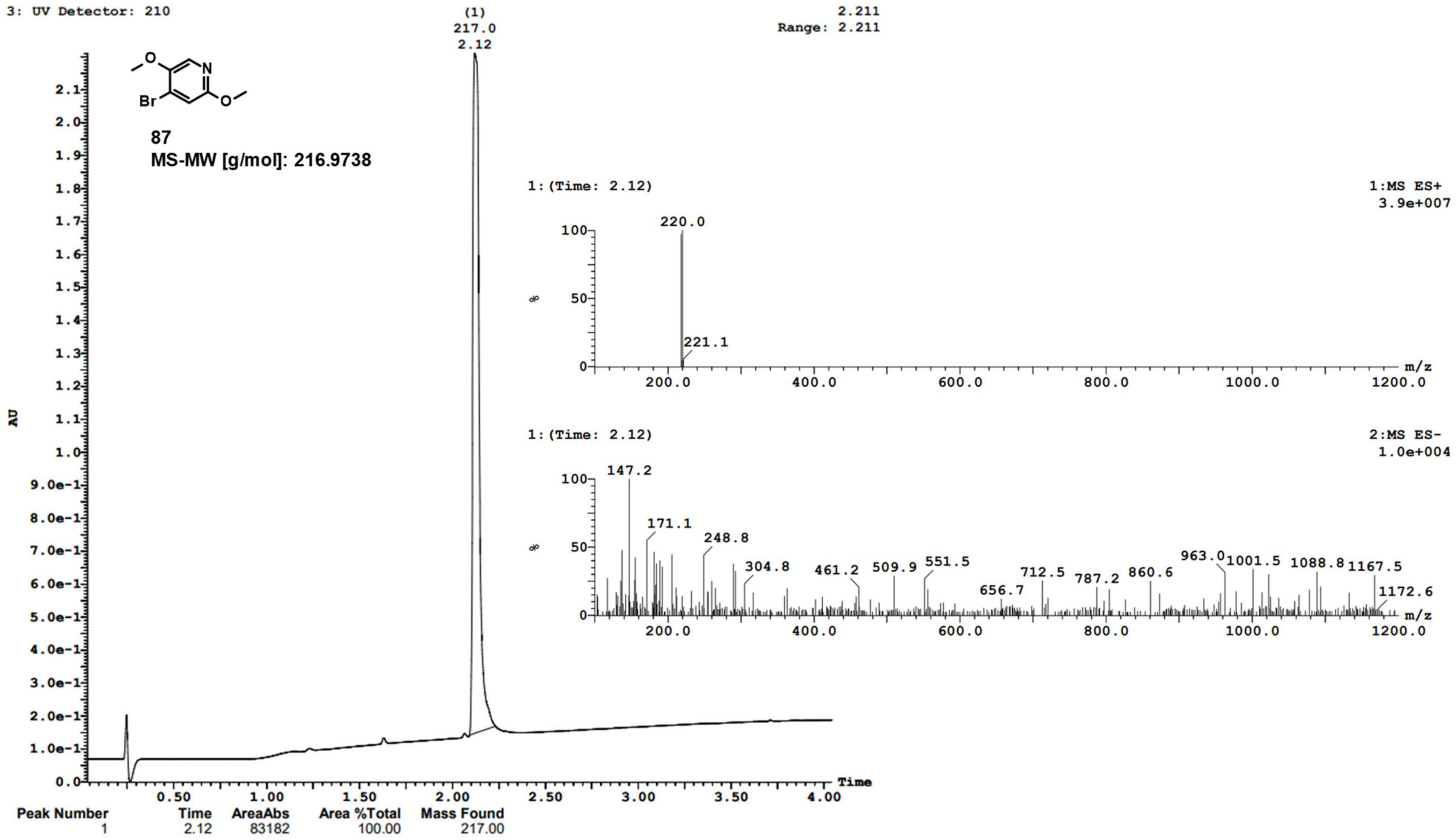
3: UV Detector: 210
(1)
217.0
2.12
2.211
Range: 2.211
87
MS-MW [g/mol]: 216.9738
AU
1:(Time: 2.12)
1:MS ES+
3.9e+007
220.0
221.1
1:(Time: 2.12)
2:MS ES-
1.0e+004
147.2
171.1
248.8
304.8
461.2
509.9
551.5
656.7
712.5
787.2
860.6
963.0
1001.5
1088.8
1167.5
1172.6
m/z
Time
Peak Number
1
Time
2.12
AreaAbs
83182
Area %Total
100.00
Mass Found
217.00

## $^1$H-NMR (400 MHz, DMSO-$d_6$)

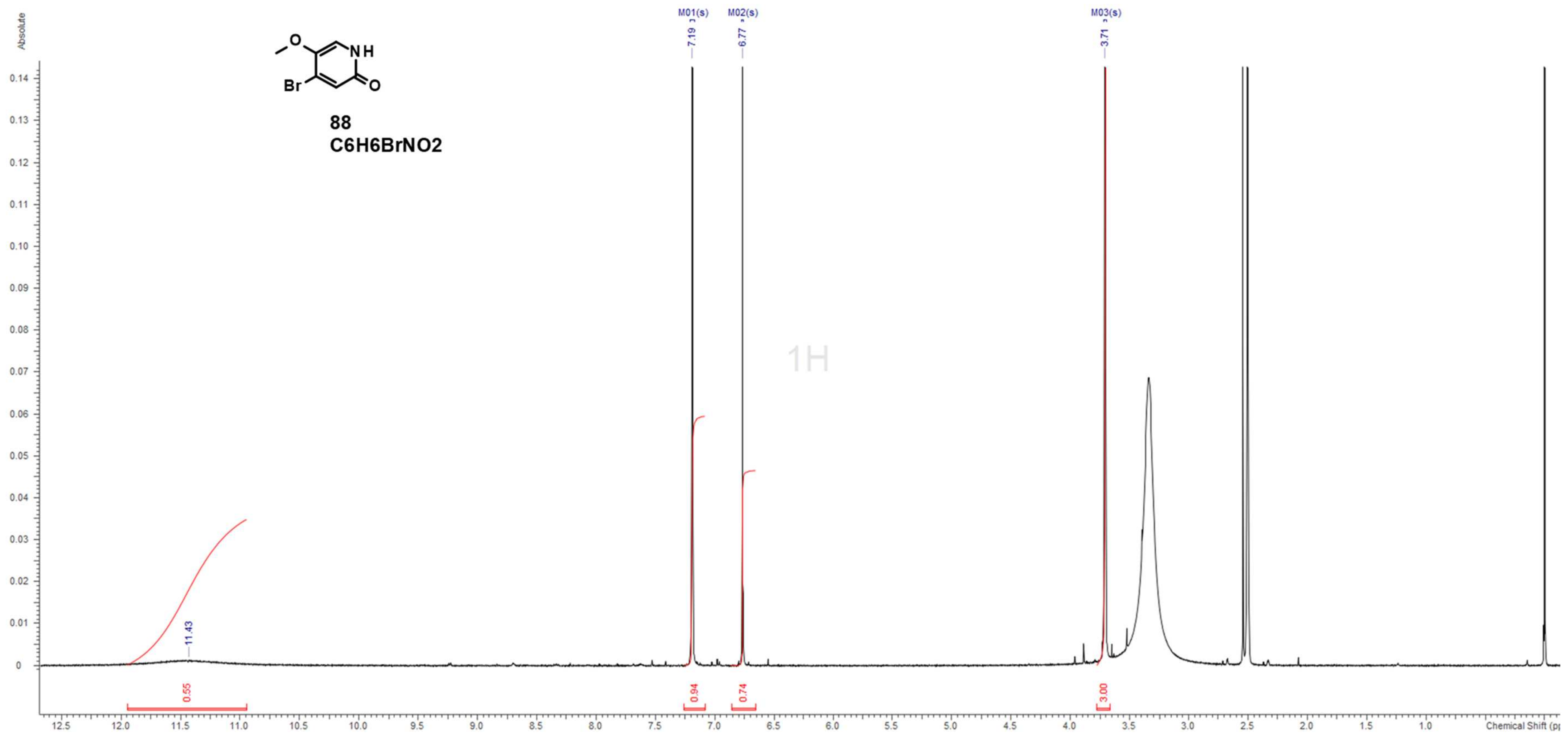

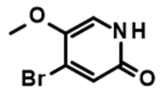

**88**
**MS-MW [g/mol]: 202.9582**

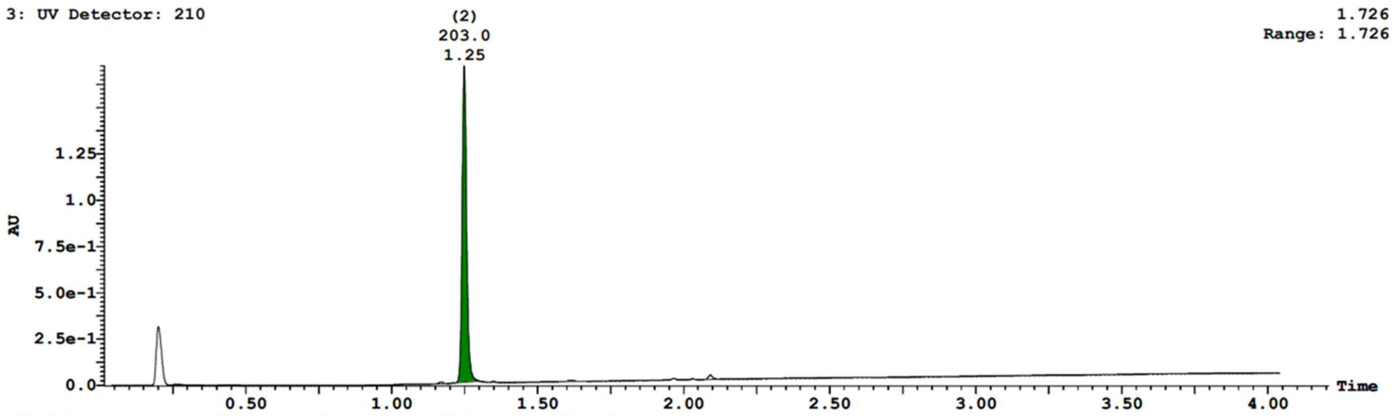


| Peak Number | Time | AreaAbs | Area %Total | Mass Found |
|---|---|---|---|---|
| 1 | 1.17 | 171 | 0.55 | |
| 2 | 1.25 | 30666 | 97.60 | 202.96 |
| 3 | 1.61 | 203 | 0.65 | |
| 4 | 2.09 | 381 | 1.21 | |

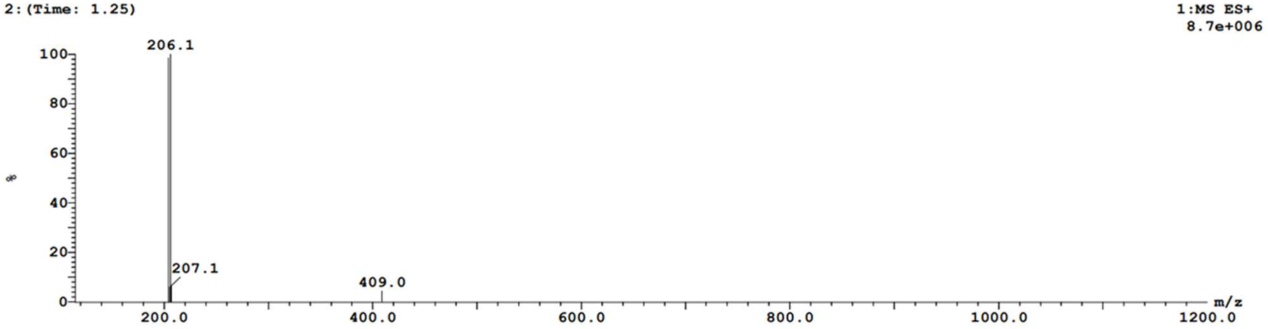


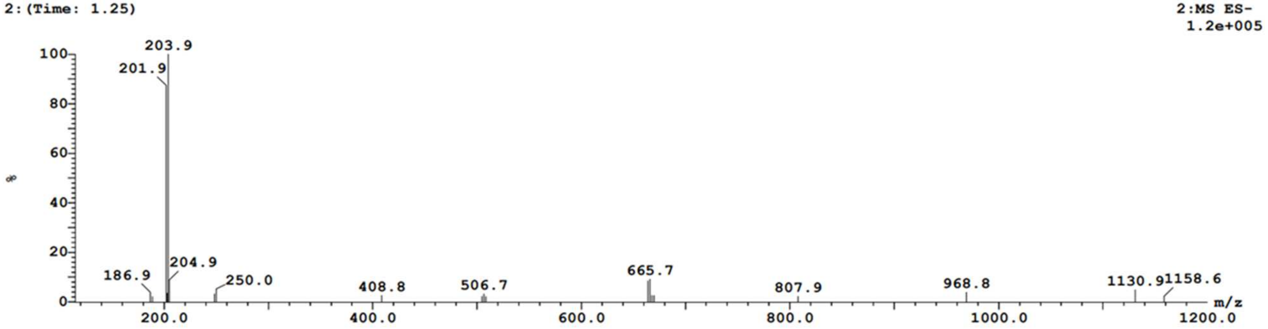

$^{1}$H-NMR (400 MHz, DMSO-$d_6$)

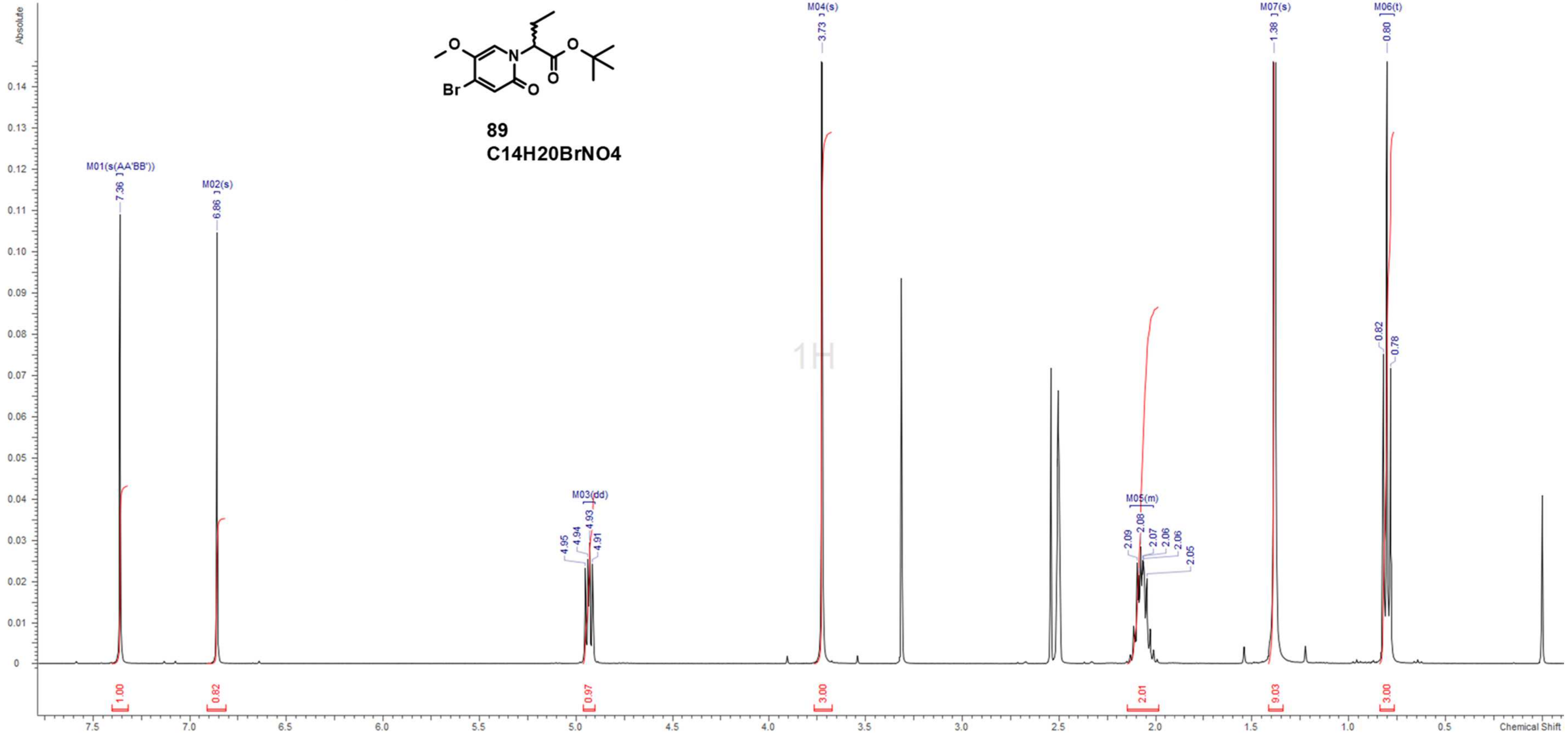

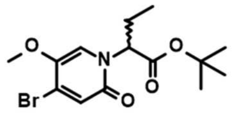

**89**
**MS-MW [g/mol]: 345.0576**

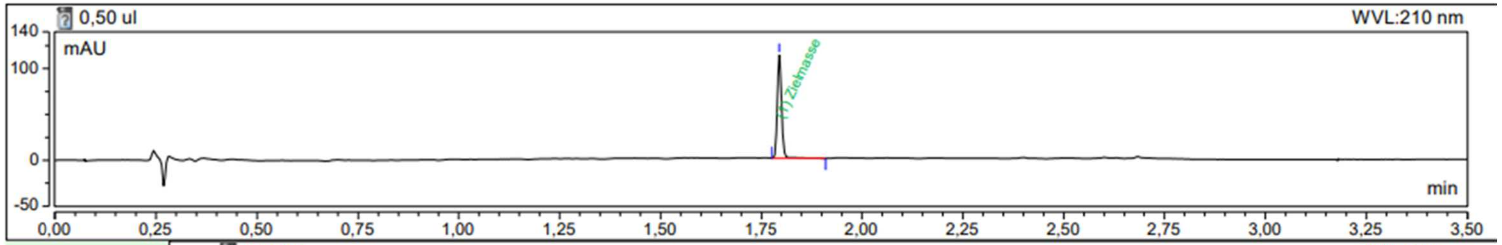


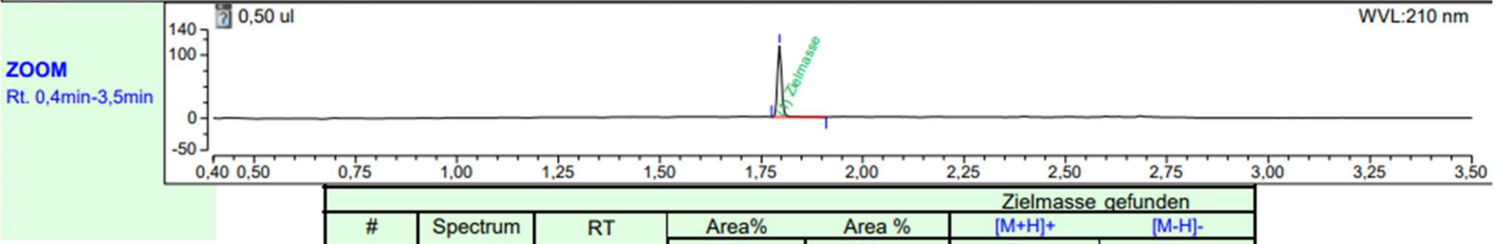


| # | Spectrum | RT min | Area% 210nm | Area % UVMax | Zielmasse gefunden [M+H]+ Pos | Zielmasse gefunden [M-H]- Neg |
|---|---|---|---|---|---|---|
| 1 | Y | 1,80 | 100,0 | 100,0 | 346,0649 | |

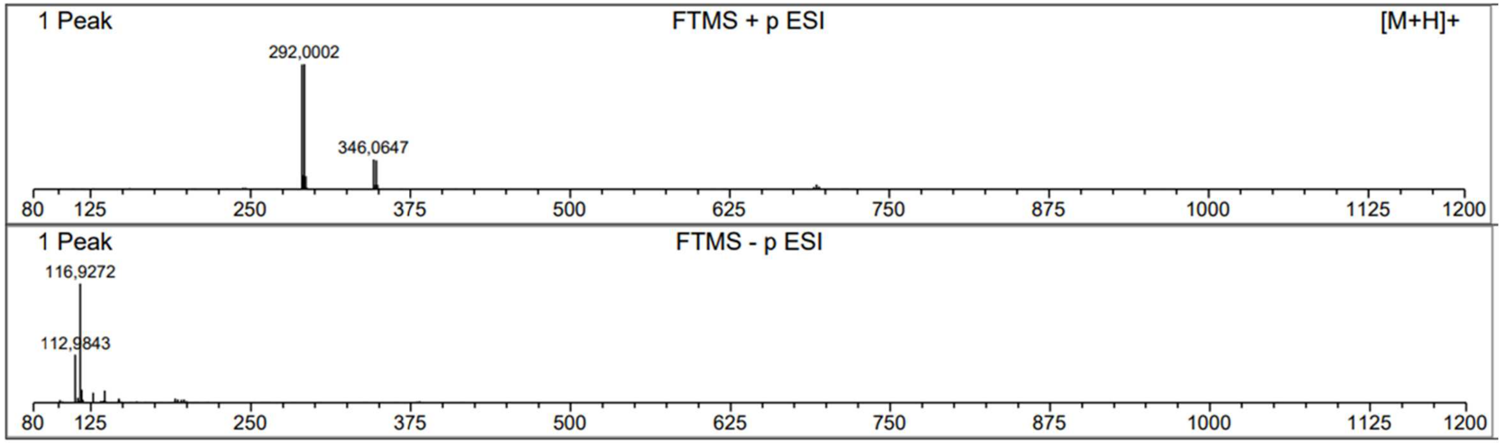

$^{1}$H-NMR (400 MHz, DMSO-$d_6$)

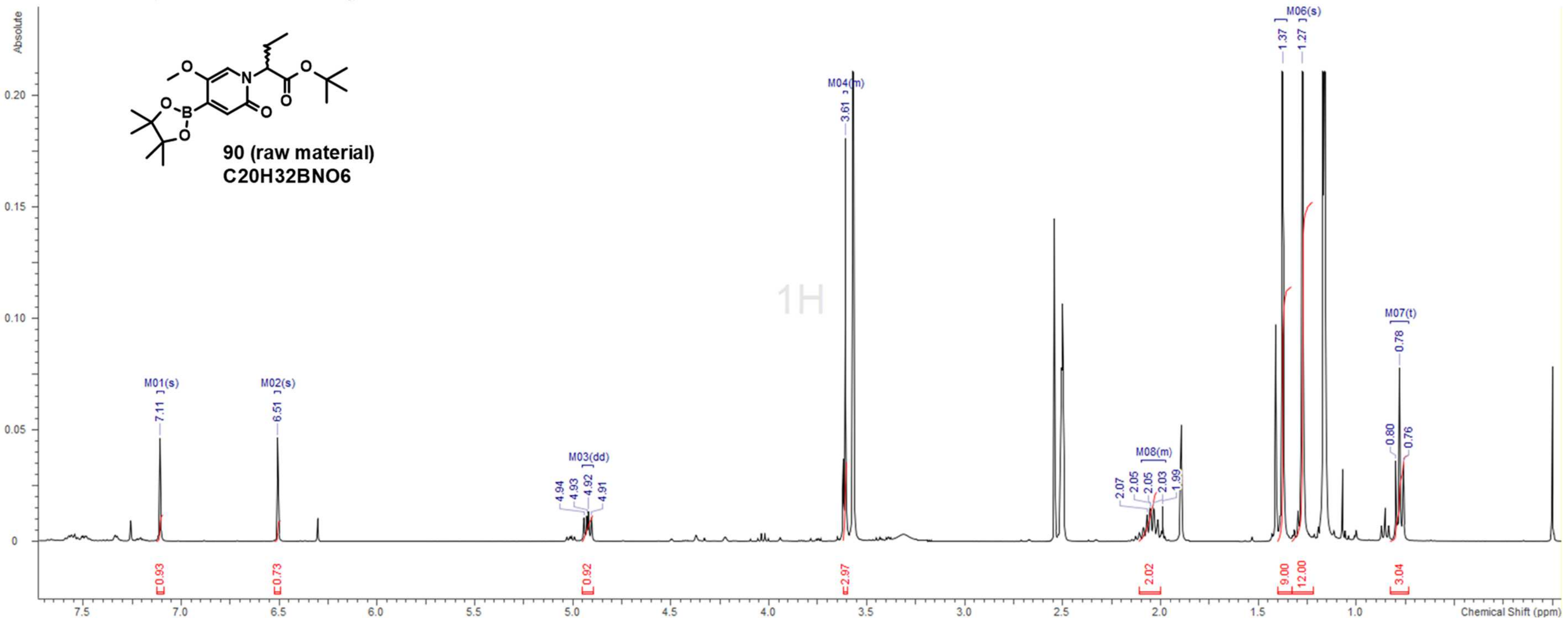

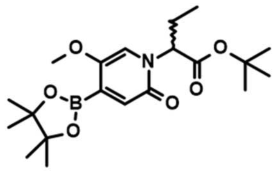

**90 (raw material)**
**MS-MW [g/mol]: 393.2323**

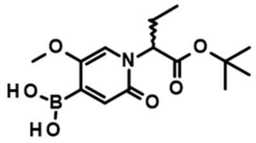

**MS-MW [g/mol]: 311.1540**

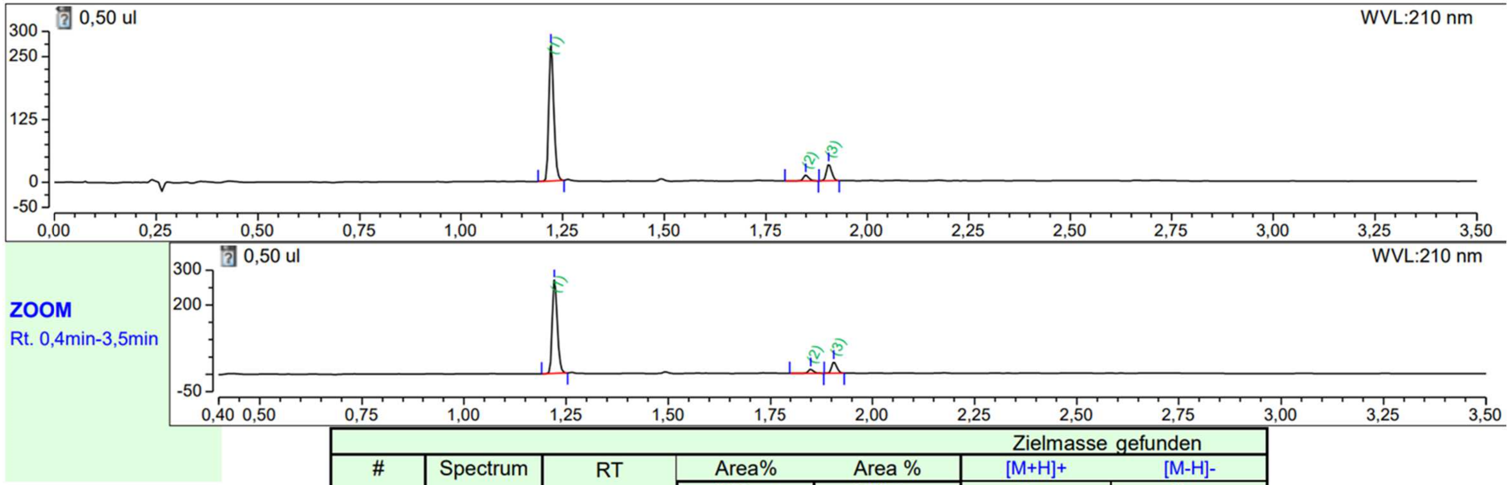


| # | Spectrum | RT min | Area% 210nm | Area % UVMax | Zielmasse gefunden [M+H]+ Pos | Zielmasse gefunden [M-H]- Neg |
|---|---|---|---|---|---|---|
| 1 | Y | 1,22 | 85,3 | 86,4 | | |
| 2 | Y | 1,85 | 3,9 | n.a. | | |
| 3 | Y | 1,91 | 10,8 | 13,6 | | |

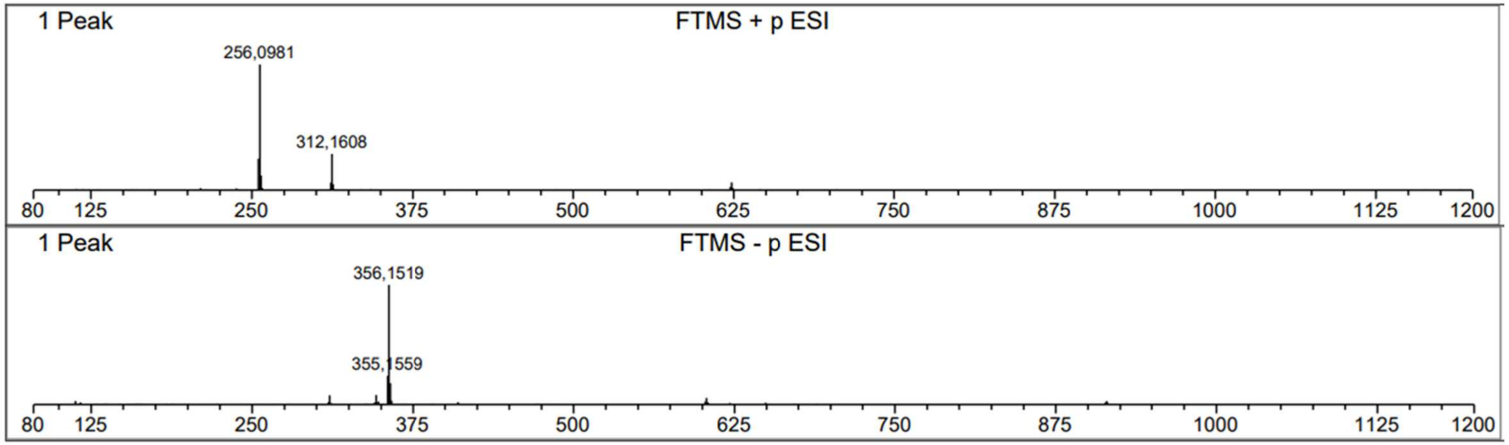

¹H-NMR (400 MHz, DMSO-$d_6$)

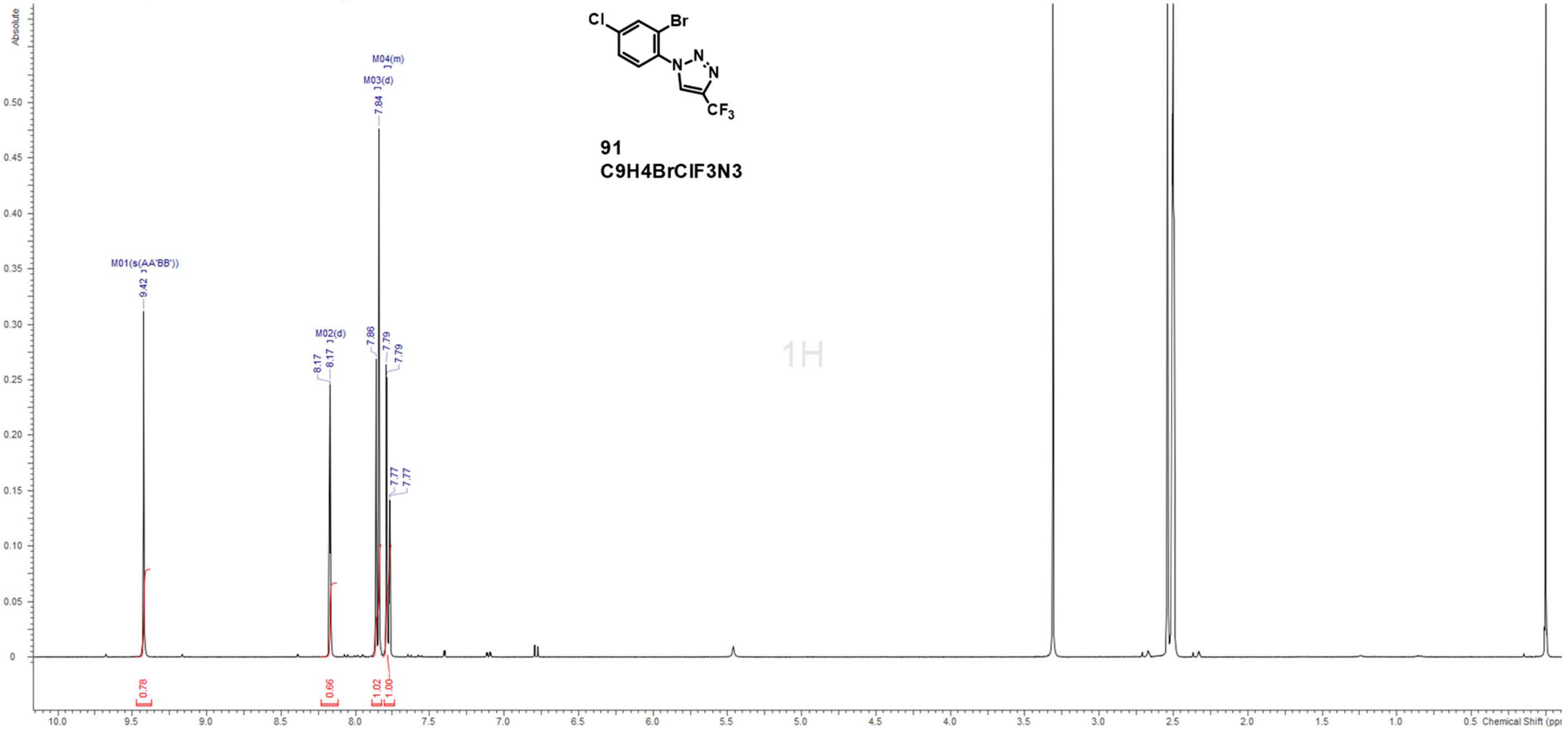

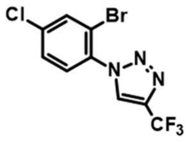

**91**
**MS-MW [g/mol]: 324.9229**

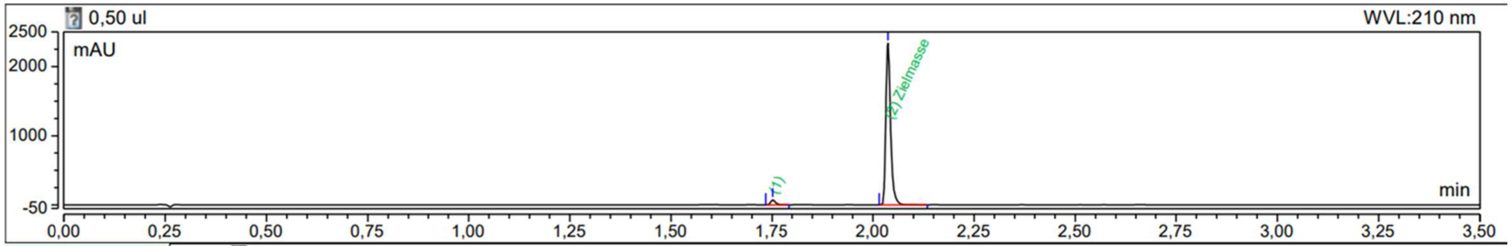


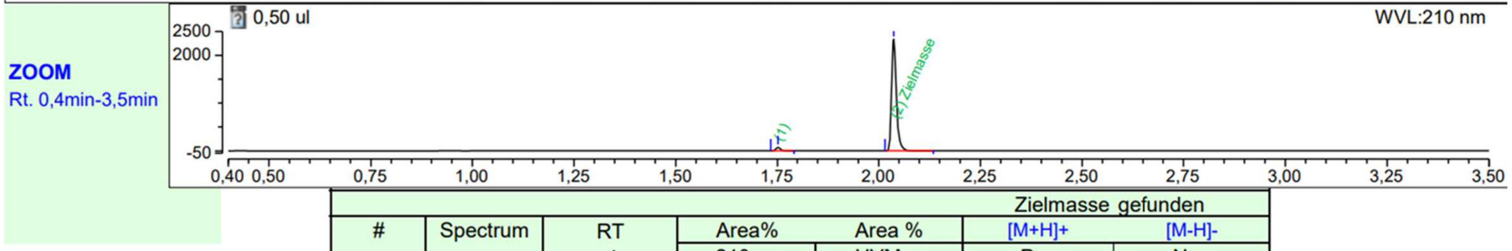


| # | Spectrum | RT | Area% | Area % | Zielmasse gefunden [M+H]+ | Zielmasse gefunden [M-H]- |
|---|---|---|---|---|---|---|
| | | min | 210nm | UVMax | Pos | Neg |
| 1 | Y | 1,75 | 3,0 | 1,3 | | |
| 2 | Y | 2,04 | 97,0 | 98,7 | 325,9302 | |

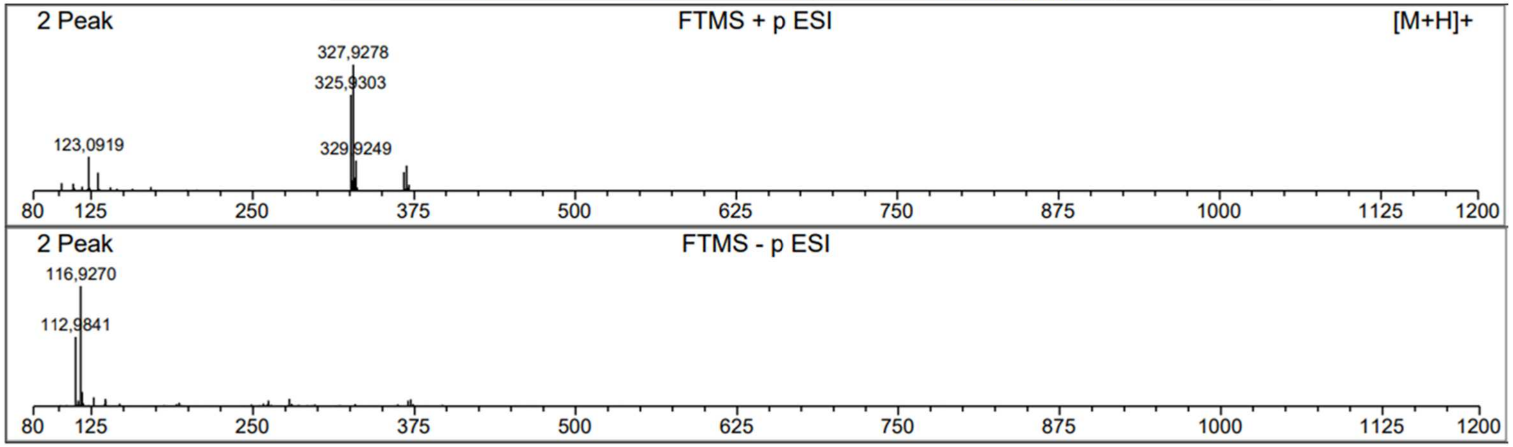

## $^1$H-NMR (400 MHz, DMSO-$d_6$)

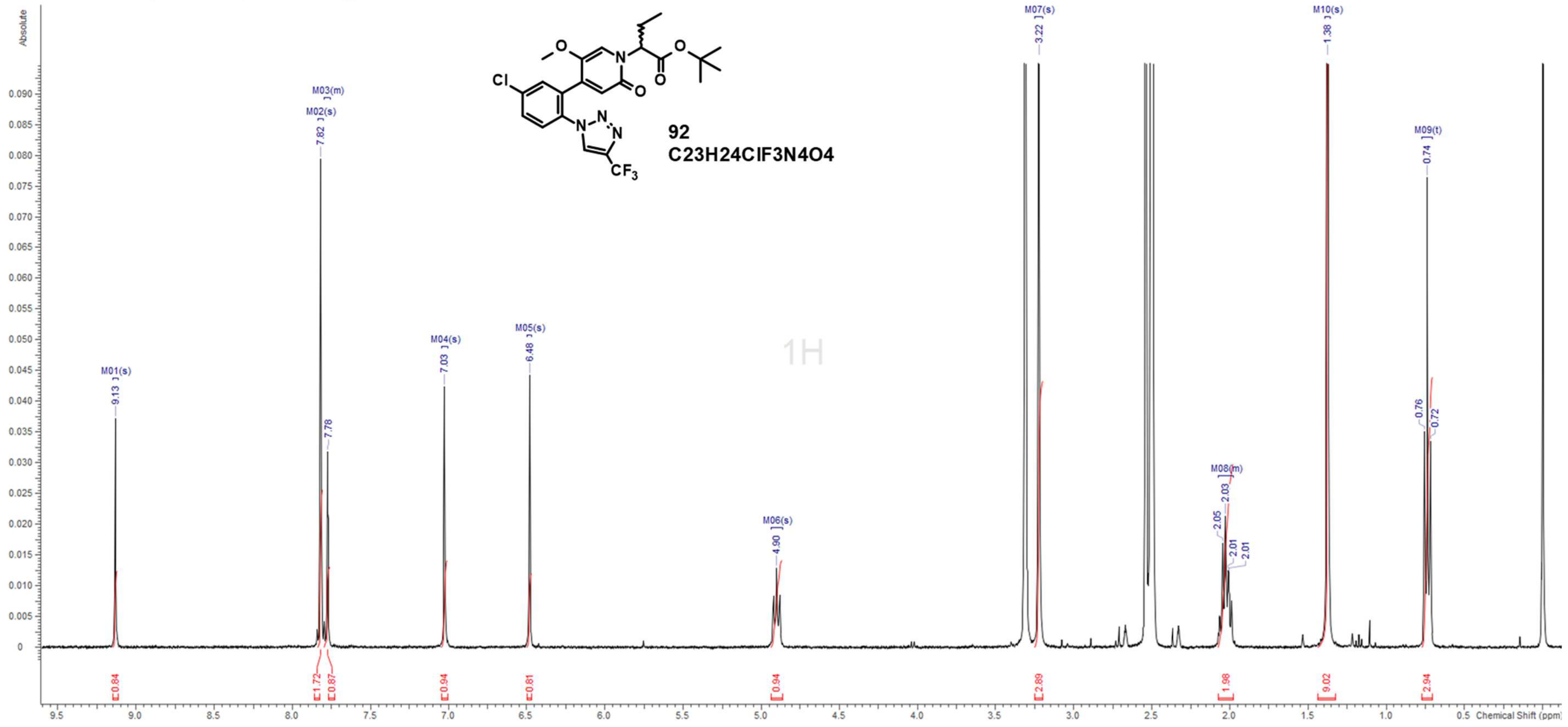

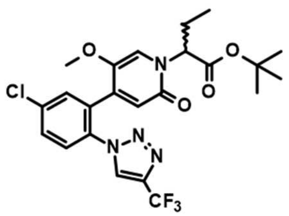


**92**
**MS-MW [g/mol]: 512.1438**

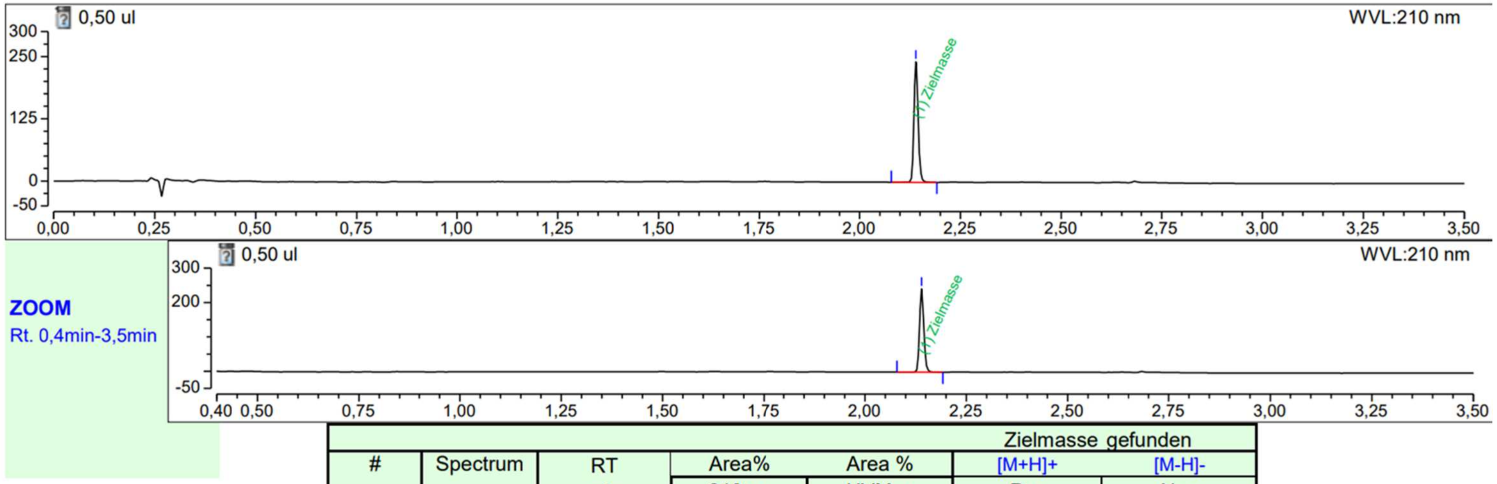


| # | Spectrum | RT min | Area% 210nm | Area % UVMax | Zielmasse gefunden [M+H]+ Pos | [M-H]- Neg |
|---|---|---|---|---|---|---|
| 1 | Y | 2,14 | 100,0 | 100,0 | 513,1511 | |

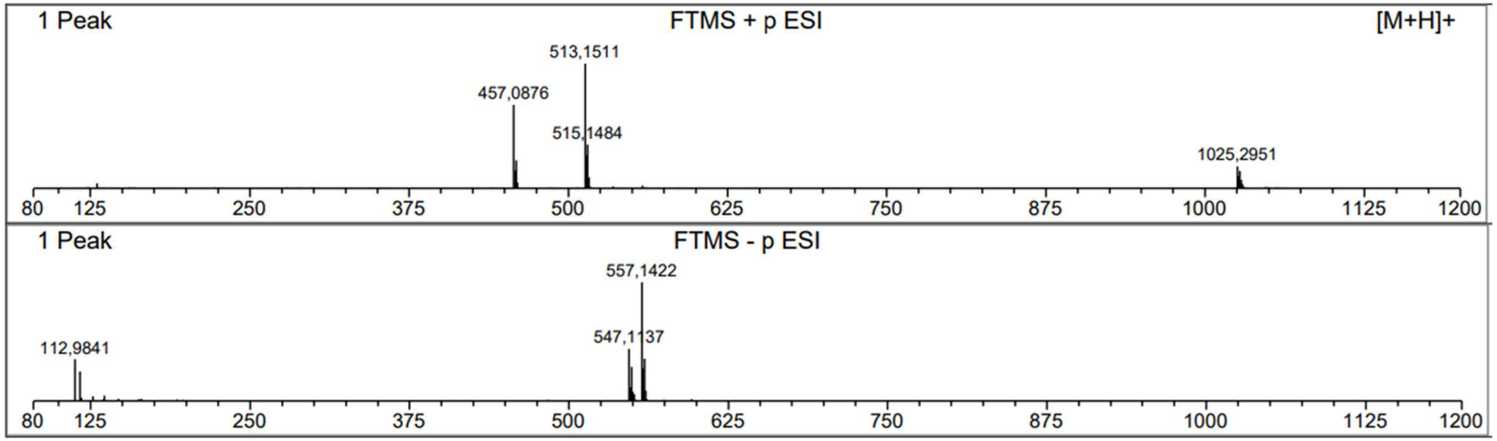

**$^{1}$H-NMR (400 MHz, DMSO-$d_6$)**

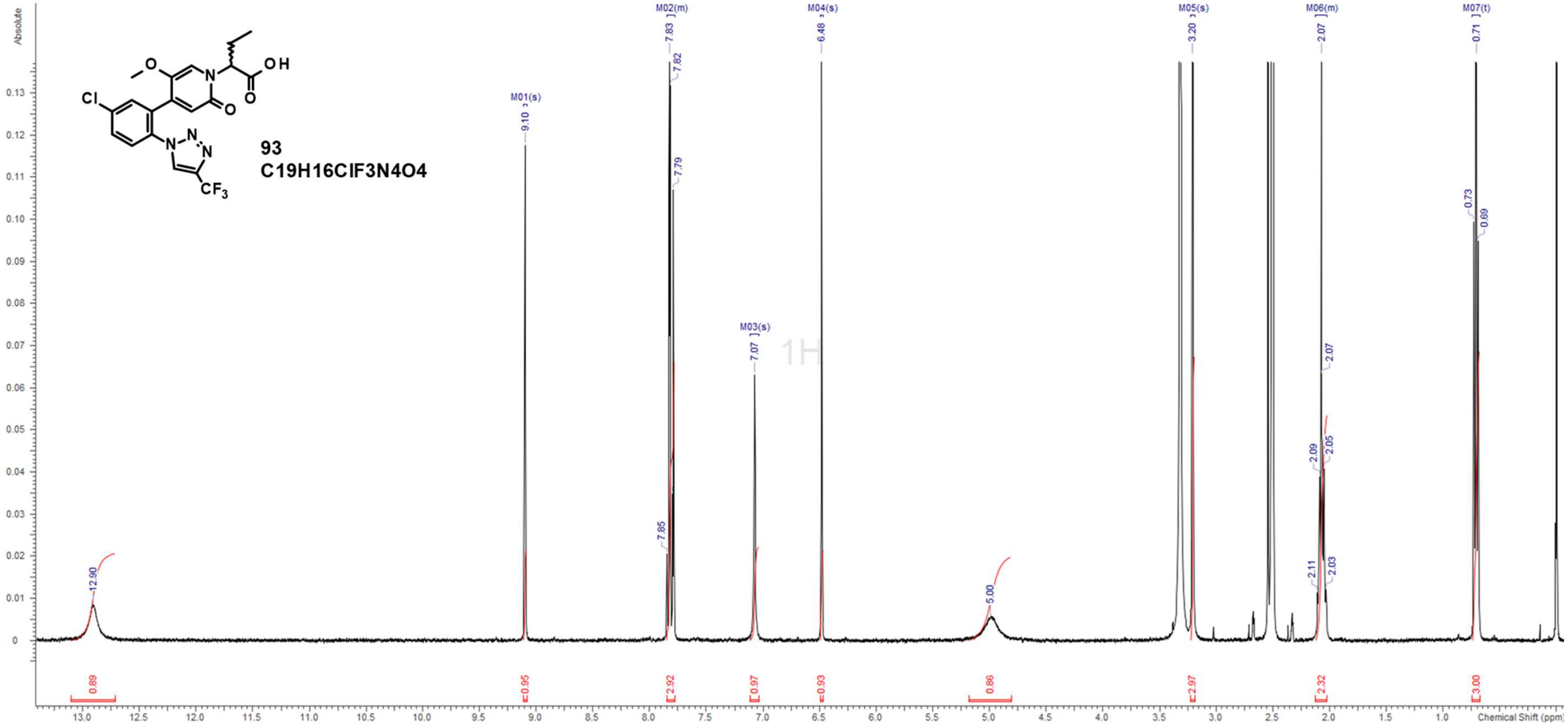

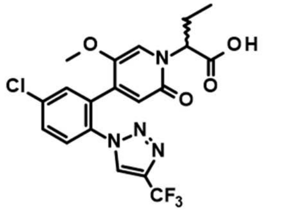

93
MS-MW [g/mol]: 456.0812

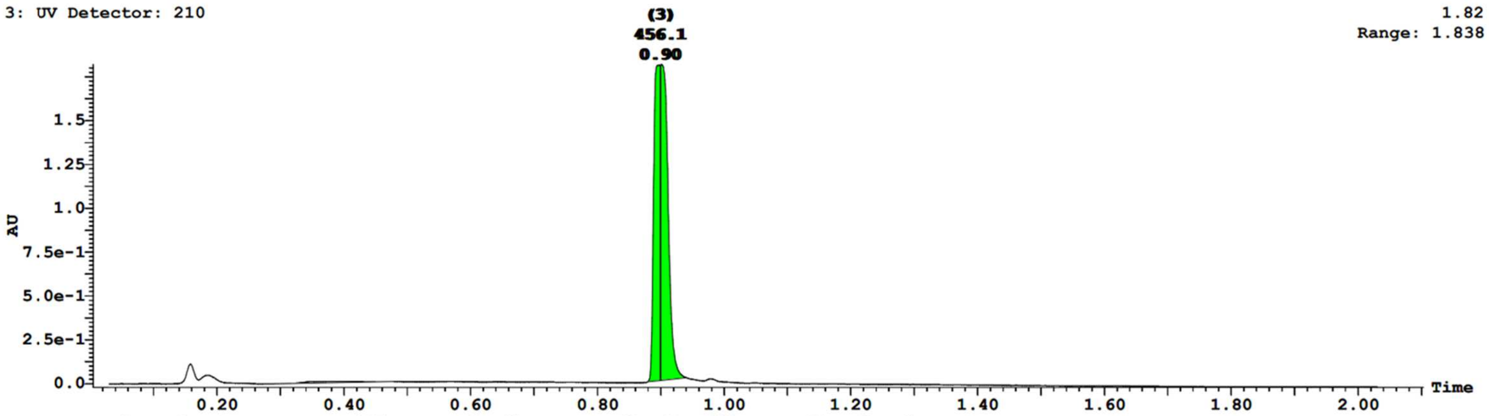


| # | Spectrum | RT | Area% | Pos Neg UV | Mass Found |
|---|---|---|---|---|---|
| 1 | --- | 0.40 | 1.80 | 114, 147, 211 | |
| 2 | --- | 0.90 | 44.46 | 457, 911, 250 | 456 |
| 3 | --- | 0.90 | 53.74 | 457, 911, 236 | 456 |

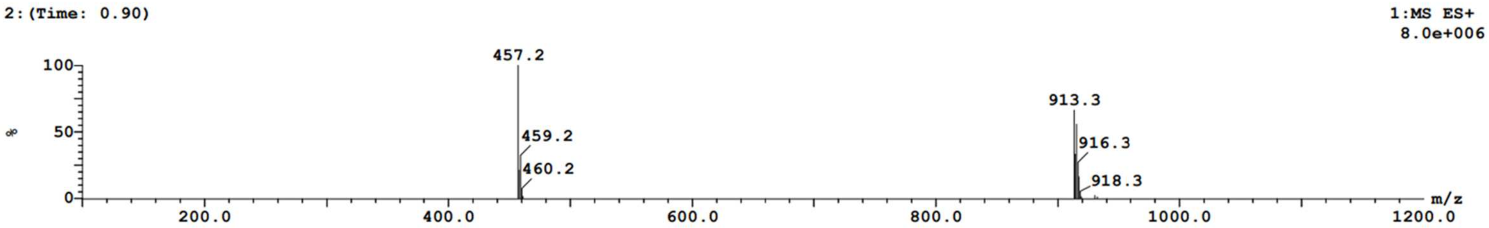


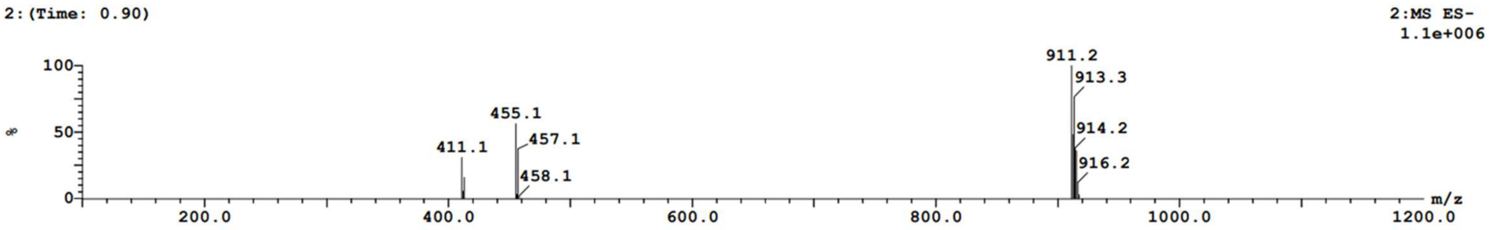

**$^1$H-NMR (400 MHz, DMSO-$d_6$)**

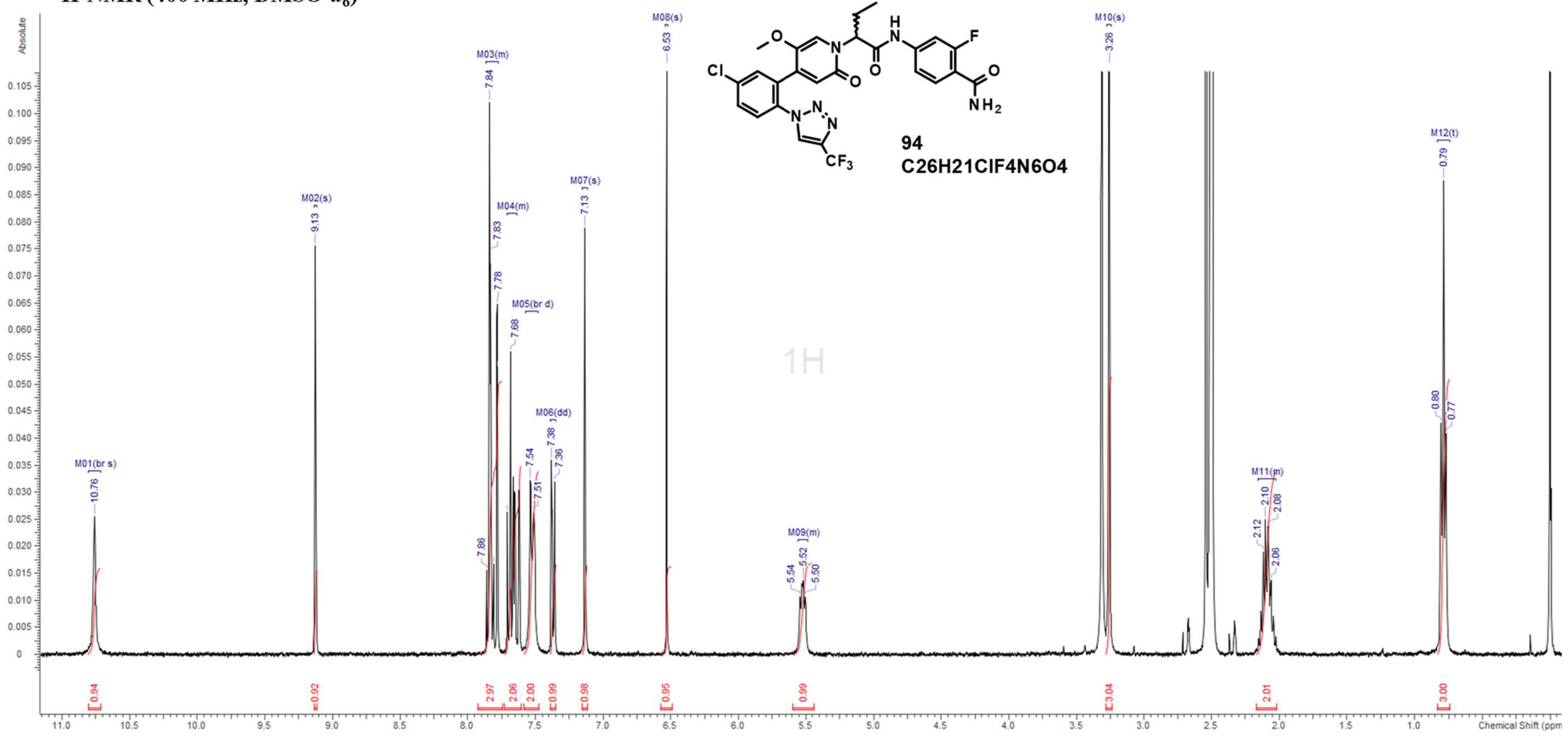

94
C26H21ClF4N6O4

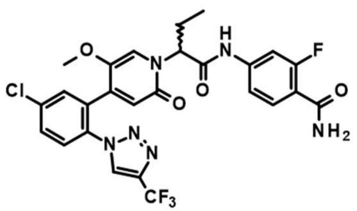

**94**
**MS-MW [g/mol]: 592.1249**

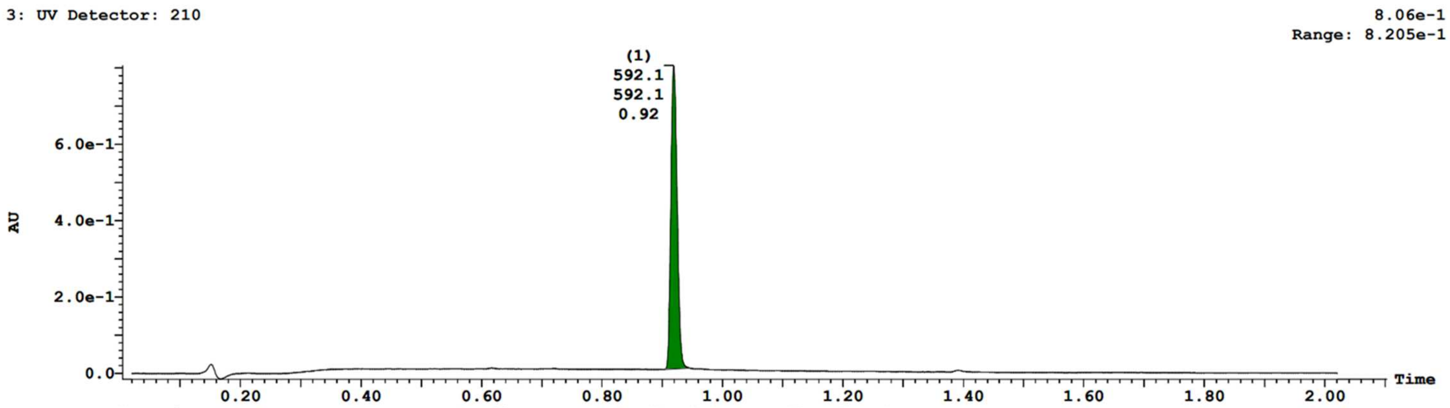


| # | Spectrum | RT | Area% | Pos Neg UV | Mass Found |
|---|---|---|---|---|---|
| 1 | --- | 0.92 | 100.00 | 593, 591, 208 | 592, 592 |

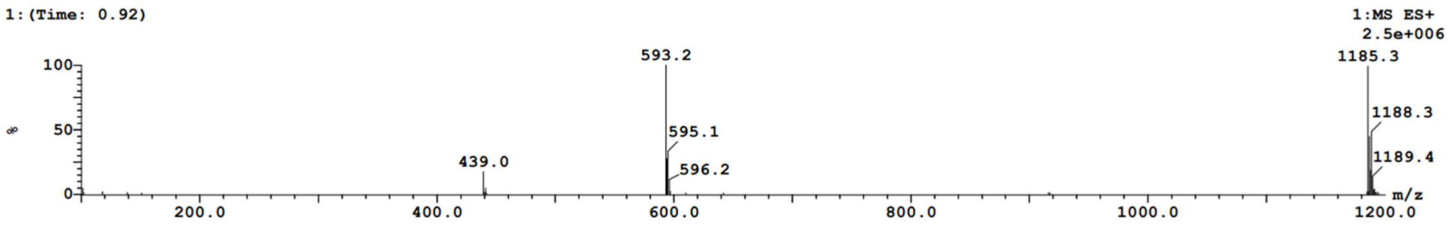


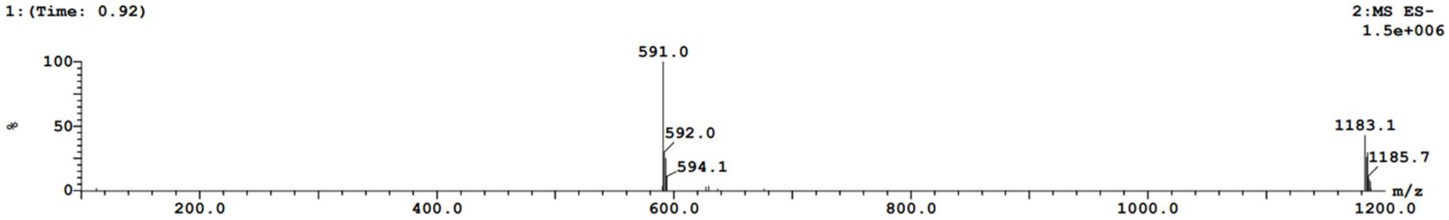

## $^1$H-NMR (500 MHz, DMSO-$d_6$)

**80**
**asundexian**
**(BAY 2433334)**
**C26H21ClF4N6O4**

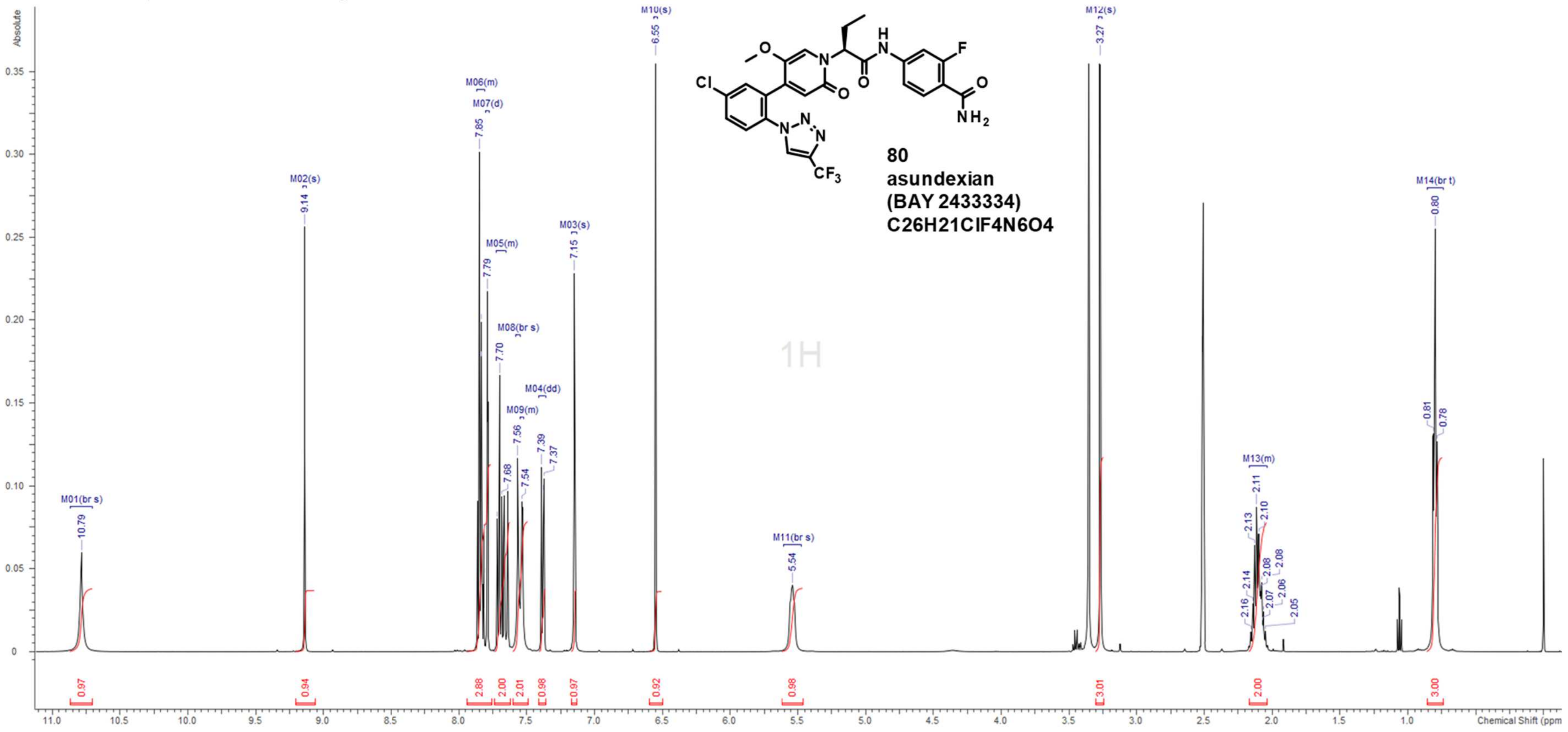

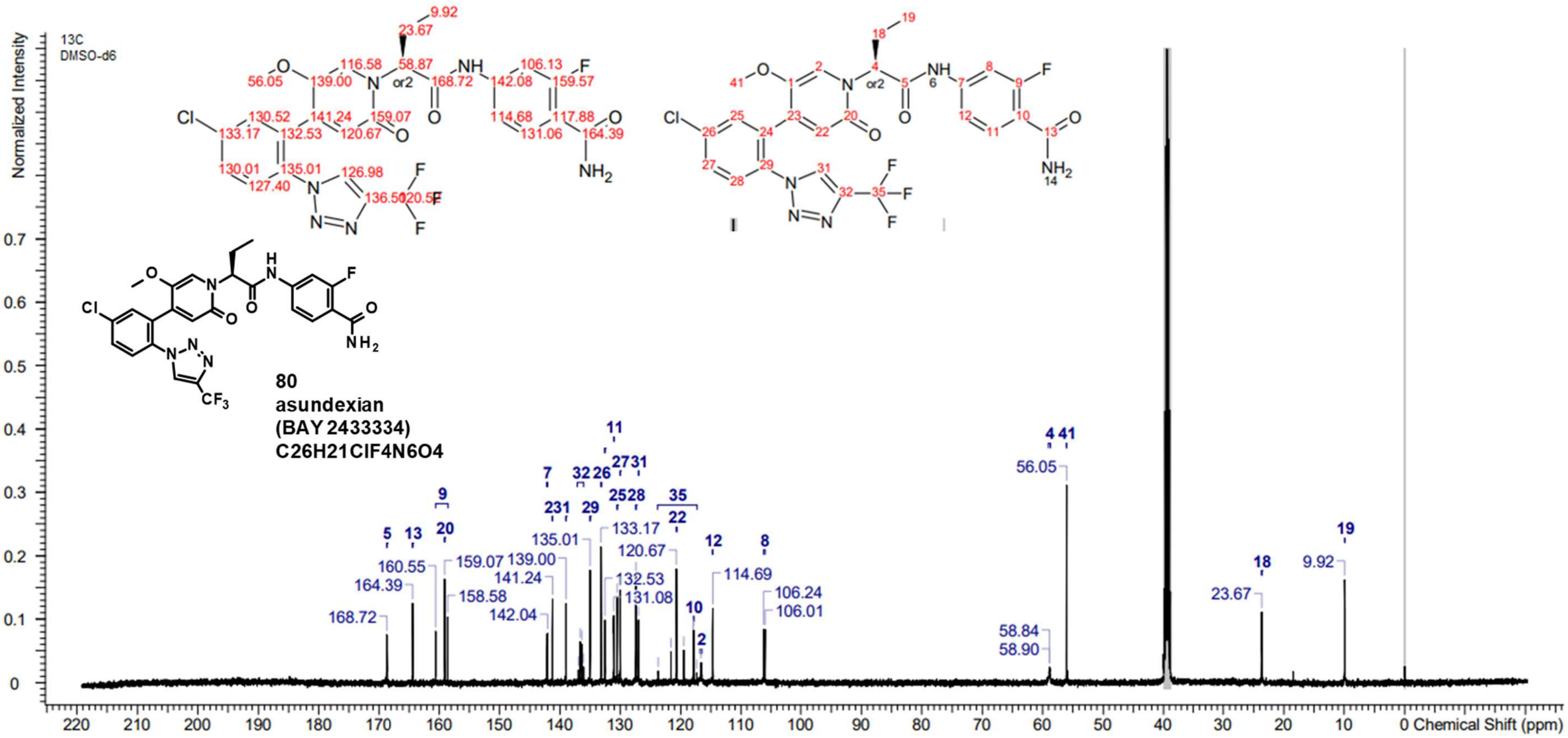
13C
DMSO-d6
80
asundexian
(BAY 2433334)
C26H21ClF4N6O4
Normalized Intensity
Chemical Shift (ppm)

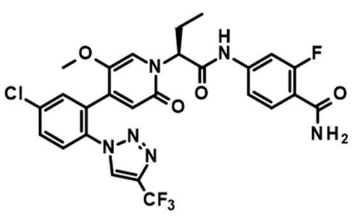


**80**
**asundexian (BAY 2433334)**
**MS-MW [g/mol]: 592.1249**

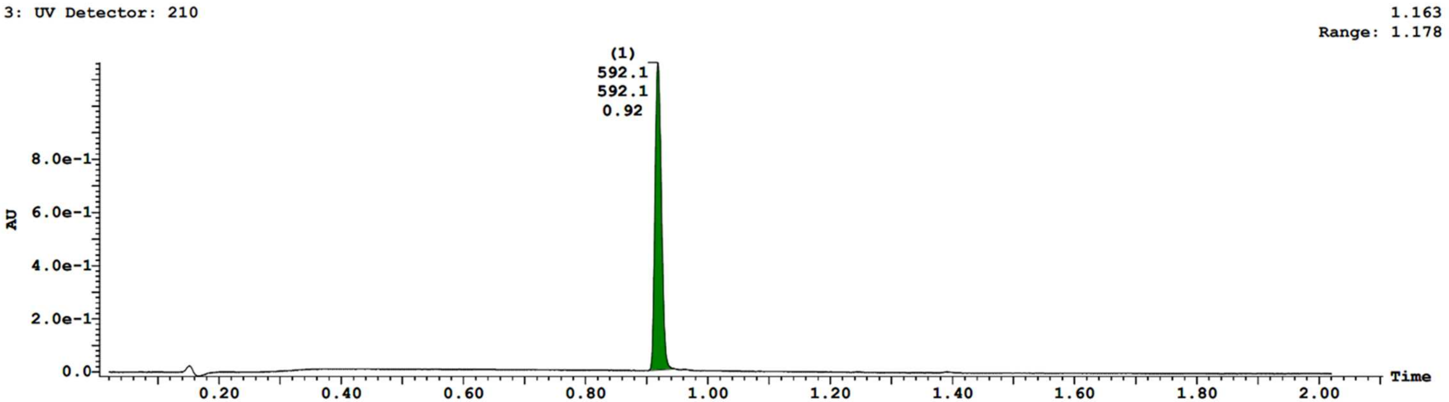


| # | Spectrum | RT | Area% | Pos Neg UV | Mass Found |
|---|---|---|---|---|---|
| 1 | --- | 0.92 | 100.00 | 593, 591, 208 | 592, 592 |

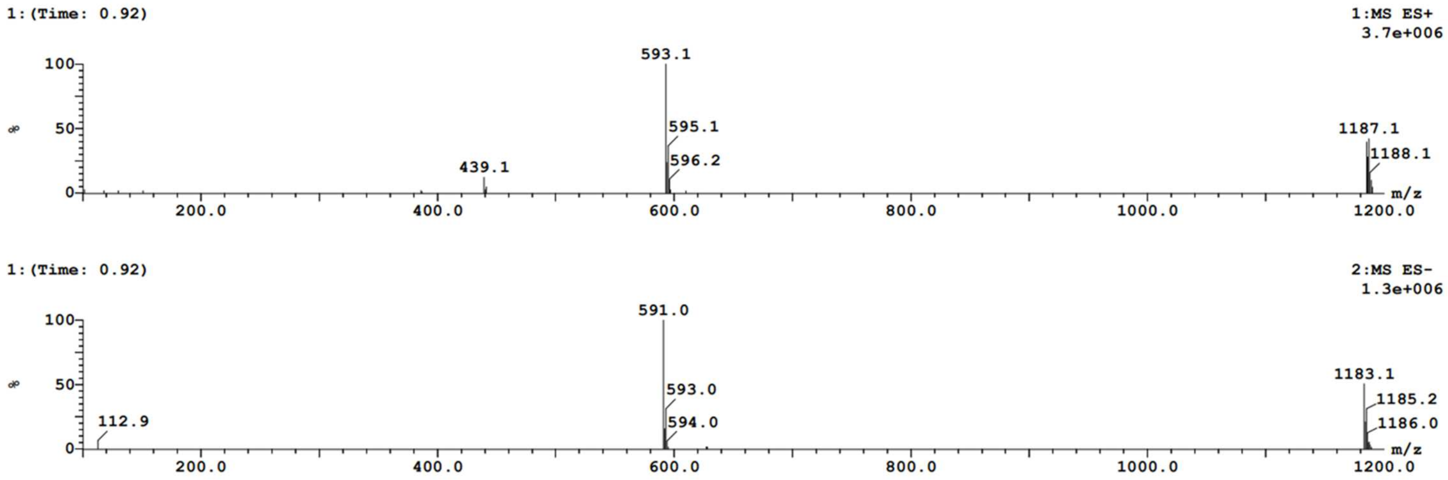

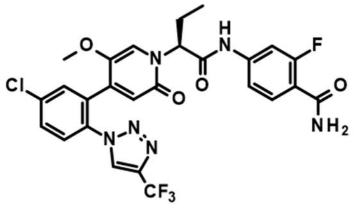

**80**
**asundexian (BAY 2433334)**
**MS-MW [g/mol]: 592.1249**

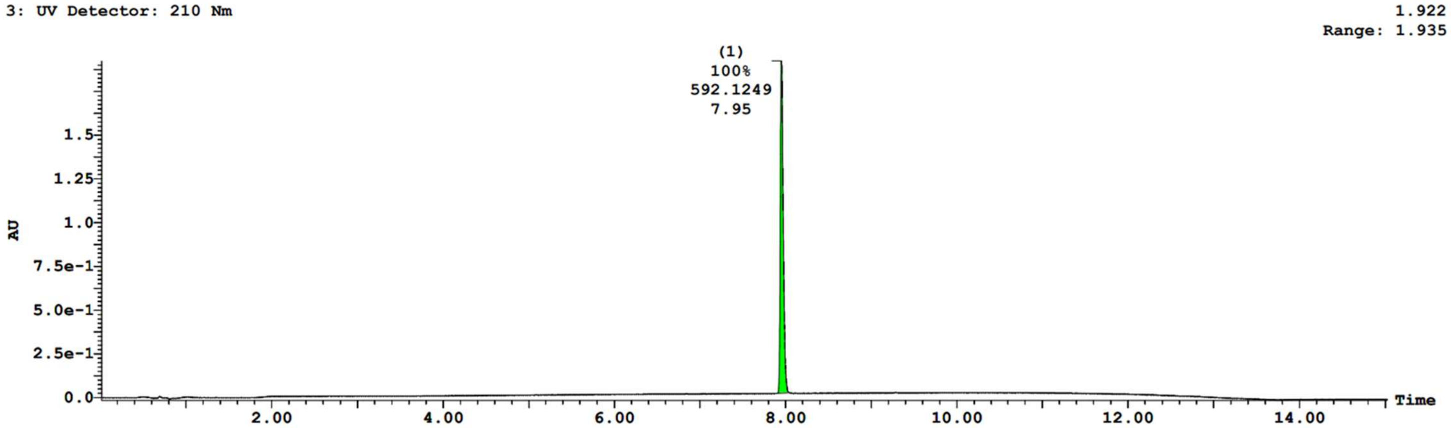


| # | Spectrum | RT | Area% | Pos UV | Mass Found |
|---|---|---|---|---|---|
| 1 | --- | 7.95 | 100.00 | 593, 211 | 592.1249 |

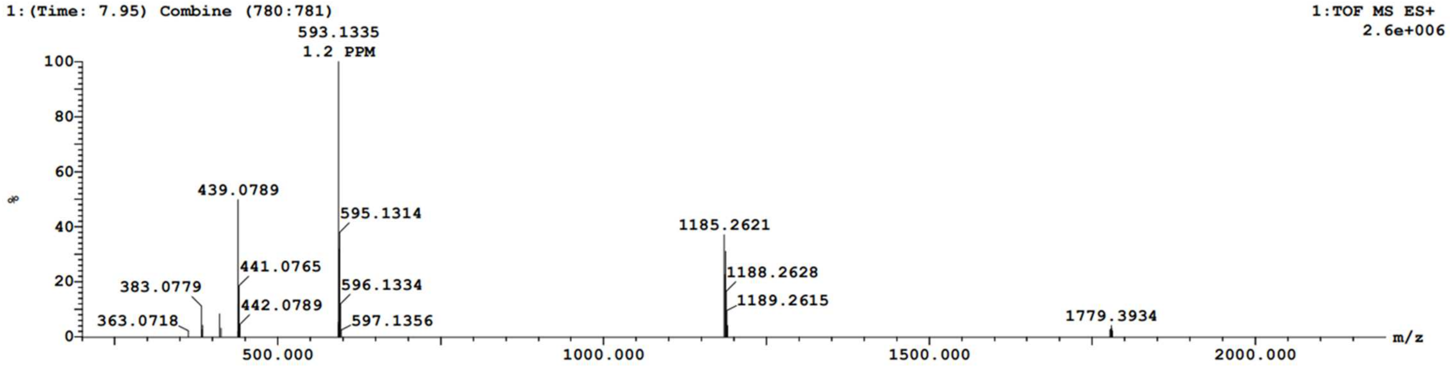

Enantiomer separation of racemate **94** resulted in:

**eutomer 80** (asundexian, BAY 2433334): chiral HPLC: $t_R$ = 1.97 min, >99% ee

**distomer**: chiral HPLC: $t_R$ = 1.26 min, >99% ee

Analytical method: SFC: column: Daicel AD-H 3 µm, 100 mm × 4.6 mm; mobile phase: 75% carbon dioxide / 25% ethanol; flow rate: 3 mL/min; UV detection: 210 nm.

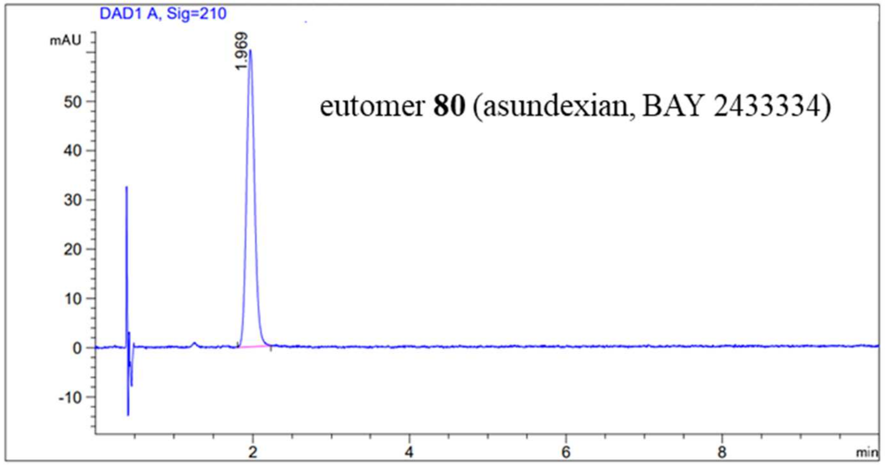


Report:

| # Peak | RetTime [Min] | Area [mAU*s] | Area % [%] | Height [mAU] | Height [%] | Width [Min] | Signal Desc. |
|---|---|---|---|---|---|---|---|
| 1 | 1.969 | 426.76 | 100.00 | 60.36 | 100.00 | 0.10 | DAD1 A, Sig=210 |

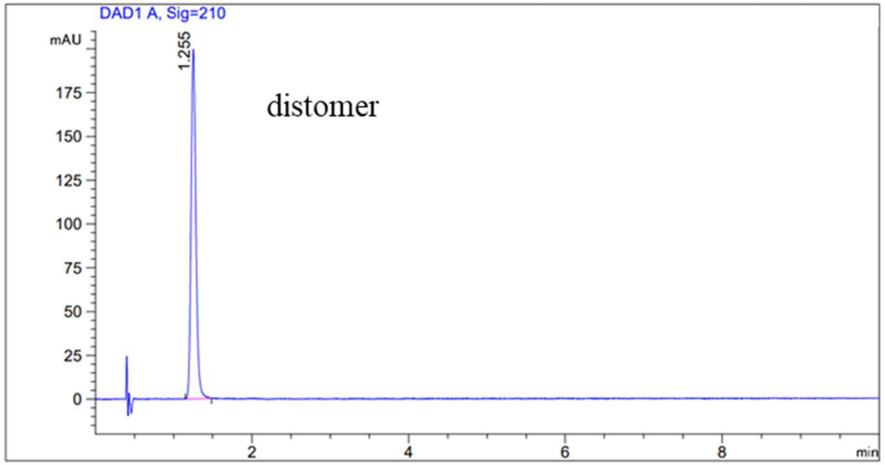


Report:

| # Peak | RetTime [Min] | Area [mAU*s] | Area % [%] | Height [mAU] | Height [%] | Width [Min] | Signal Desc. |
|---|---|---|---|---|---|---|---|
| 1 | 1.255 | 852.34 | 100.00 | 199.58 | 100.00 | 0.07 | DAD1 A, Sig=210 |

## (C) Synthesis of Compounds 3-79, 81-85, 355, and 362.

**Chemistry. General Procedures.** All commercial reagents and catalysts were used as provided by the commercial supplier without purification. Solvents for synthesis, extraction, and chromatography were of reagent grade and used as received. Moisture-sensitive reactions were carried out under an atmosphere of argon, and anhydrous solvents were used as provided by the commercial supplier. Preparative normal-phase flash chromatography was performed using Biotage Isolera chromatography systems with Biotage silica cartridges or silica gel 60 (230–400 mesh) in combination with glass columns/frits. Preparative reversed-phase (RP) chromatography was performed on 125/250 mm × 20/30/40 mm HPLC columns packed with YMC gel ODS-AQ S-5/15 $\mu$m, and UV detection. Gradients or isocratic mixtures used as eluents are indicated. All compounds tested in biological assays were of ≥95% purity, as determined by HPLC, LC/MS, or NMR data.

$^{1}$H NMR and $^{13}$C NMR spectra were recorded at RT with Bruker Avance spectrometers. Chemical shifts ($\delta$) are reported in ppm relative to TMS as an internal standard. The descriptions of the coupling patterns of $^{1}$H NMR signals are based on the optical appearance of the signals and do not necessarily reflect the physically correct interpretation. In general, the chemical shift information refers to the center of the signal. In the case of multiplets, intervals are given.

Analytical mass spectrometry was performed on HPLC/MS (Waters, Agilent, Thermo Fisher) or GC/MS (Waters, Agilent, Thermo Fisher) systems using Waters Time-of-Flight, Waters/Micromass Single Quadrupole, or Thermo Fisher Scientific Orbitrap mass spectrometers. Ionization methods were electrospray ionization (ESI) positive/negative or electron ionization (EI). LC/MS and GC/MS analyses were performed using the respective method 1−22, as noted.

<u>Method 1</u>: Instrument: Waters Acquity SQD UPLC system; column: Waters Acquity UPLC HSS T3 C18 1.8 $\mu$m, 50 mm × 1.0 mm; eluent A: water + 0.025% formic acid, eluent B: acetonitrile + 0.025% formic acid; gradient: 0.0 min 10% B → 1.2 min 95% B → 2.0 min 95% B; temperature: 50 °C; flow rate: 0.40 mL/min; UV detection: 210-400 nm.

<u>Method 2</u>: Instrument: Micromass Quattro Premier MS with Waters Acquity UPLC; column: Thermo Hypersil GOLD 1.9 $\mu$m, 50 mm × 1 mm; eluent A: 1 L water + 0.5 mL 50% formic acid, eluent B: 1 L acetonitrile + 0.5 mL 50% formic acid; gradient: 0.0 min 97% A → 0.5 min 97% A → 3.2 min 5% A → 4.0 min 5% A; temperature: 50 °C; flow rate: 0.3 mL/min; UV detection: 210 nm.

<u>Method 3</u>: Instrument: Thermo Scientific FT-MS with Thermo Scientific UltiMate 3000 UHPLC; column: Waters HSS T3 C18 1.8 $\mu$m, 75 mm × 2.1 mm; eluent A: water + 0.01% formic acid; eluent B: acetonitrile + 0.01% formic acid; gradient: 0.0 min 10% B → 2.5 min 95% B → 3.5 min 95% B; temperature: 50 °C; flow rate: 0.90 mL/min; UV detection: 210-400 nm.

Method 4: Instrument: Waters Acquity SQD UPLC System; column: Waters Acquity UPLC HSS T3 1.8 $\mu$m, 50 mm × 1 mm; eluent A: 1 L water + 0.25 mL 99% formic acid, eluent B: 1 L acetonitrile + 0.25 mL 99% formic acid; gradient: 0.0 min 90% A → 1.2 min 5% A → 2.0 min 5% A; temperature: 50 °C; flow rate: 0.40 mL/min; UV detection: 210-400 nm.

Method 5: Instrument: Agilent MS Quad 6150; HPLC: Agilent 1290; column: Waters Acquity UPLC HSS T3 1.8 $\mu$m, 50 mm × 2.1 mm; eluent A: 1 L water + 0.25 mL of 99% formic acid, eluent B: 1 L acetonitrile + 0.25 mL of 99% formic acid; gradient: 0.0 min 90% A → 0.3 min 90% A → 1.7 min 5% A → 3.0 min 5% A; temperature: 50 °C; flow rate: 1.20 mL/min; UV detection: 205–305 nm.

Method 6: Instrument: Micromass Quattro Premier with Waters UPLC Acquity; column: Thermo Hypersil GOLD 1.9 $\mu$m, 50 mm × 1 mm; eluent A: 1 L water + 0.5 mL 50% formic acid, eluent B: 1 L acetonitrile + 0.5 mL 50% formic acid; gradient: 0.0 min 90% A → 0.1 min 90% A → 1.5 min 10% A → 2.2 min 10% A; temperature: 50 °C; flow rate: 0.33 mL/min; UV detection: 210 nm.

Method 7: Instrument: Waters Acquity SQD UPLC System; column: Waters Acquity UPLC HSS T3 1.8 $\mu$m, 30 mm × 2 mm; eluent A: 1 L water + 0.25 mL 99% formic acid, eluent B: 1 L acetonitrile + 0.25 mL 99% formic acid; gradient: 0.0 min 90% A → 1.2 min 5% A → 2.0 min 5% A; temperature: 50 °C; flow rate: 0.60 mL/min; UV detection: 208-400 nm.

Method 8: MS instrument: Waters (Micromass) Quattro Micro; instrument Waters UPLC Acquity; column: Waters BEH C18 1.7 $\mu$m, 50 mm × 2.1 mm; eluent A: 1 L water + 0.01 mol of ammonium formiate, eluent B: 1 L acetonitrile; gradient: 0.0 min 95% A → 0.1 min 95% A → 2.0 min 15% A → 2.5 min 15% A → 2.51 min 10% A → 3.0 min 10% A; temperature: 40 °C; flow rate: 0.5 mL/min; UV detection: 210 nm.

Method 9: Instrument: Thermo Scientific DSQII, Thermo Scientific Trace GC Ultra; column: Restek RTX-35MS, 15 m × 200 $\mu$m × 0.33 $\mu$m; constant helium flow rate: 1.20 mL/min; temperature: 60 °C; inlet: 220 °C; gradient: 60 °C, 30 °C/min → 300 °C (maintained for 3.33 min).

Method 10: Instrument: Thermo DFS, Trace GC Ultra; column: Restek RTX-35, 15 m × 200 $\mu$m × 0.33 $\mu$m; constant helium flow rate: 1.20 mL/min; temperature: 60 °C; inlet: 220 °C; gradient: 60 °C, 30 °C/min → 300 °C (maintained for 3.33 min).

Method 11: Instrument: SHIMADZU LCMS: UFLC 20-AD and LCMS 2020 MS detector (electrospray ion source (ESI): scan between *m*/*z* 90-900 using a scan time of 0.5-1.0 s); column: Ascentis Express C18 2.7 $\mu$m, 2.1 mm × 50 mm; linear gradient: 95% A (A: 0.05% trifluoroacetic acid in water) to 100% B (B: 0.05% trifluoroacetic acid in acetonitrile) over 1.0 min with a total run time of 2.0 min; temperature: 40 °C; flow rate: 1.0 mL/min; UV detection: 190-400 nm.

Method 12: Instrument: SHIMADZU LCMS: UFLC 20-AD and LCMS 2020 MS detector (electrospray ion source (ESI): scan between *m*/*z* 90-900 using a scan time of 0.5-1.0 s); column: Ascentis Express

C18 2.7 μm, 2.1 mm × 50 mm; linear gradient: 95% A (A: 0.05% trifluoroacetic acid in water) to 100% B (B: 0.05% trifluoroacetic acid in acetonitrile) over 2.1 min with a total run time of 3.0 min; temperature: 40 °C; flow rate: 1.0 mL/min; UV detection: 190-400 nm.

Method 13: Instrument: SHIMADZU LCMS: UFLC 20-AD and LCMS 2020 MS detector (electrospray ion source (ESI): scan between *m/z* 90-900 using a scan time of 0.5-1.0 s); column: Ascentis Express C18 2.7 μm, 2.1 mm × 50 mm; linear gradient: 95% A (A: 0.05% trifluoroacetic acid in water) to 95% B (B: 0.05% trifluoroacetic acid in acetonitrile) over 2.0 min with a total run time of 3.0 min; temperature: 40 °C; flow rate: 1.0 mL/min; UV detection: 190-400 nm.

Method 14: Instrument: SHIMADZU LCMS: UFLC 20-AD and LCMS 2020 MS detector (electrospray ion source (ESI): scan between *m/z* 90-900 using a scan time of 0.5-1.0 s); column: CORTECS C18 2.7 μm, 2.1 mm × 50 mm; linear gradient: 95% A (A: 0.09% formic acid in water) to 100% B (B: 0.1% formic acid in acetonitrile) over 1.2 min with a total run time of 2.0 min; temperature: 40 °C; flow rate: 1.0 mL/min; UV detection: 190-400 nm.

Method 15: Instrument: SHIMADZU LCMS: UFLC 20-AD and LCMS 2020 MS detector (electrospray ion source (ESI): scan between *m/z* 90-900 using a scan time of 0.5-1.0 s); column: CORTECS C18 2.7 μm, 2.1 mm × 50 mm; linear gradient: 95% A (A: 0.09% formic acid in water) to 95% B (B: 0.1% formic acid in acetonitrile) over 2.0 min with a total run time of 3.0 min; temperature: 40 °C; flow rate: 1.0 mL/min; UV detection: 190-400 nm.

Method 16: Instrument: SHIMADZU LCMS: UFLC 20-AD and LCMS 2020 MS detector (electrospray ion source (ESI): scan between *m/z* 90-900 using a scan time of 0.5-1.0 s); column: Ascentis C18 2.7 μm, 2.1 mm × 50 mm; linear gradient: 95% A (A: 0.05% trifluoroacetic acid in water) to 100% B (B: 0.05% trifluoroacetic acid in acetonitrile) over 1.1 min with a total run time of 2.0 min; temperature: 45 °C; flow rate: 1.0 mL/min; UV detection: 190-400 nm.

Method 17: Instrument: SHIMADZU LCMS: UFLC 20-AD and LCMS 2020 MS detector (electrospray ion source (ESI): scan between *m/z* 90-900 using a scan time of 0.5-1.0 s); column: Ascentis C18 2.7 μm, 2.1 mm × 50 mm; linear gradient: 95% A (A: 0.05% trifluoroacetic acid in water) to 100% B (B: 0.05% trifluoroacetic acid in acetonitrile) over 1.2 min with a total run time of 2.0 min; temperature: 40 °C; flow rate: 1.0 mL/min; UV detection: 190-400 nm.

Method 18: Instrument: SHIMADZU LCMS: UFLC 20-AD and LCMS 2020 MS detector (electrospray ion source (ESI): scan between *m/z* 90-900 using a scan time of 0.5-1.0 s); column: Ascentis Express C18 2.7 μm, 2.1 × 50 mm; linear gradient: 95% A (A: 0.05% trifluoroacetic acid in water) and to 100% B (B: 0.05% trifluoroacetic acid in acetonitrile) over 1.2 min with a total run time of 2.0 min; temperature: 40 °C; flow rate: 1.0 mL/min.

Method 19: Instrument: SHIMADZU LCMS: UFLC 20-AD and LCMS 2020 MS detector (electrospray ion source (ESI): scan between *m/z* 90-900 using a scan time of 0.7 s); column: Shim-pack XR-ODS,

2.2 $\mu$m, 3.0 mm × 50 mm; linear gradient: 95% A (A: 0.05% trifluoroacetic acid in water) to 100% B (B: 0.05% trifluoroacetic acid in acetonitrile) over 2.2 min with a total run time of 3.6 min; column temperature: 40 °C; flow rate: 1.0 mL/min; UV detection: 190-400 nm.

Method 20: Instrument: Waters Acquity SQD UPLC System; column: Waters Acquity UPLC HSS T3 1.8 $\mu$m, 50 mm × 1 mm; eluent A: 1 L water + 0.25 mL 99%ige formic acid, eluent B: 1 L acetonitrile + 0.25 mL 99%ige formic acid; gradient: 0.0 min 95% A → 6.0 min 5% A → 7.5 min 5% A; temperature: 50 °C; flow rate: 0.35 mL/min; UV-Detection: 210 nm.

Method 21: Instrument: Waters TOF MS with Waters Acquity I-Class UPLC; column: Waters Acquity UPLC HSS T3, 1.8 $\mu$m, 150 mm × 2.1 mm; eluent A: 1 L water + 0.100 mL 99% trifluoroacetic acid, eluent B: 1 L acetonitrile + 0.100 mL 99% trifluoroacetic acid; gradient: 0.0 min 5% B → 1 min 5% B → 13 min 95% B → 15 min 95% B; temperature: 50 °C; flow rate: 0.60 mL/min; UV-Detection: 210 nm.

Method 22: Instrument: Waters TOF MS with Waters Acquity I-Class; column: Waters Acquity UPLC HSS T3 1.8 $\mu$m, 50 mm × 1 mm; eluent A: 1 L water + 0.100 mL 99%ige formic acid, eluent B: 1 L acetonitrile + 0.100 mL 99%ige formic acid; gradient: 0.0 min 90% A → 1.2 min 5% A → 2.0 min 5% A; temperature: 50 °C; flow rate: 0.40 mL/min; UV-Detection: 210 nm.

Assessment of optical rotation [$\alpha$] was performed using an Anton Paar polarimeter MCP2000 with parameters (wavelength, temperature, optical pathway, solvent, and concentration) as indicated.

**Abbreviations.** $[\alpha]_D^{20}$, specific angle of rotation (in polarometry); **ACN**, acetonitrile; **BPin**, boronic acid pinacol; **CAN**, ammonium cerium(IV) nitrate; **d**, doublet (in NMR); **DAST**, Diethylaminosulfur trifluoride; **DCM**, dichloromethane; **dd**, doublet of doublets (in NMR); **DIAD**, diisopropyl azodicarboxylate; **DIC**, *N,N'*-diisopropylcarbodiimde; **DIEA**, *N,N*-diisopropylethylamine; **DMF**, *N,N*-dimethylformamide; **DMSO**, dimethyl sulfoxide; **dppf**, 1,1′-bis(diphenylphosphino)ferrocene; **ee**, enantiomeric excess; **EI**, electron ionization; **ESI**, electrospray ionization; **GC**, gas chromatography; **HATU**, O-(7-azabenzotriazol-1-yl)-*N,N,N',N'*-tetramethyluronium-hexafluorphosphat; **HMDS**, hexamethyldisilazane; **HPLC**, high-performance liquid chromatography; **HRMS**, high-resolution mass spectrometry; **IC**, ion chromatography; **LDA**, lithium diisopropylamide; **m**, multiplet (in NMR); **MS**, mass spectrometry; **MTBE**, *tert*-butyl methyl ether; **MW**, molecular weight; **NCS**, *N*-chlorosuccinimide; **NMR**, nuclear magnetic resonance (in spectrometry); **ppm**, parts per million (in NMR); **q**, quartet (in NMR); **RF**, reflux; **RP**, reversed phase; **RT**, room temperature; **s**, singlet (in NMR); **SEM-Cl**, [2-(chloromethoxy)ethyl](trimethyl)silane; **SFC**, supercritical fluid chromatography; **t**, triplett (in NMR); **T3P**, propanephosphonic acid anhydride; **$Tf_2O$**, trifluoromethanesulfonic acid anhydride; **TFA**, trifluoroacetic acid; **THF**, tetrahydrofuran; **TMEDA**, *N,N,N'*-trimethyl

ethylenediamine; **TMS**, tetramethylsilane; **wt %**, percentage by weight.

**Synthesis of compound 3.**[a]

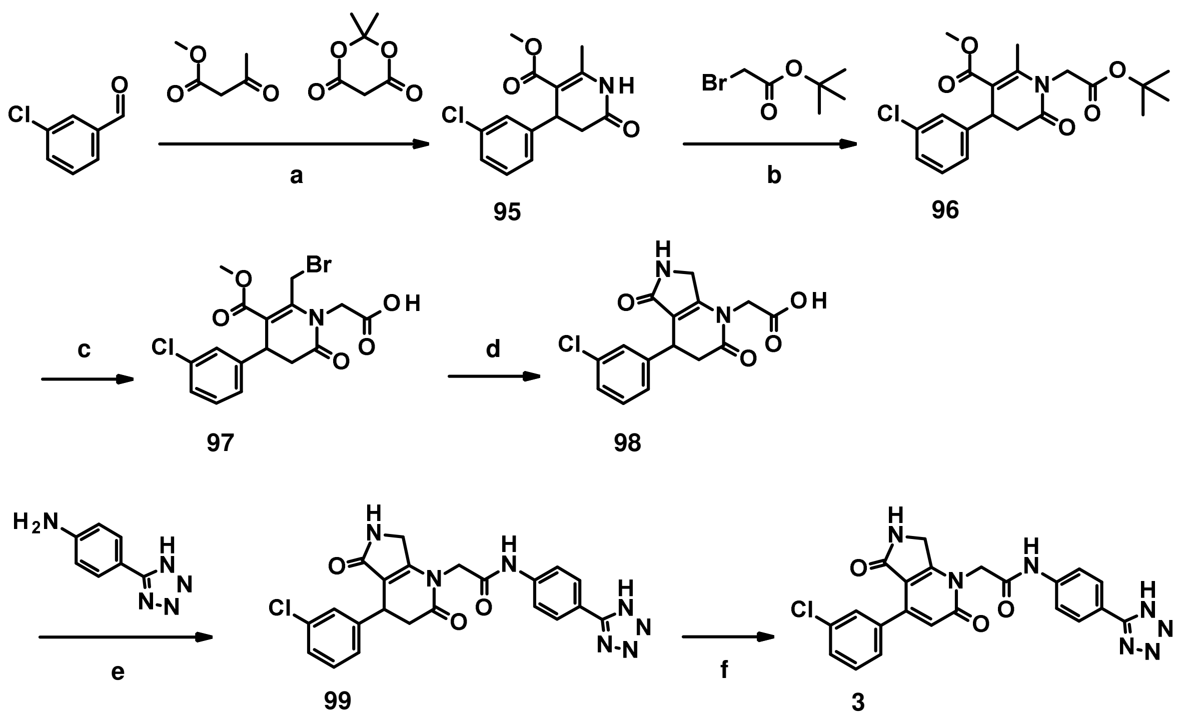

[a]Reagents and conditions: (a) $NH_4OAc$, EtOAc, RF, 38%; (b) $K_2CO_3$, DMF, 120 °C, 34%; (c) $Br_2$, DCM, 0 °C → RT, 66%; (d) $NH_3$ in MeOH, RT, 93%; (e) HATU, DIEA, DMF, RT, 5%: (f) CAN, water/acetone, RT, 41%.

**Methyl 4-(3-chlorophenyl)-2-methyl-6-oxo-1,4,5,6-tetrahydropyridine-3-carboxylate 95.**

A solution of 3-chlorobenzaldehyde (7.50 g, 53.35 mmol), methyl 3-oxobutanoate (6.20 g, 53.35 mmol, 1.0 eq.), 2,2-dimethyl-1,3-dioxane-4,6-dione (7.69 g, 53.35 mmol, 1.0 eq.) and ammonium acetate (4.30 g, 55.84 mmol, 1.05 eq.) in ethyl acetate (53 mL) was stirred under reflux for 5 h. After cooling to RT, the formed precipitate was filtered, washed with diethyl ether and dried *in vacuo* to give **95**. Yield: 5.66 g (38% of theory). LC/MS (method 1): $t_R$ = 0.91 min, MS (ESIpos): $m/z$ = 280 $[M+H]^+$.

**Methyl 1-(2-*tert*-butoxy-2-oxoethyl)-4-(3-chlorophenyl)-2-methyl-6-oxo-1,4,5,6-tetrahydro-pyridine-3-carboxylate (96).**

*tert*-Butyl bromoacetate (6.04 g, 30.97 mmol, 1.2 eq.) and potassium carbonate (7.13 g, 51.62 mmol, 2.0 eq.) were added under argon atmosphere at RT to a solution of methyl 4-(3-chlorophenyl)-2-methyl-6-oxo-1,4,5,6-tetrahydropyridine-3-carboxylate (**95**) (7.22 g, 25.81 mmol) in *N,N*-dimethylformamide (253 mL). The reaction mixture was stirred at 120 °C overnight, mixed with additional *tert*-butyl bromoacetate (2.52 g, 12.91 mmol, 0.5 eq.), stirred at 120 °C for 2 h, mixed with additional *tert*-butyl bromoacetate (2.52 g, 12.91 mmol, 0.5 eq.) and stirred at 120 °C overnight. After cooling to RT, *N,N*-

dimethylformamide was removed under reduced pressure. The residue was dissolved in ethyl acetate and extracted with water. The organic phase was dried over anhydrous magnesium sulfate, filtered, concentrated under reduced pressure and dried *in vacuo*. The residue was purified by flash silica gel chromatography (dichloromethane / methanol gradient) to give **96**. Yield: 3.88 g (88% purity, 34% of theory). LC/MS (method 4): $t_R$ = 1.20 min, MS (ESIpos): *m*/*z* = 394 $[M+H]^+$.

**[6-(Bromomethyl)-4-(3-chlorophenyl)-5-(methoxycarbonyl)-2-oxo-3,4-dihydropyridin-1(2*H*)-yl]acetic acid (97).**

Bromine (0.51 mL, 9.87 mmol, 1.0 eq.) was added under argon atmosphere to an ice-cooled solution of methyl 1-(2-*tert*-butoxy-2-oxoethyl)-4-(3-chlorophenyl)-2-methyl-6-oxo-1,4,5,6-tetrahydropyridine-3-carboxylate (**96**) (3.88 g, 9.87 mmol) in dichloromethane (79 mL). The reaction mixture was stirred at RT for 1 h, diluted with dichloromethane and washed with saturated aqueous sodium thiosulfate solution. The organic phase was dried over anhydrous sodium sulfate, filtered, concentrated under reduced pressure and dried *in vacuo* to give **97** which was used without further purification. Yield: 3.43 g (79% purity, 66% of theory). LC/MS (method 4): $t_R$ = 0.96 min, MS (ESIpos): *m*/*z* = 416 $[M+H]^+$.

**4-(3-Chlorophenyl)-2,5-dioxo-2,3,4,5,6,7-hexahydro-1*H*-pyrrolo[3,4-*b*]pyridin-1-yl]acetic acid (98).**

Ammonia solution (7 M in methanol, 12.10 mL) was added at RT to a mixture of [6-(bromomethyl)-4-(3-chlorophenyl)-5-(methoxycarbonyl)-2-oxo-3,4-dihydropyridin-1(2*H*)-yl]acetic acid (**97**) (1.41 g, 50% purity, 1.69 mmol) in acetonitrile (8 mL). The reaction mixture was stirred at RT for 30 min and concentrated under reduced pressure. The residue was stirred in aqueous hydrochloric acid solution (0.5 N, 60 mL), and the solid was filtered, washed with water and dried *in vacuo* to give **98**. Yield: 680 mg (74% purity, 93% of theory). LC/MS (method 1): $t_R$ = 0.58 min, MS (ESIpos): *m*/*z* = 321 $[M+H]^+$.

**2-[4-(3-Chlorophenyl)-2,5-dioxo-2,3,4,5,6,7-hexahydro-1*H*-pyrrolo[3,4-*b*]pyridin-1-yl]-*N*-[4-(1*H*-tetrazol-5-yl)phenyl]acetamide (99).**

A solution of HATU (498 mg, 1.31 mmol, 1.2 eq.) in *N,N*-dimethylformamide (10 mL) was added under argon atmosphere at RT to a solution of 4-(3-chlorophenyl)-2,5-dioxo-2,3,4,5,6,7-hexahydro-1*H*-pyrrolo[3,4-*b*]pyridin-1-yl]acetic acid (**98**) (500 mg, 70% purity, 1.09 mmol), 4-(1*H*-tetrazol-5-yl)aniline (211 mg, 1.31 mmol, 1.2 eq.) and *N,N*-diisopropylethylamine (418 $\mu$L, 2.40 mmol, 2.2 eq.) in *N,N*-dimethylformamide (20 mL). The reaction mixture was stirred at RT overnight and concentrated under reduced pressure. After addition of dichloromethane / water, the liquid was decanted, the remaining residue dissolved in methanol and concentrated under reduced pressure. The residue was stirred in acetonitrile, and the solid was filtered and dried *in vacuo*. The residue was purified by preparative RP-HPLC (acetonitrile / water gradient) to give **99**. Yield: 24 mg (5% of theory). LC/MS (method 4): $t_R$ = 0.75 min, MS (ESIpos): *m*/*z* = 464 $[M+H]^+$; $^1$H NMR (400 MHz, DMSO-$d_6$): δ [ppm] =

10.64 (s, 1H), 8.01 (d, $J$ = 8.6 Hz, 2H), 7.84-7.80 (m, 3H), 7.43 (s, 1H), 7.37-7.28 (m, 3H), 4.59 (d, $J$ = 16.9 Hz, 1H), 4.36 (d, $J$ = 16.9 Hz, 1H), 4.12 (dd, $J$ = 24.8 Hz, 18.7 Hz, 2H), 3.98 (d, $J$ = 8.6 Hz, 1H), 3.23 (dd, $J$ = 16.4 Hz, 8.8 Hz, 1H), 2.63 (d, $J$ = 16.4 Hz, 1H).

**2-[4-(3-Chlorophenyl)-2,5-dioxo-2,5,6,7-tetrahydro-1*H*-pyrrolo[3,4-b]pyridin-1-yl]-*N*-[4-(1*H*-tetrazol-5-yl)phenyl]acetamide (3).**

A solution of ammonium cerium(IV) nitrate (369 mg, 0.67 mmol, 4.0 eq.) in water (0.7 mL) was added at RT to a suspension of 2-[4-(3-chlorophenyl)-2,5-dioxo-2,3,4,5,6,7-hexahydro-1*H*-pyrrolo[3,4-*b*]pyridin-1-yl]-*N*-[4-(1*H*-tetrazol-5-yl)phenyl]acetamide (**99**) (78 mg, 0.17 mmol) in acetone (2.7 mL). The reaction mixture was stirred at RT overnight and added to water. The precipitate was filtered and dried *in vacuo*. The residue was purified by preparative RP-HPLC (acetonitrile / water gradient) to give **3**. Yield: 32 mg (41% of theory). LC/MS (method 6): $t_R$ = 0.86 min, MS (ESIpos): $m/z$ = 462 $[M+H]^+$; $^1$H NMR (400 MHz, DMSO-$d_6$): δ [ppm] = 10.81 (s, 1H), 8.32 (s, 1H), 8.00 (d, $J$ = 8.8 Hz, 2H), 7.79 (d, $J$ = 8.8 Hz, 2H), 7.65 (s, 1H), 7.56-7.44 (m, 3H), 6.42 (s, 1H), 4.86 (s, 2H), 4.39 (s, 2H).

**Compounds of Table 1.**

**Synthesis of compound 4.**[a]

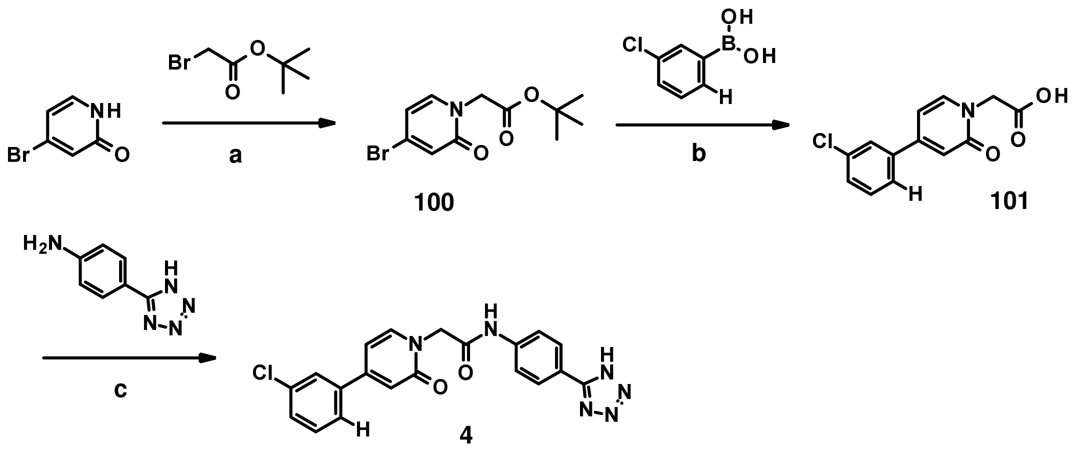


[a]Reagents and conditions: (a) $K_2CO_3$, DMF, 120 °C, 95%; (b) $Pd(Ph_3P)_4$, aq. $Na_2CO_3$, 1,4-dioxane, 150 °C/microwave, 61%; (c) HATU, DIEA, DMF, RT, 20%.

***tert*-Butyl (4-bromo-2-oxopyridin-1(2*H*)-yl)acetate (100).**

*tert*-Butyl bromoacetate (6.65 g, 34.07 mmol, 1.2 eq.) and potassium carbonate (7.85 g, 56.78 mmol, 2.0 eq.) were added under argon atmosphere at RT to a solution of 4-bromopyridin-2(1*H*)-one (4.94 g, 28.39 mmol) in *N*,*N*-dimethylformamide (247 mL). The reaction mixture was stirred at 120 °C for 2 h and added to iced water. After addition of ethyl acetate, the aqueous phase was extracted with ethyl acetate. The combined organic phases were washed with brine, dried over anhydrous sodium sulfate, filtered, concentrated under reduced pressure and dried *in vacuo* to give **100** which was used without

further purification. Yield: 8.55 g (91% purity, 95% of theory). LC/MS (method 1): $t_R$ = 0.88 min, MS (ESIpos): *m*/*z* = 288 $[M+H]^+$.

**[4-(3-Chlorophenyl)-2-oxopyridin-1(2*H*)-yl]acetic acid (101).**

(3-Chlorophenyl)boronic acid (179 mg, 1.15 mmol, 1.1 eq.) and tetrakis(triphenylphosphine)palladium (120 mg, 0.10 mmol, 0.1 eq.) were added under argon atmosphere at RT to a mixture of *tert*-butyl (4-bromo-2-oxopyridin-1(2*H*)-yl)acetate (**100**) (300 mg, 1.04 mmol) in saturated aqueous sodium carbonate solution (6 mL) and 1,4-dioxane (6 mL). The reaction mixture was stirred in the microwave at 150 °C for 10 min and filtered through Celite®, and the filter residue was washed with 1,4-dioxane. The combined filtrates were concentrated under reduced pressure. The residue was dried *in vacuo*, stirred in a mixture of dichloromethane and methanol, and the solid was filtered. The combined filtrates were concentrated under reduced pressure. The residue was dried *in vacuo* and stirred in dichloromethane, and the solid was filtered and dried *in vacuo* to give the already saponified carboxylic acid **101** as raw material, still contaminated with triphenylphosphine. Yield: 256 mg (65% purity, 61% of theory). LC/MS (method 4): $t_R$ = 0.78 min, MS (ESIpos): *m*/*z* = 264 $[M+H]^+$.

**2-[4-(3-Chlorophenyl)-2-oxopyridin-1(2*H*)-yl]-*N*-[4-(1*H*-tetrazol-5-yl)phenyl]acetamide (4).**

A solution of HATU (125 mg, 0.33 mmol, 1.2 eq.) in *N,N*-dimethylformamide (2 mL) was added under argon atmosphere at RT to a solution of [4-(3-chlorophenyl)-2-oxopyridin-1(2*H*)-yl]acetic acid (**101**) (111 mg, 65% purity, 0.27 mmol), 4-(1*H*-tetrazol-5-yl)aniline (53 mg, 0.33 mmol, 1.2 eq.) and *N,N*-diisopropylethylamine (105 $\mu$L, 0.60 mmol, 2.2 eq.) in *N,N*-dimethylformamide (3 mL). The reaction mixture was stirred at RT overnight and concentrated under reduced pressure. The residue was stirred in water, and the solid was filtered and dried *in vacuo*. The residue was purified by preparative RP-HPLC (acetonitrile / water gradient) to give **4**. Yield: 23 mg (20% of theory). LC/MS (method 6): $t_R$ = 1.00 min, MS (ESIpos): *m*/*z* = 407 $[M+H]^+$; $^1H$ NMR (400 MHz, DMSO-$d_6$): δ [ppm] = 16.7 (br s, 1H), 10.70 (s, 1H), 8.00 (d, *J* = 8.8 Hz, 2H), 7.85-7.77 (m, 4H), 7.76-7.70 (m, 1H), 7.56-7.50 (m, 2H), 6.77 (d, *J* = 1.7 Hz, 1H), 6.69 (dd, *J* = 7.1 Hz, 2.0 Hz, 1H), 4.83 (s, 2H).

**Synthesis of compound 5.**[a]

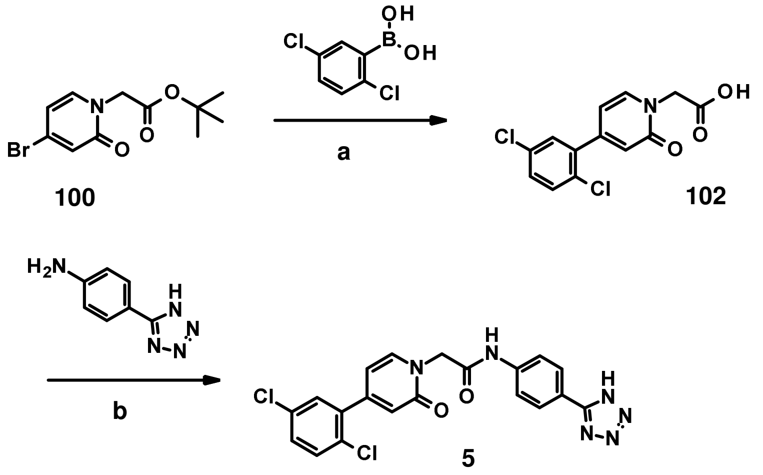


[a]Reagents and conditions: (a) $Pd(Ph_3P)_4$, aq. $Na_2CO_3$, 1,4-dioxane, 150 °C/microwave; (b) HATU, DIEA, DMF, RT, 22%.

**[4-(2,5-Dichlorophenyl)-2-oxopyridin-1(2*H*)-yl]acetic acid (102).**

(2,5-Dichlorophenyl)boronic acid (291 mg, 1.53 mmol, 1.1 eq.) and tetrakis(triphenylphosphine)-palladium (160 mg, 0.14 mmol, 0.1 eq.) were added under argon atmosphere at RT to a mixture of *tert*-butyl (4-bromo-2-oxopyridin-1(2*H*)-yl)acetate (**100**) (400 mg, 1.39 mmol) in saturated aqueous sodium carbonate solution (8 mL) and 1,4-dioxane (8 mL). The reaction mixture was stirred in the microwave at 150 °C for 10 min and filtered through Celite®, and the filter residue was washed with 1,4-dioxane. The combined filtrates were concentrated under reduced pressure. The residue was stirred in dichloromethane, and the solid was filtered and dried *in vacuo* to give the already saponified carboxylic acid **102** as raw material, still contaminated with salts, which was used without further purification. Yield: 364 mg. LC/MS (method 7): $t_R$ = 0.81 min, MS (ESIpos): $m/z$ = 298 $[M+H]^+$.

**2-[4-(2,5-Dichlorophenyl)-2-oxopyridin-1(2*H*)-yl]-*N*-[4-(1*H*-tetrazol-5-yl)phenyl]acetamide (5).**

A solution of HATU (199 mg, 0.44 mmol, 1.2 eq.) in *N,N*-dimethylformamide (2 mL) was added under argon atmosphere at RT to a solution of [4-(2,5-dichlorophenyl)-2-oxopyridin-1(2*H*)-yl]acetic acid (**102**) (200 mg, 65% purity, 0.44 mmol), 4-(1*H*-tetrazol-5-yl)aniline (84 mg, 0.52 mmol, 1.2 eq.) and *N,N*-diisopropylethylamine (167 $\mu$L, 0.96 mmol, 2.2 eq.) in *N,N*-dimethylformamide (3 mL). The reaction mixture was stirred at RT overnight and concentrated under reduced pressure. The residue was stirred in water, and the solid was filtered and dried *in vacuo*. The residue was purified by preparative RP-HPLC (acetonitrile / water gradient) to give **5**. Yield: 43 mg (22% of theory). LC/MS (method 7): $t_R$ = 0.88 min, MS (ESIpos): $m/z$ = 441 $[M+H]^+$; $^1H$ NMR (400 MHz, DMSO-$d_6$): δ [ppm] = 16.7 (br s, 1H), 10.73 (s, 1H), 8.00 (d, $J$ = 8.8 Hz, 2H), 7.82 (d, $J$ = 8.6 Hz, 2H), 7.79 (d, $J$ = 6.8 Hz, 1H), 7.64 (d, $J$ = 8.3 Hz, 1H), 7.60-7.53 (m, 2H), 6.49 (d, $J$ = 1.7 Hz, 1H), 6.38 (dd, $J$ = 6.9 Hz, 2.0 Hz, 1H), 4.85 (s, 2H).

**Synthesis of compound 6.**[a]

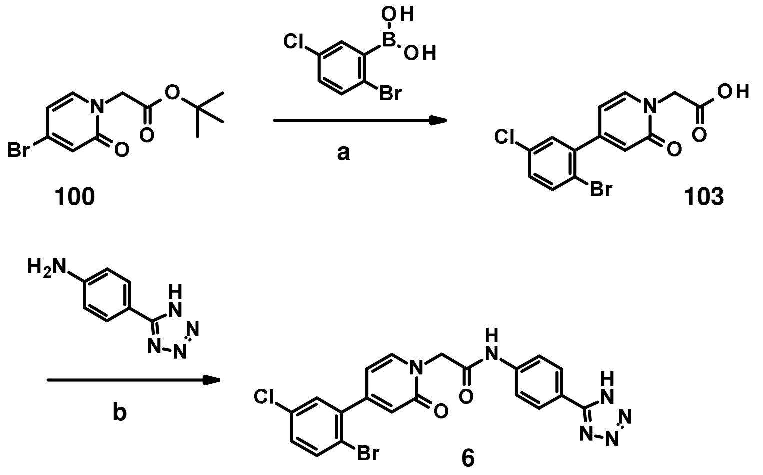


[a]Reagents and conditions: (a) $Pd(Ph_3P)_4$, aq. $Na_2CO_3$, 1,4-dioxane, 150 °C/microwave; (b) HATU, DIEA, DMF, RT, 36%.

**[4-(2-Bromo-5-chlorophenyl)-2-oxopyridin-1(2*H*)-yl]acetic acid (103).**

(2-Bromo-5-chlorophenyl)boronic acid (152 mg, 0.65 mmol, 1.1 eq.) and tetrakis(triphenylphosphine)-palladium (68 mg, 0.06 mmol, 0.1 eq.) were added under argon atmosphere at RT to a mixture of *tert*-butyl (4-bromo-2-oxopyridin-1(2*H*)-yl)acetate (**100**) (173 mg, 0.59 mmol) in saturated aqueous sodium carbonate solution (3.5 mL) and 1,4-dioxane (3.5 mL). The reaction mixture was stirred in the microwave at 150 °C for 10 min and filtered through Celite®, and the filter residue was washed with 1,4-dioxane. The combined filtrates were concentrated under reduced pressure. The residue was stirred in dichloromethane, and the solid was filtered and dried *in vacuo* to give the already saponified carboxylic acid **103** as raw material, still contaminated with salts, which was used without further purification. Yield: 501 mg. LC/MS (method 1): $t_R$ = 1.13 min, MS (ESIpos): *m*/*z* = 398 $[M+H]^+$.

**2-[4-(2-Bromo-5-chlorophenyl)-2-oxopyridin-1(2*H*)-yl]-*N*-[4-(1*H*-tetrazol-5-yl)phenyl]acetamide (6).**

A solution of HATU (107 mg, 0.28 mmol, 1.2 eq.) in *N*,*N*-dimethylformamide (2 mL) was added under argon atmosphere at RT to a solution of [4-(2-bromo-5-chlorophenyl)-2-oxopyridin-1(2*H*)-yl]acetic acid (**103**) (200 mg, 40% purity, 0.23 mmol), 4-(1*H*-tetrazol-5-yl)aniline (41 mg, 0.26 mmol, 1.1 eq.) and *N*,*N*-diisopropylethylamine (89 $\mu$L, 0.51 mmol, 2.2 eq.) in *N*,*N*-dimethylformamide (3 mL). The reaction mixture was stirred at RT for 1 h, added to iced water and concentrated under reduced pressure. The residue was purified by preparative RP-HPLC (acetonitrile / water gradient) to give **6**. Yield: 41 mg (36% of theory). LC/MS (method 4): $t_R$ = 0.94 min, MS (ESIpos): *m*/*z* = 485 $[M+H]^+$; $^1H$ NMR (400 MHz, DMSO-$d_6$): δ [ppm] = 10.44 (s, 1H), 7.91 (d, *J* = 8.8 Hz, 2H), 7.79 (d, *J* = 8.6 Hz, 1H), 7.78 (d, *J* = 7.1 Hz, 1H), 7.62 (d, *J* = 8.8 Hz, 2H), 7.54 (d, *J* = 2.7 Hz, 1H), 7.46 (dd, *J* = 8.6 Hz, 2.7 Hz, 1H), 6.43 (d, *J* = 2.0 Hz, 1H), 6.33 (dd, *J* = 7.1 Hz, 2.0 Hz, 1H), 4.82 (s, 2H).

**Synthesis of compound 7.**[a]

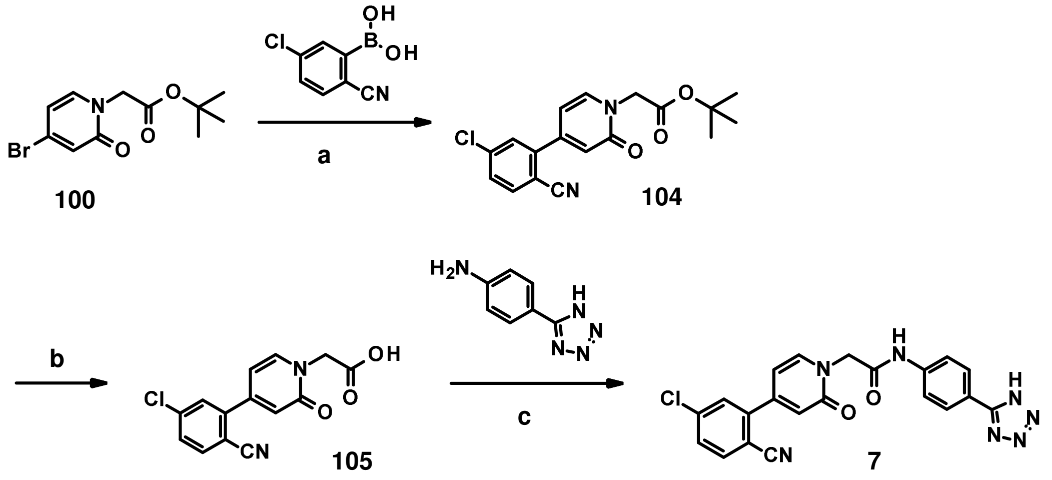


[a]Reagents and conditions: (a) $Pd(Ph_3P)_4$, $K_2CO_3$, 1,4-dioxane, 110 °C, 65%; (b) TFA, DCM, RT, 44%; (c) HATU, DIEA, DMF, RT, 23%.

***tert*-Butyl [4-(5-chloro-2-cyanophenyl)-2-oxopyridin-1(2*H*)-yl]acetate (104).**

In two consecutive campaigns, a mixture of *tert*-butyl (4-bromo-2-oxopyridin-1(2*H*)-yl)acetate (**100**) (450 mg, 1.56 mmol), (5-chloro-2-cyanophenyl)boronic acid (326 mg, 1.80 mmol, 1.15 eq.) and potassium carbonate (648 mg, 4.69 mmol, 3 eq.) in 1,4-dioxane (18 mL) was flushed with argon at RT, followed by addition of tetrakis(triphenylphosphine)palladium (180 mg, 0.16 mmol, 0.1 eq.). Both reaction mixtures were stirred at 110 °C overnight, combined and filtered through Celite®, and the filter residue was washed with 1,4-dioxane. The combined filtrates were concentrated under reduced pressure. The residue was dried *in vacuo* to give **104** which was used without further purification. Yield: 625 mg (50% purity, 65% of theory). LC/MS (method 4): $t_R$ = 1.00 min, MS (ESIpos): $m/z$ = 289 $[M\text{-}tBu+H]^+$.

**[4-(5-Chloro-2-cyanophenyl)-2-oxopyridin-1(2*H*)-yl]acetic acid (105).**

Trifluoroacetic acid (1.40 mL, 18.13 mmol, 20 eq.) was added under argon atmosphere at RT to a solution of *tert*-butyl [4-(5-chloro-2-cyanophenyl)-2-oxopyridin-1(2*H*)-yl]acetate (**104**) (625 mg, 50% purity, 0.91 mmol) in dichloromethane (5 mL). The reaction mixture was stirred at RT for 3 h and concentrated under reduced pressure. The residue was stirred in water, and the solid was filtered and dried *in vacuo* to give **105** which was used without further purification. Yield: 239 mg (50% purity, 44% of theory). LC/MS (method 6): $t_R$ = 0.81 min, MS (ESIpos): $m/z$ = 289 $[M+H]^+$.

**2-[4-(5-Chloro-2-cyanophenyl)-2-oxopyridin-1(2*H*)-yl]-*N*-[4-(1*H*-tetrazol-5-yl)phenyl]acetamide (7).**

A solution of HATU (94 mg, 0.25 mmol, 1.2 eq.) in *N,N*-dimethylformamide (1 mL) was added under argon atmosphere at RT to a solution of [4-(5-chloro-2-cyanophenyl)-2-oxopyridin-1(2*H*)-yl]acetic acid (**105**) (120 mg, 50% purity, 0.21 mmol), 4-(1*H*-tetrazol-5-yl)aniline (40 mg, 0.25 mmol, 1.2 eq.) and

*N,N*-diisopropylethylamine (80 $\mu$L, 0.46 mmol, 2.2 eq.) in *N,N*-dimethylformamide (2 mL). The reaction mixture was stirred at RT overnight and purified without further work-up by preparative RP-HPLC (acetonitrile / water gradient) to give **7**. Yield: 23 mg (23% of theory). LC/MS (method 4): $t_R$ = 0.76 min, MS (ESIpos): $m/z$ = 432 $[M+H]^+$; $^1$H NMR (400 MHz, DMSO-$d_6$): δ [ppm] = 16.7 (br s, 1H), 10.76 (s, 1H), 8.05 (d, $J$ = 8.3 Hz, 1H), 8.01 (d, $J$ = 8.8 Hz, 2H), 7.88 (d, $J$ = 7.1 Hz, 1H), 7.86-7.79 (m, 3H), 7.77 (dd, $J$ = 8.3 Hz, 2.0 Hz, 1H), 6.68 (d, $J$ = 2.0 Hz, 1H), 6.53 (dd, $J$ = 6.9 Hz, 2.0 Hz, 1H), 4.87 (s, 2H).

**Synthesis of racemate 8.**[a]

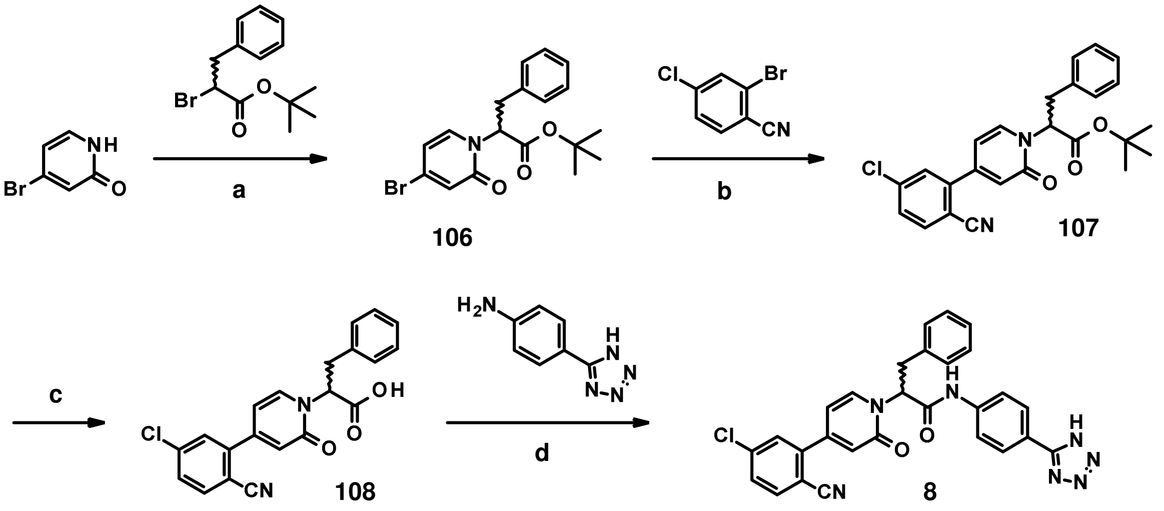


[a]Reagents and conditions: (a) $K_2CO_3$, DMF, 120 °C, 20%; (b) $Pd(Ph_3P)_4$, aq. $Na_2CO_3$, 1,4-dioxane, 110 °C, 67%; (c) TFA, DCM, RT, 100%; (d) HATU, DIEA, DMF, RT, 16%.

***tert*-Butyl 2-(4-bromo-2-oxopyridin-1(2*H*)-yl)-3-phenylpropanoate (racemate 106).**

A mixture of 4-bromopyridin-2(1*H*)-one hydrochloride (210 mg, 1.00 mmol), racemic *tert*-butyl 2-chloro-3-phenylpropanoate (289 mg, 1.20 mmol, 1.2 eq.) and potassium carbonate (346 mg, 2.50 mmol, 2.5 eq.) in *N,N*-dimethylformamide (10 mL) was stirred under argon atmosphere at 120 °C for 4 h and concentrated under reduced pressure. After addition of ethyl acetate / water and phase separation, the aqueous phase was extracted with ethyl acetate. The combined organic phases were washed with brine, dried over anhydrous sodium sulfate, filtered and concentrated under reduced pressure. The residue was purified by preparative RP-HPLC (methanol / water gradient) to give **106**. Yield: 74 mg (20% of theory). LC/MS (method 1): $t_R$ = 1.14 min, MS (ESIpos): $m/z$ = 322 $[M\text{-}tBu+H]^+$.

***tert*-Butyl 2-[4-(5-chloro-2-cyanophenyl)-2-oxopyridin-1(2*H*)-yl]-3-phenylpropanoate (racemate 107).**

A mixture of *tert*-butyl 2-(4-bromo-2-oxopyridin-1(2*H*)-yl)-3-phenylpropanoate (racemate **106**) (72 mg, 0.19 mmol), (5-chloro-2-cyanophenyl)boronic acid (40 mg, 0.22 mmol, 1.15 eq.), potassium

carbonate (79 mg, 0.57 mmol, 3.0 eq.) in 1,4-dioxane (3 mL) was flushed with argon at RT, followed by addition of tetrakis(triphenylphosphine)palladium (22 mg, 0.02 mmol, 0.1 eq.). The reaction mixture was stirred at 110 °C (preheated oil bath) for 5 h, cooled to RT and filtered through Celite®, and the filter residue was washed with 1,4-dioxane. The combined filtrates were concentrated under reduced pressure. The residue was purified by preparative RP-HPLC (methanol / water gradient) to give **107**. Yield: 58 mg (67% of theory). LC/MS (method 1): $t_R$ = 1.22 min, MS (ESIpos): *m*/*z* = 435 $[M+H]^+$.

**2-[4-(5-Chloro-2-cyanophenyl)-2-oxopyridin-1(2*H*)-yl]-3-phenylpropanoic acid (racemate 108).**

Trifluoroacetic acid (195 $\mu$L, 2.53 mmol, 20 eq.) was added under argon atmosphere at RT to a solution of *tert*-butyl 2-[4-(5-chloro-2-cyanophenyl)-2-oxopyridin-1(2*H*)-yl]-3-phenylpropanoate (racemate **107**) (58 mg, 0.13 mmol) in dichloromethane (4 mL). The reaction mixture was stirred at RT for 5 h and concentrated under reduced pressure. The residue was coevaporated several times with dichloromethane and dried *in vacuo* to give **108** which was used without further purification. Yield: 77 mg (85% purity, 100% of theory). LC/MS (method 4): $t_R$ = 0.97 min, MS (ESIpos): *m*/*z* = 379 $[M+H]^+$.

**2-[4-(5-Chloro-2-cyanophenyl)-2-oxopyridin-1(2*H*)-yl]-3-phenyl-*N*-[4-(1*H*-tetrazol-5-yl)phenyl]propenamide diethylamine adduct (racemate 8).**

A solution of HATU (79 mg, 0.21 mmol, 1.2 eq.) in *N,N*-dimethylformamide (2 mL) was added under argon atmosphere at RT to a solution of 2-[4-(5-chloro-2-cyanophenyl)-2-oxopyridin-1(2*H*)-yl]-3-phenylpropanoic acid (racemate **108**) (77 mg, 85% purity, 0.17 mmol), 4-(1*H*-tetrazol-5-yl)aniline (31 mg, 0.19 mmol, 1.1 eq.) and *N,N*-diisopropylethylamine (90 $\mu$L, 0.52 mmol, 3.0 eq.) in *N,N*-dimethylformamide (3 mL). The reaction mixture was stirred at RT overnight and concentrated under reduced pressure. The residue was stirred in water, and the solid was filtered, washed with water, dissolved in a mixture of methanol / acetonitrile and purified by preparative RP-HPLC (methanol / water gradient) to give **8**. Yield: 17 mg (94% purity, 16% of theory). LC/MS (method 1): $t_R$ = 1.02 min, MS (ESIpos): *m*/*z* = 522 $[M+H]^+$; $^1$H NMR (400 MHz, DMSO-$d_6$): δ [ppm] = 10.66 (s, 1H), 8.16 (d, *J* = 7.1 Hz, 1H), 8.01 (d, *J* = 8.3 Hz, 1H), 7.92 (d, *J* = 8.6 Hz, 2H), 7.77 (d, *J* = 2.0 Hz, 1H), 7.74 (dd, *J* = 8.3 Hz, 2.0 Hz, 1H), 7.65 (d, *J* = 8.8 Hz, 2H), 7.33-7.24 (m, 4H), 7.21-7.16 (m, 1H), 6.56 (d, *J* = 2.0 Hz, 1H), 6.50 (dd, *J* = 7.3 Hz, 2.0 Hz, 1H), 6.10 (dd, *J* = 10.0 Hz, 5.9 Hz, 1H), 2.93 (q, *J* = 7.3 Hz, 4H), 1.15 (t, *J* = 7.2 Hz, 6H).

**Synthesis of racemate 9.**[a]

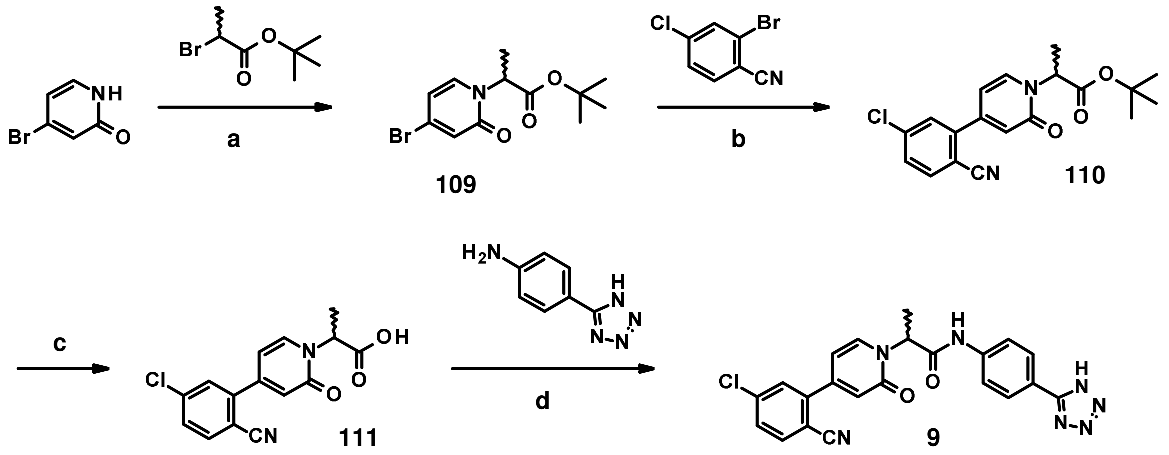

[a]Reagents and conditions: (a) $K_2CO_3$, DMF, 120 °C, 69%; (b) $Pd(Ph_3P)_4$, aq. $Na_2CO_3$, 1,4-dioxane, 110 °C, 77%; (c) TFA, DCM, RT, 94%; (d) HATU, DIEA, DMF, RT, 22%.

***tert*-Butyl 2-(4-bromo-2-oxopyridin-1(2*H*)-yl)propanoate (racemate 109).**

A mixture of 4-bromopyridin-2(1*H*)-one (6.00 g, 34.48 mmol), racemic *tert*-butyl 2-bromopropanoate (7.93 g, 37.93 mmol, 1.1 eq.) and potassium carbonate (10.96 g, 79.31 mmol, 2.3 eq.) in *N,N*-dimethylformamide (116 mL) was stirred under argon atmosphere at 120 °C for 45 min and concentrated under reduced pressure. The residue was stirred in water, and the solid was filtered, washed with water and dried *in vacuo*. The residue was purified by flash silica gel chromatography (cyclohexane / ethyl acetate gradient) to give **109**. Yield: 7.37 g (69% of theory). LC/MS (method 1): $t_R$ = 0.94 min, MS (ESIpos): *m*/*z* = 302 $[M+H]^+$; $^1H$ NMR (400 MHz, DMSO-$d_6$): δ [ppm] = 7.66 (d, *J* = 7.5 Hz, 1H), 6.75 (d, *J* = 2.2 Hz, 1H), 6.51 (dd, *J* = 7.3 Hz, 2.2 Hz, 1H), 5.04 (q, *J* = 7.3 Hz, 1H), 1.51 (d, *J* = 7.2 Hz, 3H), 1.37 (s, 9H).

***tert*-Butyl 2-[4-(5-chloro-2-cyanophenyl)-2-oxopyridin-1(2*H*)-yl]propanoate (racemate 110).**

A mixture of *tert*-butyl 2-(4-bromo-2-oxopyridin-1(2*H*)-yl)propanoate (racemate **109**) (2.40 g, 74% purity, 5.88 mmol), (5-chloro-2-cyanophenyl)boronic acid (1.23 g, 6.76 mmol, 1.15 eq.), potassium carbonate (2.44 g, 17.63 mmol, 3.0 eq.) and tetrakis(triphenylphosphine)palladium (679 mg, 0.59 mmol, 0.1 eq.) was flushed with argon at RT and mixed with 1,4-dioxane (90 mL). The reaction mixture was stirred at 110 °C (preheated oil bath) for 14 h, cooled to RT and filtered through Celite®, and the filter residue was washed with 1,4-dioxane. The combined filtrates were concentrated under reduced pressure. The residue was purified by flash silica gel chromatography (cyclohexane / ethyl acetate gradient) to give **110**. Yield: 1.86 g (87% purity, 77% of theory). LC/MS (method 1): $t_R$ = 1.07 min, MS (ESIpos): *m*/*z* = 359 $[M+H]^+$; $^1H$ NMR (400 MHz, DMSO-$d_6$): δ [ppm] = 8.03 (d, *J* = 8.4 Hz, 1H), 7.84 (d, *J* = 7.2 Hz, 1H), 7.81 (d, *J* = 2.1 Hz, 1H), 7.75 (dd, *J* = 8.3 Hz, 2.1 Hz, 1H), 6.64 (d, *J* = 2.0 Hz, 1H), 6.50 (dd, *J* = 7.2 Hz, 2.0 Hz, 1H), 5.14 (q, *J* = 7.2 Hz, 1H), 1.58 (d, *J* = 7.2 Hz, 3H), 1.40 (s, 9H).

**2-[4-(5-Chloro-2-cyanophenyl)-2-oxopyridin-1(2*H*)-yl]propanoic acid (racemate 111).**

Trifluoroacetic acid (7.75 mL, 100.55 mmol, 20 eq.) was added under argon atmosphere at RT to a solution of *tert*-butyl 2-[4-(5-chloro-2-cyanophenyl)-2-oxopyridin-1(2*H*)-yl]propanoate (racemate **110**) (2.20 g, 82% purity, 5.03 mmol) in dichloromethane (42 mL). The reaction mixture was stirred at RT for 3 h and concentrated under reduced pressure. The residue was coevaporated with dichloromethane and dried *in vacuo*. The residue was stirred in diethyl ether, and the solid was filtered and dried *in vacuo* to give **111**. Yield: 1.48 g (97% purity, 94% of theory). LC/MS (method 1): $t_R$ = 0.80 min, MS (ESIpos): *m*/*z* = 303 $[M+H]^+$; $^1H$ NMR (400 MHz, DMSO-$d_6$): δ [ppm] = 13.04 (br s, 1H), 8.04 (d, *J* = 8.3 Hz, 1H), 7.88 (d, *J* = 7.2 Hz, 1H), 7.82 (d, *J* = 2.1 Hz, 1H), 7.76 (dd, *J* = 8.4 Hz, 2.1 Hz, 1H), 6.65 (d, *J* = 2.0 Hz, 1H), 6.51 (dd, *J* = 7.1 Hz, 2.1 Hz, 1H), 5.23 (q, *J* = 7.2 Hz, 1H), 1.60 (d, *J* = 7.2 Hz, 3H).

**2-[4-(5-Chloro-2-cyanophenyl)-2-oxopyridin-1(2*H*)-yl]-*N*-[4-(1*H*-tetrazol-5-yl)phenyl]propanamide (racemate 9).**

A solution of HATU (81 mg, 0.21 mmol, 1.2 eq.) in *N,N*-dimethylformamide (2 mL) was added under argon atmosphere at RT to a solution of 2-[4-(5-chloro-2-cyanophenyl)-2-oxopyridin-1(2*H*)-yl]propanoic acid (racemate **111**) (65 mg, 83% purity, 0.18 mmol), 4-(1*H*-tetrazol-5-yl)aniline (32 mg, 0.20 mmol, 1.1 eq.) and *N,N*-diisopropylethylamine (68 μL, 0.39 mmol, 2.2 eq.) in *N,N*-dimethylformamide (3 mL). The reaction mixture was stirred at RT for 1 h and concentrated under reduced pressure. The residue was purified by preparative RP-HPLC (acetonitrile / water gradient) to give **9**. Yield: 17 mg (22% of theory). LC/MS (method 1): $t_R$ = 0.82 min, MS (ESIpos): *m*/*z* = 446 $[M+H]^+$; $^1H$ NMR (400 MHz, DMSO-$d_6$): δ [ppm] = 10.78 (s, 1H), 8.08-7.94 (m, 4H), 7.87-7.80 (m, 3H), 7.78 (dd, *J* = 8.6 Hz, 2.0 Hz, 1H), 6.67 (d, *J* = 1.2 Hz, 1H), 6.58 (dd, *J* = 7.1 Hz, 2.0 Hz, 1H), 5.59 (q, *J* = 7.3 Hz, 1H), 1.71 (d, *J* = 7.3 Hz, 3H).

**Synthesis of racemate 10.**[a]

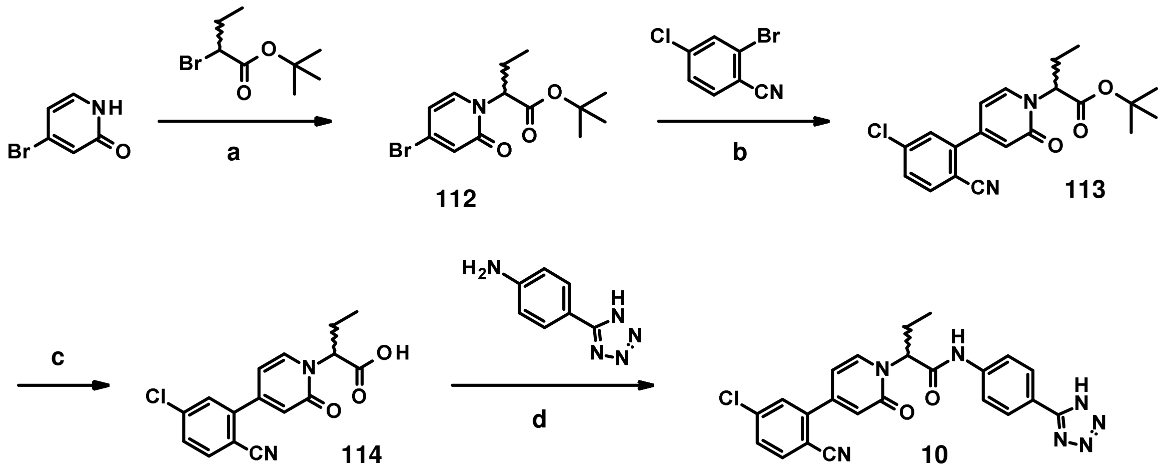


[a]Reagents and conditions: (a) $K_2CO_3$, DMF, 120 °C, 79%; (b) $Pd(Ph_3P)_4$, $K_2CO_3$, 1,4-dioxane, 110 °C, 55%; (c) TFA, DCM, RT, 94%; (d) HATU, DIEA, DMF, RT, 77%.

***tert*-Butyl 2-(4-bromo-2-oxopyridin-1(2*H*)-yl)butanoate (racemate 112).**

A mixture of 4-bromopyridin-2(1*H*)-one (348 mg, 2.00 mmol), racemic *tert*-butyl 2-bromobutanoate (535 mg, 2.40 mmol, 1.2 eq.) and potassium carbonate (415 mg, 3.00 mmol, 1.5 eq.) in *N,N*-dimethylformamide (30 mL) was stirred under argon atmosphere at 120 °C for 2 h and concentrated under reduced pressure. The residue was dissolved in ethyl acetate and extracted with water. The organic phase was washed with brine, dried over anhydrous sodium sulfate, filtered, concentrated under reduced pressure and dried *in vacuo* to give **112** which was used without further purification. Yield: 608 mg (82% purity, 79% of theory). LC/MS (method 1): $t_R$ = 0.99 min, MS (ESIpos): *m*/*z* = 316 $[M+H]^+$.

***tert*-Butyl 2-[4-(5-chloro-2-cyanophenyl)-2-oxopyridin-1(2*H*)-yl]butanoate (racemate 113).**

A mixture of *tert*-butyl 2-(4-bromo-2-oxopyridin-1(2*H*)-yl)butanoate (racemate **112**) (600 mg, 82% purity, 1.56 mmol), (5-chloro-2-cyanophenyl)boronic acid (325 mg, 1.79 mmol, 1.15 eq.), potassium carbonate (645 mg, 4.67 mmol, 3.0 eq.) in 1,4-dioxane (20 mL) was flushed with argon at RT, followed by addition of tetrakis(triphenylphosphine)palladium (180 mg, 0.16 mmol, 0.1 eq.). The reaction mixture was stirred at 110 °C (preheated oil bath) for 5 h, cooled to RT and filtered through Celite®, and the filter residue was washed with 1,4-dioxane. The combined filtrates were concentrated under reduced pressure. The residue was dissolved in ethyl acetate and extracted with water. The organic phase was washed with brine, dried over anhydrous sodium sulfate, filtered and concentrated under reduced pressure. The residue was purified by flash silica gel chromatography (dichloromethane / methanol mixture) to give **113**. Yield: 543 mg (59% purity, 55% of theory). LC/MS (method 1): $t_R$ = 1.10 min, MS (ESIpos): *m*/*z* = 373 $[M+H]^+$.

**2-[4-(5-Chloro-2-cyanophenyl)-2-oxopyridin-1(2*H*)-yl]butanoic acid (racemate 114).**

Trifluoroacetic acid (1.32 mL, 17.19 mmol, 20 eq.) was added under argon atmosphere at RT to a solution of *tert*-butyl 2-[4-(5-chloro-2-cyanophenyl)-2-oxopyridin-1(2*H*)-yl]butanoate (racemate **113**) (543 mg, 59% purity, 0.86 mmol) in dichloromethane (10 mL). The reaction mixture was stirred at RT overnight and concentrated under reduced pressure. The residue was coevaporated with dichloromethane and dried *in vacuo* to give **114** which was used without further purification. Yield: 425 mg (60% purity, 94% of theory). LC/MS (method 1): $t_R$ = 0.78 min, MS (ESIpos): *m*/*z* = 317 $[M+H]^+$.

**2-[4-(5-Chloro-2-cyanophenyl)-2-oxopyridin-1(2*H*)-yl]-*N*-[4-(1*H*-tetrazol-5-yl)phenyl]butanamide (racemate 10).**

A solution of HATU (183 mg, 0.48 mmol, 1.2 eq.) in *N,N*-dimethylformamide (4 mL) was added under argon atmosphere at RT to a solution of 2-[4-(5-chloro-2-cyanophenyl)-2-oxopyridin-1(2*H*)-yl]butanoic acid (racemate **114**) (212 mg, 60% purity, 0.40 mmol), 4-(1*H*-tetrazol-5-yl)aniline (71 mg, 0.44 mmol, 1.1 eq.) and *N,N*-diisopropylethylamine (154 μL, 0.88 mmol, 2.2 eq.) in *N,N*-dimethylformamide (6 mL). The reaction mixture was stirred at RT for 1 h and concentrated under reduced pressure. The residue was mixed with iced water, and the solid was filtered. The residue was purified by preparative RP-HPLC (acetonitrile / water gradient) to give **10**. Yield: 143 mg (77% of theory). LC/MS (method 1): $t_R$ = 0.88 min, MS (ESIpos): *m*/*z* = 460 $[M+H]^+$; $^1H$ NMR (400 MHz, DMSO-$d_6$): δ [ppm] = 16.77 (br s, 1H), 10.87 (s, 1H), 8.05 (d, *J* = 8.3 Hz, 1H), 8.01 (d, *J* = 8.8 Hz, 2H), 7.98 (d, *J* = 7.3 Hz, 1H), 7.87-7.82 (m, 3H), 7.77 (dd, *J* = 8.3 Hz, 2.0 Hz, 1H), 6.69 (d, *J* = 2.0 Hz, 1H), 6.57 (dd, *J* = 7.1 Hz, 2.2 Hz, 1H), 5.62 (dd, *J* = 9.8 Hz, 6.1 Hz, 1H), 2.25-2.15 (m, 1H), 2.15-2.04 (m, 1H), 0.92 (t, *J* = 7.2 Hz, 3H).

**Synthesis of racemate 11.**[a]

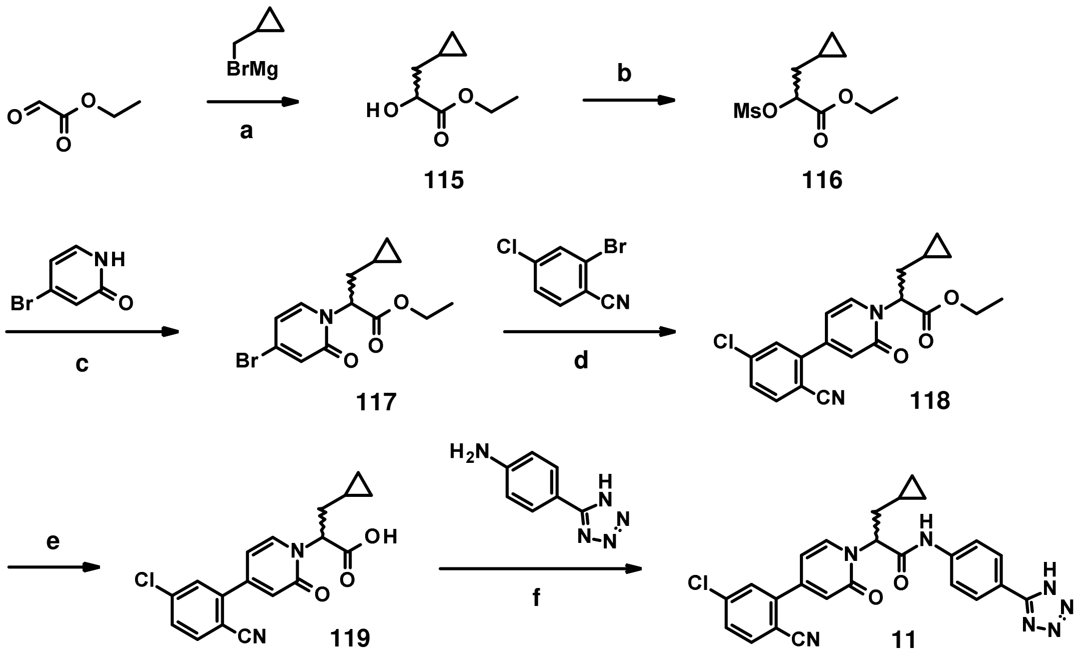

[a]Reagents and conditions: (a) THF, 0 °C → RT, 60%; (b) MsCl, DIEA, DCM, 0 °C → RT, 87%; (c) NaH, LiBr, DMF, 0 °C → 65 °C, 13%; (d) Pd(dppf)$Cl_2$-DCM complex, $K_2CO_3$, 1,4-dioxane, 130 °C/microwave, 93%; (e) LiOH, THF/water, RT, 61%; (f) HATU, DIEA, DMF, RT, 30%.

**Ethyl 3-cyclopropyl-2-hydroxypropanoate (racemate 115).**

Bromo(cyclopropylmethyl)magnesium (7.65 g, 48.00 mmol, 1.8 eq.) was added under argon atmosphere at 0 °C to a mixture of ethyl glyoxylate solution (50% in toluene, 5.29 mL, 26.67 mmol) in tetrahydrofuran (50 mL). The reaction mixture was stirred at RT for 48 h, mixed successively with ethyl acetate, water and Celite®, stirred for 5 min and filtered. After phase separation, the aqueous phase was extracted with ethyl acetate. The combined organic phases were dried over anhydrous sodium sulfate, filtered, concentrated under reduced pressure and dried *in vacuo* to give **115** which was used without further purification. Yield: 2.55 g (60% of theory). GC/MS (method 10): $t_R$ = 2.49 min, MS (EI): $m/z$ = 158 $[M]^+$.

**Ethyl 3-cyclopropyl-2-[(methylsulfonyl)oxy]propanoate (racemate 116).**

Methanesulfonyl chloride (0.45 mL, 5.77 mmol, 1.2 eq.) was added under argon atmosphere at 0 °C to a solution of ethyl 3-cyclopropyl-2-hydroxypropanoate (racemate **115**) (1.90 g, 40% purity, 4.80 mmol) and *N,N*-diisopropylethylamine (2.01 mL, 11.53 mmol, 2.4 eq.) in dichloromethane (100 mL). The reaction mixture was stirred at RT for 2 h and mixed with ice. After phase separation, the organic phase was washed with water and brine, dried over anhydrous sodium sulfate, filtered, concentrated under reduced pressure and dried *in vacuo* to give **116** which was used without further purification. Yield: 1.93 g (51% purity, 87% of theory).

**Ethyl 2-(4-bromo-2-oxopyridin-1(2*H*)-yl)-3-cyclopropylpropanoate (racemate 117).**

Sodium hydride (60% in mineral oil, 270 mg, 6.75 mmol, 1.15 eq.) was added under argon atmosphere at 0 °C to a solution of 4-bromopyridin-2(1*H*)-one (1.02 g, 5.87 mmol) in *N,N*-dimethylformamide (27 mL). The reaction mixture was stirred at 0 °C for 10 min, mixed with lithium bromide (1.17 g, 13.05 mmol, 2.3 eq.), stirred at RT for 15 min, mixed with ethyl 3-cyclopropyl-2-[(methylsulfonyl)oxy]propanoate (racemate **116**) (2.23 g, 56% purity, 5.28 mmol, 0.9 eq.), stirred at 65 °C overnight, cooled to RT and concentrated under reduced pressure. The residue was dissolved in ethyl acetate and extracted with water. The organic phase was dried over anhydrous sodium sulfate, filtered and concentrated under reduced pressure. The residue was purified by RP-HPLC (acetonitrile / water gradient) to give **117**. Yield: 246 mg (13% of theory). $^{1}$H NMR (400 MHz, DMSO-$d_6$): δ [ppm] = 7.67 (d, $J$ = 7.3 Hz, 1H), 6.76 (d, $J$ = 2.1 Hz, 1H), 6.54 (dd, $J$ = 7.3 Hz, 2.2 Hz, 1H), 5.76 (m, 1H), 5.12 (dd, $J$ = 8.7 Hz, 6.7 Hz, 1H), 4.97 (m, 2H), 4.11 (q, $J$ = 7.1 Hz, 2H), 2.21-2.10 (m, 2H), 1.92 (m, 2H), 1.15 (t, $J$ = 7.1 Hz, 3H).

**Ethyl 2-[4-(5-chloro-2-cyanophenyl)-2-oxopyridin-1(2*H*)-yl]-3-cyclopropylpropanoate (racemate 118).**

A mixture of ethyl 2-(4-bromo-2-oxopyridin-1(2*H*)-yl)-3-cyclopropylpropanoate (racemate **117**) (33 mg, 0.10 mmol), (5-chloro-2-cyanophenyl)boronic acid (24 mg, 0.13 mmol, 1.3 eq.), potassium carbonate (43 mg, 0.31 mmol, 3.0 eq.) in 1,4-dioxane (3 mL) was flushed with argon at RT, followed by addition of [1,1'-bis(diphenylphosphino)ferrocene]dichloropalladium-dichloromethane complex (13 mg, 0.02 mmol, 0.15 eq.). The reaction mixture was stirred in the microwave at 130 °C for 13 min, cooled to RT and filtered through Celite®, and the filter residue was washed with 1,4-dioxane. The combined filtrates were concentrated under reduced pressure. The residue was dissolved in ethyl acetate and extracted with water. The organic phase was concentrated under reduced pressure. The residue was purified by flash silica gel chromatography (cyclohexane / ethyl acetate gradient) to give **118**. Yield: 39 mg (91% purity, 93% of theory). LC/MS (method 1): $t_R$ = 1.12 min, MS (ESIpos): $m/z$ = 371 $[M+H]^+$.

**2-[4-(5-Chloro-2-cyanophenyl)-2-oxopyridin-1(2*H*)-yl]-3-cyclopropylpropanoic acid (racemate 119).**

Lithium hydroxide (5 mg, 0.19 mmol, 2.0 eq.) was added at RT to a solution of ethyl 2-[4-(5-chloro-2-cyanophenyl)-2-oxopyridin-1(2*H*)-yl]-3-cyclopropylpropanoate (racemate **118**) (39 mg, 91% purity, 0.10 mmol) in a mixture of tetrahydrofuran and water (3:1, 4 mL). The reaction mixture was stirred at RT for 1h, acidified with aqueous hydrochloric acid solution (1 N) and added to iced water. After addition of ethyl acetate, the organic phase was extracted with water, dried over anhydrous sodium sulfate, filtered, concentrated under reduced pressure and dried *in vacuo* to give **119** which was used without further purification. Yield: 24 mg (83% purity, 61% of theory). LC/MS (method 1): $t_R$ = 0.93 min, MS (ESIpos): $m/z$ = 343 $[M+H]^+$.

**2-[4-(5-Chloro-2-cyanophenyl)-2-oxopyridin-1(2*H*)-yl]-3-cyclopropyl-*N*-[4-(1*H*-tetrazol-5-yl)phenyl]propenamide (racemate 11).**

A solution of HATU (27 mg, 0.07 mmol, 1.2 eq.) in *N,N*-dimethylformamide (1 mL) was added under argon atmosphere at RT to a solution of 2-[4-(5-chloro-2-cyanophenyl)-2-oxopyridin-1(2*H*)-yl]-3-cyclopropylpropanoic acid (racemate **119**) (24 mg, 83% purity, 0.06 mmol), 4-(1*H*-tetrazol-5-yl)aniline (10 mg, 0.06 mmol, 1.1 eq.) and *N,N*-diisopropylethylamine (30 $\mu$L, 0.17 mmol, 3.0 eq.) in *N,N*-dimethylformamide (3 mL). The reaction mixture was stirred at RT overnight and concentrated under reduced pressure. The residue was dissolved in ethyl acetate, washed with water and brine, dried over anhydrous sodium sulfate, filtered and concentrated under reduced pressure. The residue was purified by preparative RP-HPLC (methanol / water gradient) to give **11**. Yield: 9 mg (30% of theory). LC/MS (method 1): $t_R$ = 0.99 min, MS (ESIpos): $m/z$ = 486 $[M+H]^+$; $^1$H NMR (400 MHz, DMSO-$d_6$): δ [ppm] = 10.86 (br s, 1H), 8.05 (d, $J$ = 8.3 Hz, 1H), 8.03-7.96 (m, 3H), 7.88-7.81 (m, 3H), 7.77 (dd, $J$ = 8.3 Hz, 2.1 Hz, 1H), 6.69 (d, $J$ = 2.1 Hz, 1H), 6.57 (dd, $J$ = 7.1 Hz, 2.0 Hz, 1H), 5.90-5.78 (m, 1H), 5.75-5.67 (m, 1H), 5.08-4.97 (m, 2H), 2.29-2.19 (m, 2H), 2.13-1.95 (m, 2H).

**Synthesis of racemate 12.**[a]

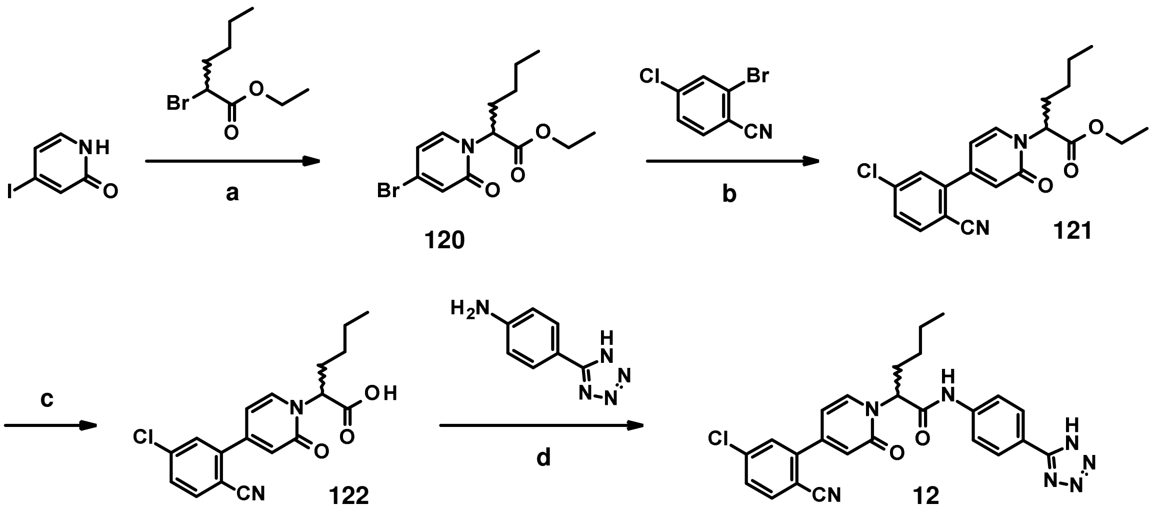


[a]Reagents and conditions: (a) NaH, LiBr, DMF, 0° → 65 °C, 43%; (b) Pd(dppf)$Cl_2$-DCM complex, $K_2CO_3$, 1,4-dioxane, 150 °C/microwave, 58%; (e) LiOH, THF/water, RT, 82%; (f) HATU, DIEA, DMF, RT, 26%.

**Ethyl 2-(4-iodo-2-oxopyridin-1(2*H*)-yl)hexanoate (racemate 120).**

Sodium hydride (60% in mineral oil, 104 mg, 2.60 mmol, 1.15 eq.) was added under argon atmosphere at 0 °C to a solution of 4-iodopyridin-2(1*H*)-one (500 mg, 2.26 mmol) in *N,N*-dimethylformamide (8 mL). The mixture was stirred at 0 °C for 10 min, mixed with lithium bromide (452 mg, 5.20 mmol, 2.3 eq.), stirred at RT for 15 min, mixed with racemic ethyl 2-bromohexanoate (0.58 mL, 3.17 mmol,

1.4 eq.), stirred at 65 °C for 1 h, cooled to RT and concentrated under reduced pressure. The residue was dissolved in ethyl acetate and extracted with water. The organic phase was dried over anhydrous sodium sulfate, filtered and concentrated under reduced pressure. The residue was purified by preparative RP-HPLC (methanol / water gradient) to give **120**. Yield: 352 mg (43% of theory). LC/MS (method 1): $t_R$ = 1.08 min, MS (ESIpos): *m*/*z* = 364 $[M+H]^+$; $^1$H NMR (400 MHz, DMSO-$d_6$): δ [ppm] = 7.45 (d, *J* = 7.3 Hz, 1H), 6.96 (d, *J* = 1.8 Hz, 1H), 6.63 (dd, *J* = 7.3 Hz, 1.8 Hz, 1H), 5.10 (dd, *J* = 9.5 Hz, 6.3 Hz, 1H), 4.10 (q, *J* = 7.0 Hz, 2H), 2.10-1.95 (m, 2H), 1.35-1.1 (m, 3H, partially concealed), 1.15 (t, *J* = 7.0 Hz, 3H), 1.09-0.96 (m, 1H), 0.82 (t, *J* = 7.2 Hz, 3H).

**Ethyl 2-[4-(5-chloro-2-cyanophenyl)-2-oxopyridin-1(2*H*)-yl]hexanoate (racemate 121).**

A mixture of ethyl 2-(4-iodo-2-oxopyridin-1(2*H*)-yl)hexanoate (racemate **120**) (150 mg, 0.41 mmol), (5-chloro-2-cyanophenyl)boronic acid (97 mg, 0.54 mmol, 1.3 eq.) and potassium carbonate (171 mg, 1.24 mmol, 3.0 eq.) was flushed with argon at RT, followed by addition of [1,1'-bis(diphenylphosphino)ferrocene]dichloropalladium-dichloromethane complex (51 mg, 0.06 mmol, 0.15 eq.) and 1,4-dioxane (6 mL). The reaction mixture was stirred in the microwave at 150 °C for 10 min, cooled to RT, combined with the reaction mixture of a previously conducted test campaign (30 mg, 0.08 mmol of racemate **120**), filtered through Celite®, and the filter residue was washed with 1,4-dioxane. The combined filtrates were concentrated under reduced pressure. The residue was dissolved in ethyl acetate and extracted with water. The organic phase was concentrated under reduced pressure. The residue was purified by preparative RP-HPLC (methanol / water gradient) to give **121**. Yield: 114 mg (58% of theory for both campaigns). LC/MS (method 1): $t_R$ = 1.15 min, MS (ESIpos): *m*/*z* = 373 $[M+H]^+$.

**2-[4-(5-Chloro-2-cyanophenyl)-2-oxopyridin-1(2*H*)-yl]hexanoic acid (racemate 122).**

Lithium hydroxide (14 mg, 0.58 mmol, 2.0 eq.) was added at RT to a mixture of ethyl 2-[4-(5-chloro-2-cyanophenyl)-2-oxopyridin-1(2*H*)-yl]hexanoate (racemate **121**) (113 mg, 0.29 mmol) in a mixture of tetrahydrofuran and water (3:1, 6 mL). The reaction mixture was stirred at RT for 1 h, acidified with aqueous hydrochloric acid solution (1 N) and added to iced water. The solid was filtered, washed with water and dried *in vacuo* to give **122** which was used without further purification. Yield: 95 mg (78% purity, 82% of theory). LC/MS (method 1): $t_R$ = 0.98 min, MS (ESIpos): *m*/*z* = 345 $[M+H]^+$.

**2-[4-(5-Chloro-2-cyanophenyl)-2-oxopyridin-1(2*H*)-yl]-*N*-[4-(1*H*-tetrazol-5-yl)phenyl]hexanamide (racemate 12).**

A solution of HATU (98 mg, 0.26 mmol, 1.2 eq.) in *N,N*-dimethylformamide (1 mL) was added under argon atmosphere at RT to a solution of 2-[4-(5-chloro-2-cyanophenyl)-2-oxopyridin-1(2*H*)-yl]hexanoic acid (racemate **122**) (95 mg, 78% purity, 0.22 mmol), 4-(1*H*-tetrazol-5-yl)aniline (42 mg, 0.26 mmol, 1.2 eq.) and *N,N*-diisopropylethylamine (82 µL, 0.47 mmol, 2.2 eq.) in *N,N*-dimethylformamide (3 mL). The reaction mixture was stirred at RT for 2 h and concentrated under

reduced pressure. The residue was dissolved in ethyl acetate and extracted with water. The aqueous phase was dried over anhydrous sodium sulfate, filtered and concentrated under reduced pressure. The residue was purified by preparative RP-HPLC (methanol / water gradient) to give **12**. Yield: 32 mg (30% of theory). LC/MS (method 1): $t_R$ = 1.07 min, MS (ESIpos): *m*/*z* = 488 $[M+H]^+$; $^1H$ NMR (400 MHz, DMSO-$d_6$): δ [ppm] = 10.87 (s, 1H), 8.05 (d, *J* = 8.3 Hz, 1H), 8.03-7.95 (m, 3H), 7.89-7.81 (m, 3H), 7.77 (dd, *J* = 8.3 Hz, 2.0 Hz, 1H), 6.69 (d, *J* = 2.0 Hz, 1H), 6.56 (dd, *J* = 7.2 Hz, 2.0 Hz, 1H), 5.75-5.67 (m, 1H), 2.21-2.03 (m, 2H), 1.41-1.30 (m, 2H), 1.29-1.18 (m, 2H), 0.88 (t, 3H).

**Compounds of Table 2.**

**Synthesis of racemate 13.**[a]

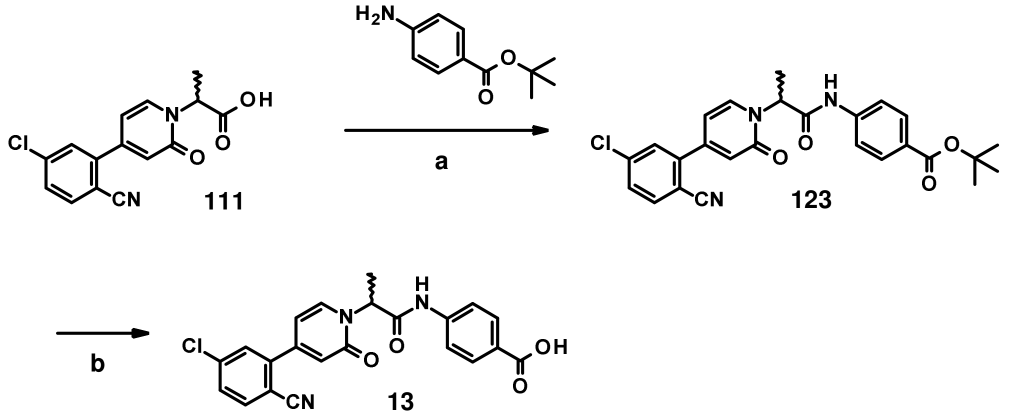


[a]Reagents and conditions: (a) HATU, DIEA, DMF, RT, 43%; (b) TFA, DCM, RT, 48%.

***tert*-Butyl 4-{2-[4-(5-chloro-2-cyanophenyl)-2-oxopyridin-1(2*H*)-yl]propanamido}benzoate (racemate 123).**

A solution of HATU (95 mg, 0.25 mmol, 1.2 eq.) in *N*,*N*-dimethylformamide (2 mL) was added under argon atmosphere at RT to a solution of 2-[4-(5-chloro-2-cyanophenyl)-2-oxopyridin-1(2*H*)-yl]propanoic acid (racemate **111**) (76 mg, 83% purity, 0.21 mmol), *tert*-butyl 4-aminobenzoate (44 mg, 0.23 mmol, 1.1 eq.) and *N*,*N*-diisopropylethylamine (80 μL, 0.46 mmol, 2.2 eq.) in *N*,*N*-dimethylformamide (3 mL). The reaction mixture was stirred at RT for 1 h and concentrated under reduced pressure. The residue was mixed with iced water. The precipitate was filtered, dissolved in dichloromethane and purified by preparative RP-HPLC (acetonitrile / water gradient) to give **123**. Yield: 43 mg (43% of theory). LC/MS (method 1): $t_R$ = 1.20 min, MS (ESIpos): *m*/*z* = 478 $[M+H]^+$.

**4-{2-[4-(5-Chloro-2-cyanophenyl)-2-oxopyridin-1(2*H*)-yl]propanamido}benzoic acid (racemate 13).**

Trifluoroacetic acid (139 μL, 1.80 mmol, 20 eq.) was added at RT to a mixture of *tert*-butyl 4-{2-[4-(5-chloro-2-cyanophenyl)-2-oxopyridin-1(2*H*)-yl]propanamido}benzoate (racemate **123**) (43 mg,

0.09 mmol) in dichloromethane (3 mL). The reaction mixture was stirred at RT for 3 h and concentrated under reduced pressure. The residue was stirred in dichloromethane, and the solid was filtered and dried *in vacuo* to give **13**. Yield: 20 mg (89% purity, 48% of theory). LC/MS (method 1): $t_R$ = 0.88 min, MS (ESIpos): $m/z$ = 422 $[M+H]^+$; $^1H$ NMR (400 MHz, DMSO-$d_6$): δ [ppm] = 12.73 (br s, 1H), 10.74 (s, 1H), 8.04 (d, $J$ = 8.3 Hz, 1H), 7.95 (d, $J$ = 7.3 Hz, 1H), 7.91 (d, $J$ = 8.7 Hz, 2H), 7.82 (d, $J$ = 2.1 Hz, 1H), 7.76 (dd, $J$ = 8.3 Hz, 2.1 Hz, 1H), 7.72 (d, $J$ = 8.8 Hz, 2H), 6.66 (d, $J$ = 2.0 Hz, 1H), 6.56 (dd, $J$ = 7.2 Hz, 2.1 Hz, 1H), 5.58 (q, $J$ = 7.3 Hz, 1H), 1.70 (d, $J$ = 7.3 Hz, 3H).

**Synthesis of racemate 14.**[a]

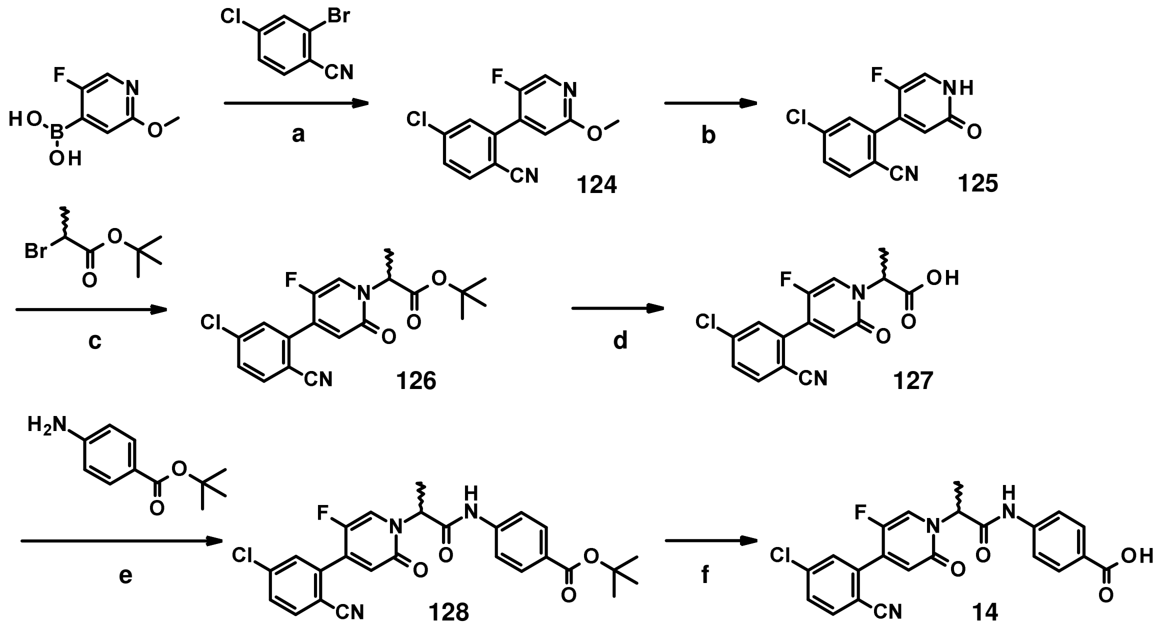


[a]Reagents and conditions: (a) Pd(dppf)$Cl_2$-DCM complex, $K_2CO_3$, 1,4-dioxane, 110 °C, 37%; (b) pyridine hydrochloride, DMF, 100 °C, 66%; (c) $K_2CO_3$, DMF, 100 °C, 25%; (d) TFA, DCM, RT, 100%; (e) HATU, DIEA, DMF, RT, 67%; (f) TFA, DCM, RT, 77%.

**4-Chloro-2-(5-fluoro-2-methoxypyridin-4-yl)benzonitrile (124).**

A mixture of 2-bromo-4-chlorobenzonitrile (295 mg, 1.36 mmol), (5-fluoro-2-methoxypyridin-4-yl)boronic acid (256 mg, 1.50 mmol, 1.1 eq.), potassium carbonate (565 mg, 4.09 mmol, 3.0 eq.) and [1,1'-bis(diphenylphosphino)ferrocene]dichloropalladium-dichloromethane complex (111 mg, 0.14 mmol, 0.1 eq.) was three times evacuated and flushed with argon at RT and mixed with 1,4-dioxane (8 mL). The reaction mixture was stirred at 110 °C (preheated oil bath) for 7 h, cooled to RT and filtered through Celite®, and the filter residue was washed with 1,4-dioxane. The combined filtrates were concentrated under reduced pressure. The residue was mixed with water, and the solid was filtered and dried *in vacuo*. The residue was mixed with *N,N*-dimethylformamide and acetonitrile, and the solid was filtered and dried *in vacuo* to give **124**. Yield: 146 mg (37% of theory). LC/MS (method 1): $t_R$ =

1.10 min, MS (ESIpos): $m/z$ = 263 $[M+H]^+$.

**4-Chloro-2-(5-fluoro-2-oxo-1,2-dihydropyridin-4-yl)benzonitrile (125).**

Pyridine hydrochloride (1.77 g, 15.35 mmol, 20 eq.) was added at RT to a mixture of 4-chloro-2-(5-fluoro-2-methoxypyridin-4-yl)benzonitrile (**124**) (210 mg, 0.77 mmol) in *N,N*-dimethylformamide (6 mL). The reaction mixture was stirred at 100 °C for 30 h, cooled to RT, mixed with additional pyridine hydrochloride (443 mg, 3.84 mmol, 5 eq.), stirred at 100 °C for 2 d, cooled to RT, mixed with additional pyridine hydrochloride (443 mg, 3.84 mmol, 5 eq.), stirred at 100 °C overnight, cooled to RT and concentrated under reduced pressure. The residue was stirred in water, and the solid was filtered, washed with water and dried *in vacuo* (residue 1). The filtrate was extracted with ethyl acetate. The combined organic phases were dried over anhydrous sodium sulfate, filtered, concentrated under reduced pressure and dried *in vacuo* (residue 2). The residue 1 was mixed with *N,N*-dimethylformamide (6 mL) and pyridine hydrochloride (621 mg, 5.37 mmol, 7 eq.), stirred at 100 °C overnight and worked-up accordingly to give residue 3. The residues 2+3 were combined and purified by preparative RP-HPLC (acetonitrile / water gradient) to give **125**. Yield: 126 mg (66% of theory). LC/MS (method 1): $t_R$ = 0.76 min, MS (ESIpos): $m/z$ = 249 $[M+H]^+$.

***tert*-Butyl 2-[4-(5-chloro-2-cyanophenyl)-5-fluoro-2-oxopyridin-1(2*H*)-yl]propanoate (racemate 126).**

A mixture of 4-chloro-2-(5-fluoro-2-oxo-1,2-dihydropyridin-4-yl)benzonitrile (**125**) (126 mg, 0.51 mmol), racemic *tert*-butyl 2-bromopropanoate (111 mg, 0.53 mmol, 1.05 eq.) and potassium carbonate (105 mg, 0.76 mmol, 1.5 eq.) in *N,N*-dimethylformamide (5 mL) was stirred under argon atmosphere at 100 °C for 45 min, cooled to RT and concentrated under reduced pressure. The residue was mixed with water and the aqueous phase extracted with ethyl acetate. The combined organic phases were concentrated under reduced pressure. The residue was purified by preparative RP-HPLC (acetonitrile / water gradient) to give **126**. Yield: 48 mg (25% of theory). LC/MS (method 1): $t_R$ = 1.09 min, MS (ESIpos): $m/z$ = 377 $[M+H]^+$.

**2-[4-(5-Chloro-2-cyanophenyl)-5-fluoro-2-oxopyridin-1(2*H*)-yl]propanoic acid (racemate 127).**

Trifluoroacetic acid (188 µL, 2.44 mmol, 20 eq.) was added at RT to a mixture of *tert*-butyl 2-[4-(5-chloro-2-cyanophenyl)-5-fluoro-2-oxopyridin-1(2*H*)-yl]propanoate (racemate **126**) (46 mg, 0.12 mmol) in dichloromethane (3 mL). The reaction mixture was stirred at RT for 5 h and concentrated under reduced pressure. The residue was coevaporated several times with dichloromethane and dried *in vacuo* to give **127** which was used without further purification. Yield: 54 mg (90% purity, 100% of theory). LC/MS (method 1): $t_R$ = 0.78 min, MS (ESIpos): $m/z$ = 321 $[M+H]^+$.

***tert*-Butyl 4-{2-[4-(5-chloro-2-cyanophenyl)-5-fluoro-2-oxopyridin-1(2*H*)-yl]propanamido}-benzoate (racemate 128).**

A solution of HATU (69 mg, 0.18 mmol, 1.2 eq.) in *N,N*-dimethylformamide (1 mL) was added under argon atmosphere at RT to a solution of 2-[4-(5-chloro-2-cyanophenyl)-5-fluoro-2-oxopyridin-1(2*H*)-yl]propanoic acid (racemate **127**) (54 mg, 90% purity, 0.15 mmol), *tert*-butyl 4-aminobenzoate (32 mg, 0.17 mmol, 1.1 eq.) and *N,N*-diisopropylethylamine (58 µL, 0.33 mmol, 2.2 eq.) in *N,N*-dimethylformamide (2 mL). The reaction mixture was stirred at RT for 4 h and purified without further work-up by preparative RP-HPLC (acetonitrile / water gradient) to give **128**. Yield: 51 mg (67% of theory). LC/MS (method 1): $t_R$ = 1.23 min, MS (ESIpos): *m*/*z* = 496 $[M+H]^+$.

**4-{2-[4-(5-Chloro-2-cyanophenyl)-5-fluoro-2-oxopyridin-1(2*H*)-yl]propanamido}benzoic acid (racemate 14).**

Trifluoroacetic acid (163 µL, 2.12 mmol, 20 eq.) was added at RT to a mixture of *tert*-butyl 4-{2-[4-(5-chloro-2-cyanophenyl)-5-fluoro-2-oxopyridin-1(2*H*)-yl]propanamido}benzoate (racemate **128**) (53 mg, 0.11 mmol) in dichloromethane (3 mL). The reaction mixture was stirred at RT for 3 h and concentrated under reduced pressure. The residue was coevaporated several times with dichloromethane, dried *in vacuo* and purified by preparative RP-HPLC (acetonitrile / water gradient) to give **14**. Yield: 36 mg (77% of theory). LC/MS (method 1): $t_R$ = 0.90 min, MS (ESIpos): *m*/*z* = 440 $[M+H]^+$; $^1$H NMR (400 MHz, DMSO-$d_6$): δ [ppm] = 12.78 (br s, 1H), 10.77 (s, 1H), 8.24 (d, *J* = 6.5 Hz, 1H), 8.09 (d, *J* = 8.3 Hz, 1H), 7.91 (d, *J* = 8.7 Hz, 2H), 7.88 (d, *J* = 1.8 Hz, 1H), 7.82 (dd, *J* = 8.3 Hz, 2.1 Hz, 1H), 7.72 (d, *J* = 8.8 Hz, 2H), 6.67 (d, *J* = 7.3 Hz, 1H), 5.55 (q, *J* = 7.3 Hz, 1H), 1.72 (d, *J* = 7.3 Hz, 3H).

**Synthesis of racemate 15.**[a]

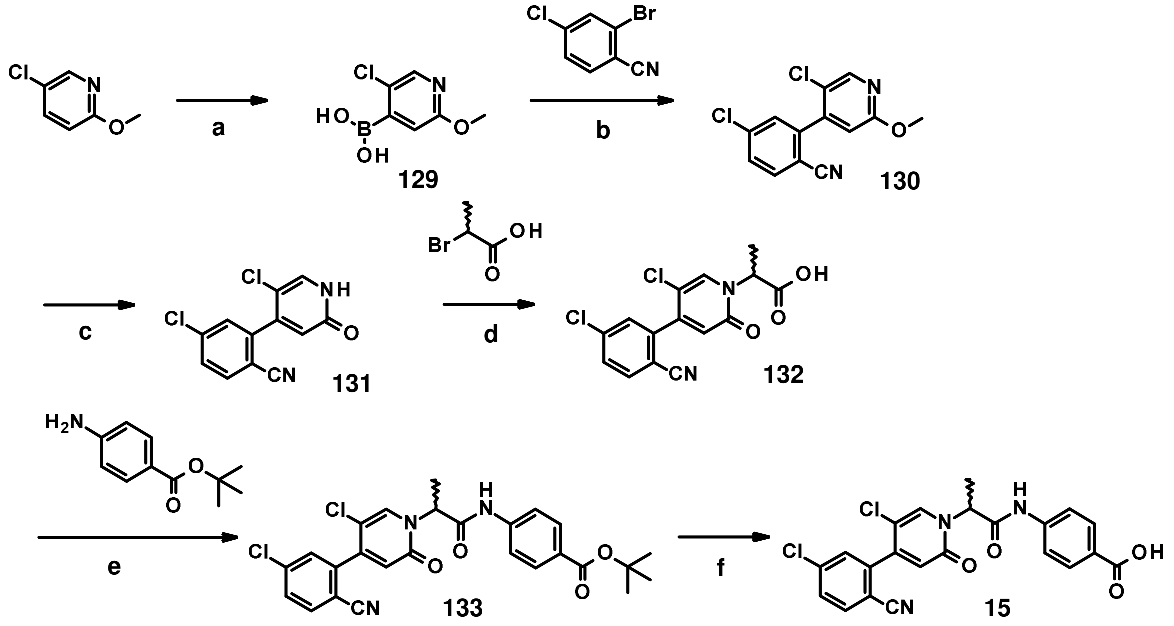


[a]Reagents and conditions: (a) i) LDA, ii) $B(OiPr)_2$, iii) aq. HCl, THF, -78 °C → RT, 73%; (b) $Pd(dppf)Cl_2$-DCM complex, $K_2CO_3$, 1,4-dioxane, 110 °C, 52%; (c) pyridine hydrochloride, DMF, 100 °C, 76%; (d) $Mg(OtBu)_2$, KOtBu, THF, 0 °C → 45 °C, 100%; (e) HATU, DIEA, DMF, RT, 85%; (f) TFA, DCM, RT, 67%.

**(5-Chloro-2-methoxypyridin-4-yl)boronic acid (129).**

Lithium diisopropylamide solution (2 M in tetrahydrofuran, 41.79 mL, 83.58 mmol, 1.2 eq.) was added under argon atmosphere at -78 °C to a solution of 5-chloro-2-methoxypyridine (10.00 g, 69.65 mmol) in tetrahydrofuran (225 mL). The reaction mixture was stirred at -78 °C for 4 h and quickly mixed with triisopropyl borate (32.63 mL, 141.39 mmol, 2.03 eq.). The reaction mixture was maintained at -78 °C for further 3 h and then slowly (!) thawed to RT overnight. The reaction mixture was cooled to -78 °C, mixed with additional lithium diisopropylamide solution (2 M in tetrahydrofuran, 20.90 mL, 41.79 mmol, 0.6 eq.), stirred at -78 °C for 2 h, mixed quickly with additional triisopropyl borate (16.07 mL, 69.65 mmol, 1.0 eq.), maintained at -78 °C for further 2 h and slowly thawed to RT. The mixture was added to water. Tetrahydrofuran was removed under reduced pressure. The aqueous phase was washed with ethyl acetate, acidified with aqueous hydrochloric acid solution (2 N), and the forming precipitate was filtered. The filtrate was extracted with ethyl acetate. The combined organic phases were dried over anhydrous sodium sulfate, filtered, concentrated under reduced pressure and dried *in vacuo* to give **129**. Yield: 10.44 g (91% purity, 73% of theory). LC/MS (method 1): $t_R$ = 0.50 min, MS (ESIpos): $m/z$ = 188 $[M+H]^+$; $^1H$ NMR (400 MHz, DMSO-$d_6$): δ [ppm] = 8.64 (br s, 2H), 8.12 (s, 1H), 6.81 (s, 1H), 3.82 (s, 3H).

**4-Chloro-2-(5-chloro-2-methoxypyridin-4-yl)benzonitrile (130).**

A mixture of (5-chloro-2-methoxypyridin-4-yl)boronic acid (**129**) (5.36 g, 91% purity, 26.03 mmol, 1.1 eq.), 2-bromo-4-chlorobenzonitrile (5.12 g, 23.66 mmol), potassium carbonate (9.81 g, 70.99 mmol, 3.0 eq.) and [1,1'-bis(diphenylphosphino)ferrocene]dichloropalladium-dichloromethane complex (1.93 g, 2.37 mmol, 0.1 eq.) was three times evacuated and flushed with argon at RT and mixed with 1,4-dioxane (145 mL). The reaction mixture was stirred at 110 °C (preheated oil bath) overnight, cooled to RT and filtered through Celite®, and the filter residue was washed with 1,4-dioxane. The combined filtrates were concentrated under reduced pressure. The residue was mixed with water and extracted with ethyl acetate. The organic phase was washed with water and brine, dried over anhydrous sodium sulfate, filtered, concentrated under reduced pressure and dried *in vacuo*. The residue was purified by flash silica gel chromatography (cyclohexane / dichloromethane gradient) to give **130**. Yield: 4.11 g (91% purity, 52% of theory). LC/MS (method 1): $t_R$ = 1.17 min, MS (ESIpos): $m/z$ = 279 $[M+H]^+$.

**4-Chloro-2-(5-chloro-2-oxo-1,2-dihydropyridin-4-yl)benzonitrile (131).**

Pyridine hydrochloride (48.82 g, 422.47 mmol, 20 eq.) was added at RT to a mixture of 4-chloro-2-(5-chloro-2-methoxypyridin-4-yl)benzonitrile (**130**) (6.34 g, 93% purity, 21.12 mmol) in *N,N*-dimethylformamide (110 mL). The reaction mixture was stirred at 100 °C for 20 h, cooled to RT and concentrated under reduced pressure. After addition of ethyl acetate / water and phase separation, the organic phase was washed with water. The combined aqueous phases were extracted with ethyl acetate. The combined organic phases were dried over anhydrous sodium sulfate, filtered, concentrated under reduced pressure and dried *in vacuo*. The residue was dissolved in *N,N*-dimethylformamide (100 mL), mixed with pyridine hydrochloride (24.41 g, 211.24 mmol, 10 eq.), stirred at 100 °C for 64 h, cooled to RT and concentrated under reduced pressure. After addition of ethyl acetate / water and phase separation, the organic phase was washed with water. The combined aqueous phases were extracted with ethyl acetate. The combined organic phases were dried over anhydrous sodium sulfate, filtered, concentrated under reduced pressure and dried *in vacuo*. The residue was stirred in dichloromethane / methanol (60:1), and the solid was filtered, washed with dichloromethane / methanol (60:1) and dried *in vacuo* to give a first batch of **131**. Yield: 3.74 g (67% of theory). The filtrate was purified by flash silica gel chromatography (dichloromethane / methanol gradient) to give a second batch of **131**. Yield: 490 mg (9% of theory). LC/MS (method 1): $t_R$ = 0.82 min, MS (ESIpos): $m/z$ = 265 $[M+H]^+$.

**2-[5-Chloro-4-(5-chloro-2-cyanophenyl)-2-oxopyridin-1(2*H*)-yl]propanoic acid (racemate 132).**

A mixture of 4-chloro-2-(5-chloro-2-oxo-1,2-dihydropyridin-4-yl)benzonitrile (**131**) (910 mg, 57% purity, 1.96 mmol), magnesium di-*tert*-butoxide (667 mg, 3.91 mmol, 2.0 eq.) and potassium *tert*-butylate (231 mg, 2.05 mmol, 1.05 eq.) in tetrahydrofuran (20 mL) was stirred under argon atmosphere at RT for 10 min and cooled with an ice bath, followed by addition of racemic 2-bromopropanoic acid (264 $\mu$L, 2.94 mmol, 1.5 eq.). The reaction mixture was stirred at RT for 2.5 h and at 45 °C for 64 h,

acidified to pH 1 with aqueous hydrochloric acid solution (6 N) and mixed with water. The aqueous phase was extracted with ethyl acetate. The combined organic phases were dried over anhydrous sodium sulfate, filtered, concentrated under reduced pressure and dried *in vacuo* to give **132** which was used without further purification. Yield: 1.06 g (78% purity, 100% of theory). LC/MS (method 1): $t_R$ = 0.86 min, MS (ESIpos): *m*/*z* = 337 $[M+H]^+$.

***tert*-Butyl 4-{2-[5-chloro-4-(5-chloro-2-cyanophenyl)-2-oxopyridin-1(2*H*)-yl]propanamido}-benzoate (racemate 133).**

A solution of HATU (170 mg, 0.45 mmol, 1.2 eq.) in *N,N*-dimethylformamide (1 mL) was added under argon atmosphere at RT to a solution of 2-[5-chloro-4-(5-chloro-2-cyanophenyl)-2-oxopyridin-1(2*H*)-yl]propanoic acid (racemate **132**) (135 mg, 93% purity, 0.37 mmol), *tert*-butyl 4-aminobenzoate (79 mg, 0.41 mmol, 1.1 eq.) and *N,N*-diisopropylethylamine (143 μL, 0.82 mmol, 2.2 eq.) in *N,N*-dimethylformamide (3 mL). The reaction mixture was stirred at RT for 5 h and concentrated under reduced pressure. After addition of ethyl acetate / water and phase separation, the aqueous phase was extracted with ethyl acetate. The combined organic phases were washed with brine, dried over anhydrous sodium sulfate, filtered, concentrated under reduced pressure and dried *in vacuo* to give **133** which was used without further purification. Yield: 281 mg (58% purity, 85% of theory). LC/MS (method 2): $t_R$ = 2.69 min, MS (ESIpos): *m*/*z* = 512 $[M+H]^+$.

**4-{2-[5-Chloro-4-(5-chloro-2-cyanophenyl)-2-oxopyridin-1(2*H*)-yl]propanamido}benzoic acid (racemate 15).**

Trifluoroacetic acid (490 μL, 6.36 mmol, 20 eq.) was added under argon atmosphere at RT to a mixture of *tert*-butyl 4-{2-[5-chloro-4-(5-chloro-2-cyanophenyl)-2-oxopyridin-1(2*H*)-yl]propanamido}-benzoate (racemate **133**) (281 mg, 58% purity, 0.32 mmol) in dichloromethane (5 mL). The reaction mixture was stirred at RT for 1 h and concentrated under reduced pressure. The residue was coevaporated several times with dichloromethane, dried *in vacuo* and purified by preparative RP-HPLC (acetonitrile / water gradient) to give **15**. Yield: 97 mg (67% of theory). LC/MS (method 1): $t_R$ = 0.96 min, MS (ESIpos): *m*/*z* = 456 $[M+H]^+$; $^1H$ NMR (400 MHz, DMSO-$d_6$): δ [ppm] = 12.78 (s, 1H), 10.77 (s, 1H), 8.19 (s, 1H), 8.08 (d, *J* = 8.7 Hz, 1H), 7.92 (d, *J* = 8.7 Hz, 2H), 7.80 (d, *J* = 8.7 Hz, 2H), 7.73 (d, *J* = 8.8 Hz, 2H), 6.68 (s, 1H), 5.56 (q, *J* = 7.3 Hz, 1H), 1.74 (d, *J* = 7.3 Hz, 3H).

**Synthesis of racemate 16.**[a]

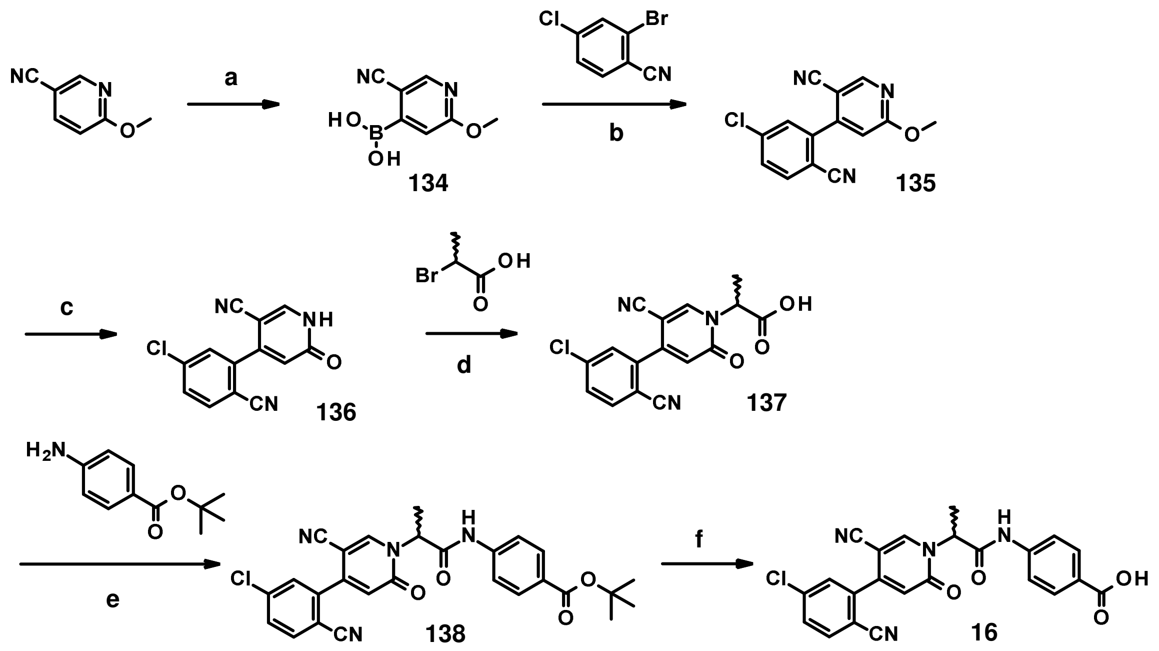

[a]Reagents and conditions: (a) i) LDA, ii) $B(OiPr)_2$, iii) aq. HCl, THF, -78 °C → RT, 70%; (b) $Pd(dppf)Cl_2$-DCM complex, $K_2CO_3$, 1,4-dioxane, 110 °C, 46%; (c) pyridine hydrochloride, DMF, 100 °C, 77%; (d) $Mg(OtBu)_2$, KOtBu, THF, 0 °C → 45 °C, 56%; (e) HATU, DIEA, DMF, RT, 30%; (f) TFA, DCM, RT, 100%.

**(5-Cyano-2-methoxypyridin-4-yl)boronic acid (134).**

Lithium diisopropylamide solution (2 M in tetrahydrofuran, 44.73 mL, 89.46 mmol, 1.2 eq.) was added under argon atmosphere at -78 °C to a solution of 6-methoxynicotinonitrile (10.00 g, 74.55 mmol) in tetrahydrofuran (300 mL). The mixture was stirred at -78 °C for 4 h and quickly mixed with triisopropyl borate (34.92 mL, 151.34 mmol, 2.03 eq.). The reaction mixture was maintained at -78 °C for further 3 h and then slowly (!) thawed to RT overnight. The mixture was mixed with water. Tetrahydrofuran was removed under reduced pressure. The aqueous phase was washed with ethyl acetate, acidified with aqueous hydrochloric acid solution (2 N) and extracted with ethyl acetate. The combined organic phases were dried over anhydrous sodium sulfate, filtered, concentrated under reduced pressure and dried *in vacuo* to give **134** which was used without further purification. Yield: 10.47 g (89% purity, 70% of theory). LC/MS (method 1): $t_R$ = 0.51 min, MS (ESIpos): $m/z$ = 179 $[M+H]^+$.

**4-(5-Chloro-2-cyanophenyl)-6-methoxynicotinonitrile (135).**

A mixture of (5-cyano-2-methoxypyridin-4-yl)boronic acid (**134**) (600 mg, 89% purity, 3.00 mmol), 2-bromo-4-chlorobenzonitrile (649 mg, 3.00 mmol, 1.0 eq.), potassium carbonate (1.24 g, 9.00 mmol, 3.0 eq.) and [1,1'-bis(diphenylphosphino)ferrocene]dichloropalladium-dichloromethane complex (245 mg, 0.30 mmol, 0.1 eq.) was three times evacuated and flushed with argon at RT and mixed with

1,4-dioxane (10 mL). The reaction mixture was stirred at 110 °C (preheated oil bath) overnight, cooled to RT and filtered through Celite®, and the filter residue was washed with 1,4-dioxane. The combined filtrates were concentrated under reduced pressure. The residue was stirred in water, and the solid was filtered and dried *in vacuo*. Furthermore, the residue was stirred in a mixture of cyclohexane / ethyl acetate (7:3), and the solid was filtered and dried *in vacuo* to give **135**. Yield: 399 mg (94% purity, 46% of theory). LC/MS (method 1): $t_R$ = 1.02 min, MS (ESIpos): *m*/*z* = 270 $[M+H]^+$.

**4-(5-Chloro-2-cyanophenyl)-6-oxo-1,6-dihydropyridine-3-carbonitrile (136).**

Pyridine hydrochloride (3.34 g, 28.86 mmol, 20 eq.) was added at RT to a mixture of 4-(5-chloro-2-cyanophenyl)-6-methoxynicotinonitrile (**135**) (414 mg, 94% purity, 1.44 mmol) in *N*,*N*-dimethylformamide (15 mL). The reaction mixture was stirred at 100 °C overnight, cooled to RT and concentrated under reduced pressure. After addition of ethyl acetate / water and phase separation, the aqueous phase was extracted with ethyl acetate. The combined organic phases were dried over anhydrous sodium sulfate, filtered, concentrated under reduced pressure and dried *in vacuo*. The residue was dissolved in *N*,*N*-dimethylformamide (10 mL), mixed with pyridine hydrochloride (1.67 g, 14.43 mmol, 10 eq.), stirred at 100 °C overnight, cooled to RT and concentrated under reduced pressure. The residue was stirred in water, and the solid was filtered, washed with water and dried *in vacuo* to give **136**. Yield: 312 mg (91% purity, 77% of theory). LC/MS (method 1): $t_R$ = 0.71 min, MS (ESIpos): *m*/*z* = 256 $[M+H]^+$.

**2-[4-(5-Chloro-2-cyanophenyl)-5-cyano-2-oxopyridin-1(2*H*)-yl]propanoic acid (racemate 137).**

A mixture of 4-(5-chloro-2-cyanophenyl)-6-oxo-1,6-dihydropyridine-3-carbonitrile (**136**) (312 mg, 91% purity, 1.11 mmol), magnesium di-*tert*-butoxide (379 mg, 2.22 mmol, 2.0 eq.) and potassium *tert*-butylate (187 mg, 1.67 mmol, 1.5 eq.) in tetrahydrofuran (15 mL) was stirred under argon atmosphere at RT for 10 min and cooled with an ice bath, followed by addition of racemic 2-bromopropanoic acid (150 $\mu$L, 1.67 mmol, 1.5 eq.). The reaction mixture was stirred at RT for 2.5 h and at 45 °C overnight, acidified to pH 1 with aqueous hydrochloric acid solution (6 N) and mixed with water. The aqueous phase was extracted with ethyl acetate. The combined organic phases were dried over anhydrous sodium sulfate, filtered, concentrated under reduced pressure and dried *in vacuo* to give **137** which was used without further purification. Yield: 240 mg (85% purity, 56% of theory). LC/MS (method 1): $t_R$ = 0.78 min, MS (ESIpos): *m*/*z* = 328 $[M+H]^+$.

***tert*-Butyl 4-{2-[4-(5-chloro-2-cyanophenyl)-5-cyano-2-oxopyridin-1(2*H*)-yl]propanamido}-benzoate (racemate 138).**

A solution of HATU (284 mg, 0.75 mmol, 1.2 eq.) in *N*,*N*-dimethylformamide (2 mL) was added under argon atmosphere at RT to a solution of 2-[4-(5-chloro-2-cyanophenyl)-5-cyano-2-oxopyridin-1(2*H*)-yl]propanoic acid (racemate **137**) (240 mg, 85% purity, 0.62 mmol), *tert*-butyl 4-aminobenzoate (132 mg, 0.69 mmol, 1.1 eq.) and *N*,*N*-diisopropylethylamine (239 $\mu$L, 1.37 mmol, 2.2 eq.) in *N*,*N*-

dimethylformamide (4 mL). The reaction mixture was stirred at RT for 2 d, mixed with additional solution of HATU (142 mg, 0.37 mmol, 0.6 eq.) in *N,N*-dimethylformamide (1 mL), stirred for 30 min, mixed with additional solution of HATU (95 mg, 0.25 mmol, 0.4 eq.) in *N,N*-dimethylformamide (1 mL), stirred for 30 min and concentrated under reduced pressure. After addition of ethyl acetate / water and phase separation, the aqueous phase was extracted with ethyl acetate. The combined organic phases were dried over anhydrous sodium sulfate, filtered, concentrated under reduced pressure and dried *in vacuo*. The residue was purified by RP-HPLC chromatography (acetonitrile / water gradient) to give **138**. Yield: 93 mg (30% of theory). LC/MS (method 1): $t_R$ = 1.17 min, MS (ESIneg): *m*/*z* = 501 $[M-H]^-$.

**4-{2-[4-(5-Chloro-2-cyanophenyl)-5-cyano-2-oxopyridin-1(2*H*)-yl]propanamido}benzoic acid (racemate 16).**

Trifluoroacetic acid (285 $\mu$L, 3.70 mmol, 20 eq.) was added under argon atmosphere at RT to a mixture of *tert*-butyl 4-{2-[4-(5-chloro-2-cyanophenyl)-5-cyano-2-oxopyridin-1(2*H*)-yl]propanamido}benzoate (racemate **138**) (93 mg, 0.19 mmol) in dichloromethane (3 mL). The reaction mixture was stirred at RT for 3.5 h and concentrated under reduced pressure. The residue was coevaporated several times with dichloromethane, dried *in vacuo* and purified by preparative RP-HPLC (acetonitrile / water gradient) to give **16**. Yield: 91 mg (100% of theory). LC/MS (method 1): $t_R$ = 0.90 min, MS (ESIpos): *m*/*z* = 447 $[M+H]^+$; $^1$H NMR (400 MHz, DMSO-$d_6$): δ [ppm] = 12.80 (br s, 1H), 10.83 (s, 1H), 8.87 (s, 1H), 8.13 (d, *J* = 8.4 Hz, 1H), 7.95-7.89 (m, 3H), 7.86 (dd, *J* = 8.4 Hz, 2.0 Hz, 1H), 7.72 (d, *J* = 8.7 Hz, 2H), 6.78 (s, 1H), 5.57 (q, *J* = 7.3 Hz, 1H), 1.78 (d, *J* = 7.3 Hz, 3H).

**Synthesis of racemate 17.**[a]

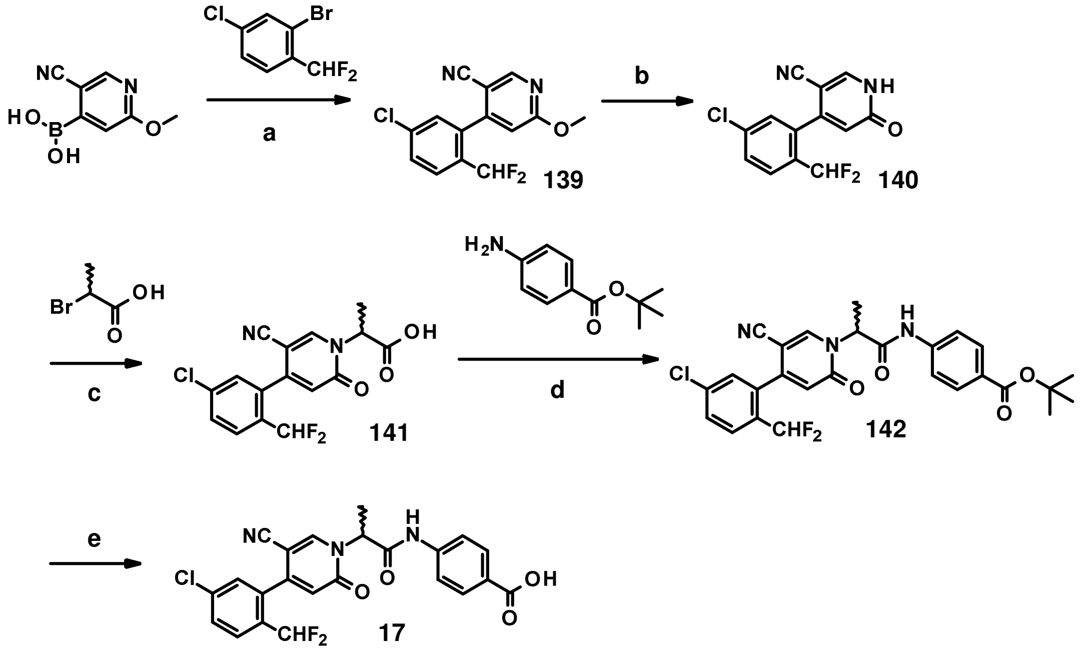

[a]Reagents and conditions: (a) precatalyst Pd-XPhos G2, aq. $K_3PO_4$, THF, 60 °C, 11%; (b) pyridine hydrochloride, DMF, 100 °C, 99%; (c) $Mg(OtBu)_2$, KOtBu, THF, 0 °C → 45 °C, 95%; (d) HATU, DIEA, DMF, RT, 40%; (e) TFA, DCM, RT, 70%.

**4-[5-Chloro-2-(difluoromethyl)phenyl]-6-methoxynicotinonitrile (139).**

A mixture of 2-bromo-4-chloro-1-(difluoromethyl)benzene (724 mg, 3.00 mmol), (5-cyano-2-methoxypyridin-4-yl)boronic acid (600 mg, 89% purity, 3.00 mmol, 1.0 eq.) and precatalyst Pd-XPhos G2 (CAS-RN 1310548-14-5) (71mg, 0.09 mmol, 0.03 eq.) was three times evacuated and flushed with argon at RT, before tetrahydrofuran (12.5 mL) and aqueous potassium phosphate solution (25 mL) were added at RT. The reaction mixture was stirred at 60 °C for 13.5 h, diluted with water and ethyl acetate and extracted with ethyl acetate. The combined organic phases were washed with brine, dried over anhydrous sodium sulfate, filtered, concentrated under reduced pressure and dried *in vacuo*. The residue was purified by RP-HPLC chromatography (acetonitrile / water gradient) to give **139**. Yield: 143 mg (65% purity, 11% of theory). LC/MS (method 1): $t_R$ = 1.15 min, MS (ESIpos): $m/z$ = 295 $[M+H]^+$.

**4-[5-Chloro-2-(difluoromethyl)phenyl]-6-oxo-1,6-dihydropyridine-3-carbonitrile (140).**

Pyridine hydrochloride (729 mg, 6.31 mmol, 20 eq.) was added at RT to a mixture of 4-[5-chloro-2-(difluoromethyl)phenyl]-6-methoxynicotinonitrile (**139**) (143 mg, 65% purity, 0.32 mmol) in *N,N*-dimethylformamide (5 mL). The reaction mixture was stirred at 100 °C overnight, cooled to RT and concentrated under reduced pressure. The residue was stirred in water, and the solid was filtered, washed with water and dried *in vacuo*. The aqueous phase was extracted with ethyl acetate. The combined organic phases were dried over anhydrous sodium sulfate, filtered, concentrated under reduced pressure

and dried *in vacuo*. Both residues were combined, dissolved in *N,N*-dimethylformamide (5 mL) and mixed at RT with additional pyridine hydrochloride (292 mg, 2.52 mmol, 8 eq.). The reaction mixture was stirred at 100 °C overnight, cooled to RT and concentrated under reduced pressure. After addition of ethyl acetate / water and phase separation, the aqueous phase was extracted with ethyl acetate. The combined organic phases were dried over anhydrous sodium sulfate, filtered and concentrated under reduced pressure. The residue was purified by RP-HPLC chromatography (acetonitrile / water gradient) to give **140**. Yield: 88 mg (99% of theory). LC/MS (method 1): $t_R$ = 0.82 min, MS (ESIpos): *m*/*z* = 281 $[M+H]^+$.

**2-{4-[5-Chloro-2-(difluoromethyl)phenyl]-5-cyano-2-oxopyridin-1(2*H*)-yl}propanoic acid (racemate 141).**

A mixture of 4-[5-chloro-2-(difluoromethyl)phenyl]-6-oxo-1,6-dihydropyridine-3-carbonitrile (**140**) (88 mg, 0.31 mmol), magnesium di-*tert*-butoxide (107 mg, 0.63 mmol, 2.0 eq.) and potassium *tert*-butylate (53 mg, 0.47 mmol, 1.5 eq.) in tetrahydrofuran (4 mL) was stirred under argon atmosphere at RT for 10 min and cooled with an ice bath, followed by addition of racemic 2-bromopropanoic acid (42 $\mu$L, 0.47 mmol, 1.5 eq.). The reaction mixture was stirred at RT for 2.5 h and at 45 °C overnight and concentrated under reduced pressure. After addition of ethyl acetate / water and phase separation, the aqueous phase was extracted with ethyl acetate. The combined organic phases were dried over anhydrous sodium sulfate, filtered, concentrated under reduced pressure and dried *in vacuo* to give a first batch of **141**. Yield: 32 mg (77% purity, 22% of theory). The aqueous phase was acidified with aqueous hydrochloric acid solution (2 N) and extracted with ethyl acetate. The combined organic phases were dried over anhydrous sodium sulfate, filtered, concentrated under reduced pressure and dried *in vacuo* to give a second batch of **141** which was used without further purification. Yield: 88 mg (92% purity, 73% of theory). LC/MS (method 1): $t_R$ = 0.87 min, MS (ESIpos): *m*/*z* = 353 $[M+H]^+$.

***tert*-Butyl 4-(2-{4-[5-chloro-2-(difluoromethyl)phenyl]-5-cyano-2-oxopyridin-1(2*H*)-yl}propan-amido)benzoate (racemate 142).**

A solution of HATU (113 mg, 0.30 mmol, 1.3 eq.) in *N,N*-dimethylformamide (1 mL) was added under argon atmosphere at RT to a solution of 2-{4-[5-chloro-2-(difluoromethyl)phenyl]-5-cyano-2-oxopyridin-1(2*H*)-yl}propanoic acid (racemate **141**) (88 mg, 92% purity, 0.23 mmol), *tert*-butyl 4-aminobenzoate (49 mg, 0.25 mmol, 1.1 eq.) and *N,N*-diisopropylethylamine (88 $\mu$L, 0.51 mmol, 2.2 eq.) in *N,N*-dimethylformamide (3 mL). The reaction mixture was stirred at RT overnight, mixed with additional HATU (44 mg, 0.12 mmol, 0.5 eq.) and *tert*-butyl 4-aminobenzoate (44 mg, 0.23 mmol, 1.0 eq.), stirred at RT overnight and concentrated under reduced pressure. After addition of ethyl acetate / water and phase separation, the aqueous phase was extracted with ethyl acetate. The combined organic phases were dried over anhydrous sodium sulfate, filtered, concentrated under reduced pressure and dried *in vacuo*. The residue was purified RP-HPLC chromatography (acetonitrile / water gradient)

to give **142**. Yield: 48 mg (40% of theory). LC/MS (method 1): $t_R$ = 1.24 min, MS (ESIpos): *m*/*z* = 528 $[M+H]^+$.

**4-(2-{4-[5-Chloro-2-(difluoromethyl)phenyl]-5-cyano-2-oxopyridin-1(2*H*)-yl}propanamido)benzoic acid (racemate 17).**

Trifluoroacetic acid (140 µL, 1.82 mmol, 20 eq.) was added under argon atmosphere at RT to a mixture of *tert*-butyl 4-(2-{4-[5-chloro-2-(difluoromethyl)phenyl]-5-cyano-2-oxopyridin-1(2*H*)-yl}propanamido)benzoate (racemate **142**) (48 mg, 0.09 mmol) in dichloromethane (4 mL). The reaction mixture was stirred at RT for 2 h and concentrated under reduced pressure. The residue was coevaporated several times with dichloromethane, dried *in vacuo* and purified by preparative RP-HPLC (acetonitrile / water gradient) to give **17**. Yield: 30 mg (70% of theory). LC/MS (method 1): $t_R$ = 0.96 min, MS (ESIpos): *m*/*z* = 472 $[M+H]^+$; $^1H$ NMR (400 MHz, DMSO-$d_6$): δ [ppm] = 12.80 (s, 1H), 10.80 (s, 1H), 8.79 (s, 1H), 7.92 (d, *J* = 8.7 Hz, 2H), 7.84-7.58 (m, 5H), 7.12-6.85 (br t, 1H), 6.54 (s, 1H), 5.58 (q, *J* = 7.3 Hz, 1H), 1.75 (d, *J* = 7.2 Hz, 3H).

**Synthesis of racemate 18.**[a]

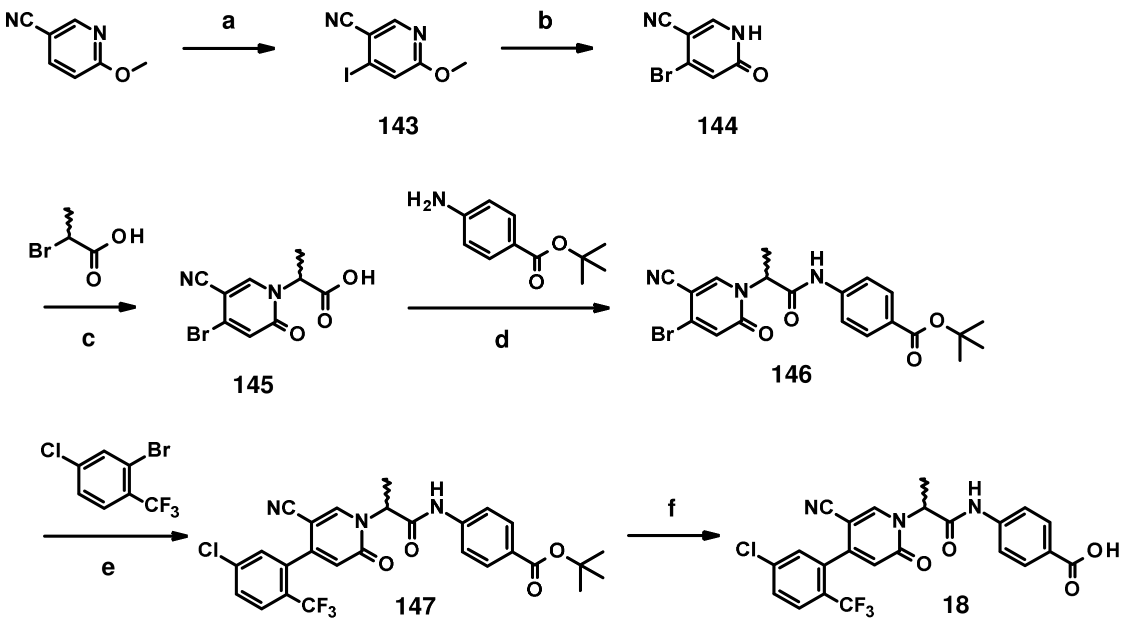


[a]Reagents and conditions: (a) LDA, $I_2$, THF, -78 °C, 48%; (b) pyridine hydrobromide, DMF, 100 °C, 49%; (c) $Mg(OtBu)_2$, KOtBu, THF, 0 °C → 45 °C, 51%; (d) HATU, DIEA, DMF, RT, 57%; (e) $Pd(dppf)Cl_2$-DCM complex, $K_2CO_3$, 1,4-dioxane, 110 °C, 55%; (f) TFA, DCM, RT, 79%.

**4-Iodo-6-methoxynicotinonitrile (143).**

Lithium diisopropylamide solution (2 M in tetrahydrofuran, 19.38 mL, 38.77 mmol, 1.3 eq.) was added under argon atmosphere at -78 °C to a solution of 6-methoxynicotinonitrile (4.00 g, 29.82 mmol) in

tetrahydrofuran (120 mL). The reaction mixture was stirred at -78 °C for 1 h, mixed at -78 °C with a solution of iodine (9.08 g, 35.78 mmol, 1.2 eq.) in tetrahydrofuran (20 mL), stirred at -78 °C for 1 h and carefully added to saturated aqueous ammonium chloride solution. The mixture was extracted with ethyl acetate. The combined organic phases were dried over anhydrous magnesium sulfate, filtered, concentrated under reduced pressure and dried *in vacuo*. The residue was mixed with dichloromethane (20 mL), and the solid was filtered and discarded. The combined filtrates were concentrated under reduced pressure. The residue was purified by flash silica gel chromatography (cyclohexane / ethyl acetate gradient) to give **143**. Yield: 4.02 g (92% purity, 48% of theory). GC/MS (method 10): $t_R$ = 5.08 min, MS (EI): *m*/*z* = 260 $[M]^+$; $^1$H NMR (400 MHz, DMSO-$d_6$): δ [ppm] = 8.63 (s, 1H), 7.61 (s, 1H), 3.92 (s, 1H).

**4-Bromo-6-oxo-1,6-dihydropyridine-3-carbonitrile (144).**

Pyridine hydrobromide (50.03 g, 312.67 mmol, 20 eq.) was added at RT to a mixture of 4-iodo-6-methoxynicotinonitrile (**143**) (4.52 g, 90% purity, 15.63 mmol) in *N*,*N*-dimethylformamide (135 mL). The reaction mixture was stirred at 100 °C overnight, cooled to RT and concentrated under reduced pressure. After addition of ethyl acetate / water and phase separation, the aqueous phase was extracted with ethyl acetate. The combined organic phases were concentrated under reduced pressure. The residue was dried *in vacuo* to give **144** in a mixture with 11% of the corresponding iodo compound, which was used without further purification. Yield: 1.99 g (77% purity, 49% of theory). LC/MS (method 1): $t_R$ = 0.48 min, MS (ESIpos): *m*/*z* = 199 $[M+H]^+$.

**2-(4-Bromo-5-cyano-2-oxopyridin-1(2*H*)-yl)propanoic acid (racemate 145).**

A mixture of 4-bromo-6-oxo-1,6-dihydropyridine-3-carbonitrile (**144**) (1.00 g, 77% purity, 3.87 mmol), magnesium di-*tert*-butoxide (1.32 g, 7.74 mmol, 2.0 eq.) and potassium *tert*-butylate (456 mg, 4.06 mmol, 1.05 eq.) in tetrahydrofuran (20 mL) was stirred under argon atmosphere at RT for 10 min and cooled with an ice bath, followed by addition of racemic 2-bromopropanoic acid (522 μL, 5.80 mmol, 1.5 eq.). The reaction mixture was stirred at RT for 2.5 h, at 35 °C overnight and at 45 °C for further 1 d, acidified with aqueous hydrochloric acid solution (6 N) and mixed with water. The aqueous phase was extracted with ethyl acetate. The combined organic phases were dried over anhydrous sodium sulfate, filtered and concentrated under reduced pressure. The residue was stirred in a mixture of dichloromethane / cyclohexane, and the solid was filtered and dried *in vacuo* to give a first batch of **145**. Yield: 648 mg (66% purity, 41% of theory). The combined filtrates were concentrated under reduced pressure. The residue was purified by preparative RP-HPLC (acetonitrile / water gradient) to give a second batch of **145**. Yield: 110 mg (91% purity, 10% of theory). LC/MS (method 1): $t_R$ = 0.58 min, MS (ESIpos): *m*/*z* = 271 $[M+H]^+$.

***tert*-Butyl 4-[2-(4-bromo-5-cyano-2-oxopyridin-1(2*H*)-yl)propanamido]benzoate (racemate 146).**

A solution of HATU (957 mg, 2.52 mmol, 1.3 eq.) in *N*,*N*-dimethylformamide (5 mL) was added under

argon atmosphere at RT to a solution of 2-(4-bromo-5-cyano-2-oxopyridin-1(2*H*)-yl)propanoic acid (racemate **145**) (750 mg, 70% purity, 1.94 mmol), *tert*-butyl 4-aminobenzoate (449 mg, 2.32 mmol, 1.2 eq.) and *N,N*-diisopropylethylamine (742 $\mu$L, 4.26 mmol, 2.2 eq.) in *N,N*-dimethylformamide (10 mL). The reaction mixture was stirred at RT for 2 h and concentrated under reduced pressure. After addition of ethyl acetate / water and phase separation, the aqueous phase was extracted with ethyl acetate. The combined organic phases were dried over anhydrous sodium sulfate, filtered, concentrated under reduced pressure to give **146**. Yield: 704 mg (70% purity, 57% of theory). LC/MS (method 1): $t_R$ = 1.12 min, MS (ESIneg): $m/z$ = 444 $[M-H]^-$.

**_tert_-Butyl 4-(2-{4-[5-chloro-2-(trifluoromethyl)phenyl]-5-cyano-2-oxopyridin-1(2_H_)-yl}propanamido)benzoate (racemate 147).**

A mixture of *tert*-butyl 4-[2-(4-bromo-5-cyano-2-oxopyridin-1(2*H*)-yl)propanamido]benzoate (racemate **146**) (127 mg, 70% purity, 0.20 mmol), [5-chloro-2-(trifluoromethyl)phenyl]boronic acid (54 mg, 0.24 mmol, 1.2 eq.), potassium carbonate (83 mg, 0.60 mmol, 3.0 eq.) and [1,1'-bis(diphenylphosphino)ferrocene]dichloropalladium-dichloromethane complex (16 mg, 0.02 mmol, 0.1 eq.) was three times evacuated and flushed with argon at RT and mixed with 1,4-dioxane (5 mL). The reaction mixture was stirred at 110 °C (preheated oil bath) overnight, cooled to RT, mixed with additional [5-chloro-2-(trifluoromethyl)phenyl]boronic acid (22 mg, 0.10 mmol, 0.5 eq.) and [1,1'-bis(diphenylphosphino)ferrocene]dichloropalladium-dichloromethane complex (8 mg, 0.01 mmol, 0.05 eq.), stirred at 110 °C for 5 h and cooled to RT. The mixture was combined with a mixture from a second campaign of same size and filtered through Celite®. The filter residue was washed with 1,4-dioxane. The combined filtrates were concentrated under reduced pressure. The residue was stirred in water, and the solid was filtered, washed with water and dried *in vacuo*. The residue was purified by flash silica gel chromatography (cyclohexane / ethyl acetate gradient) to give **147**. Yield: 124 mg (55% of theory). LC/MS (method 1): $t_R$ = 1.28 min, MS (ESIpos): $m/z$ = 546 $[M+H]^+$; $^1$H NMR (400 MHz, DMSO-$d_6$): δ [ppm] = 10.84 / 10.81 (2x s, 1H), 8.79 (s, 1H), 7.96 (dd, $J$ = 8.6 Hz, 3.8 Hz, 1H), 7.89 (d, $J$ = 8.7 Hz, 2H), 7.86-7.81 (m, 2H), 7.71 (d, $J$ = 8.7 Hz, 2H), 6.58 (s, 1H), 5.62-5.51 (m, 1H), 1.76 (d, $J$ = 7.2 Hz, 3H), 1.54 (s, 9H).

**4-(2-{4-[5-Chloro-2-(trifluoromethyl)phenyl]-5-cyano-2-oxopyridin-1(2_H_)-yl}propanamido)benzoic acid (racemate 18).**

Trifluoroacetic acid (339 $\mu$L, 4.41 mmol, 20 eq.) was added under argon atmosphere at RT to a mixture of *tert*-butyl 4-(2-{4-[5-chloro-2-(trifluoromethyl)phenyl]-5-cyano-2-oxopyridin-1(2*H*)-yl}propanamido)benzoate (racemate **147**) (124 mg, 0.22 mmol) in dichloromethane (5 mL). The reaction mixture was stirred at RT for 4 h and concentrated under reduced pressure. The residue was coevaporated several times with dichloromethane, dried *in vacuo* and purified by preparative RP-HPLC (acetonitrile / water gradient) to give **18**. Yield: 85 mg (79% of theory). LC/MS (method 1): $t_R$ =

0.99 min, MS (ESIpos): $m/z$ = 490 $[M+H]^+$; $^1$H NMR (400 MHz, DMSO-$d_6$): δ [ppm] = 12.79 (s, 1H), 10.83 / 10.80 (2x s, 1H), 8.79 (s, 1H), 7.96 (dd, $J$ = 8.7 Hz, 3.8 Hz, 1H), 7.92 (d, $J$ = 8.7 Hz, 2H), 7.87-7.69 (m, 4H), 6.85 (s, 1H), 5.63-5.55 (m, 1H), 1.76 (d, $J$ = 7.1 Hz, 3H).

**Synthesis of racemate 19 and eutomer 24.**[a]

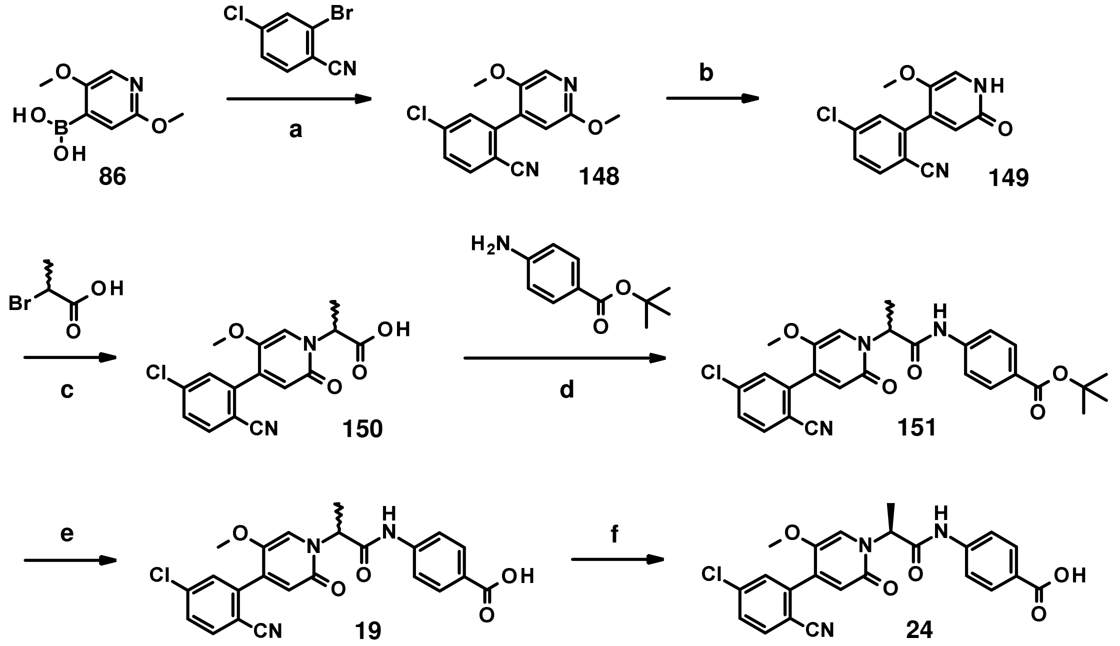


[a]Reagents and conditions: (a) Pd(dppf)$Cl_2$-DCM complex, $K_2CO_3$, 1,4-dioxane, 110 °C, 51%; (b) pyridine hydrochloride, DMF, 100 °C, 96%; (c) $Mg(OtBu)_2$, KOtBu, THF, RT, 73%; (d) HATU, DIEA, DMF, RT, 83%; (e) TFA, DCM, RT, 64%; (f) enantiomer separation.

**4-Chloro-2-(2,5-dimethoxypyridin-4-yl)benzonitrile (148).**

A mixture of 2,5-dimethoxypyridin-4-ylboronic acid (**86**) (7.87 g, 40.86 mmol), 2-bromo-4-chlorobenzonitrile (8.85 g, 40.86 mmol, 1.0 eq.), potassium carbonate (16.94 g, 122.58 mmol, 3.0 eq.) and [1,1'-bis(diphenylphosphino)ferrocene]dichloropalladium-dichloromethane complex (3.34 g, 4.09 mmol, 0.1 eq.) was three times evacuated and flushed with argon at RT and mixed with 1,4-dioxane (225 mL). The reaction mixture was stirred at 110 °C (preheated oil bath) for 19 h, mixed with additional [1,1'-bis(diphenylphosphino)ferrocene]dichloropalladium-dichloromethane complex (3.34 g, 4.09 mmol, 0.1 eq.), stirred at 110 °C for another 17 h, cooled to RT and filtered through Celite®. The filter residue was washed with 1,4-dioxane. The combined filtrates were concentrated under reduced pressure. The residue was mixed with water and extracted with ethyl acetate. The organic phases were dried over anhydrous sodium sulfate, filtered, concentrated under reduced pressure and dried *in vacuo*. The residue was purified by flash silica gel chromatography (cyclohexane / ethyl acetate mixture) to give **148**. Yield: 6.23 g (92% purity, 51% of theory). LC/MS (method 1): $t_R$ = 1.08 min, MS (ESIpos):

$m/z$ = 275 [M+H]$^+$.

**4-Chloro-2-(5-methoxy-2-oxo-1,2-dihydropyridin-4-yl)benzonitrile (149).**

Pyridine hydrochloride (55.96 g, 484.27 mmol, 20 eq.) was added at RT to a mixture of 4-chloro-2-(2,5-dimethoxypyridin-4-yl)benzonitrile (**148**) (7.23 g, 92% purity, 24.21 mmol) in *N,N*-dimethylformamide (300 mL). The reaction mixture was stirred at 100 °C for 21 h and concentrated under reduced pressure. The residue was stirred in water, and the solid was filtered, washed with water and dried *in vacuo* to give **149**. Yield: 6.66 g (91% purity, 96% of theory). LC/MS (method 1): $t_R$ = 0.76 min, MS (ESIpos): $m/z$ = 261 [M+H]$^+$; $^1$H NMR (400 MHz, DMSO-$d_6$): δ [ppm] = 11.45 (br s, 1H), 7.98 (d, $J$ = 8.3 Hz, 1H), 7.75-7.67 (m, 2H), 7.29 (br s, 1H), 6.43 (s, 1H), 3.64 (s, 3H).

**2-[4-(5-Chloro-2-cyanophenyl)-5-methoxy-2-oxopyridin-1(2*H*)-yl]propanoic acid (racemate 150).**

A mixture of 4-chloro-2-(5-methoxy-2-oxo-1,2-dihydropyridin-4-yl)benzonitrile (**149**) (599 mg, 87% purity, 2.00 mmol), magnesium di-*tert*-butoxide (682 mg, 4.00 mmol, 2.0 eq.) and potassium *tert*-butylate (236 mg, 2.10 mmol, 1.05 eq.) in tetrahydrofuran (10 mL) was stirred under argon atmosphere at RT for 10 min and cooled with an ice bath, followed by addition of racemic 2-bromopropanoic acid (270 µL, 3.00 mmol, 1.5 eq.). The reaction mixture was stirred at RT overnight, acidified to pH 1 with aqueous hydrochloric acid solution (6 N) and mixed with water. The aqueous phase was extracted with ethyl acetate. The combined organic phases were dried over anhydrous sodium sulfate, filtered, concentrated under reduced pressure and dried *in vacuo*. The residue was stirred in a mixture of ethyl acetate (2 mL), cyclohexane (2 mL) and dichloromethane (2 mL), and the solid was filtered and dried *in vacuo* to give **150** which was used without further purification. Yield: 716 mg (68% purity, 73% of theory). LC/MS (method 1): $t_R$ = 0.80 min, MS (ESIpos): $m/z$ = 333 [M+H]$^+$; $^1$H NMR (400 MHz, DMSO-$d_6$): δ [ppm] = 7.99 (d, $J$ = 9.1 Hz, 1H), 7.73 (m, 2H), 7.48 (s, 1H), 6.50 (s, 1H), 5.17 (q, $J$ = 7.2 Hz, 1H), 3.65 (s, 3H), 1.61 (d, $J$ = 7.2 Hz, 3H).

***tert*-Butyl 4-{2-[4-(5-chloro-2-cyanophenyl)-5-methoxy-2-oxopyridin-1(2*H*)-yl]propanamido}benzoate (racemate 151).**

A solution of HATU (1.53 g, 4.02 mmol, 1.2 eq.) in *N,N*-dimethylformamide (10 mL) was added under argon atmosphere at RT to a solution of 2-[4-(5-chloro-2-cyanophenyl)-5-methoxy-2-oxopyridin-1(2*H*)-yl]propanoic acid (racemate **150**) (1.53 g, 73% purity, 3.35 mmol), *tert*-butyl 4-aminobenzoate (713 mg, 3.69 mmol, 1.1 eq.) and *N,N*-diisopropylethylamine (1.29 mL, 7.38 mmol, 2.2 eq.) in *N,N*-dimethylformamide (30 mL). The reaction mixture was stirred at RT overnight and concentrated under reduced pressure. After addition of ethyl acetate / water and phase separation, the aqueous phase was extracted with ethyl acetate. The combined organic phases were washed with brine, dried over anhydrous sodium sulfate, filtered, concentrated under reduced pressure and dried *in vacuo*. The residue was purified by flash silica gel chromatography to give **151**. Yield: 1.52 g (93% purity, 83% of theory). LC/MS (method 1): $t_R$ = 1.19 min, MS (ESIpos): $m/z$ = 508 [M+H]$^+$; $^1$H NMR (400 MHz, DMSO-$d_6$):

δ [ppm] = 10.72 (s, 1H), 8.01 (d, *J* = 9.1 Hz, 1H), 7.87 (d, *J* = 6.8 Hz, 2H), 7.74 (m, 4H), 7.46 (s, 1H), 6.53 (s, 1H), 5.59 (q, *J* = 7.3 Hz, 1H), 3.70 (s, 3H), 1.74 (d, *J* = 7.3 Hz, 3H), 1.54 (s, 9H).

**4-{2-[4-(5-Chloro-2-cyanophenyl)-5-methoxy-2-oxopyridin-1(2*H*)-yl]propanamido}benzoic acid (racemate 19).**

Trifluoroacetic acid (127 µL, 1.65 mmol, 20 eq.) was added under argon atmosphere at RT to a mixture of *tert*-butyl 4-{2-[4-(5-chloro-2-cyanophenyl)-5-methoxy-2-oxopyridin-1(2*H*)-yl]propanamido}-benzoate (racemate **151**) (42 mg, 0.08 mmol) in dichloromethane (2 mL). The reaction mixture was stirred at RT for 2.5 h and concentrated under reduced pressure. The residue was purified by preparative RP-HPLC (acetonitrile / water gradient) to give **19**. Yield: 24 mg (64% of theory). LC/MS (method 1): $t_R$ = 0.92 min, MS (ESIpos): *m*/*z* = 452 $[M+H]^+$; $^1H$ NMR (400 MHz, DMSO-$d_6$): δ [ppm] = 12.77 (s, 1H), 10.71 (s, 1H), 8.01 (d, *J* = 9.1 Hz, 1H), 7.91 (d, *J* = 8.7 Hz, 2H), 7.77-7.71 (m, 4H), 7.47 (s, 1H), 6.53 (s, 1H), 5.60 (q, *J* = 7.3 Hz, 1H), 3.70 (s, 3H), 1.74 (d, *J* = 7.3 Hz, 3H).

**4-({(2*S*)-2-[4-(5-Chloro-2-cyanophenyl)-5-cyano-2-oxopyridin-1(2*H*)-yl]propanoyl}amino)benzoic acid (eutomer 24).**

Enantiomer separation of 450 mg of 4-{2-[4-(5-chloro-2-cyanophenyl)-5-methoxy-2-oxopyridin-1(2*H*)-yl]propanamido}benzoic acid (racemate **19**) gave 214 mg of distomer (chiral HPLC: $t_R$ = 4.3 min) and 223 mg of eutomer **24** (chiral HPLC: $t_R$ = 5.9 min, 99% ee).

LC/MS (method 1): $t_R$ = 0.92 min, MS (ESIpos): *m*/*z* = 452 $[M+H]^+$; $^1H$ NMR (400 MHz, DMSO-$d_6$): δ [ppm] = 12.75 (s, 1H), 10.70 (s, 1H), 8.01 (d, *J* = 9.1 Hz, 1H), 7.91 (d, *J* = 8.8 Hz, 2H), 7.77-7.71 (m, 4H), 7.47 (s, 1H), 6.53 (s, 1H), 5.60 (q, *J* = 7.3 Hz, 1H), 3.70 (s, 3H), 1.74 (d, *J* = 7.3 Hz, 3H).

Separation method: column: Daicel Chiralcel OZ 5 µm, 250 mm × 20 mm; mobile phase: 50% *iso*-hexane / 50% ethanol; temperature: 40 °C; flow rate: 15 mL/min; UV detection: 220 nm.

Analytical method: column: Daicel Chiralcel OZ 5 µm, 250 mm × 4.6 mm; mobile phase: 30% *iso*-hexanes / 70% ethanol plus 0.2% trifluoroacetic acid in 1% water; temperature: 45 °C; flow rate: 1 mL/min; UV detection: 220 nm.

**Synthesis of racemate 20.**[a]

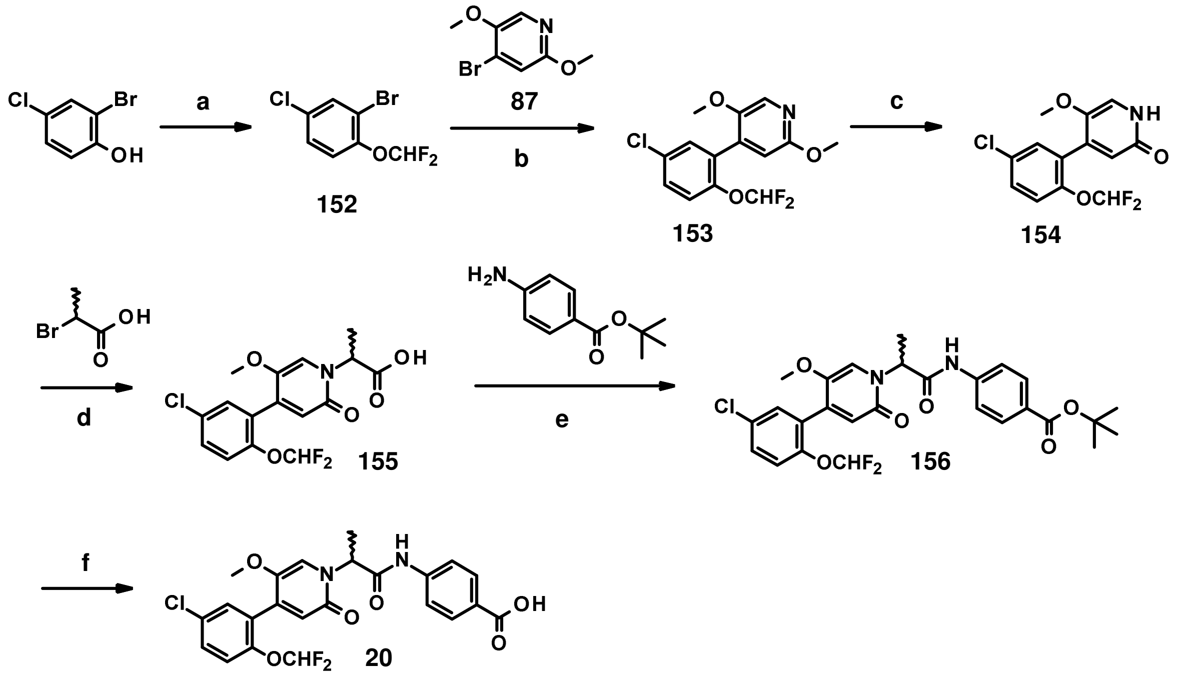

[a]Reagents and conditions: (a) aq. KOH, difluoromethyl trifluoromethanesulfonate, ACN, 0 °C, 78%; (b) Pd(dppf)$Cl_2$-DCM complex, $K_2CO_3$, 1,4-dioxane, 110 °C, 27%; (c) pyridine hydrobromide, DMF, 100 °C, 87%; (d) Mg(OtBu)$_2$, KOtBu, THF, RT; (e) HATU, DIEA, DMF, RT, 21%; (f) TFA, DCM, RT, 78%.

**2-Bromo-4-chlorophenyl-difluoromethylether (152).**

Aqueous potassium hydroxide solution (6 M, 36 mL) was added at RT to a solution of 2-bromo-4-chlorophenol (3.50 g, 16.87 mmol) in acetonitrile (36 mL). The reaction mixture was cooled with an ice bath, followed by dropwise addition of difluoromethyl trifluoromethanesulfonate [*Angew. Chem. Int. Ed.* **2013**, *52*, 1-5; *Journal of Fluorine Chemistry* **2009**, *130*, 667-670] (6.55 mL, 26.99 mmol, 1.6 eq.) under vigorously stirring. The reaction mixture was stirred for 5 min and diluted with water. The aqueous phase was extracted with diethyl ether. The combined organic phases were dried over anhydrous sodium sulfate, filtered, concentrated under reduced pressure and dried *in vacuo* to give **152**. Yield: 3.40 g (78% of theory). GC/MS (method 9): $t_R$ = 3.51 min, MS (EI): *m*/*z* = 256 $[M]^+$; $^1$H NMR (400 MHz, DMSO-*d*$_6$): δ [ppm] = 7.91 (d, *J* = 2.6 Hz, 1H), 7.55 (dd, *J* = 8.8 Hz, 2.6 Hz, 1H), 7.37 (d, *J* = 8.8 Hz, 1H), 7.30 (t, *J* = 72.9 Hz, 1H).

**4-[5-Chloro-2-(difluoromethoxy)phenyl]-2,5-dimethoxypyridine (153).**

A mixture of 2-bromo-4-chloro-1-(difluoromethoxy)benzene (**152**) (494 mg, 1.82 mmol), (2,5-dimethoxypyridin-4-yl)boronic acid (**87**) (417 mg, 2.19 mmol, 1.2 eq.), potassium carbonate (755 mg, 5.47 mmol, 3.0 eq.) and [1,1'-bis(diphenylphosphino)ferrocene]dichloropalladium-dichloromethane complex (149 mg, 0.18 mmol, 0.1 eq.) was three times evacuated and flushed with argon at RT and

mixed with 1,4-dioxane (26 mL). The reaction mixture was stirred at 110 °C (preheated oil bath) overnight, cooled to RT and concentrated under reduced pressure. After addition of ethyl acetate / water and phase separation, the aqueous phase was extracted with ethyl acetate. The combined organic phases were washed with brine, dried over anhydrous sodium sulfate, filtered and concentrated under reduced pressure. The residue was purified by flash silica gel chromatography (petroleum ether / ethyl acetate gradient) to give **153**. Yield: 170 mg (90% purity, 27% of theory). LC/MS (method 1): $t_R$ = 1.16 min, MS (ESIpos): *m/z* = 316 $[M+H]^+$; $^1$H NMR (400 MHz, DMSO-$d_6$): δ [ppm] = 7.96 (s, 1H), 7.57 (dd, *J* = 8.8 Hz, 2.7 Hz, 1H), 7.45 (d, *J* = 2.7 Hz, 1H), 7.30 (d, *J* = 8.7 Hz, 1H), 7.11 (t, *J* = 73.4 Hz, 1H), 6.74 (s, 1H), 3.83 (s, 3H), 3.75 (s, 3H).

**4-[5-Chloro-2-(difluoromethoxy)phenyl]-5-methoxypyridin-2(1*H*)-one (154).**

Pyridine hydrobromide (1.55 g, 9.69 mmol, 20 eq.) was added under argon atmosphere at RT to a mixture of 4-[5-chloro-2-(difluoromethoxy)phenyl]-2,5-dimethoxypyridine (**153**) (170 mg, 90% purity, 0.49 mmol) in *N,N*-dimethylformamide (5 mL). The reaction mixture was stirred at 100 °C for 12 h, cooled to RT and concentrated under reduced pressure. After addition of ethyl acetate / water and phase separation, the aqueous phase was extracted with ethyl acetate. The combined organic phases were washed with brine, dried over anhydrous sodium sulfate, filtered and concentrated under reduced pressure. The residue was purified by flash silica gel chromatography (dichloromethane / methanol gradient) to give **154**. Yield: 127 mg (87% of theory). LC/MS (method 1): $t_R$ = 0.84 min, MS (ESIpos): *m/z* = 302 $[M+H]^+$.

**2-{4-[5-Chloro-2-(difluoromethoxy)phenyl]-5-methoxy-2-oxopyridin-1(2*H*)-yl}propanoic acid (racemate 155).**

A mixture of 4-[5-chloro-2-(difluoromethoxy)phenyl]-5-methoxypyridin-2(1*H*)-one (**154**) (127 mg, 0.42 mmol), magnesium di-*tert*-butoxide (144 mg, 0.84 mmol, 2.0 eq.) and potassium *tert*-butylate (52 mg, 0.44 mmol, 1.05 eq.) in tetrahydrofuran (2.7 mL) was stirred under argon atmosphere at RT for 10 min and cooled with an ice bath, followed by addition of racemic 2-bromopropanoic acid (57 $\mu$L, 0.63 mmol, 1.5 eq.). The reaction mixture was stirred at RT for 2.5 h and at 45 °C overnight, acidified with aqueous hydrochloric acid solution (6 N) and mixed with water. The aqueous phase was extracted with ethyl acetate. The combined organic phases were dried over anhydrous sodium sulfate, filtered, concentrated under reduced pressure and dried *in vacuo* to give **155** which was used without further purification. Yield: 220 mg.

***tert*-Butyl 4-(2-{4-[5-chloro-2-(difluoromethoxy)phenyl]-5-methoxy-2-oxopyridin-1(2*H*)-yl}propanamido)benzoate (racemate 156).**

HATU (192 mg, 0.50 mmol, 1.2 eq.) was added under argon atmosphere at RT to a solution of 2-{4-[5-chloro-2-(difluoromethoxy)phenyl]-5-methoxy-2-oxopyridin-1(2*H*)-yl}propanoic acid (racemate **155**) (220 mg raw material), *tert*-butyl 4-aminobenzoate (89 mg, 0.46 mmol, 1.1 eq.) and *N,N*-

diisopropylethylamine (161 $\mu$L, 0.92 mmol, 2.2 eq.) in *N,N*-dimethylformamide (3 mL). The reaction mixture was stirred at RT for 5 h and concentrated under reduced pressure. After addition of diethyl ether / water and phase separation, the aqueous phase was extracted with diethyl ether. The combined organic phases were washed with brine, dried over anhydrous sodium sulfate, filtered and concentrated under reduced pressure. The residue was purified by preparative RP-HPLC (acetonitrile / 0.1% formic acid in water gradient) to give **156**. Yield: 48 mg (21% of theory). LC/MS (method 1): $t_R$ = 1.26 min, MS (ESIpos): *m/z* = 549 $[M+H]^+$; $^1$H NMR (400 MHz, DMSO-$d_6$): δ [ppm] = 10.70 (s, 1H), 7.87 (d, *J* = 8.8 Hz, 2H), 7.73 (d, *J* = 8.8 Hz, 2H), 7.58 (dd, *J* = 8.8 Hz, 2.7 Hz, 1H), 7.48 (d, *J* = 2.7 Hz, 1H), 7.35 (s, 1H), 7.30 (d, *J* = 8.7 Hz, 1H), 7.16 (t, *J* = 73.4 Hz, 1H), 6.38 (s, 1H), 5.62-5.53 (m, 1H), 3.65 (s, 3H), 1.71 (d, *J* = 7.3 Hz, 3H), 1.54 (s, 9H).

**4-(2-{4-[5-Chloro-2-(difluoromethoxy)phenyl]-5-methoxy-2-oxopyridin-1(2*H*)-yl}propanamido)-benzoic acid (racemate 20).**

Trifluoroacetic acid (1.0 mL) was added under argon atmosphere at 0 °C to a mixture of *tert*-butyl 4-(2-{4-[5-chloro-2-(difluoromethoxy)phenyl]-5-methoxy-2-oxopyridin-1(2*H*)-yl}propanamido)benzoate (racemate **156**) (47 mg, 0.09 mmol) in dichloromethane (2 mL). The reaction mixture was stirred at 0 °C for 10 min and at RT for 45 min and concentrated under reduced pressure. The residue was purified by preparative RP-HPLC (acetonitrile / 0.1% formic acid in water gradient) to give **20**. Yield: 33 mg (78% of theory). LC/MS (method 1): $t_R$ = 0.93 min, MS (ESIpos): *m/z* = 493 $[M+H]^+$; $^1$H NMR (400 MHz, DMSO-$d_6$): δ [ppm] = 12.76 (br s, 1H), 10.68 (s, 1H), 7.91 (d, *J* = 8.8 Hz, 2H), 7.73 (d, *J* = 8.8 Hz, 2H), 7.58 (dd, *J* = 8.8 Hz, 2.7 Hz, 1H), 7.47 (d, *J* = 2.7 Hz, 1H), 7.36 (s, 1H), 7.30 (d, *J* = 8.8 Hz, 1H), 7.16 (t, *J* = 73.4 Hz, 1H), 6.38 (s, 1H), 5.63-5.54 (m, 1H), 3.65 (s, 3H), 1.72 (d, *J* = 7.3 Hz, 3H).

**Synthesis of racemate 21.**[a]

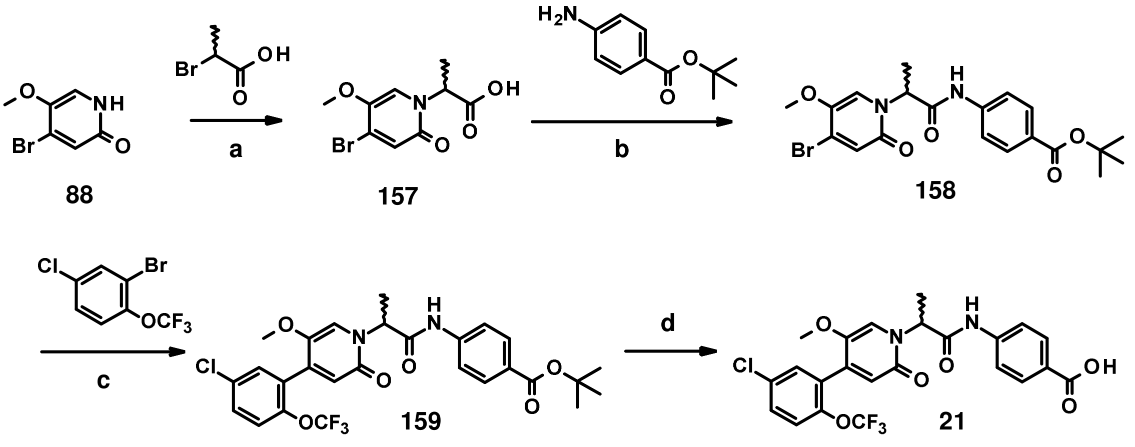


[a]Reagents and conditions: (a) $Mg(OtBu)_2$, KOtBu, THF, 0 °C → 50 °C, 53%; (b) HATU, DIEA, DMF, RT, 61%; (c) $Pd(dppf)Cl_2$-DCM complex, $K_2CO_3$, 1,4-dioxane, 110 °C, 82%; (d) TFA, DCM, RT, 54%.

**2-(4-Bromo-5-methoxy-2-oxopyridin-1(2*H*)-yl)propanoic acid (racemate 157).**

A mixture of 4-bromo-5-methoxypyridin-2(1*H*)-one (**88**) (1.24 g, 5.15 mmol), magnesium di-*tert*-butoxide (1.76 g, 10.30 mmol, 2.0 eq.) und potassium *tert*-butylate (607 mg, 5.41 mmol, 1.05 eq.) in tetrahydrofuran (30 mL) was stirred under argon atmosphere at RT for 10 min and cooled with an ice bath, followed by addition of racemic 2-bromopropanoic acid (695 $\mu$L, 7.72 mmol, 1.5 eq.). The reaction mixture was stirred at RT for 2.5 h and at 50 °C overnight, acidified with aqueous hydrochloric acid solution (6 N) and diluted with ethyl acetate and water. The forming precipitate was filtered and dried *in vacuo* to give a first batch of **157**. Yield: 205 mg (14% of theory). The phases of the filtrate were separated. The aqueous phase was extracted with ethyl acetate. The combined organic phases were dried over anhydrous sodium sulfate, filtered and concentrated under reduced pressure. The residue was reacted again as described above with magnesium di-*tert*-butoxide (1.05 g, 6.18 mmol), potassium *tert*-butylate (376 mg, 3.35 mmol) and racemic 2-bromopropanoic acid (371 $\mu$L, 4.12 mmol) in tetrahydrofuran (30 mL) followed by analogous work-up to give a second batch of **157**. Yield: 571 mg (39% of theory). LC/MS (method 1): $t_R$ = 0.57 min, MS (ESIpos): $m/z$ = 276 $[M+H]^+$.

***tert*-Butyl 4-[2-(4-bromo-5-methoxy-2-oxopyridin-1(2*H*)-yl)propanamido]benzoate (racemate 158).**

A solution of HATU (915 mg, 2.41 mmol, 1.2 eq.) in *N,N*-dimethylformamide (4 mL) was added under argon atmosphere at RT to a solution of 2-(4-bromo-5-methoxy-2-oxopyridin-1(2*H*)-yl)propanoic acid (racemate **157**) (571 mg, 2.01 mmol), *tert*-butyl 4-aminobenzoate (426 mg, 2.21 mmol, 1.1 eq.) and *N,N*-diisopropylethylamine (769 $\mu$L, 4.41 mmol, 2.2 eq.) in *N,N*-dimethylformamide (11 mL). The reaction mixture was stirred at RT for 2 h and concentrated under reduced pressure. The residue was stirred in dichloromethane, and the solid was filtered and discarded. The filtrate was concentrated under reduced pressure. The residue was purified by flash silica gel chromatography (cyclohexane / ethyl acetate gradient) to give **158**. Yield: 562 mg (61% of theory). LC/MS (method 1): $t_R$ = 1.10 min, MS (ESIpos): $m/z$ = 451 $[M+H]^+$.

***tert*-Butyl 4-(2-{4-[5-chloro-2-(trifluoromethoxy)phenyl]-5-methoxy-2-oxopyridin-1(2*H*)-yl}propanamido)benzoate (racemate 159).**

A mixture of *tert*-butyl 4-[2-(4-bromo-5-methoxy-2-oxopyridin-1(2*H*)-yl)propanamido]benzoate (racemate **158**) (125 mg, 0.28 mmol), 5-chloro-2-trifluoromethoxyphenylboronic acid (80 mg, 0.33 mmol, 1.2 eq.) and potassium carbonate (115 mg, 0.83 mmol, 3.0 eq.) in 1,4-dioxane (5.0 mL) was flushed with argon at RT, followed by addition of [1,1'-bis(diphenylphosphino)ferrocene]-dichloropalladium-dichloromethane complex (23 mg, 0.03 mmol, 0.1 eq.). The reaction mixture was stirred at 110 °C (preheated oil bath) overnight, cooled to RT and filtered through Celite®, and the filter residue was washed with 1,4-dioxane. The combined filtrates were concentrated under reduced pressure. The residue was stirred in water, and the solid was filtered, washed with water and dried *in vacuo* to

give **159**. Yield: 155 mg (83% purity, 82% of theory). LC/MS (method 1): $t_R$ = 1.34 min, MS (ESIpos): $m/z$ = 567 $[M+H]^+$.

**4-(2-{4-[5-Chloro-2-(trifluoromethoxy)phenyl]-5-methoxy-2-oxopyridin-1(2*H*)-yl}propanamido)benzoic acid (racemate 21).**

Trifluoroacetic acid (350 $\mu$L, 4.54 mmol, 20 eq.) was added under argon atmosphere at RT to a mixture of *tert*-butyl 4-(2-{4-[5-chloro-2-(trifluoromethoxy)phenyl]-5-methoxy-2-oxopyridin-1(2*H*)-yl}propanamido)benzoate (racemate **159**) (155 mg, 0.23 mmol) in dichloromethane (5 mL). The reaction mixture was stirred at RT for 3 h and concentrated under reduced pressure. The residue was coevaporated several times with dichloromethane and purified by preparative RP-HPLC (acetonitrile / water gradient) to give **21**. Yield: 67 mg (94% purity, 54% of theory). LC/MS (method 1): $t_R$ = 1.03 min, MS (ESIpos): $m/z$ = 511 $[M+H]^+$; $^1H$ NMR (400 MHz, DMSO-$d_6$): δ [ppm] = 12.76 (br s, 1H), 10.68 (s, 1H), 7.91 (d, $J$ = 8.8 Hz, 2H), 7.73 (d, $J$ = 8.8 Hz, 2H), 7.65 (dd, $J$ = 8.8 Hz, 2.6 Hz, 1H), 7.59 (d, $J$ = 2.6 Hz, 1H), 7.51 (d, $J$ = 8.8 Hz, 1H), 7.40 (s, 1H), 6.41 (s, 1H), 5.64-5.55 (m, 1H), 3.65 (s, 3H), 1.72 (d, $J$ = 7.3 Hz, 3H).

**Synthesis of racemate 22.**[a]

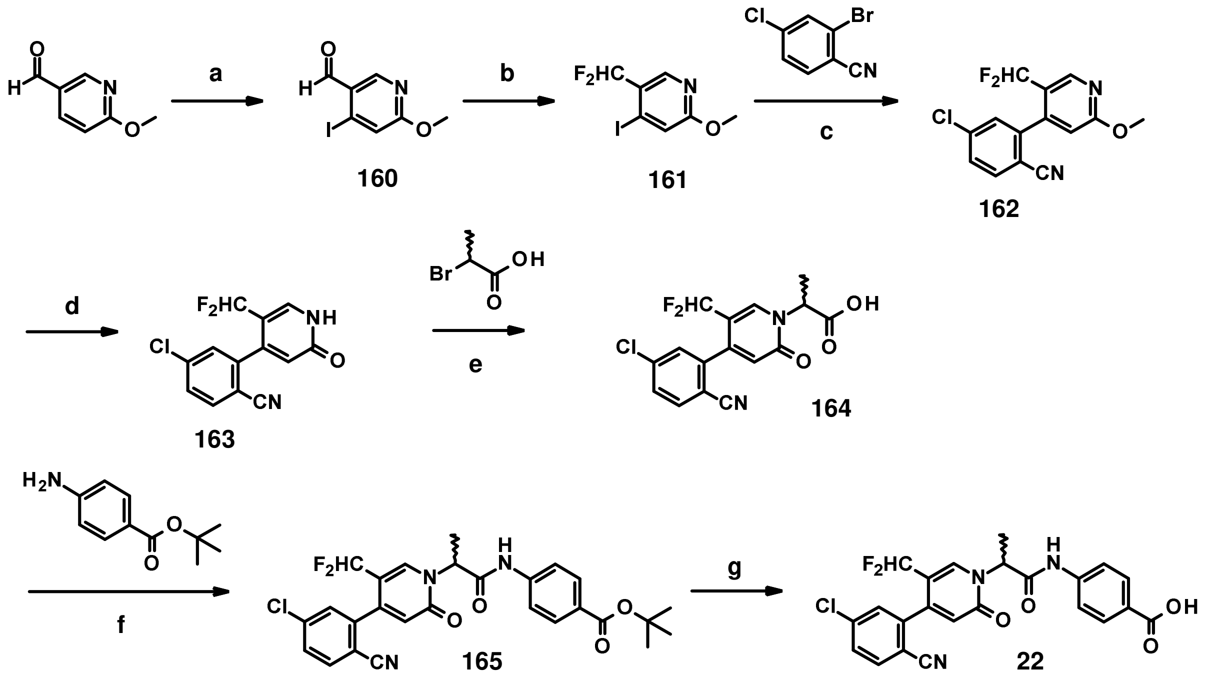


[a]Reagents and conditions: (a) *n*-BuLi, TMEDA, $I_2$, THF, -78 °C → RT, 23%; (b) DAST, DCM, 0 °C → RT, 52%; (c) Pd(dppf)$Cl_2$-DCM complex, $K_2CO_3$, 1,4-dioxane, 110 °C, 42%; (d) pyridine hydrobromide, DMF, 100 °C, 67%; (e) $Mg(OtBu)_2$, KOtBu, THF, 0 °C → 50 °C, 95%; (f) HATU, DIEA, DMF, RT, 76%; (g) TFA, DCM, RT, 80%.

**4-Iodo-6-methoxypyridin-3-carbaldehyde (160).**

*n*-Butyllithium solution (1.6 M in hexane, 25.07 mL, 40.11 mmol, 1.1 eq.) was added under argon atmosphere at -78 °C to a solution of *N,N,N'*-trimethyl ethylenediamine (5.69 mL, 43.75 mmol, 1.2 eq.) in tetrahydrofuran (135 mL). The reaction mixture was stirred at -78 °C for 45 min, mixed with 6-methoxynicotinaldehyde (5.00 g, 36.46 mmol), stirred at -78 °C for 45 min and mixed with additional *n*-butyllithium solution (1.6 M in hexane, 45.57 mL, 72.92 mmol, 2.0 eq.). The reaction mixture was stirred for 1 h while allowing to warm to -40 °C, stirred at -40 °C for 1 h, cooled again to -78 °C and mixed within 50 min with a solution of iodine (18.51 g, 72.92 mmol, 2.0 eq.) in tetrahydrofuran (90 mL). The reaction mixture was stirred at -78 °C for 4 h, allowed to slowly warm to RT overnight and added to saturated aqueous sodium chloride solution. After phase separation, the aqueous phase was extracted with ethyl acetate. The combined organic phases were dried over anhydrous sodium sulfate, filtered and concentrated under reduced pressure. The residue was stirred in acetonitrile, and the solid filtered and dried *in vacuo* to give a first batch of **160**. Yield: 647 mg (91% purity, 6% of theory). Further solid from the mother liquid was isolated to give a second batch of **160**. Yield: 1.05 g (70% purity, 8% of theory). The combined mother liquids were concentrated under reduced pressure. The residue was purified by flash silica gel chromatography (cyclohexane / ethyl acetate gradient) to give a third batch of **160**. Yield: 1.19 g (75% purity, 9% of theory). LC/MS (method 1): $t_R$ = 0.90 min, MS (ESIpos): *m/z* = 264 $[M+H]^+$; $^1H$ NMR (400 MHz, DMSO-$d_6$): δ [ppm] = 9.89 (s, 1H), 8.52 (s, 1H), 7.56 (s, 1H), 3.94 (s, 3H).

**5-(Difluoromethyl)-4-iodo-2-methoxypyridine (161).**

Diethylaminosulfur trifluoride (686 $\mu$L, 5.19 mmol, 1.5 eq.) was added under argon atmosphere at 0 °C to a solution of 4-iodo-6-methoxypyridin-3-carbaldehyde (**160**) (1.00 g, 91% purity, 3.46 mmol) in dichloromethane (30 mL). The reaction mixture was stirred at RT overnight, added dropwise (!) to a saturated aqueous sodium bicarbonate solution and stirred until carbon dioxide evolution stopped. After addition of ethyl acetate and phase separation, the aqueous phase was extracted with ethyl acetate. The combined organic phases were washed with brine, dried over anhydrous sodium sulfate, filtered, concentrated under reduced pressure and briefly (!) dried *in vacuo* to give **161**. Yield: 616 mg (83% purity, 52% of theory). LC/MS (method 1): $t_R$ = 1.04 min, MS (ESIpos): *m/z* = 286 $[M+H]^+$.

**4-Chloro-2-[5-(difluoromethyl)-2-methoxypyridin-4-yl]benzonitrile (162).**

A mixture of 5-(difluoromethyl)-4-iodo-2-methoxypyridine (**161**) (616 mg, 83% purity, 1.79 mmol), 5-chloro-2-cyanophenylboronic acid (325 mg, 1.79 mmol, 1.0 eq.), potassium carbonate (744 mg, 5.38 mmol, 3.0 eq.) and [1,1'-bis(diphenylphosphino)ferrocene]dichloropalladium-dichloromethane complex (146 mg, 0.18 mmol, 0.1 eq.) was three times evacuated and flushed with argon at RT and mixed with 1,4-dioxane (15 mL). The reaction mixture was stirred at 110 °C (preheated oil bath) overnight, mixed with additional 5-chloro-2-cyanophenylboronic acid (163 mg, 0.89 mmol, 0.5 eq.) and

[1,1'-bis(diphenylphosphino)ferrocene]dichloropalladium-dichloromethane complex (73 mg, 0.09 mmol, 0.05 eq.), stirred at 110 °C overnight, cooled to RT and concentrated under reduced pressure. The residue was mixed with water and extracted with ethyl acetate. The organic phases were washed with brine, dried over anhydrous sodium sulfate, filtered and concentrated under reduced pressure. The residue was purified by flash silica gel chromatography (cyclohexane / ethyl acetate gradient) to give **162**. Yield: 223 mg (42% of theory). LC/MS (method 1): $t_R$ = 1.11 min, MS (ESIpos): $m/z$ = 295 $[M+H]^+$.

**4-Chloro-2-[5-(difluoromethyl)-2-oxo-1,2-dihydropyridin-4-yl]benzonitrile (163).**

Pyridine hydrobromide (2.35 g, 14.66 mmol, 20 eq.) was added under argon atmosphere at RT to a mixture of 4-chloro-2-[5-(difluoromethyl)-2-methoxypyridin-4-yl]benzonitrile (**162**) (216 mg, 0.73 mmol) in *N,N*-dimethylformamide (5 mL). The reaction mixture was stirred at 100 °C overnight, cooled to RT and concentrated under reduced pressure. The residue was mixed with ethyl acetate and water, and the aqueous phase was extracted with ethyl acetate. The organic phase was dried over anhydrous sodium sulfate, filtered, concentrated under reduced pressure and dried *in vacuo* to give **163** which was used without further purification. Yield: 215 mg (64% purity, 67% of theory). LC/MS (method 1): $t_R$ = 0.81 min, MS (ESIpos): $m/z$ = 281 $[M+H]^+$.

**2-[4-(5-Chloro-2-cyanophenyl)-5-(difluoromethyl)-2-oxopyridin-1(2*H*)-yl]propanoic acid (racemate 164).**

A mixture of 4-chloro-2-[5-(difluoromethyl)-2-oxo-1,2-dihydropyridin-4-yl]benzonitrile (**163**) (215 mg, 64% purity, 0.49 mmol), magnesium di-*tert*-butoxide (167 mg, 0.98 mmol, 2.0 eq.) and potassium *tert*-butylate (58 mg, 0.52 mmol, 1.05 eq.) in tetrahydrofuran (5 mL) was stirred under argon atmosphere at RT for 10 min and cooled with an ice bath, followed by addition of racemic 2-bromopropanoic acid (66 $\mu$L, 0.74 mmol, 1.5 eq.). The reaction mixture was stirred at RT for 2.5 h and at 50 °C for 2 d and acidified with aqueous hydrochloric acid solution (6 N). After addition of ethyl acetate / water and phase separation, the aqueous phase was extracted with ethyl acetate. The combined organic phases were washed with brine, dried over anhydrous sodium sulfate, filtered, concentrated under reduced pressure and dried *in vacuo* to give **164** which was used without further purification. Yield: 256 mg (64% purity, 95% of theory). LC/MS (method 1): $t_R$ = 0.86 min, MS (ESIpos): $m/z$ = 353 $[M+H]^+$.

***tert*-Butyl 4-{2-[4-(5-chloro-2-cyanophenyl)-5-(difluoromethyl)-2-oxopyridin-1(2*H*)-yl]propanamido}benzoate (racemate 165).**

A solution of HATU (74 mg, 0.20 mmol, 1.2 eq.) in *N,N*-dimethylformamide (2 mL) was added under argon atmosphere at RT to a solution of 2-[4-(5-chloro-2-cyanophenyl)-5-(difluoromethyl)-2-oxopyridin-1(2*H*)-yl]propanoic acid (racemate 1**64**) (65 mg, 88% purity, 0.16 mmol), *tert*-butyl 4-aminobenzoate (34 mg, 0.18 mmol, 1.1 eq.) and *N,N*-diisopropylethylamine (62 $\mu$L, 0.36 mmol, 2.2 eq.)

in *N,N*-dimethylformamide (6 mL). The reaction mixture was stirred at RT for 3 h and concentrated under reduced pressure. The residue was purified without further work-up by preparative RP-HPLC (acetonitrile / water gradient) to give **165**. Yield: 65 mg (76% of theory). LC/MS (method 1): $t_R$ = 1.23 min, MS (ESIpos): *m*/*z* = 528 $[M+H]^+$; $^1H$ NMR (400 MHz, DMSO-$d_6$): δ [ppm] = 10.85 (s, 1H), 8.30 (s, 1H), 8.02 (d, *J* = 8.3 Hz, 1H), 7.88 (d, *J* = 8.6 Hz, 2H), 7.74 (m, 4H), 6.85 (t, *J* = 54.0 Hz, 1H), 6.56 (s, 1H), 5.63-5.53 (m, 1H), 1.74 (d, *J* = 7.3 Hz, 3H), 1.55 (s, 9H).

**4-{2-[4-(5-Chloro-2-cyanophenyl)-5-(difluoromethyl)-2-oxopyridin-1(2*H*)-yl]propanamido}-benzoic acid (racemate 22).**

Trifluoroacetic acid (187 μL, 2.42 mmol, 20 eq.) was added under argon atmosphere at RT to a mixture of *tert*-butyl 4-{2-[4-(5-chloro-2-cyanophenyl)-5-(difluoromethyl)-2-oxopyridin-1(2*H*)-yl]propanamido}benzoate (racemate **165**) (64 mg, 0.12 mmol) in dichloromethane (3 mL). The reaction mixture was stirred at RT for 4.5 h and concentrated under reduced pressure. The residue was coevaporated several times with dichloromethane and purified by preparative RP-HPLC (acetonitrile / water gradient) to give **22**. Yield: 46 mg (80% of theory). LC/MS (method 1): $t_R$ = 0.95 min, MS (ESIpos): *m*/*z* = 472 $[M+H]^+$; $^1H$ NMR (400 MHz, DMSO-$d_6$): δ [ppm] = 12.79 (s, 1H), 10.83 (s, 1H), 8.30 (s, 1H), 8.02 (d, *J* = 8.6 Hz, 1H), 7.91 (d, *J* = 8.7 Hz, 2H), 7.79-7.69 (m, 4H), 6.85 (t, *J* = 54.2 Hz, 1H), 6.57 (s, 1H), 5.63-5.54 (m, 1H), 1.74 (d, *J* = 6.9 Hz, 3H).

**Synthesis of racemate 23.**[a]

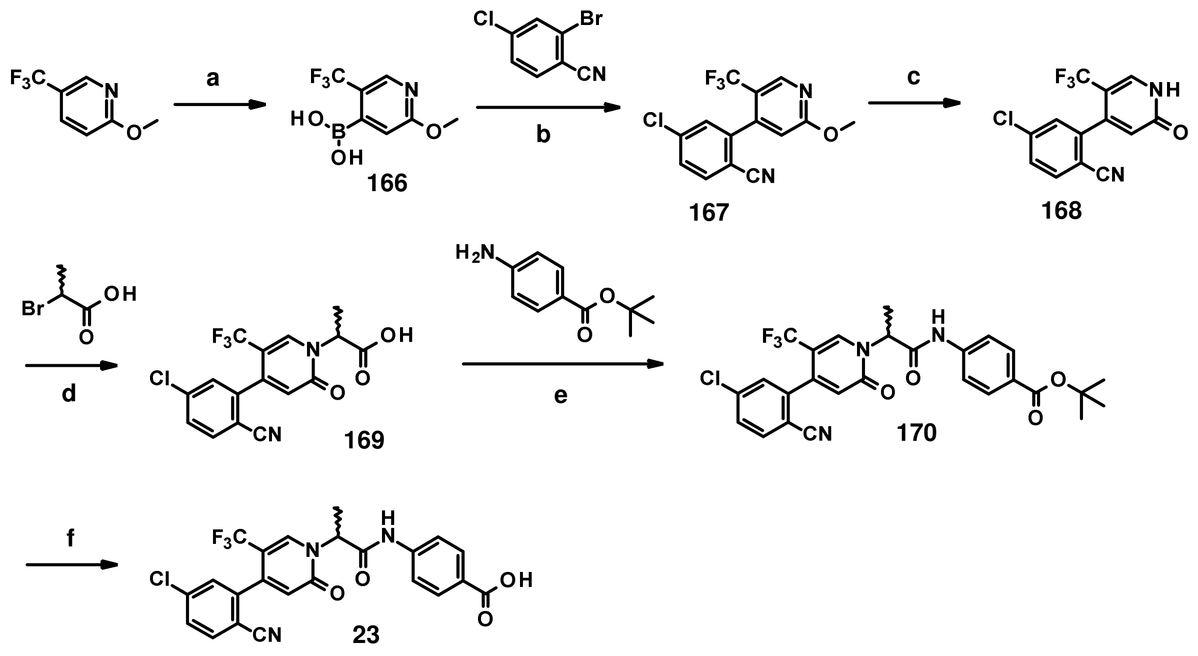

[a]Reagents and conditions: (a) i) LDA, ii) $B(OiPr)_3$, iii) aq. HCl, THF, -78 °C → RT, 34%; (b) $Pd(dppf)Cl_2$-DCM complex, $K_2CO_3$, DMF, 110 °C, 18%; (c) pyridine hydrochloride, DMF, 100 °C, 100%; (d) $Mg(OtBu)_2$, KOtBu, THF, 0 °C → 50 °C, 54%; (e) HATU, DIEA, DMF, RT, 51%; (f) TFA, DCM, RT, 20%.

**2-Methoxy-5-trifluoromethylpyridin-4-ylboronic acid (166).**

Lithium diisopropylamide solution (2 M in tetrahydrofuran, 33.87 mL, 67.75 mmol, 1.2 eq.) was added under argon atmosphere at -78 °C to a solution of 2-methoxy-5-(trifluoromethyl)pyridine (10.00 g, 56.45 mmol) in tetrahydrofuran (80 mL). The reaction mixture was stirred at -78 °C for 2 h, mixed at -78 °C with triisopropyl borate (26.35 mL, 114.61 mmol, 2.03 eq.), stirred at -78 °C for 2 h, allowed to slowly warm to RT overnight and carefully added to water. Tetrahydrofuran was removed under reduced pressure. The remaining mixture was extracted with ethyl acetate. The aqueous phase was acidified with aqueous hydrochloric acid solution (1 N) and again extracted with ethyl acetate. The combined organic phases were dried over anhydrous magnesium sulfate, filtered, concentrated under reduced pressure and dried *in vacuo*. The residue was purified by flash silica gel chromatography (cyclohexane / ethyl acetate gradient) to give **166**. Yield: 4.40 g (34% of theory). $^{1}$H NMR (400 MHz, DMSO-$d_6$): δ [ppm] = 8.63 (br s, 2H), 8.50 (s, 1H), 6.91 (s, 1H), 3.92 (s, 3H).

**4-Chloro-2-[2-methoxy-5-(trifluoromethyl)pyridin-4-yl]benzonitrile (167).**

A mixture of 2-methoxy-5-trifluoromethylpyridin-4-ylboronic acid (**166**) (1.00 g, 4.39 mmol), 2-bromo-4-chlorobenzonitrile (950 mg, 4.39 mmol, 1.0 eq.), potassium carbonate (1.82 g, 13.17 mmol, 3.0 eq.) and [1,1'-bis(diphenylphosphino)ferrocene]dichloropalladium-dichloromethane complex

(359 mg, 0.44 mmol, 0.1 eq.) was three times evacuated and flushed with argon at RT and mixed with *N,N*-dimethylformamide (24 mL). The reaction mixture was stirred at 110 °C (preheated oil bath) overnight, cooled to RT and concentrated under reduced pressure. The residue was mixed with ethyl acetate and water and filtered through Celite®. The filter residue was washed with ethyl acetate. After phase separation of the combined filtrates, the aqueous phase was extracted with ethyl acetate. The combined organic phases were concentrated under reduced pressure. The residue was purified by flash silica gel chromatography (cyclohexane / ethyl acetate gradient) to give **167**. Yield: 351 mg (71% purity, 18% of theory). LC/MS (method 1): $t_R$ = 1.19 min, MS (ESIpos): *m/z* = 313 $[M+H]^+$; $^1$H NMR (400 MHz, DMSO-$d_6$): δ [ppm] = 8.76 (s, 1H), 8.07 (d, *J* = 8.3 Hz, 1H), 7.81 (dd, *J* = 8.4 Hz, 2.1 Hz, 1H), 7.77 (d, *J* = 1.8 Hz, 1H), 7.16 (s, 1H), 4.01 (s, 3H).

**4-Chloro-2-[2-oxo-5-(trifluoromethyl)-1,2-dihydropyridin-4-yl]benzonitrile (168).**

Pyridine hydrochloride (2.36 g, 20.44 mmol, 20 eq.) was added under argon atmosphere at RT to a mixture of 4-chloro-2-[2-methoxy-5-(trifluoromethyl)pyridin-4-yl]benzonitrile (**167**) (450 mg, 71% purity, 1.02 mmol) in *N,N*-dimethylformamide (10 mL). The reaction mixture was stirred at 100 °C for 12 h, cooled to RT and mixed with ethyl acetate and water. After phase separation, the organic phase was washed with aqueous citric acid solution (10%) and brine, dried over anhydrous sodium sulfate, filtered and concentrated under reduced pressure. The residue was purified by flash silica gel chromatography (dichloromethane / methanol gradient) to give **168**. Yield: 456 mg (86% purity, 100% of theory). LC/MS (method 1): $t_R$ = 0.89 min, MS (ESIpos): *m/z* = 299 $[M+H]^+$; $^1$H NMR (400 MHz, DMSO-$d_6$): δ [ppm] = 12.58 (br s, 1H), 8.09 (s, 1H), 8.03 (d, *J* = 8.4 Hz, 1H), 7.77 (dd, *J* = 8.4 Hz, 2.1 Hz, 1H), 7.74 (d, *J* = 1.8 Hz, 1H), 6.51 (s, 1H).

**2-[4-(5-Chloro-2-cyanophenyl)-2-oxo-5-(trifluoromethyl)pyridin-1(2*H*)-yl]propanoic acid (racemate 169).**

A mixture of 4-chloro-2-[2-oxo-5-(trifluoromethyl)-1,2-dihydropyridin-4-yl]benzonitrile (**168**) (456 mg, 86% purity, 1.31 mmol), magnesium di-*tert*-butoxide (448 mg, 2.63 mmol, 2.0 eq.) and potassium *tert*-butylate (221 mg, 1.97 mmol, 1.5 eq.) in tetrahydrofuran (15 mL) was stirred under argon atmosphere at RT for 10 min and cooled with an ice bath, followed by addition of racemic 2-bromopropanoic acid (177 $\mu$L, 1.97 mmol, 1.5 eq.). The reaction mixture was stirred at RT for 2.5 h and at 50 °C overnight, mixed with water, ethyl acetate and aqueous hydrochloric acid solution. After extraction, the organic phase was concentrated under reduced pressure. The residue was dried *in vacuo* to give **169** which was used without further purification. Yield: 515 mg (51% purity, 54% of theory). LC/MS (method 1): $t_R$ = 0.91 min, MS (ESIpos): *m/z* = 371 $[M+H]^+$.

***tert*-Butyl 4-{2-[4-(5-chloro-2-cyanophenyl)-2-oxo-5-(trifluoromethyl)pyridin-1(2*H*)-yl]propanamido}benzoate (racemate 170).**

A solution of HATU (350 mg, 0.92 mmol, 1.3 eq.) in *N,N*-dimethylformamide (3 mL) was added under

argon atmosphere at RT to a solution of 2-[4-(5-chloro-2-cyanophenyl)-2-oxo-5-(trifluoromethyl)pyridin-1(2*H*)-yl]propanoic acid (racemate **169**) (515 mg, 51% purity, 0.71 mmol), *tert*-butyl 4-aminobenzoate (164 mg, 0.85 mmol, 1.2 eq.) and *N,N*-diisopropylethylamine (271 $\mu$L, 1.56 mmol, 2.2 eq.) in *N,N*-dimethylformamide (5 mL). The reaction mixture was stirred at RT for 1 h and concentrated under reduced pressure. After addition of ethyl acetate / water and phase separation, the aqueous phase was extracted with ethyl acetate. The combined organic phases were dried over anhydrous sodium sulfate, filtered and concentrated under reduced pressure. The residue was purified by flash silica gel chromatography (cyclohexane / ethyl acetate gradient) to give **170**. Yield: 251 mg (79% purity, 51% of theory). LC/MS (method 1): $t_R$ = 1.30 min, MS (ESIpos): *m*/*z* = 546 $[M+H]^+$.

**4-{2-[4-(5-Chloro-2-cyanophenyl)-2-oxo-5-(trifluoromethyl)pyridin-1(2*H*)-yl]propanamido}benzoic acid (racemate 23).**

Trifluoroacetic acid (560 $\mu$L, 7.26 mmol, 20 eq.) was added under argon atmosphere at RT to a mixture of *tert*-butyl 4-{2-[4-(5-chloro-2-cyanophenyl)-2-oxo-5-(trifluoromethyl)pyridin-1(2*H*)-yl]propanamido}benzoate (racemate **170**) (251 mg, 79% purity, 0.36 mmol) in dichloromethane (4 mL). The reaction mixture was stirred at RT for 3 h and concentrated under reduced pressure. The residue was purified without further work-up by preparative RP-HPLC (acetonitrile / water gradient) to give **23**. Yield: 35 mg (20% of theory). LC/MS (method 1): $t_R$ = 1.02 min, MS (ESIpos): *m*/*z* = 490 $[M+H]^+$; $^1$H NMR (400 MHz, DMSO-$d_6$): δ [ppm] = 12.80 (s, 1H), 10.82 / 10.78 (2x s, 1H), 8.33 (d, *J* = 8.7 Hz, 1H), 8.06 (dd, *J* = 8.4 Hz, 4.7 Hz, 1H), 7.92 (d, *J* = 8.4 Hz, 2H), 7.85-7.68 (m, 4H), 6.68 (d, *J* = 2.6 Hz, 1H), 5.68-5.57 (m, 1H), 1.79 (t, *J* = 6.9 Hz, 3H).

**Compounds of Table 3.**

**Synthesis of eutomer 25.**[a]

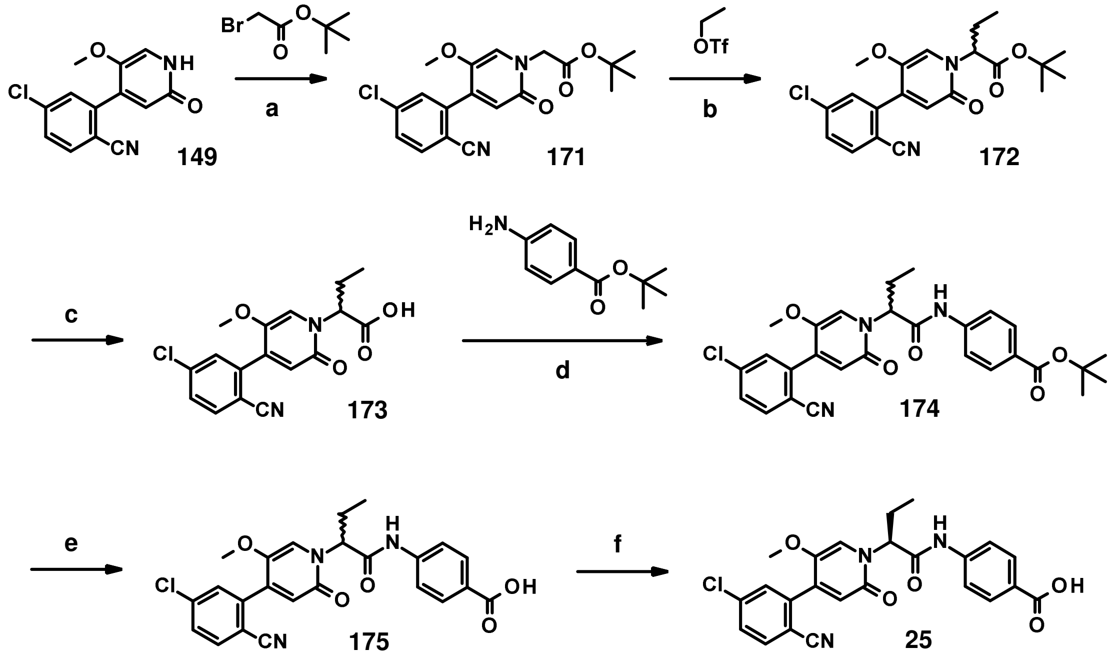

[a]Reagents and conditions: (a) $K_2CO_3$, DMF, 100 °C, 74%; (b) LiHMDS, THF, -78 °C → 0 °C, 78%; (c) TFA, DCM, RT, 83%; (d) HATU, DIEA, DMF, RT, 82%; (e) TFA, DCM, RT, 84%; (f) enantiomer separation.

***tert*-Butyl [4-(5-chloro-2-cyanophenyl)-5-methoxy-2-oxopyridin-1(2*H*)-yl]acetate (171).**

A mixture of 4-chloro-2-(5-methoxy-2-oxo-1,2-dihydropyridin-4-yl)benzonitrile (**149**) (3.00 g, 89% purity, 10.24 mmol), *tert*-butyl bromoacetate (1.85 mL, 12.29 mmol, 1.2 eq.) and potassium carbonate (2.12 g, 15.36 mmol, 1.5 eq.) in *N,N*-dimethylformamide (46 mL) was stirred under argon atmosphere at 100 °C for 45 min and concentrated under reduced pressure. After addition of ethyl acetate / water and phase separation, the aqueous phase was extracted with ethyl acetate. The combined organic phases were dried over anhydrous sodium sulfate, filtered, concentrated under reduced pressure and dried *in vacuo*. The residue was purified by flash silica gel chromatography (petroleum ether / ethyl acetate gradient) to give **171**. Yield: 2.84 g (74% of theory). LC/MS (method 1): $t_R$ = 0.97 min, MS (ESIpos): $m/z$ = 375 $[M+H]^+$; $^1H$ NMR (400 MHz, DMSO-$d_6$): δ [ppm] = 7.99 (d, $J$ = 8.1 Hz, 1H), 7.77-7.69 (m, 2H), 7.56 (s, 1H), 6.50 (s, 1H), 4.61 (s, 2H), 3.61 (s, 3H), 1.45 (s, 9H).

***tert*-Butyl 2-[4-(5-chloro-2-cyanophenyl)-5-methoxy-2-oxopyridin-1(2*H*)-yl]butanoate (racemate 172).**

Bis-(trimethylsilyl)-lithiumamide solution (1.0 M in tetrahydrofuran, 14.00 mL, 14.00 mmol, 1.05 eq.) was slowly added under argon atmosphere at -78 °C to a solution of *tert*-butyl [4-(5-chloro-2-cyanophenyl)-5-methoxy-2-oxopyridin-1(2*H*)-yl]acetate (**171**) (5.00 g, 13.34 mmol) in tetrahydrofuran

(100 mL). The reaction mixture was stirred at -78 °C for 15 min, mixed dropwise with ethyl trifluoromethanesulfonate (2.61 g, 14.67 mmol, 1.1 eq.) and stirred for 1 h while the cooling bath had been removed. The mixture was cooled to 0 °C and quenched with saturated aqueous ammonium chloride solution. After phase separation, the aqueous phase was extracted with methyl *tert*-butylether. The combined organic phases were dried over anhydrous sodium sulfate, filtered, concentrated under reduced pressure and dried *in vacuo*. The residue was purified by flash silica gel chromatography (cyclohexane / ethyl acetate gradient). The product was recrystallized from methyl *tert*-butylether to give **172**. Yield: 4.17 g (78% of theory). LC/MS (method 1): $t_R$ = 1.05 min, MS (ESIpos): *m*/*z* = 403 $[M+H]^+$; $^1$H NMR (400 MHz, DMSO-$d_6$): δ [ppm] = 7.99 (d, *J* = 8.8 Hz, 1H), 7.77-7.70 (m, 2H), 7.36 (s, 1H), 6.50 (s, 1H), 5.03 (dd, *J* = 9.0 Hz, 6.3 Hz, 1H), 3.64 (s, 3H), 2.19-2.06 (m, 2H), 1.40 (s, 9H), 0.85 (t, *J* = 7.4 Hz, 3H).

**2-[4-(5-Chloro-2-cyanophenyl)-5-methoxy-2-oxopyridin-1(2*H*)-yl]butanoic acid (racemate 173).**

Trifluoroacetic acid (7.84 mL, 101.77 mmol, 10 eq.) was added under argon atmosphere at RT to a mixture of *tert*-butyl 2-[4-(5-chloro-2-cyanophenyl)-5-methoxy-2-oxopyridin-1(2*H*)-yl]butanoate (racemate **172**) (4.10 g, 10.18 mmol) in dichloromethane (40 mL). The reaction mixture was stirred at RT for 1 h, mixed with additional trifluoroacetic acid (7.84 mL, 101.77 mmol, 10 eq.), stirred at RT for 1 h, mixed with additional trifluoroacetic acid (7.84 mL, 101.77 mmol, 10 eq.) and stirred at RT for 1 h. After complete conversion, the mixture was concentrated under reduced pressure. The residue was coevaporated three times with dichloromethane and once with toluene and dried *in vacuo*. The residue was dissolved in ethyl acetate and washed several times with a heavily diluted aqueous sodium hydrogen carbonate solution (pH value not more than 3-4, otherwise the desired product is soluble in water). The combined organic phases were dried over anhydrous sodium sulfate, filtered, concentrated under reduced pressure and dried *in vacuo*. The residue was stirred in methyl *tert*-butylether, and the solid was filtered, washed with methyl *tert*-butylether and dried *in vacuo* to give **173** which was used without further purification. Yield: 2.92 g (83% of theory). LC/MS (method 1): $t_R$ = 0.81 min, MS (ESIpos): *m*/*z* = 347 $[M+H]^+$; $^1$H NMR (400 MHz, DMSO-$d_6$): δ [ppm] = 12.97 (s, 1H), 7.99 (d, *J* = 8.3 Hz, 1H), 7.77-7.70 (m, 2H), 7.41 (s, 1H), 6.49 (s, 1H), 5.09 (dd, *J* = 9.3 Hz, 6.2 Hz, 1H), 3.64 (s, 3H), 2.21-2.09 (m, 2H), 0.84 (t, *J* = 7.4 Hz, 3H).

***tert*-Butyl 4-{2-[4-(5-chloro-2-cyanophenyl)-5-methoxy-2-oxopyridin-1(2*H*)-yl]butanamido}benzoate (racemate 174).**

A solution of HATU (72 mg, 0.19 mmol, 1.2 eq.) in *N,N*-dimethylformamide (2 mL) was added under argon atmosphere at RT to a solution of 2-[4-(5-chloro-2-cyanophenyl)-5-methoxy-2-oxopyridin-1(2*H*)-yl]butanoic acid (racemate **173**) (55 mg, 0.16 mmol), *tert*-butyl 4-aminobenzoate (34 mg, 0.17 mmol, 1.1 eq.) and *N,N*-diisopropylethylamine (61 μL, 0.35 mmol, 2.2 eq.) in *N,N*-dimethylformamide (6 mL). The reaction mixture was stirred at RT for 1 h and concentrated under

reduced pressure. The residue was purified by preparative RP-HPLC (acetonitrile / water gradient) to give **174**. Yield: 68 mg (82% of theory). LC/MS (method 1): $t_R$ = 1.23 min, MS (ESIpos): *m*/*z* = 522 $[M+H]^+$.

**4-{2-[4-(5-Chloro-2-cyanophenyl)-5-methoxy-2-oxopyridin-1(2*H*)-yl]butanamido}benzoic acid (racemate 175).**

Trifluoroacetic acid (201 µL, 2.61 mmol, 20 eq.) was added under argon atmosphere at RT to a mixture of *tert*-butyl 4-{2-[4-(5-chloro-2-cyanophenyl)-5-methoxy-2-oxopyridin-1(2*H*)-yl]butanamido}-benzoate (racemate **174**) (68 mg, 0.13 mmol) in dichloromethane (5 mL). The reaction mixture was stirred at RT for 4 h and concentrated under reduced pressure. The residue was coevaporated with dichloromethane and purified by preparative RP-HPLC (acetonitrile / water gradient) to give **175**. Yield: 51 mg (84% of theory). LC/MS (method 1): $t_R$ = 0.94 min, MS (ESIpos): *m*/*z* = 466 $[M+H]^+$; $^1H$ NMR (400 MHz, DMSO-$d_6$): δ [ppm] = 12.78 (s, 1H), 10.81 (s, 1H), 8.00 (d, *J* = 8.3 Hz, 1H), 7.92 (d, *J* = 8.7 Hz, 2H), 7.79-7.71 (m, 4H), 7.50 (s, 1H), 6.54 (s, 1H), 5.64 (dd, *J* = 9.4 Hz, 6.5 Hz, 1H), 3.69 (s, 3H), 2.25-2.11 (m, 2H), 0.91 (t, *J* = 7.2 Hz, 3H).

**4-({(2*S*)-2-[4-(5-Chloro-2-cyanophenyl)-5-methoxy-2-oxopyridin-1(2*H*)-yl]butanoyl}amino)benzoic acid (eutomer 25).**

Enantiomer separation of 433 mg of 4-{2-[4-(5-chloro-2-cyanophenyl)-5-methoxy-2-oxopyridin-1(2*H*)-yl]butanamido}benzoic acid (racemate **175**) gave 196 mg of distomer (chiral HPLC: $t_R$ = 5.22 min) and 201 mg of eutomer **25** (chiral HPLC: $t_R$ = 8.19 min, 99% ee).

LC/MS (method 1): $t_R$ = 0.92 min, MS (ESIpos): *m*/*z* = 466 $[M+H]^+$; $^1H$ NMR (400 MHz, DMSO-$d_6$): δ [ppm] = 12.77 (s, 1H), 10.81 (s, 1H), 8.00 (d, *J* = 8.1 Hz, 1H), 7.91 (d, *J* = 8.8 Hz, 2H), 7.79-7.71 (m, 4H), 7.50 (s, 1H), 6.54 (s, 1H), 5.64 (dd, *J* = 9.4 Hz, 6.6 Hz, 1H), 3.69 (s, 3H), 2.26-2.11 (m, 2H), 0.91 (t, *J* = 7.2 Hz, 3H).

Separation method: column: Daicel Chiralpak IF 5 µm, 250 mm × 20 mm; mobile phase: 50% *iso*-hexane / 50% ethanol plus 2% acetic acid; temperature: 40 °C; flow rate: 15 mL/min; UV detection: 220 nm.

Analytical method: column: Daicel Chiralpak AZ-H 5 µm, 250 mm × 4.6 mm; mobile phase: 50% *iso*-hexane / 50% ethanol plus 0.2% trifluoroacetic acid in 1% water; temperature: 40 °C; flow rate: 1 mL/min; UV detection: 220 nm.

**Synthesis of racemate 26.**[a]

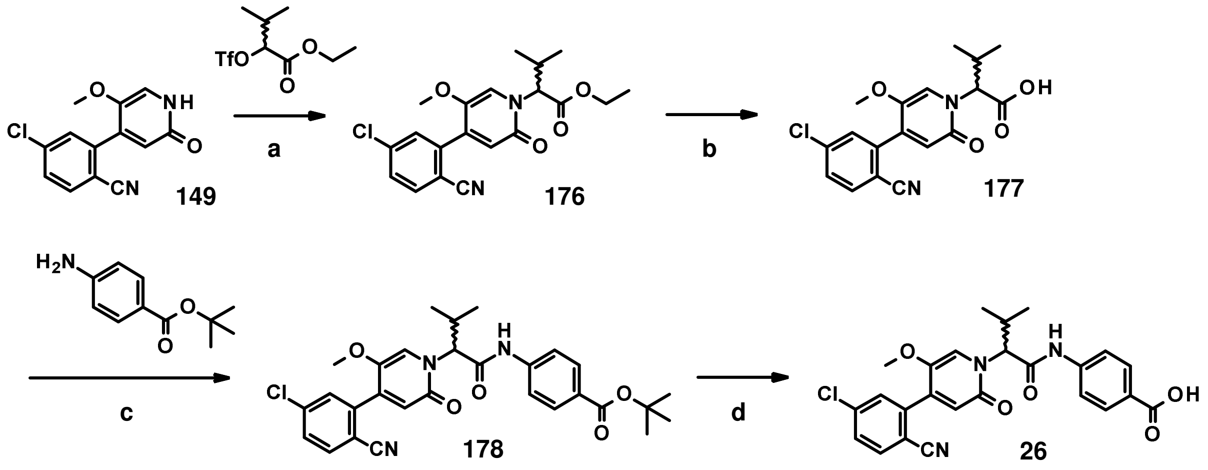

[a]Reagents and conditions: (a) NaH, THF, RT, 66%; (b) LiOH, THF/water, 82%; (c) HATU, DIEA, DMF, RT, 75%; (d) TFA, DCM, RT, 33%.

**Ethyl 2-[4-(5-chloro-2-cyanophenyl)-5-methoxy-2-oxopyridin-1(2*H*)-yl]-3-methylbutanoate (racemate 176).**

Sodium hydride (509 mg, 20.14 mmol, 1.5 eq.) was added under argon atmosphere at RT to a solution of 4-chloro-2-(5-methoxy-2-oxo-1,2-dihydropyridin-4-yl)benzonitrile (**149**) (3.50 g, 13.43 mmol) in tetrahydrofuran (130 mL). The reaction mixture was stirred at RT for 2.5 h, mixed with a solution of 3-methyl-2-{[(trifluoromethyl)sulfonyl]oxy}butanoate (5.60 g, 20.14 mmol, 1.5 eq.) in tetrahydrofuran (20 mL), stirred for at RT for 1.5 h and quenched with aqueous ammonium chloride solution. The aqueous phase was extracted with methyl *tert*-butyl ether. The combined organic phases were washed with brine, dried over anhydrous sodium sulfate, filtered, concentrated under reduced pressure and dried *in vacuo*. The residue was purified by flash silica gel chromatography (cyclohexane / ethyl acetate gradient) to give **176**. Yield: 3.70 g (66% of theory). LC/MS (method 1): $t_R$ = 1.08 min, MS (ESIpos): *m*/*z* = 389 $[M+H]^+$.

**2-[4-(5-Chloro-2-cyanophenyl)-5-methoxy-2-oxopyridin-1(2*H*)-yl]-3-methylbutanoic acid (racemate 177).**

Lithium hydroxide (342 mg, 14.27 mmol, 1.5 eq.) was added at RT to a mixture of ethyl 2-[4-(5-chloro-2-cyanophenyl)-5-methoxy-2-oxopyridin-1(2*H*)-yl]-3-methylbutanoate (racemate **176**) (3.70 g, 9.52 mmol) in a mixture of tetrahydrofuran and water (3:1, 120 mL). The reaction mixture was stirred at RT for 45 min and acidified to pH 1 with aqueous hydrochloric acid solution (1 N). Tetrahydrofuran was removed. The residue was crystallized from water, and the solid was filtered and dried *in vacuo* to give **177**. Yield: 2.80 g (82% of theory). LC/MS (method 1): $t_R$ = 0.89 min, MS (ESIpos): *m*/*z* = 361 $[M+H]^+$; $^1H$ NMR (400 MHz, DMSO-$d_6$): δ [ppm] = 13.1 (br s, 1H), 7.99 (d, *J* = 8.3 Hz, 1H), 7.76 (d, *J* = 1.8 Hz, 1H), 7.73 (dd, *J* = 8.1 Hz, 2.1 Hz, 1H), 7.41 (s, 1H), 6.51 (s, 1H), 4.94 (d, *J* = 10.0 Hz, 1H),

3.64 (s, 3H), 2.62-2.56 (m, 1H), 1.12 (d, $J$ = 6.5 Hz, 3H), 0.75 (d, $J$ = 6.7 Hz, 3H).

***tert*-Butyl 4-{2-[4-(5-chloro-2-cyanophenyl)-5-methoxy-2-oxopyridin-1(2*H*)-yl]-3-methylbutan-amido}benzoate (racemate 178).**

A solution of HATU (152 mg, 0.40 mmol, 1.2 eq.) in *N,N*-dimethylformamide (0.5 mL) was added under argon atmosphere at RT to a solution of 2-[4-(5-chloro-2-cyanophenyl)-5-methoxy-2-oxopyridin-1(2*H*)-yl]-3-methylbutanoic acid (racemate **177**) (120 mg, 0.33 mmol), *tert*-butyl 4-aminobenzoate (71 mg, 0.37 mmol, 1.1 eq.) and *N,N*-diisopropylethylamine (127 $\mu$L, 0.73 mmol, 2.2 eq.) in *N,N*-dimethylformamide (2 mL). The reaction mixture was stirred at RT overnight and mixed with ethyl acetate and water. After phase separation, the aqueous phase was extracted with ethyl acetate. The combined organic phases were washed with brine, dried over anhydrous sodium sulfate, filtered, concentrated under reduced pressure and dried *in vacuo*. The residue was purified by flash silica gel chromatography (cyclohexane / ethyl acetate gradient) to give **178**. Yield: 134 mg (75% of theory). LC/MS (method 1): $t_R$ = 1.29 min, MS (ESIpos): $m/z$ = 536 $[M+H]^+$; $^1$H NMR (400 MHz, DMSO-$d_6$): δ [ppm] = 11.0 (s, 1H), 8.00 (d, $J$ = 8.3 Hz, 1H), 7.88 (d, $J$ = 8.9 Hz, 2H), 7.78 (d, $J$ = 8.8 Hz, 2H), 7.76 (d, $J$ = 1.7 Hz, 1H), 7.73 (dd, $J$ = 8.3 Hz, 2.2 Hz, 1H), 7.63 (s, 1H), 6.56 (s, 1H), 5.52 (d, $J$ = 11.1 Hz, 1H), 3.69 (s, 3H), 2.61-2.55 (m, 1H), 1.54 (s, 9H), 1.08 (d, $J$ = 6.5 Hz, 3H), 0.82 (d, $J$ = 6.5 Hz, 3H).

**4-{2-[4-(5-Chloro-2-cyanophenyl)-5-methoxy-2-oxopyridin-1(2*H*)-yl]-3-methylbutanamido}-benzoic acid (racemate 26).**

Trifluoroacetic acid (273 $\mu$L, 3.55 mmol, 20 eq.) was added under argon atmosphere at RT to a mixture of *tert*-butyl 4-{2-[4-(5-chloro-2-cyanophenyl)-5-methoxy-2-oxopyridin-1(2*H*)-yl]-3-methylbutanamido}benzoate (racemate **178**) (95 mg, 0.18 mmol) in dichloromethane (8 mL). The reaction mixture was stirred at RT for 4 h and concentrated under reduced. The residue was stirred in a mixture of methyl *tert*-butyl ether and pentane, and the solid was filtered and dried *in vacuo*. The residue was purified by preparative RP-HPLC (acetonitrile / 0.1% aqueous ammonium formate solution gradient) to give **26**. Yield: 28 mg (33% of theory). LC/MS (method 1): $t_R$ = 1.00 min, MS (ESIpos): $m/z$ = 480 $[M+H]^+$; $^1$H NMR (400 MHz, DMSO-$d_6$): δ [ppm] = 11.0 (s, 1H), 8.00 (d, $J$ = 8.3 Hz, 1H), 7.89 (d, $J$ = 8.7 Hz, 2H), 7.78-7.71 (m, 4H), 7.65 (s, 1H), 6.56 (s, 1H), 5.52 (d, $J$ = 11.1 Hz, 1H), 3.69 (s, 3H), 1.08 (d, $J$ = 6.5 Hz, 3H), 0.82 (d, $J$ = 6.5 Hz, 3H).

**Synthesis of eutomer 27.**[a]

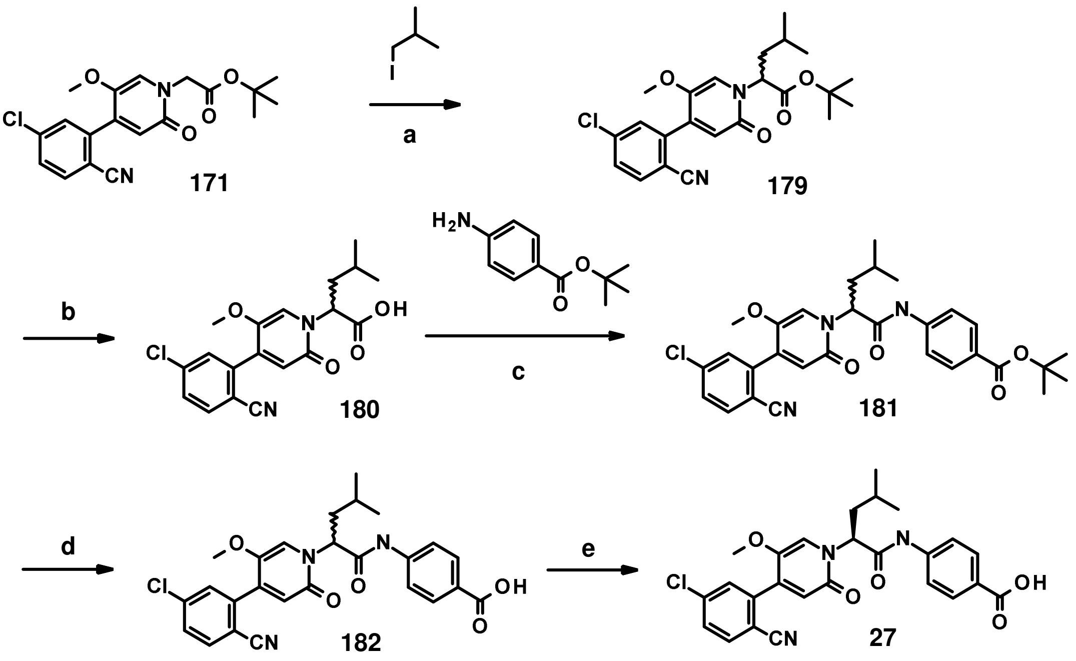

[a]Reagents and conditions: (a) LiHMDS, THF, -78 °C → 0 °C, 48%; (b) TFA, DCM, RT, 98%; (c) HATU, DIEA, DMF, RT, 60%; (d) TFA, DCM, RT, 71%; (e) enantiomer separation.

***tert*-Butyl 2-[4-(5-chloro-2-cyanophenyl)-5-methoxy-2-oxopyridin-1(2*H*)-yl]-4-methylpentanoate (racemate 179).**

Bis-(trimethylsilyl)-lithiumamide solution (1.0 M in tetrahydrofuran, 0.88 mL, 0.88 mmol, 1.1 eq.) was slowly added under argon atmosphere at -78 °C to a solution of *tert*-butyl [4-(5-chloro-2-cyanophenyl)-5-methoxy-2-oxopyridin-1(2*H*)-yl]acetate (**171**) (309 mg, 0.80 mmol) in tetrahydrofuran (12 mL). The reaction mixture was stirred at -78 °C for 10 min and mixed dropwise with a solution of 1-iodo-2-methylpropane (191 mg, 1.04 mmol, 1.3 eq.) in tetrahydrofuran (2 mL). The mixture was stirred at -78 °C for 10 min, under ice bath cooling for 2.5 h and quenched with water. The mixture was extracted with ethyl acetate. The combined organic phases were dried over anhydrous sodium sulfate, filtered, concentrated under reduced pressure and dried *in vacuo*. The residue was purified by flash silica gel chromatography (cyclohexane / ethyl acetate gradient) to give **179**. Yield: 178 mg (92% purity, 48% of theory). LC/MS (method 1): $t_R$ = 1.25 min, MS (ESIpos): *m*/*z* = 431 $[M+H]^+$.

**2-[4-(5-Chloro-2-cyanophenyl)-5-methoxy-2-oxopyridin-1(2*H*)-yl]-4-methylpentanoic acid (racemate 180).**

Trifluoroacetic acid (586 µL, 7.60 mmol, 20 eq.) was added under argon atmosphere at RT to a mixture of *tert*-butyl 2-[4-(5-chloro-2-cyanophenyl)-5-methoxy-2-oxopyridin-1(2*H*)-yl]-4-methylpentanoate (racemate **179**) (178 mg, 92% purity, 0.38 mmol) in dichloromethane (5 mL). The reaction mixture was stirred at RT for 4 h and concentrated under reduced pressure. The residue was coevaporated several

times with dichloromethane, dried *in vacuo* and purified by preparative RP-HPLC (acetonitrile / water gradient) to give **180**. Yield: 165 mg (85% purity, 98% of theory). LC/MS (method 1): $t_R$ = 0.95 min, MS (ESIpos): *m/z* = 375 $[M+H]^+$.

**_tert_-Butyl 4-({2-[4-(5-chloro-2-cyanophenyl)-5-methoxy-2-oxopyridin-1(2*H*)-yl]-4-methylpentanoyl}amino)benzoate (racemate 181).**

A solution of HATU (172 mg, 0.45 mmol, 1.2 eq.) in *N,N*-dimethylformamide (2 mL) was added under argon atmosphere at RT to a solution of 2-[4-(5-chloro-2-cyanophenyl)-5-methoxy-2-oxopyridin-1(2*H*)-yl]-4-methylpentanoic acid (racemate **180**) (166 mg, 85% purity, 0.38 mmol), *tert*-butyl 4-aminobenzoate (80 mg, 0.41 mmol, 1.1 eq.) and *N,N*-diisopropylethylamine (144 µL, 0.83 mmol, 2.2 eq.) in *N,N*-dimethylformamide (6 mL). The reaction mixture was stirred at RT for 8 h and concentrated under reduced pressure. The residue was stirred in water, and the solid was filtered, washed with water, dried *in vacuo* and purified by preparative RP-HPLC (acetonitrile / water gradient) to give **181**. Yield: 127 mg (60% of theory). LC/MS (method 1): $t_R$ = 1.33 min, MS (ESIpos): *m/z* = 550 $[M+H]^+$.

**4-({2-[4-(5-Chloro-2-cyanophenyl)-5-methoxy-2-oxopyridin-1(2*H*)-yl]-4-methylpentanoyl}amino)benzoic acid (racemate 182).**

Trifluoroacetic acid (349 µL, 4.53 mmol, 20 eq.) was added under argon atmosphere at RT to a mixture of *tert*-butyl 4-({2-[4-(5-chloro-2-cyanophenyl)-5-methoxy-2-oxopyridin-1(2*H*)-yl]-4-methylpentanoyl}amino)benzoate (racemate **181**) (127 mg, 0.23 mmol) in dichloromethane (4 mL). The reaction mixture was stirred at RT for 4 h and concentrated under reduced pressure. The residue was coevaporated several times with dichloromethane, dried *in vacuo* and purified by preparative RP-HPLC (acetonitrile / water gradient) to give **182**. Yield: 79 mg (71% of theory). LC/MS (method 1): $t_R$ = 1.07 min, MS (ESIpos): *m/z* = 494 $[M+H]^+$; $^1$H NMR (400 MHz, DMSO-$d_6$): δ [ppm] = 12.78 (s, 1H), 10.85 (s, 1H), 8.00 (d, *J* = 8.3 Hz, 1H), 7.92 (d, *J* = 8.8 Hz, 2H), 7.79-7.71 (m, 4H), 7.50 (s, 1H), 6.54 (s, 1H), 5.90-5.82 (m, 1H), 3.69 (s, 3H), 2.27-2.17 (m, 1H), 1.93-1.84 (m, 1H), 1.49-1.39 (m, 1H), 0.95 (t, *J* = 6.5 Hz, 6H).

**4-({(2*S*)-2-[4-(5-Chloro-2-cyanophenyl)-5-methoxy-2-oxopyridin-1(2*H*)-yl]-4-methylpentanoyl}amino)benzoic acid (eutomer 27).**

Enantiomer separation of 73 mg of 4-({2-[4-(5-chloro-2-cyanophenyl)-5-methoxy-2-oxopyridin-1(2*H*)-yl]-4-methylpentanoyl}amino)benzoic acid (racemate **182**) gave 37 mg of distomer (chiral HPLC: $t_R$ = 4.6 min) and 33 mg of eutomer **27** (chiral HPLC: $t_R$ = 7.0 min, 99% ee).
LC/MS (method 1): $t_R$ = 1.06 min, MS (ESIpos): *m/z* = 494 $[M+H]^+$; $^1$H NMR (400 MHz, DMSO-$d_6$): δ [ppm] = 12.78 (s, 1H), 10.85 (s, 1H), 8.00 (d, *J* = 8.2 Hz, 1H), 7.92 (d, *J* = 8.7 Hz, 2H), 7.81-7.70 (m, 4H), 7.50 (s, 1H), 6.54 (s, 1H), 5.91-5.82 (m, 1H), 3.69 (s, 3H), 2.27-2.16 (m, 1H), 1.94-1.83 (m, 1H), 1.50-1.38 (m, 1H), 0.95 (t, *J* = 6.5 Hz, 6H).

Separation method: column: Daicel Chiralpak IF 5 μm, 250 mm × 20 mm; mobile phase: 50% *iso*-hexane / 50% ethanol; temperature: 40 °C; flow rate: 15 mL/min; UV detection: 220 nm.
Analytical method: column: Daicel Chiralpak AZ-H 5 μm, 250 mm × 4.6 mm; mobile phase: 50% *iso*-hexane / 50% ethanol plus 0.2% trifluoroacetic acid in 1% water; temperature: 40 °C; flow rate: 1 mL/min; UV detection: 220 nm.

**Synthesis of eutomer 28.**[a]

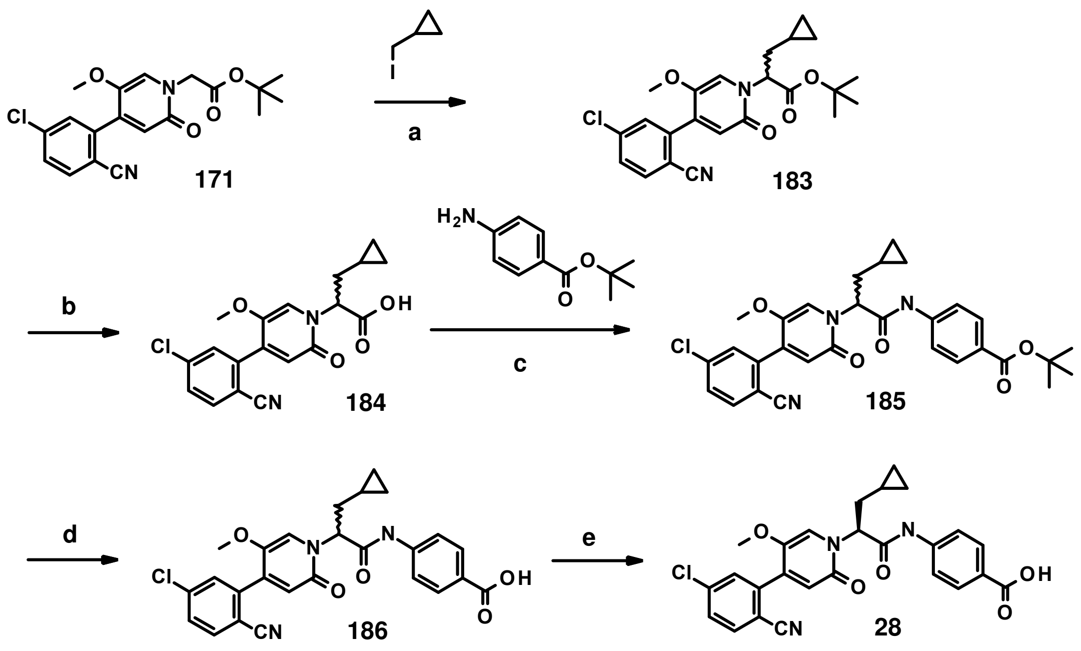

[a]Reagents and conditions: (a) LiHMDS, THF, -78 °C → 0 °C, 71%; (b) TFA, DCM, RT, 100%; (c) HATU, DIEA, DMF, RT, 61%; (d) TFA, DCM, RT, 61%; (e) enantiomer separation.

***tert*-Butyl 2-[4-(5-chloro-2-cyanophenyl)-5-methoxy-2-oxopyridin-1(2*H*)-yl]-3-cyclopropylpropanoate (racemate 183).**

Bis-(trimethylsilyl)-lithiumamide solution (1.0 M in tetrahydrofuran, 1.36 mL, 1.36 mmol, 1.1 eq.) was added under argon atmosphere at -78 °C to a solution of *tert*-butyl [4-(5-chloro-2-cyanophenyl)-5-methoxy-2-oxopyridin-1(2*H*)-yl]acetate (**171**) (464 mg, 1.24 mmol) in tetrahydrofuran (13 mL). The reaction mixture was stirred at -78 °C for 10 min and mixed with a solution of (iodomethyl)cyclopropane (293 mg, 1.61 mmol, 1.3 eq.) in tetrahydrofuran (2 mL). The mixture was stirred at -78 °C for 10 min, under ice bath cooling for 1 h and quenched with water. The mixture was extracted with ethyl acetate. The combined organic phases were dried over anhydrous sodium sulfate, filtered, concentrated under reduced pressure and dried *in vacuo*. The residue was purified by flash silica gel chromatography (cyclohexane / ethyl acetate gradient) to give **183**. Yield: 379 mg (71% of theory). LC/MS (method 1): $t_R$ = 1.18 min, MS (ESIpos): $m/z$ = 429 $[M+H]^+$.

**2-[4-(5-Chloro-2-cyanophenyl)-5-methoxy-2-oxopyridin-1(2*H*)-yl]-3-cyclopropylpropanoic acid (racemate 184).**

Trifluoroacetic acid (1.36 mL, 17.63 mmol, 20 eq.) was added under argon atmosphere at RT to a mixture of *tert*-butyl 2-[4-(5-chloro-2-cyanophenyl)-5-methoxy-2-oxopyridin-1(2*H*)-yl]-3-cyclopropylpropanoate (racemate **183**) (378 mg, 0.88 mmol) in dichloromethane (5 mL). The reaction mixture was stirred at RT for 4 h and concentrated under reduced pressure. The residue was coevaporated several times with dichloromethane and dried *in vacuo* to give **184** which was used without further purification. Yield: 420 mg (92% purity, 100% of theory). LC/MS (method 1): $t_R$ = 0.90 min, MS (ESIpos): *m*/*z* = 373 $[M+H]^+$.

***tert*-Butyl 4-{2-[4-(5-chloro-2-cyanophenyl)-5-methoxy-2-oxopyridin-1(2*H*)-yl]-3-cyclopropylpropanamido}benzoate (racemate 185).**

A solution of HATU (512 mg, 1.35 mmol, 1.3 eq.) in *N,N*-dimethylformamide (3 mL) was added under argon atmosphere at RT to a solution of 2-[4-(5-chloro-2-cyanophenyl)-5-methoxy-2-oxopyridin-1(2*H*)-yl]-3-cyclopropylpropanoic acid (racemate **184**) (420 mg, 92% purity, 1.04 mmol), *tert*-butyl 4-aminobenzoate (240 mg, 1.24 mmol, 1.2 eq.) and *N,N*-diisopropylethylamine (397 $\mu$L, 2.28 mmol, 2.2 eq.) in *N,N*-dimethylformamide (5 mL). The reaction mixture was stirred at RT for 1 h and concentrated under reduced pressure. The residue was mixed with ethyl acetate and water. After phase separation, the aqueous phase was extracted with ethyl acetate. The combined organic phases were dried over anhydrous sodium sulfate, filtered, concentrated under reduced pressure and dried *in vacuo*. The residue was purified by flash silica gel chromatography (cyclohexane / ethyl acetate gradient) to give **185**. Yield: 348 mg (61% of theory). LC/MS (method 1): $t_R$ = 1.29 min, MS (ESIpos): *m*/*z* = 548 $[M+H]^+$.

**4-{2-[4-(5-Chloro-2-cyanophenyl)-5-methoxy-2-oxopyridin-1(2*H*)-yl]-3-cyclopropylpropan-amido}benzoic acid (racemate 186).**

Trifluoroacetic acid (448 $\mu$L, 5.82 mmol, 20 eq.) was added under argon atmosphere at RT to a mixture of *tert*-butyl 4-{2-[4-(5-chloro-2-cyanophenyl)-5-methoxy-2-oxopyridin-1(2*H*)-yl]-3-cyclopropylpropanamido}benzoate (racemate **185**) (161 mg, 0.29 mmol) in dichloromethane (5 mL). The reaction mixture was stirred at RT for 3.5 h and concentrated under reduced pressure. The residue was coevaporated several times with dichloromethane, dried *in vacuo* and purified by preparative RP-HPLC (acetonitrile / water gradient) to give **186**. Yield: 88 mg (61% of theory). LC/MS (method 1): $t_R$ = 1.02 min, MS (ESIpos): *m*/*z* = 492 $[M+H]^+$.

**4-({(2*S*)-2-[4-(5-Chloro-2-cyanophenyl)-5-methoxy-2-oxopyridin-1(2*H*)-yl]-3-cyclopropylpropanoyl}amino)benzoic acid (eutomer 28).**

Enantiomer separation of 138 mg of 4-{2-[4-(5-chloro-2-cyanophenyl)-5-methoxy-2-oxopyridin-1(2*H*)-yl]-3-cyclopropylpropanamido}benzoic acid (racemate **186**) gave 56 mg of distomer (chiral HPLC: $t_R$ = 4.1 min) and 52 mg of eutomer **28** (chiral HPLC: $t_R$ = 5.4 min, 98% ee).

LC/MS (method 1): $t_R$ = 1.01 min, MS (ESIpos): $m/z$ = 492 $[M+H]^+$; $^1H$ NMR (400 MHz, DMSO-$d_6$): δ [ppm] = 12.77 (br s, 1H), 10.79 (s, 1H), 8.00 (d, $J$ = 9.0 Hz, 1H), 7.91 (d, $J$ = 8.8 Hz, 2H), 7.78-7.71 (m, 4H), 7.54 (s, 1H), 6.53 (s, 1H), 5.80 (dd, $J$ = 9.5 Hz, 6.1 Hz, 1H), 3.70 (s, 3H), 2.28-2.18 (m, 1H), 1.96-1.87 (m, 1H), 0.71-0.61 (m, 1H), 0.47-0.33 (m, 2H), 0.23-0.10 (m, 2H).

Separation method: column: Daicel Chiralpak IF 5 µm, 250 mm × 20 mm; mobile phase: 25% *iso*-hexane / 75% ethanol; temperature: 45 °C; flow rate: 15 mL/min; UV detection: 220 nm.

Analytical method: column: Daicel Chiralpak IF 5 µm, 250 mm × 4.6 mm; mobile phase: 25% *iso*-hexane / 75% ethanol plus 0.2% trifluoroacetic acid in 1% water; temperature: 30 °C; flow rate: 1 mL/min; UV detection: 220 nm.

**Synthesis of eutomer 29.**[a]

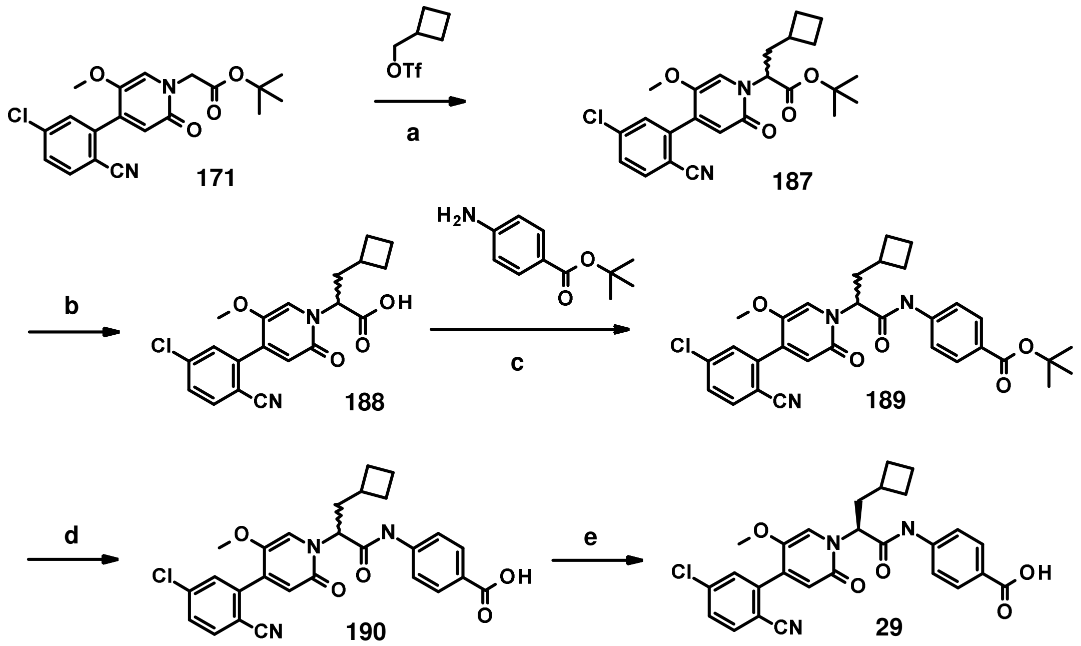


[a]Reagents and conditions: (a) LiHMDS, THF, -78 °C → RT, 89%; (b) TFA, DCM, RT, 100%; (c) HATU, DIEA, DMF, RT, 65%; (d) TFA, DCM, RT, 83%; (e) enantiomer separation.

### ***tert*-Butyl 2-[4-(5-chloro-2-cyanophenyl)-5-methoxy-2-oxopyridin-1(2*H*)-yl]-3-cyclobutylpropanoate (racemate 187).**

Bis-(trimethylsilyl)-lithiumamide solution (1.0 M in tetrahydrofuran, 3.17 mL, 3.17 mmol, 1.2 eq.) was added under argon atmosphere at -78 °C to a solution of *tert*-butyl [4-(5-chloro-2-cyanophenyl)-5-methoxy-2-oxopyridin-1(2*H*)-yl]acetate (**171**) (1.00 g, 2.64 mmol) in tetrahydrofuran (20 mL). The reaction mixture was stirred at -78 °C for 30 min and mixed with cyclobutylmethyl trifluoromethanesulfonate (1.22 g, 80% purity, 4.49 mmol, 1.7 eq.). The mixture was stirred at -78 °C for 30 min, under ice bath cooling for 15 min and allowed to warm to RT. The mixture was quenched

with water and extracted with ethyl acetate. The combined organic phases were washed with brine, dried over anhydrous sodium sulfate, filtered, concentrated under reduced pressure and dried *in vacuo* to give **187** which was used without further purification. Yield: 1.22 g (85% purity, 89% of theory). LC/MS (method 1): $t_R$ = 1.20 min, MS (ESIpos): *m*/*z* = 443 $[M+H]^+$.

**2-[4-(5-Chloro-2-cyanophenyl)-5-methoxy-2-oxopyridin-1(2*H*)-yl]-3-cyclobutylpropanoic acid (racemate 188).**

Trifluoroacetic acid (3.62 mL, 47.00 mmol, 20 eq.) was added under argon atmosphere at 0 °C to a mixture of *tert*-butyl 2-[4-(5-chloro-2-cyanophenyl)-5-methoxy-2-oxopyridin-1(2*H*)-yl]-3-cyclobutylpropanoate (racemate **187**) (1.22 g, 2.35 mmol) in dichloromethane (15 mL). The reaction mixture was stirred at RT for 7 h and concentrated under reduced pressure. The residue was coevaporated with toluene and dried *in vacuo*. The residue was redissolved in dichloromethane (15 mL), mixed at 0 °C with trifluoroacetic acid (3.62 mL, 47.00 mmol, 20 eq.), stirred at RT for 5.5 h and concentrated under reduced pressure. The residue was coevaporated twice with toluene and dried *in vacuo* to give **188** which was used without further purification. Yield: 1.22 g (78% purity, 100% of theory). LC/MS (method 2): $t_R$ = 2.13 min, MS (ESIpos): *m*/*z* = 387 $[M+H]^+$.

***tert*-Butyl 4-{2-[4-(5-chloro-2-cyanophenyl)-5-methoxy-2-oxopyridin-1(2*H*)-yl]-3-cyclobutylpropanamido}benzoate (racemate 189).**

A solution of HATU (1.13 g, 2.97 mmol, 1.2 eq.) in *N,N*-dimethylformamide (5 mL) was added under argon atmosphere at RT to a solution of 2-[4-(5-chloro-2-cyanophenyl)-5-methoxy-2-oxopyridin-1(2*H*)-yl]-3-cyclobutylpropanoic acid (racemate **188**) (1.23 g, 78% purity, 2.47 mmol), *tert*-butyl 4-aminobenzoate (526 mg, 2.72 mmol, 1.1 eq.) and *N,N*-diisopropylethylamine (948 $\mu$L, 5.44 mmol, 2.2 eq.) in *N,N*-dimethylformamide (15 mL). The reaction mixture was stirred at RT overnight and concentrated under reduced pressure. The residue was mixed with ethyl acetate and water. After phase separation, the aqueous phase was extracted with ethyl acetate. The combined organic phases were washed with brine, dried over anhydrous sodium sulfate, filtered, concentrated under reduced pressure and dried *in vacuo*. The residue was purified by flash silica gel chromatography (cyclohexane / ethyl acetate gradient) to give **189**. Yield: 947 mg (65% of theory). LC/MS (method 1): $t_R$ = 1.31 min, MS (ESIpos): *m*/*z* = 562 $[M+H]^+$.

**4-{2-[4-(5-Chloro-2-cyanophenyl)-5-methoxy-2-oxopyridin-1(2*H*)-yl]-3-cyclobutylpropanamido}benzoic acid (racemate 190).**

Trifluoroacetic acid (2.49 mL, 32.36 mmol, 20 eq.) was added under argon atmosphere at 0 °C to a mixture of *tert*-butyl 4-{2-[4-(5-chloro-2-cyanophenyl)-5-methoxy-2-oxopyridin-1(2*H*)-yl]-3-cyclobutylpropanamido}benzoate (racemate **189**) (947 mg, 1.62 mmol) in dichloromethane (15 mL). The reaction mixture was allowed to warm to RT, stirred at RT for 2.5 h and concentrated under reduced pressure. The residue was coevaporated several times with toluene, dried *in vacuo* and purified by flash

silica gel chromatography (cyclohexane / ethyl acetate gradient) to give **190**. Yield: 688 mg (83% of theory). LC/MS (method 1): $t_R$ = 1.02 min, MS (ESIpos): $m/z$ = 506 $[M+H]^+$; $^1H$ NMR (400 MHz, DMSO-$d_6$): δ [ppm] = 12.77 (s, 1H), 10.80 (s, 1H), 8.00 (d, $J$ = 9.1 Hz, 1H), 7.91 (d, $J$ = 8.7 Hz, 2H), 7.80-7.70 (m, 4H), 7.51 (s, 1H), 6.51 (s, 1H), 5.75-5.65 (m, 1H), 3.69 (s, 3H), 2.35-2.16 (m, 3H), 2.01-1.88 (m, 2H), 1.85-1.60 (m, 4H).

**4-({(2*S*)-2-[4-(5-Chloro-2-cyanophenyl)-5-methoxy-2-oxopyridin-1(2*H*)-yl]-3-cyclobutylpropanoyl}amino)benzoic acid (eutomer 29).**

Enantiomer separation of 150 mg of 4-{2-[4-(5-chloro-2-cyanophenyl)-5-methoxy-2-oxopyridin-1(2*H*)-yl]-3-cyclobutylpropanamido}benzoic acid (racemate **190**) gave 38 mg of distomer (chiral HPLC: $t_R$ = 0.99 min) and 49 mg of eutomer **29** (chiral HPLC: $t_R$ = 1.85 min, >99% ee).
LC/MS (method 3): $t_R$ = 1.90 min, MS (ESIpos): $m/z$ = 506 $[M+H]^+$; $^1H$ NMR (400 MHz, DMSO-$d_6$): δ [ppm] = 12.77 (s, 1H), 10.80 (s, 1H), 8.00 (d, $J$ = 9.0 Hz, 1H), 7.91 (d, $J$ = 8.7 Hz, 2H), 7.80-7.70 (m, 4H), 7.51 (s, 1H), 6.51 (s, 1H), 5.75-5.65 (m, 1H), 3.69 (s, 3H), 2.35-2.16 (m, 3H), 2.01-1.88 (m, 2H), 1.85-1.60 (m, 4H).
Separation method: SFC: column: Daicel Chiralpak AZ-H 5 μm, 250 mm × 20 mm; mobile phase: 60% carbon dioxide / 40% ethanol; temperature: 40 °C; flow rate: 80 mL/min; UV detection: 210 nm.
Analytical method: SFC: column: Daicel Chiralpak AZ-H 5 μm, 250 mm × 4.6 mm; mobile phase: 60% carbon dioxide / 40% ethanol; temperature: 40 °C; flow rate: 3 mL/min; UV detection: 210 nm.

**Synthesis of racemate 30.**[a]

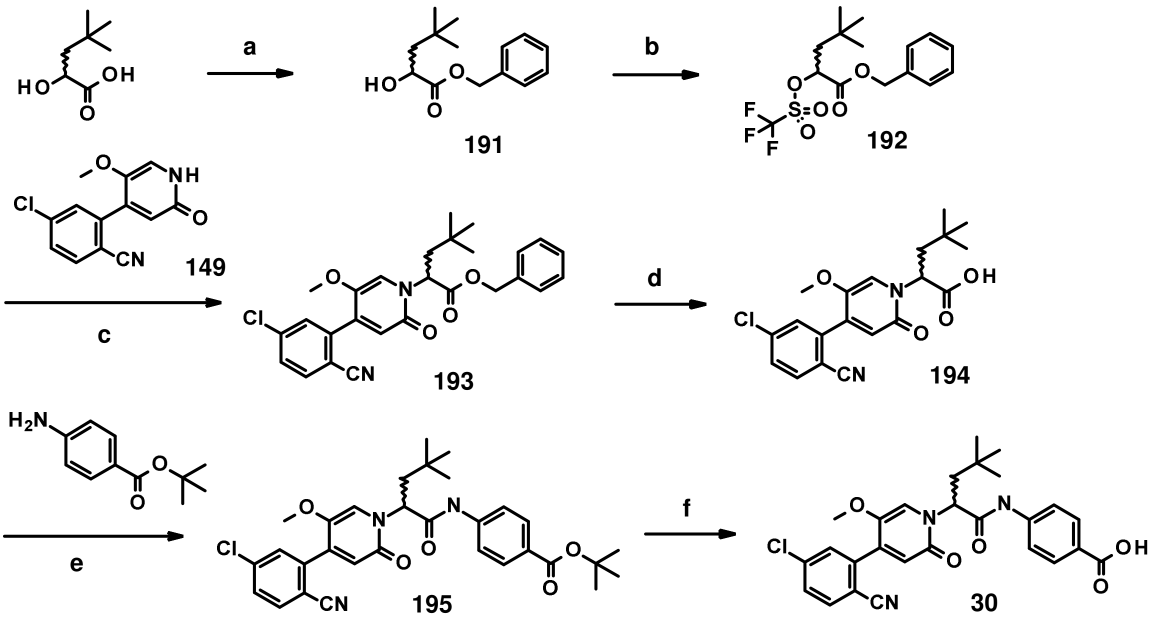

[a]Reagents and conditions: (a) i) $Cs_2CO_3$, MeOH/water, RT, ii) BnBr, DMF, 0 °C → RT, 54%; (b) $Tf_2O$, 2,6-dimethylpyridine, DCM, 0 °C, 99%; (c) NaH, THF, RT, 71%; (d) NaH, THF (not dried), RT, 83%; (e) HATU, DIEA, DMF, RT, 62%; (f) TFA, DCM, RT, 64%.

**Benzyl 2-hydroxy-4,4-dimethylpentanoate (racemate 191).**

Cesium carbonate (743 mg, 2.28 mmol, 0.5 eq.) was added at RT to solution of racemic 2-hydroxy-4,4-dimethylpentanoic acid (667 mg, 4.56 mmol) in a mixture of methanol (8.7 mL) and water (1.7 mL). The reaction mixture was stirred at RT for 1 h and concentrated under reduced pressure. The residue was dried *in vacuo* for 4 h, dissolved in *N,N*-dimethylformamide (10 mL) and cooled to 0 °C, followed by slow addition of benzyl bromide (516 $\mu$L, 4.33 mmol, 0.95 eq.). The reaction mixture was stirred at RT for 12 h and quenched with water. The aqueous phase was extracted with ethyl acetate. The combined organic phases were dried over anhydrous magnesium sulfate, filtered and concentrated under reduced pressure. The residue was purified by flash silica gel chromatography (cyclohexane / ethyl acetate gradient) to give **191**. Yield: 584 mg (54% of theory). $^1$H NMR (400 MHz, $CDCl_3$): δ [ppm] = 7.39-7.33 (m, 5H), 5.23 (d, $J$ = 12.2 Hz, 1H), 5.18 (d, $J$ = 12.2 Hz, 1H), 4.29 (ddd, 1H), 2.62 (d, $J$ = 7.3 Hz, 1H), 1.73 (dd, $J$ = 14.3 Hz, 2.4 Hz, 1H), 1.49 (dd, $J$ = 14.3 Hz, 9.4 Hz, 1H), 0.99 (s, 9H).

**Benzyl 4,4-dimethyl-2-{[(trifluoromethyl)sulfonyl]oxy}pentanoate (racemate 192).**

Trifluoromethanesulfonic acid anhydride (254 $\mu$L, 1.50 mmol) and 2,6-dimethylpyridine (175 $\mu$L, 1.50 mmol) were added under argon atmosphere at 0 °C to a mixture of benzyl 2-hydroxy-4,4-dimethylpentanoate (racemate **191**) (236 mg, 1.00 mmol) in dichloromethane (10 mL). The reaction mixture was stirred at 0 °C for 1 h and diluted with methyl *tert*-butyl ether. The mixture was washed three times with a mixture (3:1, 10 mL) of brine and aqueous hydrochloric acid solution (1 N), dried

over anhydrous magnesium sulfate, filtered and concentrated under reduced pressure to give **192** which was used without further purification. Yield: 365 mg (99% of theory).

**Benzyl 2-[4-(5-chloro-2-cyanophenyl)-5-methoxy-2-oxopyridin-1(2*H*)-yl]-4,4-dimethylpentanoate (racemate 193).**

Sodium hydride (60% in mineral oil, 42 mg, 1.04 mmol, 1.2 eq.) was added in portions under argon atmosphere at RT to a suspension of 4-chloro-2-(5-methoxy-2-oxo-1,2-dihydropyridin-4-yl)benzonitrile (**149**) (261 mg, 87% purity, 0.87 mmol) in tetrahydrofuran (10 mL). The reaction mixture was stirred at RT for 1 h, mixed with a solution of benzyl 4,4-dimethyl-2-{[(trifluoromethyl)sulfonyl]oxy}pentanoate (**192**) (481 mg, 1.31 mmol) in tetrahydrofuran (3 mL), stirred at RT for 1 h and quenched with saturated aqueous ammonium chloride solution. The aqueous phase was extracted with methyl *tert*-butyl ether. The combined organic phases were dried over anhydrous magnesium sulfate, filtered and concentrated under reduced pressure. The residue was purified by flash silica gel chromatography (cyclohexane / ethyl acetate gradient) to give **193**. Yield: 294 mg (71% of theory). LC/MS (method 1): $t_R$ = 1.29 min, MS (ESIpos): *m*/*z* = 479 $[M+H]^+$; $^1H$ NMR (400 MHz, DMSO-$d_6$): δ [ppm] = 7.99 (d, *J* = 8.2 Hz, 1H), 7.74-7.70 (m, 2H), 7.55 (s, 1H), 7.39-7.30 (m, 5H), 6.53 (s, 1H), 5.56-5.50 (m, 1H), 5.18 (s, 2H), 3.63 (s, 3H), 2.19-2.10 (m, 2H), 0.87 (s, 9H).

**2-[4-(5-Chloro-2-cyanophenyl)-5-methoxy-2-oxopyridin-1(2*H*)-yl]-4,4-dimethylpentanoic acid (racemate 194).**

Sodium hydride (60% in mineral oil, 18 mg, 0.44 mmol, 1.5 eq.) was added at RT to a solution of benzyl 2-[4-(5-chloro-2-cyanophenyl)-5-methoxy-2-oxopyridin-1(2*H*)-yl]-4,4-dimethylpentanoate (racemate **193**) (140 mg, 0.29 mmol) in tetrahydrofuran (5 mL, not dried). The reaction mixture was stirred at RT for 15 min and quenched by addition of saturated aqueous ammonium chloride solution (5 mL), dichloromethane (10 mL) and aqueous hydrochloric acid solution (1 N, 0.5 mL). After phase separation, the aqueous phase was extracted with dichloromethane. The combined organic phases were dried over anhydrous sodium sulfate, filtered, concentrated under reduced pressure and dried *in vacuo* to give **194** which was used without further purification. Yield: 110 mg (86% purity, 83% of theory). LC/MS (method 1): $t_R$ = 0.99 min, MS (ESIneg): *m*/*z* = 387 $[M-H]^-$; $^1H$ NMR (400 MHz, DMSO-$d_6$): δ [ppm] = 13.1 (s, 1H), 7.97 (d, *J* = 8.8 Hz, 1H), 7.76-7.69 (m, 2H), 7.52 (s, 1H), 6.48 (s, 1H), 5.50-5.38 (br s, 1H), 3.65 (s, 3H), 2.16-2.10 (m, 2H), 0.86 (s, 9H).

***tert*-Butyl 4-({2-[4-(5-chloro-2-cyanophenyl)-5-methoxy-2-oxopyridin-1(2*H*)-yl]-4,4-dimethylpentanoyl}amino)benzoate (racemate 195).**

A solution of HATU (129 mg, 0.34 mmol, 1.2 eq.) in *N*,*N*-dimethylformamide (2 mL) was added under argon atmosphere at RT to a solution of 2-[4-(5-chloro-2-cyanophenyl)-5-methoxy-2-oxopyridin-1(2*H*)-yl]-4,4-dimethylpentanoic acid (racemate **194**) (110 mg, 0.28 mmol), *tert*-butyl 4-aminobenzoate (66 mg, 0.34 mmol, 1.2 eq.) and *N*,*N*-diisopropylethylamine (148 µL, 0.85 mmol, 3.0 eq.) in *N*,*N*-

dimethylformamide (7 mL). The reaction mixture was stirred at RT overnight and concentrated under reduced pressure. The residue was purified by preparative RP-HPLC (acetonitrile / 0.05% formic acid in water gradient) to give **195**. Yield: 100 mg (62% of theory). LC/MS (method 2): $t_R$ = 2.90 min, MS (ESIpos): *m*/*z* = 564 $[M+H]^+$; $^1$H NMR (400 MHz, DMSO-$d_6$): δ [ppm] = 10.9 (s, 1H), 7.99 (d, *J* = 8.9 Hz, 1H), 7.88 (d, *J* = 8.8 Hz, 2H), 7.77 (d, *J* = 8.9 Hz, 2H), 7.74-7.71 (m, 2H), 7.60 (s, 1H), 6.53 (s, 1H), 5.98 (dd, *J* = 9.2 Hz, 4.9 Hz, 1H), 3.70 (s, 3H), 2.14 (dd, *J* = 14.6 Hz, 9.3 Hz, 1H), 2.02 (dd, *J* = 14.6 Hz, 4.9 Hz, 1H), 1.54 (s, 9H), 0.92 (s, 9H).

**4-({2-[4-(5-Chloro-2-cyanophenyl)-5-methoxy-2-oxopyridin-1(2*H*)-yl]-4,4-dimethylpentanoyl}amino)benzoic acid (racemate 30).**

Trifluoroacetic acid (270 μL, 3.51 mmol, 20 eq.) was added under argon atmosphere at RT to a suspension of *tert*-butyl 4-({2-[4-(5-chloro-2-cyanophenyl)-5-methoxy-2-oxopyridin-1(2*H*)-yl]-4,4-dimethylpentanoyl}amino)benzoate (racemate **195**) (99 mg, 0.18 mmol) in dichloromethane (7 mL). The reaction mixture was stirred at RT for 5 h and concentrated under reduced pressure. The residue was coevaporated several times with dichloromethane, dried *in vacuo* and purified by preparative RP-HPLC (acetonitrile / water gradient) to give **30**. Yield: 57 mg (64% of theory). LC/MS (method 1): $t_R$ = 1.07 min, MS (ESIneg): *m*/*z* = 506 $[M-H]^-$; $^1$H NMR (400 MHz, DMSO-$d_6$): δ [ppm] = 12.8 (s, 1H), 10.9 (s, 1H), 7.99 (d, *J* = 8.9 Hz, 1H), 7.91 (d, *J* = 8.8 Hz, 2H), 7.77 (d, *J* = 8.8 Hz, 2H), 7.74-7.71 (m, 2H), 7.60 (s, 1H), 6.54 (s, 1H), 5.99 (dd, *J* = 8.8 Hz, 5.1 Hz, 1H), 3.70 (s, 3H), 2.12 (dd, *J* = 14.6 Hz, 9.1 Hz, 1H), 2.03 (dd, *J* = 14.6 Hz, 7.1 Hz, 1H), 0.92 (s, 9H).

**Synthesis of eutomer 31.**[a]

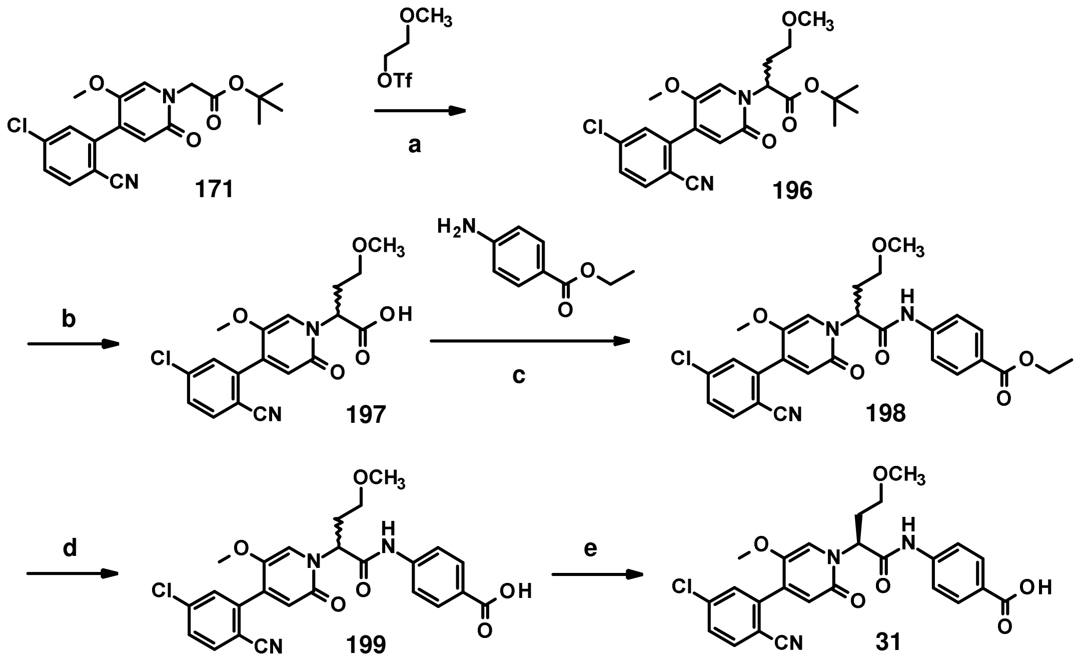

[a]Reagents and conditions: (a) LiHMDS, THF, -70 °C → RT, 84%; (b) HCl/1,4-dioxane, RT, 99%; (c) Oxima®, DIC, DMF, 40 °C, 85%; (d) $Cs_2CO_3$, MeOH/water, 60 °C, 88%; (e) enantiomer separation.

***tert*-Butyl 2-[4-(5-chloro-2-cyanophenyl)-5-methoxy-2-oxopyridin-1(2*H*)-yl]-4-methoxybutanoate (racemate 196).**

Bis-(trimethylsilyl)-lithiumamide solution (1.0 M in tetrahydrofuran, 18.01 mL, 18.01 mmol, 1.35 eq.) was added dropwise under argon atmosphere at -70 °C to a solution of *tert*-butyl [4-(5-chloro-2-cyanophenyl)-5-methoxy-2-oxopyridin-1(2*H*)-yl]acetate (**171**) (5.00 g, 13.34 mmol) in tetrahydrofuran (106 mL). The reaction mixture was stirred at -70 °C for 20 min, mixed with 2-methoxyethyl trifluoromethanesulfonate (3.19 g, 15.34 mmol, 1.15 eq.), stirred at -70 °C for 15 min, allowed to warm to RT and quenched with saturated aqueous ammonium chloride solution. After addition of ethyl acetate and phase separation, the aqueous phase was extracted with ethyl acetate. The combined organic phases were washed with brine, dried over anhydrous sodium sulfate, filtered and concentrated under reduced pressure. The residue was purified by flash silica gel chromatography (cyclohexane / ethyl acetate gradient) to give **196**. Yield: 5.10 g (84% of theory). LC/MS (method 3): $t_R$ = 1.92 min, MS (ESIpos): $m/z$ = 433 $[M+H]^+$; $^1H$ NMR (400 MHz, DMSO-$d_6$): δ [ppm] = 8.02-7.96 (m, 1H), 7.76-7.70 (m, 2H), 7.37 (s, 1H), 6.48 (s, 1H), 5.15-5.07 (m, 1H), 3.64 (s, 3H), 3.43-3.35 (m, 1H), 3.20 (s, 3H), 3.2-3.12 (m, 1H, partially concealed), 2.39-2.29 (m, 2H), 1.40 (s, 9H).

**2-[4-(5-Chloro-2-cyanophenyl)-5-methoxy-2-oxopyridin-1(2*H*)-yl]-4-methoxybutanoic acid (racemate 197).**

A mixture of *tert*-butyl 2-[4-(5-chloro-2-cyanophenyl)-5-methoxy-2-oxopyridin-1(2*H*)-yl]-4-

methoxybutanoate (racemate **196**) (5.10 g, 11.19 mmol) in hydrogen chloride solution (4 M in 1,4-dioxane, 111 mL) was stirred at RT overnight and concentrated under reduced pressure at RT. The residue was coevaporated with tetrahydrofuran and dried *in vacuo* to give **197** which was used without further purification. Yield: 4.40 g (99% of theory). LC/MS (method 3): $t_R$ = 1.37 min, MS (ESIpos): *m*/*z* = 377 $[M+H]^+$; $^1$H NMR (400 MHz, DMSO-$d_6$): δ [ppm] = 13.0 (br s, 1H), 7.99 (d, *J* = 8.2 Hz, 1H), 7.75-7.72 (m, 2H), 7.42 (s, 1H), 6.48 (s, 1H), 5.17-5.09 (m, 1H), 3.63 (s, 3H), 3.41-3.31 (m, 1H), 3.19 (s, 3H), 3.15-3.10 (m, 1H), 2.38-2.33 (m, 2H).

**Ethyl 4-{2-[4-(5-chloro-2-cyanophenyl)-5-methoxy-2-oxopyridin-1(2*H*)-yl]-4-methoxybutan-amido}benzoate (racemate 198).**

Ethyl 4-aminobenzoate (658 mg, 3.98 mmol, 1.0 eq.) and Oxima® (566 mg, 3.98 mmol, 1.0 eq.) were successively added under argon atmosphere at RT to a solution of 2-[4-(5-chloro-2-cyanophenyl)-5-methoxy-2-oxopyridin-1(2*H*)-yl]-4-methoxybutanoic acid (racemate **197**) (1.50 g, 3.98 mmol) in *N*,*N*-dimethylformamide (39 mL), followed by dropwise addition of *N*,*N'*-diisopropylcarbodiimde (620 µL, 3.98 mmol, 1.0 eq.). The reaction mixture was stirred at 40 °C overnight and mixed with water. The forming precipitate was filtered, washed with water and dried *in vacuo* to give **198** which was used without further purification. Yield: 1.87 g (85% of theory). LC/MS (method 1): $t_R$ = 1.10 min, MS (ESIpos): *m*/*z* = 524 $[M+H]^+$; $^1$H NMR (400 MHz, DMSO-$d_6$): δ [ppm] = 10.8 (s, 1H), 8.00 (d, *J* = 9.2 Hz, 1H), 7.94 (d, *J* = 8.7 Hz, 2H), 7.79 (d, *J* = 8.7 Hz, 2H), 7.76-7.70 (m, 2H), 7.51 (s, 1H), 6.53 (s, 1H), 5.80-5.71 (m, 1H), 4.29 (q, *J* = 7.1 Hz, 2H), 3.69 (s, 3H), 3.43-3.25 (m, 2H, partially concealed), 3.21 (s, 3H), 2.45-2.40 (m, 2H), 1.31 (t, *J* = 7.1 Hz, 3H).

**4-{2-[4-(5-Chloro-2-cyanophenyl)-5-methoxy-2-oxopyridin-1(2*H*)-yl]-4-methoxybutanamido}-benzoic acid (racemate 199).**

Cesium carbonate (2.24 g, 6.87 mmol, 2.0 eq.) was added at RT to a mixture of ethyl 4-{2-[4-(5-chloro-2-cyanophenyl)-5-methoxy-2-oxopyridin-1(2*H*)-yl]-4-methoxybutanamido}benzoate (**198**) (1.80 g, 3.44 mmol) in a mixture of methanol (36 mL) and water (9 mL). The reaction mixture was stirred at 60 °C for 6 h, cooled to RT and acidified to pH 3 with aqueous hydrochloric acid solution (1 N). Methanol was removed at 30 °C under reduced pressure. The residual solution was extracted with ethyl acetate. The combined organic phases were dried over anhydrous sodium sulfate, filtered, concentrated under reduced pressure and dried *in vacuo* to give **199**. Yield: 1.66 g (88% of theory). LC/MS (method 1): $t_R$ = 0.89 min, MS (ESIneg): *m*/*z* = 494 $[M-H]^-$.

**4-({(2*S*)-2-[4-(5-Chloro-2-cyanophenyl)-5-methoxy-2-oxopyridin-1(2*H*)-yl]-4-methoxybutanoyl}amino)benzoic acid (eutomer 31).**

Enantiomer separation of 1.66 g of 4-{2-[4-(5-chloro-2-cyanophenyl)-5-methoxy-2-oxopyridin-1(2*H*)-yl]-4-methoxybutanamido}benzoic acid (racemate **199**) gave 707 mg of distomer (chiral HPLC: $t_R$ = 4.59 min) and 631 mg of eutomer **31** (chiral HPLC: $t_R$ = 8.11 min, 98% ee).

LC/MS (method 1): $t_R$ = 0.89 min, MS (ESIneg): $m/z$ = 494 [M-H]$^-$; $^1$H NMR (400 MHz, DMSO-$d_6$): δ [ppm] = 12.8 (s, 1H), 10.76 (s, 1H), 8.00 (d, $J$ = 8.9 Hz, 1H), 7.91 (d, $J$ = 8.9 Hz, 2H), 7.77 (d, $J$ = 8.9 Hz, 2H), 7.76-7.70 (m, 2H), 7.51 (s, 1H), 6.53 (s, 1H), 5.81-5.72 (m, 1H), 3.69 (s, 3H), 3.43-3.25 (m, 2H), 3.20 (s, 3H), 2.44-2.39 (m, 2H); rotation value: $[\alpha]_{589}^{19.9}$ = -95.05° (c 0.33 g/100 cm$^3$, methanol). Separation method: SFC: column: Daicel Chiralpak AZ-H 5 $\mu$m, 250 mm × 20 mm; mobile phase: 70% carbon dioxide / 30% ethanol; temperature: 40 °C; flow rate: 80 mL/min; UV detection: 210 nm. Analytical method: SFC: column: Daicel Chiralpak AZ-H 5 $\mu$m, 250 mm × 4.6 mm; mobile phase: 70% carbon dioxide / 30% ethanol; temperature: 40 °C; flow rate: 3 mL/min; UV detection: 210 nm.

**Synthesis of eutomer 32.**[a]

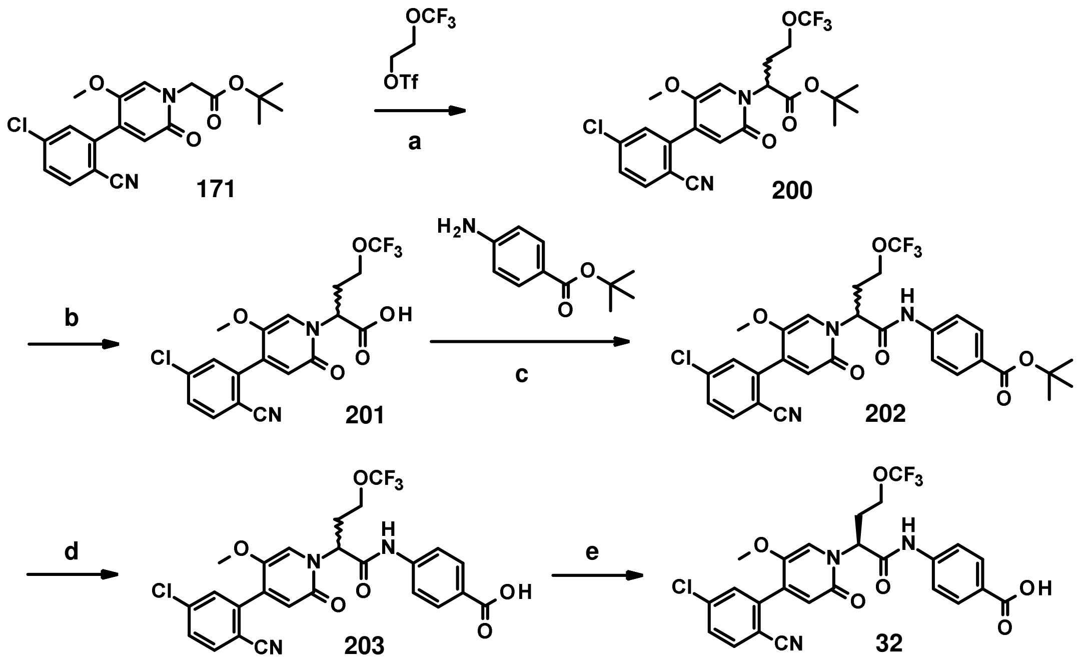


[a]Reagents and conditions: (a) LiHMDS, THF, -78 °C → RT, 62%; (b) TFA, DCM, RT, 96%; (c) Oxima®, DIC, DMF, 40 °C, 64%; (d) TFA, DCM, 0 °C, 47%; (e) enantiomer separation.

***tert*-Butyl 2-[4-(5-chloro-2-cyanophenyl)-5-methoxy-2-oxopyridin-1(2*H*)-yl]-4-(trifluoromethoxy)butanoate (racemate 200).**

Bis-(trimethylsilyl)-lithiumamide solution (1.0 M in tetrahydrofuran, 1.39 mL, 1.39 mmol, 1.1 eq.) was added dropwise under argon atmosphere at -78 °C to a solution of *tert*-butyl [4-(5-chloro-2-cyanophenyl)-5-methoxy-2-oxopyridin-1(2*H*)-yl]acetate (**171**) (500 mg, 1.26 mmol) in tetrahydrofuran (10 mL). The reaction mixture was stirred at -78 °C for 15 min, mixed with 2-(trifluoromethoxy)ethyl trifluoromethanesulfonate (495 mg, 1.89 mmol, 1.5 eq.), stirred at -78 °C for 15 min, at RT for 1 h and quenched with saturated aqueous ammonium chloride solution. After addition of ethyl acetate and phase separation, the aqueous phase was extracted with ethyl acetate. The combined organic phases were dried over anhydrous magnesium sulfate, filtered and concentrated under reduced pressure. The residue was

purified by flash silica gel chromatography (cyclohexane / ethyl acetate gradient) to give **200**. Yield: 386 mg (62% of theory). LC/MS (method 1): $t_R$ = 1.18 min, MS (ESIpos): *m/z* = 487 $[M+H]^+$; $^1H$ NMR (400 MHz, DMSO-$d_6$): δ [ppm] = 7.99 (d, *J* = 8.3 Hz, 1H), 7.75-7.70 (m, 2H), 7.45 (s, 1H), 6.52 (s, 1H), 5.14 (dd, *J* = 8.7 Hz, 5.5 Hz, 1H), 4.22-4.16 (m, 1H), 4.04-3.98 (m, 1H), 3.63 (s, 3H), 2.59-2.51 (m, 2H), 1.40 (s, 9H).

**2-[4-(5-Chloro-2-cyanophenyl)-5-methoxy-2-oxopyridin-1(2*H*)-yl]-4-(trifluoromethoxy)butanoic acid (racemate 201).**

Trifluoroacetic acid (2.28 mL, 29.58 mmol, 37.5 eq.) was added under argon atmosphere at RT to a solution of *tert*-butyl 2-[4-(5-chloro-2-cyanophenyl)-5-methoxy-2-oxopyridin-1(2*H*)-yl]-4-(trifluoromethoxy)butanoate (racemate **200**) (384 mg, 0.79 mmol) in dichloromethane (7.9 mL). The reaction mixture was stirred at RT for 4 h and concentrated under reduced pressure. The residue was coevaporated several times with toluene and dried *in vacuo* to give **201** which was used without further purification. Yield: 330 mg (96% of theory). LC/MS (method 1): $t_R$ = 0.94 min, MS (ESIpos): *m/z* = 431 $[M+H]^+$; $^1H$ NMR (400 MHz, DMSO-$d_6$): δ [ppm] = 13.2 (s, 1H), 7.99 (d, *J* = 8.3 Hz, 1H), 7.74-7.71 (m, 2H), 7.50 (s, 1H), 6.51 (s, 1H), 5.18 (dd, *J* = 8.8 Hz, 5.6 Hz, 1H), 4.22-4.15 (m, 1H), 4.02-3.95 (m, 1H), 3.63 (s, 3H), 2.62-2.51 (m, 2H).

***tert*-Butyl 4-{2-[4-(5-chloro-2-cyanophenyl)-5-methoxy-2-oxopyridin-1(2*H*)-yl]-4-(trifluoromethoxy)butanamido}benzoate (racemate 202).**

*tert*-Butyl 4-aminobenzoate (148 mg, 0.77 mmol, 1.0 eq.) and Oxima® (109 mg, 0.77 mmol, 1.0 eq.) were successively added under argon atmosphere at RT to a solution of 2-[4-(5-chloro-2-cyanophenyl)-5-methoxy-2-oxopyridin-1(2*H*)-yl]-4-(trifluoromethoxy)butanoic acid (racemate **201**) (330 mg, 0.77 mmol) in *N,N*-dimethylformamide (7.7 mL), followed by dropwise addition of *N,N'*-diisopropylcarbodiimde (120 µL, 0.77 mmol, 1.0 eq.). The reaction mixture was stirred at 40 °C overnight and concentrated under reduced pressure. The residue was purified by flash silica gel chromatography (cyclohexane / ethyl acetate gradient) to give **202**. Yield: 329 mg (64% of theory). LC/MS (method 1): $t_R$ = 1.27 min, MS (ESIpos): *m/z* = 606 $[M+H]^+$; $^1H$ NMR (400 MHz, DMSO-$d_6$): δ [ppm] = 10.8 (s, 1H), 8.00 (d, *J* = 8.2 Hz, 1H), 7.88 (d, *J* = 8.7 Hz, 2H), 7.77-7.71 (m, 4H), 7.51 (s, 1H), 6.56 (s, 1H), 5.85-5.77 (m, 1H), 4.20-4.14 (m, 1H), 4.03-3.95 (m, 1H), 3.69 (s, 3H), 2.68-2.60 (m, 2H), 1.54 (s, 9H).

**4-{2-[4-(5-Chloro-2-cyanophenyl)-5-methoxy-2-oxopyridin-1(2*H*)-yl]-4-(trifluoromethoxy)-butanamido}benzoic acid (racemate 203).**

Trifluoroacetic acid (574 µL, 7.45 mmol, 30 eq.) was added under argon atmosphere at 0 °C to a solution of *tert*-butyl 4-{2-[4-(5-chloro-2-cyanophenyl)-5-methoxy-2-oxopyridin-1(2*H*)-yl]-4-(trifluoromethoxy)butanamido}benzoate (racemate **202**) (151 mg, 0.25 mmol) in dichloromethane (3.4 mL). The reaction mixture was stirred at 0 °C for 5 h and concentrated under reduced pressure. The

residue was purified by preparative RP-HPLC (acetonitrile / water gradient) to give **203**. Yield: 65 mg (47% of theory). LC/MS (method 1): $t_R$ = 1.02 min, MS (ESIneg): *m/z* = 548 $[M-H]^-$; $^1H$ NMR (400 MHz, DMSO-$d_6$): δ [ppm] = 12.8 (s, 1H), 10.8 (s, 1H), 8.00 (d, *J* = 8.3 Hz, 1H), 7.91 (d, *J* = 8.8 Hz, 2H), 7.76-7.70 (m, 4H), 7.51 (s, 1H), 6.56 (s, 1H), 5.85-5.78 (m, 1H), 4.20-4.15 (m, 1H), 4.03-3.99 (m, 1H), 3.69 (s, 3H), 2.66-2.60 (m, 2H).

**4-{[(2*S*)-2-[4-(5-Chloro-2-cyanophenyl)-5-methoxy-2-oxopyridin-1(2*H*)-yl]-4-(trifluoromethoxy)butanoyl]amino}benzoic acid (eutomer 32).**

Enantiomer separation of 165 mg of 4-{2-[4-(5-chloro-2-cyanophenyl)-5-methoxy-2-oxopyridin-1(2*H*)-yl]-4-(trifluoromethoxy)butanamido}benzoic acid (racemate **203**) gave 65 mg of distomer (chiral HPLC: $t_R$ = 1.00 min) and 69 mg of eutomer **32** (chiral HPLC: $t_R$ = 2.01 min, 94% ee).

LC/MS (method 1): $t_R$ = 1.02 min, MS (ESIneg): *m/z* = 548 $[M-H]^-$; $^1H$ NMR (400 MHz, DMSO-$d_6$): δ [ppm] = 12.8 (s, 1H), 10.77 (s, 1H), 8.00 (d, *J* = 8.3 Hz, 1H), 7.92 (d, *J* = 8.8 Hz, 2H), 7.79-7.69 (m, 4H), 7.51 (s, 1H), 6.56 (s, 1H), 5.85-5.78 (m, 1H), 4.21-4.14 (m, 1H), 4.04-3.96 (m, 1H), 3.69 (s, 3H), 2.66-2.59 (m, 2H).

Separation method: SFC: column: Daicel Chiralpak AZ-H 5 μm, 250 mm × 30 mm; mobile phase: 70% carbon dioxide / 30% ethanol; temperature: 40 °C; flow rate: 100 mL/min; UV detection: 210 nm. Analytical method: SFC: column: Daicel Chiralpak AZ-H 5 μm, 250 mm × 4.6 mm; mobile phase: 85% carbon dioxide / 15% ethanol; temperature: 30 °C; flow rate: 3 mL/min; UV detection: 220 nm.

**Synthesis of eutomer 33.**[a]

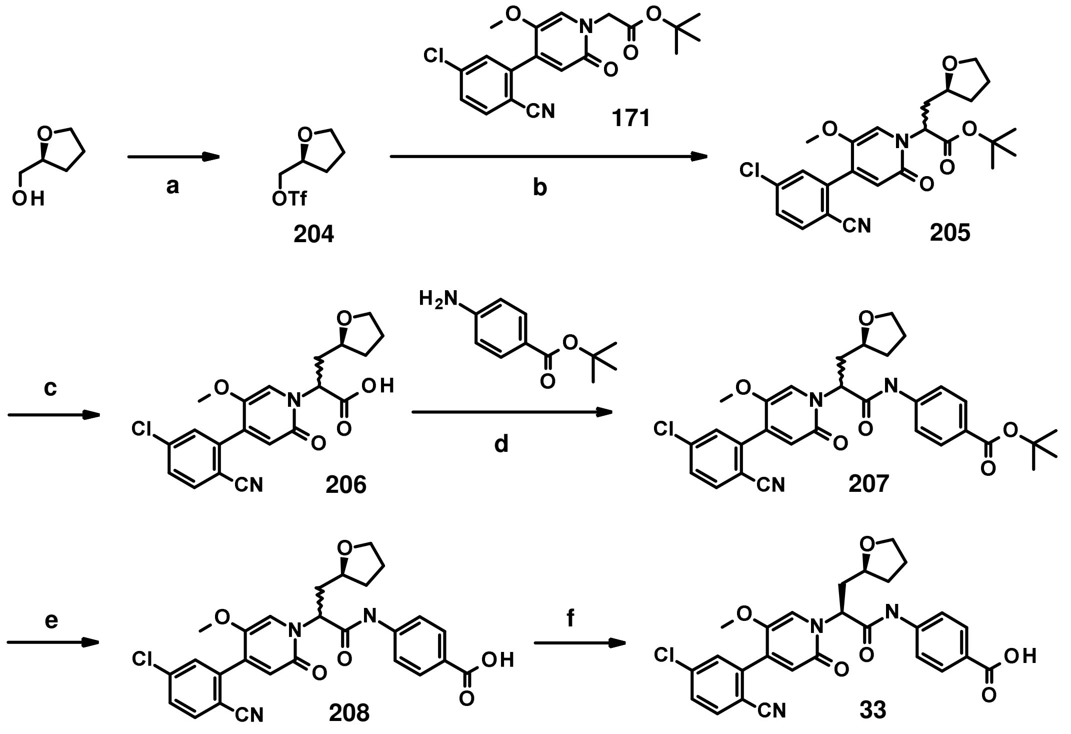

[a]Reagents and conditions: (a) $Tf_2O$, 2,6-dimethylpyridine, DCM, 0 °C; (b) LiHMDS, THF, -78 °C → RT, 49%; (c) TFA, DCM, 0 °C → RT, 96%; (d) HATU, DIEA, DMF, RT, 79%; (e) TFA, DCM, 0 °C → RT, 79%; (f) diastereomer separation.

**(2*S*)-Tetrahydrofuran-2-ylmethyl trifluoromethanesulfonate (204).**

2,6-Dimethylpyridine (2.20 mL, 18.90 mmol, 1.1 eq.) was added under argon atmosphere at 0 °C to a mixture of (2*S*)-tetrahydrofuran-2-ylmethanol (2.19 g, 80% purity, 17.19 mmol) in dichloromethane (35 mL), followed by dropwise addition of trifluoromethanesulfonic acid anhydride (3.05 mL, 18.04 mmol, 1.05 eq.) at 0 °C. The reaction mixture was stirred at 0 °C for 30 min and diluted with methyl *tert*-butyl ether. The mixture was washed three times with a mixture (3:1) of brine and aqueous hydrochloric acid solution (1 N), dried over anhydrous sodium sulfate, filtered and concentrated under reduced pressure (at RT and 95 mbar) to give **204** which was immediately used without further purification. Yield: 3.89 g. $^1$H NMR (400 MHz, DMSO-$d_6$): δ [ppm] = 4.35 (dd, *J* = 11.7 Hz, 2.8 Hz, 1H), 4.17 (dd, *J* = 11.7 Hz, 7.6 Hz, 1H), 4.09 (dq, 1H), 3.86-3.70 (m, 2H), 2.00-1.79 (m, 3H), 1.60-1.47 (m, 1H).

***tert*-Butyl 2-[4-(5-chloro-2-cyanophenyl)-5-methoxy-2-oxopyridin-1(2*H*)-yl]-3-[(2*S*)-tetrahydrofuran-2-yl]propanoate (mixture of two enantiopure diastereomers 205).**

Bis-(trimethylsilyl)-lithiumamide solution (1.0 M in tetrahydrofuran, 8.93 mL, 8.93 mmol, 1.2 eq.) was added under argon atmosphere at -78 °C to a solution of *tert*-butyl [4-(5-chloro-2-cyanophenyl)-5-methoxy-2-oxopyridin-1(2*H*)-yl]acetate (**171**) (3.00 g, 93% purity, 7.44 mmol) in tetrahydrofuran

(80 mL). The reaction mixture was stirred at -78 °C for 15 min and mixed with (2*S*)-tetrahydrofuran-2-ylmethyl trifluoromethanesulfonate (**204**) (3.49 g, 80% purity, 11.91 mmol, 1.6 eq.). The mixture was stirred at -78 °C for 15 min, allowed to warm to RT, quenched with water and extracted with ethyl acetate. The combined organic phases were washed with brine, dried over anhydrous sodium sulfate, filtered, concentrated under reduced pressure and dried *in vacuo*. The residue was purified by flash silica gel chromatography (cyclohexane / ethyl acetate gradient) to give **205**. Yield: 1.71 g (49% of theory). LC/MS (method 5): $t_R$ = 1.39 min, MS (ESIpos): *m*/*z* = 403 [M-*t*Bu+H]$^+$.

**2-[4-(5-Chloro-2-cyanophenyl)-5-methoxy-2-oxopyridin-1(2*H*)-yl]-3-[(2*S*)-tetrahydrofuran-2-yl]propanoic acid (mixture of two enantiopure diastereomers 206).**

Trifluoroacetic acid (5.18 mL, 67.18 mmol, 20 eq.) was added under argon atmosphere at 0 °C to a mixture of *tert*-butyl 2-[4-(5-chloro-2-cyanophenyl)-5-methoxy-2-oxopyridin-1(2*H*)-yl]-3-[(2*S*)-tetrahydrofuran-2-yl]propanoate (mixture of two enantiopure diastereomers **205**) (1.57 g, 3.36 mmol) in dichloromethane (30 mL). The reaction mixture was stirred at 0 °C for 10 min, allowed to warm to RT, stirred at RT for 5 h and concentrated under reduced pressure. The residue was coevaporated with dichloromethane and toluene and dried *in vacuo* to give **206** which was used without further purification. Yield: 1.41 g (92% purity, 96% of theory). LC/MS (method 5): diastereomer 1: $t_R$ = 1.09 min, MS (ESIpos): *m*/*z* = 403 [M+H]$^+$; diastereomer 2: $t_R$ = 1.11 min, MS (ESIpos): *m*/*z* = 403 [M+H]$^+$.

***tert*-Butyl 4-{2-[4-(5-chloro-2-cyanophenyl)-5-methoxy-2-oxopyridin-1(2*H*)-yl]-3-[(2*S*)-tetrahydrofuran-2-yl]propanamido}benzoate (mixture of two enantiopure diastereomers 207).**

A solution of HATU (1.60 g, 4.22 mmol, 1.2 eq.) in *N,N*-dimethylformamide (5 mL) was added under argon atmosphere at RT to a solution of 2-[4-(5-chloro-2-cyanophenyl)-5-methoxy-2-oxopyridin-1(2*H*)-yl]-3-[(2*S*)-tetrahydrofuran-2-yl]propanoic acid (mixture of two enantiopure diastereomers **206**) (1.54 g, 92% purity, 3.52 mmol), *tert*-butyl 4-aminobenzoate (747 mg, 3.87 mmol, 1.1 eq.) and *N,N*-diisopropylethylamine (1.35 mL, 7.73 mmol, 2.2 eq.) in *N,N*-dimethylformamide (15 mL). The reaction mixture was stirred at RT for 3 h and concentrated under reduced pressure. The residue was dissolved in ethyl acetate and extracted with water. The combined organic phases were washed with brine, dried over anhydrous sodium sulfate, filtered and concentrated under reduced pressure. The residue was purified by flash silica gel chromatography (cyclohexane / ethyl acetate gradient) to give **207**. Yield: 1.61 g (79% of theory). LC/MS (method 1): $t_R$ = 1.23 min, MS (ESIpos): *m*/*z* = 578 [M+H]$^+$.

**4-{2-[4-(5-Chloro-2-cyanophenyl)-5-methoxy-2-oxopyridin-1(2*H*)-yl]-3-[(2*S*)-tetrahydrofuran-2-yl]propanamido}benzoic acid (mixture of two enantiopure diastereomers 208).**

Trifluoroacetic acid (4.30 mL, 55.77 mmol, 20 eq.) was added under argon atmosphere at 0 °C to a mixture of *tert*-butyl 4-{2-[4-(5-chloro-2-cyanophenyl)-5-methoxy-2-oxopyridin-1(2*H*)-yl]-3-[(2*S*)-tetrahydrofuran-2-yl]propanamido}benzoate (mixture of two enantiopure diastereomers **207**) (1.61 g, 2.79 mmol) in dichloromethane (30 mL). The reaction mixture was allowed to warm to RT, stirred at

RT for 4 h and concentrated under reduced pressure. The residue was coevaporated several times with toluene and dichloromethane and dried *in vacuo*. The residue was stirred in diethyl ether, and the solid was filtered and dried *in vacuo* to give **208**. Yield: 1.27 g (90% purity, 79% of theory). LC/MS (method 5): $t_R$ = 1.19 min, MS (ESIpos): *m/z* = 522 $[M+H]^+$.

**4-({(2*S*)-2-[4-(5-Chloro-2-cyanophenyl)-5-methoxy-2-oxopyridin-1(2*H*)-yl]-3-[(2*S*)-tetrahydrofuran-2-yl]propanoyl}amino)benzoic acid (eutomer 33).**

Diastereomer separation of 1.27 g of 4-{2-[4-(5-chloro-2-cyanophenyl)-5-methoxy-2-oxopyridin-1(2*H*)-yl]-3-[(2*S*)-tetrahydrofuran-2-yl]propanamido}benzoic acid (mixture of two enantiopure diastereomers **208**) gave 350 mg of eutomer **33** (chiral HPLC: $t_R$ = 4.31 min, >99% de) and 452 mg of distomer (chiral HPLC: $t_R$ = 6.69 min).

LC/MS (method 1): $t_R$ = 0.96 min, MS (ESIpos): *m/z* = 522 $[M+H]^+$; $^1$H NMR (400 MHz, DMSO-$d_6$): δ [ppm] = 12.75 (br s, 1H), 10.77 (s, 1H), 7.99 (d, *J* = 8.8 Hz, 1H), 7.91 (d, *J* = 8.5 Hz, 2H), 7.77 (d, *J* = 8.8 Hz, 2H), 7.75-7.70 (m, 2H), 7.55 (s, 1H), 6.52 (s, 1H), 5.86-5.79 (m, 1H), 3.80-3.70 (m, 2H), 3.68 (s, 3H), 3.63-3.55 (m, 1H), 2.36-2.24 (m, 2H), 2.01-1.72 (3x m, 3H), 1.69-1.57 (m, 1H).

Separation method: SFC: column: Daicel Chiralpak IC 5 μm, 250 mm × 20 mm; mobile phase: 75% carbon dioxide / 25% ethanol; temperature: 40 °C; flow rate: 100 mL/min; UV detection: 210 nm.

Analytical method: SFC: column: Daicel Chiralpak IC 5 μm, 250 mm × 4.6 mm; mobile phase: 70% carbon dioxide / 30% ethanol; flow rate: 3 mL/min; UV detection: 210 nm.

**Synthesis of (2*S*)-tetrahydro-2*H*-pyran-2-ylmethyl trifluoromethanesulfonate 214.**[a]

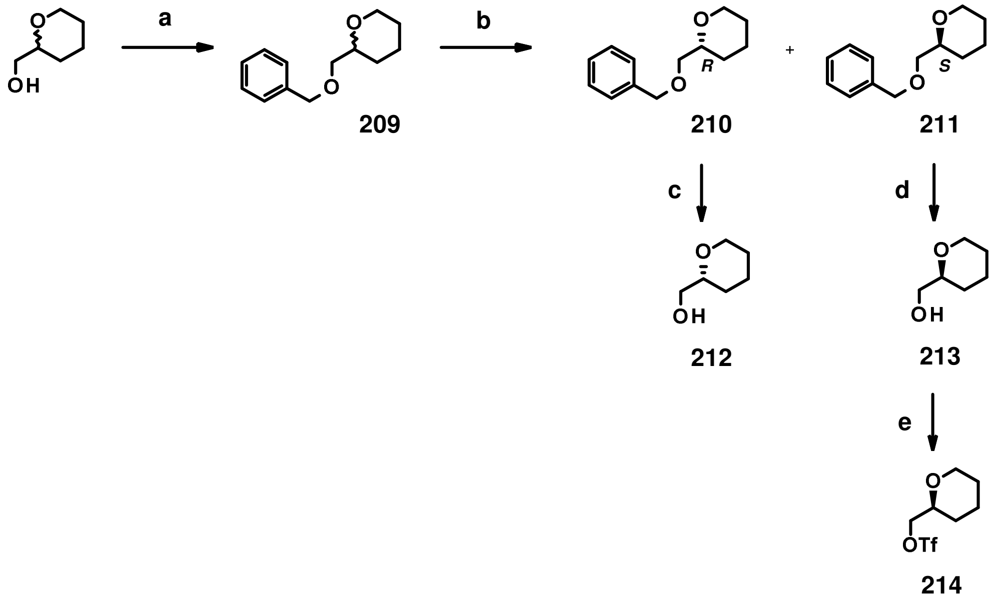


[a]Reagents and conditions: (a) BnBr, NaH, THF, 0 °C → RT, 94%; (b) enantiomer separation; (c) $H_2$, Pd/C, EtOH, RT, 63%; (d) $H_2$, Pd/C, EtOH, RT, 86%; (e) $Tf_2O$, $NEt_3$, DCM, -78 °C → RT, 87%.

**2-[(Benzyloxy)methyl]tetrahydro-2*H*-pyran (racemate 209).**

A solution of racemic tetrahydro-2*H*-pyran-2-ylmethanol (25.00 g, 215.22 mmol) in tetrahydrofuran (500 mL) was added under argon atmosphere at 0 °C to a mixture of sodium hydride (60% in mineral oil, 9.47 g, 236.74 mmol, 1.1 eq.) in tetrahydrofuran (500 mL). The reaction mixture was stirred at 0 °C for 30 min, mixed dropwise with benzyl bromide (25.74 mL, 215.22 mmol, 1.0 eq.), stirred at 0 °C for 30 min and at RT for 1 h and quenched with saturated aqueous ammonium chloride solution. After addition of methyl *tert*-butyl ether and phase separation, the aqueous phase was extracted with *tert*-butyl methyl ether. The combined organic phases were dried over anhydrous magnesium sulfate, filtered and concentrated under reduced pressure. The residue was purified by flash silica gel chromatography (cyclohexane / ethyl acetate gradient) to give **209**. Yield: 41.90 g (94% of theory). LC/MS (method 2): $t_R$ = 2.18 min, MS (ESIpos): *m/z* = 207 $[M+H]^+$; $^1$H NMR (400 MHz, DMSO-$d_6$): δ [ppm] = 7.37-7.25 (m, 5H), 4.47 (s, 2H), 3.87-3.81 (m, 1H), 3.47-3.28 (m, 4H), 1.80-1.72 (m, 1H), 1.58-1.37 (m, 4H), 1.25-1.13 (m, 1H).

**(2*R*)-2-[(Benzyloxy)methyl]tetrahydro-2*H*-pyran (210) and (2*S*)-2-[(benzyloxy)methyl]-tetrahydro-2*H*-pyran (211).**

Enantiomer separation of 41.90 g of 2-[(benzyloxy)methyl]tetrahydro-2*H*-pyran (racemate **209**) gave 16.68 g of (2*R*)-2-[(benzyloxy)methyl]tetrahydro-2*H*-pyran (**210**) (chiral HPLC: $t_R$ = 5.28 min, 99% ee, 93% purity; $[\alpha]_{589}^{20.0}$ = +14.9° (c 0.43 g/100 cm$^3$, $CHCl_3$)) and 17.00 g of (2*S*)-2-[(benzyloxy)methyl]tetrahydro-2*H*-pyran (**211**) (chiral HPLC: $t_R$ = 7.36 min, 96% ee, 96% purity; $[\alpha]_{589}^{20.0}$ = -13.9° (c 0.61 g/100 cm$^3$, $CHCl_3$)).
Separation method: column: Daicel Chiralpak OD-H 5 $\mu$m, 250 mm × 20 mm; mobile phase: 95% *iso*-hexane / 5% 2-propanol; temperature: 25 °C; flow rate: 25 mL/min; UV detection: 210 nm.
Analytical method: column: Daicel Chiralpak OD-H 5 $\mu$m, 250 mm × 4.6 mm; mobile phase: 95% *iso*-hexane / 5% 2-propanol; flow rate: 1 mL/min; UV detection: 220 nm.

**(2*R*)-Tetrahydro-2*H*-pyran-2-ylmethanol (212).**

Palladium (10% on charcoal, 2.00 g) was added under argon atmosphere at RT to a solution of (2*R*)-2-[(benzyloxy)methyl]tetrahydro-2*H*-pyran (**210**) (16.70 g, 93% purity, 75.29 mmol) in ethanol (150 mL). The reaction mixture was stirred under hydrogen atmosphere (1 bar) at RT overnight, filtered through Celite®, mixed under argon atmosphere at RT with additional palladium (10% on charcoal, 1.50 g) and stirred under hydrogen atmosphere (1 bar) at RT for another 72 h. The mixture was filtered through Celite®, and the filter residue was washed with ethanol. The combined filtrates were concentrated under reduced pressure. The residue was purified by flash silica gel chromatography (cyclohexane / ethyl acetate gradient) to give **212**. Yield: 5.47 g (63% of theory). GC/MS (method 9): $t_R$ = 2.16 min, MS (EI): *m/z* = 116 $[M]^+$; $^1$H NMR (400 MHz, DMSO-$d_6$): δ [ppm] = 4.51 (t, *J* = 11.4 Hz, 1H), 3.87-3.81 (m, 1H), 3.37-3.18 (m, 4H), 1.80-1.71 (m, 1H), 1.59-1.50 (m, 1H), 1.49-1.36 (m, 3H), 1.19-1.05 (m,

1H); optical rotation: $[\alpha]_{589}^{20.0}$ = -9.4° (c 0.4 g/100 $cm^3$, $CHCl_3$).

**(2*S*)-Tetrahydro-2*H*-pyran-2-ylmethanol (213).**

Palladium (10% on charcoal, 3.51 g) was added under argon atmosphere at RT to a solution of (2*S*)-2-[(benzyloxy)methyl]tetrahydro-2*H*-pyran (**211**) (17.00 g, 82.41 mmol) in ethanol (120 mL). The reaction mixture was stirred under hydrogen atmosphere (1 bar) at RT overnight, mixed under argon atmosphere at RT with additional palladium (10% on charcoal, 1.75 g) and stirred under hydrogen atmosphere (1 bar) at RT for another 72 h. The mixture was filtered through Celite®, and the filter residue was washed with ethanol. The combined filtrates were concentrated under reduced pressure. The residue was purified by flash silica gel chromatography (dichloromethane / methanol gradient) to give **213**. Yield: 8.23 g (86% of theory). GC/MS (method 10): $t_R$ = 1.82 min, MS (EI): *m*/*z* = 116 $[M]^+$; $^1$H NMR (400 MHz, DMSO-$d_6$): δ [ppm] = 4.51 (t, *J* = 11.4 Hz, 1H), 3.87-3.81 (m, 1H), 3.37-3.18 (m, 4H), 1.80-1.71 (m, 1H), 1.59-1.50 (m, 1H), 1.49-1.36 (m, 3H), 1.19-1.05 (m, 1H); optical rotation: $[\alpha]_{589}^{20.0}$ = +9.1° (c 0.36 g/100 $cm^3$, $CHCl_3$), compare with A. Aponick, B. Biannic *Org. Lett.* **2011**, *13*, 1330-1333.

**(2*S*)-Tetrahydro-2*H*-pyran-2-ylmethyl trifluoromethanesulfonate (214).**

A solution of (2*S*)-tetrahydro-2*H*-pyran-2-ylmethanol (**213**) (330 mg, 2.84 mmol) and triethylamine (0.48 mL, 3.41 mmol, 1.2 eq.) in dichloromethane (5 mL) was added dropwise under argon atmosphere at -78 °C to a solution of trifluoromethanesulfonic anhydride (0.57 mL, 3.41 mmol, 1.2 eq.) in dichloromethane (10 mL). The reaction mixture was stirred at -78 °C for 30 min, allowed to come to RT and diluted with *tert*-butyl methyl ether. The mixture was washed three times with a mixture (3:1) of brine and aqueous hydrochloric acid solution (1 N). The organic phase was dried over anhydrous sodium sulfate, filtered and concentrated under reduced pressure (at <25 °C and >150 mbar) to give **214** which was used without further purification. Yield: 617 mg (87% of theory). $^1$H NMR (400 MHz, DMSO-$d_6$): δ [ppm] = 4.32 (dd, *J* = 12.1 Hz, 2.4 Hz, 1H), 4.18 (dd, *J* = 12.1 Hz, 7.0 Hz, 1H), 4.00-3.92 (m, 1H), 3.60-3.52 (m, 1H), 3.48-3.39 (m, 1H), 1.85-1.74 (m, 1H), 1.56-1.41 (m, 4H), 1.28-1.14 (m, 1H).

**Synthesis of eutomer 34.**[a]

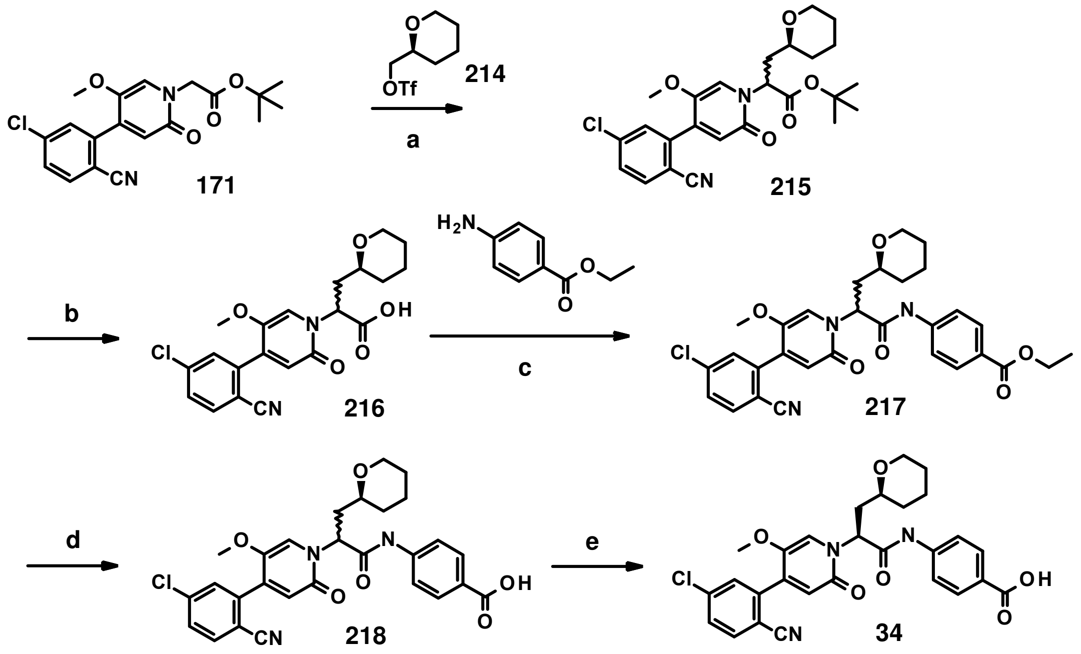

[a]Reagents and conditions: (a) LiHMDS, THF, -78 °C → RT, 81%; (b) TFA, DCM, RT, 100%; (c) Oxima®, DIC, DMF, 40 °C, 85%; (d) $Cs_2CO_3$, MeOH, 60 °C, 74%; (e) diastereomer separation.

***tert*-Butyl 2-[4-(5-chloro-2-cyanophenyl)-5-methoxy-2-oxopyridin-1(2*H*)-yl]-3-[(2*S*)-tetrahydro-2*H*-pyran-2-yl]propanoate (mixture of two enantiopure diastereomers 215).**

Bis-(trimethylsilyl)-lithiumamide solution (1.0 M in tetrahydrofuran, 12.03 mL, 12.03 mmol, 1.1 eq.) was added under argon atmosphere at -78 °C to a solution of *tert*-butyl [4-(5-chloro-2-cyanophenyl)-5-methoxy-2-oxopyridin-1(2*H*)-yl]acetate (**171**) (4.10 g, 10.94 mmol) in tetrahydrofuran (90 mL). The reaction mixture was stirred at -78 °C for 15 min and mixed with (2*S*)-tetrahydro-2*H*-pyran-2-ylmethyl trifluoromethanesulfonate (**214**) (4.08 g, 16.41 mmol, 1.5 eq.). The mixture was stirred at -78 °C for 15 min, allowed to warm to RT, stirred at RT for 2 h, quenched with aqueous ammonium chloride solution and extracted with methyl *tert*-butyl ether. The combined organic phases were dried over anhydrous sodium sulfate, filtered and concentrated under reduced pressure. The residue was purified by flash silica gel chromatography (cyclohexane / ethyl acetate gradient) to give **215**. Yield: 4.20 g (81% of theory). LC/MS (method 1): $t_R$ = 1.15 min, MS (ESIpos): $m/z$ = 473 $[M+H]^+$.

**2-[4-(5-Chloro-2-cyanophenyl)-5-methoxy-2-oxopyridin-1(2*H*)-yl]-3-[(2*S*)-tetrahydro-2*H*-pyran-2-yl]propanoic acid (mixture of two enantiopure diastereomers 216).**

Trifluoroacetic acid (59.68 mL, 777.00 mmol, 37.5 eq.) was added under argon atmosphere at RT to a mixture of *tert*-butyl 2-[4-(5-chloro-2-cyanophenyl)-5-methoxy-2-oxopyridin-1(2*H*)-yl]-3-[(2*S*)-tetrahydro-2*H*-pyran-2-yl]propanoate (mixture of two enantiopure diastereomers **215**) (9.80 g, 20.72 mmol) in dichloromethane (245 mL). The reaction mixture was stirred at RT for 2.5 h and concentrated under reduced pressure. The residue was coevaporated with toluene and dried *in vacuo* to

give **216** which was used without further purification. Yield: 8.70 g (100% of theory). LC/MS (method 1): $t_R$ = 0.96 min, MS (ESIpos): $m/z$ = 417 $[M+H]^+$.

**Ethyl 4-{2-[4-(5-chloro-2-cyanophenyl)-5-methoxy-2-oxopyridin-1(2*H*)-yl]-3-[(2*S*)-tetrahydro-2*H*-pyran-2-yl]propanamido}benzoate (mixture of two enantiopure diastereomers 217).**

Ethyl 4-aminobenzoate (3.88 g, 23.51 mmol, 1.0 eq.) and Oxima® (3.34 g, 23.51 mmol, 1.0 eq.) were successively added under argon atmosphere at RT to a solution of 2-[4-(5-chloro-2-cyanophenyl)-5-methoxy-2-oxopyridin-1(2*H*)-yl]-3-[(2*S*)-tetrahydro-2*H*-pyran-2-yl]propanoic acid (mixture of two enantiopure diastereomers **216**) (9.80 g, 23.51 mmol) in *N,N*-dimethylformamide (234 mL), followed by dropwise addition of *N,N'*-diisopropylcarbodiimde (3.66 mL, 23.51 mmol, 1.0 eq.). The reaction mixture was stirred at 40 °C for 16 h and concentrated under reduced pressure. The residue was purified by flash silica gel chromatography (cyclohexane / ethyl acetate gradient) to give **217**. Yield: 11.30 g (85% of theory). LC/MS (method 1): diastereomer 1: $t_R$ = 1.19 min, MS (ESIpos): $m/z$ = 564 $[M+H]^+$; diastereomer 2: $t_R$ = 1.21 min, MS (ESIpos): $m/z$ = 564 $[M+H]^+$.

**4-{2-[4-(5-Chloro-2-cyanophenyl)-5-methoxy-2-oxopyridin-1(2*H*)-yl]-3-[(2*S*)-tetrahydro-2*H*-pyran-2-yl]propanamido}benzoic acid (mixture of two enantiopure diastereomers 218).**

Cesium carbonate (11.06 g, 40.07 mmol, 2.0 eq.) and water (52 mL) were added at RT to a mixture of ethyl 4-{2-[4-(5-chloro-2-cyanophenyl)-5-methoxy-2-oxopyridin-1(2*H*)-yl]-3-[(2*S*)-tetrahydro-2*H*-pyran-2-yl]propanamido}benzoate (mixture of two enantiopure diastereomers **217**) (11.30 g, 20.03 mmol) in methanol (210 mL). The reaction mixture was stirred at 60 °C for 28 h and cooled to RT. Methanol was removed under reduced pressure. The residual suspension was diluted with water (91 mL), acidified with aqueous hydrochloric acid solution (1 N) to pH 3 and extracted several times with ethyl acetate. The combined organic phases were dried over anhydrous sodium sulfate, filtered, concentrated under reduced pressure and dried *in vacuo* to give **218** which was submitted for diastereomer separation without further purification. Yield: 10.70 g (75% purity, 74% of theory). LC/MS (method 1): $t_R$ = 1.00 min, MS (ESIpos): $m/z$ = 536 $[M+H]^+$.

**4-({(2*S*)-2-[4-(5-Chloro-2-cyanophenyl)-5-methoxy-2-oxopyridin-1(2*H*)-yl]-3-[(2*S*)-tetrahydro-2*H*-pyran-2-yl]propanoyl}amino)benzoic acid (eutomer 34).**

Diastereomer separation of 10.70 g (75% purity) of 4-{2-[4-(5-chloro-2-cyanophenyl)-5-methoxy-2-oxopyridin-1(2*H*)-yl]-3-[(2*S*)-tetrahydro-2*H*-pyran-2-yl]propanamido}benzoic acid (mixture of two enantiopure diastereomers **218**) gave 3.07 g of distomer (chiral HPLC: $t_R$ = 3.53 min) and 1.99 g of eutomer **34** (chiral HPLC: $t_R$ = 6.20 min, >99% de).

LC/MS (method 1): $t_R$ = 1.00 min, MS (ESIpos): $m/z$ = 536 $[M+H]^+$; $^1H$ NMR (400 MHz, DMSO-$d_6$): δ [ppm] = 12.76 (s, 1H), 10.75 (s, 1H), 7.99 (d, *J* = 8.8 Hz, 1H), 7.91 (d, *J* = 8.8 Hz, 2H), 7.79 (d, *J* = 8.8 Hz, 2H), 7.74-7.71 (m, 2H), 7.53 (s, 1H), 6.53 (s, 1H), 5.83 (t, *J* = 7.7 Hz, 1H), 3.91-3.83 (m, 1H), 3.68 (s, 3H), 3.28-3.22 (m, 2H), 2.35-2.28 (m, 1H), 2.19-2.12 (m, 1H), 1.79-1.72 (m, 1H), 1.67-1.60 (m,

1H), 1.47-1.36 (m, 3H), 1.33-1.23 (m, 1H).

Separation method: SFC: column: Daicel Chiralpak AD-H 20 $\mu$m, 360 mm × 50 mm; mobile phase: 75% carbon dioxide / 25% ethanol; temperature: 20 °C; flow rate: 400 mL/min; UV detection: 220 nm.

Analytical method: SFC: column: Daicel Chiralpak AD-H 5 $\mu$m, 250 mm × 4.6 mm; mobile phase: 70% carbon dioxide / 30% ethanol; flow rate: 3 mL/min; UV detection: 210 nm.

**Synthesis of 35.**[a]

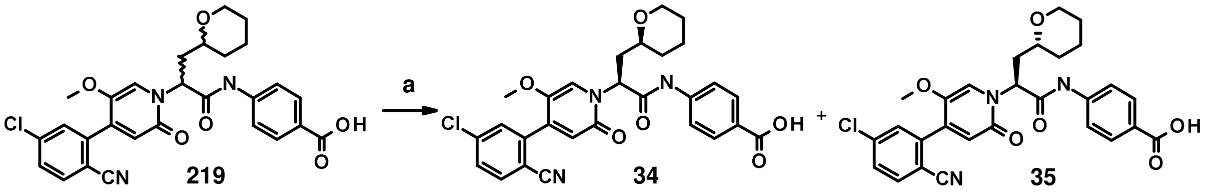

[a]Reagents and conditions: (a) diastereomer and enantiomer separation.

**4-{2-[4-(5-Chloro-2-cyanophenyl)-5-methoxy-2-oxopyridin-1(2*H*)-yl]-3-(tetrahydro-2*H*-pyran-2-yl)propanamido}benzoic acid (mixture of racemic diastereomers 219).**

4-{2-[4-(5-Chloro-2-cyanophenyl)-5-methoxy-2-oxopyridin-1(2*H*)-yl]-3-(tetrahydro-2*H*-pyran-2-yl)propanamido}benzoic acid (mixture of racemic diastereomers **219**) was synthesized analogous to the synthesis of **218** starting from racemic tetrahydro-2*H*-pyran-2-ylmethyl trifluoromethanesulfonate. LC/MS (method 1): $t_R$ = 1.00 min, MS (ESIneg): *m*/*z* = 534 $[M-H]^-$; $^1$H NMR (400 MHz, DMSO-$d_6$): δ [ppm] = 12.8 (s, 1H), 10.8 / 10.7 (2x s, 1H), 8.02-7.98 (m, 1H), 7.93-7.87 (m, 2H), 7.80-7.71 (m, 4H), 7.53 / 7.49 (2x s, 1H), 6.52 / 6.51 (2x s, 1H), 5.85-5.71 (m, 1H), 3.90-3.78 (m, 1H), 3.69 / 3.68 (2x s, 3H), 3.29-3.15 (m, 1H), 3.13-3.05 (m, 1H), 2.42-2.11 (m, 2H), 1.78-1.70 (m, 1H), 1.67-1.56 (m, 1H), 1.47-1.35 (m, 3H), 1.30-1.19 (m, 1H).

**4-({(2*S*)-2-[4-(5-Chloro-2-cyanophenyl)-5-methoxy-2-oxopyridin-1(2*H*)-yl]-3-[(2*R*)-tetrahydro-2*H*-pyran-2-yl]propanoyl}amino)benzoic acid (35).**

Diastereomer and enantiomer separation of 1.17 g of 4-{2-[4-(5-chloro-2-cyanophenyl)-5-methoxy-2-oxopyridin-1(2*H*)-yl]-3-(tetrahydro-2*H*-pyran-2-yl)propanamido}benzoic acid (mixture of racemic diastereomers **219**) gave 231 mg of distomer (enantiomer 1 of diastereomer 1, chiral HPLC: $t_R$ = 9.96 min), 54 mg of **34** (enantiomer 1 of diastereomer 2, chiral HPLC: $t_R$ = 15.34 min, 99% ee), 91 mg of distomer (enantiomer 2 of diastereomer 2, chiral HPLC: $t_R$ = 20.83min) and 183 mg of **35** (enantiomer 2 of diastereomer 1, chiral HPLC: $t_R$ = 27.14 min, 99% ee).

**34:** LC/MS (method 1): $t_R$ = 1.00 min, MS (ESIpos): *m*/*z* = 536 $[M+H]^+$; $^1$H NMR (400 MHz, DMSO-$d_6$): δ [ppm] = 12.8 (s, 1H), 10.7 (s, 1H), 7.99 (d, 1H), 7.90 (d, 2H), 7.77 (d, 2H), 7.74-7.71 (m, 2H), 7.52 (s, 1H), 6.52 (s, 1H), 5.78-5.71 (m, 1H), 3.90-3.84 (m, 1H), 3.68 (s, 3H), 3.28-3.22 (m, 2H), 2.34-2.27 (m, 1H), 2.19-2.10 (m, 1H), 1.78-1.73 (m, 1H), 1.67-1.60 (m, 1H), 1.47-1.36 (m, 3H), 1.33-1.23

(m, 1H).

**35:** LC/MS (method 1): $t_R$ = 1.00 min, MS (ESIpos): *m/z* = 536 [M+H]$^+$; $^1$H NMR (400 MHz, DMSO-$d_6$): δ [ppm] = 12.7 (s, 1H), 10.7 (s, 1H), 8.00 (d, 1H), 7.90 (d, 2H), 7.77-7.72 (m, 4H), 7.49 (s, 1H), 6.51 (s, 1H), 5.78-5.71 (m, 1H), 3.84-3.79 (m, 1H), 3.69 (s, 3H), 3.23-3.15 (m, 1H), 3.13-3.05 (m, 1H), 2.42-2.32 (m, 1H), 2.26-2.18 (m, 1H), 1.78-1.71 (m, 1H), 1.62-1.56 (m, 1H), 1.46-1.37 (m, 3H), 1.30-1.20 (m, 1H).

Separation method: SFC: column: Daicel Chiralpak AD-H 5 μm, 250 mm × 20 mm; mobile phase: 80% carbon dioxide / 20% 2-propanol; temperature: 40 °C; flow rate: 80 mL/min; UV detection: 210 nm.

Analytical method: column: Daicel Chiralpak AZ-H 5 μm, 250 mm × 4.6 mm; mobile phase: 50% *iso*-hexane / 50% 2-propanol plus 0.2% trifluoroacetic acid in 1% water; flow rate: 3 mL/min; UV detection: 210 nm.

**Synthesis of eutomer 36.**[a]

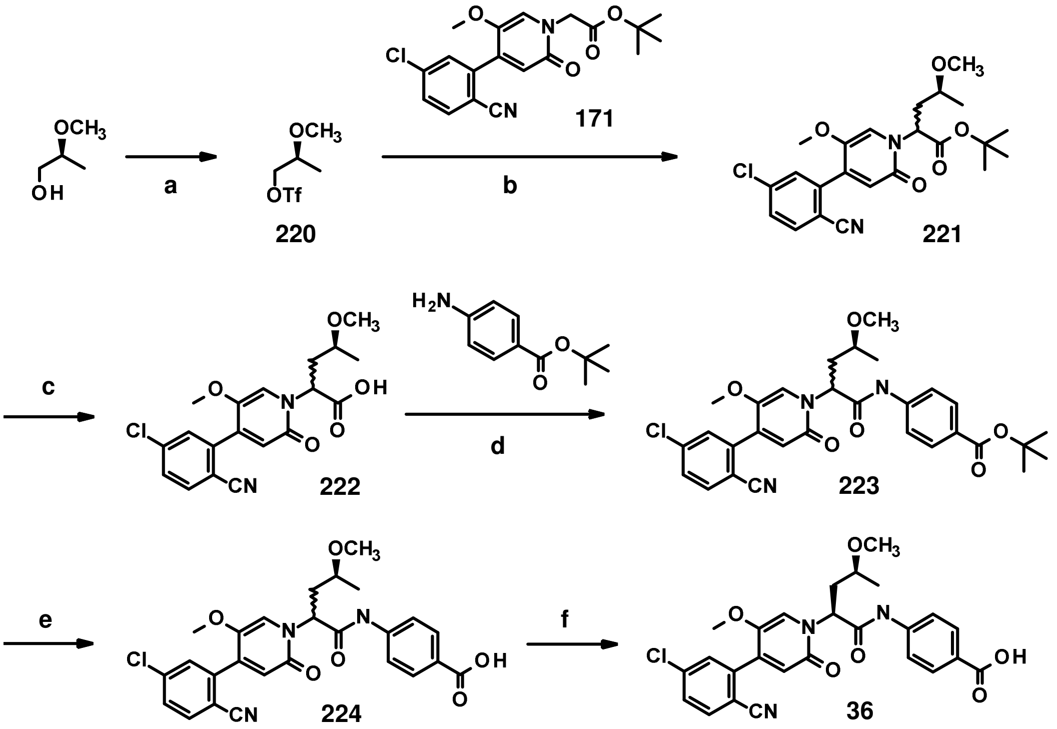


[a]Reagents and conditions: (a) $Tf_2O$, 2,6-dimethylpyridine, DCM, 0 °C; (b) LiHMDS, THF, -78 °C → RT, 73%; (c) TFA, DCM, RT, 100%; (d) HATU, DIEA, DMF, 71%; (e) TFA, DCM, RT, 70%; (f) diastereomer separation.

**(2*S*)-2-Methoxypropyl trifluoromethanesulfonate (220).**

2,6-Dimethylpyridine (917 μL, 7.87 mmol, 1.1 eq.) was added under argon atmosphere at 0 °C to a mixture of (2*S*)-(+)-2-methoxypropan-1-ol (645 mg, 7.16 mmol) in dichloromethane (12 mL), followed by dropwise addition of trifluoromethanesulfonic acid anhydride (1.27 mL, 7.52 mmol, 1.05 eq.) at

0 °C. The reaction mixture was stirred at 0 °C for 30 min and diluted with methyl *tert*-butyl ether. The mixture was washed three times with a mixture (3:1, 3 mL) of brine and aqueous hydrochloric acid solution (1 N), dried over anhydrous sodium sulfate, filtered and concentrated under reduced pressure (at RT and 90 mbar) to give **220** which was immediately used without further purification. Yield: 1.51 g.

***tert*-Butyl 2-[4-(5-chloro-2-cyanophenyl)-5-methoxy-2-oxopyridin-1(2*H*)-yl]-2,3,5-trideoxy-4-*O*-methyl-L-glycero-pentonate (mixture of two enantiopure diastereomers 221).**

Bis-(trimethylsilyl)-lithiumamide solution (1.0 M in tetrahydrofuran, 1.27 mL, 1.27 mmol, 1.1 eq.) was added under argon atmosphere at -78 °C to a solution of *tert*-butyl [4-(5-chloro-2-cyanophenyl)-5-methoxy-2-oxopyridin-1(2*H*)-yl]acetate (**171**) (450 mg, 1.15 mmol) in tetrahydrofuran (15 mL). The reaction mixture was stirred at -78 °C for 15 min and mixed with (2*S*)-2-methoxypropyl trifluoromethanesulfonate (**220**) (384 mg, 1.73 mmol, 1.5 eq.). The mixture was stirred at -78 °C for 15 min, allowed to warm to RT, quenched with water and extracted with ethyl acetate. The combined organic phases were washed with brine, dried over anhydrous sodium sulfate, filtered, concentrated under reduced pressure and dried *in vacuo*. The residue was purified by flash silica gel chromatography (cyclohexane / ethyl acetate gradient) to give **221**. Yield: 375 mg (73% of theory). LC/MS (method 1): $t_R$ = 1.09 min, MS (ESIpos): $m/z$ = 447 $[M+H]^+$.

**2-[4-(5-Chloro-2-cyanophenyl)-5-methoxy-2-oxopyridin-1(2*H*)-yl]-2,3,5-trideoxy-4-*O*-methyl-L-glycero-pentonic acid (mixture of two enantiopure diastereomers 222).**

Trifluoroacetic acid (1.29 mL, 16.78 mmol, 20 eq.) was added under argon atmosphere at 0 °C to a mixture of *tert*-butyl 2-[4-(5-chloro-2-cyanophenyl)-5-methoxy-2-oxopyridin-1(2*H*)-yl]-2,3,5-trideoxy-4-*O*-methyl-L-glycero-pentonate (mixture of two enantiopure diastereomers **221**) (375 mg, 0.84 mmol) in dichloromethane (10 mL). The reaction mixture was stirred at RT for 5 h and concentrated under reduced pressure. The residue was coevaporated with dichloromethane and toluene and dried *in vacuo* to give **222** which was used without further purification. Yield: 391 mg (92% purity, 100% of theory). LC/MS (method 22): diastereomer 1: $t_R$ = 2.28 min, MS (ESIpos): $m/z$ = 391 $[M+H]^+$; diastereomer 2: $t_R$ = 2.36 min, MS (ESIpos): $m/z$ = 391 $[M+H]^+$.

***tert*-Butyl 4-({(4*S*)-2-[4-(5-chloro-2-cyanophenyl)-5-methoxy-2-oxopyridin-1(2*H*)-yl]-4-methoxypentanoyl}amino)benzoate (mixture of two enantiopure diastereomers 223).**

A solution of HATU (420 mg, 1.10 mmol, 1.2 eq.) in *N,N*-dimethylformamide (4 mL) was added under argon atmosphere at RT to a solution of 2-[4-(5-chloro-2-cyanophenyl)-5-methoxy-2-oxopyridin-1(2*H*)-yl]-2,3,5-trideoxy-4-*O*-methyl-L-glycero-pentonic acid (mixture of two enantiopure diastereomers **222**) (391 mg, 92% purity, 0.92 mmol), *tert*-butyl 4-aminobenzoate (196 mg, 1.01 mmol, 1.1 eq.) and *N,N*-diisopropylethylamine (353 µL, 2.03 mmol, 2.2 eq.) in *N,N*-dimethylformamide (8 mL). The reaction mixture was stirred at RT for 30 min and concentrated under reduced pressure. The residue was crystallized from water, and the solid was filtered and dried *in vacuo*. The residue was

purified by flash silica gel chromatography (cyclohexane / ethyl acetate gradient) to give **223**. Yield: 387 mg (71% of theory). LC/MS (method 1): diastereomer 1: $t_R$ = 1.23 min, MS (ESIpos): *m/z* = 566 $[M+H]^+$; diastereomer 2: $t_R$ = 1.24 min, MS (ESIpos): *m/z* = 566 $[M+H]^+$.

**4-({(4*S*)-2-[4-(5-Chloro-2-cyanophenyl)-5-methoxy-2-oxopyridin-1(2*H*)-yl]-4-methoxypentanoyl}amino)benzoic acid (mixture of two enantiopure diastereomers 224).**

Trifluoroacetic acid (1.01 mL, 13.13 mmol, 20 eq.) was added under argon atmosphere at 0 °C to a mixture of *tert*-butyl 4-({(4*S*)-2-[4-(5-chloro-2-cyanophenyl)-5-methoxy-2-oxopyridin-1(2*H*)-yl]-4-methoxypentanoyl}amino)benzoate (mixture of two enantiopure diastereomers **223**) (387 mg, 0.66 mmol) in dichloromethane (8 mL). The reaction mixture was allowed to warm to RT, stirred at RT for 3 h and concentrated under reduced pressure. The residue was coevaporated several times with dichloromethane, dried *in vacuo* and purified by preparative RP-HPLC (acetonitrile / water gradient) to give **224**. Yield: 245 mg (70% of theory). LC/MS (method 1): $t_R$ = 0.96 min, MS (ESIpos): *m/z* = 510 $[M+H]^+$; $^1H$ NMR (400 MHz, DMSO-$d_6$): δ [ppm] = 12.77 (br s, 1H), 10.80 / 10.75 (2x s, 1H), 8.00 (d, *J* = 8.1 Hz, 1H), 7.94-7.87 (m, 2H), 7.81-7.71 (m, 4H), 7.57 / 7.51 (2x s, 1H), 6.53 (2x s, 1H), 5.89-5.80 (m, 1H), 3.69 (s, 3H), 3.25-3.19 / 3.17-3.09 (2x m, 1H), 3.19 / 3.12 (2x s, 3H), 2.43-2.28 (m, 1H), 2.28-2.17 (m, 1H), 1.16 / 1.14 (2x d, *J* = 6.2 Hz, 3H).

**4-({(2*S*,4*S*)-2-[4-(5-Chloro-2-cyanophenyl)-5-methoxy-2-oxopyridin-1(2*H*)-yl]-4-methoxypentanoyl}amino)benzoic acid (eutomer 36).**

Diastereomer separation of 240 mg of 4-({(4*S*)-2-[4-(5-chloro-2-cyanophenyl)-5-methoxy-2-oxopyridin-1(2*H*)-yl]-4-methoxypentanoyl}amino)benzoic acid (mixture of two enantiopure diastereomers **224**) gave 57 mg of distomer (chiral HPLC: $t_R$ = 8.1 min) and 10 mg of eutomer **36** (chiral HPLC: $t_R$ = 10.9 min, 98% de).

LC/MS (method 1): $t_R$ = 0.96 min, MS (ESIpos): *m/z* = 510 $[M+H]^+$; $^1H$ NMR (400 MHz, DMSO-$d_6$): δ [ppm] = 12.75 (br s, 1H), 10.77 (s, 1H), 7.99 (d, *J* = 8.1 Hz, 1H), 7.91 (d, *J* = 8.7 Hz, 2H), 7.77 (d, *J* = 8.7 Hz, 2H), 7.75-7.70 (m, 2H), 7.57 (s, 1H), 6.53 (s, 1H), 5.85 (dd, *J* = 8.5 Hz, 6.7 Hz, 1H), 3.69 (s, 3H), 3.26-3.20 (m, 1H), 3.19 (s, 3H), 2.36-2.27 (m, 1H), 2.26-2.18 (m, 1H), 1.16 (d, *J* = 6.1 Hz, 3H).

Separation method: column: Daicel Chiralpak IF 5 µm, 250 mm × 20 mm; mobile phase: 50% *iso*-hexane / 50% ethanol plus 0.2% trifluoroacetic acid in 1% water; temperature: 23 °C; flow rate: 20 mL/min; UV detection: 220 nm.

Analytical method: column: Daicel Chiralpak AZ-H 5 µm, 250 mm × 4.6 mm; mobile phase: 50% *iso*-hexane / 50% ethanol plus 0.2% trifluoroacetic acid in 1% water; temperature: 40 °C; flow rate: 1 mL/min; UV detection: 220 nm.

**Synthesis of eutomer 37.**[a]

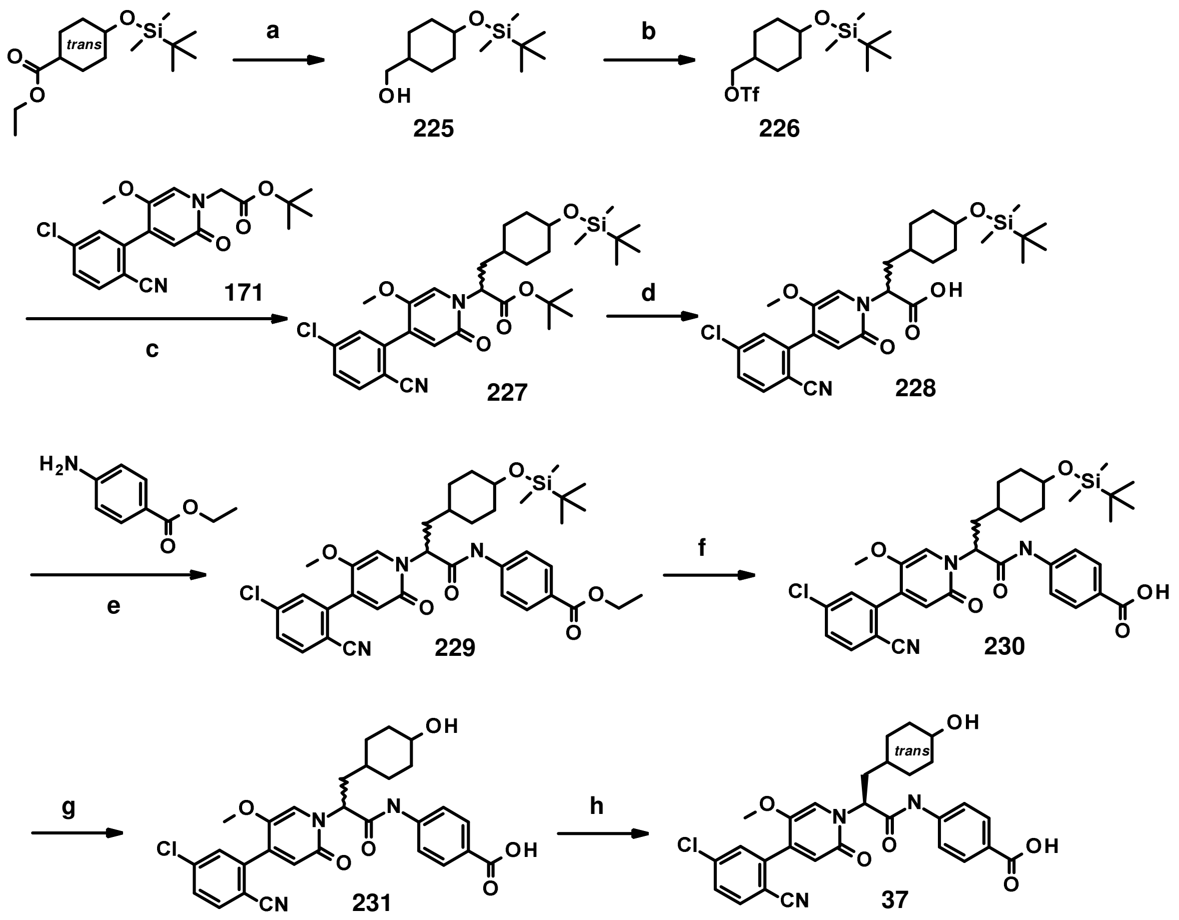

[a]Reagents and conditions: (a) LiAlH4, MTBE, 40 °C, 95%; (b) $Tf_2O$, 2,6-dimethylpyridine, DCM, 0 °C; (c) LiHMDS, THF, -78 °C → RT, 42%; (d) LiOH × $H_2O$, THF/EtOH/water, RT, 100%; (e) Oxima®, DIC, DMF, 40 °C, 31%; (f) $Cs_2CO_3$, MeOH/water, 60 °C; (g) HF-pyridine complex, aq. HCl, THF, RT, 81%; (h) enantiomer separation.

**(4-{[*tert*-Butyl(dimethyl)silyl]oxy}cyclohexyl)methanol (*trans*-isomer 225).**

A solution of ethyl 4-{[*tert*-butyl(dimethyl)silyl]oxy}cyclohexanecarboxylate (*trans*-isomer) (7.80 g, 27.23 mmol) in methyl *tert*-butyl ether (90 mL) was added dropwise under argon atmosphere at RT to lithium aluminum hydride solution (2.4 M in tetrahydrofuran, 12.48 mL, 29.95 mmol, 1.1 eq.). The reaction mixture was stirred at 40 °C for 6 h, cooled to RT and mixed with water (7 mL) and aqueous potassium hydroxide solution (15%, 7 mL). The precipitate (salts) was filtered. The organic phase was dried over anhydrous sodium sulfate, filtered and concentrated under reduced pressure to give **225** which was used without further purification. Yield: 6.30 g (95% of theory). GC/MS (method 9): $t_R$ = 4.74 min, MS (EI): $m/z$ = 244 $[M]^+$.

**(4-{[*tert*-Butyl(dimethyl)silyl]oxy}cyclohexyl)methyl trifluoromethanesulfonate (*trans*-isomer 226).**

2,6-Dimethylpyridine (4.50 mL, 38.66 mmol, 1.5 eq.) and trifluoromethanesulfonic anhydride (6.54 mL, 38.66 mmol, 1.5 eq.) were added under argon atmosphere at 0 °C to a solution of (4-{[*tert*-butyl(dimethyl)silyl]oxy}cyclohexyl)methanol (*trans*-isomer **225**) (6.30 g, 25.77 mmol) in dichloromethane (90 mL). The reaction mixture was stirred at 0 °C for 1 h and diluted with *tert*-butyl methyl ether. The mixture was washed three times with a mixture (3:1) of brine and aqueous hydrogen chloride solution (1 N) and once with saturated aqueous sodium hydrogen carbonate solution. The organic phase was dried over anhydrous sodium sulfate, filtered and concentrated under reduced pressure to give **226** which was used without further purification. Yield: 9.70 g (100% of theory).

***tert*-Butyl 3-(4-{[*tert*-butyl(dimethyl)silyl]oxy}cyclohexyl)-2-[4-(5-chloro-2-cyanophenyl)-5-methoxy-2-oxopyridin-1(2*H*)-yl]propanoate (racemic *trans*-isomer 227).**

Bis-(trimethylsilyl)-lithiumamide solution (1.0 M in tetrahydrofuran, 13.58 mL, 13.58 mmol, 1.1 eq.) was added under argon atmosphere at -78 °C to a solution of *tert*-butyl [4-(5-chloro-2-cyanophenyl)-5-methoxy-2-oxopyridin-1(2*H*)-yl]acetate (**171**) (4.90 g, 94% purity, 12.35 mmol) in tetrahydrofuran (98 mL). The reaction mixture was stirred at -78 °C for 15 min and mixed with (4-{[*tert*-butyl(dimethyl)silyl]oxy}cyclohexyl)methyl trifluoromethanesulfonate (*trans*-isomer **226**) (6.97 g, 18.52 mmol, 1.5 eq.). The mixture was stirred at -78 °C for 15 min, allowed to warm to RT, stirred at RT for 2 h, quenched with aqueous ammonium chloride solution and extracted with *tert*-butyl methyl ether. The combined organic phases were dried over anhydrous sodium sulfate, filtered and concentrated under reduced pressure. The residue was purified by flash silica gel chromatography (cyclohexane / ethyl acetate gradient) to give **227**. Yield: 3.10 g (42% of theory). LC/MS (method 1): $t_R$ = 1.59 min, MS (ESIpos): $m/z$ = 601 $[M+H]^+$; $^1H$ NMR (400 MHz, DMSO-$d_6$): δ [ppm] = 7.97 (d, $J$ = 8.2 Hz, 1H), 7.75-7.68 (m, 2H), 7.36 (s, 1H), 6.48 (s, 1H), 5.25-5.19 (m, 1H), 3.62 (s, 3H), 3.56-3.45 (m, 1H), 2.14-1.54 (m, 6H), 1.39 (s, 9H), 1.23-0.88 (m, 5H), 0.82 (s, 9H), 0.00 (s, 6H).

**3-(4-{[*tert*-Butyl(dimethyl)silyl]oxy}cyclohexyl)-2-[4-(5-chloro-2-cyanophenyl)-5-methoxy-2-oxopyridin-1(2*H*)-yl]propanoic acid (racemic *trans*-isomer 228).**

Lithium hydroxide monohydrate (1.08 g, 25.78 mmol, 5.0 eq.) was added at RT to a solution of *tert*-butyl 3-(4-{[*tert*-butyl(dimethyl)silyl]oxy}cyclohexyl)-2-[4-(5-chloro-2-cyanophenyl)-5-methoxy-2-oxopyridin-1(2*H*)-yl]propanoate (racemic *trans*-isomer **227**) (3.10 g, 5.16 mmol) in a mixture of tetrahydrofuran (32 mL), ethanol (16 mL) and water (16 mL).The reaction mixture was stirred at RT for 6 h, acidified to pH 4-5 with aqueous hydrochloric acid solution(1 N) and extracted with ethyl acetate. The combined organic phases were dried over anhydrous sodium sulfate, filtered and concentrated under reduced pressure to give **228** which was used without further purification. Yield: 2.80 g (100% of theory). LC/MS (method 1): $t_R$ = 1.37 min, MS (ESIpos): $m/z$ = 545 $[M+H]^+$.

**Ethyl 4-{3-(4-{[*tert*-butyl(dimethyl)silyl]oxy}cyclohexyl)-2-[4-(5-chloro-2-cyanophenyl)-5-methoxy-2-oxopyridin-1(2*H*)-yl]propanamido}benzoate (racemic *trans*-isomer 229).**

A mixture of 3-(4-{[*tert*-butyl(dimethyl)silyl]oxy}cyclohexyl)-2-[4-(5-chloro-2-cyanophenyl)-5-methoxy-2-oxopyridin-1(2*H*)-yl]propanoic acid (racemic *trans*-isomer **228**) (2.80 g, 5.14 mmol), ethyl 4-aminobenzoate (848 mg, 5.14 mmol, 1.0 eq.) and Oxima® (730 mg, 5.14 mmol, 1.0 eq.) in *N,N*-dimethylformamide (51 mL) was degassed and flushed with argon at RT for 10 min, followed by addition of *N,N'*-diisopropylcarbodiimde (800 $\mu$L, 5.14 mmol, 1.0 eq.). The reaction mixture was stirred at 40 °C overnight, diluted with aqueous lithium chloride solution (10%, 350 mL) and ethyl acetate (280 mL). After phase separation, the aqueous phase was extracted with ethyl acetate. The combined organic phases were dried over anhydrous sodium sulfate, filtered and concentrated under reduced pressure. The residue was purified by flash silica gel chromatography (cyclohexane / ethyl acetate gradient) to give **229**. Yield: 1.10 g (31% of theory). LC/MS (method 1): $t_R$ = 1.56 min, MS (ESIpos): *m/z* = 692 $[M+H]^+$.

**4-{3-(4-{[*tert*-Butyl(dimethyl)silyl]oxy}cyclohexyl)-2-[4-(5-chloro-2-cyanophenyl)-5-methoxy-2-oxopyridin-1(2*H*)-yl]propanamido}benzoic acid (racemic *trans*-isomer 230).**

Cesium carbonate (1.04 g, 3.18 mmol, 2.0 eq.) was added at RT to a mixture of ethyl 4-{3-(4-{[*tert*-butyl(dimethyl)silyl]oxy}cyclohexyl)-2-[4-(5-chloro-2-cyanophenyl)-5-methoxy-2-oxopyridin-1(2*H*)-yl]propanamido}benzoate (racemic *trans*-isomer **229**) (1.10 g, 1.59 mmol) in methanol (22 mL) and water (5.5 mL). The reaction mixture was stirred at 60 °C for 6 h, cooled to RT, mixed with additional cesium carbonate (0.52 g, 1.59 mmol, 1.0 eq.) and methanol (11 mL), stirred at 60 °C for 3 h, cooled to RT and acidified to pH 3 with aqueous hydrochloric acid solution (1 N). Methanol was removed under reduced pressure. The residual mixture was extracted with ethyl acetate. The combined organic phases were dried over anhydrous sodium sulfate, filtered and concentrated under reduced pressure to give **230** in a mixture with fully deprotected **231**. Yield: 900 mg. LC/MS (method 1): **230** / **231**: $t_R$ = 0.88 min / 1.39 min, MS (ESIpos): *m/z* = 550 / 664 $[M+H]^+$.

**4-{2-[4-(5-Chloro-2-cyanophenyl)-5-methoxy-2-oxopyridin-1(2*H*)-yl]-3-(4-hydroxycyclohexyl)propanamido}benzoic acid (racemic *trans*-isomer 231).**

Hydrogen fluoride pyridine complex (282 $\mu$L) was added at RT to a solution of the mixture of **230** and **231** (racemic *trans*-isomers) (900 mg) in tetrahydrofuran (25 mL). The reaction mixture was stirred at RT for 1.5 h (incomplete conversion), mixed with aqueous hydrochloric acid solution (1 N, 9 mL), stirred at RT for 2 h and concentrated under reduced pressure. The residue was mixed with water and extracted with ethyl acetate. The combined organic phases were dried over anhydrous sodium sulfate, filtered and concentrated under reduced pressure to give **231**. Yield: 600 mg (81% of theory). LC/MS (method 1): $t_R$ = 0.88 min, MS (ESIpos): *m/z* = 550 $[M+H]^+$.

**4-{[(2*S*)-2-[4-(5-Chloro-2-cyanophenyl)-5-methoxy-2-oxopyridin-1(2*H*)-yl]-3-(*trans*-4-hydroxycyclohexyl)propanoyl]amino}benzoic acid (*trans*-eutomer 37).**

Enantiomer separation of 600 mg of 4-{2-[4-(5-chloro-2-cyanophenyl)-5-methoxy-2-oxopyridin-1(2*H*)-yl]-3-(4-hydroxycyclohexyl)propanamido}benzoic acid (racemic *trans*-isomer **231**) gave 166 mg of distomer (chiral HPLC: $t_R$ = 5.2 min) and 157 mg of eutomer **37** (chiral HPLC: $t_R$ = 7.3 min, >99% ee). LC/MS (method 1): $t_R$ = 0.89 min, MS (ESIpos): *m*/*z* = 500 $[M+H]^+$; $^1$H NMR (400 MHz, DMSO-$d_6$): δ [ppm] = 12.75 (s, 1H), 10.79 (s, 1H), 8.00 (d, *J* = 8.3 Hz, 1H), 7.91 (d, *J* = 8.8 Hz, 2H), 7.79-7.70 (m, 4H), 7.49 (s, 1H), 6.54 (s, 1H), 5.87-5.80 (m, 1H), 4.44 (d, *J* = 4.4 Hz, 1H), 3.69 (s, 3H), 2.23-2.12 (m, 1H), 1.95-1.85 (m, 1H), 1.84-1.68 (m, 4H), 1.10-0.93 (m, 5H).

Separation method: column: Daicel Chiralpak AZ-H 5 µm, 250 mm × 20 mm; mobile phase: 50% *iso*-hexane / 50% ethanol with 0.2% glacial acetic acid; temperature: 40 °C; flow rate: 20 mL/min; UV detection: 220 nm.

Analytical method: column: Daicel Chiralpak AZ-H 5 µm, 250 mm × 4.6 mm; mobile phase: 50% *iso*-hexane / 50% ethanol with 0.2% glacial acetic acid; flow rate: 1 mL/min; UV detection: 220 nm.

**Compounds of Table 4.**

**Synthesis of mixture of two enantiopure diastereomers 38.**[a]

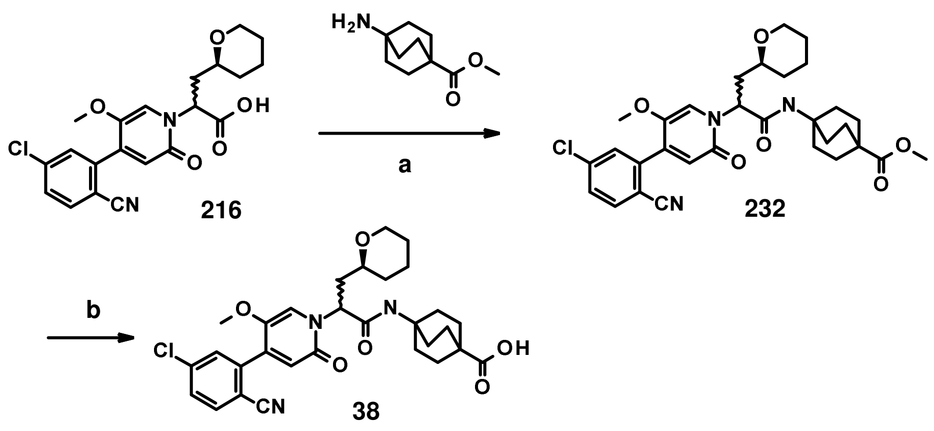


[a]Reagents and conditions: (a) T3P, pyridine, 80 °C, 75%; (b) $Cs_2CO_3$, MeOH/water, 60 °C, 71%.

**Methyl 4-{2-[4-(5-chloro-2-cyanophenyl)-5-methoxy-2-oxopyridin-1(2*H*)-yl]-3-[(2*S*)-tetrahydro-2*H*-pyran-2-yl]propanamido}bicyclo[2.2.2]octane-1-carboxylate (mixture of two enantiopure diastereomers 232).**

Propylphosphonic anhydride (T3P, 50% solution in ethyl acetate, 707 µL, 1.21 mmol, 4.0 eq.) was added under argon atmosphere at RT to a solution of 2-[4-(5-chloro-2-cyanophenyl)-5-methoxy-2-oxopyridin-1(2*H*)-yl]-3-[(2*S*)-tetrahydro-2*H*-pyran-2-yl]propanoic acid (mixture of two enantiopure diastereomers **216**) (126 mg, 0.30 mmol) and methyl 4-aminobicyclo[2.2.2]octane-1-carboxylate hydrochloride

(100 mg, 0.45 mmol, 1.5 eq.) in pyridine (3 mL). The reaction mixture was stirred at 80 °C overnight, cooled to RT and concentrated under reduced pressure. The residue was purified by preparative RP-HPLC (acetonitrile / 0.05% formic acid in water gradient) to give **232**. Yield: 133 mg (75% of theory). LC/MS (method 1): $t_R$ = 1.08 min, MS (ESIpos): *m*/*z* = 582 $[M+H]^+$; $^1$H NMR (400 MHz, DMSO-$d_6$): δ [ppm] = 8.02-7.96 (m, 1H), 7.96-7.89 (m, 1H), 7.75-7.69 (m, 2H), 7.47 / 7.42 (2x s, 1H), 6.46 / 6.44 (2x s, 1H), 5.64-5.52 (m, 1H), 3.88-3.76 (m, 1H), 3.63 / 3.62 (2x s, 3H), 3.56 (s, 3H), 3.28-3.00 (m, 2H), 2.15-1.88 (m, 2H), 1.88-1.68 (m, 13H), 1.62-1.51 (m, 1H), 1.46-1.29 (m, 3H), 1.28-1.11 (m, 1H).

**4-{2-[4-(5-Chloro-2-cyanophenyl)-5-methoxy-2-oxopyridin-1(2*H*)-yl]-3-[(2*S*)-tetrahydro-2*H*-pyran-2-yl]propanamido}bicyclo[2.2.2]octane-1-carboxylic acid (mixture of two enantiopure diastereomers 38).**

Cesium carbonate (147 mg, 0.45 mmol, 2.0 eq.) was added at RT to a mixture of methyl 4-{2-[4-(5-chloro-2-cyanophenyl)-5-methoxy-2-oxopyridin-1(2*H*)-yl]-3-[(2*S*)-tetrahydro-2*H*-pyran-2-yl]propanamido}bicyclo[2.2.2]octane-1-carboxylate (mixture of two enantiopure diastereomers **232**) (133 mg, 0.23 mmol) in methanol (4.4 mL) and water (1.1 mL). The reaction mixture was stirred at RT for 1 h and at 60 °C overnight and cooled to RT. Methanol was removed under reduced pressure. The residual suspension was diluted with water, acidified to pH 3 with aqueous hydrochloric acid solution (1 N) and extracted with ethyl acetate. The combined organic phases were dried over anhydrous magnesium sulfate, filtered and concentrated under reduced pressure. The residue was purified by preparative RP-HPLC (acetonitrile / 0.05% formic acid in water gradient) to give **38**. Yield: 92 mg (71% of theory). LC/MS (method 1): $t_R$ = 0.95 min, MS (ESIpos): *m*/*z* = 568 $[M+H]^+$; $^1$H NMR (400 MHz, DMSO-$d_6$): δ [ppm] = 12.0 (br s, 1H), 8.01-7.96 (m, 1H), 7.95-7.88 (m, 1H), 7.75-7.69 (m, 2H), 7.47 / 7.43 (2x s, 1H), 6.46 / 6.45 (2x s, 1H), 5.63-5.52 (m, 1H), 3.88-3.76 (m, 1H), 3.63 / 3.62 (2x s, 3H), 3.28-3.00 (m, 2H), 2.15-1.88 (m, 2H), 1.88-1.68 (m, 13H), 1.63-1.51 (m, 1H), 1.46-1.29 (m, 3H), 1.28-1.11 (m, 1H).

**Synthesis of mixture of two enantiopure diastereomers 39.**[a]

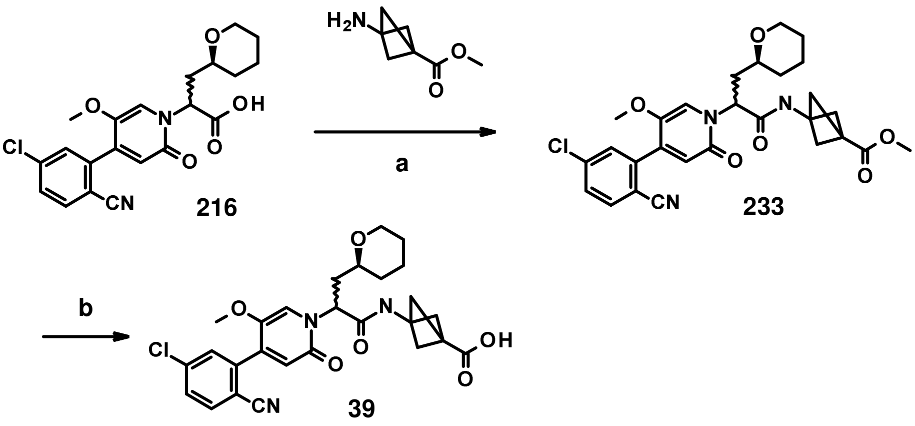

[a]Reagents and conditions: (a) T3P, DIEA, DMF, RT, 25%; (b) LiOH, THF/water, RT, 39%.

**Methyl 3-{2-[4-(5-chloro-2-cyanophenyl)-5-methoxy-2-oxopyridin-1(2*H*)-yl]-3-[(2*S*)-tetrahydro-2*H*-pyran-2-yl]propanamido}bicyclo[1.1.1]pentane-1-carboxylate (mixture of two enantiopure diastereomers 233).**

Propylphosphonic anhydride (T3P, 50% solution in *N,N*-dimethylformamide, 433 µL, 0.73 mmol, 3.0 eq.) was added under argon atmosphere at RT to a solution of 2-[4-(5-chloro-2-cyanophenyl)-5-methoxy-2-oxopyridin-1(2*H*)-yl]-3-[(2*S*)-tetrahydro-2*H*-pyran-2-yl]propanoic acid (mixture of two enantiopure diastereomers **216**) (102 mg, 0.24 mmol), methyl 3-aminobicyclo[1.1.1]pentane-1-carboxylate trifluoroacetate (121 mg, 77% purity, 0.36 mmol, 1.5 eq.) and *N,N*-diisopropylethylamine (127 µL, 0.73 mmol, 3.0 eq.) in *N,N*-dimethylformamide (3 mL). The reaction mixture was stirred at RT overnight, mixed with water and extracted with ethyl acetate. The combined organic phases were washed with brine, dried over anhydrous sodium sulfate, filtered and concentrated under reduced pressure. The residue was purified by preparative RP-HPLC (acetonitrile / water gradient) to give **233**. Yield: 33 mg (25% of theory). LC/MS (method 3): $t_R$ = 1.85 min / 1.88 min, MS (ESIpos): *m*/*z* = 540 $[M+H]^+$.

**3-{2-[4-(5-Chloro-2-cyanophenyl)-5-methoxy-2-oxopyridin-1(2*H*)-yl]-3-[(2*S*)-tetrahydro-2*H*-pyran-2-yl]propanamido}bicyclo[1.1.1]pentane-1-carboxylic acid (mixture of two enantiopure diastereomers 39).**

Lithium hydroxide (7 mg, 0.31 mmol, 5.0 eq.) was added at RT to a mixture of methyl 3-{2-[4-(5-chloro-2-cyanophenyl)-5-methoxy-2-oxopyridin-1(2*H*)-yl]-3-[(2*S*)-tetrahydro-2*H*-pyran-2-yl]propanamido}bicyclo[1.1.1]pentane-1-carboxylate (mixture of two enantiopure diastereomers **233**) (33 mg, 0.06 mmol) in a mixture of tetrahydrofuran and water (3:1, 1.6 mL). The reaction mixture was stirred at RT overnight, acidified with aqueous hydrochloric acid solution (1 N) and extracted with ethyl acetate. The combined organic phases were washed with brine, dried over anhydrous sodium sulfate, filtered and concentrated under reduced pressure. The residue was purified by preparative RP-HPLC

(acetonitrile / water gradient) to give **39**. Yield: 13 mg (39% of theory). LC/MS (method 3): $t_R$ = 1.64 min, MS (ESIpos): $m/z$ = 526 $[M+H]^+$; $^1H$ NMR (400 MHz, DMSO-$d_6$): δ [ppm] = 12.4 (br s, 1H), 8.99 / 8.93 (2x s, 1H), 7.99 (d, $J$ = 8.0 Hz, 1H), 7.75-7.69 (m, 2H), 7.43 / 7.39 (2x s, 1H), 6.47 / 6.45 (2x s, 1H), 5.57-5.50 / 5.50-5.41 (2x m, 1H), 3.86-3.77 (m, 1H), 3.64 (s, 3H), 3.3-2.92 (m, 2H, partly concealed), 2.30-1.99 (m, 2H), 2.19 / 2.17 (2x s, 6H), 1.79-1.65 (m, 1H), 1.64-1.47 (m, 1H), 1.46-1.29 (m, 3H), 1.26-1.13 (m, 1H).

**Synthesis of racemate 40.**[a]

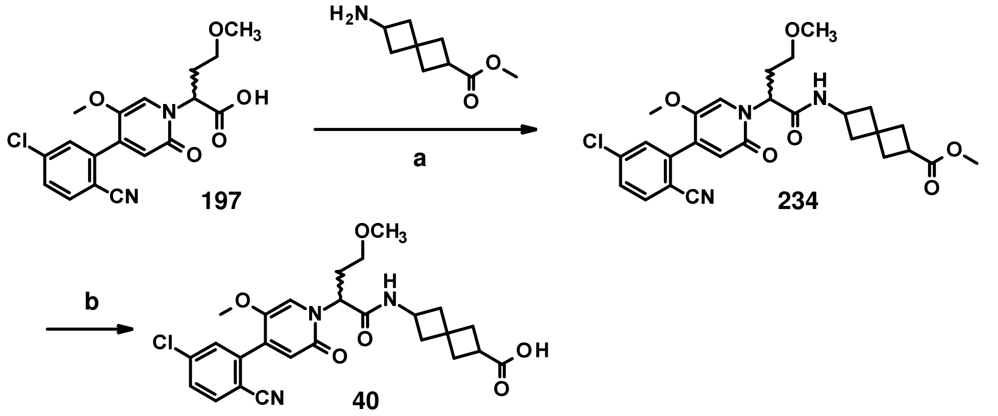


[a]Reagents and conditions: (a) T3P, pyridine, 50 °C, 75%; (b) LiOH × $H_2O$, THF/water, RT, 86%.

**Methyl 6-{2-[4-(5-chloro-2-cyanophenyl)-5-methoxy-2-oxopyridin-1(2*H*)-yl]-4-methoxybutanamido}spiro[3.3]heptane-2-carboxylate (racemate 234).**

Propylphosphonic anhydride (T3P, 50% solution in ethyl acetate, 252 µL, 1.06 mmol, 4.0 eq.) was added under argon atmosphere at RT to a solution of 2-[4-(5-chloro-2-cyanophenyl)-5-methoxy-2-oxopyridin-1(2*H*)-yl]-4-methoxybutanoic acid (racemate **197**) (100 mg, 0.27 mmol) and methyl 6-aminospiro[3.3]heptane-2-carboxylate hydrochloride (55 mg, 0.27 mmol, 1.0 eq.) in pyridine (1.5 mL). The reaction mixture was stirred at 50 °C overnight, cooled to RT and purified without further work-up by preparative RP-HPLC (acetonitrile / 0.1% formic acid in water gradient) to give **234**. Yield: 105 mg (75% of theory). LC/MS (method 3): $t_R$ = 1.76 min, MS (ESIpos): $m/z$ = 528 $[M+H]^+$; $^1H$ NMR (400 MHz, DMSO-$d_6$): δ [ppm] = 8.57 (d, $J$ = 7.5 Hz, 1H), 7.99 (d, $J$ = 8.7 Hz, 1H), 7.75-7.67 (m, 2H), 7.44 (s, 1H), 6.47 (s, 1H), 5.54-5.45 (m, 1H), 4.09-3.98 (m, 1H), 3.63 (s, 3H), 3.58 (s, 3H), 3.3-3.18 (m, 2H, partly concealed), 3.18 (s, 3H), 3.07-2.97 (m, 1H), 2.39-2.08 (m, 8H), 2.01-1.86 (m, 2H).

**6-{2-[4-(5-Chloro-2-cyanophenyl)-5-methoxy-2-oxopyridin-1(2*H*)-yl]-4-methoxybutanamido}-spiro[3.3]heptane-2-carboxylic acid (racemate 40).**

Lithium hydroxide monohydrate (17 mg, 0.39 mmol, 2.0 eq.) was added at RT to a mixture of methyl 6-{2-[4-(5-chloro-2-cyanophenyl)-5-methoxy-2-oxopyridin-1(2*H*)-yl]-4-

methoxybutanamido}spiro[3.3]heptane-2-carboxylate (racemate **234**) (104 mg, 0.20 mmol) in a mixture of tetrahydrofuran and water (3:1, 5.8 mL). The reaction mixture was stirred at RT for 5 h. Tetrahydrofuran was removed under reduced pressure. The residue was purified by preparative RP-HPLC (acetonitrile / 0.1% formic acid in water gradient) to give **40**. Yield: 87 mg (86% of theory). LC/MS (method 3): $t_R$ = 1.52 min, MS (ESIpos): $m/z$ = 514 $[M+H]^+$; $^1H$ NMR (400 MHz, DMSO-$d_6$): δ [ppm] = 12.0 (br s, 1H), 8.57 (d, $J$ = 7.0 Hz, 1H), 7.98 (d, $J$ = 8.8 Hz, 1H), 7.75-7.69 (m, 2H), 7.44 (s, 1H), 6.47 (s, 1H), 5.54-5.46 (m, 1H), 4.09-3.98 (m, 1H), 3.63 (s, 3H), 3.3-3.18 (m, 2H, partly concealed), 3.18 (s, 3H), 2.96-2.85 (m, 1H), 2.39-2.04 (m, 8H), 2.00-1.85 (m, 2H).

**Synthesis of mixture of racemic diastereomers 41.**[a]

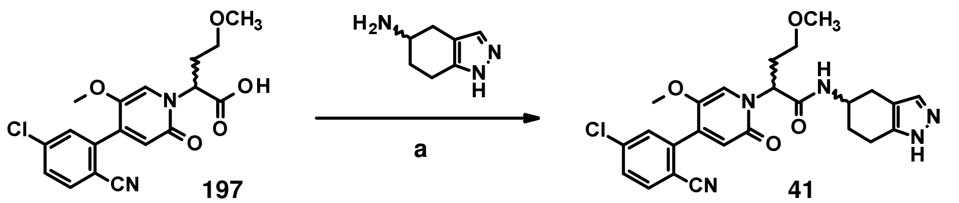

197 41

[a]Reagents and conditions: (a) T3P, pyridine, 70 °C, 20%.

**2-[4-(5-Chloro-2-cyanophenyl)-5-methoxy-2-oxopyridin-1(2*H*)-yl]-4-methoxy-*N*-(4,5,6,7-tetrahydro-1*H*-indazol-5-yl)butanamide (mixture of racemic diastereomers 41).**

Propylphosphonic anhydride (T3P, 50% solution in ethyl acetate, 316 µL, 0.53 mmol, 4.0 eq.) was added under argon atmosphere at RT to a solution of 2-[4-(5-chloro-2-cyanophenyl)-5-methoxy-2-oxopyridin-1(2*H*)-yl]-4-methoxybutanoic acid (racemate **197**) (50 mg, 0.13 mmol) and racemic 4,5,6,7-tetrahydro-1*H*-indazol-5-amine (27 mg, 0.20 mmol, 1.5 eq.) in pyridine (0.75 mL). The reaction mixture was stirred at 50 °C for 2 h and at 70 °C for 16 h, cooled to RT and purified without further work-up by preparative RP-HPLC (acetonitrile / water gradient) to give **41**. Yield: 13 mg (20% of theory). LC/MS (method 3): $t_R$ = 1.38 min, MS (ESIpos): $m/z$ = 496 $[M+H]^+$; $^1H$ NMR (400 MHz, DMSO-$d_6$): δ [ppm] = 8.48 (d, $J$ = 8.1 Hz, 1H), 7.99 (d, $J$ = 9.1 Hz, 1H), 7.75-7.69 (m, 2H), 7.50 (s, 1H), 7.33-7.23 (m, 1H), 6.48 (s, 1H), 5.65-5.57 (m, 1H), 4.00-3.89 (m, 1H), 3.65 (s, 3H), 3.3-3.22 (m, 2H, partly concealed), 3.19 (s, 3H), 2.79-2.58 (m, 3H), 2.45-2.16 (m, 3H), 1.97-1.67 (m, 2H).

**Synthesis of racemate 42.**[a]

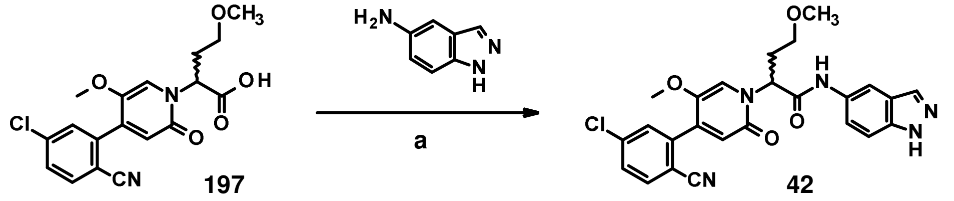

[a]Reagents and conditions: (a) HATU, DIEA, DMF, RT, 42%.

**2-[4-(5-Chloro-2-cyanophenyl)-5-methoxy-2-oxopyridin-1(2*H*)-yl]-*N*-(1*H*-indazol-5-yl)-4-methoxybutanamide (racemate 42).**

A solution of HATU (182 mg, 0.48 mmol, 1.2 eq.) in *N,N*-dimethylformamide (2 mL) was added under argon atmosphere at RT to a solution of 2-[4-(5-chloro-2-cyanophenyl)-5-methoxy-2-oxopyridin-1(2*H*)-yl]-4-methoxybutanoic acid (racemate **197**) (150 mg, 0.40 mmol), 1*H*-indazol-5-amine (58 mg, 0.44 mmol, 1.1 eq.) and *N,N*-diisopropylethylamine (153 $\mu$L, 0.88 mmol, 2.2 eq.) in *N,N*-dimethylformamide (7 mL). The reaction mixture was stirred at RT overnight and concentrated under reduced pressure. The residue was dissolved in ethyl acetate and water and extracted with ethyl acetate. The combined organic phases were dried over anhydrous magnesium sulfate, filtered and concentrated under reduced pressure. The residue was purified by preparative RP-HPLC (acetonitrile / water gradient) to give **42**. Yield: 88 mg (94% purity, 42% of theory). LC/MS (method 1): $t_R$ = 0.87 min, MS (ESIpos): $m/z$ = 492 $[M+H]^+$; $^1$H NMR (400 MHz, DMSO-$d_6$): δ [ppm] = 13.0 (br s, 1H), 10.46 (s, 1H), 8.14 (s, 1H), 8.04-7.97 (m, 2H), 7.76-7.70 (m, 2H), 7.56 (s, 1H), 7.49 (s, 2H), 6.53 (s, 1H), 5.84-5.77 (m, 1H), 3.69 (s, 3H), 3.44-3.36 (m, 1H), 3.35-3.26 (m, 1H, partly concealed), 3.22 (s, 3H), 2.45-2.34 (m, 2H).

**Synthesis of racemate 43.**[a]

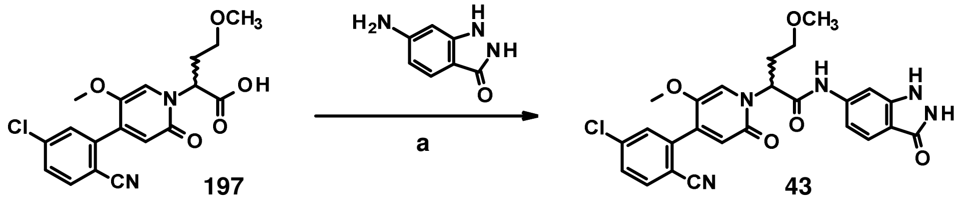

[a]Reagents and conditions: (a) HATU, DIEA, DMF, 40 °C, 52%.

**2-[4-(5-Chloro-2-cyanophenyl)-5-methoxy-2-oxopyridin-1(2*H*)-yl]-4-methoxy-*N*-(3-oxo-2,3-dihydro-1*H*-indazol-6-yl)butanamide (racemate 43).**

A solution of HATU (182 mg, 0.48 mmol, 1.2 eq.) in *N,N*-dimethylformamide (2 mL) was added under argon atmosphere at RT to a solution of 2-[4-(5-chloro-2-cyanophenyl)-5-methoxy-2-oxopyridin-1(2*H*)-yl]-4-methoxybutanoic acid (racemate **197**) (150 mg, 0.40 mmol), 6-amino-1,2-dihydro-3*H*-indazol-3-one (65 mg, 0.44 mmol, 1.1 eq.) and *N,N*-diisopropylethylamine (153 $\mu$L, 0.88 mmol,

2.2 eq.) in *N,N*-dimethylformamide (7 mL). The reaction mixture was stirred at 40 °C for 4 h and concentrated under reduced pressure. The residue was dissolved in ethyl acetate and water and extracted with ethyl acetate. The combined organic phases were dried over anhydrous magnesium sulfate, filtered and concentrated under reduced pressure. The residue was purified by flash silica gel chromatography (dichloromethane / methanol gradient) to give **43**. Yield: 105 mg (52% of theory). LC/MS (method 1): $t_R$ = 0.79 min, MS (ESIpos): *m*/*z* = 508 $[M+H]^+$; $^1H$ NMR (400 MHz, DMSO-$d_6$): δ [ppm] = 11.1 (br s, 1H), 10.55 (s, 1H), 10.5 (br s, 1H, partly concealed), 8.00 (d, *J* = 9.1 Hz, 1H), 7.86 (s, 1H), 7.77-7.70 (m, 2H), 7.57-7.50 (m, 2H), 7.07 (dd, *J* = 8.7 Hz, 1.6 Hz, 1H), 6.53 (s, 1H), 5.83-5.75 (m, 1H), 3.69 (s, 3H), 3.44-3.36 (m, 1H), 3.3-3.25 (m, 1H, partly concealed), 3.21 (s, 3H), 2.45-2.35 (m, 2H).

**Synthesis of eutomer 44.**[a]

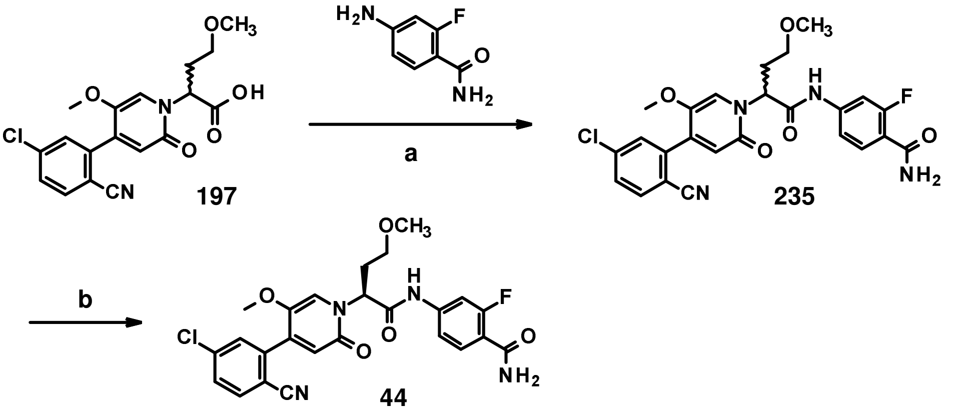


[a]Reagents and conditions: (a) T3P, pyridine, 50 °C, 98%; (b) enantiomer separation.

**4-{2-[4-(5-Chloro-2-cyanophenyl)-5-methoxy-2-oxopyridin-1(2*H*)-yl]-4-methoxybutanamido}-2-fluorobenzamide (racemate 235).**

Propylphosphonic anhydride (T3P, 50% solution in ethyl acetate, 574 µL, 0.96 mmol, 1.6 eq.) was added under argon atmosphere at RT to a solution of 2-[4-(5-chloro-2-cyanophenyl)-5-methoxy-2-oxopyridin-1(2*H*)-yl]-4-methoxybutanoic acid (racemate **197**) (250 mg, 0.60 mmol) and 4-amino-2-fluorobenzamide (144 mg, 0.91 mmol, 1.5 eq.) in pyridine (2 mL). The reaction mixture was stirred at 50 °C for 1 h, cooled to RT and purified without further work-up by preparative RP-HPLC (acetonitrile / 0.1% formic acid in water gradient) to give **235**. Yield: 302 mg (98% of theory). LC/MS (method 1): $t_R$ = 0.83 min, MS (ESIpos): *m*/*z* = 513 $[M+H]^+$; $^1H$ NMR (400 MHz, DMSO-$d_6$): δ [ppm] = 10.80 (s, 1H), 8.00 (d, *J* = 9.1 Hz, 1H), 7.76-7.63 (m, 4H), 7.57-7.47 (m, 3H), 7.44 (dd, *J* = 8.6 Hz, 2.0 Hz, 1H), 6.53 (s, 1H), 5.77-5.69 (m, 1H), 3.69 (s, 3H), 3.44-3.36 (m, 1H), 3.3-3.24 (m, 1H, partly concealed), 3.20 (s, 3H), 2.46-2.37 (m, 2H).

**4-({(2*S*)-2-[4-(5-Chloro-2-cyanophenyl)-5-methoxy-2-oxopyridin-1(2*H*)-yl]-4-methoxybutanoyl}amino)-2-fluorobenzamide (eutomer 44).**

Enantiomer separation of 300 mg of 4-{2-[4-(5-chloro-2-cyanophenyl)-5-methoxy-2-oxopyridin-1(2*H*)-yl]-4-methoxybutanamido}-2-fluorobenzamide (racemate **235**) gave 120 mg of eutomer **44** (chiral HPLC: $t_R$ = 3.49 min, >99% ee) and 117 mg of distomer (chiral HPLC: $t_R$ = 4.47 min).
LC/MS (method 1): $t_R$ = 0.83 min, MS (ESIpos): $m/z$ = 513 $[M+H]^+$; $^1H$ NMR (400 MHz, DMSO-$d_6$): δ [ppm] = 10.80 (s, 1H), 8.00 (d, *J* = 9.2 Hz, 1H), 7.76-7.63 (m, 4H), 7.57-7.47 (m, 3H), 7.44 (dd, *J* = 8.6 Hz, 2.0 Hz, 1H), 6.53 (s, 1H), 5.76-5.69 (m, 1H), 3.69 (s, 3H), 3.44-3.36 (m, 1H), 3.3-3.24 (m, 1H, partly concealed), 3.20 (s, 3H), 2.46-2.37 (m, 2H).
Separation method: SFC: column: Daicel Chiralpak IC-H 5 μm, 250 mm × 20 mm; mobile phase: 80% carbon dioxide / 20% ethanol; temperature: 40 °C; flow rate: 80 mL/min; UV detection: 210 nm.
Analytical method: SFC: column: Daicel Chiralpak IC-H 5 μm, 250 mm × 4.6 mm; mobile phase: 70% carbon dioxide / 30% ethanol; temperature: 40 °C; flow rate: 3 mL/min; UV detection: 210 nm.

**Synthesis of eutomer 45.**[a]

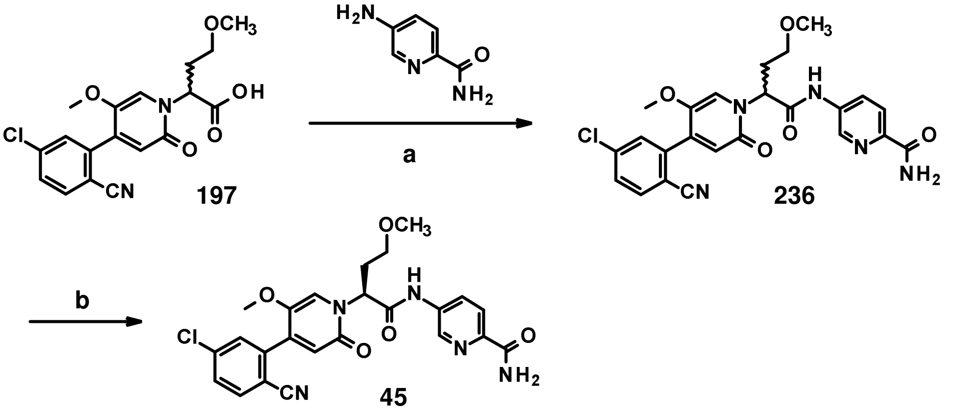


[a]Reagents and conditions: (a) T3P, pyridine, 50 °C, 96%; (b) enantiomer separation.

**5-{2-[4-(5-Chloro-2-cyanophenyl)-5-methoxy-2-oxopyridin-1(2*H*)-yl]-4-methoxybutanamido}pyridine-2-carboxamide (racemate 236).**

Propylphosphonic anhydride (T3P, 50% solution in ethyl acetate, 574 μL, 0.96 mmol, 1.6 eq.) was added under argon atmosphere at RT to a solution of 2-[4-(5-chloro-2-cyanophenyl)-5-methoxy-2-oxopyridin-1(2*H*)-yl]-4-methoxybutanoic acid (racemate **197**) (250 mg, 0.60 mmol) and 5-aminopyridine-2-carboxamide (131 mg, 0.91 mmol, 1.5 eq.) in pyridine (2 mL). The reaction mixture was stirred at 50 °C for 1 h, cooled to RT and purified without further work-up by preparative RP-HPLC (acetonitrile / 0.1% formic acid in water gradient) to give **236**. Yield: 287 mg (96% of theory). LC/MS (method 1): $t_R$ = 0.79 min, MS (ESIpos): $m/z$ = 496 $[M+H]^+$; $^1H$ NMR (400 MHz, DMSO-$d_6$): δ [ppm] = 10.89 (s, 1H), 8.86 (d, *J* = 2.3 Hz, 1H), 8.23 (dd, *J* = 8.6 Hz, 2.5 Hz, 1H), 8.05-7.96 (m, 3H), 7.77-7.70 (m, 2H), 7.56-

7.48 (m, 2H), 6.54 (s, 1H), 5.79-5.71 (m, 1H), 3.69 (s, 3H), 3.46-3.37 (m, 1H), 3.3-3.24 (m, 1H, partly concealed), 3.21 (s, 3H), 2.5-2.38 (m, 2H, partly concealed).

**5-({(2*S*)-2-[4-(5-Chloro-2-cyanophenyl)-5-methoxy-2-oxopyridin-1(2*H*)-yl]-4-methoxybutanoyl}amino)pyridine-2-carboxamide (eutomer 45).**

Enantiomer separation of 285 mg of 5-{2-[4-(5-chloro-2-cyanophenyl)-5-methoxy-2-oxopyridin-1(2*H*)-yl]-4-methoxybutanamido}pyridine-2-carboxamide (racemate **236**) gave 114 mg of distomer (chiral HPLC: $t_R$ = 2.12 min) and 117 mg of eutomer **45** (chiral HPLC: $t_R$ = 2.50 min, 97% ee).
LC/MS (method 3): $t_R$ = 1.43 min, MS (ESIpos): $m/z$ = 496 $[M+H]^+$; $^1$H NMR (400 MHz, DMSO-$d_6$): δ [ppm] = 10.89 (s, 1H), 8.86 (d, $J$ = 2.2 Hz, 1H), 8.23 (dd, $J$ = 8.6 Hz, 2.5 Hz, 1H), 8.05-7.96 (m, 3H), 7.77-7.71 (m, 2H), 7.56-7.48 (m, 2H), 6.54 (s, 1H), 5.79-5.72 (m, 1H), 3.69 (s, 3H), 3.46-3.3 (m, 1H, partly concealed), 3.3-3.24 (m, 1H, partly concealed), 3.21 (s, 3H), 2.5-2.38 (m, 2H, partly concealed).
Separation method: SFC: column: Daicel Chiralpak OD-H 5 μm, 250 mm × 20 mm; mobile phase: 86% carbon dioxide / 14% methanol; temperature: 40 °C; flow rate: 80 mL/min; UV detection: 210 nm.
Analytical method: SFC: column: Daicel Chiralpak OD-H 5 μm, 250 mm × 4.6 mm; mobile phase: 80% carbon dioxide / 20% methanol; temperature: 40 °C; flow rate: 3 mL/min; UV detection: 210 nm.

**Synthesis of eutomer 46.**[a]

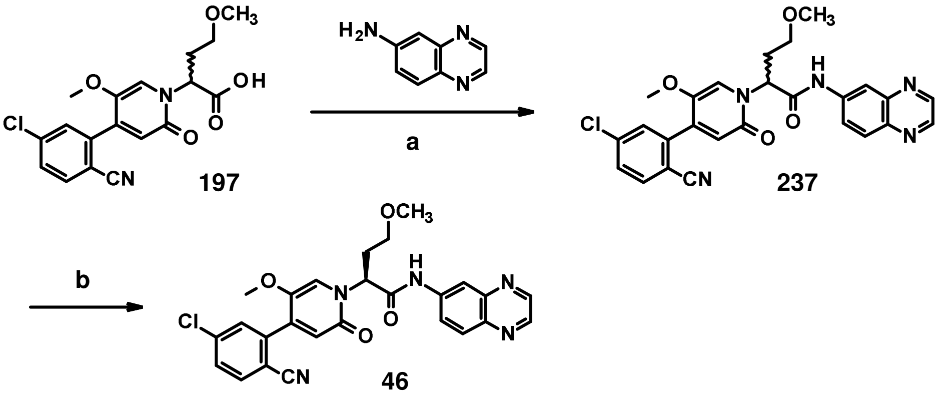


[a]Reagents and conditions: (a) T3P, pyridine, 60 °C, 96%; (b) enantiomer separation.

**2-[4-(5-Chloro-2-cyanophenyl)-5-methoxy-2-oxopyridin-1(2*H*)-yl]-4-methoxy-*N*-(quinoxalin-6-yl)butanamide (racemate 237).**

Propylphosphonic anhydride (T3P, 50% solution in ethyl acetate, 229 μL, 0.39 mmol, 1.6 eq.) was added under argon atmosphere at 60 °C to a solution of 2-[4-(5-chloro-2-cyanophenyl)-5-methoxy-2-oxopyridin-1(2*H*)-yl]-4-methoxybutanoic acid (racemate **197**) (100 mg, 0.24 mmol) and quinoxalin-6-amine (53 mg, 0.36 mmol, 1.5 eq.) in pyridine (2 mL). The reaction mixture was stirred at 60 °C until complete conversion, cooled to RT and purified without further work-up by preparative RP-HPLC (acetonitrile / 0.1% formic acid in water gradient) to give **237**. Yield: 117 mg (96% of theory). LC/MS

(method 3): $t_R$ = 1.63 min, MS (ESIpos): $m/z$ = 504 $[M+H]^+$; $^1H$ NMR (400 MHz, DMSO-$d_6$): δ [ppm] = 10.95 (s, 1H), 8.90 (d, $J$ = 2.0 Hz, 1H), 8.84 (d, $J$ = 1.8 Hz, 1H), 8.55 (d, $J$ = 2.2 Hz, 1H), 8.08 (d, $J$ = 9.2 Hz, 1H), 8.05-7.97 (m, 2H), 7.77-7.70 (m, 2H), 7.56 (s, 1H), 6.55 (s, 1H), 5.86-5.78 (m, 1H), 3.71 (s, 3H), 3.48-3.38 (m, 1H), 3.36-3.26 (m, 1H, partly concealed), 3.22 (s, 3H), 2.5-2.39 (m, 2H, partly concealed).

**(2*S*)-2-[4-(5-Chloro-2-cyanophenyl)-5-methoxy-2-oxopyridin-1(2*H*)-yl]-4-methoxy-*N*-(quinoxalin-6-yl)butanamide (eutomer 46).**

Enantiomer separation of 163 mg of 2-[4-(5-chloro-2-cyanophenyl)-5-methoxy-2-oxopyridin-1(2*H*)-yl]-4-methoxy-*N*-(quinoxalin-6-yl)butanamide (racemate **237**) gave 57 mg of distomer (chiral HPLC: $t_R$ = 0.92 min) and 55 mg of eutomer **46** (chiral HPLC: $t_R$ = 1.18 min, 97% ee).

LC/MS (method 3): $t_R$ = 1.63 min, MS (ESIpos): $m/z$ = 504 $[M+H]^+$; $^1H$ NMR (400 MHz, DMSO-$d_6$): δ [ppm] = 10.95 (s, 1H), 8.90 (d, $J$ = 1.6 Hz, 1H), 8.84 (d, $J$ = 1.7 Hz, 1H), 8.55 (d, $J$ = 2.1 Hz, 1H), 8.08 (d, $J$ = 9.2 Hz, 1H), 8.04-7.97 (m, 2H), 7.78-7.71 (m, 2H), 7.56 (s, 1H), 6.55 (s, 1H), 5.85-5.78 (m, 1H), 3.71 (s, 3H), 3.48-3.39 (m, 1H), 3.37-3.25 (m, 1H, partly concealed), 3.22 (s, 3H), 2.5-2.40 (m, 2H, partly concealed).

Separation method: SFC: column: Daicel Chiralpak OJ-H 5 μm, 250 mm × 30 mm; mobile phase: 80% carbon dioxide / 20% methanol; temperature: 40 °C; flow rate: 100 mL/min; UV detection: 210 nm.

Analytical method: SFC: column: Daicel Chiralpak OJ-H 5 μm, 250 mm × 4.6 mm; mobile phase: 80% carbon dioxide / 20% methanol; temperature: 40 °C; flow rate: 3 mL/min; UV detection: 210 nm.

**Synthesis of racemate 48 and eutomer 47.**[a]

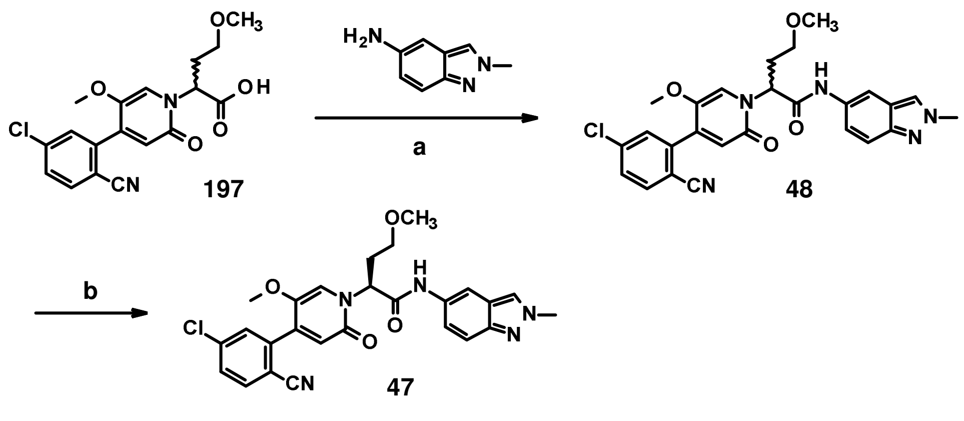


[a]Reagents and conditions: (a) T3P, pyridine, 50 °C, 84%; (b) enantiomer separation.

**2-[4-(5-Chloro-2-cyanophenyl)-5-methoxy-2-oxopyridin-1(2*H*)-yl]-4-methoxy-*N*-(2-methyl-2*H*-indazol-5-yl)butanamide (racemate 48).**

Propylphosphonic anhydride (T3P, 50% solution in ethyl acetate, 574 $\mu$L, 0.96 mmol, 1.6 eq.) was added under argon atmosphere at RT to a solution of 2-[4-(5-chloro-2-cyanophenyl)-5-methoxy-2-oxopyridin-1(2*H*)-yl]-4-methoxybutanoic acid (racemate **197**) (250 mg, 0.60 mmol) and 2-methyl-2*H*-indazol-5-amine (133 mg, 0.91 mmol, 1.5 eq.) in pyridine (2 mL). The reaction mixture was stirred at 50 °C for 1 h, cooled to RT and purified without further work-up by preparative RP-HPLC (acetonitrile / 0.1% formic acid in water gradient) to give **48**. Yield: 257 mg (84% of theory). LC/MS (method 1): $t_R$ = 0.89 min, MS (ESIpos): *m*/*z* = 506 $[M+H]^+$; $^1$H NMR (400 MHz, DMSO-$d_6$): δ [ppm] = 10.39 (s, 1H), 8.25 (s, 1H), 8.14 (d, *J* = 1.2 Hz, 1H), 8.00 (d, *J* = 9.0 Hz, 1H), 7.76-7.69 (m, 2H), 7.58-7.51 (m, 2H), 7.31 (dd, *J* = 9.3 Hz, 2.0 Hz, 1H), 6.53 (s, 1H), 5.84-5.75 (m, 1H), 4.13 (s, 3H), 3.69 (s, 3H), 3.44-3.35 (m, 1H), 3.34-3.25 (m, 1H, partly concealed), 3.22 (s, 3H), 2.44-2.34 (m, 2H).

**(2*S*)-2-[4-(5-Chloro-2-cyanophenyl)-5-methoxy-2-oxopyridin-1(2*H*)-yl]-4-methoxy-*N*-(2-methyl-2*H*-indazol-5-yl)butanamide (eutomer 47).**

Enantiomer separation of 255 mg of 2-[4-(5-chloro-2-cyanophenyl)-5-methoxy-2-oxopyridin-1(2*H*)-yl]-4-methoxy-*N*-(2-methyl-2*H*-indazol-5-yl)butanamide (racemate **48**) gave 131 mg of distomer (chiral HPLC: $t_R$ = 2.14 min) and 107 mg of eutomer **47** (chiral HPLC: $t_R$ = 2.76 min, >99% ee).
LC/MS (method 3): $t_R$ = 1.55 min, MS (ESIpos): *m*/*z* = 506 $[M+H]^+$; $^1$H NMR (400 MHz, DMSO-$d_6$): δ [ppm] = 10.39 (s, 1H), 8.25 (s, 1H), 8.14 (d, *J* = 1.2 Hz, 1H), 8.00 (d, *J* = 9.1 Hz, 1H), 7.76-7.70 (m, 2H), 7.58-7.52 (m, 2H), 7.31 (dd, *J* = 9.3 Hz, 2.0 Hz, 1H), 6.53 (s, 1H), 5.84-5.76 (m, 1H), 4.13 (s, 3H), 3.69 (s, 3H), 3.44-3.25 (m, 2H, partly concealed), 3.22 (s, 3H), 2.44-2.34 (m, 2H).
Separation method: SFC: column: Daicel Chiralpak OD-H 5 $\mu$m, 250 mm × 20 mm; mobile phase: 80% carbon dioxide / 20% methanol; temperature: 40 °C; flow rate: 80 mL/min; UV detection: 210 nm.
Analytical method: SFC: column: Daicel Chiralpak OD-H 5 $\mu$m, 250 mm × 4.6 mm; mobile phase: 80% carbon dioxide / 20% methanol; temperature: 40 °C; flow rate: 3 mL/min; UV detection: 210 nm.

**Compounds of Table 5.**

**Synthesis of heteroaryl-substituted 2-bromo-4-chloroaryls.**

**3-(2-Bromo-4-chlorophenyl)-4,5-dihydro-1,2-oxazole (241).**[a]

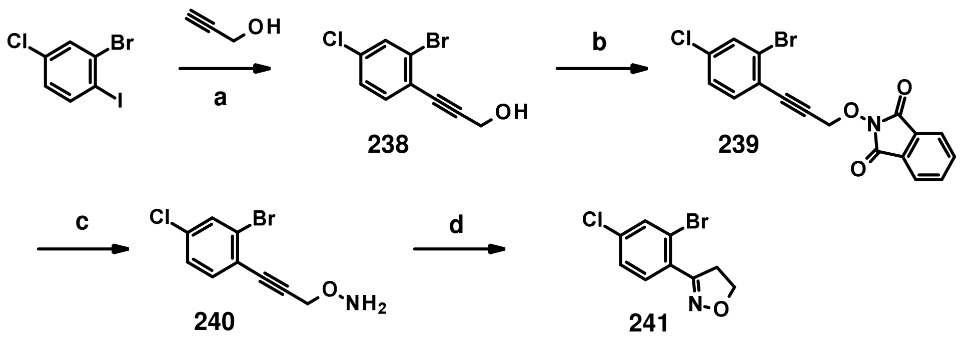

[a]Reagents and conditions: (a) $(Ph_3P)_2PdCl_2$, CuI, diethylamine, RT, 76%; (b) *N*-hydroxyphthalimide, $Ph_3P$, DIAD, DCM, 0 °C → RT, 66%; (c) $N_2H_4 \times H_2O$, DCM, 0 °C → RT, 91%; (d) [(2-biphenyl)di-*tert*-butylphosphine]gold(I) hexafluoroantimonate acetonitrile monoadduct, DCM, RT, 73%.

**3-(2-Bromo-4-chlorophenyl)prop-2-yn-1-ol (238).**

A mixture of 2-bromo-4-chloro-1-iodobenzene (2.00 g, 6.30 mmol), prop-2-yn-1-ol (451 $\mu$L, 7.56 mmol, 1.2 eq.), bis(triphenylphosphine)palladium(II) dichloride (137 mg, 0.19 mmol, 0.03 eq.) and copper(I) iodide (60 mg, 0.32 mmol, 0.05 eq.) in diethylamine (32 mL) was stirred at RT overnight, cooled with an ice bath and mixed with dichloromethane (100 mL) and water (100 mL). The aqueous phase was extracted with dichloromethane. The combined organic phases were washed with water and brine, dried over anhydrous sodium sulfate, filtered and concentrated under reduced pressure. The residue was purified by flash silica gel chromatography (cyclohexane / ethyl acetate gradient) to give **238**. Yield: 1.17 g (76% of theory). GC/MS (method 9): $t_R$ = 5.85 min, MS (EI): *m*/*z* = 244 $[M]^+$; $^1$H NMR (400 MHz, DMSO-$d_6$): δ [ppm] = 7.86 (d, *J* = 2.1 Hz, 1H), 7.55 (d, *J* = 8.4 Hz, 1H), 7.48 (dd, *J* = 8.4 Hz, 2.1 Hz, 1H), 5.42 (t, *J* = 6.1 Hz, 1H), 4.35 (d, *J* = 6.1 Hz, 2H).

**2-{[3-(2-Bromo-4-chlorophenyl)prop-2-yn-1-yl]oxy}-1*H*-isoindole-1,3(2*H*)-dione (239).**

Diisopropyl azodicarboxylate (DIAD, 1.80 mL, 9.17 mmol, 1.5 eq.) was added at 0 °C to a solution of 3-(2-bromo-4-chlorophenyl)prop-2-yn-1-ol (**238**) (1.50 g, 6.11 mmol), *N*-hydroxyphthalimide (1.20 g, 7.33 mmol, 1.2 eq.) and triphenylphosphine (2.40 g, 9.17 mmol, 1.5 eq.) in dichloromethane (24 mL). The reaction mixture was stirred at 0 °C for 30 min, allowed to warm to RT overnight and concentrated under reduced pressure. The residue was purified by flash silica gel chromatography (cyclohexane / ethyl acetate gradient) to give **239**. Yield: 1.63 g (66% of theory). LC/MS (method 8): $t_R$ = 2.17 min, MS (ESIpos): *m*/*z* = 390 $[M+H]^+$; $^1$H NMR (400 MHz, DMSO-$d_6$): δ [ppm] = 7.94-7.84 (m, 5H), 7.59-7.47 (m, 2H), 5.20 (s, 2H).

**1-[3-(Aminooxy)prop-1-yn-1-yl]-2-bromo-4-chlorobenzene (240).**

Hydrazine hydrate (974 µL, 20.03 mmol, 5.0 eq.) was added at 0 °C to a solution of 2-{[3-(2-bromo-4-chlorophenyl)prop-2-yn-1-yl]oxy}-1*H*-isoindole-1,3(2*H*)-dione (**239**) (1.63 g, 4.01 mmol) in dichloromethane (20 mL). The mixture was stirred at 0 °C for 10 min, at RT overnight and diluted with aqueous sodium carbonate solution (5%, 20 mL). After phase separation, the aqueous phase was extracted with ethyl acetate. The combined organic phases were washed with brine, dried over anhydrous sodium sulfate, filtered and concentrated under reduced pressure. The residue was purified by flash silica gel chromatography (cyclohexane / ethyl acetate mixture) to give **240**. Yield: 997 mg (91% of theory). LC/MS (method 3): $t_R$ = 1.77 min, MS (ESIpos): *m*/*z* = 260 $[M+H]^+$; $^1$H NMR (400 MHz, DMSO-$d_6$): δ [ppm] = 7.89-7.87 (m, 1H), 7.62-7.58 (m, 1H), 7.52-7.48 (m, 1H), 6.26 (s, 2H), 4.47 (s, 2H).

**3-(2-Bromo-4-chlorophenyl)-4,5-dihydro-1,2-oxazole (241).**

[(2-Biphenyl)di-*tert*-butylphosphine]gold(I) hexafluoroantimonate acetonitrile monoadduct (56 mg, 0.07 mmol, 0.02 eq.) was added at RT to a solution of 1-[3-(aminooxy)prop-1-yn-1-yl]-2-bromo-4-chlorobenzene (**240**) (997 mg, 3.65 mmol) in dichloromethane (39 mL). The reaction mixture was stirred at RT for 30 min, mixed with triethylamine (509 µL, 3.65 mmol, 1.0 eq.) and filtered through silica gel. The filter residue was washed with dichloromethane. The combined filtrates were concentrated under reduced pressure. The residue was purified by flash silica gel chromatography (cyclohexane / ethyl acetate gradient) to give **241**. Yield: 706 mg (73% of theory). LC/MS (method 20): $t_R$ = 2.77 min, MS (ESIpos): *m*/*z* = 260 $[M+H]^+$; $^1$H NMR (400 MHz, DMSO-$d_6$): δ [ppm] = 7.91-7.89 (m, 1H), 7.60-7.56 (m, 2H), 4.42 (t, *J* = 10.2 Hz, 2H), 3.43 (t, *J* = 10.2 Hz, 2H).

**3-(2-Bromo-4-chlorophenyl)-1,2-oxazole (242).**[a]

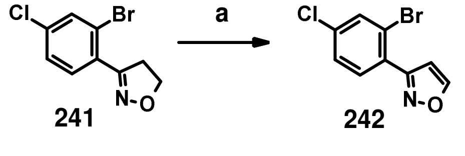


[a]Reagents and conditions: (a) $MnO_2$, toluene/1,4-dioxane, RF, 55%.

Manganese (IV)oxide (3.34 g, 38.40 mmol, 15 eq.) was added at RT to a solution of 3-(2-bromo-4-chlorophenyl)-4,5-dihydro-1,2-oxazole (**241**) (667 mg, 2.56 mmol) in a mixture of toluene and 1,4-dioxane (10:1, 37 mL). The reaction mixture was heated at reflux for 24 h (Dean-Stark water separator), mixed with additional manganese (IV)oxide (900 mg, 10.35 mmol, 4.0 eq.), heated at reflux for further 24 h, cooled to RT, diluted with methanol and filtered through kieselguhr. The filtrate was concentrated under reduced pressure. The residue was purified by flash silica gel chromatography (cyclohexane / ethyl acetate gradient) to give **242**. Yield: 380 mg (95% purity, 55% of theory). $^1$H NMR (400 MHz,

DMSO-$d_6$): δ [ppm] = 9.09 (d, $J$ = 1.7 Hz, 1H), 7.98 (d, $J$ = 1.2 Hz, 1H), 7.69-7.61 (m, 2H), 6.98 (d, $J$ = 1.7 Hz, 1H).

**5-(2-Bromo-4-chlorophenyl)-1,3-oxazole (243).**[a]

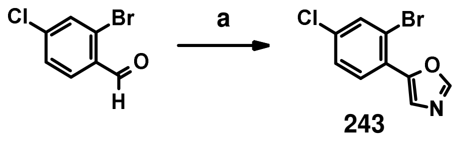

[a]Reagents and conditions: (a) isocyanomethyl 4-methylphenyl sulfone, $K_2CO_3$, MeOH, 75 °C; 82%.

A mixture of 2-bromo-4-chlorobenzaldehyde (10.00 g, 45.56 mmol), isocyanomethyl 4-methylphenyl sulfone (9.78 g, 50.12 mmol, 1.1 eq.) and potassium carbonate (12.70 g, 91.13 mmol, 2.0 eq.) in methanol (100 mL) was stirred at 75 °C overnight, cooled to RT and added to cold water. The forming precipitate was filtered, dried *in vacuo* and recrystallized from hexane to give **243**. Yield: 9.80 g (82% of theory). LC/MS (method 19): $t_R$ = 2.18 min, MS (ESIpos): $m/z$ = 258 $[M+H]^+$; $^1H$ NMR (400 MHz, DMSO-$d_6$): δ [ppm] = 8.57 (s, 1H), 7.91 (s, 1H), 7.83 (s, 1H), 7.75 (d, 1H), 7.58 (d, 1H).

**4-(2-Bromo-4-chlorophenyl)-1,3-oxazole (245).**[a]

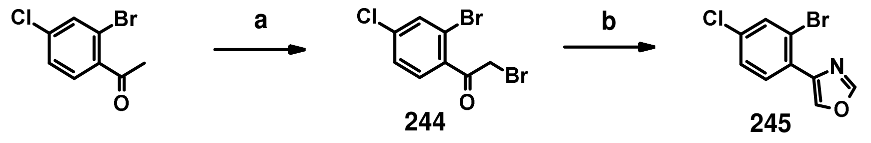

[a]Reagents and conditions: (a) $Br_2$, glacial AcOH, 40 °C, 79%; (b) ammonium formate, HCOOH, RF, 21%.

**2-Bromo-1-(2-bromo-4-chlorophenyl)ethenone (244).**

Bromine (1.10 mL, 21.41 mmol, 1.0 eq.) was added dropwise at RT to a mixture of 2-bromo-4-chloroacetophenone (5.00 g, 21.41 mmol) in glacial acetic acid (21.5 mL). The reaction mixture was stirred at RT for 30 min, warmed to 40 °C (discoloration and exothermic!) and kept below 50 °C by cooling with cold water. After 1.5 h, after the reaction had gone to completion and the temperature returned to RT, the mixture was concentrated under reduced pressure (at <40 °C) to give **244** which was used without further purification. Yield: 6.60 g (80% purity, 79% of theory). LC/MS (method 5): $t_R$ = 1.38 min, MS (ESIpos): $m/z$ = 311 $[M+H]^+$.

**4-(2-Bromo-4-chlorophenyl)-1,3-oxazole (245).**

Anhydrous ammonium formate (4.26 g, 67.61 mmol, 4.0 eq.) was added at RT to a mixture of 2-bromo-1-(2-bromo-4-chlorophenyl)ethanone (**244**) (6.60 g, 16.90 mmol) in formic acid (21.0 mL). The reaction

mixture was heated at reflux for 8 h. Residual formic acid was removed under reduced pressure. The residue was diluted with water and ethyl acetate, and the mixture was cautiously made alkaline using sodium carbonate. After phase separation, and the aqueous phase was extracted with ethyl acetate. The combined organic phases were washed with brine, dried and concentrated under reduced pressure. The residue was purified by flash silica gel chromatography (petroleum ether / ethyl acetate gradient) and subsequently by preparative RP-HPLC (acetonitrile / 0.1% formic acid in water gradient) to give **245**. Yield: 0.90 g (21% of theory). LC/MS (method 1): $t_R$ = 1.15 min, MS (ESIpos): $m/z$ = 258 $[M+H]^+$; $^1H$ NMR (400 MHz, DMSO-$d_6$): δ [ppm] = 8.77 (d, $J$ = 0.6 Hz, 1H), 8.57 (d, $J$ = 0.7 Hz, 1H), 7.96 (d, $J$ = 8.6 Hz, 1H), 7.89 (d, $J$ = 2.2 Hz, 1H), 7.60 (dd, $J$ = 8.4 Hz, 2.2 Hz, 1H).

**2-(2-Bromo-4-chlorophenyl)-1,3,4-oxadiazole (247).**[a]

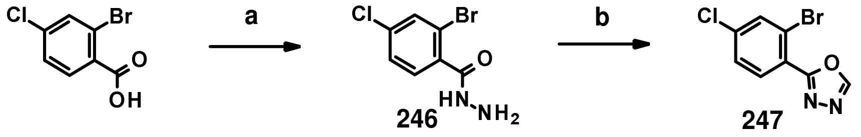


[a]Reagents and conditions: (a) i) CDI, DMAP, THF, 70 °C, ii) $N_2H_4$, RT, 82%; (b) p-TsOH × $H_2O$, $HC(OEt)_3$, RF, 80%.

**2-Bromo-4-chlorobenzohydrazide (246).**

1,1´-Carbonyldiimidazole (1.50 g, 9.27 mmol, 1.5 eq.) and 4-dimethylaminopyridine (0.38 g, 3.09 mmol, 0.5 eq.) were added under argon atmosphere at RT to a mixture of 2-bromo-4-chlorobenzoic acid (1.50 g, 6.18 mmol) in tetrahydrofuran (58 mL). The reaction mixture was stirred at 70 °C for 3 h, cooled to RT and mixed (rapidly in one portion to prevent dimer formation) with hydrazine solution (1 M in tetrahydrofuran, 8.03 mL, 8.03 mmol, 1.3 eq.) (slightly exothermic!). The mixture was stirred at RT for 75 min, mixed with additional hydrazine solution (1 M in tetrahydrofuran, 8.03 mL, 8.03 mmol, 1.3 eq.), stirred at RT for further 30 min and diluted with dichloromethane (60 mL) and saturated aqueous sodium bicarbonate solution (60 mL). After phase separation, the aqueous phase was extracted with dichloromethane. The combined organic phases were washed with water, dried over anhydrous magnesium sulfate, filtered and concentrated under reduced pressure. The residue was purified by flash silica gel chromatography (dichloromethane / methanol gradient) to give **246**. Yield: 1.30 g (82% of theory). LC/MS (method 8): $t_R$ = 1.11 min, MS (ESIpos): $m/z$ = 249 $[M+H]^+$; $^1H$ NMR (400 MHz, DMSO-$d_6$): δ [ppm] = 9.58 (br s, 1H), 7.81 (d, $J$ = 2.0 Hz, 1H), 7.52 (dd, $J$ = 8.2 Hz, 2.1 Hz, 1H), 7.37 (d, $J$ = 8.2 Hz, 1H), 4.49 (br s, 2H).

**2-(2-Bromo-4-chlorophenyl)-1,3,4-oxadiazole (247).**

para-Toluenesulfonic acid monohydrate (20 mg) was added at RT to a mixture of 2-bromo-4-chlorobenzohydrazide (**246**) (1.30 g, 5.05 mmol) in triethyl orthoformate (16.81 mL, 101.08 mmol,

20 eq.). The reaction mixture was heated at reflux overnight and cooled to RT. The precipitate formed was filtered with suction and washed with pentane to give a first batch of **247**. The combined filtrates were concentrated under reduced pressure. The residue was stirred in pentane, and the solid was filtered with suction, washed with pentane and dried *in vacuo* to give a second batch of **247**. Total yield: 1.11 g (80% of theory). LC/MS (method 1): $t_R$ = 0.85 min, MS (ESIpos): *m*/*z* = 259 $[M+H]^+$; $^1$H NMR (400 MHz, DMSO-$d_6$): δ [ppm] = 9.48 (s, 1H), 8.07 (s, 1H), 7.95 (d, *J* = 8.4 Hz, 1H), 7.72 (d, *J* = 8.4 Hz, 1H).

**1-(2-Bromo-4-chlorophenyl)-1*H*-tetrazole (248).**[a]

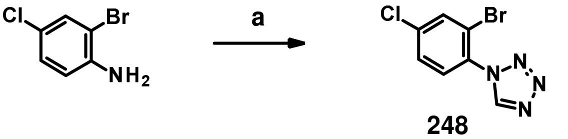

**248**

[a]Reagents and conditions: (a) $NaN_3$, $HC(OEt)_3$, AcOH, 80 °C, 53%.

Triethyl orthoformate (1.28 mL, 7.69 mmol, 3.0 eq.) was added under argon atmosphere at RT to a mixture of 2-bromo-4-chloroaniline (529 mg, 2.56 mmol) and sodium azide (500 mg, 7.69 mmol, 3.0 eq.) in acetic acid (26 mL). The reaction mixture was stirred at 80 °C for 3 h, at RT overnight and concentrated under reduced pressure. The residue was stirred in saturated aqueous sodium bicarbonate solution (17.5 mL). The aqueous phase was extracted with diethyl ether. The combined organic phases were dried over anhydrous sodium sulfate, filtered and concentrated under reduced pressure. The residue was purified by flash silica gel chromatography (cyclohexane / ethyl acetate gradient) to give **248**. Yield: 436 mg (81% purity, 53% of theory). LC/MS (method 1): $t_R$ = 0.84 min, MS (ESIpos): *m*/*z* = 259 $[M+H]^+$; $^1$H NMR (400 MHz, DMSO-$d_6$): δ [ppm] = 9.91 (s, 1H), 8.18 (d, *J* = 2.2 Hz, 1H), 7.84 (d, *J* = 8.6 Hz, 1H), 7.79 (d, *J* = 8.4 Hz, 2.2 Hz, 1H).

**1-(2-Bromo-4-chlorophenyl)-1*H*-imidazole (249).**[a]

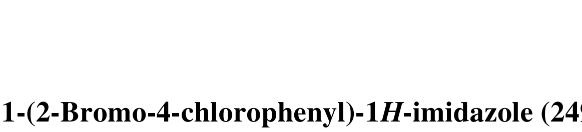

**249**

[a]Reagents and conditions: (a) 1,2-diformylhydrazine, $Me_3SiCl$, $NEt_3$, pyridine, 100 °C, 76%.

Chlorotrimethylsilane (23.05 mL, 181.62 mmol, 15 eq.) was added at RT to a solution of 2-bromo-4-chloroaniline (2.50 g, 12.11 mmol), 1,2-diformylhydrazine (3.20 g, 36.32 mmol, 3.0 eq.) and triethylamine (11.81 mL, 84.76 mmol, 7.0 eq.) in pyridine (120 mL).The reaction mixture was stirred at 100 °C overnight, cooled to RT and diluted with ethyl acetate. The mixture was washed three times with

water, dried, filtered and concentrated under reduced pressure. The residue was purified by flash silica gel chromatography (dichloromethane / methanol gradient) to give **249**. Yield: 2.40 g (76% of theory). LC/MS (method 3): $t_R$ = 1.18 min, MS (ESIpos): $m/z$ = 258 $[M+H]^+$; $^1H$ NMR (400 MHz, DMSO-$d_6$): δ [ppm] = 8.83 (s, 2H), 8.09 (d, $J$ = 2.0 Hz, 1H), 7.70 (dd, $J$ = 8.6 Hz, 2.1 Hz, 1H), 7.67 (d, $J$ = 8.4 Hz, 1H).

**1-(2-Bromo-4-chlorophenyl)-1*H*-imidazole (250).**[a]

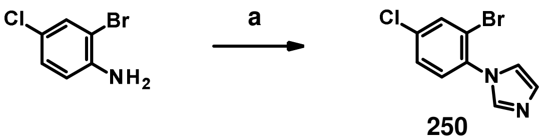


**250**

[a]Reagents and conditions: (a) i) oxalaldehyde, MeOH, ii) $H_4NCl$, HCOH, MeOH, RF, iii) phosphoric acid, RF, 28%.

Oxalaldehyde solution (40% in water, 2.20 mL, 19.37 mmol, 1.0 eq.) was added at RT to a mixture of 2-bromo-4-chloroaniline (4.00 g, 19.37 mmol) in methanol (11 mL). The reaction mixture was stirred at RT for 3 h. Methanol (88 mL), ammonium chloride (2.07 g, 38.74 mmol, 2.0 eq.) and formaldehyde solution (37% in water, 3.05 mL, 40.68 mmol, 2.1 eq.) were added. The reaction mixture was stirred under reflux for 1 h, mixed dropwise with phosphoric acid (85%, 2 mL) over a period of 10 min, stirred under reflux for 6 h and concentrated under reduced pressure. The residue was mixed with iced water and dichloromethane. With vigorous stirring, the mixture was carefully adjusted to pH 9 by addition of sodium carbonate. After phase separation, the aqueous phase was extracted with dichloromethane. The combined organic phases were dried over anhydrous sodium sulfate, filtered and concentrated under reduced pressure. The residue was purified by flash silica gel chromatography (dichloromethane / methanol gradient). The residue was stirred in a little diethyl ether for 20 min, and the solid was filtered with suction, washed with diethyl ether and dried *in vacuo* to give **250**. Yield: 1.40 g (28% of theory). LC/MS (method 8): $t_R$ = 1.58 min, MS (ESIpos): $m/z$ = 257 $[M+H]^+$; $^1H$ NMR (400 MHz, DMSO-$d_6$): δ [ppm] = 8.02 (d, $J$ = 2.2 Hz, 1H), 7.87 (s, 1H), 7.64 (dd, $J$ = 8.6 Hz, 2.2 Hz, 1H), 7.55 (d, $J$ = 8.6 Hz, 1H), 7.41 (s, 1H), 7.10 (s, 1H).

**1-(2-Bromo-4-chlorophenyl)-4-chloro-1*H*-imidazole (251).**[a]

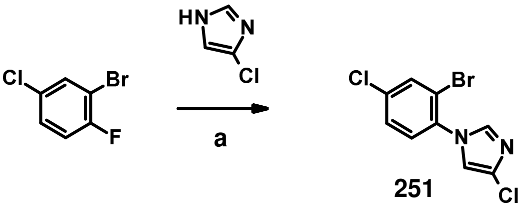


[a]Reagents and conditions: (a) $K_2CO_3$, DMF, 130 °C/microwave, 64%.

A mixture of 2-bromo-4-chloro-1-fluorobenzene (891 $\mu$L, 7.02 mmol), 4-chloro-1*H*-imidazole (720 mg, 7.02 mmol, 1.0 eq.) and potassium carbonate (2.91 g, 21.07 mmol, 3.0 eq.) in *N,N*-dimethyl formamide (30 mL) was divided into two microwave vessels and stirred in the microwave at 130 °C for 3 h. After cooling to RT, the two reaction mixtures were combined, mixed with cold water and stirred for 5 min. The suspension was filtered, and the solid was washed with ice-water and pentane and dried *in vacuo* to give **251**. Yield: 1.33 g (64% of theory). LC/MS (method 1): $t_R$ = 0.97 min, MS (ESIpos): *m*/*z* = 291 $[M+H]^+$; $^1$H NMR (400 MHz, DMSO-$d_6$): δ [ppm] = 8.05 (d, *J* = 2.2 Hz, 1H), 7.89 (d, *J* = 1.3 Hz, 1H), 7.67 (dd, *J* = 8.6 Hz, 2.2 Hz, 1H), 7.62 (d, *J* = 8.6 Hz, 1H), 7.60 (d, *J* = 1.5 Hz, 1H).

**1-(2-Bromo-4-chlorophenyl)-4-chloro-1*H*-1,2,3-triazole (254).**[a]

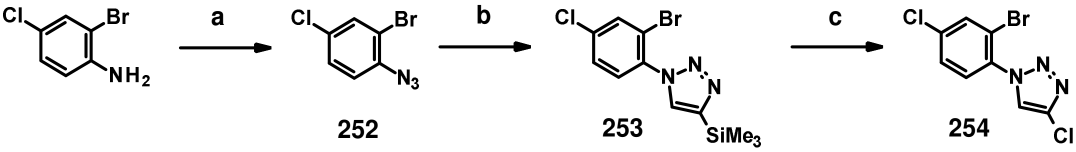


[a]Reagents and conditions: (a) *tert*-$BuNO_2$, $Me_3SiN_3$, ACN, RT, 99%; (b) ethynyl(trimethylsilyl)silane, toluene, 110 °C, 91%; (c) NCS, KF, ACN, 90 °C, 69%.

**1-Azido-2-bromo-4-chlorobenzene (252).**

*tert*-Butyl nitrite (2.75 g, 26.64 mmol, 1.1 eq.) was added dropwise under argon atmosphere at 0 °C to a solution of 2-bromo-4-chloroaniline (5.00 g, 24.22 mmol) and trimethylsilyl azide (3.35 g, 29.06 mmol, 1.2 eq.) in acetonitrile (120 mL). The reaction mixture was stirred at RT for 72 h and concentrated under reduced pressure. The residue was purified by silica gel chromatography (dichloromethane) to give **252**. Yield: 5.60 g (99% of theory). $^1$H NMR (400 MHz, DMSO-$d_6$): δ [ppm] = 7.80 (d, *J* = 2.0 Hz, 1H), 7.58-7.52 (dd, *J* = 8.7 Hz, 2.3 Hz, 1H), 7.47-7.44 (d, *J* = 8.7 Hz, 1H).

**1-(2-Bromo-4-chlorophenyl)-4-(trimethylsilyl)-1*H*-1,2,3-triazole (253).**

Ethynyl(trimethylsilyl)silane (7.61 g, 77.43 mmol) was added under argon atmosphere at RT to a solution of 1-azido-2-bromo-4-chlorobenzene (**252**) (6.00 g, 25.81 mmol) in toluene (48.0 mL). The

reaction mixture was stirred at 110 °C for 12 h, cooled to RT and concentrated under reduced pressure. The residue was purified by silica gel chromatography (petroleum ether / ethyl acetate mixture) to give **253**. Yield: 7.80 g (91% of theory). LC/MS (method 11): $t_R$ = 1.21 min, MS (ESIpos): *m*/*z* = 330 $[M+H]^+$; $^1$H NMR (400 MHz, DMSO-$d_6$): δ [ppm] = 8.55 (s, 1H), 8.08 (d, *J* = 2.0 Hz, 1H), 7.69 (dd, *J* = 8.5 Hz, 2.0 Hz, 1H), 7.65 (d, *J* = 8.4 Hz, 1H), 0.31 (s, 9H).

**1-(2-Bromo-4-chlorophenyl)-4-chloro-1*H*-1,2,3-triazole (254).**

*N*-Chlorosuccinimide (38.77 g, 290.30 mmol, 12 eq.) and potassium fluoride (8.43 g, 145.15 mmol, 6 eq.) were added under argon atmosphere at RT to a solution of 1-(2-bromo-4-chlorophenyl)-4-(trimethylsilyl)-1*H*-1,2,3-triazole (**253**) (8.00 g, 24.19 mmol) in acetonitrile (250 mL). The reaction mixture was stirred at 90 °C for 40 h, cooled to RT and filtered. The filtrate was concentrated under reduced pressure. The residue was purified by silica gel chromatography (petroleum ether / ethyl acetate mixture) to give **254**. Yield: 5.00 g (69% of theory). LC/MS (method 12): $t_R$ = 1.55 min, MS (ESIpos): *m*/*z* = 292 $[M+H]^+$; $^1$H NMR (400 MHz, DMSO-$d_6$): δ [ppm] = 8.88 (s, 1H), 8.13 (d, *J* = 2.0 Hz, 1H), 7.76 (d, *J* = 8.1 Hz, 1H), 7.73 (dd, *J* = 8.6 Hz, 2.0 Hz, 1H).

**1-(2-Bromo-4-chlorophenyl)-4-(difluoromethyl)-1*H*-1,2,3-triazole (257).**[a]

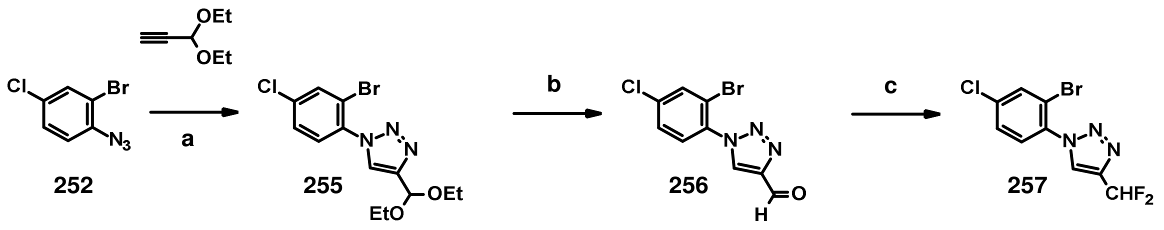


[a]Reagents and conditions: (a) toluene, 110 °C, 78%; (b) AcOH, water, RT, 91%; (c) DAST, DCM, RT, 68%.

**1-(2-Bromo-4-chlorophenyl)-4-(diethoxymethyl)-1*H*-1,2,3-triazole (255).**

3,3-Diethoxyprop-1-yne (4.96 g, 38.71 mmol, 1.5 eq.) was added under argon atmosphere at RT to a solution of 1-azido-2-bromo-4-chlorobenzene (**252**) (6.00 g, 25.81 mmol) in toluene (60 mL). The reaction mixture was stirred at 110 °C for 15 h, cooled to RT and concentrated under reduced pressure. The residue was purified by silica gel chromatography (petroleum ether / ethyl acetate mixture) to give **255**. Yield: 8.10 g (90% purity, 78% of theory). LC/MS (method 11): $t_R$ = 1.12 min, MS (ESIpos): *m*/*z* = 360 $[M+H]^+$; $^1$H NMR (300 MHz, DMSO-$d_6$): δ [ppm] = 8.49 (s, 1H), 8.11 (s, 1H), 7.72-7.70 (m, 2H), 5.78 (s, 1H), 3.67-3.57 (m, 4H), 1.17 (t, *J* = 7.1 Hz, 6H).

**1-(2-Bromo-4-chlorophenyl)-1*H*-1,2,3-triazole-4-carbaldehyde (256).**

1-(2-Bromo-4-chlorophenyl)-4-(diethoxymethyl)-1*H*-1,2,3-triazole (**255**) (2.00 g, 5.55 mmol) was added at RT to a mixture of acetic acid (13.00 mL, 221.83 mmol) in water (60 mL). The reaction mixture

was stirred at RT overnight and diluted with water. The aqueous phase was extracted with dichloromethane. The combined organic phases were washed with water and brine, dried over anhydrous sodium sulfate, filtered, concentrated under reduced pressure and dried *in vacuo* to give **256** which was used without further purification. Yield: 1.50 g (91% of theory). LC/MS (method 11): $t_R$ = 0.98 min, MS (ESIpos): *m/z* = 286 $[M+H]^+$; $^1H$ NMR (400 MHz, DMSO-$d_6$): δ [ppm] = 10.12 (s, 1H), 9.35 (s, 1H), 8.14 (s, 1H), 7.80-7.74 (m, 2H).

**1-(2-Bromo-4-chlorophenyl)-4-(difluoromethyl)-1*H*-1,2,3-triazole (257).**

Diethylaminosulfur trifluoride (3.15 g, 19.55 mmol) was added at RT to a solution of 1-(2-bromo-4-chlorophenyl)-1*H*-1,2,3-triazole-4-carbaldehyde (**256**) (2.80 g, 9.77 mmol) in dichloromethane (60 mL). The reaction mixture was stirred at RT for 2 h and added to an ice-cooled, saturated aqueous sodium bicarbonate solution. After phase separation, the aqueous phase was extracted with dichloromethane. The combined organic phases were washed with water and brine, dried over anhydrous sodium sulfate, filtered and concentrated under reduced pressure. The residue was purified by silica gel chromatography (petroleum ether / ethyl acetate mixture) to give **257**. Yield: 2.07 g (68% of theory). LC/MS (method 12): $t_R$ = 1.49 min, MS (ESIpos): *m/z* = 308 $[M+H]^+$; $^1H$ NMR (400 MHz, DMSO-$d_6$): δ [ppm] = 9.01 (s, 1H), 8.12 (s, 1H), 7.80-7.72 (m, 2H), 7.34 (t, *J* = 54.0 Hz, 1H); $^{19}F$ NMR (376 MHz, DMSO-$d_6$): δ [ppm] = -112.23 (s, 2F).

**1-(2-Bromo-4-chlorophenyl)-4-(trifluoromethyl)-1*H*-1,2,3-triazole (91).**[a]

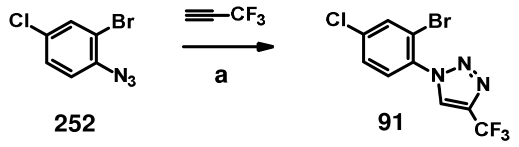


[a]Reagents and conditions: (a) $Cu_2O$, ACN, RT, 71%.

In a 3-neck flask (equipped with an empty balloon to catch excess gas and avoid pressure build up, however, it remained empty during the reaction), copper(I) oxide (690 mg, 4.8 mmol) was added at RT to a solution of 1-azido-2-bromo-4-chlorobenzene (**252**) (10.4 g, 44.7 mmol) in acetonitrile (600 mL). Trifluoropropyne (5 g cylinder) was bubbled gently through the solution at RT for 10-15 min until the cylinder was empty. After capping the flask and stirring for 3 d, approximately 80% conversion to desired product was observed. Further trifluoropropyne (1 g from a second 5 g cylinder) was bubbled gently through the solution. The solution was stirred overnight and concentrated under reduced pressure. The residue was taken up in a mixture of *n*-heptane / dichloromethane (1:1) and filtered through a plug of silica gel. The residue was crystallized from *n*-heptane to give a first batch of **91** (9.5 g). Precipitation from the mother liquor gave a second batch of **91** (0.9 g). The batches were combined. Yield: 10.4 g (71% of theory). LC/MS (method 3): $t_R$ = 2.04 min, MS (ESIpos): *m/z* = 326 $[M+H]^+$; $^1H$ NMR

(400 MHz, DMSO-$d_6$): δ [ppm] = 9.42 (s, 1H), 8.17 (d, $J$ = 2.2 Hz, 1H), 7.85 (d, $J$ = 8.6 Hz, 1H), 7.78 (dd, $J$ = 8.6 Hz, 2.2 Hz, 1H).

**2-(2-Bromo-4-chlorophenyl)-5-(difluoromethyl)-1,3,4-oxadiazole (260).**[a]

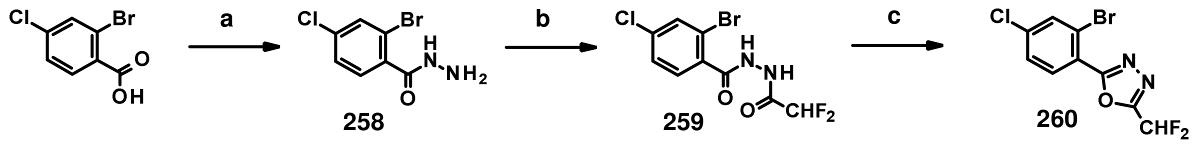

[a]Reagents and conditions: (a) i) CDI, DMAP, THF, 70 °C, ii) $N_2H_4$, RT, 82%; (b) difluoroacetic anhydride, $NEt_3$, DCM, RT, 36%; (c) $SOCl_2$, 50 °C, 71%.

**2-Bromo-4-chlorobenzohydrazide (258).**

1,1´-Carbonyldiimidazole (1.50 g, 9.27 mmol, 1.5 eq.) and 4-dimethylaminopyridine (0.38 g, 3.09 mmol, 0.5 eq.) were added under argon atmosphere at RT to a mixture of 2-bromo-4-chlorobenzoic acid (1.50 g, 6.18 mmol) in tetrahydrofuran (58 mL). The reaction mixture was stirred at 70 °C for 3 h and cooled to RT, followed by addition of hydrazine solution (1 M in tetrahydrofuran, 8.03 mL, 8.03 mmol, 1.3 eq.) in one portion. The reaction mixture was stirred at RT for 75 min, mixed with additional hydrazine solution (1 M in tetrahydrofuran, 8.03 mL, 8.03 mmol, 1.3 eq.), stirred for further 30 min and mixed with dichloromethane (60 mL) and saturated aqueous sodium bicarbonate solution (60 mL). The aqueous phase was extracted with dichloromethane. The combined organic phases were washed with water, dried over anhydrous magnesium sulfate, filtered and concentrated under reduced pressure. The residue was purified by flash silica gel chromatography (dichloromethane / methanol gradient) to give **258**. Yield: 1.30 g (82% of theory). LC/MS (method 8): $t_R$ = 1.11 min, MS (ESIpos): $m/z$ = 249 $[M+H]^+$; $^1H$ NMR (400 MHz, DMSO-$d_6$): δ [ppm] = 9.58 (br s, 1H), 7.81 (d, $J$ = 2.0 Hz, 1H), 7.52 (dd, $J$ = 8.2 Hz, 2.1 Hz, 1H), 7.37 (d, $J$ = 8.2 Hz, 1H), 4.49 (br s, 2H).

**2-Bromo-4-chloro-*N*'-(difluoroacetyl)benzohydrazide (259).**

Difluoroacetic anhydride (9.98 g, 57.31 mmol, 1.3 eq.) and triethylamine (10.45 mL, 74.95 mmol, 1.7 eq.) were added dropwise at 0 °C to a solution of 2-bromo-4-chlorobenzohydrazide (**258**) (11.00 g, 44.09 mmol) in dichloromethane (400 mL). The reaction mixture was stirred at RT for 22 h and diluted with dichloromethane. The organic phase was washed with saturated aqueous sodium bicarbonate solution and with brine, dried over anhydrous sodium sulfate, filtered and concentrated under reduced pressure to give **259** which was used without further purification. Yield: 5.20 g (36% of theory). LC/MS (method 14): $t_R$ = 0.81 min, MS (ESIpos): $m/z$ = 327 $[M+H]^+$; $^1H$ NMR (400 MHz, DMSO-$d_6$): δ [ppm] = 11.17 (br s, 1H), 10.69 (br s, 1H), 7.89 (d, $J$ = 2.0 Hz, 1H), 7.59 (dd, $J$ = 8.2 Hz, 2.0 Hz, 1H), 7.48 (d, $J$ = 8.2 Hz, 1H), 6.42 (t, $J$ = 52.9 Hz, 1H).

**2-(2-Bromo-4-chlorophenyl)-5-(difluoromethyl)-1,3,4-oxadiazole (260).**

A solution of 2-bromo-4-chloro-*N'*-(difluoroacetyl)benzohydrazide (**259**) (2.60 g, 7.90 mmol) in thionyl chloride (75 mL) was stirred at 50 °C for 12 h, cooled to RT and concentrated under reduced pressure. The residue was taken up in ethyl acetate. The organic phase was washed with saturated aqueous sodium bicarbonate solution, with water and with brine. The organic phase was dried over anhydrous sodium sulfate, filtered and concentrated under reduced pressure. The residue was purified by silica gel chromatography (petroleum ether / ethyl acetate mixture) to give **260**. Yield: 1.80 g (71% of theory). LC/MS (method 15): $t_R$ = 1.60 min, MS (ESIpos): *m*/*z* = 309 $[M+H]^+$; $^1H$ NMR (400 MHz, DMSO-$d_6$): δ [ppm] = 8.12 (d, *J* = 2.0 Hz, 1H), 7.98 (d, *J* = 8.4 Hz, 1H), 7.73 (dd, *J* = 8.5 Hz, 2.0 Hz, 1H), 7.60 (t, *J* = 51.2 Hz, 1H); $^{19}F$ NMR (376 MHz, DMSO-$d_6$): δ [ppm] = -120.83 (s, 2F).

**2-(2-Bromo-4-chlorophenyl)-5-(difluoromethyl)-1,3,4-thiadiazole (261).**[a]

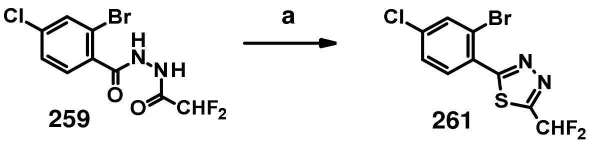


[a]Reagents and conditions: (a) phosphorus pentasulfide, toluene, 130 °C, 49%.

A mixture of 2-bromo-4-chloro-*N'*-(difluoroacetyl)benzohydrazide (**259**) (2.60 g, 7.94 mmol) and phosphorus pentasulfide (3.53 g, 15.88 mmol, 2.0 eq.) in toluene (100 mL) was heated at 130 °C for 2 h, cooled to RT and concentrated under reduced pressure. After addition of ethyl acetate / water and phase separation, the organic phase was washed with aqueous sodium hypochlorite solution (0.78 mM, 100 mL), with water and with brine, dried over anhydrous sodium sulfate, filtered and concentrated under reduced pressure. The residue was purified by flash silica gel chromatography (petroleum ether / ethyl acetate mixture) to give **261**. Yield: 1.28 g (49% of theory). LC/MS (method 13): $t_R$ = 1.80 min, MS (ESIpos): *m*/*z* = 325 $[M+H]^+$; $^1H$ NMR (400 MHz, DMSO-$d_6$): δ [ppm] = 8.13-8.10 (m, 2H), 7.71 (dd, *J* = 8.4 Hz, 2.1 Hz, 1H), 7.69 (t, *J* = 53.1 Hz, 1H); $^{19}F$ NMR (376 MHz, DMSO-$d_6$): δ [ppm] = -109.50 (s, 2F).

**3-(2-Bromo-4-chlorophenyl)-5-(difluoromethyl)-1,2-oxazole (265).**[a]

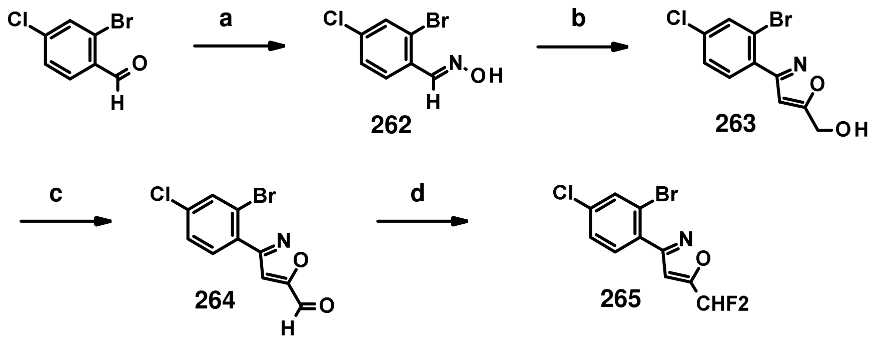


[a]Reagents and conditions: (a) NaOAc, $HONH_2$ × HCl, MeOH, RT, 72%; (b) prop-2-yn-1-ol, aq. NaClO, DCM, RT, 74%; (c) Dess-Martin periodinane, DCM, RT, 99%; (d) DAST, DCM, RT, 93%.

**1-(2-Bromo-4-chlorophenyl)-*N*-hydroxymethanimine (E/Z mixture 262).**

Sodium acetate (5.38 g, 65.61 mmol, 1.8 eq.) was added under argon atmosphere at RT to a solution of 2-bromo-4-chlorobenzaldehyde (8.00 g, 36.45 mmol) in methanol (80 mL), followed by addition of hydroxylamine hydrochloride (2.79 g, 40.10 mmol) in portions. The reaction mixture was stirred at RT for 2 h and concentrated under reduced pressure. The residue was taken up in dichloromethane. The organic phase was washed with water and brine, dried over anhydrous sodium sulfate, filtered and concentrated under reduced pressure to give **262** which was used without further purification. Yield: 6.50 g (72% of theory). LC/MS (method 16): $t_R$ = 0.92 min, MS (ESIpos): $m/z$ = 234 $[M+H]^+$; $^1$H NMR (400 MHz, DMSO-$d_6$): δ [ppm] = 11.74 (s, 1H), 8.29 (s, 1H), 7.78-7.72 (m, 2H), 7.50-7.43 (m, 1H).

**[3-(2-Bromo-4-chlorophenyl)-1,2-oxazol-5-yl]methanol (263).**

Aqueous sodium hypochlorite solution (8% strength, 17.86 g, 19.19 mmol, 1.5 eq.) and prop-2-yn-1-ol (1.44 g, 25.59 mmol, 2.0 eq.) were added at RT to a solution of 1-(2-bromo-4-chlorophenyl)-*N*-hydroxymethanimine (E/Z mixture **262**) (3.00 g, 12.79 mmol) in dichloromethane (60 mL). The reaction mixture was stirred at RT for 15 h and diluted with dichloromethane. The mixture was washed with water and brine, dried over anhydrous sodium sulfate, filtered and concentrated under reduced pressure. The residue was purified by silica gel chromatography (petroleum ether / ethyl acetate mixture) to give **263**. Yield: 2.74 g (74% of theory). LC/MS (method 17): $t_R$ = 1.05 min, MS (ESIpos): $m/z$ = 288 $[M+H]^+$; $^1$H NMR (400 MHz, DMSO-$d_6$): δ [ppm] = 7.95 (s, 1H), 7.63-7.57 (m, 2H), 6.75 (s, 1H), 5.74 (t, $J$ = 6.0 Hz, 1H), 4.63 (d, $J$ = 6.0 Hz, 2H).

**3-(2-Bromo-4-chlorophenyl)-1,2-oxazole-5-carbaldehyde (264).**

A solution of Dess-Martin periodinane (2.29 g, 5.41 mmol, 1.3 eq.) in dichloromethane (15 mL) was added dropwise at 0 °C to a solution of [3-(2-bromo-4-chlorophenyl)-1,2-oxazol-5-yl]methanol (**263**) (1.20 g, 4.20 mmol) in dichloromethane (15 mL). The reaction mixture was stirred at RT for 2 h and diluted with dichloromethane. The organic phase was washed with a mixture of aqueous saturated

sodium thiosulphate solution and aqueous saturated sodium bicarbonate solution (1:1), with water and with brine, dried over anhydrous sodium sulfate, filtered and concentrated under reduced pressure to give **264** which was used without further purification. Yield: 1.20 g (99% of theory). $^1$H NMR (400 MHz, DMSO-$d_6$): δ [ppm] = 9.97 (s, 1H), 8.02 (d, $J$ = 2.0 Hz, 1H), 7.78 (s, 1H), 7.70 (d, $J$ = 8.3 Hz, 1H), 7.66 (dd, $J$ = 8.3 Hz, 2.0 Hz, 1H).

**3-(2-Bromo-4-chlorophenyl)-5-(difluoromethyl)-1,2-oxazole (265).**

Diethylaminosulfur trifluoride (1.35 g, 8.38 mmol, 2.0 eq.) was added at RT to a solution of 3-(2-bromo-4-chlorophenyl)-1,2-oxazole-5-carbaldehyde (**264**) (1.20 g, 4.20 mmol) in dichloromethane (24 mL). The reaction mixture was stirred at RT for 15 h and diluted with dichloromethane. The organic phase was washed with water and brine, dried over anhydrous sodium sulfate, filtered and concentrated under reduced pressure. The crude product was purified by silica gel chromatography (petroleum ether / ethyl acetate mixture) to give **265**. Yield: 1.21 g (93% of theory). LC/MS (method 13): $t_R$ = 1.78 min, MS (ESIpos): $m/z$ = 308 $[M+H]^+$; $^1$H NMR (400 MHz, DMSO-$d_6$): δ [ppm] = 8.00 (d, $J$ = 1.6 Hz, 1H), 7.68 (d, $J$ = 8.2 Hz, 1H), 7.64 (dd, $J$ = 8.3 Hz, 2.0 Hz, 1H), 7.43 (t, $J$ = 52.6 Hz, 1H), 7.42-7.40 (m, 1H); $^{19}$F NMR (376 MHz, DMSO-$d_6$): δ [ppm] = -118.03 (s, 2F).

**5-(2-Bromo-4-chlorophenyl)-2-(difluoromethyl)-1,3-oxazole (268).**[a]

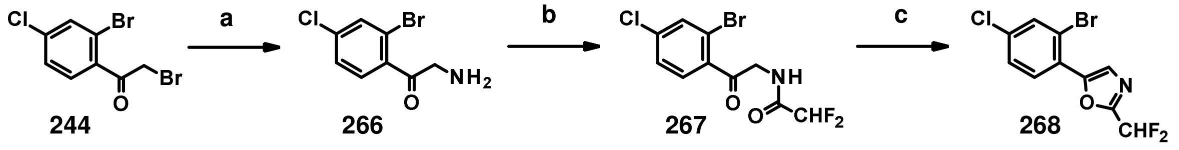


[a]Reagents and conditions: (a) i) hexamethylenetetramine, $CHCl_3$, RT ii) conc. HCl, MeOH, RF; (b) difluoroacetic anhydride, $NEt_3$, DCM, RT, 41%; (c) phosphorus pentoxide, $CHCl_3$, 60 °C, 42%.

**2-Amino-1-(2-bromo-4-chlorophenyl)ethanone hydrochloride (266).**

Hexamethylenetetramine (2.29 g, 16.33 mmol, 1.02 eq.) was added at RT to a solution of 2-bromo-1-(2-bromo-4-chlorophenyl)ethanone (**244**) (5.00 g, 16.00 mmol) in chloroform (50 mL). After stirring at RT for 4 h, the solid was collected by filtration, washed with water and dried *in vacuo*. The residue was dissolved in methanol (50 mL), mixed with concentrated hydrochloric acid (20 mL), refluxed for 3 h, cooled to RT and evaporated under reduced pressure to give **266** which was used without further purification. Yield: 7.00 g. LC/MS (method 11): $t_R$ = 0.71 min, MS (ESIpos): $m/z$ = 248 $[M+H]^+$; $^1$H NMR (400 MHz, DMSO-$d_6$): δ [ppm] = 8.43 (br s, 3H), 7.97-7.94 (m, 2H), 7.70-7.67 (m, 1H), 4.49 (s, 2H).

**$N$-[2-(2-Bromo-4-chlorophenyl)-2-oxoethyl]-2,2-difluoroacetamide (267).**

Difluoroacetic anhydride (4.76 g, 27.37 mmol, 1.3 eq.) and triethylamine (4.99 mL, 35.79 mmol, 1.7 eq.) were added at 0 °C to a solution of 2-amino-1-(2-bromo-4-chlorophenyl)ethanone hydrochloride (**266**) (6.00 g, 21.06 mmol) in dichloromethane (200 mL). After stirring at RT for 22 h, the reaction mixture was diluted with dichloromethane, washed with saturated sodium bicarbonate solution and brine, dried over anhydrous sodium sulfate, filtered and concentrated under reduced pressure. The residue was purified by silica gel chromatography (petroleum ether / ethyl acetate mixture) to give **267**. Yield: 3.00 g (41% of theory). LC/MS (method 11): $t_R$ = 1.02 min, MS (ESIpos): $m/z$ = 326 $[M+H]^+$; $^1H$ NMR (400 MHz, DMSO-$d_6$): δ [ppm] = 9.29 (br t, $J$ = 5.4 Hz, 1H), 7.92 (d, $J$ = 2.0 Hz, 1H), 7.76 (d, $J$ = 8.3 Hz, 1H), 7.62 (dd, $J$ = 8.3 Hz, 2.0 Hz, 1H), 6.34 (t, $J$ = 53.4 Hz, 1H), 4.52 (d, $J$ = 5.6 Hz, 2H); $^{19}F$ NMR (376 MHz, DMSO-$d_6$): δ [ppm] = -126.09 (s, 2F).

**5-(2-Bromo-4-chlorophenyl)-2-(difluoromethyl)-1,3-oxazole (268).**

Phosphorus pentoxide (3.91 g, 27.56 mmol, 3.0 eq.) was added at RT to a solution of $N$-[2-(2-bromo-4-chlorophenyl)-2-oxoethyl]-2,2-difluoroacetamide (**267**) (3.00 g, 9.19 mmol) in chloroform (300 mL). The reaction mixture was heated at 60 °C for 24 h, cooled to RT, diluted with water and extracted with ethyl acetate. The aqueous phase was adjusted to pH 7 with sodium carbonate and extracted with ethyl acetate. The combined organic phases were washed with brine, dried over anhydrous sodium sulfate, filtered and concentrated under reduced pressure. The residue was purified by silica gel chromatography (petroleum ether / ethyl acetate mixture) to give **268**. Yield: 1.21 g (42% of theory). LC/MS (method 13): $t_R$ = 1.77 min, MS (ESIpos): $m/z$ = 308 $[M+H]^+$; $^1H$ NMR (400 MHz, DMSO-$d_6$): δ [ppm] = 8.00-7.98 (m, 2H), 7.79 (d, $J$ = 8.4 Hz, 1H), 7.65 (dd, $J$ = 8.6 Hz, 2.1 Hz, 1H), 7.31 (t, $J$ = 51.9 Hz, 1H); $^{19}F$ NMR (376 MHz, DMSO-$d_6$): δ [ppm] = -118.87 (s, 2F).

**5-(2-Bromo-4-chlorophenyl)-1-{[2-(trimethylsilyl)ethoxy]methyl}-1$H$-pyrazole (271).**[a]

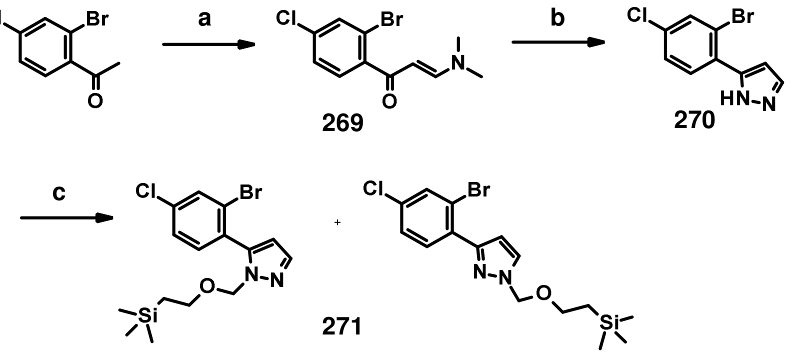


[a]Reagents and conditions: (a) 1,1-dimethoxy-$N,N$-dimethylmethanamine, DMF, 120 °C, 89%; (b) $N_2H_4 \times H_2O$, MeOH/water, 80 °C, 96%; (c) i) NaH, THF, 0 °C, ii) SEM-Cl, RT, 87%.

**1-(2-Bromo-4-chlorophenyl)-3-(dimethylamino) prop-2-en-1-one (269).**

A solution of 1-(2-bromo-4-chlorophenyl)ethanone (2.00 g, 8.56 mmol) in *N,N*-dimethylformamide (20 mL) was treated with 1,1-dimethoxy-*N,N*-dimethylmethanamine (3.06 g, 25.70 mmol, 3.0 eq.) and heated at 120 °C for 24 h. After cooling to RT, the reaction mixture was evaporated under reduced pressure to give **269** which was used without further purification. Yield: 2.40 g (92% purity, 89% of theory). LC/MS (method 18): $t_R$ = 0.96 min, MS (ESIpos): *m*/*z* = 288 $[M+H]^+$.

**5-(2-Bromo-4-chlorophenyl)-1*H*-pyrazole (270).**

Hydrazine hydrate (1.91 g, 30.54 mmol, 4.0 eq.) and water (12.5 mL) were added at RT to a solution of 1-(2-bromo-4-chlorophenyl)-3-(dimethylamino)prop-2-en-1-one (**269**) (2.40 g, 7.63 mmol) in methanol (50 mL). The reaction mixture was stirred at 80 °C for 2 h, cooled to RT and concentrated under reduced pressure. The residue was dissolved in dichloromethane, washed with saturated sodium carbonate solution, with water and with brine. The organic phase was dried over anhydrous sodium sulfate, filtered and evaporated under reduced pressure to give **270**. Yield: 1.92 (96% of theory). LC/MS (method 18): $t_R$ = 1.08 min, MS (ESIpos): *m*/*z* = 257 $[M+H]^+$; $^1$H NMR (400 MHz, DMSO-$d_6$): δ [ppm] = 13.10-13.20 (m, 1H), 7.81-7.89 (m, 2H), 7.70 (d, *J* = 8.3 Hz, 1H), 7.48-7.56 (m, 1H), 6.60-6.72 (m, 1H).

**Mixture of 5-(2-bromo-4-chlorophenyl)-1-{[2-(trimethylsilyl)ethoxy]methyl}-1*H*-pyrazole and 3-(2-bromo-4-chlorophenyl)-1-{[2-(trimethylsilyl)ethoxy]methyl}-1*H*-pyrazole (271).**

A solution of 5-(2-bromo-4-chlorophenyl)-1*H*-pyrazole (**270**) (309 mg, 1.20 mmol) in tetrahydrofuran (10 mL) was added dropwise under argon atmosphere at 0 °C to a mixture of sodium hydride (60% in mineral oil, 173 mg, 4.32 mmol, 3.6 eq.) in tetrahydrofuran (10 mL). The reaction mixture was allowed to warm to RT, mixed with a solution of [2-(chloromethoxy)ethyl](trimethyl)silane (284 μL, 1.44 mmol, 1.2 eq.) in tetrahydrofuran (10 mL), stirred until completeness of the reaction and evaporated under reduced pressure. The residue was purified by flash silica gel chromatography (cyclohexane / ethyl acetate gradient) to give **271** as regioisomeric mixture (approx. 1:1). Yield: 403 mg (87% of theory). LC/MS (method 1): $t_R$ = 1.41 min / 1.45 min, MS (ESIpos): *m*/*z* = 387 $[M+H]^+$; $^1$H NMR (400 MHz, DMSO-$d_6$): δ [ppm] = 7.99 / 7.95 / 7.85 / 7.64-7.60 / 7.59 / 7.55 / 7.52 (3x "d" / m / 3x "d", *J* = 2.5 / 2.0 / 2.1 / 2.0 / 2.0 / 2.1 Hz, 3H), 7.67 / 7.50 (2x d, *J* = 8.3 / 8.3 Hz, 1H), 6.76 / 6.46 (2x d, *J* = 2.3 / 1.5 Hz, 1H), 5.46 / 5.25 (2x s, 2H), 3.59 / 3.37 (2x t, *J* = 8.0 / 8.3 Hz, 2H), 0.84 / 0.72 (2x t, *J* = 8.0 / 8.3 Hz, 2H), -0.05 / -0.09 (2x s, 9H).

**Synthesis of racemate 49.**[a]

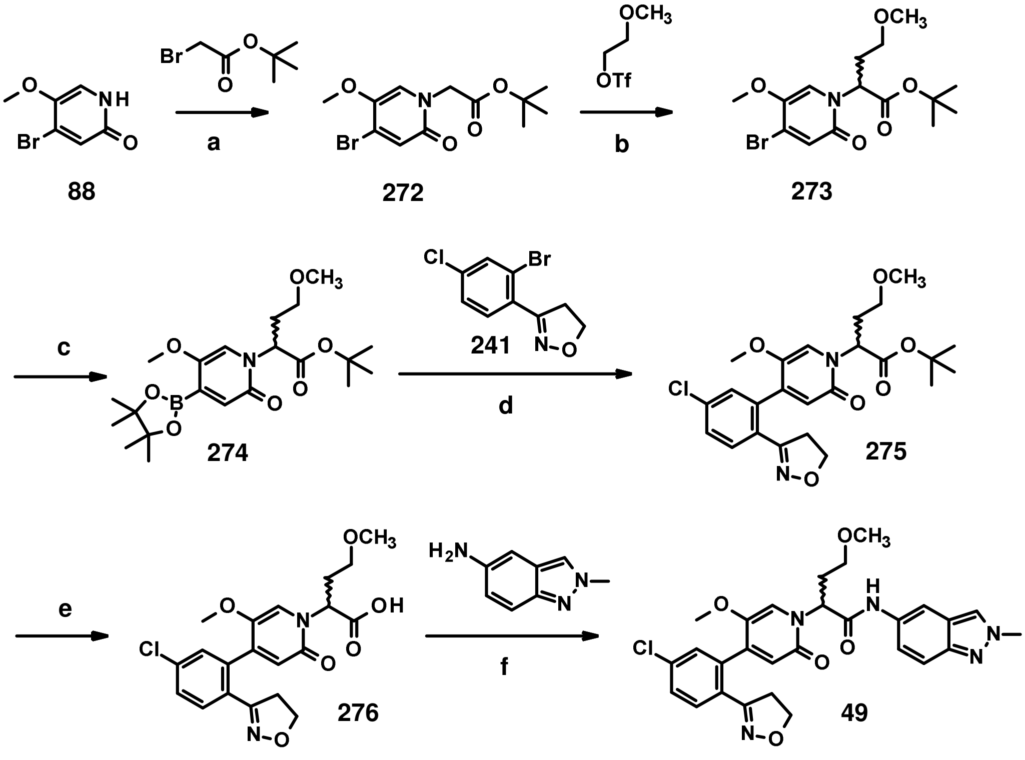

[a]Reagents and conditions: (a) K2CO3, DMF, 50 °C, 80%; (b) LiHMDS, THF, -70 °C → RT, 72%; (c) $(BPin)_2$, Pd(dppf)$Cl_2$-DCM complex, KOAc, 1,4-dioxane, 80 °C, 100%; (d) Pd(dppf)$Cl_2$-DCM complex, $K_2CO_3$, 1,4-dioxane, 80 °C, 58%; (e) HCl/1,4-dioxane, RT, 94%; (f) T3P, pyridine, 50 °C, 83%.

***tert*-Butyl (4-bromo-5-methoxy-2-oxopyridin-1(2*H*)-yl)acetate (272).**

*tert*-Butyl bromoacetate (10.63 mL, 70.58 mmol, 1.2 eq.) was added under argon atmosphere at RT to a mixture of 4-bromo-5-methoxypyridin-2(1*H*)-one (**88**) (12.00 g, 58.82 mmol) and potassium carbonate (12.19 g, 88.22 mmol, 1.5 eq.) in *N,N*-dimethylformamide (267 mL). The mixture was stirred at 50 °C for 80 min and concentrated under reduced pressure. The residue was mixed with water (120 mL), stirred for 5 min and filtered with suction. The solid was washed with water, suspended in acetonitrile and concentrated under reduced pressure. The residue was purified by flash silica gel chromatography (dichloromethane / methanol gradient) to give **272**. Yield: 15.00 g (80% of theory). LC/MS (method 3): $t_R$ = 1.49 min, MS (ESIpos): $m/z$ = 318 $[M+H]^+$; $^1H$ NMR (400 MHz, DMSO-$d_6$): δ [ppm] = 7.53 (s, 1H), 6.85 (s, 1H), 4.53 (s, 2H), 3.69 (s, 3H), 1.42 (s, 9H).

***tert*-Butyl 2-(4-bromo-5-methoxy-2-oxopyridin-1(2*H*)-yl)-4-methoxybutanoate (racemate 273).**

Bis(trimethylsilyl)lithium amide solution (1.0 M in tetrahydrofuran, 14.66 mL, 1.35 eq.) was added dropwise under argon atmosphere at -70 °C to a solution of *tert*-butyl (4-bromo-5-methoxy-2-oxopyridin-1(2*H*)-yl)acetate (**272**) (3.60 g, 10.86 mmol) in tetrahydrofuran (138 mL). The reaction

mixture was stirred at -70 °C for 20 min, mixed dropwise with 2-methoxyethyl trifluoromethanesulfonate (1.93 mL, 12.49 mmol, 1.15 eq.), stirred at -70 °C for 15 min and at RT for 1.5 h. The reaction mixture was again cooled to -70 °C, mixed dropwise with additional bis(trimethylsilyl)lithium amide solution (1.0 M in tetrahydrofuran, 4.9 mL, 0.45 eq.), followed after 15 min by addition of 2-methoxyethyl trifluoromethanesulfonate (0.65 mL, 4.23 mmol, 0.39 eq.). The mixture was stirred at -70 °C for 15 min and at RT for 3 h, followed by addition of first saturated aqueous ammonium chloride solution (40 mL) and then water and ethyl acetate. After phase separation, the organic phase was washed with brine, dried over anhydrous sodium sulfate, filtered and concentrated under reduced pressure. The residue was purified by flash silica gel chromatography (cyclohexane / ethyl acetate gradient) to give **273**. Yield: 3.09 g (95% purity, 72% of theory). LC/MS (method 1): $t_R$ = 0.94 min, MS (ESIpos): *m*/*z* = 376 $[M+H]^+$; $^1$H NMR (400 MHz, DMSO-$d_6$): δ [ppm] = 7.36 (s, 1H), 6.85 (s, 1H), 5.04 (dd, *J* = 8.8 Hz, 6.0 Hz, 1H), 3.71 (s, 3H), 3.39-3.29 (m, 1H), 3.20-3.03 (m, 1H), 2.35-2.20 (m, 2H), 1.38 (s, 9H).

***tert*-Butyl 4-methoxy-2-[5-methoxy-2-oxo-4-(4,4,5,5-tetramethyl-1,3,2-dioxaborolan-2-yl)pyridin-1(2*H*)-yl]butanoate (racemate 274).**

[1,1-Bis(diphenylphosphino)ferrocene]dichloropalladium-dichloromethane complex (379 mg, 0.46 mmol, 0.03 eq.) was added under argon atmosphere at RT to a mixture of *tert*-butyl 2-(4-bromo-5-methoxy-2-oxopyridin-1(2*H*)-yl)-4-methoxybutanoate (racemate **273**) (6.00 g, 15.47 mmol), bis(pinacolato)diboron (4.32 g, 17.02 mmol, 1.1 eq.) and potassium acetate (4.55 g, 46.41 mmol, 3.0 eq.) in 1,4-dioxane (161 mL). The reaction mixture was stirred at 80 °C for 5 h, cooled to RT and filtered through kieselguhr, and the filter residue was washed with 1,4-dioxane. The combined filtrates were concentrated under reduced pressure. The residue was dried at 40 °C under high vacuum to give **274**. Yield: 9.90 g (66% purity, 100% of theory). $^1$H NMR (400 MHz, DMSO-$d_6$): δ [ppm] = 7.09 (s, 1H), 6.49 (s, 1H), 5.00 (dd, *J* = 9.4 Hz, 5.7 Hz, 1H), 3.60 (s, 3H), 3.36-3.27 (m, 1H), 3.17 (s, 3H), 3.14-3.05 (m, 1H), 2.30-2.21 (m, 2H), 1.37 (s, 9H), 1.27 (s, 12H).

***tert*-Butyl 2-{4-[5-chloro-2-(4,5-dihydro-1,2-oxazol-3-yl)phenyl]-5-methoxy-2-oxopyridin-1(2*H*)-yl}-4-methoxybutanoate (racemate 275).**

A mixture of *tert*-butyl 4-methoxy-2-[5-methoxy-2-oxo-4-(4,4,5,5-tetramethyl-1,3,2-dioxaborolan-2-yl)pyridin-1(2*H*)-yl]butanoate (racemate **274**) (343 mg, 70% purity, 0.57 mmol, 1.0 eq.), 3-(2-bromo-4-chlorophenyl)-4,5-dihydro-1,2-oxazole (**241**) (150 mg, 0.57 mmol) and potassium carbonate (235 mg, 1.70 mmol, 3.0 eq.) in 1,4-dioxane (5.5 mL) was flushed with argon at RT for 5 min, followed by addition of [1,1'-bis(diphenylphosphino)ferrocene]dichloropalladium-dichloromethane complex (14 mg, 0.02 mmol, 0.03 eq.). The reaction mixture was stirred at 80 °C overnight, cooled to RT, filtered through kieselguhr, and the filter residue was washed with dichloromethane. The combined filtrates were concentrated under reduced pressure. The residue was purified by flash silica gel chromatography

(cyclohexane / ethyl acetate gradient) to give **275**. Yield: 166 mg (95% purity, 58% of theory). LC/MS (method 1): $t_R$ = 1.02 min, MS (ESIpos): *m*/*z* = 477 $[M+H]^+$; $^1$H NMR (400 MHz, DMSO-$d_6$): δ [ppm] = 7.64 (d, *J* = 8.4 Hz, 1H), 7.58 (dd, *J* = 8.4 Hz, 2.2 Hz, 1H), 7.43 (d, *J* = 2.2 Hz, 1H), 7.16 (s, 1H), 6.32 (s, 1H), 5.09-4.98 (m, 1H), 4.33-4.16 (m, 2H), 3.53 (s, 3H), 3.39-3.08 (m, 4H), 3.20 (s, 3H), 2.36-2.18 (m, 2H), 1.40 (s, 9H).

**2-{4-[5-Chloro-2-(4,5-dihydro-1,2-oxazol-3-yl)phenyl]-5-methoxy-2-oxopyridin-1(2*H*)-yl}-4-methoxybutanoic acid hydrochloride (racemate 276).**

A mixture of *tert*-butyl 2-{4-[5-chloro-2-(4,5-dihydro-1,2-oxazol-3-yl)phenyl]-5-methoxy-2-oxopyridin-1(2*H*)-yl}-4-methoxybutanoate (racemate **275**) (149 mg, 95% purity, 0.30 mmol) in hydrogen chloride solution (4 M in 1,4-dioxane, 3.0 mL) was stirred at RT overnight and concentrated under reduced pressure to give **276** which was used without further purification. Yield: 134 mg (95% purity, 94% of theory). LC/MS (method 3): $t_R$ = 1.33 min, MS (ESIpos): *m*/*z* = 421 $[M+H]^+$; $^1$H NMR (400 MHz, DMSO-$d_6$): δ [ppm] = 7.64 (d, *J* = 8.4 Hz, 1H), 7.58 (dd, *J* = 8.3 Hz, 2.2 Hz, 1H), 7.45 (d, *J* = 2.1 Hz, 1H), 7.20 (s, 1H), 6.31 (s, 1H), 5.21-4.92 (m, 1H), 4.33-4.20 (m, 2H), 3.53 (s, 3H), 3.40-3.04 (m, 7H), 2.38-2.25 (m, 2H).

**2-{4-[5-Chloro-2-(4,5-dihydro-1,2-oxazol-3-yl)phenyl]-5-methoxy-2-oxopyridin-1(2*H*)-yl}-4-methoxy-*N*-(2-methyl-2*H*-indazol-5-yl)butanamide (racemate 49).**

Propylphosphonic anhydride (T3P, 50% solution in ethyl acetate, 130 µL, 0.22 mmol, 1.6 eq.) was added under argon atmosphere at RT to a solution of 2-{4-[5-chloro-2-(4,5-dihydro-1,2-oxazol-3-yl)phenyl]-5-methoxy-2-oxopyridin-1(2*H*)-yl}-4-methoxybutanoic acid hydrochloride (racemate **276**) (66 mg, 0.14 mmol) and 2-methyl-2*H*-indazole-5-amine (31 mg, 0.21 mmol, 1.5 eq.) in pyridine (1 mL). The reaction mixture was stirred at 50 °C for 1 h, cooled to RT, diluted with acetonitrile / water and purified by preparative RP-HPLC (acetonitrile / 0.1% formic acid in water gradient) to give **49**. Yield: 64 mg (83% of theory). LC/MS (method 3): $t_R$ = 1.55 min, MS (ESIpos): *m*/*z* = 550 $[M+H]^+$; $^1$H NMR (400 MHz, DMSO-$d_6$): δ [ppm] = 10.34 (s, 1H), 8.25 (s, 1H), 8.13 (d, *J* = 1.2 Hz, 1H), 7.65 (d, *J* = 8.3 Hz, 1H), 7.59 (dd, *J* = 8.4 Hz, 2.2 Hz, 1H), 7.55 (d, *J* = 9.2 Hz, 1H), 7.45 (d, *J* = 2.1 Hz, 1H), 7.37 (s, 1H), 7.31 (dd, *J* = 9.2 Hz, 2.0 Hz, 1H), 6.36 (s, 1H), 5.81-5.71 (m, 1H), 4.32-4.22 (m, 2H), 4.13 (s, 3H), 3.58 (s, 3H), 3.40-3.18 (m, 4H, partially concealed), 3.21 (s, 3H), 2.43-2.26 (m, 2H).

**Synthesis of racemate 50.**[a]

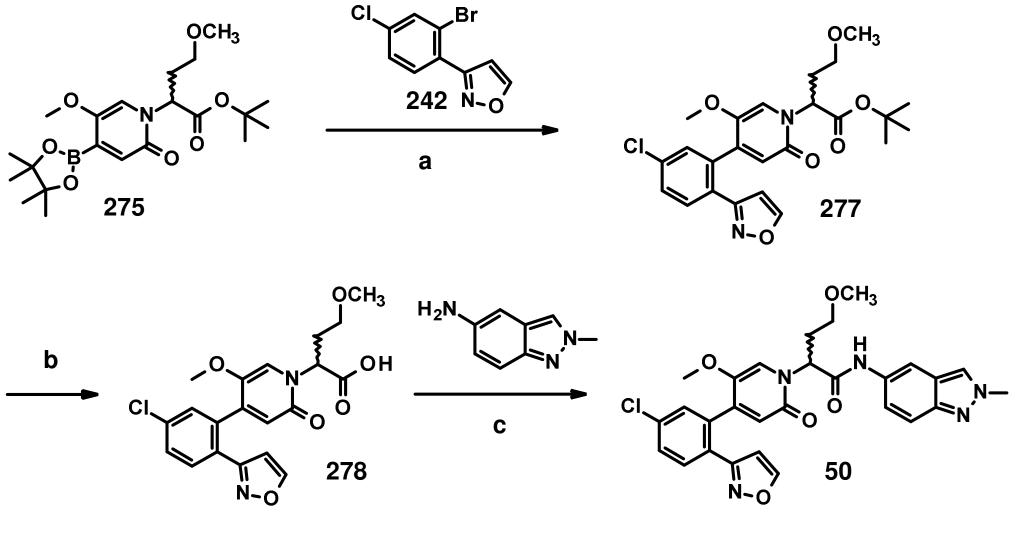

[a]Reagents and conditions: (a) Pd(dppf)$Cl_2$-DCM complex, $K_2CO_3$, 1,4-dioxane, 80 °C, 57%; (b) aq. LiOH, THF, 35 °C, 94%; (c) T3P, pyridine, 50 °C, 68%.

***tert*-Butyl 2-{4-[5-chloro-2-(1,2-oxazol-3-yl)phenyl]-5-methoxy-2-oxopyridin-1(2*H*)-yl}-4-methoxybutanoate (racemate 277).**

A mixture of *tert*-butyl 4-methoxy-2-[5-methoxy-2-oxo-4-(4,4,5,5-tetramethyl-1,3,2-dioxaborolan-2-yl)pyridin-1(2*H*)-yl]butanoate (racemate **275**) (264 mg, 0.63 mmol) in 1,4-dioxane (2.76 mL), 3-(2-bromo-4-chlorophenyl)-1,2-oxazole (**242**) (170 mg, 95% purity, 0.63 mmol, 1.0 eq.) and potassium carbonate (259 mg, 1.87 mmol, 3.0 eq.) in 1,4-dioxane (3.8 mL) was flushed with argon at RT for 5 min, followed by addition of [1,1'-bis(diphenylphosphino)ferrocene]dichloropalladium-dichloromethane complex (30.6 mg, 0.04 mmol, 0.06 eq.). The reaction mixture was stirred at 80 °C overnight, cooled to RT, filtered through kieselguhr, and the filter residue was washed with dichloromethane and acetonitrile. The combined filtrates were concentrated under reduced pressure. The residue was purified by flash silica gel chromatography (cyclohexane / ethyl acetate gradient) to give **277**. Yield: 210 mg (80% purity, 57% of theory). LC/MS (method 1): $t_R$ = 1.03 min, MS (ESIpos): $m/z$ = 475 $[M+H]^+$.

**2-{4-[5-Chloro-2-(1,2-oxazol-3-yl)phenyl]-5-methoxy-2-oxopyridin-1(2*H*)-yl}-4-methoxybutanoic acid (racemate 278).**

Lithium hydroxide solution (0.5 M in water, 3.54 mL, 1.77 mmol) was added at RT to a solution of *tert*-butyl 2-{4-[5-chloro-2-(1,2-oxazol-3-yl)phenyl]-5-methoxy-2-oxopyridin-1(2*H*)-yl}-4-methoxy-butanoate (racemate **277**) (210 mg, 80% purity, 0.35 mmol) in tetrahydrofuran (7.6 mL). The reaction mixture was stirred at 35 °C for 20 h, cooled to RT, neutralized with aqueous hydrochloric acid solution (1 N) and purified by preparative RP-HPLC (acetonitrile / 0.1% formic acid in water gradient) to give **278**. Yield: 140 mg (94% of theory). LC/MS (method 1): $t_R$ = 0.77 min, MS (ESIpos): $m/z$ = 419 $[M+H]^+$.

**2-{4-[5-Chloro-2-(1,2-oxazol-3-yl)phenyl]-5-methoxy-2-oxopyridin-1(2*H*)-yl}-4-methoxy-*N*-(2-methyl-2*H*-indazol-5-yl)butanamide (racemate 50).**

Propylphosphonic anhydride (T3P, 50% solution in ethyl acetate, 199 $\mu$L, 0.33 mmol, 4.0 eq.) was added under argon atmosphere at RT to a solution of 2-{4-[5-chloro-2-(1,2-oxazol-3-yl)phenyl]-5-methoxy-2-oxopyridin-1(2*H*)-yl}-4-methoxybutanoic acid (racemate **278**) (35 mg, 0.08 mmol) and 2-methyl-2*H*-indazole-5-amine (19 mg, 0.13 mmol, 1.5 eq.) in pyridine (0.74 mL). The reaction mixture was stirred at 50 °C for 1.5 h, cooled to RT and purified by preparative RP-HPLC (acetonitrile / 0.1% formic acid in water gradient) to give **50**. Yield: 31 mg (68% of theory). LC/MS (method 1): $t_R$ = 0.91 min, MS (ESIpos): *m*/*z* = 548 $[M+H]^+$; $^1$H NMR (500 MHz, DMSO-$d_6$): δ [ppm] = 10.33 (br s, 1H), 8.91 (d, *J* = 1.5 Hz, 1H), 8.25 (s, 1H), 8.12 (d, *J* = 1.3 Hz, 1H), 7.74 (d, *J* = 8.4 Hz, 1H), 7.64 (dd, *J* = 8.4 Hz, 2.3 Hz, 1H), 7.55 (d, *J* = 9.2 Hz, 1H), 7.53 (d, *J* = 2.2 Hz, 1H), 7.32 (s, 1H), 7.31 (dd, *J* = 9.2 Hz, 1.9 Hz, 1H), 6.53 (d, *J* = 1.7 Hz, 1H), 6.38 (s, 1H), 5.76 (br s, 1H), 4.13 (s, 3H), 3.39-3.19 (m, 1H and others partially concealed), 3.22 (s, 3H), 2.42-2.28 (m, 2H).

**Synthesis of racemate 51.**[a]

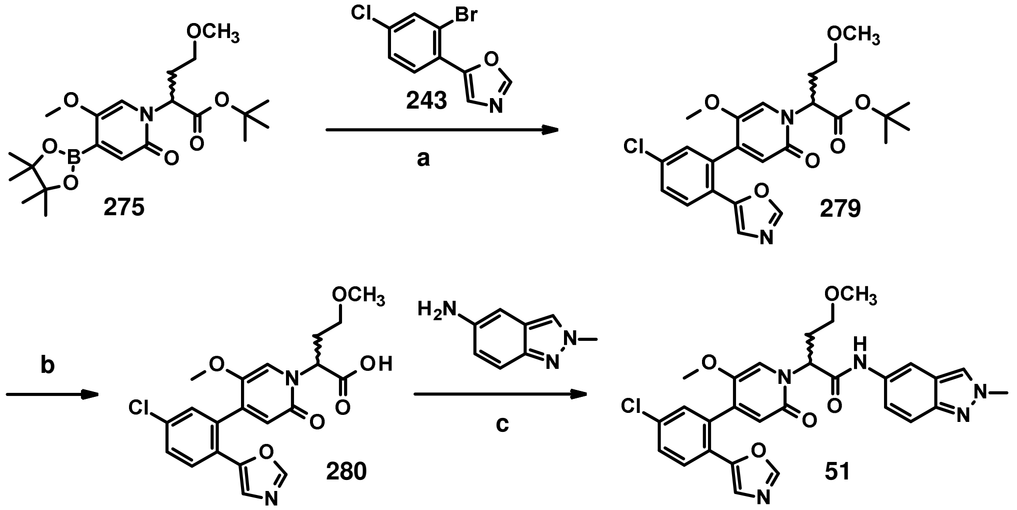


[a]Reagents and conditions: (a) Pd(dppf)$Cl_2$-DCM complex, $K_2CO_3$, 1,4-dioxane, 80 °C, 71%; (b) HCl/1,4-dioxane, RT, 97%; (c) T3P, pyridine, 50 °C, 52%.

***tert*-Butyl 2-{4-[5-chloro-2-(1,3-oxazol-5-yl)phenyl]-5-methoxy-2-oxopyridin-1(2*H*)-yl}-4-methoxybutanoate (racemate 279).**

A mixture of *tert*-butyl 4-methoxy-2-[5-methoxy-2-oxo-4-(4,4,5,5-tetramethyl-1,3,2-dioxaborolan-2-yl)pyridin-1(2*H*)-yl]butanoate (racemate **275**) (1.27 g. 65% purity, 1.95 mmol), 5-(2-bromo-4-chlorophenyl)-1,3-oxazole (**243**) (504 mg, 1.95 mmol, 1.0 eq.) and potassium carbonate (808 mg, 5.85 mmol, 3.0 eq.) in 1,4-dioxane (20 mL) was flushed with argon at RT for 5 min, followed by addition of [1,1'-bis(diphenylphosphino)ferrocene]dichloropalladium-dichloromethane complex (48 mg,

0.06 mmol, 0.03 eq.). The reaction mixture was stirred at 80 °C for 1 d, cooled to RT, filtered through kieselguhr, and the filter residue was washed with dichloromethane and acetonitrile. The combined filtrates were concentrated under reduced pressure. The residue was purified by flash silica gel chromatography (dichloromethane / methanol gradient) and subsequently by preparative RP-HPLC (acetonitrile / 0.1% formic acid in water gradient) to give **279**. Yield: 660 mg (71% of theory). LC/MS (method 1): $t_R$ = 1.02 min, MS (ESIpos): *m*/*z* = 475 $[M+H]^+$; $^1$H NMR (400 MHz, DMSO-$d_6$): δ [ppm] = 8.38 (s, 1H), 7.77 (d, *J* = 8.4 Hz, 1H), 7.62 (dd, *J* = 8.4 Hz, 2.2 Hz, 1H), 7.46 (d, *J* = 2.2 Hz, 1H), 7.27 (s, 1H), 6.79 (s, 1H), 6.39 (s, 1H), 5.18-4.95 (m, 1H), 3.45-3.33 (m, 1H, partially concealed), 3.38 (s, 3H), 3.22 (s, 3H), 3.21-3.13 (m, 1H), 2.39-2.27 (m, 2H), 1.42 (s, 9H).

**2-{4-[5-Chloro-2-(1,3-oxazol-5-yl)phenyl]-5-methoxy-2-oxopyridin-1(2*H*)-yl}-4-methoxybutanoic acid (racemate 280).**

A mixture of *tert*-butyl 2-{4-[5-chloro-2-(1,3-oxazol-5-yl)phenyl]-5-methoxy-2-oxopyridin-1(2*H*)-yl}-4-methoxybutanoate (racemate **279**) (660 mg, 1.38 mmol) in hydrogen chloride solution (4 M in 1,4-dioxane, 13 mL) was stirred at RT overnight and concentrated under reduced pressure (at maximal 25 °C). The residue was coevaporated with tetrahydrofuran and dried *in vacuo* to give **280** which was used without further purification. Yield: 700 mg (80% purity, 97% of theory). LC/MS (method 3): $t_R$ = 1.39 min, MS (ESIpos): *m*/*z* = 419 $[M+H]^+$; $^1$H NMR (400 MHz, DMSO-$d_6$): δ [ppm] = 8.38 (s, 1H), 7.77 (d, *J* = 8.4 Hz, 1H), 7.62 (dd, *J* = 8.6 Hz, 2.2 Hz, 1H), 7.48 (d, *J* = 2.1 Hz, 1H), 7.30 (s, 1H), 6.83 (s, 1H), 6.37 (s, 1H), 5.14 (br s, 1H), 3.57 (s, 3H), 3.45-3.38 (m, 1H), 3.22 (s, 3H), 3.25-3.11 (m, 1H), 2.45-2.27 (m, 2H).

**2-{4-[5-Chloro-2-(1,3-oxazol-5-yl)phenyl]-5-methoxy-2-oxopyridin-1(2*H*)-yl}-4-methoxy-*N*-(2-methyl-2*H*-indazol-5-yl)butanamide (racemate 51).**

Propylphosphonic anhydride (T3P, 50% solution in ethyl acetate, 66 μL, 0.11 mmol, 1.6 eq.) was added under argon atmosphere at RT to a solution of 2-{4-[5-chloro-2-(1,3-oxazol-5-yl)phenyl]-5-methoxy-2-oxopyridin-1(2*H*)-yl}-4-methoxybutanoic acid (racemate **280**) (37 mg, 80% purity, 0.07 mmol) and 2-methyl-2*H*-indazole-5-amine (17 mg, 89% purity, 0.11 mmol, 1.5 eq.) in pyridine (0.58 mL). The reaction mixture was stirred at 50 °C for 2 h, cooled to RT and purified by preparative RP-HPLC (acetonitrile / 0.1% formic acid in water gradient) to give **51**. Yield: 20 mg (52% of theory). LC/MS (method 3): $t_R$ = 1.57 min, MS (ESIpos): *m*/*z* = 548 $[M+H]^+$; $^1$H NMR (400 MHz, DMSO-$d_6$): δ [ppm] = 10.35 (br s, 1H), 8.39 (s, 1H), 8.26 (s, 1H), 8.14 (d, *J* = 1.2 Hz, 1H), 7.78 (d, *J* = 8.6 Hz, 1H), 7.62 (dd, *J* = 8.6 Hz, 2.2 Hz, 1H), 7.55 (d, *J* = 9.2 Hz, 1H), 7.48 (d, *J* = 2.2 Hz, 1H), 7.43 (s, 1H), 7.32 (dd, *J* = 9.2 Hz, 2.0 Hz, 1H), 6.90 (s, 1H), 6.41 (s, 1H), 5.87-5.72 (m, 1H), 4.13 (s, 3H), 3.43 (s, 3H), 3.43-3.37 (m, 1H), 3.36-3.26 (m, 1H), 3.24 (s, 3H), 2.44-2.34 (m, 2H).

**Synthesis of racemate 52.**[a]

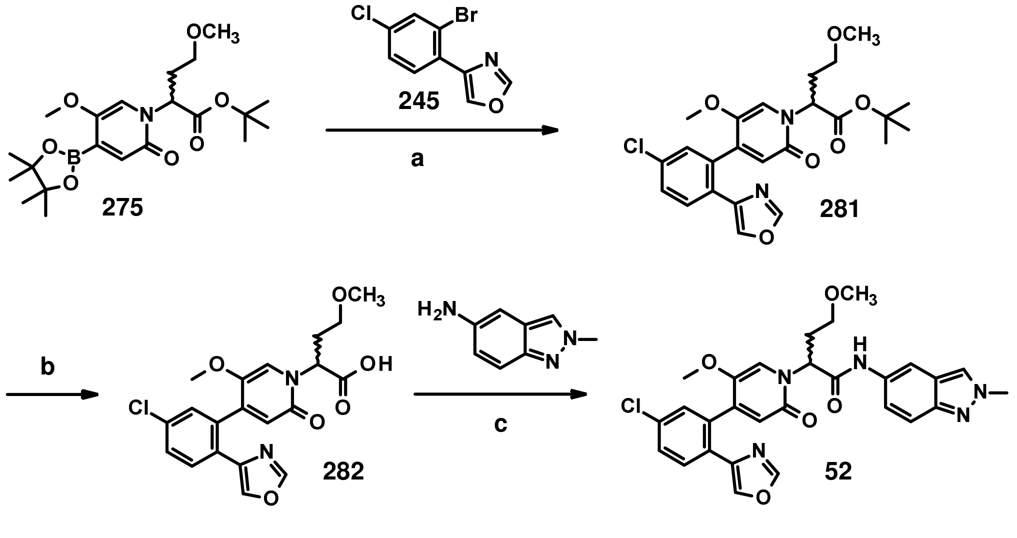


[a]Reagents and conditions: (a) Pd(dppf)$Cl_2$-DCM complex, $K_2CO_3$, 1,4-dioxane, 80 °C, 18%; (b) HCl/1,4-dioxane, RT, 100%; (c) T3P, pyridine, 50 °C, 48%.

***tert*-Butyl 2-{4-[5-chloro-2-(1,3-oxazol-4-yl)phenyl]-5-methoxy-2-oxopyridin-1(2*H*)-yl}-4-methoxybutanoate (racemate 281).**

A mixture of *tert*-butyl 4-methoxy-2-[5-methoxy-2-oxo-4-(4,4,5,5-tetramethyl-1,3,2-dioxaborolan-2-yl)pyridin-1(2*H*)-yl]butanoate (racemate **275**) (720 mg, 66% purity, 1.12 mmol) in 1,4-dioxane (2.9 mL), 4-(2-bromo-4-chlorophenyl)-1,3-oxazole (**245**) (290 mg, 1.12 mmol, 1.0 eq.) and potassium carbonate (465 mg, 3.37 mmol, 3.0 eq.) in 1,4-dioxane (9.1 mL) was flushed with argon at RT for 5 min, followed by addition of [1,1'-bis(diphenylphosphino)ferrocene]dichloropalladium-dichloromethane complex (27 mg, 0.03 mmol, 0.03 eq.). The reaction mixture was stirred at 80 °C for 18 h, cooled to RT, filtered through kieselguhr, and the filter residue was washed with dichloromethane and acetonitrile. The combined filtrates were concentrated under reduced pressure. The residue was purified by flash silica gel chromatography (cyclohexane / ethyl acetate gradient) to give **281** which was used without further purification. Yield: 140 mg (70% purity, 18% of theory). LC/MS (method 3): $t_R$ = 1.91 min, MS (ESIpos): $m/z$ = 475 $[M+H]^+$.

**2-{4-[5-Chloro-2-(1,3-oxazol-4-yl)phenyl]-5-methoxy-2-oxopyridin-1(2*H*)-yl}-4-methoxybutanoic acid (racemate 282).**

A mixture of *tert*-butyl 2-{4-[5-chloro-2-(1,3-oxazol-4-yl)phenyl]-5-methoxy-2-oxopyridin-1(2*H*)-yl}-4-methoxybutanoate (racemate **281**) (140 mg, 70% purity, 0.21 mmol) in hydrogen chloride solution (4 M in 1,4-dioxane, 2.3 mL) was stirred at RT for 8 h and concentrated under reduced pressure (at maximal 25 °C). The residue was coevaporated with tetrahydrofuran and dried *in vacuo* to give **282** which was used without further purification. Yield: 135 mg (64% purity, 100% of theory). LC/MS (method 3): $t_R$ = 1.43 min, MS (ESIpos): $m/z$ = 419 $[M+H]^+$.

**2-{4-[5-Chloro-2-(1,3-oxazol-4-yl)phenyl]-5-methoxy-2-oxopyridin-1(2*H*)-yl}-4-methoxy-*N*-(2-methyl-2*H*-indazol-5-yl)butanamide (racemate 52).**

Propylphosphonic anhydride (T3P, 50% solution in ethyl acetate, 65 $\mu$L, 0.11 mmol, 1.6 eq.) was added under argon atmosphere at RT to a solution of 2-{4-[5-chloro-2-(1,3-oxazol-4-yl)phenyl]-5-methoxy-2-oxopyridin-1(2*H*)-yl}-4-methoxybutanoic acid (racemate **282**) (45 mg, 64% purity, 0.07 mmol) and 2-methyl-2*H*-indazole-5-amine (17 mg, 89% purity, 0.10 mmol, 1.5 eq.) in pyridine (0.57 mL). The reaction mixture was stirred at 50 °C for 2 h, cooled to RT and purified by preparative RP-HPLC (acetonitrile / 0.1% formic acid in water gradient) to give **52**. Yield: 18 mg (48% of theory). LC/MS (method 3): $t_R$ = 1.59 min, MS (ESIpos): $m/z$ = 548 $[M+H]^+$; $^1$H NMR (400 MHz, DMSO-$d_6$): δ [ppm] = 10.32 (br s, 1H), 8.43-8.39 (m, 1H), 8.26 (s, 1H), 8.13 (d, $J$ = 1.3 Hz, 1H), 7.88 (d, $J$ = 8.4 Hz, 1H), 7.68 (d, $J$ = 0.6 Hz, 1H), 7.58 (dd, $J$ = 8.4 Hz, 2.2 Hz, 1H), 7.55 (d, $J$ = 9.2 Hz, 1H), 7.43-7.36 (m, 2H), 7.31 (dd, $J$ = 9.2 Hz, 1.8 Hz, 1H), 6.36 (s, 1H), 5.82-5.73 (m, 1H), 4.13 (s, 3H), 3.45-3.38 (m, 1H, partially concealed), 3.42 (s, 3H), 3.37-3.28 (m, 1H, partially concealed), 3.24 (s, 3H), 2.43-2.34 (m, 2H).

**Synthesis of racemate 53.**[a]

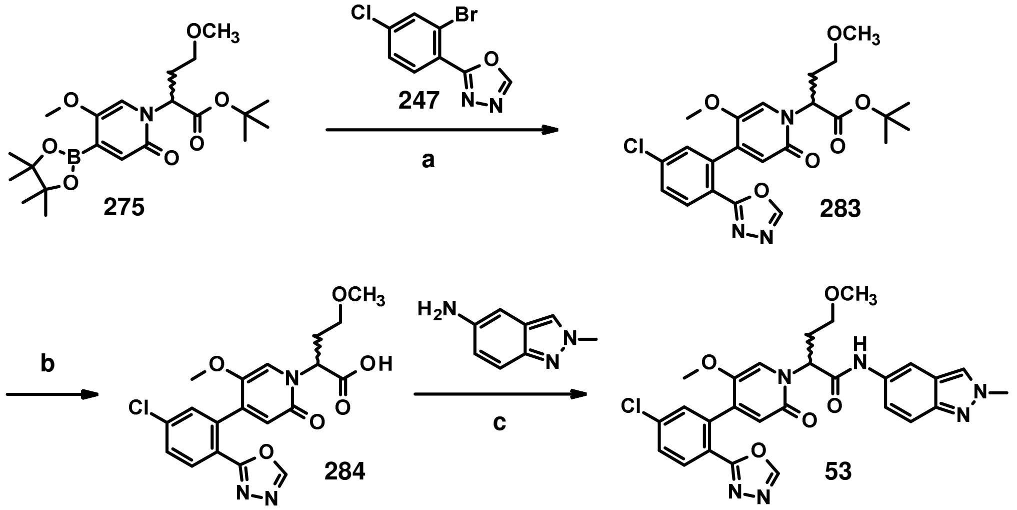


[a]Reagents and conditions: (a) Pd(dppf)$Cl_2$-DCM complex, $K_2CO_3$, 1,4-dioxane, 80 °C, 55%; (b) LiOH × $H_2O$, THF/water, RT, 34%; (c) T3P, pyridine, 50 °C, 39%.

***tert*-Butyl 2-{4-[5-chloro-2-(1,3,4-oxadiazol-2-yl)phenyl]-5-methoxy-2-oxopyridin-1(2*H*)-yl}-4-methoxybutanoate (racemate 283).**

A mixture of *tert*-butyl 4-methoxy-2-[5-methoxy-2-oxo-4-(4,4,5,5-tetramethyl-1,3,2-dioxaborolan-2-yl)pyridin-1(2*H*)-yl]butanoate (racemate **275**) (704 mg, 66% purity, 1.10 mmol), 2-(2-bromo-4-chlorophenyl)-1,3,4-oxadiazole (**247**) (300 mg, 1.10 mmol, 1.0 eq.) and potassium carbonate (455 mg, 3.30 mmol, 3.0 eq.) in 1,4-dioxane (11 mL) was flushed with argon at RT for 5 min, followed by addition of [1,1'-bis(diphenylphosphino)ferrocene]dichloropalladium-dichloromethane complex (27 mg,

0.03 mmol, 0.03 eq.). The reaction mixture was stirred at 80 °C for 12 h, cooled to RT, filtered through kieselguhr, and the filter residue was washed with dichloromethane and acetonitrile. The combined filtrates were concentrated under reduced pressure. The residue was purified by flash silica gel chromatography (dichloromethane / methanol gradient) to give **283** which was used without further purification. Yield: 410 mg (70% purity, 55% of theory). LC/MS (method 3): $t_R$ = 1.76 min, MS (ESIpos): *m/z* = 476 $[M+H]^+$.

**2-{4-[5-Chloro-2-(1,3,4-oxadiazol-2-yl)phenyl]-5-methoxy-2-oxopyridin-1(2*H*)-yl}-4-methoxybutanoic acid (racemate 284).**

A solution of lithium hydroxide monohydrate (127 mg, 3.02 mmol, 5.0 eq.) in water (18 mL) was added at RT to a solution of *tert*-butyl 2-{4-[5-chloro-2-(1,3,4-oxadiazol-2-yl)phenyl]-5-methoxy-2-oxopyridin-1(2*H*)-yl}-4-methoxybutanoate (racemate **283**) (410 mg, 70% purity, 0.60 mmol) in tetrahydrofuran (9 mL) and ethanol (18 mL). The reaction mixture was stirred at RT for 7 h, neutralized with aqueous hydrochloric acid solution (1 N) and concentrated under reduced pressure. After addition of ethyl acetate / water and phase separation, the aqueous phase was extracted with ethyl acetate. The combined organic phases were dried over anhydrous magnesium sulfate, filtered and concentrated under reduced pressure. The residue was purified by preparative RP-HPLC (acetonitrile / 0.1% formic acid in water gradient) to give **284** which was used without further purification. Yield: 100 mg (86% purity, 34% of theory). LC/MS (method 3): $t_R$ = 1.21 min, MS (ESIpos): *m/z* = 420 $[M+H]^+$.

**2-{4-[5-Chloro-2-(1,3,4-oxadiazol-2-yl)phenyl]-5-methoxy-2-oxopyridin-1(2*H*)-yl}-4-methoxy-*N*-(2-methyl-2*H*-indazol-5-yl)butanamide (racemate 53).**

Propylphosphonic anhydride (T3P, 50% solution in ethyl acetate, 58 $\mu$L, 0.10 mmol, 1.6 eq.) was added under argon atmosphere at RT to a solution of 2-{4-[5-chloro-2-(1,3,4-oxadiazol-2-yl)phenyl]-5-methoxy-2-oxopyridin-1(2*H*)-yl}-4-methoxybutanoic acid (racemate **284**) (30 mg, 86% purity, 0.06 mmol) and 2-methyl-2*H*-indazole-5-amine (15 mg, 89% purity, 0.09 mmol, 1.5 eq.) in pyridine (1 mL). The reaction mixture was stirred at 50 °C for 1 h, cooled to RT and purified by preparative RP-HPLC (acetonitrile / 0.1% formic acid in water gradient) and subsequently by flash silica gel chromatography (dichloromethane / methanol gradient) to give **53**. Yield: 13 mg (39% of theory). LC/MS (method 1): $t_R$ = 0.79 min, MS (ESIpos): *m/z* = 549 $[M+H]^+$; $^1H$ NMR (400 MHz, DMSO-$d_6$): δ [ppm] = 10.37 (s, 1H), 9.26 (s, 1H), 8.25 (s, 1H), 8.14 (d, *J* = 1.2 Hz, 1H), 8.01 (d, *J* = 8.4 Hz, 1H), 7.74 (dd, *J* = 8.4 Hz, 2.2 Hz, 1H), 7.64 (d, *J* = 2.1 Hz, 1H), 7.55 (d, *J* = 9.2 Hz, 1H), 7.35 (s, 1H), 7.32 (dd, *J* = 9.2 Hz, 2.0 Hz, 1H), 6.49 (s, 1H), 5.82-5.73 (m, 1H), 4.13 (s, 3H), 3.43-3.3 (m, 2H, partially concealed), 3.35 (s, 3H), 3.24 (s, 3H), 2.43-2.28 (m, 2H).

**Synthesis of racemate 54.**[a]

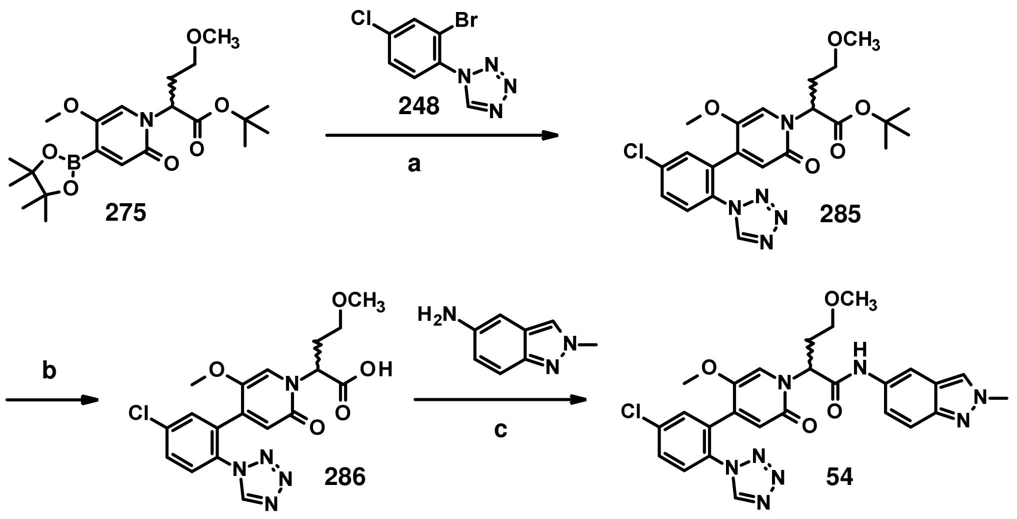

[a]Reagents and conditions: (a) Pd(dppf)$Cl_2$-DCM complex, $K_2CO_3$, 1,4-dioxane, 80 °C, 19%; (b) HCl/1,4-dioxane, RT, 90%; (c) T3P, pyridine, 50 °C, 64%.

***tert*-Butyl 2-{4-[5-chloro-2-(1*H*-tetrazol-1-yl)phenyl]-5-methoxy-2-oxopyridin-1(2*H*)-yl}-4-methoxybutanoate (racemate 285).**

A mixture of *tert*-butyl 4-methoxy-2-[5-methoxy-2-oxo-4-(4,4,5,5-tetramethyl-1,3,2-dioxaborolan-2-yl)pyridin-1(2*H*)-yl]butanoate (racemate **275**) (425 mg, 70% purity, 0.70 mmol), 1-(2-bromo-4-chlorophenyl)-1*H*-tetrazole (**248**) (225 mg, 81% purity, 0.70 mmol, 1.0 eq.) and potassium carbonate (291 mg, 2.11 mmol, 3.0 eq.) in 1,4-dioxane (7 mL) was flushed with argon at RT for 5 min, followed by addition of [1,1'-bis(diphenylphosphino)ferrocene]dichloropalladium-dichloromethane complex (17 mg, 0.02 mmol, 0.03 eq.). The reaction mixture was stirred at 80 °C for 3 d, cooled to RT, filtered through kieselguhr, and the filter residue was washed with dichloromethane. The combined filtrates were concentrated under reduced pressure. The residue was purified by preparative RP-HPLC (acetonitrile / 0.1% formic acid in water gradient) to give **285**. Yield: 64 mg (19% of theory). LC/MS (method 3): $t_R$ = 1.73 min, MS (ESIpos): $m/z$ = 476 $[M+H]^+$; $^1H$ NMR (400 MHz, DMSO-$d_6$): δ [ppm] = 9.64 (s, 1H), 7.86-7.70 (m, 3H), 7.03 (s, 1H), 6.47 (s, 1H), 5.01-4.91 (m, 1H), 3.52-3.40 (m, 1H), 3.23 (s, 3H), 3.17 (s, 3H), 3.09-2.98 (m, 1H), 2.29-2.20 (m, 2H), 1.38 (s, 9H).

**2-{4-[5-Chloro-2-(1*H*-tetrazol-1-yl)phenyl]-5-methoxy-2-oxopyridin-1(2*H*)-yl}-4-methoxybutanoic acid hydrochloride (racemate 286).**

A mixture of *tert*-butyl 2-{4-[5-chloro-2-(1*H*-tetrazol-1-yl)phenyl]-5-methoxy-2-oxopyridin-1(2*H*)-yl}-4-methoxybutanoate (racemate **285**) (64 mg, 0.13 mmol) in hydrogen chloride solution (4 M in 1,4-dioxane, 1.3 mL) was stirred at RT overnight and concentrated under reduced pressure (at maximal 25 °C). The residue was coevaporated with tetrahydrofuran and dried *in vacuo* to give **286** which was used without further purification. Yield: 58 mg (95% purity, 90% of theory). LC/MS (method 1): $t_R$ =

0.70 min, MS (ESIpos): $m/z$ = 420 [M+H]$^+$; $^1$H NMR (400 MHz, DMSO-$d_6$): δ [ppm] = 9.66 (s, 1H), 7.87-7.73 (m, 3H), 7.07 (s, 1H), 6.46 (s, 1H), 4.61 (br s, 1H), 3.34-3.25 (m, 1H), 3.23 (s, 3H), 3.16 (s, 3H), 3.04-2.91 (m, 1H), 2.34-2.22 (m, 2H).

**2-{4-[5-Chloro-2-(1*H*-tetrazol-1-yl)phenyl]-5-methoxy-2-oxopyridin-1(2*H*)-yl}-4-methoxy-*N*-(2-methyl-2*H*-indazol-5-yl)butanamide (racemate 54).**

Propylphosphonic anhydride (T3P, 50% solution in ethyl acetate, 56 μL, 0.10 mmol, 1.6 eq.) was added under argon atmosphere at RT to a solution of 2-{4-[5-chloro-2-(1*H*-tetrazol-1-yl)phenyl]-5-methoxy-2-oxopyridin-1(2*H*)-yl}-4-methoxybutanoic acid hydrochloride (racemate **286**) (29 mg, 95% purity, 0.06 mmol) and 2-methyl-2*H*-indazole-5-amine (15 mg, 0.09 mmol, 1.5 eq.) in pyridine (1 mL). The reaction mixture was stirred at 50 °C for 1.5 h, cooled to RT and purified by preparative RP-HPLC (acetonitrile / 0.1% formic acid in water gradient) to give **54**. Yield: 21 mg (64% of theory). LC/MS (method 1): $t_R$ = 0.77 min, MS (ESIpos): $m/z$ = 549 [M+H]$^+$; $^1$H NMR (400 MHz, DMSO-$d_6$): δ [ppm] = 10.30 (br s, 1H), 9.68 (s, 1H), 8.24 (s, 1H), 8.10 (s, 1H), 7.85-7.75 (m, 3H), 7.54 (d, $J$ = 9.2 Hz, 1H), 7.29 (dd, $J$ = 9.2 Hz, 1.8 Hz, 1H), 7.21 (s, 1H), 6.50 (s, 1H), 5.77-5.63 (m, 1H), 4.13 (s, 3H), 3.35-3.25 (m, 4H, partially concealed), 3.21 (s, 3H), 3.2-3.11 (m, 1H, partially concealed), 2.39-2.22 (m, 2H).

**Synthesis of racemate 55.**[a]

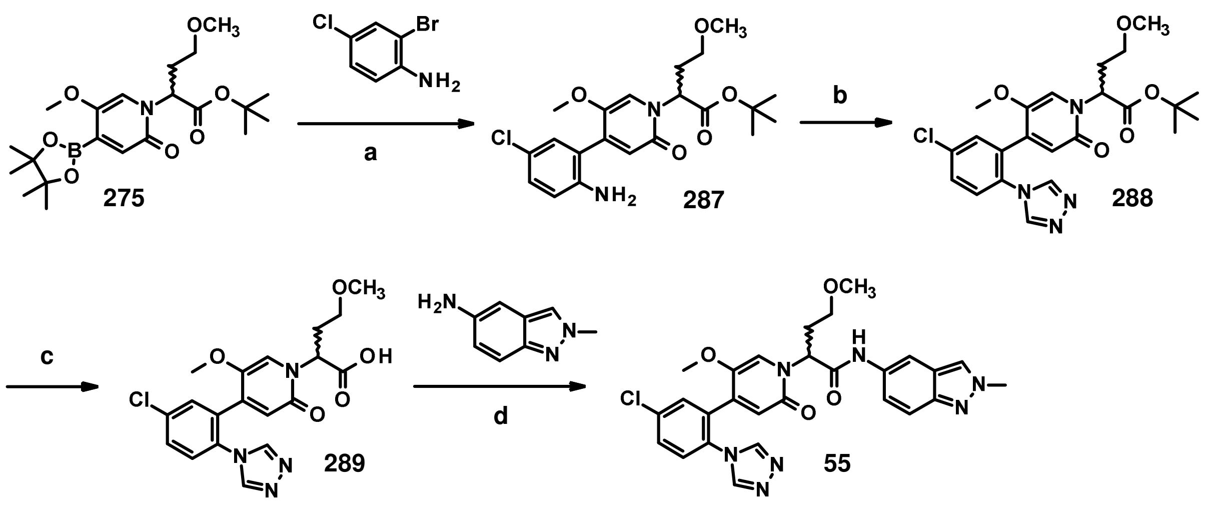


[a]Reagents and conditions: (a) Pd(dppf)$Cl_2$-DCM complex, $K_2CO_3$, 1,4-dioxane, 80 °C, 84%; (b) $Me_3SiCl$, *N'*-formylformic hydrazide, $NEt_3$, pyridine, 100 °C, 53%; (c) HCl/1,4-dioxane, RT, 79%; (d) T3P, pyridine, 50 °C, 70%.

***tert*-Butyl 2-[4-(2-amino-5-chlorophenyl)-5-methoxy-2-oxopyridin-1(2*H*)-yl]-4-methoxybutanoate (racemate 287).**

A mixture of *tert*-butyl 4-methoxy-2-[5-methoxy-2-oxo-4-(4,4,5,5-tetramethyl-1,3,2-dioxaborolan-2-yl)pyridin-1(2*H*)-yl]butanoate (racemate **275**) (718 mg, 1.70 mmol), 2-bromo-4-chloroaniline (350 mg,

1.70 mmol, 1.0 eq.) and potassium carbonate (703 mg, 5.09 mmol, 3.0 eq.) in 1,4-dioxane (18 mL) was flushed with argon at RT for 5 min, followed by addition of [1,1'-bis(diphenylphosphino)ferrocene]dichloropalladium-dichloromethane complex (83 mg, 0.10 mmol, 0.06 eq.). The reaction mixture was stirred at 80 °C for 3 d, cooled to RT, filtered through kieselguhr, and the filter residue was washed with dichloromethane. The combined filtrates were concentrated under reduced pressure. The residue was purified by flash silica gel chromatography (cyclohexane / ethyl acetate gradient) to give **287**. Yield: 674 mg (89% purity, 84% of theory). LC/MS (method 3): $t_R$ = 1.81 min, MS (ESIpos): *m*/*z* = 423 $[M+H]^+$.

***tert*-Butyl 2-{4-[5-chloro-2-(4*H*-1,2,4-triazol-4-yl)phenyl]-5-methoxy-2-oxopyridin-1(2*H*)-yl}-4-methoxybutanoate (racemate 288).**

Chlorotrimethylsilane (2.20 mL, 17.36 mmol, 15 eq.) was added dropwise under argon atmosphere at RT to a solution of *tert*-butyl 2-[4-(2-amino-5-chlorophenyl)-5-methoxy-2-oxopyridin-1(2*H*)-yl]-4-methoxybutanoate (racemate **287**) (550 mg, 89% purity, 1.16 mmol), *N*'-formylformic hydrazide (306 mg, 3.47 mmol, 3.0 eq.) and triethylamine (1.13 mL, 8.10 mmol, 7.0 eq.) in pyridine (12 mL). The reaction suspension was stirred at 100 °C for 5 h, cooled to RT, mixed with ethyl acetate (150 mL) and washed four times with water. The organic phase was dried over anhydrous sodium sulfate, filtered and concentrated under reduced pressure. The residue was purified by flash silica gel chromatography (dichloromethane / methanol gradient) to give **288**. Yield: 310 mg (94% purity, 53% of theory). LC/MS (method 1): $t_R$ = 0.87 min, MS (ESIpos): *m*/*z* = 475 $[M+H]^+$.

**2-{4-[5-Chloro-2-(4*H*-1,2,4-triazol-4-yl)phenyl]-5-methoxy-2-oxopyridin-1(2*H*)-yl}-4-methoxybutanoic acid hydrochloride (racemate 289).**

A mixture of *tert*-butyl 2-{4-[5-chloro-2-(4*H*-1,2,4-triazol-4-yl)phenyl]-5-methoxy-2-oxopyridin-1(2*H*)-yl}-4-methoxybutanoate (racemate **288**) (370 mg, 94% purity, 0.73 mmol) in hydrogen chloride solution (4 M in 1,4-dioxane, 18 mL) was stirred at RT overnight, concentrated under reduced pressure (at maximal 25 °C) and dried *in vacuo* to give **289** which was used without further purification. Yield: 378 mg (70% purity, 79% of theory). LC/MS (method 1): $t_R$ = 0.64 min, MS (ESIpos): *m*/*z* = 419 $[M+H]^+$.

**2-{4-[5-Chloro-2-(4*H*-1,2,4-triazol-4-yl)phenyl]-5-methoxy-2-oxopyridin-1(2*H*)-yl}-4-methoxy-*N*-(2-methyl-2*H*-indazol-5-yl)butanamide (racemate 55).**

Propylphosphonic anhydride (T3P, 50% solution in ethyl acetate, 230 µL, 0.39 mmol, 4.0 eq.) was added under argon atmosphere at RT to a solution of 2-{4-[5-chloro-2-(4*H*-1,2,4-triazol-4-yl)phenyl]-5-methoxy-2-oxopyridin-1(2*H*)-yl}-4-methoxybutanoic acid hydrochloride (racemate **289**) (63 mg, 70% purity, 0.10 mmol) and 2-methyl-2*H*-indazole-5-amine (22 mg, 0.15 mmol, 1.5 eq.) in pyridine (0.8 mL). The reaction mixture was stirred at 50 °C for 1.5 h, cooled to RT and purified by preparative RP-HPLC (acetonitrile / 0.1% formic acid in water gradient) to give **55**. Yield: 37 mg (70% of theory).

LC/MS (method 1): $t_R$ = 0.75 min, MS (ESIpos): $m/z$ = 548 $[M+H]^+$; $^1$H NMR (400 MHz, DMSO-$d_6$): δ [ppm] = 10.29 (br s, 1H), 8.55 (s, 2H), 8.24 (s, 1H), 8.11 (d, $J$ = 1.2 Hz, 1H), 7.74 (dd, $J$ = 8.6 Hz, 2.3 Hz, 1H), 7.68 (d, $J$ = 2.3 Hz, 1H), 7.64 (d, $J$ = 8.6 Hz, 1H), 7.53 (d, $J$ = 9.2 Hz, 1H), 7.29 (dd, $J$ = 9.2 Hz, 1.8 Hz, 1H), 7.22 (s, 1H), 6.49 (s, 1H), 5.71 (br s, 1H), 4.12 (s, 3H), 3.36 (s, 3H), 3.29-3.24 (m, 1H), 3.20 (s, 3H), 3.19-3.12 (m, 1H), 2.38-2.23 (m, 2H).

**Synthesis of racemate 56.**[a]

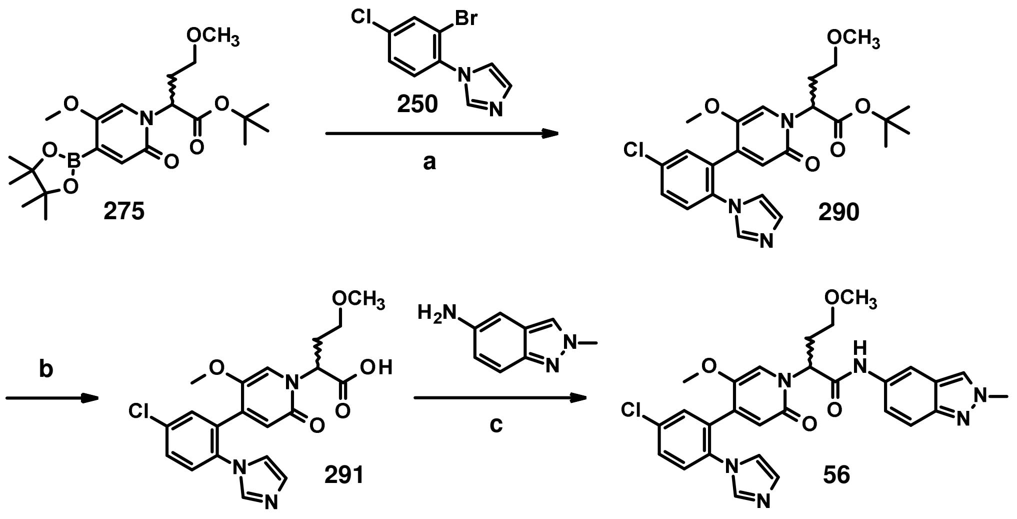


[a]Reagents and conditions: (a) Pd(dppf)$Cl_2$-DCM complex, $Cs_2CO_3$, 1,4-dioxane, 80 °C, 18%; (b) HCl/1,4-dioxane, RT, 99%; (c) T3P, pyridine, 50 °C, 44%.

***tert*-Butyl 2-{4-[5-chloro-2-(1*H*-imidazol-1-yl)phenyl]-5-methoxy-2-oxopyridin-1(2*H*)-yl}-4-methoxybutanoate (racemate 290).**

A mixture of *tert*-butyl 4-methoxy-2-[5-methoxy-2-oxo-4-(4,4,5,5-tetramethyl-1,3,2-dioxaborolan-2-yl)pyridin-1(2*H*)-yl]butanoate (racemate **275**) (368 mg, 58% purity, 0.50 mmol), 1-(2-bromo-4-chlorophenyl)-1*H*-imidazole (**250**) (130 mg, 0.50 mmol, 1.0 eq.) and cesium carbonate (492 mg, 1.51 mmol, 3.0 eq.) in 1,4-dioxane (5 mL) was flushed with argon at RT for 5 min, followed by addition of [1,1'-bis(diphenylphosphino)ferrocene]dichloropalladium-dichloromethane complex (41 mg, 0.05 mmol, 0.1 eq.). The reaction mixture was stirred at 80 °C overnight, cooled to RT, filtered through kieselguhr, and the filter residue was washed with dichloromethane. The combined filtrates were concentrated under reduced pressure. The residue was purified by flash silica gel chromatography (cyclohexane / ethyl acetate gradient) and subsequently by preparative RP-HPLC (acetonitrile / 0.1% formic acid in water gradient) to give **290**. Yield: 44 mg (18% of theory). LC/MS (method 3): $t_R$ = 1.33 min, MS (ESIpos): $m/z$ = 474 $[M+H]^+$; $^1$H NMR (400 MHz, DMSO-$d_6$): δ [ppm] = 7.67 (dd, $J$ = 8.4 Hz, 2.5 Hz, 1H), 7.63-7.57 (m, 2H), 7.53 (d, $J$ = 8.4 Hz, 1H), 7.12 (s, 1H), 7.06 (s, 1H), 6.89 (s, 1H), 6.42 (s, 1H), 5.01-4.92 (m, 1H), 3.36-3.24 (m, 4H), 3.17 (s, 3H), 3.09-2.97 (m, 1H), 2.30-2.19 (m, 2H),

1.38 (s, 9H).

**2-{4-[5-Chloro-2-(1*H*-imidazol-1-yl)phenyl]-5-methoxy-2-oxopyridin-1(2*H*)-yl}-4-methoxybutanoic acid hydrochloride (racemate 291).**

A mixture of *tert*-butyl 2-{4-[5-chloro-2-(1*H*-imidazol-1-yl)phenyl]-5-methoxy-2-oxopyridin-1(2*H*)-yl}-4-methoxybutanoate (racemate **290**) (42 mg, 0.09 mmol) in hydrogen chloride solution (4 M in 1,4-dioxane, 3 mL) was stirred at RT for 3 d, concentrated under reduced pressure (at maximal 25 °C) and dried *in vacuo* to give **291** which was used without further purification. Yield: 43 mg (92% purity, 99% of theory). LC/MS (method 1): $t_R$ = 0.52 min, MS (ESIpos): *m*/*z* = 418 $[M+H]^+$; $^1$H NMR (400 MHz, DMSO-$d_6$): δ [ppm] = 9.38 (s, 1H), 7.88-7.71 (m, 5H), 7.15 (s, 1H), 6.51 (s, 1H), 5.02 (br s, 1H), 3.36-3.25 (m, 4H), 3.15 (s, 3H), 3.03-2.90 (m, 1H), 2.35-2.23 (m, 2H).

**2-{4-[5-Chloro-2-(1*H*-imidazol-1-yl)phenyl]-5-methoxy-2-oxopyridin-1(2*H*)-yl}-4-methoxy-*N*-(2-methyl-2*H*-indazol-5-yl)butanamide (racemate 56).**

Propylphosphonic anhydride (T3P, 50% solution in ethyl acetate, 77 µL, 0.13 mmol, 1.6 eq.) was added under argon atmosphere at RT to a solution of 2-{4-[5-chloro-2-(1*H*-imidazol-1-yl)phenyl]-5-methoxy-2-oxopyridin-1(2*H*)-yl}-4-methoxybutanoic acid hydrochloride (racemate **291**) (41 mg, 90% purity, 0.08 mmol) and 2-methyl-2*H*-indazole-5-amine (20 mg, 89% purity, 0.12 mmol, 1.5 eq.) in pyridine (0.67 mL). The reaction mixture was stirred at 50 °C for 3 h, cooled to RT and purified by preparative RP-HPLC (acetonitrile / 0.1% formic acid in water gradient) to give **56**. Yield: 20 mg (44% of theory). LC/MS (method 3): $t_R$ = 1.05 min, MS (ESIpos): *m*/*z* = 547 $[M+H]^+$; $^1$H NMR (400 MHz, DMSO-$d_6$): δ [ppm] = 10.30 (s, 1H), 8.24 (s, 1H), 8.10 (d, *J* = 1.1 Hz, 1H), 7.74 (br s, 1H), 7.68 (dd, *J* = 8.6 Hz, 2.4 Hz, 1H), 7.61 (d, *J* = 2.4 Hz, 1H), 7.59-7.49 (m, 2H), 7.29 (dd, *J* = 9.2 Hz, 1.8 Hz, 1H), 7.25 (s, 1H), 7.19 (s, 1H), 6.97 (s, 1H), 6.45 (s, 1H), 5.79-5.63 (m, 1H), 4.13 (s, 3H), 3.37 (s, 3H), 3.35-3.11 (m, 2H, partially concealed), 3.20 (s, 3H), 2.39-2.21 (m, 2H).

**Synthesis of racemate 57.**[a]

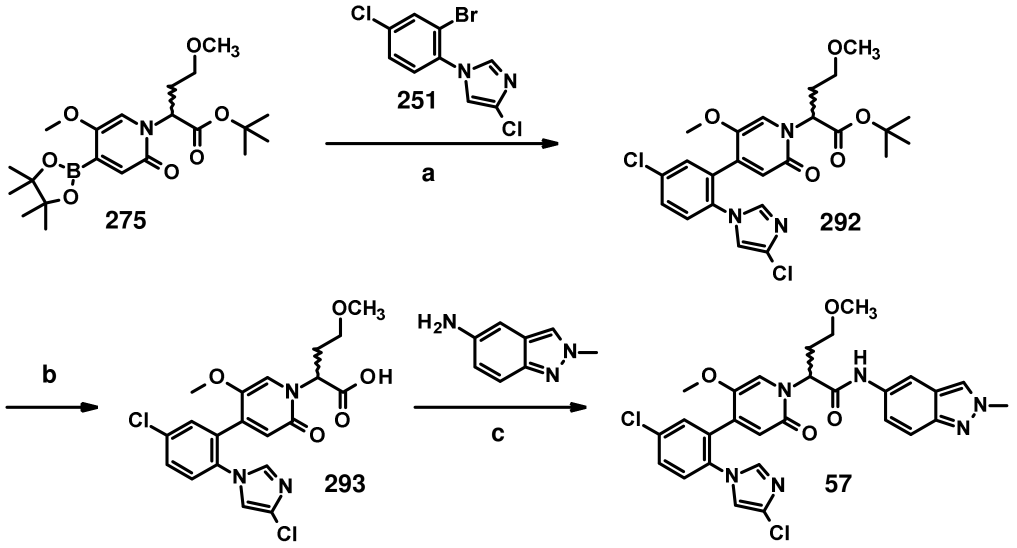

[a]Reagents and conditions: (a) Pd(dppf)$Cl_2$-DCM complex, $K_2CO_3$, 1,4-dioxane, 60 °C → 90 °C, 43%; (b) HCl/1,4-dioxane, RT, 97%; (c) T3P, pyridine, 50 °C, 78%.

***tert*-Butyl 2-{4-[5-chloro-2-(4-chloro-1*H*-imidazol-1-yl)phenyl]-5-methoxy-2-oxopyridin-1(2*H*)-yl}-4-methoxybutanoate (racemate 292).**

A mixture of *tert*-butyl 4-methoxy-2-[5-methoxy-2-oxo-4-(4,4,5,5-tetramethyl-1,3,2-dioxaborolan-2-yl)pyridin-1(2*H*)-yl]butanoate (racemate **275**) (1.20 g, 55% purity, 1.56 mmol), 1-(2-bromo-4-chlorophenyl)-4-chloro-1*H*-imidazole (**251**) (475 mg, 1.56 mmol, 1.0 eq.) and potassium carbonate (648 mg, 4.69 mmol, 3.0 eq.) in 1,4-dioxane (16 mL) was flushed with argon at RT for 5 min, followed by addition of [1,1'-bis(diphenylphosphino)ferrocene]dichloropalladium-dichloromethane complex (77 mg, 0.09 mmol, 0.06 eq.). The reaction mixture was stirred at 60 °C for 5 h, at 80 °C for 1 d and at 90 °C for 1 d, cooled to RT, filtered through kieselguhr, and the filter residue was washed with dichloromethane and acetonitrile. The combined filtrates were concentrated under reduced pressure. The residue was purified by flash silica gel chromatography (cyclohexane / ethyl acetate gradient) to give **292**. Yield: 345 mg (43% of theory). LC/MS (method 3): $t_R$ = 1.89 min, MS (ESIpos): $m/z$ = 508 $[M+H]^+$.

**2-{4-[5-Chloro-2-(4-chloro-1*H*-imidazol-1-yl)phenyl]-5-methoxy-2-oxopyridin-1(2*H*)-yl}-4-methoxybutanoic acid hydrochloride (racemate 293).**

A mixture of *tert*-butyl 2-{4-[5-chloro-2-(4-chloro-1*H*-imidazol-1-yl)phenyl]-5-methoxy-2-oxopyridin-1(2*H*)-yl}-4-methoxybutanoate (racemate **292**) (344 mg, 0.67 mmol) in hydrogen chloride solution (4 M in 1,4-dioxane, 6.7 mL) was stirred at RT overnight, concentrated under reduced pressure (at maximal 25 °C) and dried *in vacuo* to give **293** which was used without further purification. Yield: 345 mg (92% purity, 97% of theory). LC/MS (method 3): $t_R$ = 1.35 min, MS (ESIpos): $m/z$ = 452 $[M+H]^+$.

**2-{4-[5-Chloro-2-(4-chloro-1*H*-imidazol-1-yl)phenyl]-5-methoxy-2-oxopyridin-1(2*H*)-yl}-4-methoxy-*N*-(2-methyl-2*H*-indazol-5-yl)butanamide (racemate 57).**

Propylphosphonic anhydride (T3P, 50% solution in ethyl acetate, 134 $\mu$L, 0.23 mmol, 3.0 eq.) was added under argon atmosphere at RT to a solution of 2-{4-[5-chloro-2-(4-chloro-1*H*-imidazol-1-yl)phenyl]-5-methoxy-2-oxopyridin-1(2*H*)-yl}-4-methoxybutanoic acid hydrochloride (racemate **293**) (40 mg, 92% purity, 0.08 mmol) and 2-methyl-2*H*-indazole-5-amine (17 mg, 0.11 mmol, 1.5 eq.) in pyridine (1 mL). The reaction mixture was stirred at 50 °C for 1 h, cooled to RT and purified by preparative RP-HPLC (acetonitrile / 0.1% formic acid in water gradient) to give **57**. Yield: 37 mg (92% purity, 78% of theory). LC/MS (method 3): $t_R$ = 1.56 min, MS (ESIpos): *m*/*z* = 581 $[M+H]^+$; $^1$H NMR (400 MHz, DMSO-$d_6$): δ [ppm] = 10.29 (br s, 1H), 8.24 (s, 1H), 8.10 (d, *J* = 1.4 Hz, 1H), 7.70 (dd, *J* = 8.4 Hz, 2.3 Hz, 1H), 7.66-7.62 (m, 2H), 7.59 (d, *J* = 8.6 Hz, 1H), 7.53 (d, *J* = 9.2 Hz, 1H), 7.32-7.24 (m, 3H), 6.47 (s, 1H), 5.78-5.66 (m, 1H), 4.13 (s, 3H), 3.39 (s, 3H), 3.36-3.33 (m, 1H, partially concealed), 3.20 (s, 3H), 3.18-3.11 (m, 1H), 2.38-2.28 (m, 2H).

**Synthesis of racemate 58.**[a]

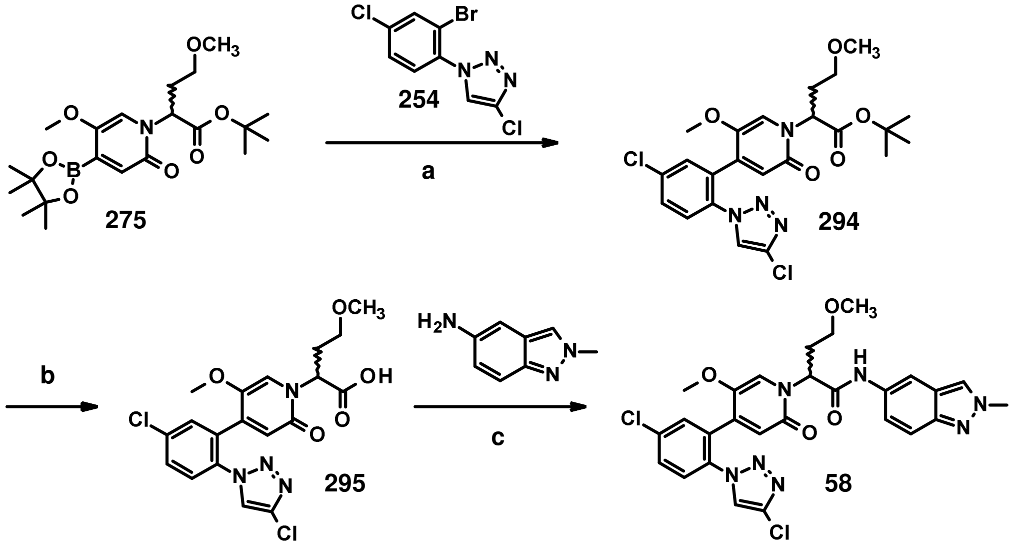


[a]Reagents and conditions: (a) Pd(dppf)$Cl_2$-DCM complex, $Na_2CO_3$, DMF/water, 100 °C, 54%; (b) HCl/1,4-dioxane, RT, 83%; (c) T3P, pyridine, 50 °C, 38%.

***tert*-Butyl 2-{4-[5-chloro-2-(4-chloro-1*H*-1,2,3-triazol-1-yl)phenyl]-5-methoxy-2-oxopyridin-1(2*H*)-yl}-4-methoxybutanoate (racemate 294).**

A mixture of *tert*-butyl 4-methoxy-2-[5-methoxy-2-oxo-4-(4,4,5,5-tetramethyl-1,3,2-dioxaborolan-2-yl)pyridin-1(2*H*)-yl]butanoate (racemate **275**) (1.04 g, 50% purity, 1.23 mmol, 1.2 eq.), 1-(2-bromo-4-chlorophenyl)-4-chloro-1*H*-1,2,3-triazole (**254**) (300 mg, 1.02 mmol) and sodium carbonate (326 mg, 3.07 mmol, 3.0 eq.) in *N*,*N*-dimethylformamide (2.7 mL) and water (0.85 mL) was flushed with argon

at RT for 10 min, followed by addition of [1,1'-bis(diphenylphosphino)ferrocene]dichloropalladium-dichloromethane complex (84 mg, 0.10 mmol, 0.1 eq.). The reaction mixture was stirred at 100 °C for 2 h, cooled to RT and mixed with ethyl acetate and water. After phase separation, the aqueous phase was extracted with ethyl acetate. The combined organic phases were dried over anhydrous magnesium sulfate, filtered and concentrated under reduced pressure. The residue was purified by flash silica gel chromatography (cyclohexane / ethyl acetate gradient) to give **294**. Yield: 348 mg (81% purity, 54% of theory). LC/MS (method 3): $t_R$ = 1.96 min, MS (ESIpos): *m*/*z* = 509 $[M+H]^+$.

**2-{4-[5-Chloro-2-(4-chloro-1*H*-1,2,3-triazol-1-yl)phenyl]-5-methoxy-2-oxopyridin-1(2*H*)-yl}-4-methoxybutanoic acid (racemate 295).**

A mixture of *tert*-butyl 2-{4-[5-chloro-2-(4-chloro-1*H*-1,2,3-triazol-1-yl)phenyl]-5-methoxy-2-oxopyridin-1(2*H*)-yl}-4-methoxybutanoate (racemate **294**) (348 mg, 81% purity, 0.55 mmol) in hydrogen chloride solution (4 M in 1,4-dioxane, 8.3 mL) was stirred at RT overnight and concentrated under reduced pressure. The residue was dried *in vacuo* to give **295** which was used without further purification. Yield: 280 mg (81% purity, 83% of theory). LC/MS (method 3): $t_R$ = 1.41 min, MS (ESIpos): *m*/*z* = 453 $[M+H]^+$.

**2-{4-[5-Chloro-2-(4-chloro-1*H*-1,2,3-triazol-1-yl)phenyl]-5-methoxy-2-oxopyridin-1(2*H*)-yl}-4-methoxy-*N*-(2-methyl-2*H*-indazol-5-yl)butanamide (racemate 58).**

Propylphosphonic anhydride (T3P, 50% solution in ethyl acetate, 354 $\mu$L, 0.60 mmol, 4.0 eq.) was added under argon atmosphere at RT to a solution of 2-{4-[5-chloro-2-(4-chloro-1*H*-1,2,3-triazol-1-yl)phenyl]-5-methoxy-2-oxopyridin-1(2*H*)-yl}-4-methoxybutanoic acid (racemate **295**) (83 mg, 81% purity, 0.15 mmol) and 2-methyl-2*H*-indazole-5-amine (37 mg, 89% purity, 0.22 mmol, 1.5 eq.) in pyridine (0.8 mL). The reaction mixture was stirred at 50 °C for 1 h, cooled to RT and purified by preparative RP-HPLC (acetonitrile / 0.1% formic acid in water gradient) to give **58**. Yield: 34 mg (38% of theory). LC/MS (method 3): $t_R$ = 1.58 min, MS (ESIpos): *m*/*z* = 582 $[M+H]^+$; $^1$H NMR (400 MHz, DMSO-$d_6$): δ [ppm] = 10.29 (br s, 1H), 8.63 (s, 1H), 8.25 (s, 1H), 8.10 (d, *J* = 1.3 Hz, 1H), 7.79 (dd, *J* = 8.5 Hz, 2.3 Hz, 1H), 7.76 (d, *J* = 8.4 Hz, 1H), 7.73 (d, *J* = 2.2 Hz, 1H), 7.54 (d, *J* = 9.2 Hz, 1H), 7.30 (dd, *J* = 9.2 Hz, 1.9 Hz, 1H), 7.24 (s, 1H), 6.46 (s, 1H), 5.76-5.65 (m, 1H), 4.13 (s, 3H), 3.40-3.28 (m, partially concealed), 3.21 (s, 3H), 3.19-3.13 (m, 1H), 2.38-2.26 (m, 2H).

**Synthesis of racemate 59.**[a]

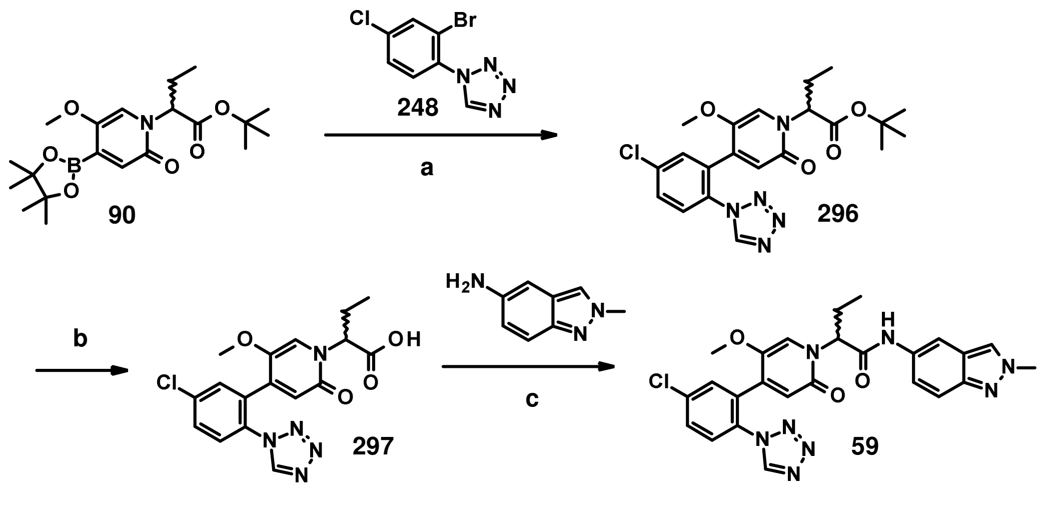

[a]Reagents and conditions: (a) Pd(dppf)$Cl_2$-DCM complex, $Na_2CO_3$, DMF/water, 100 °C, 46%; (b) HCl/1,4-dioxane, RT, 53%; (c) T3P, pyridine, 50 °C, 63%.

***tert*-Butyl 2-{4-[5-chloro-2-(1*H*-tetrazol-1-yl)phenyl]-5-methoxy-2-oxopyridin-1(2*H*)-yl}butanoate (racemate 296).**

A mixture of 1-(2-bromo-4-chlorophenyl)-1*H*-tetrazole (**248**) (400 mg, 1.54 mmol), *tert*-butyl 2-[5-methoxy-2-oxo-4-(4,4,5,5-tetramethyl-1,3,2-dioxaborolan-2-yl)pyridin-1(2*H*)-yl]butanoate (racemate **90**) (1.45 g, 50% purity, 1.85 mmol, 1.2 eq.) and sodium carbonate (490 mg, 4.62 mmol, 3.0 eq.) in a mixture of *N,N*-dimethylformamide (4.1 mL) and water (1.3 mL) was flushed with argon at RT for 10 min, followed by addition of [1,1'-bis(diphenylphosphino)ferrocene]dichloropalladium-dichloromethane complex (126 mg, 0.15 mmol, 0.1 eq.). The reaction mixture was stirred at 100 °C for 2 h and cooled to RT. After addition of ethyl acetate / water and phase separation, the aqueous phase was extracted with ethyl acetate. The combined organic phases were dried over anhydrous magnesium sulfate, filtered and concentrated under reduced pressure. The residue was purified by flash silica gel chromatography (cyclohexane / ethyl acetate gradient) to give **296** which was used without further purification. Yield: 313 mg (46% of theory). LC/MS (method 3): $t_R$ = 1.80 min, MS (ESIpos): *m*/*z* = 446 $[M+H]^+$.

**2-{4-[5-Chloro-2-(1*H*-tetrazol-1-yl)phenyl]-5-methoxy-2-oxopyridin-1(2*H*)-yl}butanoic acid (racemate 297).**

A mixture of *tert*-butyl 2-{4-[5-chloro-2-(1*H*-tetrazol-1-yl)phenyl]-5-methoxy-2-oxopyridin-1(2*H*)-yl}butanoate (racemate **296**) (313 mg, 0.70 mmol) in hydrogen chloride solution (4 M in 1,4-dioxane, 5.0 mL) was stirred at RT overnight. The precipitated solid was filtered, washed with 1,4-dioxane and diethyl ether and dried *in vacuo* to give **297** which was used without further purification. Yield: 144 mg (53% of theory). LC/MS (method 3): $t_R$ = 1.26 min, MS (ESIpos): *m*/*z* = 390 $[M+H]^+$.

**2-{4-[5-Chloro-2-(1*H*-tetrazol-1-yl)phenyl]-5-methoxy-2-oxopyridin-1(2*H*)-yl}-*N*-(2-methyl-2*H*-indazol-5-yl)butanamide (racemate 59).**

Propylphosphonic anhydride (T3P, 50% solution in ethyl acetate, 293 $\mu$L, 0.49 mmol, 4.0 eq.) was added under argon atmosphere at RT to a solution of 2-{4-[5-chloro-2-(1*H*-tetrazol-1-yl)phenyl]-5-methoxy-2-oxopyridin-1(2*H*)-yl}butanoic acid (racemate **297**) (48 mg, 0.12 mmol) and 2-methyl-2*H*-indazole-5-amine (27 mg, 0.19 mmol, 1.5 eq.) in pyridine (0.7 mL). The reaction mixture was stirred at 50 °C for 1 h, cooled to RT, diluted with acetonitrile / water and purified by preparative RP-HPLC (acetonitrile / 0.1% formic acid in water gradient) to give **59**. Yield: 41 mg (63% of theory). LC/MS (method 3): $t_R$ = 1.45 min, MS (ESIneg): *m*/*z* = 517 $[M-H]^-$; $^1H$ NMR (400 MHz, DMSO-$d_6$): δ [ppm] = 10.36 (s, 1H), 9.68 (s, 1H), 8.24 (s, 1H), 8.12 (s, 1H), 7.84-7.76 (m, 3H), 7.55 (d, *J* = 9.2 Hz, 1H), 7.27 (dd, *J* = 9.2 Hz, 2.0 Hz, 1H), 7.20 (s, 1H), 6.51 (s, 1H), 5.65-5.55 (m, 1H), 4.13 (s, 3H), 3.29 (s, 3H), 2.17-1.93 (m, 2H), 0.82 (t, *J* = 7.2 Hz, 3H).

**Synthesis of racemate 60and eutomer 61.**[a]

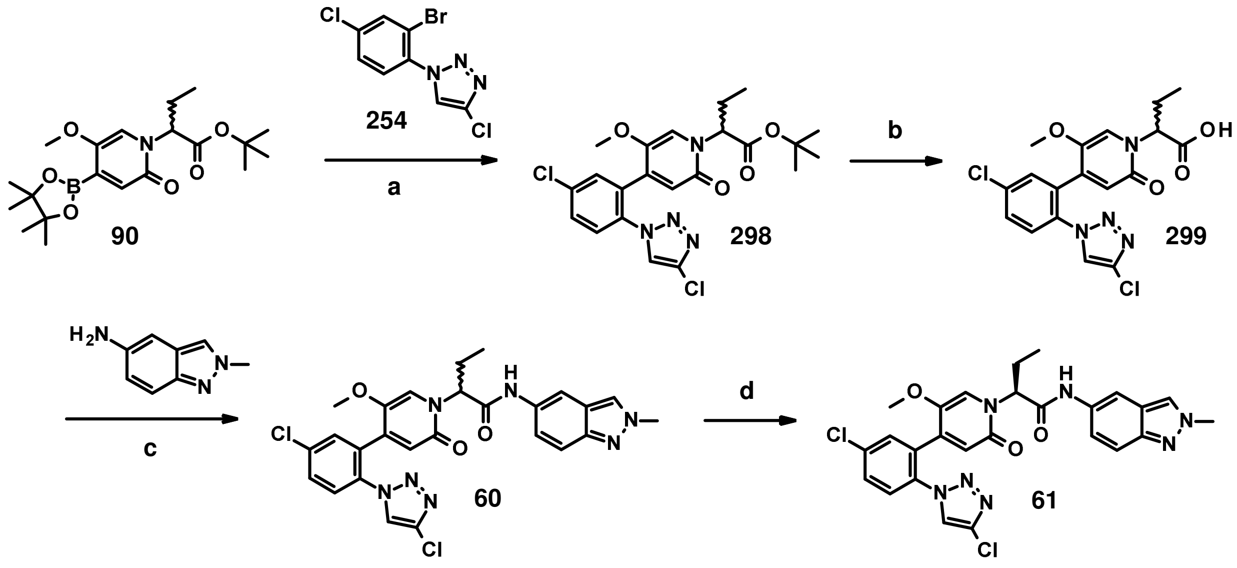


[a]Reagents and conditions: (a) Pd(dppf)$Cl_2$-DCM complex, $Na_2CO_3$, DMF/water, 100 °C, 55%; (b) HCl/1,4-dioxane, RT, 87%; (c) T3P, pyridine, 50 °C, 70%; (d) enantiomer separation.

***tert*-Butyl 2-{4-[5-chloro-2-(4-chloro-1*H*-1,2,3-triazol-1-yl)phenyl]-5-methoxy-2-oxopyridin-1(2*H*)-yl}butanoate (racemate 298).**

A mixture of *tert*-butyl 2-[5-methoxy-2-oxo-4-(4,4,5,5-tetramethyl-1,3,2-dioxaborolan-2-yl)pyridin-1(2*H*)-yl]butanoate (racemate **90**) (533 mg, 50% purity, 0.68 mmol, 1.2 eq.), 1-(2-bromo-4-chlorophenyl)-4-chloro-1*H*-1,2,3-triazole (**254**) (165 mg, 0.57 mmol) and sodium carbonate (180 mg, 1.69 mmol, 3.0 eq,) in a mixture of *N*,*N*-dimethylformamide / water (3:1, 2.0 mL) was flushed with argon at RT for 10 min, followed by addition of [1,1'-bis(diphenylphosphino)ferrocene]-

dichloropalladium-dichloromethane complex (46 mg, 0.05 mmol, 0.1 eq.). The reaction mixture was shaken at 100 °C for 2 h and cooled to RT. After addition of ethyl acetate / water and phase separation, the aqueous phase was extracted with ethyl acetate. The combined organic phases were dried over anhydrous magnesium sulfate, filtered and concentrated under reduced pressure. The residue was purified by flash silica gel chromatography (cyclohexane / ethyl acetate gradient) to give **298**. Yield: 148 mg (55% of theory). LC/MS (method 3): $t_R$ = 2.00 min, MS (ESIpos): *m*/*z* = 479 $[M+H]^+$; $^1$H NMR (400 MHz, DMSO-$d_6$): δ [ppm] = 8.59 (s, 1H), 7.81-7.72 (m, 3H), 7.06 (s, 1H), 6.43 (s, 1H), 4.95-4.88 (m, 1H), 3.28 (s, 3H), 2.09-1.98 (m, 2H), 1.39 (s, 9H), 0.77 (t, *J* = 7.4 Hz, 3H).

**2-{4-[5-Chloro-2-(4-chloro-1*H*-1,2,3-triazol-1-yl)phenyl]-5-methoxy-2-oxopyridin-1(2*H*)-yl}butanoic acid (racemate 299).**

Hydrogen chloride solution (4 M in 1,4-dioxane, 11.6 mL) was added to *tert*-butyl 2-{4-[5-chloro-2-(4-chloro-1*H*-1,2,3-triazol-1-yl)phenyl]-5-methoxy-2-oxopyridin-1(2*H*)-yl}butanoate (racemate **298**) (372 mg, 0.78 mmol). The reaction mixture was stirred at RT overnight and concentrated under reduced pressure. The residue was dried *in vacuo* to give **299** which was used without further purification. Yield: 307 mg (87% of theory). LC/MS (method 1): $t_R$ = 0.79 min, MS (ESIpos): *m*/*z* = 423 $[M+H]^+$; $^1$H NMR (400 MHz, DMSO-$d_6$): δ [ppm] = 8.59 (s, 1H), 7.81-7.72 (m, 3H), 7.11 (s, 1H), 6.43 (s, 1H), 5.08-4.94 (m, 1H), 3.57 (s, 3H), 2.14-2.02 (m, 2H), 0.75 (t, *J* = 7.4 Hz, 3H).

**2-{4-[5-Chloro-2-(4-chloro-1*H*-1,2,3-triazol-1-yl)phenyl]-5-methoxy-2-oxopyridin-1(2*H*)-yl}-*N*-(2-methyl-2*H*-indazol-5-yl)butanamide (racemate 60).**

Propylphosphonic anhydride (T3P, 50% solution in ethyl acetate, 567 µL, 0.95 mmol, 4.0 eq.) was added under argon atmosphere at RT to a solution of 2-{4-[5-chloro-2-(4-chloro-1*H*-1,2,3-triazol-1-yl)phenyl]-5-methoxy-2-oxopyridin-1(2*H*)-yl}butanoic acid (racemate **299**) (101 mg, 0.24 mmol) in pyridine (6.3 mL). The reaction mixture was heated to 50 °C, and 2-methyl-2*H*-indazole-5-amine (46 mg, 0.31 mmol, 1.3 eq.) was added. The reaction mixture was stirred at 50 °C for additional 1 h, cooled to RT, diluted with acetonitrile / water (1:1) and purified by preparative RP-HPLC (acetonitrile / 0.1% formic acid in water gradient) to give **60**. Yield: 93 mg (70% of theory). LC/MS (method 3): $t_R$ = 1.65 min, MS (ESIneg): *m*/*z* = 550 $[M-H]^-$; $^1$H NMR (400 MHz, DMSO-$d_6$): δ [ppm] = 10.35 (s, 1H), 8.63 (s, 1H), 8.25 (s, 1H), 8.13-8.10 (m, 1H), 7.82-7.72 (m, 3H), 7.55 (d, *J* = 9.2 Hz, 1H), 7.27 (dd, *J* = 9.2 Hz, 2.0 Hz, 1H), 7.24 (s, 1H), 6.47 (s, 1H), 5.64-5.57 (m, 1H), 4.13 (s, 3H), 3.32 (s, 3H, partially concealed), 2.17-1.98 (m, 2H), 0.83 (t, *J* = 7.2 Hz, 3H).

**(2*S*)-2-{4-[5-Chloro-2-(4-chloro-1*H*-1,2,3-triazol-1-yl)phenyl]-5-methoxy-2-oxopyridin-1(2*H*)-yl}-*N*-(2-methyl-2*H*-indazol-5-yl)butanamide (eutomer 61).**

Enantiomer separation of 93 mg of 2-{4-[5-chloro-2-(4-chloro-1*H*-1,2,3-triazol-1-yl)phenyl]-5-methoxy-2-oxopyridin-1(2*H*)-yl}-*N*-(2-methyl-2*H*-indazol-5-yl)butanamide (racemate **60**) gave 38 mg distomer (chiral HPLC: $t_R$ = 1.4 min) and 33 mg of eutomer **61** (chiral HPLC: $t_R$ = 3.6 min, >99% ee).

LC/MS (method 1): $t_R$ = 0.90 min, MS (ESIpos): $m/z$ = 552 $[M+H]^+$; $^1$H NMR (400 MHz, DMSO-$d_6$): δ [ppm] = 10.35 (s, 1H), 8.63 (s, 1H), 8.25 (s, 1H), 8.13-8.10 (m, 1H), 7.82-7.72 (m, 3H), 7.55 (d, $J$ = 9.2 Hz, 1H), 7.27 (dd, $J$ = 9.2 Hz, 2.0 Hz, 1H), 7.24 (s, 1H), 6.47 (s, 1H), 5.64-5.57 (m, 1H), 4.13 (s, 3H), 3.32 (s, 3H, partially concealed), 2.19-1.97 (m, 2H), 0.83 (t, $J$ = 7.2 Hz, 3H).
Separation method: SFC: column: Daicel Chiralpak AD-H 5µm, 250 mm × 20 mm; mobile phase: 70% carbon dioxide / 30% methanol; temperature: 40 °C; flow rate: 80 mL/min; UV detection: 210 nm.
Analytical method: SFC: column: Daicel Chiralpak AD-H 3 µm, 100 mm × 4.6 mm; mobile phase: 60% carbon dioxide / 40% methanol; temperature: 40 °C; flow rate: 3 mL/min; UV detection: 210 nm.

**Synthesis of racemate 62and eutomer 63.**[a]

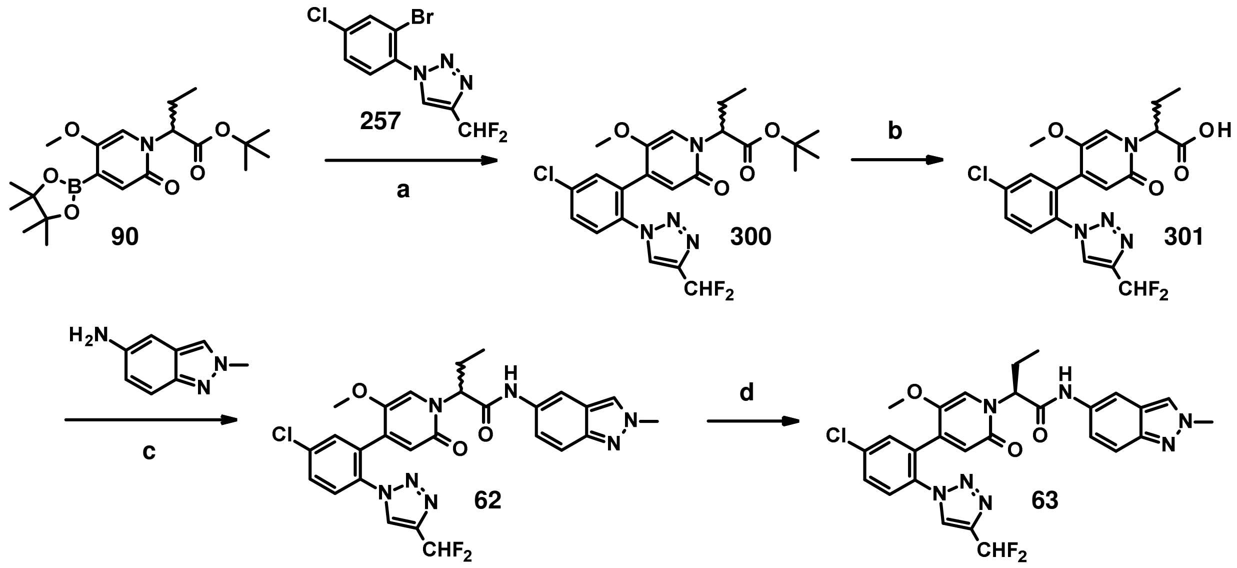


[a]Reagents and conditions: (a) Pd(dppf)$Cl_2$-DCM complex, $K_2CO_3$, 1,4-dioxane, 80 °C, 86%; (b) aq. LiOH, THF, RT, 76%; (c) T3P, pyridine, 50 °C, 70%; (d) enantiomer separation.

***tert*-Butyl 2-[4-{5-chloro-2-[4-(difluoromethyl)-1*H*-1,2,3-triazol-1-yl]phenyl}-5-methoxy-2-oxopyridin-1(2*H*)-yl]butanoate (racemate 300).**

A mixture of *tert*-butyl 2-[5-methoxy-2-oxo-4-(4,4,5,5-tetramethyl-1,3,2-dioxaborolan-2-yl)pyridin-1(2*H*)-yl]butanoate (racemate **90**) (1.95 g, 58% purity, 2.88 mmol), 1-(2-bromo-4-chlorophenyl)-4-(difluoromethyl)-1*H*-1,2,3-triazole (**257**) (888 mg, 2.88 mmol, 1.0 eq.) and potassium carbonate (1.19 g, 8.64 mmol, 3.0 eq.) in 1,4-dioxane (28.5 mL) was flushed with argon at RT for 5 min, followed by addition of [1,1'-bis(diphenylphosphino)ferrocene]dichloropalladium-dichloromethane complex (235 mg, 0.29 mmol, 0.1 eq.). The reaction mixture was stirred at 80 °C for 4 h, cooled to RT, filtered through Celite®, and the filter residue was washed with dichloromethane and acetonitrile. The combined filtrates were concentrated under reduced pressure. The residue was separated by flash silica gel chromatography (cyclohexane / ethyl acetate gradient) to give **300**. Yield: 1.22 g (86% of theory).

LC/MS (method 3): $t_R$ = 1.97 min, MS (ESIpos): $m/z$ = 495 $[M+H]^+$; $^1$H NMR (400 MHz, DMSO-$d_6$): δ [ppm] = 8.71 (s, 1H), 7.81-7.72 (m, 3H), 7.20 (t, $J$ = 54.0 Hz, 1H), 7.02 (s, 1H), 6.47 (s, 1H), 4.95-4.86 (m, 1H), 3.22 (s, 3H), 2.09-1.96 (m, 2H), 1.38 (s, 9H), 0.75 (t, $J$ = 7.3 Hz, 3H).

**2-[4-{5-Chloro-2-[4-(difluoromethyl)-1*H*-1,2,3-triazol-1-yl]phenyl}-5-methoxy-2-oxopyridin-1(2*H*)-yl]butanoic acid (racemate 301).**

Aqueous lithium hydroxide solution (1 M, 12.33 mL, 12.33 mmol, 5.0 eq.) was added to a solution of *tert*-butyl 2-[4-{5-chloro-2-[4-(difluoromethyl)-1*H*-1,2,3-triazol-1-yl]phenyl}-5-methoxy-2-oxopyridin-1(2*H*)-yl]butanoate (racemate **300**) (1.22 g, 2.47 mmol) in tetrahydrofuran (17.7 mL). The reaction mixture was stirred at RT overnight, mixed with water, acidified to pH 4 with aqueous hydrochloric acid solution (1 N) and extracted with ethyl acetate. The combined organic phases were washed with saturated aqueous sodium chloride solution, dried over anhydrous sodium sulfate, filtered and concentrated under reduced pressure to give **301** which was used without further purification. Yield: 826 mg (76% of theory). LC/MS (method 1): $t_R$ = 0.79 min, MS (ESIpos): $m/z$ = 439 $[M+H]^+$; $^1$H NMR (400 MHz, DMSO-$d_6$): δ [ppm] = 12.89 (s, 1H), 8.69 (s, 1H), 7.81-7.73 (m, 3H), 7.20 (t, $J$ = 54.0 Hz, 1H), 7.07 (s, 1H), 6.46 (s, 1H), 5.09-4.82 (m, 1H), 3.21 (s, 3H), 2.13-2.01 (m, 2H), 0.73 (t, $J$ = 7.4 Hz, 3H).

**2-[4-{5-Chloro-2-[4-(difluoromethyl)-1*H*-1,2,3-triazol-1-yl]phenyl}-5-methoxy-2-oxopyridin-1(2*H*)-yl]-*N*-(2-methyl-2*H*-indazol-5-yl)butanamide (racemate 62).**

Propylphosphonic anhydride (T3P, 50% solution in ethyl acetate, 661 μL, 1.11 mmol, 4 eq.) was added under argon atmosphere at RT to a solution of 2-[4-{5-chloro-2-[4-(difluoromethyl)-1*H*-1,2,3-triazol-1-yl]phenyl}-5-methoxy-2-oxopyridin-1(2*H*)-yl]butanoic acid (racemate **301**) (122 mg, 0.28 mmol) in pyridine (1.5 mL). The reaction mixture was heated to 50 °C, and 2-methyl-2*H*-indazole-5-amine (61 mg, 0.42 mmol, 1.3 eq.) was added. The reaction mixture was stirred at 50 °C for additional 1 h, cooled to RT, diluted with acetonitrile / water (1:1) and purified by preparative RP-HPLC (acetonitrile / 0.1% formic acid in water gradient) to give **62**. Yield: 111 mg (70% of theory). LC/MS (method 3): $t_R$ = 1.63 min, MS (ESIpos): $m/z$ = 568 $[M+H]^+$; $^1$H NMR (400 MHz, DMSO-$d_6$): δ [ppm] = 10.35 (s, 1H), 8.73 (s, 1H), 8.24 (s, 1H), 8.11 (d, $J$ = 1.2 Hz, 1H), 7.80-7.72 (m, 3H), 7.54 (d, $J$ = 9.2 Hz, 1H), 7.26 (dd, $J$ = 9.2 Hz, 1.9 Hz, 1H), 7.22 (t, $J$ = 54.0 Hz, 1H), 7.20 (s, 1H), 6.50 (s, 1H), 5.64-5.55 (m, 1H), 4.13 (s, 3H), 3.27 (s, 3H), 2.17-1.95 (m, 2H), 0.81 (t, $J$ = 7.2 Hz, 3H).

**(2*S*)-2-[4-{5-Chloro-2-[4-(difluoromethyl)-1*H*-1,2,3-triazol-1-yl]phenyl}-5-methoxy-2-oxopyridin-1(2*H*)-yl]-*N*-(2-methyl-2*H*-indazol-5-yl)butanamide (eutomer 63).**

Enantiomer separation of 110 mg of 2-[4-{5-chloro-2-[4-(difluoromethyl)-1*H*-1,2,3-triazol-1-yl]phenyl}-5-methoxy-2-oxopyridin-1(2*H*)-yl]-*N*-(2-methyl-2*H*-indazol-5-yl)butanamide (racemate **62**) gave 48 mg distomer (chiral HPLC: $t_R$ = 1.4 min) and 43 mg of eutomer **63** (chiral HPLC: $t_R$ = 2.9 min, >99% ee).

LC/MS (method 3): $t_R$ = 1.63 min, MS (ESIpos): $m/z$ = 568 $[M+H]^+$; $^1H$ NMR (400 MHz, DMSO-$d_6$): δ [ppm] = 10.35 (s, 1H), 8.73 (s, 1H), 8.24 (s, 1H), 8.11 (d, $J$ = 1.3 Hz, 1H), 7.80-7.72 (m, 3H), 7.54 (d, $J$ = 9.3 Hz, 1H), 7.26 (dd, $J$ = 9.2 Hz, 1.8 Hz, 1H), 7.22 (t, $J$ = 54.1 Hz, 1H), 7.20 (s, 1H), 6.50 (s, 1H), 5.64-5.56 (m, 1H), 4.13 (s, 3H), 3.27 (s, 3H), 2.17-1.95 (m, 2H), 0.81 (t, $J$ = 7.3 Hz, 3H).
Separation method: SFC: column: Daicel Chiralpak AD-H 5 μm, 250 mm × 20 mm; mobile phase: 70% carbon dioxide / 30% ethanol; temperature: 40 °C; flow rate: 80 mL/min; UV detection: 210 nm.
Analytical method: SFC: column: Daicel Chiralpak AD-H 3 μm, 100 mm × 4.6 mm; mobile phase: 70% carbon dioxide / 30% ethanol; temperature: 40 °C; flow rate: 3 mL/min; UV detection: 210 nm.

**Synthesis of racemate 64and eutomer 65.**[a]

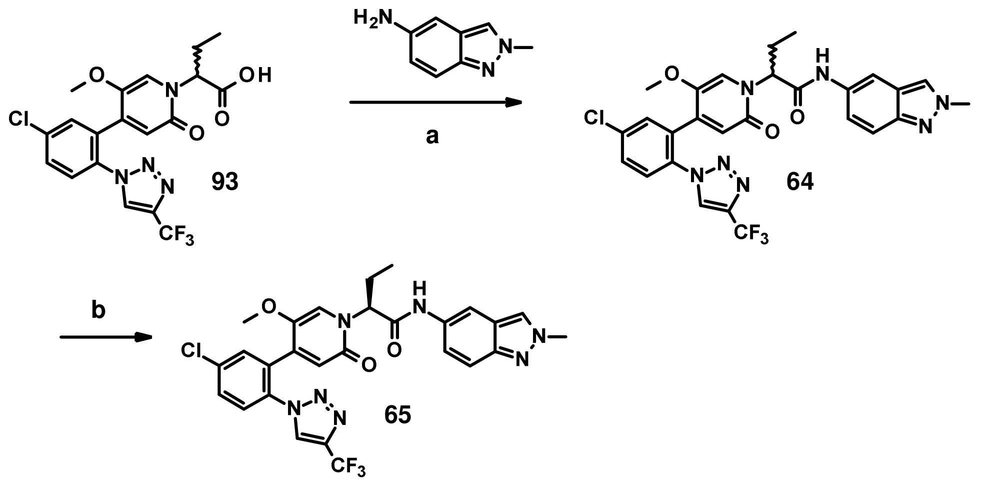

[a]Reagents and conditions: (a) T3P, pyridine, 50 °C, 77%; (b) enantiomer separation.

**2-[4-{5-Chloro-2-[4-(trifluoromethyl)-1*H*-1,2,3-triazol-1-yl]phenyl}-5-methoxy-2-oxopyridin-1(2*H*)-yl]-*N*-(2-methyl-2*H*-indazol-5-yl)butanamide (racemate 64).**

Propylphosphonic anhydride (T3P, 50% solution in ethyl acetate, 448 μL, 0.75 mmol, 4.0 eq.) was added under argon atmosphere at RT to a solution of 2-[4-{5-chloro-2-[4-(trifluoromethyl)-1*H*-1,2,3-triazol-1-yl]phenyl}-5-methoxy-2-oxopyridin-1(2*H*)-yl]butanoic acid (racemate **93**) (86 mg, 0.19 mmol) and 2-methyl-2*H*-indazol-5-amine (42 mg, 0.28 mmol, 1.5 eq.) in pyridine (1.5 mL). The reaction mixture was stirred at 50 °C for 1 h, cooled to RT and purified by preparative RP-HPLC (acetonitrile / 0.1% formic acid in water gradient) to give **64**. Yield: 87 mg (77% of theory). LC/MS (method 1): $t_R$ = 0.95 min, MS (ESIneg): $m/z$ = 584 $[M-H]^-$; $^1H$ NMR (400 MHz, DMSO-$d_6$): δ [ppm] = 10.34 (s, 1H), 9.14 (s, 1H), 8.24 (s, 1H), 8.10 (d, $J$ = 1.2 Hz, 1H), 7.86-7.79 (m, 2H), 7.78 (d, $J$ = 1.7 Hz, 1H), 7.54 (d, $J$ = 9.2 Hz, 1H), 7.26 (dd, $J$ = 9.2 Hz, 2.0 Hz, 1H), 7.19 (s, 1H), 6.52 (s, 1H), 5.63-5.50 (m, 1H), 4.12 (s, 3H), 3.26 (s, 3H), 2.15-1.97 (m, 2H), 0.79 (t, $J$ = 7.2 Hz, 3H).

**(2*S*)-2-[4-{5-Chloro-2-[4-(trifluoromethyl)-1*H*-1,2,3-triazol-1-yl]phenyl}-5-methoxy-2-oxopyridin-1(2*H*)-yl]-*N*-(2-methyl-2*H*-indazol-5-yl)butanamide (eutomer 65).**

Enantiomer separation of 84 mg of 2-[4-{5-chloro-2-[4-(trifluoromethyl)-1*H*-1,2,3-triazol-1-yl]phenyl}-5-methoxy-2-oxopyridin-1(2*H*)-yl]-*N*-(2-methyl-2*H*-indazol-5-yl)butanamide (racemate **64**) gave 29 mg of distomer (chiral HPLC: $t_R$ = 1.38 min) and 31 mg of eutomer **65** (chiral HPLC: $t_R$ = 2.98 min, >99% ee).

LC/MS (method 1): $t_R$ = 0.94 min, MS (ESIpos): $m/z$ = 586 $[M+H]^+$; $^1H$ NMR (400 MHz, DMSO-$d_6$): δ [ppm] = 10.34 (s, 1H), 9.14 (s, 1H), 8.24 (s, 1H), 8.10 (d, *J* = 1.1 Hz, 1H), 7.86-7.79 (m, 2H), 7.78 (d, *J* = 1.8 Hz, 1H), 7.54 (d, *J* = 9.2 Hz, 1H), 7.26 (dd, *J* = 9.2 Hz, 2.0 Hz, 1H), 7.19 (s, 1H), 6.52 (s, 1H), 5.63-5.50 (m, 1H), 4.13 (s, 3H), 3.27 (s, 3H), 2.17-1.96 (m, 2H), 0.80 (t, *J* = 7.2 Hz, 3H).

Separation method: SFC: column: Daicel Chiralpak AD-H 5 µm, 250 mm × 20 mm; mobile phase: 75% carbon dioxide / 25% ethanol; temperature: 40 °C; flow rate: 80 mL/min; UV detection: 210 nm.

Analytical method: SFC: column: Daicel Chiralpak AD-H 3 µm, 100 mm × 4.6 mm; mobile phase: 75% carbon dioxide / 25% ethanol; flow rate: 3 mL/min; UV detection: 210 nm.

**Synthesis of racemate 66.**[a]

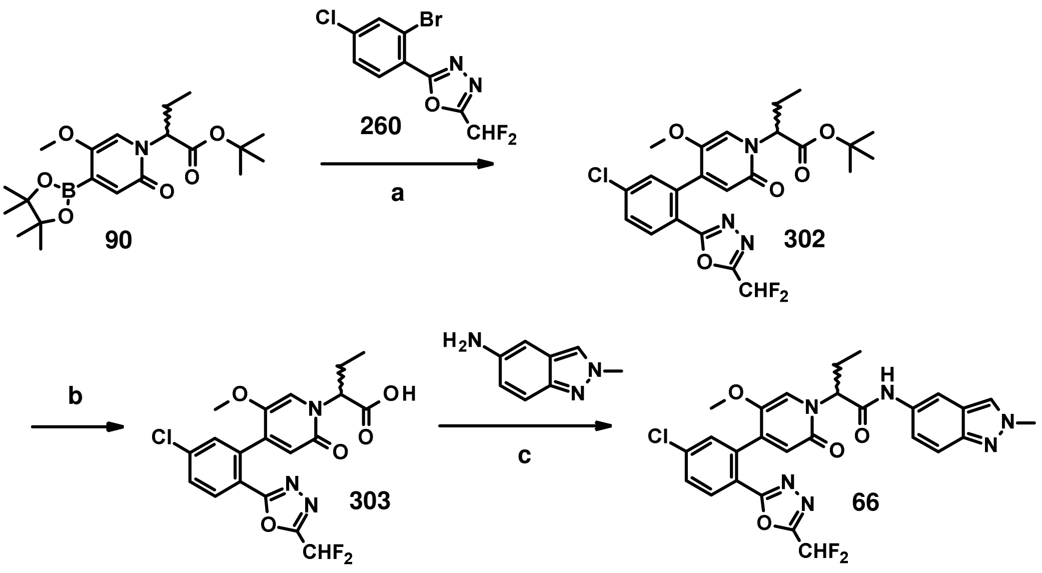


[a]Reagents and conditions: (a) Pd(dppf)$Cl_2$-DCM complex, $K_2CO_3$, 1,4-dioxane, 80 °C, 67%; (b) TFA, DCM, RT, 57%; (c) T3P, pyridine, 50 °C, 54%.

***tert*-Butyl 2-[4-{5-chloro-2-[5-(difluoromethyl)-1,3,4-oxadiazol-2-yl]phenyl}-5-methoxy-2-oxopyridin-1(2*H*)-yl]butanoate (racemate 302).**

A mixture of 2-(2-bromo-4-chlorophenyl)-5-(difluoromethyl)-1,3,4-oxadiazole (**260**) (245 mg, 0.79 mmol, 1.1 eq.), *tert*-butyl 2-[5-methoxy-2-oxo-4-(4,4,5,5-tetramethyl-1,3,2-dioxaborolan-2-yl)pyridin-1(2*H*)-yl]butanoate (racemate **90**) (566 mg, 50% purity, 0.72 mmol) and potassium carbonate

(298 mg, 2.16 mmol, 3.0 eq.) in 1,4-dioxane (7.3 mL) was flushed with argon at RT for 5 min, followed by addition of [1,1'-bis(diphenylphosphino)ferrocene]dichloropalladium-dichloromethane complex (18 mg, 0.22 mmol, 0.03 eq.). The reaction mixture was stirred at 80 °C for 20 h, cooled to RT and filtered through Celite®, the filter residue was washed with dichloromethane and acetonitrile. The combined filtrates were concentrated under reduced pressure. The residue was purified by flash silica gel chromatography (cyclohexane / ethyl acetate gradient) to give **302**. Yield: 245 mg (67% of theory). LC/MS (method 3): $t_R$ = 2.06 min, MS (ESIpos): *m*/*z* = 496 $[M+H]^+$; $^1H$ NMR (400 MHz, DMSO-$d_6$): δ [ppm] = 8.07 (d, *J* = 8.4 Hz, 1H), 7.78 (dd, *J* = 8.4 Hz, 2.2 Hz, 1H), 7.00 (d, *J* = 1.8 Hz, 1H), 7.47 (t, *J* = 51.2 Hz, 1H), 7.16 (br s, 1H), 6.52 (s, 1H), 5.14-4.86 (m, 1H), 3.29 (s, 3H), 2.14-2.03 (m, 2H), 1.41 (s, 9H), 0.84 (t, *J* = 7.4 Hz, 3H).

**2-[4-{5-Chloro-2-[5-(difluoromethyl)-1,3,4-oxadiazol-2-yl]phenyl}-5-methoxy-2-oxopyridin-1(2*H*)-yl]butanoic acid (racemate 303).**

Trifluoroacetic acid (2.10 mL, 27.22 mmol, 50 eq.) was added under argon atmosphere at RT to a solution of *tert*-butyl 2-[4-{5-chloro-2-[5-(difluoromethyl)-1,3,4-oxadiazol-2-yl]phenyl}-5-methoxy-2-oxopyridin-1(2*H*)-yl]butanoate (racemate **302**) (270 mg, 0.54 mmol) in dichloromethane (14.8 mL). The reaction mixture was stirred at RT for 24 h and concentrated under reduced pressure. The residue was purified by RP-HPLC (acetonitrile / 0.1% formic acid in water gradient) to give **303**. Yield: 140 mg (57% of theory). LC/MS (method 5): $t_R$ = 1.09 min, MS (ESIpos): *m*/*z* = 440 $[M+H]^+$; $^1H$ NMR (400 MHz, DMSO-$d_6$): δ [ppm] = 12.95 (br s, 1H), 8.08 (d, *J* = 8.4 Hz, 1H), 7.78 (dd, *J* = 8.4 Hz, 2.2 Hz, 1H), 7.71 (d, *J* = 2.2 Hz, 1H), 7.45 (t, *J* = 51.2 Hz, 1H), 7.20 (br s, 1H), 6.51 (s, 1H), 5.32-4.80 (m, 1H), 3.29 (s, 3H), 2.19-2.04 (m, 2H), 0.83 (t, *J* = 7.4 Hz, 3H).

**2-[4-{5-Chloro-2-[5-(difluoromethyl)-1,3,4-oxadiazol-2-yl]phenyl}-5-methoxy-2-oxopyridin-1(2*H*)-yl]-*N*-(2-methyl-2*H*-indazol-5-yl)butanamide (racemate 66).**

Propylphosphonic anhydride (T3P, 50% solution in ethyl acetate, 54 μL, 0.09 mmol, 4.0 eq.) was added under argon atmosphere at RT to a solution of 2-[4-{5-chloro-2-[5-(difluoromethyl)-1,3,4-oxadiazol-2-yl]phenyl}-5-methoxy-2-oxopyridin-1(2*H*)-yl]butanoic acid (racemate **303**) (10 mg, 0.02 mmol) and 2-methyl-2*H*-indazole-5-amine (5 mg, 0.03 mmol, 1.5 eq.) in pyridine (0.13 mL). The reaction mixture was stirred at 50 °C for 23 h, cooled to RT and purified by preparative RP-HPLC (acetonitrile / 0.1% formic acid in water gradient) to give **66**. Yield: 7 mg (54% of theory). LC/MS (method 5): $t_R$ = 1.22 min, MS (ESIneg): *m*/*z* = 567 $[M-H]^-$; $^1H$ NMR (400 MHz, DMSO-$d_6$): δ [ppm] = 10.4 (s, 1H), 8.25 (s, 1H), 8.13 (s, 1H), 8.08 (d, *J* = 8.0 Hz, 1H), 7.78 (dd, *J* = 8.4 Hz, 2.1 Hz, 1H), 7.71 (d, *J* = 2.1 Hz, 1H), 7.55 (d, *J* = 9.2 Hz, 1H), 7.47 (t, *J* = 51.7 Hz, 1H), 7.35 (s, 1H), 7.29 (dd, *J* = 9.3 Hz, 1.8 Hz, 1H), 6.56 (s, 1H), 5.73-5.61 (m, 1H), 4.13 (s, 3H), 3.34 (s, 3H), 2.22-1.98 (m, 2H), 0.91 (t, *J* = 7.3 Hz, 3H).

**Synthesis of racemate 67.**[a]

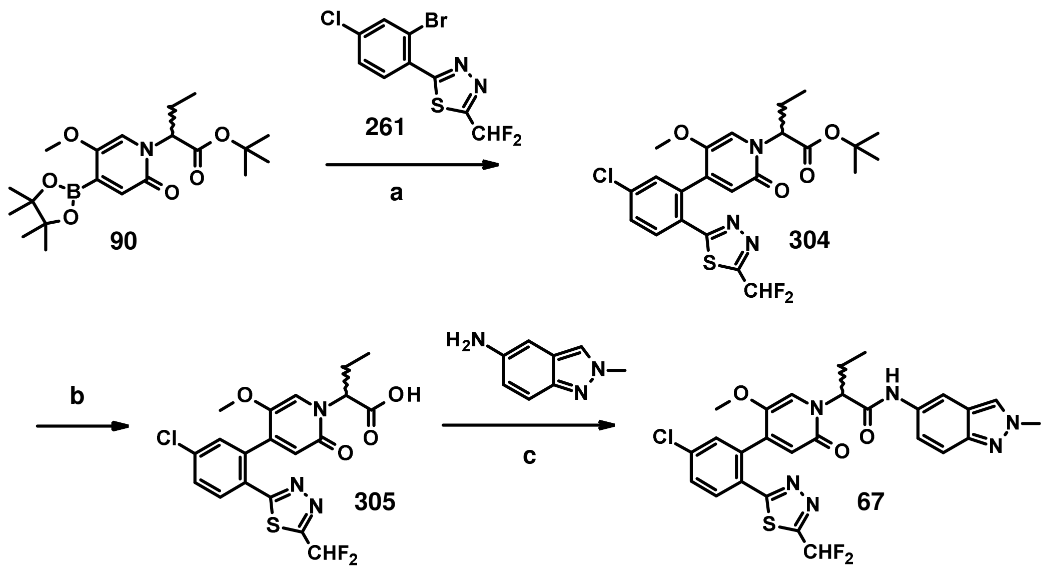

[a]Reagents and conditions: (a) Pd(dppf)$Cl_2$-DCM complex, $K_2CO_3$, 1,4-dioxane, 80 °C, 63%; (b) TFA, DCM, RT, 91%; (c) T3P, pyridine, 40 °C, 78%.

***tert*-Butyl 2-[4-{5-chloro-2-[5-(difluoromethyl)-1,3,4-thiadiazol-2-yl]phenyl}-5-methoxy-2-oxopyridin-1(2*H*)-yl]butanoate (racemate 304).**

A mixture of 2-(2-bromo-4-chlorophenyl)-5-(difluoromethyl)-1,3,4-thiadiazole (**261**) (300 mg, 0.92 mmol), *tert*-butyl 2-[5-methoxy-2-oxo-4-(4,4,5,5-tetramethyl-1,3,2-dioxaborolan-2-yl)pyridin-1(2*H*)-yl]butanoate (racemate **90**) (997 mg, 40% purity, 1.01 mmol, 1.1 eq.) and potassium carbonate (382 mg, 2.76 mmol, 3.0 eq.) in 1,4-dioxane (10 mL) was flushed with argon at RT for 20 min, followed by addition of [1,1'-bis(diphenylphosphino)ferrocene]dichloropalladium-dichloromethane complex (23 mg, 0.28 mmol, 0.03 eq.). The reaction mixture was stirred at 80 °C for 18 h, cooled to RT, filtered through Celite®, and the filter residue was washed with dichloromethane and acetonitrile. The combined filtrates were concentrated under reduced pressure. The residue was purified by flash silica gel chromatography (cyclohexane / ethyl acetate gradient) and subsequently by preparative RP-HPLC (acetonitrile / water gradient) to give **304**. Yield: 423 mg (70% purity, 63% of theory). LC/MS (method 3): $t_R$ = 2.12 min, MS (ESIpos): *m*/*z* = 512 $[M+H]^+$; $^1H$ NMR (400 MHz, DMSO-$d_6$): δ [ppm] = 8.08 (d, *J* = 8.4 Hz, 1H), 7.74 (dd, *J* = 8.4 Hz, 2.3 Hz, 1H), 7.66 (d, *J* = 2.2 Hz, 1H), 7.58 (t, *J* = 53.1 Hz, 1H), 7.22 (br s, 1H), 6.54 (s, 1H), 4.99-4.93 (m, 1H), 3.29 (s, 3H), 2.14-2.03 (m, 2H), 1.41 (s, 9H), 0.82 (t, *J* = 7.4 Hz, 3H).

**2-[4-{5-Chloro-2-[5-(difluoromethyl)-1,3,4-thiadiazol-2-yl]phenyl}-5-methoxy-2-oxopyridin-1(2*H*)-yl]butanoic acid (racemate 305).**

Trifluoroacetic acid (1.32 mL, 17.09 mmol, 50 eq.) was added under argon atmosphere at RT to a solution of *tert*-butyl 2-[4-{5-chloro-2-[5-(difluoromethyl)-1,3,4-thiadiazol-2-yl]phenyl}-5-methoxy-2-

oxopyridin-1(2*H*)-yl]butanoate (racemate **304**) (250 mg, 0.34 mmol) in dichloromethane (9.8 mL). The reaction mixture was stirred at RT for 7 h and concentrated under reduced pressure. The residue was purified by preparative RP-HPLC (acetonitrile / 0.1% formic acid in water gradient) to give **305**. Yield: 142 mg (91% of theory). LC/MS (method 3): $t_R$ = 1.57 min, MS (ESIpos): *m*/*z* = 456 $[M+H]^+$; $^1$H NMR (400 MHz, DMSO-$d_6$): δ [ppm] = 12.96 (br s, 1H), 8.07 (d, *J* = 8.4 Hz, 1H), 7.73 (dd, *J* = 8.4 Hz, 2.2 Hz, 1H), 7.67 (d, *J* = 2.2 Hz, 1H), 7.58 (t, *J* = 53.1 Hz, 1H), 7.26 (br s, 1H), 6.53 (s, 1H), 5.37-4.76 (m, 1H), 3.29 (s, 3H), 2.19-2.06 (m, 2H), 0.80 (t, *J* = 7.3 Hz, 3H).

**2-[4-{5-Chloro-2-[5-(difluoromethyl)-1,3,4-thiadiazol-2-yl]phenyl}-5-methoxy-2-oxopyridin-1(2*H*)-yl]-*N*-(2-methyl-2*H*-indazol-5-yl)butanamide (racemate 67).**

Propylphosphonic anhydride (T3P, 50% solution in ethyl acetate, 120 µL, 0.20 mmol, 4.0 eq.) was added under argon atmosphere at RT to a solution of 2-[4-{5-chloro-2-[5-(difluoromethyl)-1,3,4-thiadiazol-2-yl]phenyl}-5-methoxy-2-oxopyridin-1(2*H*)-yl]butanoic acid (racemate **305**) (23 mg, 0.05 mmol) and 2-methyl-2*H*-indazole-5-amine (11 mg, 0.08 mmol, 1.5 eq.) in pyridine (0.25 mL). The reaction mixture was stirred at 40 °C for 25 min, cooled to RT and purified by preparative RP-HPLC (acetonitrile / water gradient) to give **67**. Yield: 23 mg (78% of theory). LC/MS (method 3): $t_R$ = 1.75 min, MS (ESIpos): *m*/*z* = 585 $[M+H]^+$; $^1$H NMR (400 MHz, DMSO-$d_6$): δ [ppm] = 10.38 (br s, 1H), 8.25 (s, 1H), 8.13 (d, *J* = 1.5 Hz, 1H), 8.08 (d, *J* = 8.3 Hz, 1H), 7.74 (dd, *J* = 8.4 Hz, 2.2 Hz, 1H), 7.67 (d, *J* = 2.1 Hz, 1H), 7.58 (t, *J* = 53.2 Hz, 1H), 7.55 (d, *J* = 9.1 Hz, 1H), 7.37 (s, 1H), 7.28 (dd, *J* = 9.2 Hz, 1.9 Hz, 1H), 6.57 (s, 1H), 5.73-5.61 (m, 1H), 4.13 (s, 3H), 3.34 (s, 3H), 2.22-2.05 (m, 2H), 0.88 (t, *J* = 7.2 Hz, 3H).

**Synthesis of racemate 68.**[a]

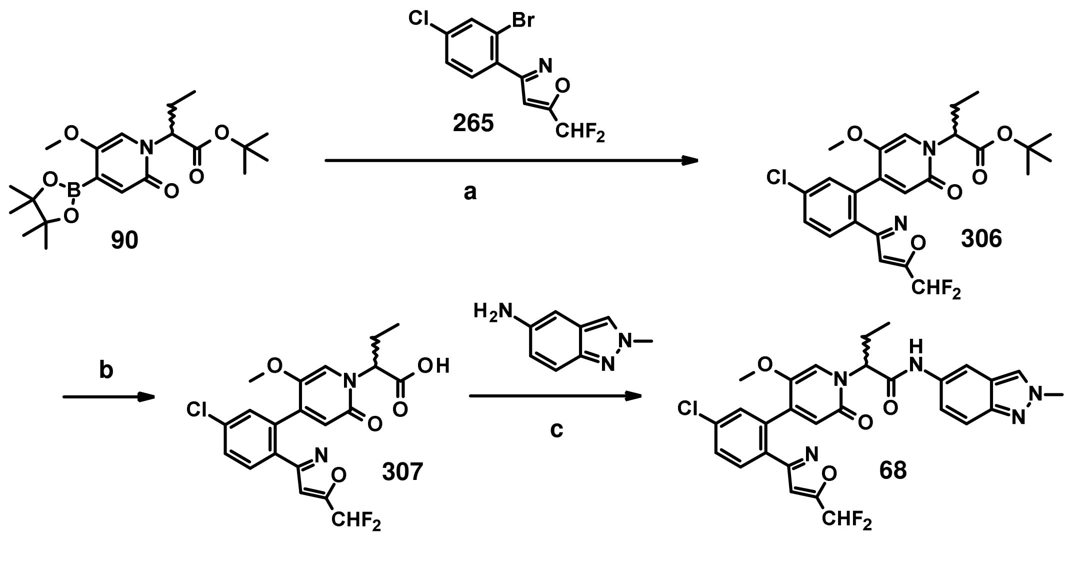


[a]Reagents and conditions: (a) Pd(dppf)$Cl_2$-DCM complex, $K_2CO_3$, 1,4-dioxane, 80 °C, 61%; (b) TFA, DCM, RT, 87%; (c) T3P, pyridine, 50 °C, 90%.

***tert*-Butyl 2-[4-{5-chloro-2-[5-(difluoromethyl)-1,2-oxazol-3-yl]phenyl}-5-methoxy-2-oxopyridin-1(2*H*)-yl]butanoate (racemate 306).**

A mixture of 3-(2-bromo-4-chlorophenyl)-5-(difluoromethyl)-1,2-oxazole (**265**) (300 mg, 0.97 mmol), *tert*-butyl 2-[5-methoxy-2-oxo-4-(4,4,5,5-tetramethyl-1,3,2-dioxaborolan-2-yl)pyridin-1(2*H*)-yl]butanoate (racemate **90**) (421 mg, 1.07 mmol, 1.1 eq.) and potassium carbonate (403 mg, 2.92 mmol, 3.0 eq.) in 1,4-dioxane (10 mL) was flushed with argon at RT for 20 min, followed by addition of [1,1'-bis(diphenylphosphino)ferrocene]dichloropalladium-dichloromethane complex (24 mg, 0.29 mmol, 0.03 eq.). The reaction mixture was stirred at 80 °C for 18 h, filtered through Celite®, and the filter residue was washed with dichloromethane and acetonitrile. The combined filtrates were concentrated under reduced pressure. The residue was purified by flash silica gel chromatography (cyclohexane / ethyl acetate gradient) to give **306**. Yield: 292 mg (61% of theory). LC/MS (method 3): $t_R$ = 2.16 min, MS (ESIpos): *m*/*z* = 495 $[M+H]^+$.

**2-[4-{5-Chloro-2-[5-(difluoromethyl)-1,2-oxazol-3-yl]phenyl}-5-methoxy-2-oxopyridin-1(2*H*)-yl]butanoic acid (racemate 307).**

Trifluoroacetic acid (1.56 mL, 20.21 mmol, 50 eq.) was added at RT to a solution of *tert*-butyl 2-[4-{5-chloro-2-[5-(difluoromethyl)-1,2-oxazol-3-yl]phenyl}-5-methoxy-2-oxopyridin-1(2*H*)-yl]butanoate (racemate **306**) (200 mg, 0.40 mmol) in dichloromethane (12 mL). The reaction mixture was stirred at RT for 24 h and concentrated under reduced pressure. The residue was purified by preparative RP-HPLC (acetonitrile / 0.1% formic acid in water gradient) to give **307**. Yield: 154 mg (87% of theory). LC/MS (method 3): $t_R$ = 1.65 min, MS (ESIpos): *m*/*z* = 439 $[M+H]^+$; $^1$H NMR (400 MHz, DMSO-$d_6$): δ [ppm] = 12.9 (br s, 1H), 7.77 (d, *J* = 8.3 Hz, 1H), 7.68 (dd, *J* = 8.3 Hz, 2.2 Hz, 1H), 7.60 (d, *J* = 2.1 Hz, 1H), 7.31 (t, *J* = 52.7 Hz, 1H), 7.18 (s, 1H), 6.89 (s, 1H), 6.41 (s, 1H), 5.18-4.96 (m, 1H), 3.29 (s, 3H), 2.15-2.06 (m, 2H), 0.78 (t, *J* = 7.4 Hz, 3H).

**2-[4-{5-Chloro-2-[5-(difluoromethyl)-1,2-oxazol-3-yl]phenyl}-5-methoxy-2-oxopyridin-1(2*H*)-yl]-*N*-(2-methyl-2*H*-indazol-5-yl)butanamide (racemate 68).**

Propylphosphonic anhydride (T3P, 50% solution in ethyl acetate, 54 µL, 0.09 mmol, 4.0 eq.) was added under argon atmosphere at RT to a solution of 2-[4-{5-chloro-2-[5-(difluoromethyl)-1,2-oxazol-3-yl]phenyl}-5-methoxy-2-oxopyridin-1(2*H*)-yl]butanoic acid (racemate **307**) (10 mg, 0.02 mmol) and 2-methyl-2*H*-indazole-5-amine (5 mg, 0.03 mmol, 1.5 eq.) in pyridine (0.13 mL). The reaction mixture was stirred at 50 °C for 50 min, cooled to RT and purified by preparative RP-HPLC (acetonitrile / 0.1% formic acid in water gradient) to give **68**. Yield: 12 mg (90% of theory). LC/MS (method 3): $t_R$ = 1.81 min, MS (ESIpos): *m*/*z* = 568 $[M+H]^+$; $^1$H NMR (400 MHz, DMSO-$d_6$): δ [ppm] = 10.37 (br s, 1H), 8.25 (s, 1H), 8.13 (d, *J* = 1.2 Hz, 1H), 7.78 (d, *J* = 8.3 Hz, 1H), 7.68 (dd, *J* = 8.3 Hz, 2.2 Hz, 1H), 7.59 (d, *J* = 2.2 Hz, 1H), 7.55 (d, *J* = 9.3 Hz, 1H), 7.32 (t, *J* = 52.8 Hz, 1H, partially concealed), 7.32-7.26 (m, 2H, partially concealed), 6.98-6.94 (m, 1H), 6.45 (s, 1H), 5.68-5.60 (m, 1H), 4.13 (s, 3H), 3.34

(s, 3H), 2.20-2.00 (m, 2H), 0.87 (t, $J$ = 7.2 Hz, 3H).

**Synthesis of racemate 69.**[a]

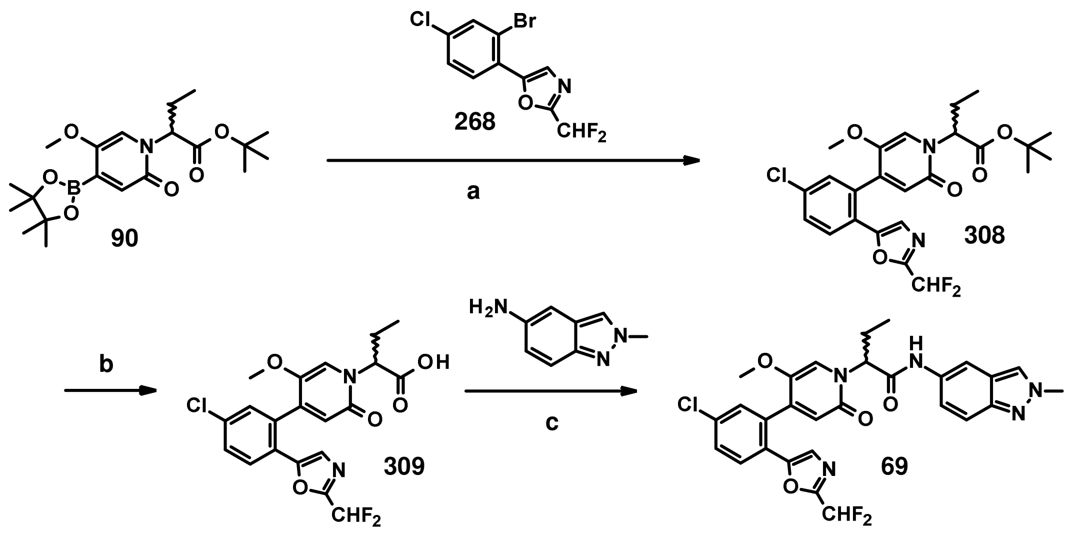

[a]Reagents and conditions: (a) Pd(dppf)$Cl_2$-DCM complex, $K_2CO_3$, 1,4-dioxane, 100 °C/microwave, 79%; (b) HCl/1,4-dioxane, RT, 99%; (c) T3P, pyridine, 50 °C, 79%.

***tert*-Butyl 2-[4-{5-chloro-2-[2-(difluoromethyl)-1,3-oxazol-5-yl]phenyl}-5-methoxy-2-oxopyridin-1(2*H*)-yl]butanoate (racemate 308).**

A mixture of *tert*-butyl 2-[5-methoxy-2-oxo-4-(4,4,5,5-tetramethyl-1,3,2-dioxaborolan-2-yl)pyridin-1(2*H*)-yl]butanoate (racemate **90**) (940 mg, 60% purity, 1.43 mmol), 5-(2-bromo-4-chlorophenyl)-2-(difluoromethyl)-1,3-oxazole (**268**) (531 mg, 1.72 mmol, 1.2 eq.) and potassium carbonate (595 mg, 4.30 mmol) in 1,4-dioxane (14 mL) was flushed with argon at RT for 5 min, followed by addition of [1,1-bis-(diphenylphosphino)-ferrocene]dichloropalladium dichloromethane complex (70 mg, 0.09 mmol, 0.06 eq.). The reaction mixture was stirred in the microwave at 100 °C for 2 h, cooled to RT, filtered through Celite®, and the filter residue was washed with ethyl acetate. The combined filtrates were washed with brine. After phase separation, the organic phase was dried over anhydrous sodium sulfate, filtered and concentrated under reduced pressure. The residue was purified by flash silica gel chromatography (cyclohexane / ethyl acetate gradient) to give **308**. Yield: 570 mg (79% of theory). LC/MS (method 1): $t_R$ = 1.15 min, MS (ESIpos): $m/z$ = 495 $[M+H]^+$.

**2-[4-{5-Chloro-2-[2-(difluoromethyl)-1,3-oxazol-5-yl]phenyl}-5-methoxy-2-oxopyridin-1(2*H*)-yl]butanoic acid (racemate 309).**

A mixture of *tert*-butyl 2-[4-{5-chloro-2-[2-(difluoromethyl)-1,3-oxazol-5-yl]phenyl}-5-methoxy-2-oxopyridin-1(2*H*)-yl]butanoate (racemate **308**) (570 mg, 1.13 mmol) in hydrogen chloride solution (4 M in 1,4-dioxane, 11 mL) was stirred at RT overnight and concentrated under reduced pressure. The

residue was dried *in vacuo* to give **309** which was used without further purification. Yield: 489 mg (99% of theory). LC/MS (method 3): $t_R$ = 1.61 min, MS (ESIpos): *m*/*z* = 439 $[M+H]^+$.

**2-[4-{5-Chloro-2-[2-(difluoromethyl)-1,3-oxazol-5-yl]phenyl}-5-methoxy-2-oxopyridin-1(2*H*)-yl]-*N*-(2-methyl-2*H*-indazol-5-yl)butanamide (racemate 69).**

Propylphosphonic anhydride (T3P, 50% solution in ethyl acetate, 203 µL, 0.34 mmol, 3.0 eq.) was added under argon atmosphere at RT to a solution of 2-[4-{5-chloro-2-[2-(difluoromethyl)-1,3-oxazol-5-yl]phenyl}-5-methoxy-2-oxopyridin-1(2*H*)-yl]butanoic acid (racemate **309**) (50 mg, 0.11 mmol) and 2-methyl-2*H*-indazole-5-amine (25 mg, 0.17 mmol, 1.5 eq.) in pyridine (1 mL). The reaction mixture was stirred at 50 °C for 1 h, cooled to RT and purified by preparative RP-HPLC (acetonitrile / 0.1% formic acid in water gradient) to give **69**. Yield: 51 mg (79% of theory). LC/MS (method 3): $t_R$ = 1.76 min, MS (ESIpos): *m*/*z* = 568 $[M+H]^+$; $^1$H NMR (400 MHz, DMSO-$d_6$): δ [ppm] = 10.39 (br s, 1H), 8.25 (s, 1H), 8.14 (d, *J* = 1.2 Hz, 1H), 7.83 (d, *J* = 8.4 Hz, 1H), 7.67 (dd, *J* = 8.4 Hz, 2.2 Hz, 1H), 7.59-7.52 (m, 2H), 7.39 (s, 1H), 7.29 (dd, *J* = 9.3 Hz, 2.0 Hz, 1H), 7.17 (t, *J* = 52.0 Hz, 1H), 6.46 (s, 1H), 5.72-5.63 (m, 1H), 4.13 (s, 3H), 3.41 (s, 3H), 2.24-2.02 (m, 2H), 0.92 (t, *J* = 7.3 Hz, 3H).

**Synthesis of racemate 70.**[a]

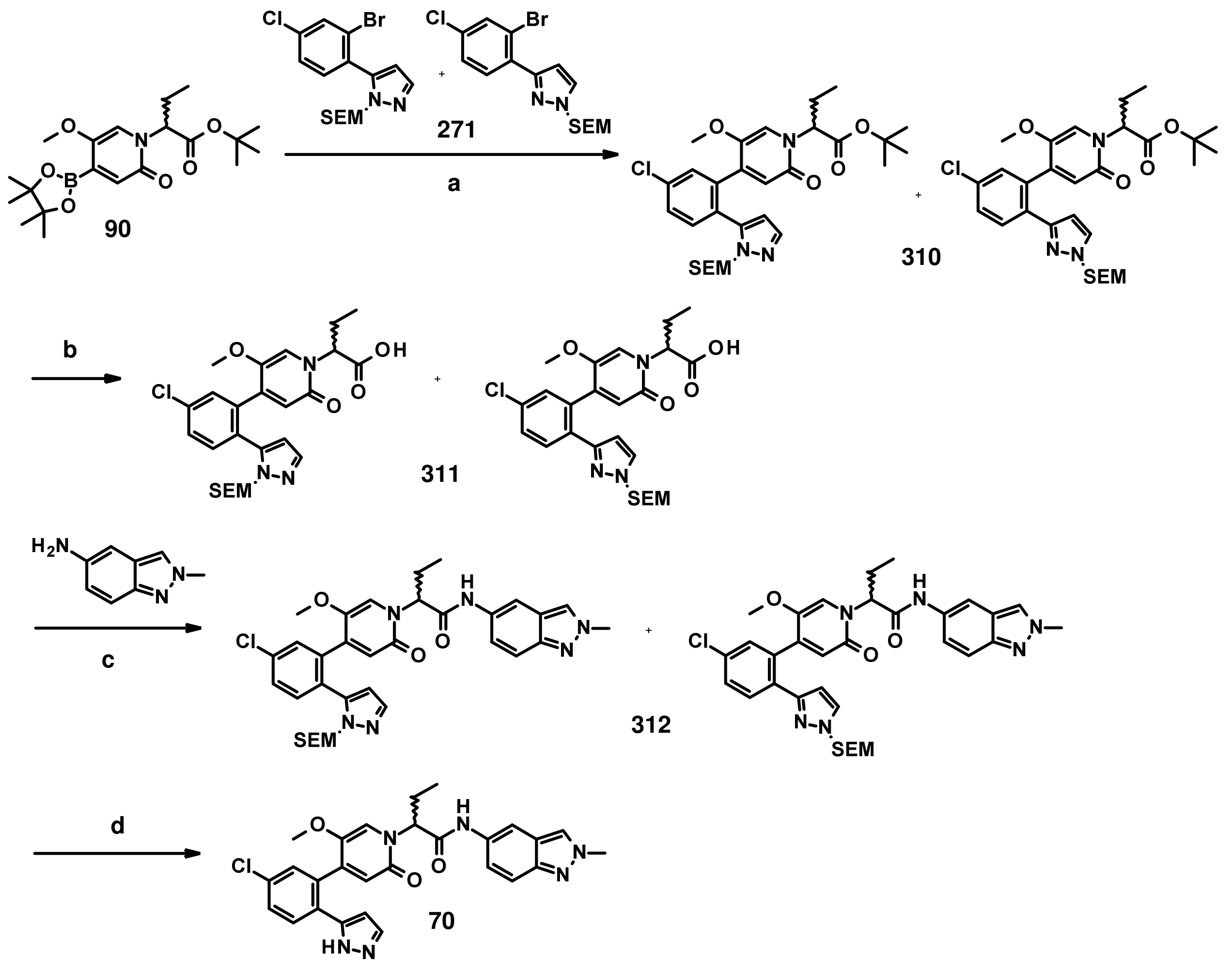


[a]Reagents and conditions: (a) Pd(dppf)$Cl_2$-DCM complex, $K_2CO_3$, 1,4-dioxane, 80 °C, 83%; (b) LiOH, EtOH/THF, RT, 99%; (c) T3P, pyridine, 50 °C, 59%.

**Mixture of *tert*-butyl 2-{4-[5-chloro-2-(1-{[2-(trimethylsilyl)ethoxy]methyl}-1*H*-pyrazol-5-yl)phenyl]-5-methoxy-2-oxopyridin-1(2*H*)-yl}butanoate and *tert*-butyl 2-{4-[5-chloro-2-(1-{[2-(trimethylsilyl)ethoxy]methyl}-1*H*-pyrazol-3-yl)phenyl]-5-methoxy-2-oxopyridin-1(2*H*)-yl}butanoate (racemic mixture 310).**

A mixture of *tert*-butyl 2-[5-methoxy-2-oxo-4-(4,4,5,5-tetramethyl-1,3,2-dioxaborolan-2-yl)pyridin-1(2*H*)-yl]butanoate (racemate **90**) (648 mg, 50% purity, 0.82 mmol), a mixture of 5-(2-bromo-4-chlorophenyl)-1-{[2-(trimethylsilyl)ethoxy]methyl}-1*H*-pyrazole and 3-(2-bromo-4-chlorophenyl)-1-{[2-(trimethylsilyl)ethoxy]methyl}-1*H*-pyrazole (**271**) (351 mg, 0.91 mmol, 1.1 eq.) and potassium carbonate (342 mg, 2.47 mmol, 3.0 eq.) in 1,4-dioxane (8.4 mL) was flushed with argon at RT for 5 min, followed by addition of [1,1-bis-(diphenylphosphino)-ferrocene]dichloropalladium dichloromethane complex (20 mg, 0.03 mmol, 0.03 eq.). The reaction mixture was stirred at 80 °C for 12 h, cooled to RT, filtered through Celite®, and the filter residue was washed with acetonitrile and dichloromethane. The

combined filtrates were concentrated under reduced pressure. The residue was purified by flash silica gel chromatography (cyclohexane / ethyl acetate gradient) to give **310**. Yield: 393 mg (83% of theory). LC/MS (method 3): $t_R$ = 2.56 min / 2.63 min, MS (ESIpos): *m/z* = 574 $[M+H]^+$.

**Mixture of 2-{4-[5-chloro-2-(1-{[2-(trimethylsilyl)ethoxy]methyl}-1*H*-pyrazol-5-yl)phenyl]-5-methoxy-2-oxopyridin-1(2*H*)-yl}butanoic acid and 2-{4-[5-chloro-2-(1-{[2-(trimethylsilyl)ethoxy]methyl}-1*H*-pyrazol-3-yl)phenyl]-5-methoxy-2-oxopyridin-1(2*H*)-yl}butanoic acid (racemic mixture 311).**

Lithium hydroxide (68 mg, 2.84 mmol, 5.0 eq.) was added to a suspension of a mixture of *tert*-butyl 2-{4-[5-chloro-2-(1-{[2-(trimethylsilyl)ethoxy]methyl}-1*H*-pyrazol-5-yl)phenyl]-5-methoxy-2-oxopyridin-1(2*H*)-yl}butanoate and *tert*-butyl 2-{4-[5-chloro-2-(1-{[2-(trimethylsilyl)ethoxy]methyl}-1*H*-pyrazol-3-yl)phenyl]-5-methoxy-2-oxopyridin-1(2*H*)-yl}butanoate (racemic mixture **310**) (358 mg, 0.57 mmol) in a mixture of ethanol (40 mL) and tetrahydrofuran (20 mL). The reaction mixture was stirred at RT for 20 h and mixed with ethyl acetate and pH 5 buffer. After phase separation, the organic phase was dried over anhydrous sodium sulfate, filtered and concentrated under reduced pressure to give **311** which was used without further purification. Yield: 290 mg (99% of theory). LC/MS (method 3): $t_R$ = 2.09 min / 2.16 min, MS (ESIpos): *m/z* = 518 $[M+H]^+$.

**Mixture of 2-{4-[5-chloro-2-(1-{[2-(trimethylsilyl)ethoxy]methyl}-1*H*-pyrazol-5-yl)phenyl]-5-methoxy-2-oxopyridin-1(2*H*)-yl}-*N*-(2-methyl-2*H*-indazol-5-yl)butanamide and 2-{4-[5-chloro-2-(1-{[2-(trimethylsilyl)ethoxy]methyl}-1*H*-pyrazol-3-yl)phenyl]-5-methoxy-2-oxopyridin-1(2*H*)-yl}-*N*-(2-methyl-2*H*-indazol-5-yl)butanamide (racemic mixture 312).**

Propylphosphonic anhydride (T3P, 50% solution in ethyl acetate, 211 µL, 0.36 mmol, 4.0 eq.) was added under argon atmosphere at RT to a solution of a mixture of 2-{4-[5-chloro-2-(1-{[2-(trimethylsilyl)ethoxy]methyl}-1*H*-pyrazol-5-yl)phenyl]-5-methoxy-2-oxopyridin-1(2*H*)-yl}butanoic acid and 2-{4-[5-chloro-2-(1-{[2-(trimethylsilyl)ethoxy]methyl}-1*H*-pyrazol-3-yl)phenyl]-5-methoxy-2-oxopyridin-1(2*H*)-yl}butanoic acid (racemic mixture **311**) (46 mg, 0.09 mmol) and 2-methyl-2*H*-indazole-5-amine (20 mg, 0.13 mmol, 1.5 eq.) in pyridine (0.5 mL). The reaction mixture was stirred at 50 °C for 23 h, cooled to RT and purified by preparative RP-HPLC (acetonitrile / 0.1% formic acid in water gradient) to give **312**. Yield: 34 mg (59% of theory). LC/MS (method 3): $t_R$ = 2.23 min / 2.30 min, MS (ESIpos): *m/z* = 647 $[M+H]^+$.

**2-{4-[5-Chloro-2-(1*H*-pyrazol-5-yl)phenyl]-5-methoxy-2-oxopyridin-1(2*H*)-yl}-*N*-(2-methyl-2*H*-indazol-5-yl)butanamide (racemate 70).**

Trifluoroacetic acid (67 µL, 0.87 mmol, 20 eq.) was added at RT to a solution of a mixture of 2-{4-[5-chloro-2-(1-{[2-(trimethylsilyl)ethoxy]methyl}-1*H*-pyrazol-5-yl)phenyl]-5-methoxy-2-oxopyridin-1(2*H*)-yl}-*N*-(2-methyl-2*H*-indazol-5-yl)butanamide and 2-{4-[5-chloro-2-(1-{[2-(trimethylsilyl)ethoxy]methyl}-1*H*-pyrazol-3-yl)phenyl]-5-methoxy-2-oxopyridin-1(2*H*)-yl}-*N*-(2-

methyl-2*H*-indazol-5-yl)butanamide (racemic mixture **312**) (28 mg, 0.04 mmol) in dichloromethane (2.4 mL). The reaction mixture was stirred at RT for 3 h and purified by preparative RP-HPLC (acetonitrile / 0.1% formic acid in water gradient) to give **70**. Yield: 2 mg (9% of theory). LC/MS (method 5): $t_R$ = 1.10 min, MS (ESIpos): *m*/*z* = 517 $[M+H]^+$; $^1H$ NMR (400 MHz, DMSO-$d_6$): δ [ppm] = 13.11 / 12.86 (2x br s, 1H), 10.39 (br s, 1H), 8.25 (s, 1H), 8.14 (s, 1H), 7.80 (d, *J* = 8.4 Hz, 1H), 7.67-7.41 (m, 3H), 7.35 (d, *J* = 8.7 Hz, 2H), 7.29 (dd, *J* = 9.2 Hz, 1.9 Hz, 1H), 6.40-6.25 (m, 1H), 6.06-5.85 (m, 1H), 5.66 (dd, *J* = 9.4 Hz, 6.0 Hz, 1H), 4.13 (s, 3H), 3.37 (s, 3H), 2.20-2.00 (m, 2H), 0.90 (t, *J* = 7.1 Hz, 3H).

**Compounds of Table 6.**

**Synthesis of compound 71.**[a]

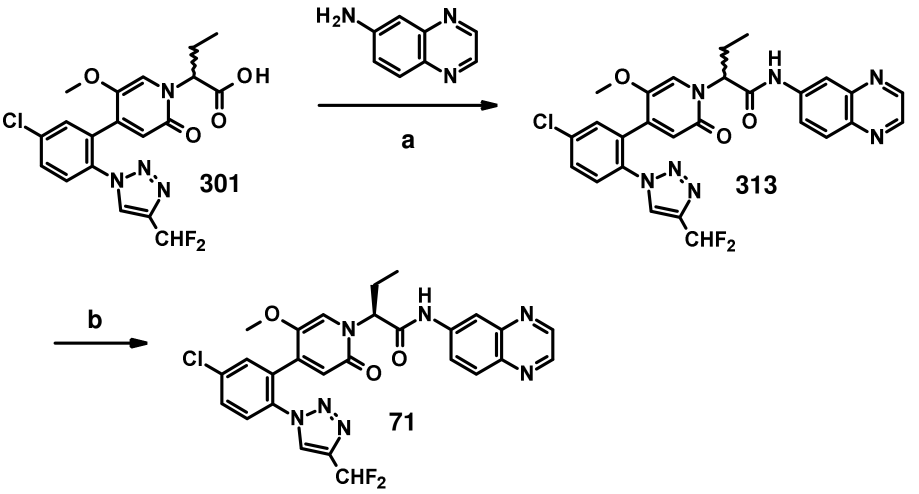


[a]Reagents and conditions: (a) T3P, pyridine, 50 °C, 73%; (b) enantiomer separation.

***N*-(Quinoxalin-6-yl)-2-[4-{5-chloro-2-[4-(difluoromethyl)-1*H*-1,2,3-triazol-1-yl]phenyl}-5-methoxy-2-oxopyridin-1(2*H*)-yl]butanamide (racemate 313).**

Propylphosphonic anhydride (T3P, 50% solution in ethyl acetate, 662 µL, 1.11 mmol, 4.0 eq.) was added under argon atmosphere at RT to a solution of 2-[4-{5-chloro-2-[4-(difluoromethyl)-1*H*-1,2,3-triazol-1-yl]phenyl}-5-methoxy-2-oxopyridin-1(2*H*)-yl]butanoic acid (racemate **301**) (122 mg, 0.28 mmol) in pyridine (2.3 mL). The reaction mixture was heated to 50 °C, and quinoxaline-6-amine (61 mg, 0.42 mmol, 1.5 eq.) was added. The reaction mixture was stirred at 50 °C for additional 1 h, cooled to RT and purified without further work-up by preparative RP-HPLC (acetonitrile / 0.1% formic acid in water gradient) to give **313**. Yield: 115 mg (73% of theory). LC/MS (method 1): $t_R$ = 0.89 min, MS (ESIpos): *m*/*z* = 566 $[M+H]^+$; $^1H$ NMR (400 MHz, DMSO-$d_6$): δ [ppm] = 10.93 (s, 1H), 8.92-8.81 (m, 2H), 8.74 (s, 1H), 8.51 (d, *J* = 2.3 Hz, 1H), 8.07 (d, *J* = 9.1 Hz, 1H), 7.96 (dd, *J* = 9.2 Hz, 2.3 Hz, 1H), 7.83-7.72 (m, 3H), 7.22 (t, *J* = 54.0 Hz, 1H), 7.20 (s, 1H), 6.53 (s, 1H), 5.67-5.58 (m, 1H), 3.28 (s, 3H),

2.25-2.04 (m, 2H), 0.84 (t, $J$ = 7.2 Hz, 3H).

**$N$-(Quinoxalin-6-yl)-(2$S$)-2-[4-{5-chloro-2-[4-(difluoromethyl)-1$H$-1,2,3-triazol-1-yl]phenyl}-5-methoxy-2-oxopyridin-1(2$H$)-yl]butanamide (eutomer 71).**

Enantiomer separation of 115 mg of $N$-(quinoxalin-6-yl)-2-[4-{5-chloro-2-[4-(difluoromethyl)-1$H$-1,2,3-triazol-1-yl]phenyl}-5-methoxy-2-oxopyridin-1(2$H$)-yl]butanamide (racemate **313**) gave 46 mg of distomer (chiral HPLC: $t_R$ = 2.61 min) and 42 mg of eutomer **71** (chiral HPLC: $t_R$ = 4.60 min, >99% ee).

LC/MS (method 3): $t_R$ = 1.68 min, MS (ESIpos): $m/z$ = 566 $[M+H]^+$; $^1$H NMR (400 MHz, DMSO-$d_6$): δ [ppm] = 10.92 (s, 1H), 8.89 (d, $J$ = 2.0 Hz, 1H), 8.84 (d, $J$ = 1.8 Hz, 1H), 8.74 (s, 1H), 8.51 (d, $J$ = 2.2 Hz, 1H), 8.07 (d, $J$ = 9.2 Hz, 1H), 7.96 (dd, $J$ = 9.2 Hz, 2.2 Hz, 1H), 7.83-7.72 (m, 3H), 7.23 (t, $J$ = 54.0 Hz, 1H), 7.20 (s, 1H), 6.53 (s, 1H), 5.67-5.58 (m, 1H), 3.28 (s, 3H), 2.25-2.04 (m, 2H), 0.84 (t, $J$ = 7.2 Hz, 3H).

Separation method: SFC: column: Daicel Chiralpak AD-H 5 $\mu$m, 250 mm × 20 mm; mobile phase: 70% carbon dioxide / 30% ethanol; temperature: 40 °C; flow rate: 80 mL/min; UV detection: 210 nm.

Analytical method: SFC: column: Daicel Chiralpak AD-H 3 $\mu$m, 100 mm × 4.6 mm; mobile phase: 70% carbon dioxide / 30% methanol; temperature: 40 °C; flow rate: 3 mL/min; UV detection: 210 nm.

**Synthesis of compound 72.**[a]

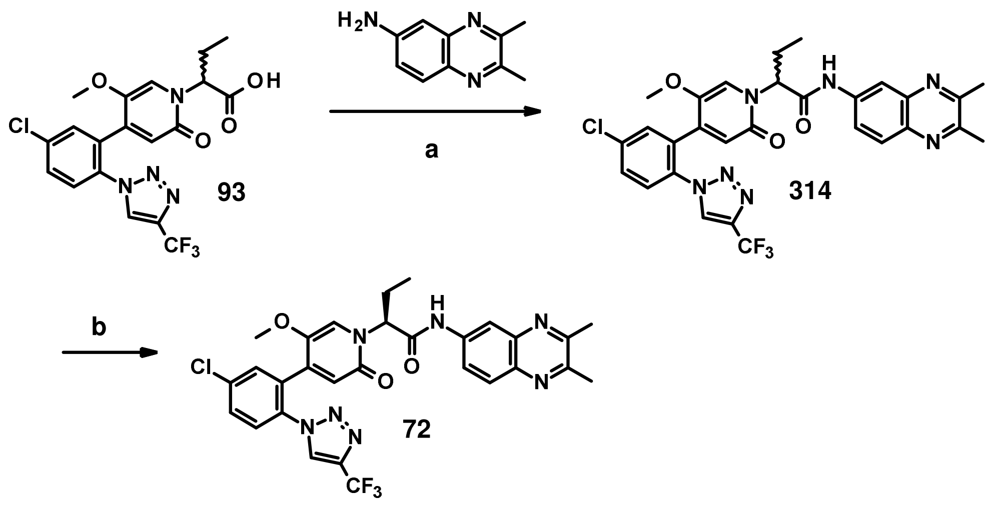


[a]Reagents and conditions: (a) T3P, pyridine, 50 °C, 87%; (b) enantiomer separation.

**2-[4-{5-Chloro-2-[4-(trifluoromethyl)-1$H$-1,2,3-triazol-1-yl]phenyl}-5-methoxy-2-oxopyridin-1(2$H$)-yl]-$N$-(2,3-dimethylquinoxalin-6-yl)butanamide (racemate 314).**

Propylphosphonic anhydride (T3P, 50% solution in ethyl acetate, 448 $\mu$L, 0.75 mmol, 4.0 eq.) was added under argon atmosphere at RT to a solution of 2-[4-{5-chloro-2-[4-(trifluoromethyl)-1$H$-1,2,3-triazol-1-yl]phenyl}-5-methoxy-2-oxopyridin-1(2$H$)-yl]butanoic acid (racemate **93**) (86 mg, 0.19 mmol) and

2,3-dimethylquinoxalin-6-amine (49 mg, 0.28 mmol, 1.5 eq.) in pyridine (1.5 mL). The reaction mixture was stirred at 50 °C for 1 h, cooled to RT and purified without further work-up by preparative RP-HPLC (acetonitrile / 0.1% formic acid in water gradient) to give **314**. Yield: 100 mg (87% of theory). LC/MS (method 1): $t_R$ = 1.02 min, MS (ESIpos): *m/z* = 612 $[M+H]^+$; $^1H$ NMR (400 MHz, DMSO-$d_6$): δ [ppm] = 10.78 (s, 1H), 9.14 (s, 1H), 8.33 (d, *J* = 2.3 Hz, 1H), 7.91 (d, *J* = 8.9 Hz, 1H), 7.87-7.77 (m, 4H), 7.19 (s, 1H), 6.54 (s, 1H), 5.66-5.57 (m, 1H), 3.28 (s, 3H), 2.65 (s, 3H), 2.64 (s, 3H), 2.22-2.03 (m, 2H), 0.81 (t, *J* = 7.2 Hz, 3H).

**(2*S*)-2-[4-{5-Chloro-2-[4-(trifluoromethyl)-1*H*-1,2,3-triazol-1-yl]phenyl}-5-methoxy-2-oxopyridin-1(2*H*)-yl]-*N*-(2,3-dimethylquinoxalin-6-yl)butanamide (eutomer 72).**

Enantiomer separation of 88 mg of 2-[4-{5-chloro-2-[4-(trifluoromethyl)-1*H*-1,2,3-triazol-1-yl]phenyl}-5-methoxy-2-oxopyridin-1(2*H*)-yl]-*N*-(2,3-dimethylquinoxalin-6-yl)butanamide (racemate **314**) gave 34 mg of distomer (chiral HPLC: $t_R$ = 1.96 min) and 34 mg of eutomer **72** (chiral HPLC: $t_R$ = 4.72 min, >99% ee).

LC/MS (method 1): $t_R$ = 1.02 min, MS (ESIpos): *m/z* = 612 $[M+H]^+$; $^1H$ NMR (400 MHz, DMSO-$d_6$): δ [ppm] = 10.78 (s, 1H), 9.14 (s, 1H), 8.33 (d, *J* = 2.2 Hz, 1H), 7.91 (d, *J* = 8.9 Hz, 1H), 7.88-7.77 (m, 4H), 7.20 (s, 1H), 6.54 (s, 1H), 5.66-5.57 (m, 1H), 3.28 (s, 3H), 2.65 (s, 3H), 2.64 (s, 3H), 2.22-2.03 (m, 2H), 0.81 (t, *J* = 7.2 Hz, 3H).

Separation method: SFC: column: Daicel Chiralpak AD-H 5 μm, 250 mm × 20 mm; mobile phase: 75% carbon dioxide / 25% ethanol; temperature: 40 °C; flow rate: 80 mL/min; UV detection: 210 nm.

Analytical method: SFC: column: Daicel Chiralpak AD-H 3 μm, 100 mm × 4.6 mm; mobile phase: 75% carbon dioxide, 25% ethanol; flow rate: 3 mL/min; UV detection: 210 nm.

**Synthesis of compound 73.**[a]

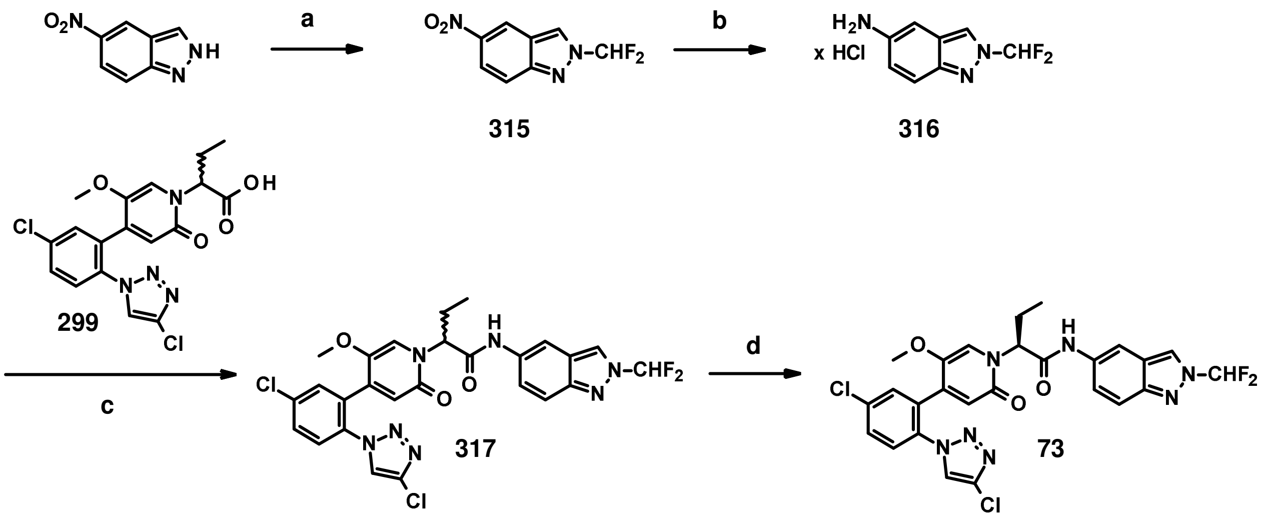


[a]Reagents and conditions: (a) difluoro(fluorosulfonyl)acetic acid, $K_2CO_3$, EtOAc, RT, 63%; (b) i) $H_2$, Pd/C, EtOH, RT, ii) HCl/1,4-dioxane, RT, 53%; (c) T3P, pyridine, RT, 75%; (d) enantiomer separation.

**2-(Difluoromethyl)-5-nitro-2*H*-indazole (315).**

Potassium carbonate (1.27 g, 9.20 mmol, 2.0 eq.) and difluoro(fluorosulfonyl)acetic acid (1.64 g, 9.20 mmol, 2.0 eq.) were added under argon atmosphere at RT to a solution of 5-nitro-1*H*-indazole (0.75 g, 4.60 mmol) in ethyl acetate (22.5 mL). The reaction mixture was stirred at RT for 2 h (until gas evolution ceased) and diluted cautiously with aqueous saturated sodium carbonate solution. After phase separation, the aqueous phase was extracted with ethyl acetate. The combined organic phases were washed with water and brine, dried over anhydrous magnesium sulfate, filtered and concentrated under reduced pressure. The residue was purified by flash silica gel chromatography (cyclohexane / ethyl acetate-gradient) to give **315**. Yield: 617 mg (63% of theory). $^1$H NMR (400 MHz, DMSO-$d_6$): δ [ppm] = 9.31 (s, 1H), 8.99 (d, $J$ = 2.2 Hz, 1H), 8.27 (t, $J$ = 58.6 Hz, 1H), 8.12 (dd, $J$ = 9.5 Hz, 2.4 Hz, 1H), 7.95 (d, $J$ = 9.5 Hz, 1H).

**2-(Difluoromethyl)-2*H*-indazol-5-amine hydrochloride (316).**

Palladium (10% on charcoal, 151 mg) was added under argon atmosphere at RT to a solution of 2-(difluoromethyl)-5-nitro-2*H*-indazole (**315**) (605 mg, 2.84 mmol) in ethanol (15 mL). The reaction mixture was stirred under hydrogen atmosphere (1 bar) at RT for 3 h, filtered through Celite®, and the filter residue was washed with ethanol. The combined filtrates were concentrated under reduced pressure. The residue was taken up in 1,4-dioxane (10 mL) and mixed with hydrochloric acid solution (4 M in 1,4-dioxane, 2 mL). The resulting suspension was diluted with 1,4-dioxane (5 mL), stirred for 5 min and filtered. The filter residue was washed with diethyl ether and dried *in vacuo* to give **316** which was used without further purification. Yield: 369 mg (90% purity, 53% of theory). LC/MS (method 3): $t_R$ = 0.52 min, MS (ESIpos): $m/z$ = 184 $[M+H]^+$; $^1$H NMR (400 MHz, DMSO-$d_6$): δ [ppm] = 9.3 (br s,

2H), 8.92 (s, 1H), 8.16 (t, $J$ = 59.0 Hz, 1H), 7.82 (d, $J$ = 9.3 Hz, 1H), 7.74-7.66 (m, 1H), 7.29 (d, $J$ = 9.3 Hz, 1H).

**2-{4-[5-Chloro-2-(4-chloro-1*H*-1,2,3-triazol-1-yl)phenyl]-5-methoxy-2-oxopyridin-1(2*H*)-yl}-*N*-[2-(difluoromethyl)-2*H*-indazol-5-yl]butanamide (racemate 317).**

Propylphosphonic anhydride (T3P, 50% solution in ethyl acetate, 562 µL, 0.95 mmol, 4.0 eq.) was added under argon atmosphere at RT to a solution of 2-{4-[5-chloro-2-(4-chloro-1*H*-1,2,3-triazol-1-yl)phenyl]-5-methoxy-2-oxopyridin-1(2*H*)-yl}butanoic acid (racemate **316**) (100 mg, 0.24 mmol) and 2-(difluoromethyl)-2*H*-indazol-5-amine hydrochloride (69 mg, 90% purity, 0.28 mmol, 1.2 eq.) in pyridine (1.8 mL). The reaction mixture was stirred at RT for additional 5 min and purified without further work-up by preparative RP-HPLC (acetonitrile / 0.1% formic acid in water gradient) to give **317**. Yield: 104 mg (75% of theory). LC/MS (method 3): $t_R$ = 1.84 min, MS (ESIpos): $m/z$ = 588 $[M+H]^+$; $^1H$ NMR (400 MHz, DMSO-$d_6$): δ [ppm] = 10.50 (s, 1H), 8.80 (s, 1H), 8.63 (s, 1H), 8.26 (s, 1H), 8.09 (t, $J$ = 59.2 Hz, 1H), 7.83-7.66 (m, 4H), 7.42 (dd, $J$ = 9.4 Hz, 1.8 Hz, 1H), 7.23 (s, 1H), 6.48 (s, 1H), 5.66-5.56 (m, 1H), 3.32 (s, 3H), 2.20-1.99 (m, 2H), 0.84 (t, $J$ = 7.2 Hz, 3H).

**(2*S*)-2-{4-[5-Chloro-2-(4-chloro-1*H*-1,2,3-triazol-1-yl)phenyl]-5-methoxy-2-oxopyridin-1(2*H*)-yl}-*N*-[2-(difluoromethyl)-2*H*-indazol-5-yl]butanamide (eutomer 73).**

Enantiomer separation of 95 mg of 2-{4-[5-chloro-2-(4-chloro-1*H*-1,2,3-triazol-1-yl)phenyl]-5-methoxy-2-oxopyridin-1(2*H*)-yl}-*N*-[2-(difluoromethyl)-2*H*-indazol-5-yl]butanamide (racemate **317**) gave 27 mg of distomer (chiral HPLC: $t_R$ = 1.41 min) and 28 mg of eutomer **73** (chiral HPLC: $t_R$ = 3.48 min, >99% ee).

LC/MS (method 3): $t_R$ = 1.83 min, MS (ESIneg): $m/z$ = 586 $[M-H]^-$; $^1H$ NMR (400 MHz, DMSO-$d_6$): δ [ppm] = 10.50 (s, 1H), 8.79 (s, 1H), 8.63 (s, 1H), 8.26 (s, 1H), 8.09 (t, $J$ = 59.1 Hz, 1H), 7.82-7.67 (m, 4H), 7.42 (dd, $J$ = 9.3 Hz, 1.8 Hz, 1H), 7.23 (s, 1H), 6.48 (s, 1H), 5.66-5.56 (m, 1H), 3.32 (s, 3H), 2.20-1.99 (m, 2H), 0.84 (t, $J$ = 7.2 Hz, 3H).

Separation method: SFC: column: Daicel Chiralpak AD-H 5 µm, 250 mm × 20 mm; mobile phase: 70% carbon dioxide / 30% ethanol; temperature: 40 °C; flow rate: 80 mL/min; UV detection: 210 nm.

Analytical method: SFC: column: Daicel Chiralpak AD-H 3 µm, 100 mm × 4.6 mm; mobile phase: 70% carbon dioxide, 30% ethanol; flow rate: 3 mL/min; UV detection: 210 nm.

**Synthesis of compound 74.**[a]

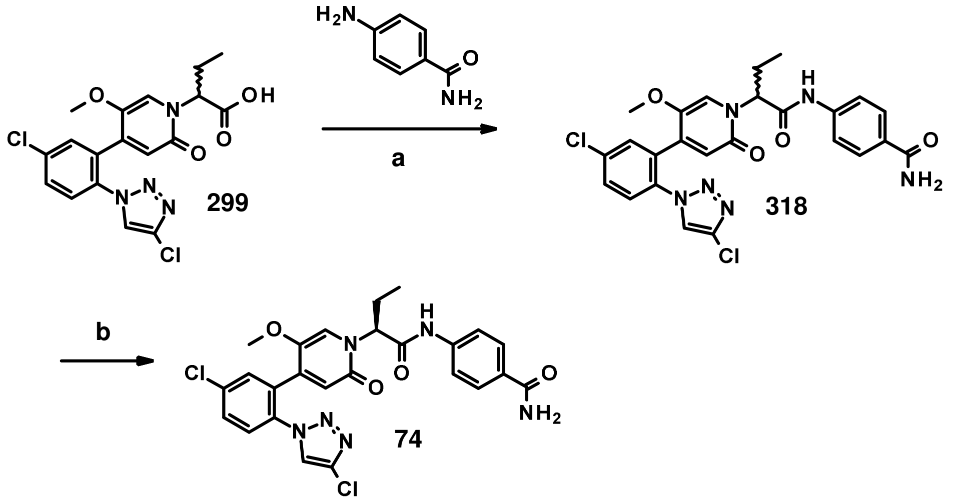


[a]Reagents and conditions: (a) T3P, pyridine, 40 °C, 76%; (b) enantiomer separation.

**4-(2-{4-[5-Chloro-2-(4-chloro-1*H*-1,2,3-triazol-1-yl)phenyl]-5-methoxy-2-oxopyridin-1(2*H*)-yl}butanamido)benzamide (racemate 318).**

Propylphosphonic anhydride (T3P, 50% solution in ethyl acetate, 647 $\mu$L, 1.13 mmol, 1.6 eq.) was added under argon atmosphere at RT to a solution of 2-{4-[5-chloro-2-(4-chloro-1*H*-1,2,3-triazol-1-yl)phenyl]-5-methoxy-2-oxopyridin-1(2*H*)-yl}butanoic acid (racemate **299**) (300 mg, 0.71 mmol) in pyridine (4 mL). The reaction mixture was heated to 40 °C, and 4-aminobenzamide (125 mg, 0.92 mmol, 1.3 eq.) was added. The reaction mixture was stirred at 40 °C for additional 10 min, cooled to RT, diluted with acetonitrile / water (1:1) and purified by preparative RP-HPLC (acetonitrile / 0.1% formic acid in water gradient) to give **318**. Yield: 293 mg (76% of theory). LC/MS (method 1): $t_R$ = 0.83 min, MS (ESIpos): *m*/*z* = 541 $[M+H]^+$; $^1$H NMR (400 MHz, DMSO-$d_6$): δ [ppm] = 10.61 (s, 1H), 8.62 (s, 1H), 7.90-7.72 (m, 6H), 7.67 (d, *J* = 8.8 Hz, 2H), 7.24 (br s, 1H), 7.19 (s, 1H), 6.47 (s, 1H), 5.61-5.53 (m, 1H), 3.3 (s, 3H, concealed), 2.19-1.99 (m, 2H), 0.82 (t, *J* = 7.3 Hz, 3H).

**4-{[(2*S*)-2-{4-[5-Chloro-2-(4-chloro-1*H*-1,2,3-triazol-1-yl)phenyl]-5-methoxy-2-oxopyridin-1(2*H*)-yl}butanoyl]amino}benzamide (eutomer 74).**

Enantiomer separation of 284 mg of 4-(2-{4-[5-chloro-2-(4-chloro-1*H*-1,2,3-triazol-1-yl)phenyl]-5-methoxy-2-oxopyridin-1(2*H*)-yl}butanamido)benzamide (racemate **318**) gave 125 mg of distomer (chiral HPLC: $t_R$ = 3.16 min) and 109 mg of eutomer **74** (chiral HPLC: $t_R$ = 5.77 min, >99% ee).
LC/MS (method 3): $t_R$ = 1.51 min, MS (ESIpos): *m*/*z* = 541 $[M+H]^+$; $^1$H NMR (400 MHz, DMSO-$d_6$): δ [ppm] = 10.61 (s, 1H), 8.62 (s, 1H), 7.90-7.72 (m, 6H), 7.67 (d, *J* = 8.7 Hz, 2H), 7.24 (br s, 1H), 7.19 (s, 1H), 6.47 (s, 1H), 5.61-5.54 (m, 1H), 3.3 (s, 3H, concealed), 2.19-2.00 (m, 2H), 0.82 (t, *J* = 7.2 Hz, 3H).
Separation method: SFC: column: Daicel Chiralpak AD-H 5 $\mu$m, 250 mm × 20 mm; mobile phase: 75%

carbon dioxide / 35% ethanol; temperature: 40 °C; flow rate: 80 mL/min; UV detection: 210 nm. Analytical method: SFC: column: Daicel Chiralpak AD-H 3 μm, 100 mm × 4.6 mm; mobile phase: 75% carbon dioxide, 25% ethanol; flow rate: 3 mL/min; UV detection: 210 nm.

**Synthesis of eutomer 75.**[a]

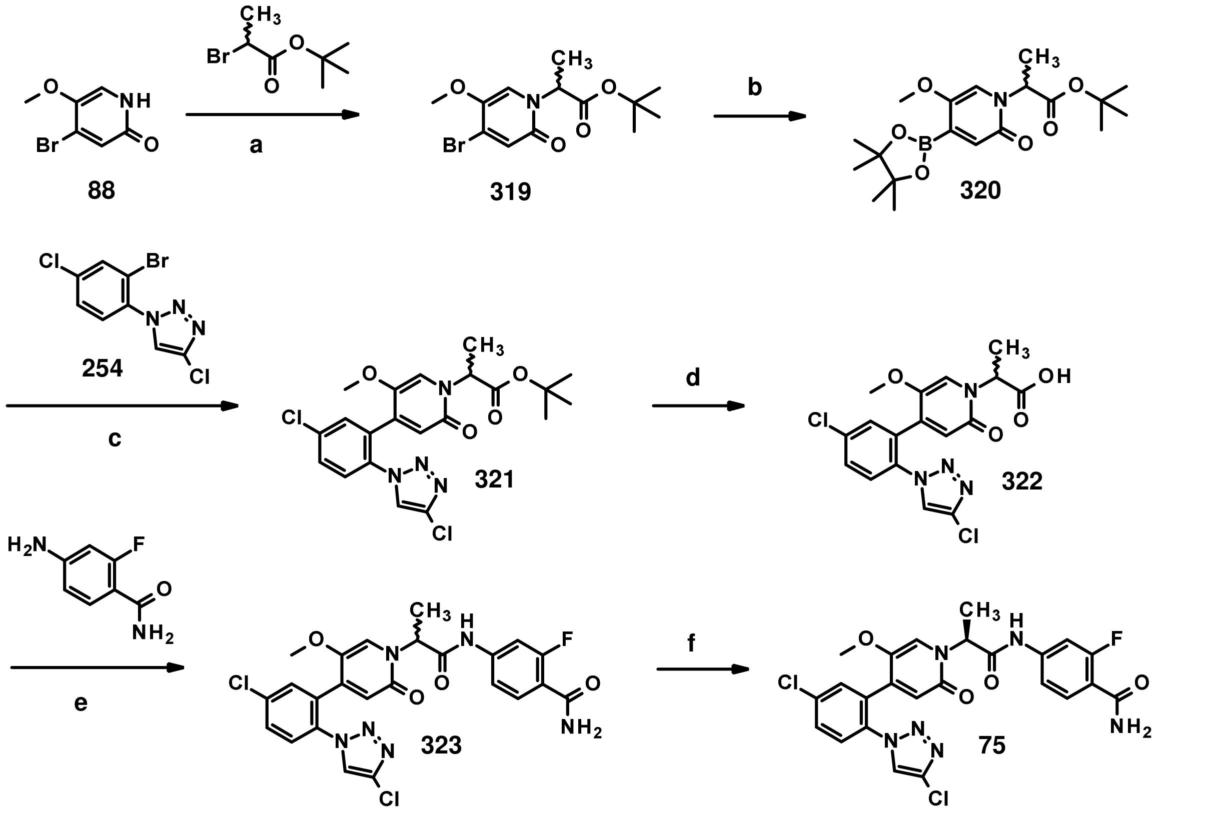


[a]Reagents and conditions: (a) $K_2CO_3$, DMF, 50 °C, 45%; (b) $(BPin)_2$, Pd(dppf)$Cl_2$-DCM complex, KOAc, 1,4-dioxane, 80 °C, 50%; (c) Pd(dppf)$Cl_2$-DCM complex, aq. $Na_2CO_3$, 1,4-dioxane, 100 °C, 53%; (d) HCl/1,4-dioxane, RT, 84%; (e) T3P, pyridine, 50 °C, 68%; (f) enantiomer separation.

***tert*-Butyl 2-(4-bromo-5-methoxy-2-oxopyridin-1(2*H*)-yl)propanoate (racemate 319).**

Racemic *tert*-butyl 2-bromopropanoate (11.71 mL, 7.58 mmol, 1.2 eq.) was added under argon atmosphere at RT to a suspension of 4-bromo-5-methoxypyridin-2(1*H*)-one (**88**) (12.00 g, 58.82 mmol) and potassium carbonate (20.32 g, 147.04 mmol, 2.5 eq.) in *N,N*-dimethylformamide (210 mL). The reaction mixture was stirred at 50 °C for 2 h and diluted with brine. After addition of ethyl acetate and phase separation, the aqueous phase was extracted with ethyl acetate. The combined organic phases were washed with brine, dried and concentrated under reduced pressure. The residue was purified by flash silica gel chromatography (cyclohexane / ethyl acetate gradient) to give **319**. Yield: 8.70 g (45% of theory). LC/MS (method 3): $t_R$ = 1.64 min, MS (ESIpos): $m/z$ = 332 $[M+H]^+$; $^1H$ NMR (400 MHz, DMSO-$d_6$): δ [ppm] = 7.40 (s, 1H), 6.85 (s, 1H), 5.00 (q, $J$ = 7.2 Hz, 2H), 3.73 (s, 3H), 1.53 (d, 3H),

1.38 (s, $J$ = 7.2 Hz, 9H).

***tert*-Butyl 2-[5-methoxy-2-oxo-4-(4,4,5,5-tetramethyl-1,3,2-dioxaborolan-2-yl)pyridin-1(2*H*)-yl]propanoate (racemate 320).**

[1,1-Bis(diphenylphosphino)ferrocene]dichloropalladium-dichloromethane complex (0.96 g, 1.17 mmol, 0.03 eq.) was added under argon atmosphere at RT to a suspension of *tert*-butyl 2-(4-bromo-5-methoxy-2-oxopyridin-1(2*H*)-yl)propanoate (racemate **319**) (13.00 g, 39.13 mmol), bis(pinacolato)diboron (10.93 g, 43.05 mmol, 1.1 eq.) and potassium acetate (11.52 g, 117.40 mmol, 3.0 eq.) in 1,4-dioxane (284 mL). The mixture was stirred at 80 °C for 1.5 h, cooled to RT and filtered through Celite®, and the filter residue was washed with ethyl acetate. The combined filtrates were concentrated under reduced pressure. The residue was dried *in vacuo* to give **320** which was used without further purification. Yield: 31.80 g (50% purity). LC/MS (method 1): $t_R$ = 0.61 min, MS (ESIpos): $m/z$ = 298 $[M+H]^+$ [boronic acid fragment].

***tert*-Butyl 2-{4-[5-chloro-2-(4-chloro-1*H*-1,2,3-triazol-1-yl)phenyl]-5-methoxy-2-oxopyridin-1(2*H*)-yl}propanoate (racemate 321).**

1-(2-Bromo-4-chlorophenyl)-4-chloro-1*H*-1,2,3-triazole (**254**) (4.30 g, 14.68 mmol) and [1,1-bis(diphenylphosphino)ferrocene]dichloropalladium-dichloromethane complex (1.20 g, 1.47 mmol, 0.1 eq.) were added under argon atmosphere at RT to a mixture of *tert*-butyl 2-[5-methoxy-2-oxo-4-(4,4,5,5-tetramethyl-1,3,2-dioxaborolan-2-yl)pyridin-1(2*H*)-yl]propanoate (racemate **320**) (14.61 g, 40% purity, 15.41 mmol, 1.05 eq.) and aqueous sodium carbonate solution (22.02 mL, 44.03 mmol, 3.0 eq.) in 1,4-dioxane (135 mL). The reaction mixture was stirred at 100 °C for 2 h, cooled to RT and added to water. The aqueous phase was extracted with methyl *tert*-butyl ether. The combined organic phases were dried over anhydrous sodium sulfate, filtered, concentrated under reduced pressure and dried *in vacuo*. The residue was purified by flash silica gel chromatography (cyclohexane / ethyl acetate gradient) to give **321**. Yield: 3.87 g (94% purity, 53% of theory). LC/MS (method 1): $t_R$ = 1.00 min, MS (ESIpos): $m/z$ = 465 $[M+H]^+$; $^1$H NMR (400 MHz, DMSO-$d_6$): δ [ppm] = 8.62 (s, 1H), 7.78 (dd, $J$ = 8.6 Hz, 2.3 Hz, 1H), 7.73 (d, $J$ = 8.6 Hz, 1H), 7.71 (d, $J$ = 2.2 Hz, 1H), 7.12 (s, 1H), 6.41 (s, 1H), 5.06-4.92 (m, 1H), 3.92 (s, 3H), 1.50 (d, $J$ = 7.1 Hz, 3H), 1.38 (s, 9H).

**2-{4-[5-Chloro-2-(4-chloro-1*H*-1,2,3-triazol-1-yl)phenyl]-5-methoxy-2-oxopyridin-1(2*H*)-yl}propanoic acid (racemate 322).**

Hydrogen chloride solution (4 M in 1,4-dioxane, 144 mL) was added to *tert*-butyl 2-{4-[5-chloro-2-(4-chloro-1*H*-1,2,3-triazol-1-yl)phenyl]-5-methoxy-2-oxopyridin-1(2*H*)-yl}propanoate (racemate **321**) (4.50 g, 9.67 mmol). The reaction mixture was stirred at RT for 4 h and concentrated under reduced pressure. The residue was stirred in acetonitrile, and the solid was filtered, washed with acetonitrile and dried *in vacuo* to give a first batch of **322**. Yield: 1.60 g (86% purity, 35% of theory). The combined filtrates were concentrated under reduced pressure. The residue stirred in acetonitrile, and the solid was

filtered, washed with acetonitrile and dried *in vacuo* to give a second batch of **322**. Yield: 2.40 g (81% purity, 49% of theory). LC/MS (method 1): $t_R$ = 0.75 min, MS (ESIpos): *m*/*z* = 409 $[M+H]^+$.

**4-[(2-{4-[5-Chloro-2-(4-chloro-1*H*-1,2,3-triazol-1-yl)phenyl]-5-methoxy-2-oxopyridin-1(2*H*)-yl}propanoyl)amino]-2-fluorobenzamide (racemate 323).**

Propylphosphonic anhydride (T3P, 50% solution in ethyl acetate, 600 µL, 1.01 mmol, 1.6 eq.) was added under argon atmosphere at RT to a solution of 2-{4-[5-chloro-2-(4-chloro-1*H*-1,2,3-triazol-1-yl)phenyl]-5-methoxy-2-oxopyridin-1(2*H*)-yl}propanoic acid (racemate **322**) (300 mg, 86% purity, 0.63 mmol) in pyridine (16.7 mL). The reaction mixture was heated to 50 °C, and 4-amino-2-fluorobenzamide (126 mg, 0.82 mmol, 1.3 eq.) was added. The reaction mixture was stirred at 50 °C for additional 1 h, cooled to RT, diluted with acetonitrile / water (1:1) and purified by preparative RP-HPLC (acetonitrile / 0.1% formic acid in water gradient) to give **323**. Yield: 234 mg (68% of theory). LC/MS (method 1): $t_R$ = 0.81 min, MS (ESIpos): *m*/*z* = 545 $[M+H]^+$; $^1$H NMR (400 MHz, DMSO-$d_6$): δ [ppm] = 10.68 (s, 1H), 8.67 (s, 1H), 7.81-7.60 (m, 5H), 7.56-7.48 (m, 2H), 7.41-7.34 (m, 1H), 7.16 (s, 1H), 6.46 (s, 1H), 5.51 (q, *J* = 7.3 Hz, 1H), 3.33 (s, 3H), 1.64 (d, *J* = 7.3 Hz, 3H).

**4-{[(2*S*)-2-{4-[5-Chloro-2-(4-chloro-1*H*-1,2,3-triazol-1-yl)phenyl]-5-methoxy-2-oxopyridin-1(2*H*)-yl}propanoyl]amino}-2-fluorobenzamide (eutomer 75).**

Enantiomer separation of 234 mg of 4-[(2-{4-[5-chloro-2-(4-chloro-1*H*-1,2,3-triazol-1-yl)phenyl]-5-methoxy-2-oxopyridin-1(2*H*)-yl}propanoyl)amino]-2-fluorobenzamide (racemate **323**) gave 103 mg of distomer (chiral HPLC: $t_R$ = 3.0 min) and 106 mg of eutomer **75** (chiral HPLC: $t_R$ = 8.6 min, >99% ee). LC/MS (method 3): $t_R$ = 1.49 min, MS (ESIpos): *m*/*z* = 545 $[M+H]^+$; $^1$H NMR (400 MHz, DMSO-$d_6$): δ [ppm] = 10.68 (s, 1H), 8.66 (s, 1H), 7.82-7.60 (m, 5H), 7.57-7.46 (m, 2H), 7.41-7.34 (m, 1H), 7.16 (s, 1H), 6.46 (s, 1H), 5.51 (q, *J* = 7.3 Hz, 1H), 3.33 (s, 3H), 1.64 (d, *J* = 7.3 Hz, 3H).

Separation method: SFC: column: Daicel Chiralpak AD-H 5 µm, 250 mm × 20 mm; mobile phase: 75% carbon dioxide / 25% ethanol; temperature: 40 °C; flow rate: 80 mL/min; UV detection: 210 nm.

Analytical method: column: SFC: Daicel Chiralpak AD-H 3 µm, 100 mm × 4.6 mm; mobile phase: 75% carbon dioxide / 25% ethanol; flow rate: 3 mL/min; UV detection: 210 nm.

**Synthesis of eutomer 76.**[a]

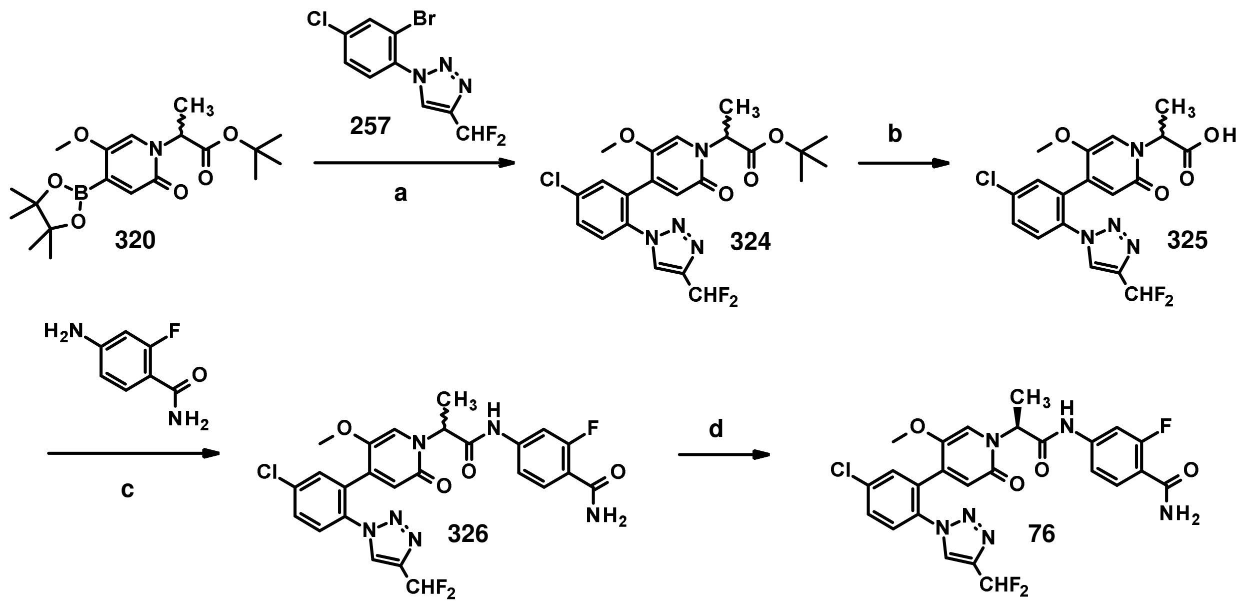

[a]Reagents and conditions: (a) Pd(dppf)$Cl_2$-DCM complex, aq. $Na_2CO_3$, 1,4-dioxane, 100 °C, 84%; (b) aq. LiOH, THF, RT, 67%; (c) T3P, pyridine, 50 °C, 43%; (d) enantiomer separation.

***tert*-Butyl 2-[4-{5-chloro-2-[4-(difluoromethyl)-1*H*-1,2,3-triazol-1-yl]phenyl}-5-methoxy-2-oxopyridin-1(2*H*)-yl]propanoate (racemate 324).**

Sodium carbonate solution (2 M in water, 24.31 mL, 48.62 mmol, 3.0 eq.) and 1-(2-bromo-4-chlorophenyl)-4-(difluoromethyl)-1*H*-1,2,3-triazole (**257**) (5.00 g, 16.21 mmol) were added under argon atmosphere at RT to a solution of methyl 2-[5-methoxy-2-oxo-4-(4,4,5,5-tetramethyl-1,3,2-dioxaborolan-2-yl)pyridin-1(2*H*)-yl]propanoate (racemate **320**) (16.14 g, 40% purity, 17.02 mmol, 1.05 eq.) in 1,4-dioxane (149 mL), followed by addition of [1,1'-bis(diphenylphosphino)ferrocene]dichloropalladium-dichloromethane complex (1.32 g, 1.62 mmol, 0.1 eq.). The reaction mixture was stirred at 100 °C for 2 h, cooled to RT and added to water. After phase separation, the aqueous phase was extracted with methyl *tert*-butyl ether. The combined organic phases were dried, filtered and concentrated under reduced pressure. The residue was purified by flash silica gel chromatography (cyclohexane / ethyl acetate gradient) to give **324** which was used without further purification. Yield: 2.60 g (84% purity). LC/MS (method 1): $t_R$ = 1.00 min, MS (ESIpos): $m/z$ = 481 $[M+H]^+$.

**2-[4-{5-Chloro-2-[4-(difluoromethyl)-1*H*-1,2,3-triazol-1-yl]phenyl}-5-methoxy-2-oxopyridin-1(2*H*)-yl]propanoic acid (racemate 325).**

Aqueous lithium hydroxide solution (1 M, 22.71 mL, 22.71 mmol, 5.0 eq.) was added to a solution of *tert*-butyl 2-[4-{5-chloro-2-[4-(difluoromethyl)-1*H*-1,2,3-triazol-1-yl]phenyl}-5-methoxy-2-oxopyridin-1(2*H*)-yl]propanoate (racemate **324**) (2.60 g, 84% purity, 4.51 mmol) in tetrahydrofuran

(32.7 mL). The reaction mixture was stirred at RT for 16 h, mixed with water, acidified to pH 4 with aqueous hydrochloric acid solution (1 N) and extracted with ethyl acetate. The combined organic phases were washed with brine, dried over anhydrous sodium sulfate, filtered and concentrated under reduced pressure to give **325** which was used without further purification. Yield: 1.30 g (67% of theory). LC/MS (method 1): $t_R$ = 0.74 min, MS (ESIpos): $m/z$ = 425 $[M+H]^+$; $^1$H NMR (400 MHz, DMSO-$d_6$): δ [ppm] = 12.84 (s, 1H), 8.74 (s, 1H), 7.81-7.70 (m, 3H), 7.23 (t, $J$ = 54.0 Hz, 1H), 7.13 (s, 1H), 6.45 (s, 1H), 5.13-5.04 (m, 1H), 3.22 (s, 3H), 1.52 (d, $J$ = 7.2 Hz, 3H).

**4-({2-[4-{5-Chloro-2-[4-(difluoromethyl)-1*H*-1,2,3-triazol-1-yl]phenyl}-5-methoxy-2-oxopyridin-1(2*H*)-yl]propanoyl}amino)-2-fluorobenzamide (racemate 326).**

Propylphosphonic anhydride (T3P, 50% solution in ethyl acetate, 896 µL, 1.51 mmol, 1.6 eq.) was added under argon atmosphere at RT to a solution of 2-[4-{5-chloro-2-[4-(difluoromethyl)-1*H*-1,2,3-triazol-1-yl]phenyl}-5-methoxy-2-oxopyridin-1(2*H*)-yl]propanoic acid (racemate **325**) (400 mg, 0.94 mmol) in pyridine (24.9 mL). The reaction mixture was heated to 50 °C, and 4-amino-2-fluorobenzamide (189 mg, 1.22 mmol, 1.3 eq.) was added. The reaction mixture was stirred at 50 °C for additional 1 h, cooled to RT, diluted with acetonitrile / water (1:1) and purified by preparative RP-HPLC (acetonitrile / 0.1% formic acid in water gradient) to give **326**. Yield: 229 mg (43% of theory). LC/MS (method 1): $t_R$ = 0.80 min, MS (ESIpos): $m/z$ = 561 $[M+H]^+$; $^1$H NMR (400 MHz, DMSO-$d_6$): δ [ppm] = 10.68 (s, 1H), 8.77 (s, 1H), 7.83-7.75 (m, 2H), 7.73-7.59 (m, 3H), 7.57-7.47 (m, 2H), 7.40-7.32 (m, 1H), 7.24 (t, $J$ = 53.9 Hz, 1H), 7.13 (s, 1H), 6.49 (s, 1H), 5.57-5.45 (m, 1H), 3.27 (s, 3H), 1.63 (d, $J$ = 7.3 Hz, 3H).

**4-({(2*S*)-2-[4-{5-Chloro-2-[4-(difluoromethyl)-1*H*-1,2,3-triazol-1-yl]phenyl}-5-methoxy-2-oxopyridin-1(2*H*)-yl]propanoyl}amino)-2-fluorobenzamide (eutomer 76).**

Enantiomer separation of 229 mg of 4-({2-[4-{5-chloro-2-[4-(difluoromethyl)-1*H*-1,2,3-triazol-1-yl]phenyl}-5-methoxy-2-oxopyridin-1(2*H*)-yl]propanoyl}amino)-2-fluorobenzamide (racemate **326**) gave 93 mg of distomer (chiral HPLC: $t_R$ = 2.3 min) and 92 mg of eutomer **76** (chiral HPLC: $t_R$ = 4.2 min, >99% ee).

LC/MS (method 1): $t_R$ = 0.81 min, MS (ESIpos): $m/z$ = 561 $[M+H]^+$; $^1$H NMR (400 MHz, DMSO-$d_6$): δ [ppm] = 10.68 (s, 1H), 8.77 (s, 1H), 7.83-7.75 (m, 2H), 7.73-7.59 (m, 3H), 7.57-7.47 (m, 2H), 7.40-7.33 (m, 1H), 7.24 (t, $J$ = 53.9 Hz, 1H), 7.13 (s, 1H), 6.49 (s, 1H), 5.57-5.45 (m, 1H), 3.27 (s, 3H), 1.63 (d, $J$ = 7.3 Hz, 3H).

Separation method: SFC: column: Daicel Chiralpak AD-H 5 µm, 250 mm × 20 mm; mobile phase: 80% carbon dioxide / 20% ethanol; temperature: 40 °C; flow rate: 80 mL/min; UV detection: 210 nm.

Analytical method: SFC: column: Daicel Chiralpak AD-H 3 µm, 100 mm × 4.6 mm; mobile phase: 75% carbon dioxide / 25% ethanol; flow rate: 3 mL/min; UV detection: 210 nm.

**Synthesis of eutomer 77.**[a]

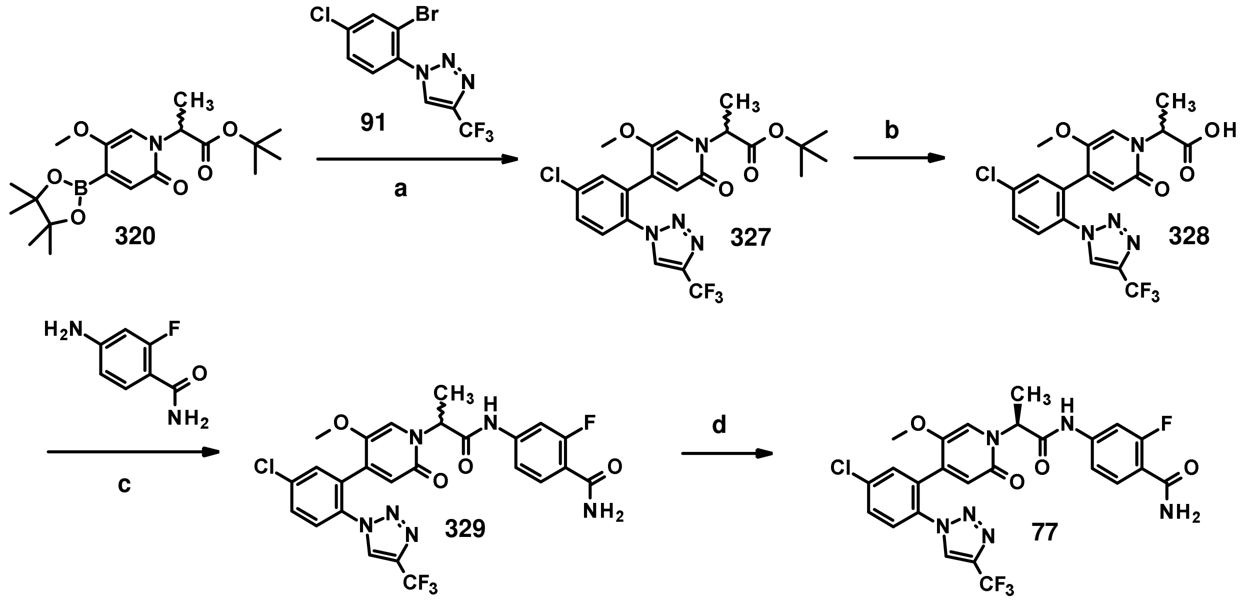

[a]Reagents and conditions: (a) Pd(dppf)$Cl_2$-DCM complex, $K_2CO_3$, 1,4-dioxane, 80 °C, 81%; (b) HCl/1,4-dioxane, RT, 100%; (c) T3P, pyridine, 40 °C, 49%; (d) enantiomer separation.

***tert*-Butyl 2-[4-{5-chloro-2-[4-(trifluoromethyl)-1*H*-1,2,3-triazol-1-yl]phenyl}-5-methoxy-2-oxopyridin-1(2*H*)-yl]propanoate (racemate 327).**

[1,1-Bis(diphenylphosphino)ferrocene]dichloropalladium-dichloromethane complex (147 mg, 0.18 mmol, 0.06 eq.) was added under argon atmosphere at RT to mixture of *tert*-butyl 2-[5-methoxy-2-oxo-4-(4,4,5,5-tetramethyl-1,3,2-dioxaborolan-2-yl)pyridin-1(2*H*)-yl]propanoate (racemate **320**) (2.17 g, 52% purity, 3.01 mmol), 1-(2-bromo-4-chlorophenyl)-4-(trifluoromethyl)-1*H*-1,2,3-triazole (**91**) (0.98 g, 3.01 mmol, 1.0 eq.) and potassium carbonate (1.25 g, 9.02 mmol, 3.0 eq.) in 1,4-dioxane (30.5 mL). The reaction mixture was stirred at 80 °C for 4 h, cooled to RT and filtered through Celite®, and the filter residue was washed with dichloromethane and acetonitrile. The combined filtrates were concentrated under reduced pressure. The residue was purified by flash silica gel chromatography (cyclohexane / ethyl acetate gradient) to give **327**. Yield: 1.21 g (81% of theory). LC/MS (method 3): $t_R$ = 2.02 min, MS (ESIpos): $m/z$ = 499 $[M+H]^+$; $^1H$ NMR (400 MHz, DMSO-$d_6$): δ [ppm] = 9.17 (s, 1H), 7.83-7.79 (m, 2H), 7.75 (s, 1H), 7.08 (s, 1H), 6.47 (s, 1H), 5.06-4.93 (m, 1H), 3.25 (s, 3H), 1.48 (d, $J$ = 7.1 Hz, 3H), 1.37 (s, 9H).

**2-[4-{5-Chloro-2-[4-(trifluoromethyl)-1*H*-1,2,3-triazol-1-yl]phenyl}-5-methoxy-2-oxopyridin-1(2*H*)-yl]propanoic acid (racemate 328).**

A mixture of *tert*-butyl 2-[4-{5-chloro-2-[4-(trifluoromethyl)-1*H*-1,2,3-triazol-1-yl]phenyl}-5-methoxy-2-oxopyridin-1(2*H*)-yl]propanoate (racemate **327**) (1.19 g, 2.39 mmol) in hydrogen chloride solution (4 M in 1,4-dioxane, 31.9 mL) was stirred at RT overnight and concentrated under reduced

pressure to give **328** which was used without further purification. Yield: 1.17 g (90% purity, 100% of theory). LC/MS (method 1): $t_R$ = 0.82 min, MS (ESIpos): $m/z$ = 443 $[M+H]^+$; $^1$H NMR (400 MHz, DMSO-$d_6$): δ [ppm] = 12.85 (s, 1H), 9.15 (s, 1H), 7.86-7.79 (m, 2H), 7.76 (s, 1H), 7.13 (s, 1H), 6.47 (s, 1H), 5.13-5.04 (m, 1H), 3.22 (s, 3H), 1.52 (d, $J$ = 7.2 Hz, 3H).

**4-({2-[4-{5-Chloro-2-[4-(trifluoromethyl)-1*H*-1,2,3-triazol-1-yl]phenyl}-5-methoxy-2-oxopyridin-1(2*H*)-yl]propanoyl}amino)-2-fluorobenzamide (racemate 329).**

Propylphosphonic anhydride (T3P, 50% solution in ethyl acetate, 0.77 mL, 1.30 mmol, 1.6 eq.) was added under argon atmosphere at RT to a solution of 2-[4-{5-chloro-2-[4-(trifluoromethyl)-1*H*-1,2,3-triazol-1-yl]phenyl}-5-methoxy-2-oxopyridin-1(2*H*)-yl]propanoic acid (racemate **328**) (400 mg, 90% purity, 0.81 mmol) in pyridine (21.5 mL). The reaction mixture was heated to 40 °C, and 4-amino-2-fluorobenzamide (163 mg, 1.06 mmol, 1.3 eq.) was added. The reaction mixture was stirred at 40 °C for additional 25 min, cooled to RT and concentrated under reduced pressure. The residue was dissolved in acetonitrile, acidified with aqueous hydrochloric acid solution (1 N) and purified by preparative RP-HPLC (acetonitrile / 0.1% formic acid in water gradient) to give **329**. Yield: 231 mg (49% of theory). LC/MS (method 1): $t_R$ = 0.87 min, MS (ESIpos): $m/z$ = 579 $[M+H]^+$; $^1$H NMR (400 MHz, DMSO-$d_6$): δ [ppm] = 10.67 (s, 1H), 9.17 (s, 1H), 7.87-7.80 (m, 2H), 7.75 (s, 1H), 7.72-7.59 (m, 2H), 7.56-7.48 (m, 2H), 7.36 (dd, $J$ = 8.6 Hz, 1.8 Hz, 1H), 7.12 (s, 1H), 6.51 (s, 1H), 5.56-5.46 (m, 1H), 3.27 (s, 3H), 1.63 (d, $J$ = 7.2 Hz, 3H).

**4-({(2*S*)-2-[4-{5-Chloro-2-[4-(trifluoromethyl)-1*H*-1,2,3-triazol-1-yl]phenyl}-5-methoxy-2-oxopyridin-1(2*H*)-yl]propanoyl}amino)-2-fluorobenzamide (eutomer 77).**

Enantiomer separation of 230 mg of 4-({2-[4-{5-chloro-2-[4-(trifluoromethyl)-1*H*-1,2,3-triazol-1-yl]phenyl}-5-methoxy-2-oxopyridin-1(2*H*)-yl]propanoyl}amino)-2-fluorobenzamide (racemate **329**) gave 106 mg of distomer (chiral HPLC: $t_R$ = 1.0 min) and 96 mg of eutomer **77** (chiral HPLC: $t_R$ = 1.8 min, >99% ee).

LC/MS (method 3): $t_R$ = 1.62 min, MS (ESIpos): $m/z$ = 579 $[M+H]^+$; $^1$H NMR (400 MHz, DMSO-$d_6$): δ [ppm] = 10.67 (s, 1H), 9.17 (s, 1H), 7.87-7.79 (m, 2H), 7.75 (s, 1H), 7.72-7.59 (m, 2H), 7.57-7.47 (m, 2H), 7.36 (dd, $J$ = 8.6 Hz, 1.8 Hz, 1H), 7.12 (s, 1H), 6.51 (s, 1H), 5.56-5.46 (m, 1H), 3.27 (s, 3H), 1.63 (d, $J$ = 7.2 Hz, 3H).

Separation method: SFC: column: Daicel Chiralpak AD-H 5 $\mu$m, 250 mm × 20 mm; mobile phase: 80% carbon dioxide / 20% ethanol; temperature: 40 °C; flow rate: 80 mL/min; UV detection: 210 nm.

Analytical method: SFC: column: Daicel Chiralpak AD-H 3 $\mu$m, 100 mm × 4.6 mm; mobile phase: 70% carbon dioxide / 30% ethanol; flow rate: 3 mL/min; UV detection: 210 nm.

**Synthesis of eutomer 78.**[a]

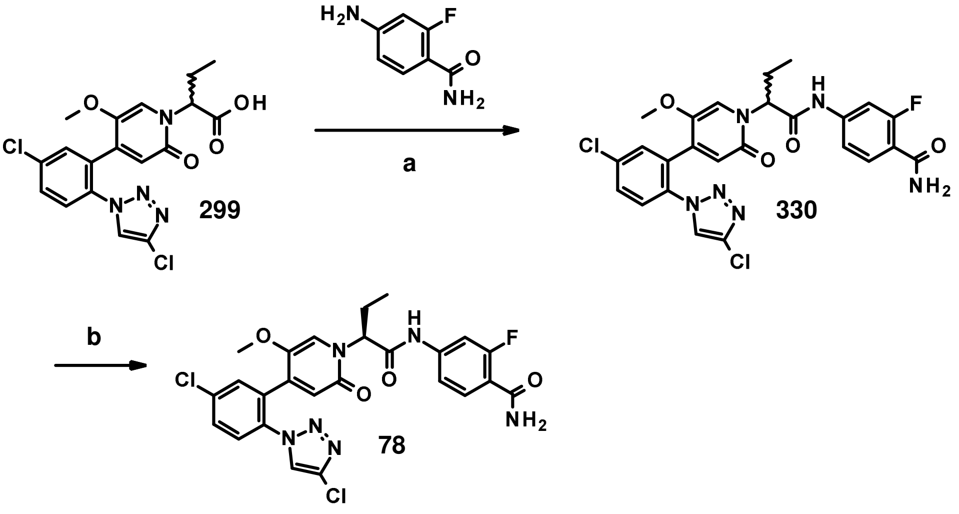

[a]Reagents and conditions: (a) T3P, pyridine, 50 °C, 65%; (b) enantiomer separation.

**4-[(2-{4-[5-Chloro-2-(4-chloro-1*H*-1,2,3-triazol-1-yl)phenyl]-5-methoxy-2-oxopyridin-1(2*H*)-yl}butanoyl)amino]-2-fluorobenzamide (racemate 330).**

Propylphosphonic anhydride (T3P, 50% solution in ethyl acetate, 562 µL, 0.95 mmol, 2.0 eq.) was added under argon atmosphere at RT to a solution of 2-{4-[5-chloro-2-(4-chloro-1*H*-1,2,3-triazol-1-yl)phenyl]-5-methoxy-2-oxopyridin-1(2*H*)-yl}butanoic acid (racemate **299**) (200 mg, 0.47 mmol) in pyridine (12.5 mL). The mixture was heated to 50 °C, and 4-amino-2-fluorobenzamide (95 mg, 0.61 mmol, 1.3 eq.) was added. The reaction mixture was stirred at 50 °C for additional 1 h, cooled to RT, diluted with acetonitrile / water (1:1) and purified by preparative RP-HPLC (acetonitrile / 0.1% formic acid in water gradient) to give **330**. Yield: 171 mg (65% of theory). LC/MS (method 3): $t_R$ = 1.59 min, MS (ESIpos): $m/z$ = 559 $[M+H]^+$; $^1$H NMR (400 MHz, DMSO-$d_6$): δ [ppm] = 10.78 (s, 1H), 8.62 (s, 1H), 7.82-7.73 (m, 3H), 7.72-7.61 (m, 2H), 7.57-7.48 (m, 2H), 7.41-7.35 (m, 1H), 7.18 (s, 1H), 6.48 (s, 1H), 5.58-5.49 (m, 1H), 2.18-2.02 (m, 2H), 0.82 (t, $J$ = 7.2 Hz, 3H).

**4-{[(2*S*)-2-{4-[5-Chloro-2-(4-chloro-1*H*-1,2,3-triazol-1-yl)phenyl]-5-methoxy-2-oxopyridin-1(2*H*)-yl}butanoyl]amino}-2-fluorobenzamide (eutomer 78).**

Enantiomer separation of 170 mg of 4-[(2-{4-[5-chloro-2-(4-chloro-1*H*-1,2,3-triazol-1-yl)phenyl]-5-methoxy-2-oxopyridin-1(2*H*)-yl}butanoyl)amino]-2-fluorobenzamide (racemate **330**) gave 68 mg of distomer (chiral HPLC: $t_R$ = 1.3 min) and 68 mg of eutomer **78** (chiral HPLC: $t_R$ = 1.6 min, >99% ee). LC/MS (method 3): $t_R$ = 1.59 min, MS (ESIpos): $m/z$ = 559 $[M+H]^+$; $^1$H NMR (400 MHz, DMSO-$d_6$): δ [ppm] = 10.77 (s, 1H), 8.62 (s, 1H), 7.82-7.72 (m, 3H), 7.72-7.61 (m, 2H), 7.57-7.48 (m, 2H), 7.41-7.35 (m, 1H), 7.18 (s, 1H), 6.48 (s, 1H), 5.58-5.49 (m, 1H), 2.18-2.02 (m, 2H), 0.82 (t, $J$ = 7.2 Hz, 3H). Separation method: SFC: column: Daicel Chiralpak AD-H 5 µm, 250 mm × 20 mm; mobile phase: 75% carbon dioxide / 25% ethanol; temperature: 40 °C; flow rate: 80 mL/min; UV detection: 210 nm.

Analytical method: SFC: column: Daicel Chiralpak AD-H 3 $\mu$m, 100 mm × 4.6 mm; mobile phase: 70% carbon dioxide / 30% ethanol; flow rate: 3 mL/min; UV detection: 210 nm.

**Synthesis of eutomer 79.**[a]

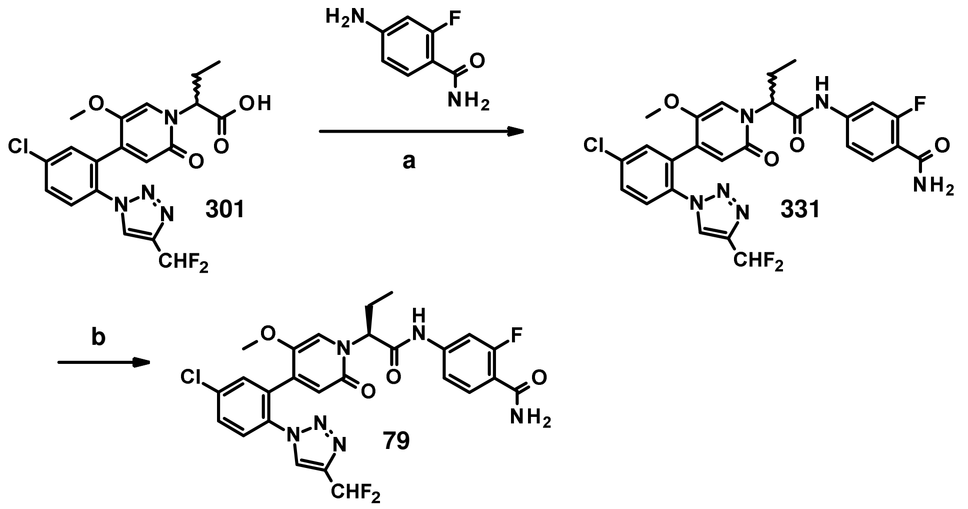

[a]Reagents and conditions: (a) T3P, pyridine, 40 °C, 59%; (b) enantiomer separation.

**4-({2-[4-{5-Chloro-2-[4-(difluoromethyl)-1*H*-1,2,3-triazol-1-yl]phenyl}-5-methoxy-2-oxopyridin-1(2*H*)-yl]butanoyl}amino)-2-fluorobenzamide (racemate 331).**

Propylphosphonic anhydride (T3P, 50% solution in ethyl acetate, 0.76 mL, 1.28 mmol, 1.6 eq.) was added under argon atmosphere at RT to a solution of 2-[4-{5-chloro-2-[4-(difluoromethyl)-1*H*-1,2,3-triazol-1-yl]phenyl}-5-methoxy-2-oxopyridin-1(2*H*)-yl]butanoic acid (racemate **301**) (350 mg, 0.80 mmol) in pyridine (16.7 mL). The reaction mixture was heated to 40 °C, and 4-amino-2-fluorobenzamide (160 mg, 1.04 mmol, 1.3 eq.) was added. The reaction mixture was stirred at 40 °C for additional 15 min and immediately concentrated under reduced pressure. The residue was taken up in acetonitrile, acidified with aqueous hydrochloric acid solution (1 N) and purified by preparative RP-HPLC (acetonitrile / 0.1% formic acid in water gradient) to give **331**. Yield: 270 mg (59% of theory). LC/MS (method 1): $t_R$ = 0.85 min, MS (ESIpos): $m/z$ = 575 $[M+H]^+$; $^1H$ NMR (400 MHz, DMSO-$d_6$): δ [ppm] = 10.77 (br s, 1H), 8.72 (s, 1H), 7.82-7.73 (m, 3H), 7.72-7.60 (m, 2H), 7.56-7.48 (m, 2H), 7.40-7.33 (m, 1H), 7.23-7.05 (m, 2H), 6.51 (s, 1H), 5.57-5.48 (m, 1H), 3.26 (s, 3H), 2.16-2.01 (m, 2H), 0.80 (t, $J$ = 7.2 Hz, 3H).

**4-({(2*S*)-2-[4-{5-Chloro-2-[4-(difluoromethyl)-1*H*-1,2,3-triazol-1-yl]phenyl}-5-methoxy-2-oxopyridin-1(2*H*)-yl]butanoyl}amino)-2-fluorobenzamide (eutomer 79).**

Enantiomer separation of 268 mg of 4-({2-[4-{5-chloro-2-[4-(difluoromethyl)-1*H*-1,2,3-triazol-1-yl]phenyl}-5-methoxy-2-oxopyridin-1(2*H*)-yl]butanoyl}amino)-2-fluorobenzamide (racemate **331**) gave 104 mg of distomer (chiral HPLC: $t_R$ = 0.79 min) and 106 mg of eutomer **79** (chiral HPLC: $t_R$ =

2.10 min, >99% ee).

LC/MS (method 3): $t_R$ = 1.58 min, MS (ESIpos): *m*/*z* = 575 $[M+H]^+$; $^1$H NMR (400 MHz, DMSO-$d_6$): δ [ppm] = 10.77 (br s, 1H), 8.72 (s, 1H), 7.82-7.72 (m, 3H), 7.72-7.60 (m, 2H), 7.57-7.47 (m, 2H), 7.40-7.33 (m, 1H), 7.24-7.06 (m, 2H), 6.51 (s, 1H), 5.57-5.48 (m, 1H), 3.26 (s, 3H), 2.16-2.00 (m, 2H), 0.80 (t, *J* = 7.2 Hz, 3H).

Separation method: SFC: column: Chiralpak AS-H 5 μm, 250 mm × 20 mm; mobile phase: 70% carbon dioxide / 30% ethanol; temperature: 40 °C; flow rate: 80 mL/min; UV detection: 210 nm.

Analytical method: SFC: column: Chiralpak AS-H 3 μm, 100 mm × 4.6 mm; mobile phase: 70% carbon dioxide / 30% ethanol; temperature: 40 °C; flow rate: 3 mL/min; UV detection: 210 nm.

**Synthesis of eutomer 81.**[a]

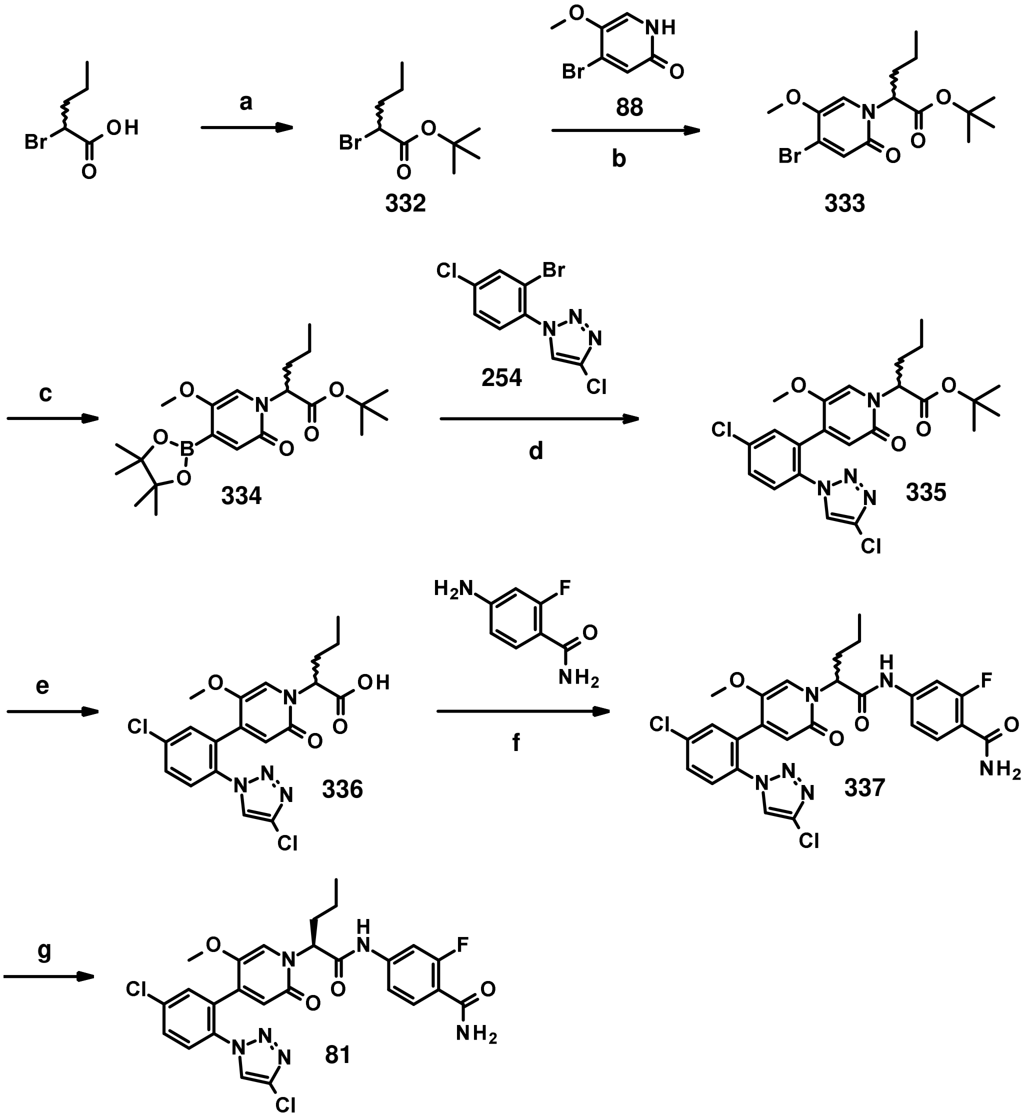

[a]Reagents and conditions: (a) $HClO_4$, *tert*-butyl acetate, RT, 81%; (b) $K_2CO_3$, DMF, 50 °C, 53%; (c) $(BPin)_2$, Pd(dppf)$Cl_2$-DCM complex, KOAc, 1,4-dioxane, 80 °C, 100%; (d) Pd(dppf)$Cl_2$-DCM complex, aq. $Na_2CO_3$, 1,4-dioxane, 100 °C, 43%; (e) HCl/1,4-dioxane, RT, 100%; (f) T3P, pyridine, 50 °C, 75%; (g) enantiomer separation.

***tert*-Butyl 2-bromopentanoate (racemate 332).**

Perchloric acid (71 $\mu$L, 70% purity, 0.83 mmol, 0.05 eq.) was added at RT to a solution of racemic 2-bromopentanoic acid (3.00 g, 16.57 mmol) in *tert*-butyl acetate (56 mL, 410 mmol, 25 eq.). The reaction mixture was stirred at RT overnight. After addition of water and phase separation, the organic phase was washed with aqueous sodium carbonate solution (5%, 50 mL) and water (20 mL) and dried over anhydrous sodium sulfate, filtered and concentrated under reduced pressure to give **332** which was used without further purification. Yield: 3.40 g (94% purity, 81% of theory). GC/MS (method 9): $t_R$ = 2.89 min, MS (EI): $m/z$ = 221 $[M-15]^+$.

***tert*-Butyl 2-(4-bromo-5-methoxy-2-oxopyridin-1(2*H*)-yl)pentanoate (racemate 333).**

Potassium carbonate (3.28 g, 23.72 mmol, 1.5 eq.) and *tert*-butyl 2-bromopentanoate (racemate **332**) (5.00 g, 90% purity, 18.98 mmol, 1.2 eq.) were added under argon atmosphere at RT to a solution of 4-bromo-5-methoxypyridin-2(1*H*)-one (**88**) (3.40 g, 15.81 mmol) in *N,N*-dimethylformamide (70 mL). The reaction mixture was stirred at 50 °C for 70 min. *N,N*-Dimethylformamide was removed at 40 °C *in vacuo*. After addition of ethyl acetate / water and phase separation, the aqueous phase was extracted with ethyl acetate. The combined organic phases were washed with water and brine, dried over anhydrous sodium sulfate, filtered and concentrated under reduced pressure. The residue was purified by flash silica gel chromatography (cyclohexane / ethyl acetate gradient) to give **333**. Yield: 3.10 g (53% of theory). LC/MS (method 3): $t_R$ = 1.93 min, MS (ESIpos): *m/z* = 360 $[M+H]^+$; $^1$H NMR (400 MHz, DMSO-$d_6$): δ [ppm] = 7.36 (s, 1H), 6.85 (s, 1H), 5.05 (dd, *J* = 10. 1 Hz, 5.3 Hz, 1H), 3.72 (s, 3H), 2.13-1.94 (m, 2H), 1.38 (s, 9H), 1.27-1.09 (m, 2H), 0.86 (t, *J* = 7.3 Hz, 3H).

***tert*-Butyl 2-[5-methoxy-2-oxo-4-(4,4,5,5-tetramethyl-1,3,2-dioxaborolan-2-yl)pyridin-1(2*H*)-yl]pentanoate (racemate 334).**

A mixture of *tert*-butyl 2-(4-bromo-5-methoxy-2-oxopyridin-1(2*H*)-yl)pentanoate (racemate **333**) (1.55 g, 4.22 mmol), bis(pinacolato)-diboron (1.18 g, 4.64 mmol, 1.1 eq.) and potassium acetate (1.24 g, 12.67 mmol, 3.0 eq.) in 1,4-dioxane (42 mL) was flushed with argon at RT for 5 min, before [1,1'-bis(diphenylphosphino)ferrocene]dichloropalladium-dichloromethane complex (207 mg, 0.25 mmol, 0.06 eq.) was added. The reaction mixture was stirred at 80 °C overnight, cooled to RT and filtered through Celite®, and the filter residue was washed with 1,4-dioxane. The combined filtrates were concentrated under reduced pressure. The residue was dried at 40 °C under high vacuum to give **334** which was used without further purification. Yield: 3.02 g (57% purity, 100% of theory).

***tert*-Butyl 2-{4-[5-chloro-2-(4-chloro-1*H*-1,2,3-triazol-1-yl)phenyl]-5-methoxy-2-oxopyridin-1(2*H*)-yl}pentanoate (racemate 335).**

A mixture of *tert*-butyl 2-[5-methoxy-2-oxo-4-(4,4,5,5-tetramethyl-1,3,2-dioxaborolan-2-yl)pyridin-1(2*H*)-yl]pentanoate (racemate **334**) (815 mg, 50% purity, 1.00 mmol), 1-(2-bromo-4-chlorophenyl)-4-chloro-1*H*-1,2,3-triazole (**254**) (299 mg, 1.00 mmol, 1.0 eq.) and [1,1'-bis(diphenylphosphino)-ferrocene]dichloropalladium-dichloromethane complex (82 mg, 0.10 mmol, 0.1 eq.) was flushed with argon at RT for 10 min. 1,4-dioxane (10 mL) and aqueous sodium carbonate solution (2 M, 1.50 mL, 3.00 mmol) were added. The reaction mixture was stirred at 100 °C for 2 h, cooled to RT and filtered through Celite®, the filter residue was washed with dichloromethane and acetonitrile. The combined filtrates were concentrated under reduced pressure. The residue was purified by flash silica gel chromatography (cyclohexane / ethyl acetate gradient) to give **335**. Yield: 211 mg (43% of theory). LC/MS (method 3): $t_R$ = 2.12 min, MS (ESIpos): *m/z* = 493 $[M+H]^+$; $^1$H NMR (400 MHz, DMSO-$d_6$): δ [ppm] = 8.57 (s, 1H), 7.81-7.70 (m, 3H), 7.07 (s, 1H), 6.42 (s, 1H), 5.07-4.99 (m, 1H), 3.27 (s, 3H),

2.11-1.90 (m, 2H), 1.39 (s, 9H), 1.22-1.08 (m, 2H), 0.87 (t, *J* = 7.3 Hz, 3H).

**2-{4-[5-Chloro-2-(4-chloro-1*H*-1,2,3-triazol-1-yl)phenyl]-5-methoxy-2-oxopyridin-1(2*H*)-yl}-pentanoic acid (racemate 336).**

*tert*-Butyl 2-{4-[5-chloro-2-(4-chloro-1*H*-1,2,3-triazol-1-yl)phenyl]-5-methoxy-2-oxopyridin-1(2*H*)-yl}pentanoate (racemate **335**) (211 mg, 0.43 mmol) in hydrogen chloride solution (4 M in 1,4-dioxane, 4.3 mL) was stirred at RT overnight and concentrated under reduced pressure. The residue was dried *in vacuo* to give **336** which was used without further purification. Yield: 188 mg (100% of theory). LC/MS (method 3): $t_R$ = 1.55 min, MS (ESIpos): *m*/*z* = 437 $[M+H]^+$; $^1$H NMR (400 MHz, DMSO-$d_6$): δ [ppm] = 12.93 (s, 1H), 8.55 (s, 1H), 7.81-7.70 (m, 3H), 7.11 (s, 1H), 6.42 (s, 1H), 5.23-5.00 (m, 1H), 3.26 (s, 3H), 2.17-1.92 (m, 2H), 1.19-1.04 (m, 2H), 0.87 (t, *J* = 7.3 Hz, 3H).

**4-{[2-{4-[5-Chloro-2-(4-chloro-1*H*-1,2,3-triazol-1-yl)phenyl]-5-methoxy-2-oxopyridin-1(2*H*)-yl}pentanoyl]amino}-2-fluorobenzamide (racemate 337).**

Propylphosphonic anhydride (T3P, 50% solution in ethyl acetate, 137 µL, 0.23 mmol, 3.0 eq.) was added dropwise under argon atmosphere at RT to a solution of 2-{4-[5-chloro-2-(4-chloro-1*H*-1,2,3-triazol-1-yl)phenyl]-5-methoxy-2-oxopyridin-1(2*H*)-yl}pentanoic acid (racemate **336**) (35 mg, 0.77 mmol) and 4-amino-2-fluorobenzamide (18 mg, 0.12 mmol, 1.5 eq.) in pyridine (1.0 mL). The reaction mixture was stirred at 50 °C for 1 h, cooled to RT and purified by preparative RP-HPLC (acetonitrile / 0.1% formic acid in water gradient) to give **337**. Yield: 33 mg (75% of theory). LC/MS (method 3): $t_R$ = 1.70 min, MS (ESIpos): *m*/*z* = 573 $[M+H]^+$; $^1$H NMR (400 MHz, DMSO-$d_6$): δ [ppm] = 10.78 (s, 1H), 8.59 (s, 1H), 7.81-7.72 (m, 3H), 7.72-7.61 (m, 2H), 7.57-7.49 (m, 2H), 7.39 (dd, *J* = 8.5 Hz, 1.5 Hz, 1H), 7.18 (s, 1H), 6.47 (s, 1H), 5.64 (br dd, 1H), 3.31 (s, 3H), 2.13-1.97 (m, 2H), 1.27-1.12 (m, 2H), 0.91 (t, *J* = 7.3 Hz, 3H).

**4-{[(2*S*)-2-{4-[5-Chloro-2-(4-chloro-1*H*-1,2,3-triazol-1-yl)phenyl]-5-methoxy-2-oxopyridin-1(2*H*)-yl}pentanoyl]amino}-2-fluorobenzamide (eutomer 81).**

Enantiomer separation of 25 mg of 4-{[2-{4-[5-chloro-2-(4-chloro-1*H*-1,2,3-triazol-1-yl)phenyl]-5-methoxy-2-oxopyridin-1(2*H*)-yl}pentanoyl]amino}-2-fluorobenzamide (racemate **337**) gave 11 mg of distomer (chiral HPLC: $t_R$ = 4.6 min) and 10 mg of eutomer **81** (chiral HPLC: $t_R$ = 7.3 min, >99% ee).
LC/MS (method 1): $t_R$ = 0.92 min, MS (ESIpos): *m*/*z* = 573 $[M+H]^+$; $^1$H NMR (400 MHz, DMSO-$d_6$): δ [ppm] = 10.78 (s, 1H), 8.59 (s, 1H), 7.82-7.72 (m, 3H), 7.72-7.61 (m, 2H), 7.57-7.49 (m, 2H), 7.39 (dd, *J* = 8.7 Hz, 1.8 Hz, 1H), 7.18 (s, 1H), 6.47 (s, 1H), 5.64 (br dd, 1H), 3.31 (s, 3H), 2.13-1.97 (m, 2H), 1.27-1.12 (m, 2H), 0.91 (t, *J* = 7.3 Hz, 3H).
Separation method: SFC: column: Daicel Chiralpak AD-H 5 µm, 250 mm × 20 mm; mobile phase: 78% carbon dioxide / 22% ethanol; temperature: 40 °C; flow rate: 70 mL/min; UV detection: 210 nm.
Analytical method: SFC: column: Daicel Chiralpak AD-H 3 µm, 100 mm × 4.6 mm; mobile phase: 70% carbon dioxide / 30% ethanol; flow rate: 3 mL/min; UV detection: 210 nm.

**Synthesis of eutomer 82.**[a]

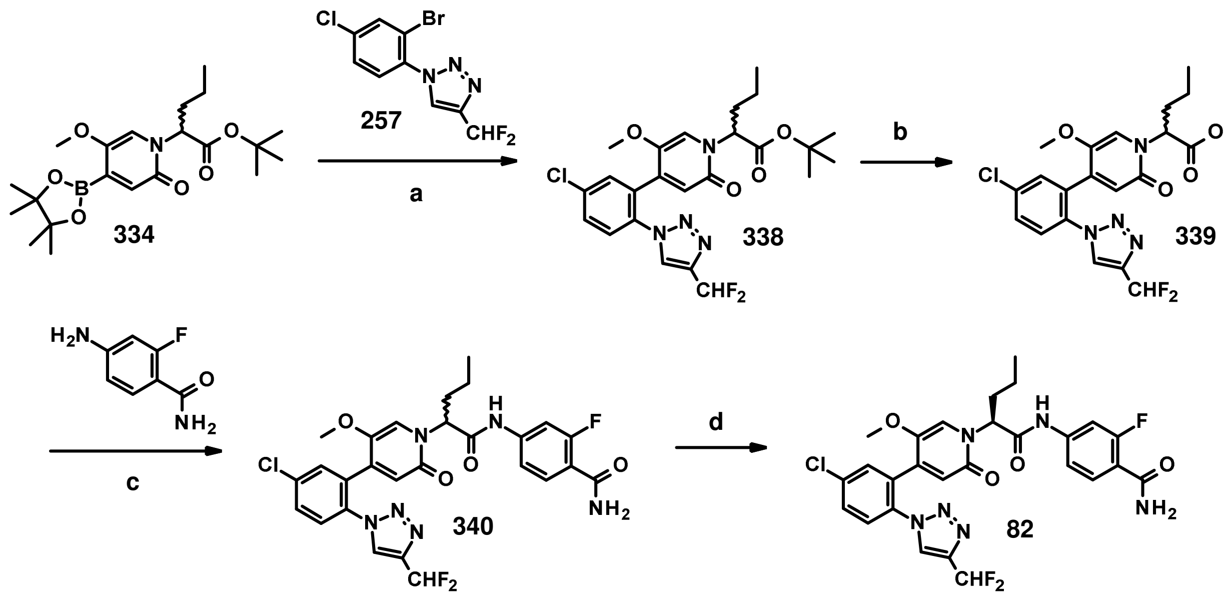

[a]Reagents and conditions: (a) Pd(dppf)$Cl_2$-DCM complex, aq. $Na_2CO_3$, 1,4-dioxane, 100 °C, 49%; (b) HCl/1,4-dioxane, RT, 97%; (c) T3P, pyridine, 50 °C, 84%; (d) enantiomer separation.

***tert*-Butyl 2-[4-{5-chloro-2-[4-(difluoromethyl)-1*H*-1,2,3-triazol-1-yl]phenyl}-5-methoxy-2-oxopyridin-1(2*H*)-yl]pentanoate (racemate 338).**

A mixture of *tert*-butyl 2-[5-methoxy-2-oxo-4-(4,4,5,5-tetramethyl-1,3,2-dioxaborolan-2-yl)pyridin-1(2*H*)-yl]pentanoate (racemate **334**) (754 mg, 57% purity, 1.06 mmol), 1-(2-bromo-4-chlorophenyl)-4-(difluoromethyl)-1*H*-1,2,3-triazole (**257**) (329 mg, 1.06 mmol, 1.0 eq.) and [1,1'-bis(diphenylphosphino)ferrocene]dichloropalladium-dichloromethane complex (86 mg, 0.11 mmol, 0.1 eq.) was flushed with argon at RT for 10 min, followed by addition of 1,4-dioxane (10.6 mL) and aqueous sodium carbonate solution (2 M, 1.57 mL, 3.15 mmol, 3.0 eq.). The reaction mixture was stirred in the microwave at 100 °C for 2 h, cooled to RT and filtered through Celite®, and the filter residue was washed with dichloromethane and acetonitrile. The combined filtrates were concentrated under reduced pressure. The residue was purified by flash silica gel chromatography (cyclohexane / ethyl acetate gradient) to give **338**. Yield: 270 mg (49% of theory). LC/MS (method 3): $t_R$ = 2.07 min, MS (ESIpos): $m/z$ = 509 $[M+H]^+$; $^1H$ NMR (400 MHz, DMSO-$d_6$): δ [ppm] = 8.70 (s, 1H), 7.81-7.71 (m, 3H), 7.20 (t, $J$ = 54.0 Hz, 1H), 7.03 (s, 1H), 6.46 (s, 1H), 5.06-4.98 (m, 1H), 3.22 (s, 3H), 2.10-1.88 (m, 2H), 1.38 (s, 9H), 1.22-1.05 (m, 2H), 0.86 (t, $J$ = 7.3 Hz, 3H).

**2-[4-{5-Chloro-2-[4-(difluoromethyl)-1*H*-1,2,3-triazol-1-yl]phenyl}-5-methoxy-2-oxopyridin-1(2*H*)-yl]pentanoic acid (racemate 339).**

*tert*-Butyl 2-[4-{5-chloro-2-[4-(difluoromethyl)-1*H*-1,2,3-triazol-1-yl]phenyl}-5-methoxy-2-oxopyridin-1(2*H*)-yl]pentanoate (racemate **338**) (269 mg, 0.52 mmol) in hydrogen chloride solution

(4 M in 1,4-dioxane, 5.2 mL) was stirred at RT overnight and concentrated under reduced pressure. The residue was dried *in vacuo* to give **339** which was used without further purification. Yield: 236 mg (97% of theory). LC/MS (method 3): $t_R$ = 1.56 min, MS (ESIpos): *m*/*z* = 453 $[M+H]^+$; $^1H$ NMR (400 MHz, DMSO-$d_6$): δ [ppm] = 12.9 (br s, 1H), 8.67 (s, 1H), 7.81-7.71 (m, 3H), 7.20 (t, *J* = 54.1 Hz, 1H), 7.08 (s, 1H), 6.46 (s, 1H), 5.19-5.03 (m, 1H), 3.21 (s, 3H), 2.14-1.90 (m, 2H), 1.16-1.04 (m, 2H), 0.85 (t, *J* = 7.3 Hz, 3H).

**4-({2-[4-{5-Chloro-2-[4-(difluoromethyl)-1*H*-1,2,3-triazol-1-yl]phenyl}-5-methoxy-2-oxopyridin-1(2*H*)-yl]pentanoyl}amino)-2-fluorobenzamide (racemate 340).**

Propylphosphonic anhydride (T3P, 50% solution in ethyl acetate, 131 µL, 0.22 mmol, 3.0 eq.) was added dropwise under argon atmosphere at RT to a solution of 2-[4-{5-chloro-2-[4-(difluoromethyl)-1*H*-1,2,3-triazol-1-yl]phenyl}-5-methoxy-2-oxopyridin-1(2*H*)-yl]pentanoic acid (racemate **339**) (39 mg, 85% purity, 0.73 mmol) and 4-amino-2-fluorobenzamide (17 mg, 0.11 mmol, 1.5 eq.) in pyridine (1.0 mL). The reaction mixture was stirred at 50 °C for 1 h, cooled to RT and purified by preparative RP-HPLC (acetonitrile / 0.1% formic acid in water gradient) to give **340**. Yield: 36 mg (84% of theory). LC/MS (method 3): $t_R$ = 1.66 min, MS (ESIpos): *m*/*z* = 589 $[M+H]^+$; $^1H$ NMR (400 MHz, DMSO-$d_6$): δ [ppm] = 10.78 (s, 1H), 8.70 (s, 1H), 7.79 (s, 2H), 7.74 (s, 1H), 7.72-7.61 (m, 2H), 7.57-7.48 (m, 2H), 7.38 (dd, *J* = 8.5 Hz, 1.9 Hz, 1H), 7.21 (t, *J* = 54.0 Hz, 1H), 7.15 (s, 1H), 6.50 (s, 1H), 5.67-5.59 (m, 1H), 3.26 (s, 3H), 2.12-1.96 (m, 2H), 1.27-1.09 (m, 2H), 0.90 (t, *J* = 7.3 Hz, 3H).

**4-({(2*S*)-2-[4-{5-Chloro-2-[4-(difluoromethyl)-1*H*-1,2,3-triazol-1-yl]phenyl}-5-methoxy-2-oxopyridin-1(2*H*)-yl]pentanoyl}amino)-2-fluorobenzamide (eutomer 82).**

Enantiomer separation of 86 mg of 4-({2-[4-{5-chloro-2-[4-(difluoromethyl)-1*H*-1,2,3-triazol-1-yl]phenyl}-5-methoxy-2-oxopyridin-1(2*H*)-yl]pentanoyl}amino)-2-fluorobenzamide (racemate **340**) gave 39 mg of distomer (chiral HPLC: $t_R$ = 2.3 min) and 33 mg of eutomer **82** (chiral HPLC: $t_R$ = 12.6 min, >99% ee).

LC/MS (method 1): $t_R$ = 0.90 min, MS (ESIpos): *m*/*z* = 589 $[M+H]^+$; $^1H$ NMR (400 MHz, DMSO-$d_6$): δ [ppm] = 10.78 (s, 1H), 8.70 (s, 1H), 7.79 (s, 2H), 7.74 (s, 1H), 7.72-7.60 (m, 2H), 7.57-7.48 (m, 2H), 7.38 (dd, *J* = 8.5 Hz, 1.9 Hz, 1H), 7.21 (t, *J* = 54.0 Hz, 1H), 7.15 (s, 1H), 6.50 (s, 1H), 5.67-5.59 (m, 1H), 3.26 (s, 3H), 2.12-1.96 (m, 2H), 1.27-1.09 (m, 2H), 0.90 (t, *J* = 7.3 Hz, 3H).

Separation method: SFC: column: Daicel Chiralpak AS-H 5 µm, 250 mm × 20 mm; mobile phase: 70% carbon dioxide / 30% ethanol; temperature: 40 °C; flow rate: 80 mL/min; UV detection: 210 nm.

Analytical method: SFC: column: Daicel Chiralpak AS-H 3 µm, 100 mm × 4.6 mm; mobile phase: 70% carbon dioxide / 30% ethanol; flow rate: 3 mL/min; UV detection: 210 nm.

**Synthesis of eutomer 83.**[a]

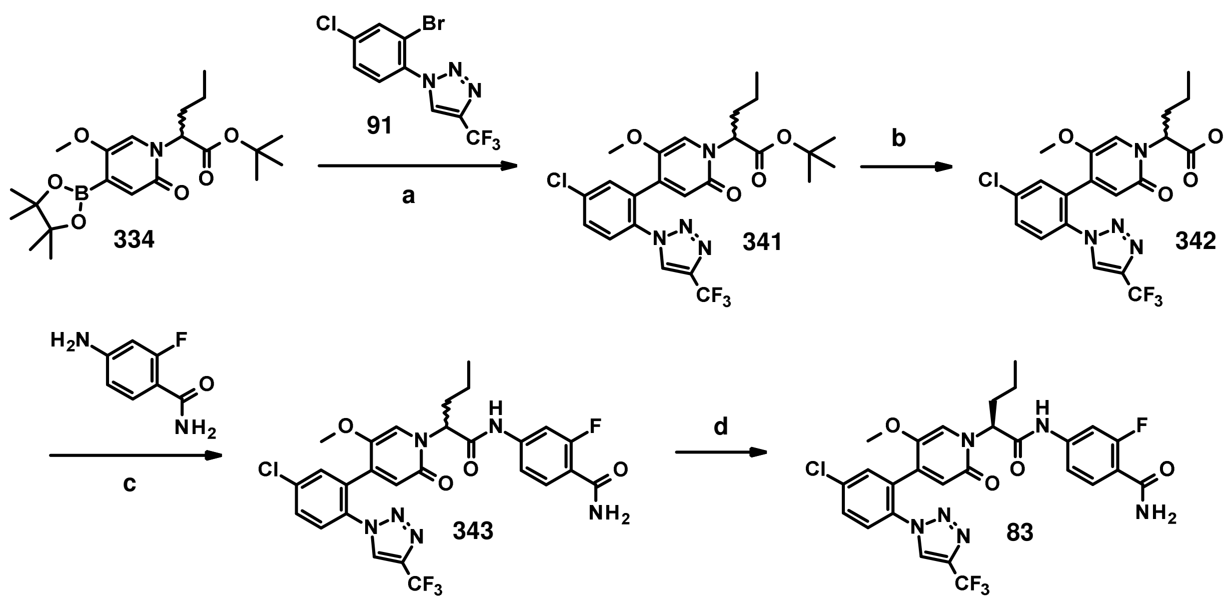

[a]Reagents and conditions: (a) Pd(dppf)$Cl_2$-DCM complex, aq. $Na_2CO_3$, 1,4-dioxane, 100 °C, 64%; (b) HCl/1,4-dioxane, RT, 100%; (c) T3P, pyridine, 40 °C, 67%; (d) enantiomer separation.

***tert*-Butyl 2-[4-{5-chloro-2-[4-(trifluoromethyl)-1*H*-1,2,3-triazol-1-yl]phenyl}-5-methoxy-2-oxopyridin-1(2*H*)-yl]pentanoate (341).**

A mixture of *tert*-butyl 2-[5-methoxy-2-oxo-4-(4,4,5,5-tetramethyl-1,3,2-dioxaborolan-2-yl)pyridin-1(2*H*)-yl]pentanoate (racemate **334**) (2.01 g, 62% purity, 3.06 mmol) and 1-(2-bromo-4-chlorophenyl)-4-(trifluoromethyl)-1*H*-1,2,3-triazole (**91**) (1.00 g, 3.06 mmol, 1.0 eq.) in 1,4-dioxane (30.7 mL) was flushed with argon at RT for 10 min, followed by addition of [1,1'-bis(diphenylphosphino)ferrocene]dichloropalladium-dichloromethane complex (250 mg, 0.31 mmol, 0.1 eq.) and aqueous sodium carbonate solution (2 M, 4.60 mL, 9.19 mmol, 3.0 eq.). The reaction mixture was stirred at 100 °C for 2 h, cooled to RT and filtered through Celite®, and the filter residue was washed with dichloromethane and acetonitrile. The combined filtrates were concentrated under reduced pressure. The residue was purified by flash silica gel chromatography (cyclohexane / ethyl acetate gradient) to give **341** which was used without further purification. Yield: 1.50 g (69% purity, 64% of theory). LC/MS (method 1): $t_R$ = 1.18 min, MS (ESIpos): *m*/*z* = 527 $[M+H]^+$.

**2-[4-{5-Chloro-2-[4-(trifluoromethyl)-1*H*-1,2,3-triazol-1-yl]phenyl}-5-methoxy-2-oxopyridin-1(2*H*)-yl]pentanoic acid (342).**

A mixture of *tert*-butyl 2-[4-{5-chloro-2-[4-(trifluoromethyl)-1*H*-1,2,3-triazol-1-yl]phenyl}-5-methoxy-2-oxopyridin-1(2*H*)-yl]pentanoate (**341**) (1.50 g, 69% purity, 1.96 mmol) in hydrogen chloride solution (4 M in 1,4-dioxane, 30.65 mL) was stirred at RT for 48 h and concentrated under reduced pressure to give **342** which was used without further purification. Yield: 1.50 g (71% purity, 100% of

theory). LC/MS (method 1): $t_R$ = 0.94 min, MS (ESIpos): *m*/*z* = 471 [M+H]$^+$.

**4-({2-[4-{5-Chloro-2-[4-(trifluoromethyl)-1*H*-1,2,3-triazol-1-yl]phenyl}-5-methoxy-2-oxopyridin-1(2*H*)-yl]pentanoyl}amino)-2-fluorobenzamide (racemate 343).**

Propylphosphonic anhydride (T3P, 50% solution in ethyl acetate, 430 µL, 0.72 mmol, 1.6 eq.) was added under argon atmosphere at RT to a solution of 2-[4-{5-chloro-2-[4-(trifluoromethyl)-1*H*-1,2,3-triazol-1-yl]phenyl}-5-methoxy-2-oxopyridin-1(2*H*)-yl]pentanoic acid (racemate **342**) (300 mg, 71% purity, 0.45 mmol) in pyridine (2.4 mL). The reaction mixture was heated to 40 °C, and 4-amino-2-fluorobenzamide (91 mg, 0.59 mmol, 1.3 eq.) was added. The reaction mixture was stirred at 40 °C for additional 10 min, cooled to RT, diluted with *N*,*N*-dimethylformamide and purified by preparative RP-HPLC (acetonitrile / 0.1% formic acid in water gradient) to give **343**. Yield: 184 mg (67% of theory). LC/MS (method 3): $t_R$ = 1.81 min, MS (ESIpos): *m*/*z* = 607 [M+H]$^+$; $^1$H NMR (400 MHz, DMSO-$d_6$): δ [ppm] = 10.76 (br s, 1H), 9.11 (s, 1H), 7.87-7.80 (m, 2H), 7.79-7.77 (m, 1H), 7.72-7.60 (m, 2H), 7.57-7.49 (m, 2H), 7.37 (dd, *J* = 8.5 Hz, 1.9 Hz, 1H), 7.14 (s, 1H), 6.52 (s, 1H), 5.68-5.59 (m, 1H), 3.26 (s, 3H), 2.15-1.95 (m, 2H), 1.23-1.09 (m, 2H), 0.89 (t, *J* = 7.3 Hz, 3H).

**4-({(2*S*)-2-[4-{5-Chloro-2-[4-(trifluoromethyl)-1*H*-1,2,3-triazol-1-yl]phenyl}-5-methoxy-2-oxopyridin-1(2*H*)-yl]pentanoyl}amino)-2-fluorobenzamide (eutomer 83).**

Enantiomer separation of 177 mg of 4-({2-[4-{5-chloro-2-[4-(trifluoromethyl)-1*H*-1,2,3-triazol-1-yl]phenyl}-5-methoxy-2-oxopyridin-1(2*H*)-yl]pentanoyl}amino)-2-fluorobenzamide (racemate **343**) gave 82 mg of distomer (chiral HPLC: $t_R$ = 1.15 min) and 72 mg of eutomer **83** (chiral HPLC: $t_R$ = 1.75 min, >99% ee).

LC/MS (method 3): $t_R$ = 1.81 min, MS (ESIpos): *m*/*z* = 607 [M+H]$^+$; $^1$H NMR (400 MHz, DMSO-$d_6$): δ [ppm] = 10.77 (br s, 1H), 9.11 (s, 1H), 7.87-7.80 (m, 2H), 7.79-7.77 (m, 1H), 7.72-7.60 (m, 2H), 7.57-7.49 (m, 2H), 7.38 (dd, *J* = 8.5 Hz, 1.9 Hz, 1H), 7.14 (s, 1H), 6.52 (s, 1H), 5.68-5.59 (m, 1H), 3.26 (s, 3H), 2.15-1.95 (m, 2H), 1.23-1.09 (m, 2H), 0.89 (t, *J* = 7.3 Hz, 3H).

Separation method: SFC: column: Daicel Chiralpak AD-H 5 µm, 250 mm × 20 mm; mobile phase: 75% carbon dioxide / 25% ethanol; temperature: 40 °C; flow rate: 80 mL/min; UV detection: 210 nm.

Analytical method: SFC: column: Daicel Chiralpak AD-H 3 µm, 100 mm × 4.6 mm; mobile phase: 75% carbon dioxide / 25% ethanol; flow rate: 3 mL/min; UV detection: 210 nm.

**Synthesis of compound 344.**[a]

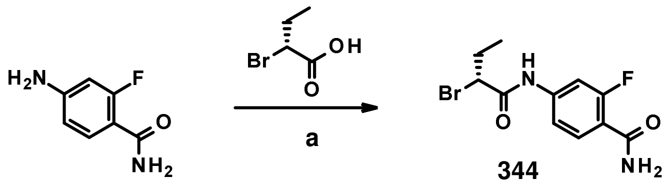


[a]Reagents and conditions: (a) T3P, pyridine, THF, 0 °C → RT, 90%.

**4-{[(2*R*)-2-Bromobutanoyl]amino}-2-fluorobenzamide (344).**

(2*R*)-2-Bromobutanoic acid (3.54 g, 21.19 mmol, 1.1 eq.), pyridine (1.71 mL, 21.19 mmol, 1.1 eq.) and propylphosphonic anhydride (T3P, 50% solution in ethyl acetate, 17.21 mL, 28.90 mmol, 1.5 eq.) were added under argon atmosphere at 0-5 °C to a suspension of 4-amino-2-fluorobenzamide (3.00 g, 19.27 mmol) in tetrahydrofuran (30 mL). The reaction mixture was allowed to warm to RT, stirred for 30 min, cooled to 10 °C, mixed dropwise with water (35 mL), stirred for 15 min, mixed with additional water (25 mL) and stirred for 30 min. The forming precipitate was filtered, washed with water and dried *in vacuo* to give a first batch of **344**. Yield: 5.23 g (90% of theory). From the combined filtrates, further precipitate formed which was filtered, washed with water and dried *in vacuo* to give a second batch of **344**. Yield: 0.50 g (9% of theory). LC/MS (method 3): $t_R$ = 1.25 min, MS (ESIpos): *m*/*z* = 303 $[M+H]^+$; $^1H$ NMR (400 MHz, DMSO-$d_6$): δ [ppm] = 10.70 (br s, 1H), 7.70 (t, *J* = 8.5 Hz, 1H), 7.65 (dd, *J* = 13.3 Hz, 2.0 Hz, 1H), 7.55 (br s, 1H), 7.52 (br s, 1H), 7.35 (dd, *J* = 8.6 Hz, 2.0 Hz, 1H), 4.46 (t, *J* = 7.3 Hz, 1H), 2.16-2.03 (m, 1H), 2.01-1.88 (m, 1H), 0.96 (t, *J* = 7.3 Hz, 3H).

**Synthesis of eutomer 84.**[a]

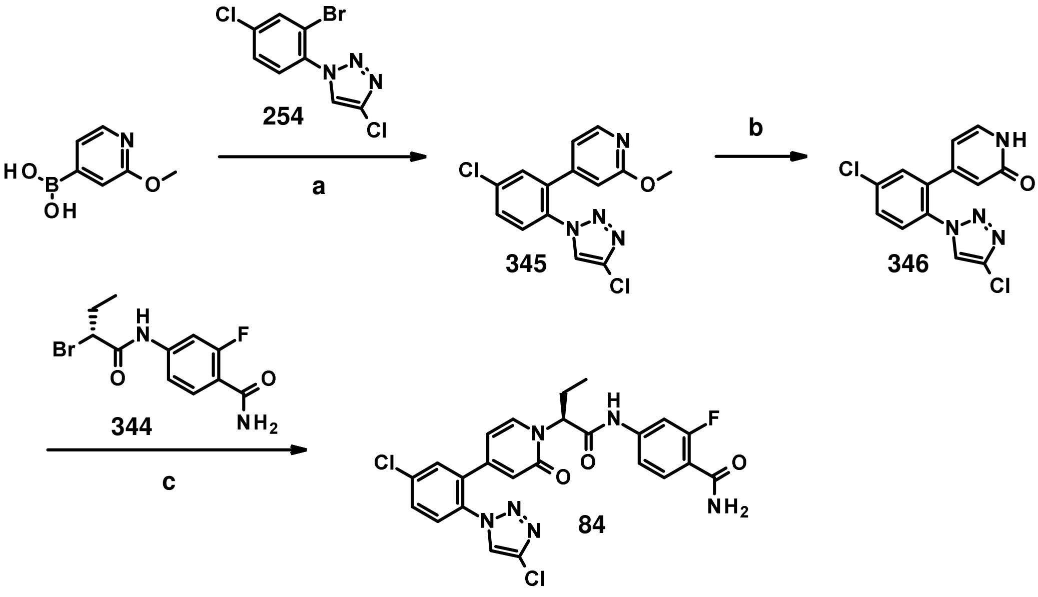


[a]Reagents and conditions: (a) Pd(dppf)$Cl_2$-DCM complex, $K_2CO_3$, 1,4-dioxane, 110 °C, 74%; (b) pyridine hydrobromide, DMF, 100 °C, 87%; (c) 1,1,3,3-tetramethylguanidine, 2-propanol/acetone, RT, 77%.

**4-[5-Chloro-2-(4-chloro-1*H*-1,2,3-triazol-1-yl)phenyl]-2-methoxypyridine (345).**

A mixture of (2-methoxypyridin-4-yl)boronic acid (76 mg, 0.50 mmol), 1-(2-bromo-4-chlorophenyl)-4-chloro-1*H*-1,2,3-triazole (**254**) (146 mg, 0.50 mmol, 1.0 eq.) and potassium carbonate (207 mg, 1.50 mmol, 3.0 eq.) in 1,4-dioxane (10 mL) was flushed with argon at RT for 10 min, followed by addition of [1,1'-bis(diphenylphosphino)ferrocene]dichloropalladium-dichloromethane complex (31 mg, 0.04 mmol, 0.075 eq.). The reaction mixture was stirred at 110 °C for 3 h, cooled to RT, mixed with additional (2-methoxypyridin-4-yl)boronic acid (76 mg, 0.50 mmol) and two drops of water, stirred at 110 °C for 3 h, cooled to RT and filtered through Celite®. The filter residue was washed with 1,4-dioxane. The combined filtrates were concentrated under reduced pressure. The residue was purified by flash silica gel chromatography (cyclohexane / ethyl acetate gradient) to give **345**. Yield: 126 mg (94% purity, 74% of theory). LC/MS (method 3): $t_R$ = 1.96 min, MS (ESIpos): *m*/*z* = 321 $[M+H]^+$; $^1H$ NMR (600 MHz, DMSO-$d_6$): δ [ppm] = 8.62 (s, 1H), 8.09 (d, *J* = 5.3 Hz, 1H), 7.82-7.73 (m, 3H), 6.65 (s, 1H), 6.62 (d, *J* = 5.1 Hz, 1H), 3.84 (s, 3H).

**4-[5-Chloro-2-(4-chloro-1*H*-1,2,3-triazol-1-yl)phenyl]pyridin-2(1*H*)-one (346).**

Pyridine hydrobromide (590 mg, 3.69 mmol, 10 eq.) was added at RT to a mixture of 4-[5-chloro-2-(4-chloro-1*H*-1,2,3-triazol-1-yl)phenyl]-2-methoxypyridine (**345**) (126 mg, 0.37 mmol) in *N*,*N*-dimethylformamide (4 mL). The reaction mixture was stirred at 100 °C for 45 min and concentrated under reduced pressure. The residue was stirred in water, and the solid was filtered, washed with water

and dried *in vacuo* to give **346** which was used without further purification. Yield: 101 mg (87% of theory). LC/MS (method 3): $t_R$ = 1.25 min, MS (ESIpos): $m/z$ = 307 $[M+H]^+$; $^1H$ NMR (600 MHz, DMSO-$d_6$): δ [ppm] = 11.65 (br s, 1H), 8.68 (s, 1H), 7.80-7.70 (m, 3H), 7.29 (d, $J$ = 6.7 Hz, 1H), 6.18 (s, 1H), 5.73 (d, $J$ = 6.9 Hz, 1H).

**4-{[(2*S*)-2-{4-[5-Chloro-2-(4-chloro-1*H*-1,2,3-triazol-1-yl)phenyl]-2-oxopyridin-1(2*H*)-yl}butanoyl]amino}-2-fluorobenzamide (eutomer 84).**

1,1,3,3-Tetramethylguanidine (120 µL, 0.96 mmol, 3.0 eq.) was added under argon atmosphere at RT to a solution of 4-[5-chloro-2-(4-chloro-1*H*-1,2,3-triazol-1-yl)phenyl]pyridin-2(1*H*)-one (**346**) (100 mg, 0.32 mmol) in a mixture of 2-propanol (1 mL) and acetone (0.25 mL). The reaction mixture was stirred at RT for 15 min, mixed with 4-{[(2*R*)-2-bromobutanoyl]amino}-2-fluorobenzamide (**344**) (106 mg, 0.35 mmol, 1.1 eq.) and additional 2-propanol (1 mL) and acetone (0.25 mL) and stirred at RT overnight. The suspension was diluted with additional 2-propanol (2 mL) and acetone (0.5 mL) and mixed with additional 1,1,3,3-tetramethylguanidine (120 µL, 0.96 mmol, 3.0 eq.). The solution was stirred at RT overnight and concentrated under reduced pressure. The residue was purified by preparative RP-HPLC (acetonitrile / water gradient) to give **84**. Yield: 130 mg (77% of theory). LC/MS (method 22): $t_R$ = 0.89 min, MS (ESIpos): $m/z$ = 529 $[M+H]^+$; $^1H$ NMR (600 MHz, DMSO-$d_6$): δ [ppm] = 10.80 (br s, 1H), 8.67 (s, 1H), 7.83-7.78 (m, 2H), 7.76 (d, $J$ = 8.2 Hz, 1H), 7.71-7.66 (m, 2H), 7.63 (d, $J$ = 13.1 Hz, 1H), 7.53 (br s, 1H), 7.51 (br s, 1H), 7.36 (d, $J$ = 8.6 Hz, 1H), 6.34 (d, $J$ = 1.6 Hz, 1H), 5.88 (dd, $J$ = 7.2 Hz, 1.6 Hz, 1H), 5.49 (dd, $J$ = 10.2 Hz, 5.7 Hz, 1H), 2.16-2.07 (m, 1H), 2.03-1.94 (m, 1H), 0.81 (t, $J$ = 7.2 Hz, 3H).

**Synthesis of eutomer 85.**[a]

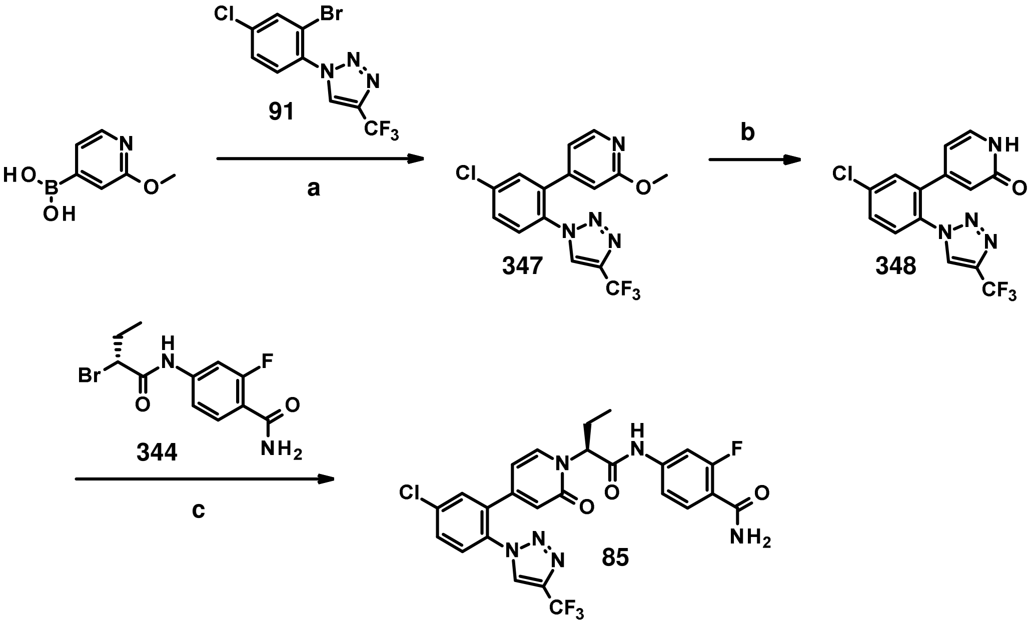


[a]Reagents and conditions: (a) Pd(dppf)$Cl_2$-DCM complex, $K_2CO_3$, 1,4-dioxane, 110 °C, 67%; (b) pyridine hydrobromide, DMF, 100 °C, 76%; (c) 1,1,3,3-tetramethylguanidine, 2-propanol/acetone, RT, 82%.

**4-{5-Chloro-2-[4-(trifluoromethyl)-1*H*-1,2,3-triazol-1-yl]phenyl}-2-methoxypyridine (347).**

A mixture of (2-methoxypyridin-4-yl)boronic acid (76 mg, 0.50 mmol), 1-(2-bromo-4-chlorophenyl)-4-(trifluoromethyl)-1*H*-1,2,3-triazole (**91**) (163 mg, 0.50 mmol, 1.0 eq.) and potassium carbonate (207 mg, 1.50 mmol, 3.0 eq.) in 1,4-dioxane (10 mL) was flushed with argon at RT for 10 min, followed by addition of [1,1'-bis(diphenylphosphino)ferrocene]dichloropalladium-dichloromethane complex (31 mg, 0.04 mmol, 0.075 eq.). The reaction mixture was stirred at 110 °C for 3.5 h, cooled to RT and filtered through Celite®, and the filter residue was washed with in 1,4-dioxane. The combined filtrates were concentrated under reduced pressure. The residue was purified by flash silica gel chromatography (cyclohexane / ethyl acetate gradient) to give **347**. Yield: 120 mg (67% of theory). LC/MS (method 3): $t_R$ = 2.09 min, MS (ESIpos): $m/z$ = 355 $[M+H]^+$; $^1H$ NMR (600 MHz, DMSO-$d_6$): δ [ppm] = 9.15 (s, 1H), 8.07 (d, $J$ = 5.3 Hz, 1H), 7.86-7.79 (m, 3H), 6.64 (s, 1H), 6.60 (d, $J$ = 5.3 Hz, 1H), 3.83 (s, 3H).

**4-{5-Chloro-2-[4-(trifluoromethyl)-1*H*-1,2,3-triazol-1-yl]phenyl}pyridin-2(1*H*)-one (348).**

Pyridine hydrobromide (536 mg, 3.35 mmol, 10 eq.) was added at RT to a mixture of 4-{5-chloro-2-[4-(trifluoromethyl)-1*H*-1,2,3-triazol-1-yl]phenyl}-2-methoxypyridine (**347**) (120 mg, 0.34 mmol) in *N,N*-dimethylformamide (4 mL). The reaction mixture was stirred at 100 °C for 1 h and concentrated under reduced pressure. The residue was stirred in water, and the solid was filtered, washed with water and dried *in vacuo* to give **348** which was used without further purification. Yield: 89 mg (76% of theory). LC/MS (method 3): $t_R$ = 1.43 min, MS (ESIpos): $m/z$ = 341 $[M+H]^+$; $^1H$ NMR (600 MHz, DMSO-$d_6$):

δ [ppm] = 11.64 (br s, 1H), 9.20 (s, 1H), 7.84-7.75 (m, 3H), 7.28 (d, *J* = 6.9 Hz, 1H), 6.18 (s, 1H), 5.72 (d, *J* = 6.7 Hz, 1H).

**4-({(2*S*)-2-[4-{5-Chloro-2-[4-(trifluoromethyl)-1*H*-1,2,3-triazol-1-yl]phenyl}-2-oxopyridin-1(2*H*)-yl]butanoyl}amino)-2-fluorobenzamide (eutomer 85).**

1,1,3,3-Tetramethylguanidine (94 μL, 0.75 mmol, 3.0 eq.) was added under argon atmosphere at RT to a solution of 4-{5-chloro-2-[4-(trifluoromethyl)-1*H*-1,2,3-triazol-1-yl]phenyl}pyridin-2(1*H*)-one (**348**) (87 mg, 0.25 mmol) in a mixture of 2-propanol (0.8 mL) and acetone (0.2 mL). The reaction mixture was stirred at RT for 15 min, mixed with 4-{[(2*R*)-2-bromobutanoyl]amino}-2-fluorobenzamide (**344**) (83 mg, 0.28 mmol, 1.1 eq.) and additional 2-propanol (0.8 mL) and acetone (0.2 mL) and stirred at RT overnight. The suspension was diluted with additional 2-propanol (1.6 mL) and acetone (0.4 mL) and mixed with additional 1,1,3,3-tetramethylguanidine (94 μL, 0.75 mmol, 3.0 eq.). The solution was stirred at RT overnight and concentrated under reduced pressure. The residue was purified by preparative RP-HPLC (acetonitrile / water gradient) to give **85**. Yield: 116 mg (82% of theory). LC/MS (method 22): $t_R$ = 0.94 min, MS (ESIpos): *m*/*z* = 562 $[M+H]^+$; $^1H$ NMR (600 MHz, DMSO-$d_6$): δ [ppm] = 10.7 (br s, 1H), 9.15 (s, 1H), 7.86-7.80 (m, 3H), 7.71-7.65 (m, 2H), 7.61 (d, *J* = 13.3 Hz, 1H), 7.52 (br s, 1H), 7.50 (br s, 1H), 7.34 (d, *J* = 8.4 Hz, 1H), 6.34 (d, *J* = 1.8 Hz, 1H), 5.84 (dd, *J* = 7.2 Hz, 1.8 Hz, 1H), 5.48 (dd, *J* = 10.6 Hz, 5.7 Hz, 1H), 2.15-2.06 (m, 1H), 2.02-1.92 (m, 1H), 0.77 (t, *J* = 7.2 Hz, 3H).

**Compounds of Endnote 39.**

**Synthesis of compound 355.**[a]

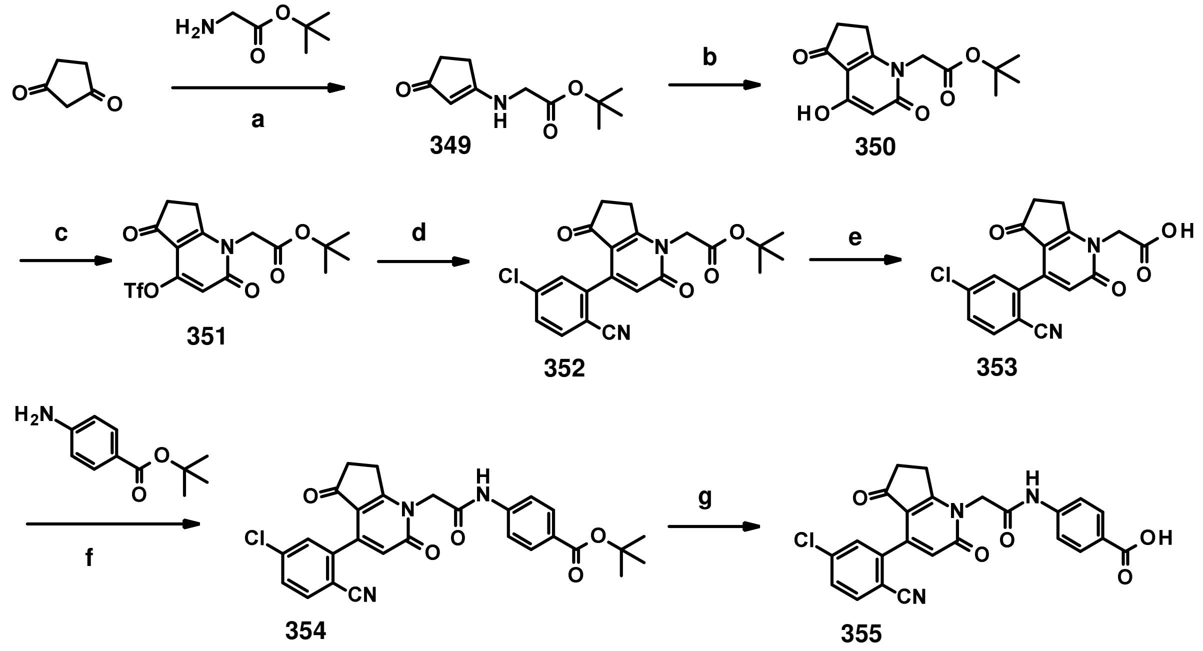

[a]Reagents and conditions: (a) p-TsOH × $H_2O$, toluene, RF, 82%; (b) bis(2,4,6-trichlorophenyl) malonate, diethyleneglycol dimethylether, 100 °C, 49%; (c) 1,1,1-trifluoro-*N*-phenyl-*N*-[(trifluoromethyl)sulfonyl]methanesulfonamide, $NEt_3$, DCM, 0 °C → RT, 55%; (d) (5-chloro-2-cyanophenyl)boronic acid, $Pd(Ph_3P)_4$, $K_2CO_3$, 1,4-dioxane, 110 °C, 50%; (e) TFA, DCM, RT, 90%; (f) HATU, DIEA, DMF, RT, 40%; (g) TFA, DCM, RT, 55%.

***tert*-Butyl *N*-(3-oxocyclopent-1-en-1-yl)glycinate (349).**

A solution of cyclopentane-1,3-dione (6.73 g, 68.61 mmol), *tert*-butyl glycinate (9.00 g, 68.61 mmol, 1.0 eq.) and 4-toluenesulfonic acid monohydrate (1.31 g, 6.86 mmol, 0.1 eq.) in toluene (350 mL) was stirred under reflux for 3 h (Dean-Stark water separator) and cooled to RT. Toluene was removed *in vacuo*. After addition water / dichloromethane and phase separation, the organic phase was washed with water and brine, dried over anhydrous sodium sulfate, filtered and concentrated under reduced pressure. The residue was stirred in cyclohexane, and the solid was filtered and dried *in vacuo* to give **349**. Yield: 11.90 g (82% of theory). LC/MS (method 1): $t_R$ = 0.58 min, MS (ESIpos): *m*/*z* = 212 $[M+H]^+$.

***tert*-Butyl (4-hydroxy-2,5-dioxo-2,5,6,7-tetrahydro-1*H*-cyclopenta[*b*]pyridin-1-yl)acetate (350).**

A solution of *tert*-butyl *N*-(3-oxocyclopent-1-en-1-yl)glycinate (**349**) (1.50 g, 7.10 mmol) and bis(2,4,6-trichlorophenyl) malonate (3.62 g, 7.81 mmol, 1.1 eq.) in diethyleneglycol dimethylether (20 mL) was stirred at 100 °C for 3 h, cooled to RT and evaporated under reduced pressure. The residue was stirred in diethyl ether (50 mL), and the solid was filtered, washed with diethyl ether and dried *in vacuo* to give **350**. Yield: 1.08 g (89% purity, 49% of theory). LC/MS (method 1): $t_R$ = 0.86 min, MS (ESIpos): *m*/*z* =

280 [M+H]$^+$; $^1$H NMR (400 MHz, DMSO-$d_6$): δ [ppm] = 11.17 (br s, 1H), 5.54 (s, 1H), 4.63 (s, 2H), 2.92-2.88 (m, 2H), 2.55-2.49 (m, 2H), 1.42 (s, 9H).

***tert*-Butyl (2,5-dioxo-4-{[(trifluoromethyl)sulfonyl]oxy}-2,5,6,7-tetrahydro-1*H*-cyclopenta[*b*]pyridin-1-yl)acetate (351).**

Triethylamine (580 µL, 4.18 mmol, 1.1 eq.) was added under argon atmosphere at 0 °C to a solution of *tert*-butyl (4-hydroxy-2,5-dioxo-2,5,6,7-tetrahydro-1*H*-cyclopenta[*b*]pyridin-1-yl)acetate (**350**) (1.06 g, 3.80 mmol) in dichloromethane (21 mL), followed by addition of 1,1,1-trifluoro-*N*-phenyl-*N*-[(trifluoromethyl)sulfonyl]methanesulfonamide (1.49 g, 4.18 mmol, 1.1 eq.) in portions. The reaction mixture was stirred at RT for 2 d and concentrated under reduced pressure. The residue was purified by flash silica gel chromatography (cyclohexane / ethyl acetate gradient) to give **351**. Yield: 950 mg (90% purity, 55% of theory). LC/MS (method 1): $t_R$ = 1.03 min, MS (ESIpos): *m*/*z* = 412 [M+H]$^+$; $^1$H NMR (400 MHz, DMSO-$d_6$): δ [ppm] = 6.64 (s, 1H), 4.76 (s, 2H), 3.10-3.07 (m, 2H), 2.73-2.69 (m, 2H), 1.43 (s, 9H).

***tert*-Butyl [4-(5-chloro-2-cyanophenyl)-2,5-dioxo-2,5,6,7-tetrahydro-1*H*-cyclopenta[*b*]pyridin-1-yl]acetate (352).**

A mixture of *tert*-butyl (2,5-dioxo-4-{[(trifluoromethyl)sulfonyl]oxy}-2,5,6,7-tetrahydro-1*H*-cyclopenta[*b*]pyridin-1-yl)acetate (**351**) (472 mg, 1.15 mmol), (5-chloro-2-cyanophenyl)boronic acid (239 mg, 1.32 mmol, 1.15 eq.) and potassium carbonate (478 mg, 3.44 mmol, 3.0 eq.) in 1,4-dioxane (15 mL) was flushed with argon at RT, followed by addition of tetrakis(triphenylphosphine)palladium (133 mg, 0.12 mmol, 0.1 eq.). The reaction mixture was stirred at 110 °C for 16 h, cooled to RT, filtered and concentrated under reduced pressure. The residue was purified by flash silica gel chromatography (cyclohexane / ethyl acetate gradient) to give **352**. Yield: 236 mg (50% of theory). LC/MS (method 1): $t_R$ = 1.00 min, MS (ESIneg): *m*/*z* = 397 [M-H]$^-$; $^1$H NMR (400 MHz, DMSO-$d_6$): δ [ppm] = 7.97 (d, *J* = 8.4 Hz, 1H), 7.74 (dd, *J* = 8.3 Hz, 2.1 Hz, 1H), 7.68 (d, *J* = 2.1 Hz, 1H), 6.49 (s, 1H), 4.81 (s, 2H), 3.10-3.05 (m, 2H), 2.64-2.60 (m, 2H), 1.45 (s, 9H).

**[4-(5-Chloro-2-cyanophenyl)-2,5-dioxo-2,5,6,7-tetrahydro-1*H*-cyclopenta[*b*]pyridin-1-yl]acetic acid (353).**

Trifluoroacetic acid (0.58 mL, 7.52 mmol, 20 eq.) was added at RT to a solution of *tert*-butyl [4-(5-chloro-2-cyanophenyl)-2,5-dioxo-2,5,6,7-tetrahydro-1*H*-cyclopenta[*b*]pyridin-1-yl]acetate (**352**) (150 mg, 0.38 mmol) in dichloromethane (9.5 mL). The reaction mixture was stirred at RT for 27 h and concentrated under reduced pressure. The residue was coevaporated three times with dichloromethane and dried *in vacuo* to give **353**. Yield: 120 mg (90% of theory). LC/MS (method 1): $t_R$ = 0.65 min, MS (ESIpos): *m*/*z* = 343 [M+H]$^+$; $^1$H NMR (400 MHz, DMSO-$d_6$): δ [ppm] = 13.4 (br s, 1H), 7.97 (d, *J* = 8.3 Hz, 1H), 7.74 (dd, *J* = 8.3 Hz, 2.1 Hz, 1H), 7.69 (d, *J* = 2.0 Hz, 1H), 6.49 (s, 1H), 4.82 (s, 2H), 3.13-3.07 (m, 2H), 2.64-2.59 (m, 2H).

***tert*-Butyl 4-{2-[4-(5-chloro-2-cyanophenyl)-2,5-dioxo-2,5,6,7-tetrahydro-1*H*-cyclopenta[*b*]pyridin-1-yl]acetamido}benzoate (354).**

A solution of HATU (120 mg, 0.32 mmol, 1.2 eq.) in *N,N*-dimethylformamide (1 mL) was added under argon atmosphere at RT to a solution of [4-(5-chloro-2-cyanophenyl)-2,5-dioxo-2,5,6,7-tetrahydro-1*H*-cyclopenta[*b*]pyridin-1-yl]acetic acid (**353**) (90 mg, 0.26 mmol), *tert*-butyl 4-aminobenzoate (61 mg, 0.32 mmol, 1.2 eq.) and *N,N*-diisopropylethylamine (137 $\mu$L, 0.79 mmol, 3.0 eq.) in *N,N*-dimethylformamide (3 mL). The reaction mixture was stirred at RT overnight and purified without further work-up by preparative RP-HPLC (acetonitrile / 0.05% formic acid in water gradient) to give **354**. Yield: 55 mg (40% of theory). LC/MS (method 1): $t_R$ = 1.12 min, MS (ESIneg): *m*/*z* = 516 $[M-H]^-$; $^1$H NMR (400 MHz, DMSO-$d_6$): δ [ppm] = 10.88 (s, 1H), 7.98 (d, *J* = 8.3 Hz, 1H), 7.89 (d, *J* = 8.7 Hz, 2H), 7.76-7.68 (m, 4H), 6.49 (s, 1H), 4.97 (s, 2H), 3.17-3.12 (m, 2H), 2.65-2.60 (m, 2H), 1.54 (s, 9H).

**4-{2-[4-(5-Chloro-2-cyanophenyl)-2,5-dioxo-2,5,6,7-tetrahydro-1*H*-cyclopenta[*b*]pyridin-1-yl]acetamido}benzoic acid (355).**

Trifluoroacetic acid (0.16 mL, 2.09 mmol, 20 eq.) was added at RT to a solution of *tert*-butyl 4-{2-[4-(5-chloro-2-cyanophenyl)-2,5-dioxo-2,5,6,7-tetrahydro-1*H*-cyclopenta[*b*]pyridin-1-yl]acetamido}-benzoate (**354**) (54 mg, 0.10 mmol) in dichloromethane (5.4 mL). The reaction mixture was stirred at RT overnight and concentrated under reduced pressure. The residue was coevaporated three times with dichloromethane and purified by preparative RP-HPLC (acetonitrile / 0.05% formic acid in water gradient) to give **355**. Yield: 27 mg (55% of theory). LC/MS (method 1): $t_R$ = 0.80 min, MS (ESIpos): *m*/*z* = 461 $[M+H]^+$; $^1$H NMR (400 MHz, DMSO-$d_6$): δ [ppm] = 12.77 (br s, 1H), 10.87 (s, 1H), 7.98 (d, *J* = 8.3 Hz, 1H), 7.92 (d, *J* = 8.6 Hz, 2H), 7.77-7.68 (m, 4H), 6.50 (s, 1H), 4.98 (s, 2H), 3.17-3.12 (m, 2H), 2.66-2.60 (m, 2H).

**Synthesis of racemate 362.**[a]

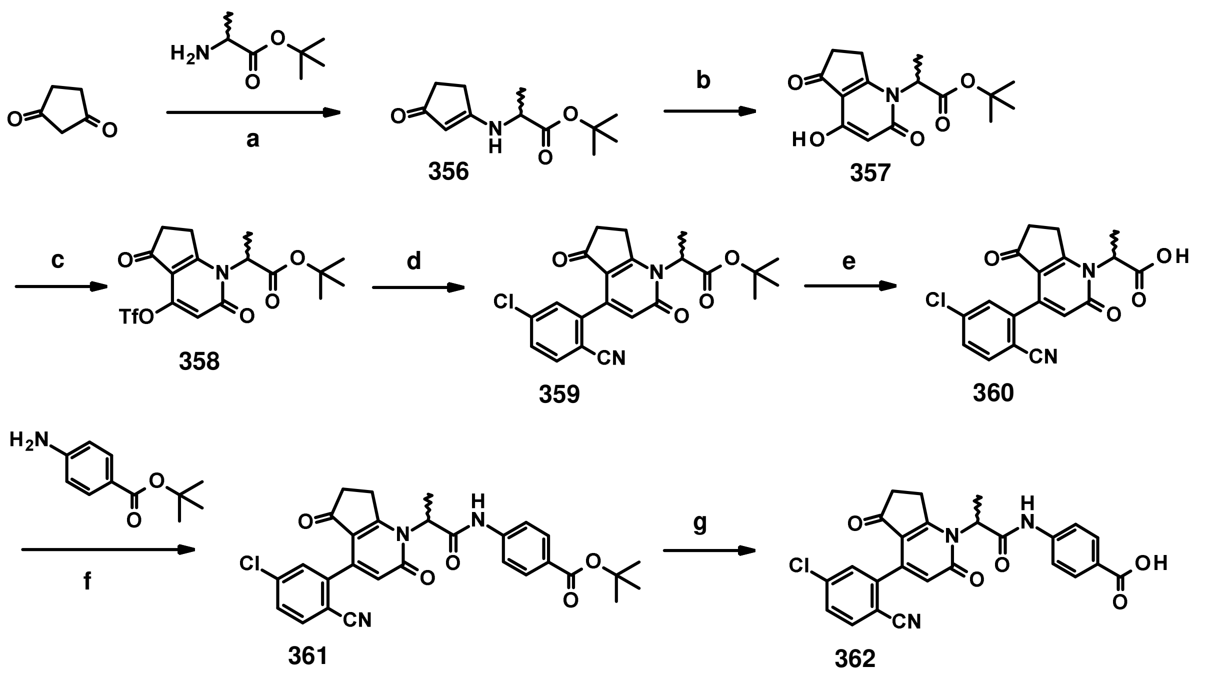

[a]Reagents and conditions: (a) p-TsOH × $H_2O$, toluene, RF, 89%; (b) bis(2,4,6-trichlorophenyl) malonate, diethyleneglycol dimethylether, 100 °C, 14%; (c) 1,1,1-trifluoro-*N*-phenyl-*N*-[(trifluoromethyl)sulfonyl]methanesulfonamide, $NEt_3$, DCM, 0 °C → RT, 19%; (d) (5-chloro-2-cyanophenyl)boronic acid, $Pd(Ph_3P)_4$, $K_2CO_3$, 1,4-dioxane, 110 °C, 45%; (e) TFA, DCM, RT, 100%; (f) HATU, DIEA, DMF, RT, 60%; (g) TFA, DCM, RT, 59%.

***tert*-Butyl *N*-(3-oxocyclopent-1-en-1-yl)alaninate (racemate 356).**

A solution of cyclopentane-1,3-dione (1.70 g, 17.36 mmol, 1.05 eq.), racemic *tert*-butyl alaninate (2.40 g, 16.53 mmol) and 4-toluenesulfonic acid monohydrate (314 mg, 1.65 mmol, 0.1 eq.) in toluene (80 mL) was stirred under reflux for 7 h (Dean-Stark water separator) and cooled to RT. Toluene was removed *in vacuo*. The residue was mixed with dichloromethane and washed with water and brine. The combined aqueous phases were extracted with dichloromethane. The combined organic phases were dried over anhydrous sodium sulfate, filtered and concentrated under reduced pressure to give **356** which was used without further purification. Yield: 3.48 g (89% of theory). LC/MS (method 1): $t_R$ = 0.64 min, MS (ESIpos): $m/z$ = 226 $[M+H]^+$.

***tert*-Butyl 2-(4-hydroxy-2,5-dioxo-2,5,6,7-tetrahydro-1*H*-cyclopenta[*b*]pyridin-1-yl)propanoate (racemate 357).**

A solution of *tert*-butyl *N*-(3-oxocyclopent-1-en-1-yl)alaninate (racemate **356**) (2.00 g, 8.88 mmol) and bis(2,4,6-trichlorophenyl) malonate (4.52 g, 9.76 mmol, 1.1 eq.) in diethyleneglycol dimethylether (25 mL) was stirred at 100 °C for 5 h, mixed with additional bis(2,4,6-trichlorophenyl) malonate (2.26 g, 4.88 mmol, 0.55 eq.) and stirred at 100 °C for another 8 h. The reaction mixture was cooled to RT and

evaporated under reduced pressure. The residue was coevaporated three times with water, dried *in vacuo* and purified by flash silica gel chromatography (cyclohexane / ethyl acetate gradient) to give **357**. Yield: 229 mg (90% purity, 8% of theory) and 573 mg (65% purity, 14% of theory). LC/MS (method 1): $t_R$ = 0.69 min, MS (ESIpos): *m/z* = 294 $[M+H]^+$.

***tert*-Butyl 2-(2,5-dioxo-4-{[(trifluoromethyl)sulfonyl]oxy}-2,5,6,7-tetrahydro-1*H*-cyclopenta[*b*]pyridin-1-yl)propanoate (racemate 358).**

Triethylamine (115 µL, 0.83 mmol, 1.2 eq.) was added at 0 °C to a solution of *tert*-butyl 2-(4-hydroxy-2,5-dioxo-2,5,6,7-tetrahydro-1*H*-cyclopenta[*b*]pyridin-1-yl)propanoate (racemate **357**) (220 mg, 90% purity, 0.68 mmol) in dichloromethane (6 mL), followed by addition of 1,1,1-trifluoro-*N*-phenyl-*N*-[(trifluoromethyl)sulfonyl]methanesulfonamide (295 g, 0.83 mmol, 1.2 eq.) in portions. The reaction mixture was stirred at RT overnight, diluted with dichloromethane, washed with aqueous hydrochloric acid solution (1 N) and evaporated under reduced under reduced pressure. The residue was purified by RP-HPLC (acetonitrile / 0.05% formic acid in water gradient) to give **358**. Yield: 59 mg (91% purity, 19% of theory). LC/MS (method 1): $t_R$ = 1.04 min, MS (ESIpos): *m/z* = 426 $[M+H]^+$; $^1$H NMR (400 MHz, DMSO-$d_6$): δ [ppm] = 6.60 (s, 1H), 5.09 (q, *J* = 6.8 Hz, 2H), 3.27-3.09 (m, 2H), 2.80-2.59 (m, 2H), 1.53 (d, *J* = 6.9 Hz, 3H), 1.36 (s, 9H).

***tert*-Butyl 2-[4-(5-chloro-2-cyanophenyl)-2,5-dioxo-2,5,6,7-tetrahydro-1*H*-cyclopenta[*b*]pyridin-1-yl]propanoate (racemate 359).**

A mixture of *tert*-butyl 2-(2,5-dioxo-4-{[(trifluoromethyl)sulfonyl]oxy}-2,5,6,7-tetrahydro-1*H*-cyclopenta[*b*]pyridin-1-yl)propanoate (racemate **358**) (89 mg, 0.21 mmol), (5-chloro-2-cyanophenyl)boronic acid (44 mg, 0.24 mmol, 1.15 eq.) and potassium carbonate (87 mg, 0.63 mmol, 3.0 eq.) in 1,4-dioxane (3 mL) was flushed with argon at RT, followed by addition of tetrakis(triphenylphosphine)palladium (24 mg, 0.02 mmol, 0.1 eq.). The reaction mixture was stirred at 110 °C overnight, cooled to RT, filtered and concentrated under reduced pressure. The residue was purified by RP-HPLC (acetonitrile / 0.05% formic acid in water gradient) to give **359**. Yield: 39 mg (45% of theory). LC/MS (method 1): $t_R$ = 1.04 min, MS (ESIneg): *m/z* = 413 $[M-H]^-$; $^1$H NMR (400 MHz, DMSO-$d_6$): δ [ppm] = 7.97 (d, *J* = 8.3 Hz, 1H), 7.73 (dd, *J* = 8.3 Hz, 2.1 Hz, 1H), 7.66 (d, *J* = 2.1 Hz, 1H), 6.44 (s, 1H), 5.10 (q, *J* = 6.8 Hz, 1H), 3.3-3.09 (m, 2H, partially concealed), 2.71-2.5 (m, 2H, partially concealed), 1.58 (d, *J* = 6.8 Hz, 3H), 1.38 (s, 9H).

**2-[4-(5-Chloro-2-cyanophenyl)-2,5-dioxo-2,5,6,7-tetrahydro-1*H*-cyclopenta[*b*]pyridin-1-yl]propanoic acid (racemate 360).**

Trifluoroacetic acid (0.21 mL, 2.71 mmol, 20 eq.) was added at RT to a solution *tert*-butyl 2-[4-(5-chloro-2-cyanophenyl)-2,5-dioxo-2,5,6,7-tetrahydro-1*H*-cyclopenta[*b*]pyridin-1-yl]propanoate (racemate **359**) (56 mg, 0.14 mmol) in dichloromethane (2.5 mL). The reaction mixture was stirred at RT overnight and concentrated under reduced pressure. The residue was coevaporated three times with

dichloromethane and dried *in vacuo* to give **360**. Yield: 62 mg (100% of theory). LC/MS (method 1): $t_R$ = 0.75 min, MS (ESIpos): *m/z* = 357 $[M+H]^+$; $^1H$ NMR (400 MHz, DMSO-$d_6$): δ [ppm] = 12.9 (br s, 1H), 7.96 (d, *J* = 8.3 Hz, 1H), 7.73 (dd, *J* = 8.3 Hz, 2.1 Hz, 1H), 7.67 (d, *J* = 2.0 Hz, 1H), 6.43 (s, 1H), 5.11 (q, *J* = 6.8 Hz, 1H), 3.29-3.11 (m, 2H), 2.70-2.5 (m, 2H, partially concealed), 1.59 (d, *J* = 6.8 Hz, 3H).

***tert*-Butyl 4-{2-[4-(5-chloro-2-cyanophenyl)-2,5-dioxo-2,5,6,7-tetrahydro-1*H*-cyclopenta[*b*]pyridin-1-yl]propanamido}benzoate (racemate 361).**

A solution of HATU (59 mg, 0.16 mmol, 1.2 eq.) in *N,N*-dimethylformamide (0.5 mL) was added under argon atmosphere at RT to a solution of 2-[4-(5-chloro-2-cyanophenyl)-2,5-dioxo-2,5,6,7-tetrahydro-1*H*-cyclopenta[*b*]pyridin-1-yl]propanoic acid (racemate **360**) (46 mg, 0.13 mmol), *tert*-butyl 4-aminobenzoate (30 mg, 0.16 mmol, 1.2 eq.) and *N,N*-diisopropylethylamine (68 µL, 0.39 mmol, 3.0 eq.) in *N,N*-dimethylformamide (4 mL). The reaction mixture was stirred at RT overnight, mixed with additional HATU (30 mg, 0.08 mmol, 0.6 eq.) and *N,N*-diisopropylethylamine (34 µL, 0.20 mmol, 1.5 eq.), stirred at RT for another 24 h and concentrated under reduced pressure. The residue was purified by preparative RP-HPLC (acetonitrile / 0.05% formic acid in water gradient) to give **361**. Yield: 42 mg (60% of theory). LC/MS (method 1): $t_R$ = 1.13 min, MS (ESIneg): *m/z* = 532 $[M-H]^-$; $^1H$ NMR (400 MHz, DMSO-$d_6$): δ [ppm] = 9.97 (br s, 1H), 7.98 (d, *J* = 8.4 Hz, 1H), 7.86 (d, *J* = 8.8 Hz, 2H), 7.77-7.67 (m, 3H), 7.63 (d, *J* = 2.0 Hz, 1H), 6.43 (s, 1H), 5.31-5.22 (m, 1H), 3.24-3.12 (m, 2H), 2.69-2.61 (m, 2H), 1.66 (d, *J* = 6.9 Hz, 3H), 1.54 (s, 9H).

**4-{2-[4-(5-Chloro-2-cyanophenyl)-2,5-dioxo-2,5,6,7-tetrahydro-1*H*-cyclopenta[*b*]pyridin-1-yl]propanamido}benzoic acid (racemate 362).**

Trifluoroacetic acid (116 µL, 1.50 mmol, 20 eq.) was added at RT to a suspension of *tert*-butyl 4-{2-[4-(5-chloro-2-cyanophenyl)-2,5-dioxo-2,5,6,7-tetrahydro-1*H*-cyclopenta[*b*]pyridin-1-yl]propanamido}-benzoate (racemate **361**) (40 mg, 0.08 mmol) in dichloromethane (3 mL). The reaction mixture was stirred at RT for 3 h and concentrated under reduced pressure. The residue was purified by preparative RP-HPLC (acetonitrile / water gradient) to give **362**. Yield: 21 mg (59% of theory). LC/MS (method 1): $t_R$ = 0.86 min, MS (ESIpos): *m/z* = 476 $[M+H]^+$; $^1H$ NMR (400 MHz, DMSO-$d_6$): δ [ppm] = 12.73 (br s, 1H), 9.96 (br s, 1H), 7.98 (d, *J* = 8.4 Hz, 1H), 7.90 (d, *J* = 8.8 Hz, 2H), 7.76-7.67 (m, 3H), 7.63 (d, *J* = 2.0 Hz, 1H), 6.42 (s, 1H), 5.32-5.22 (m, 1H), 3.24-3.13 (m, 2H), 2.70-2.61 (m, 2H), 1.66 (d, *J* = 6.9 Hz, 3H).

**(D) LC/MS data of compounds 1–85, 355, and 362.**

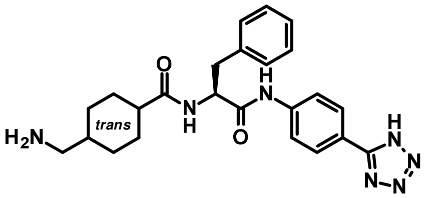


**1**

MS-MW [g/mol]: 447.2383

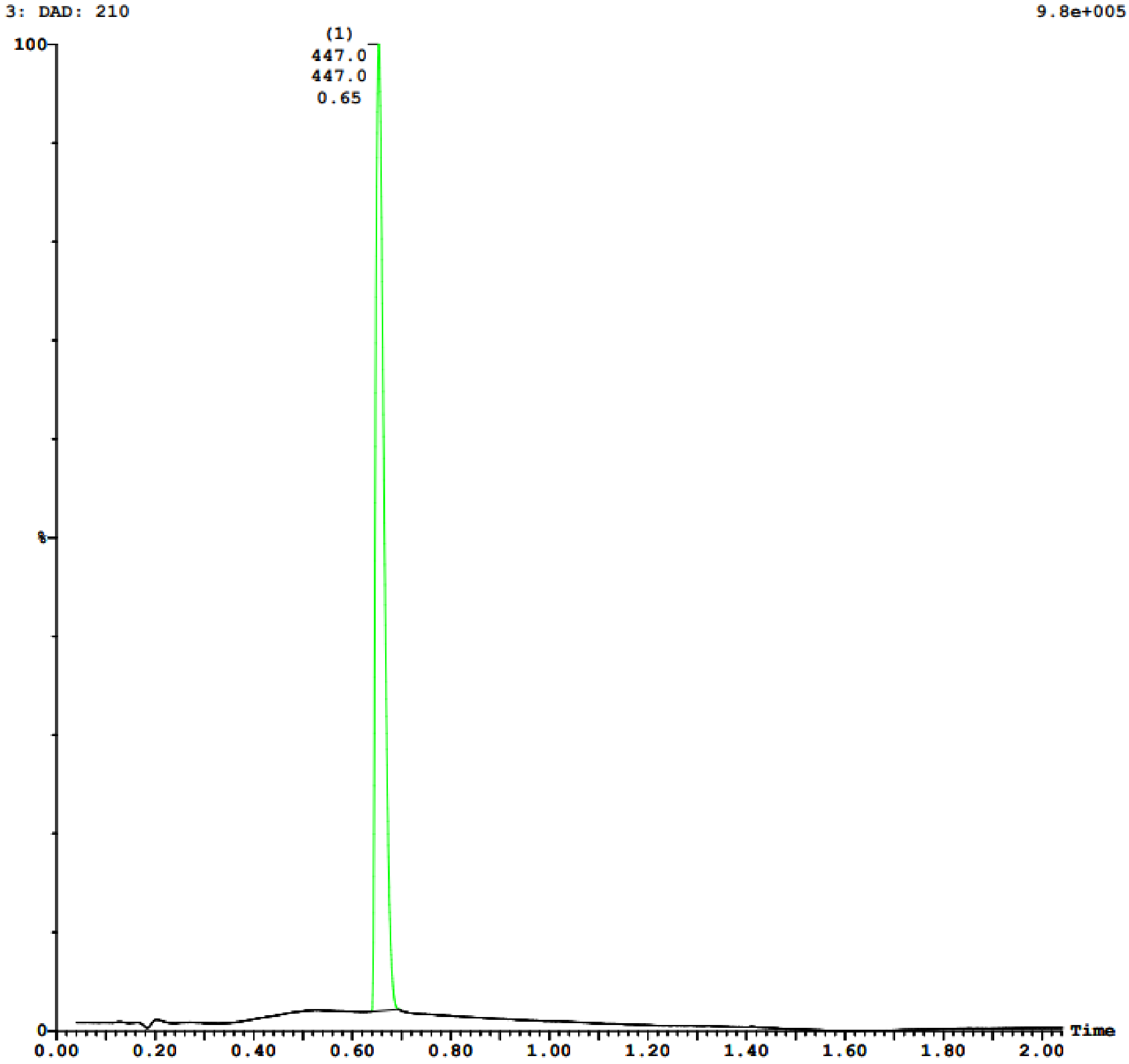


| Peak Number | Time | AreaAbs | Area %Total | Mass Found |
|---|---|---|---|---|
| 1 | 0.65 | 18979 | 100.00 | 447.00 |

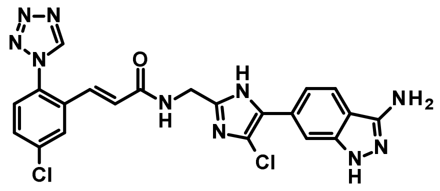

**2**

MS-MW [g/mol]: 494.0886

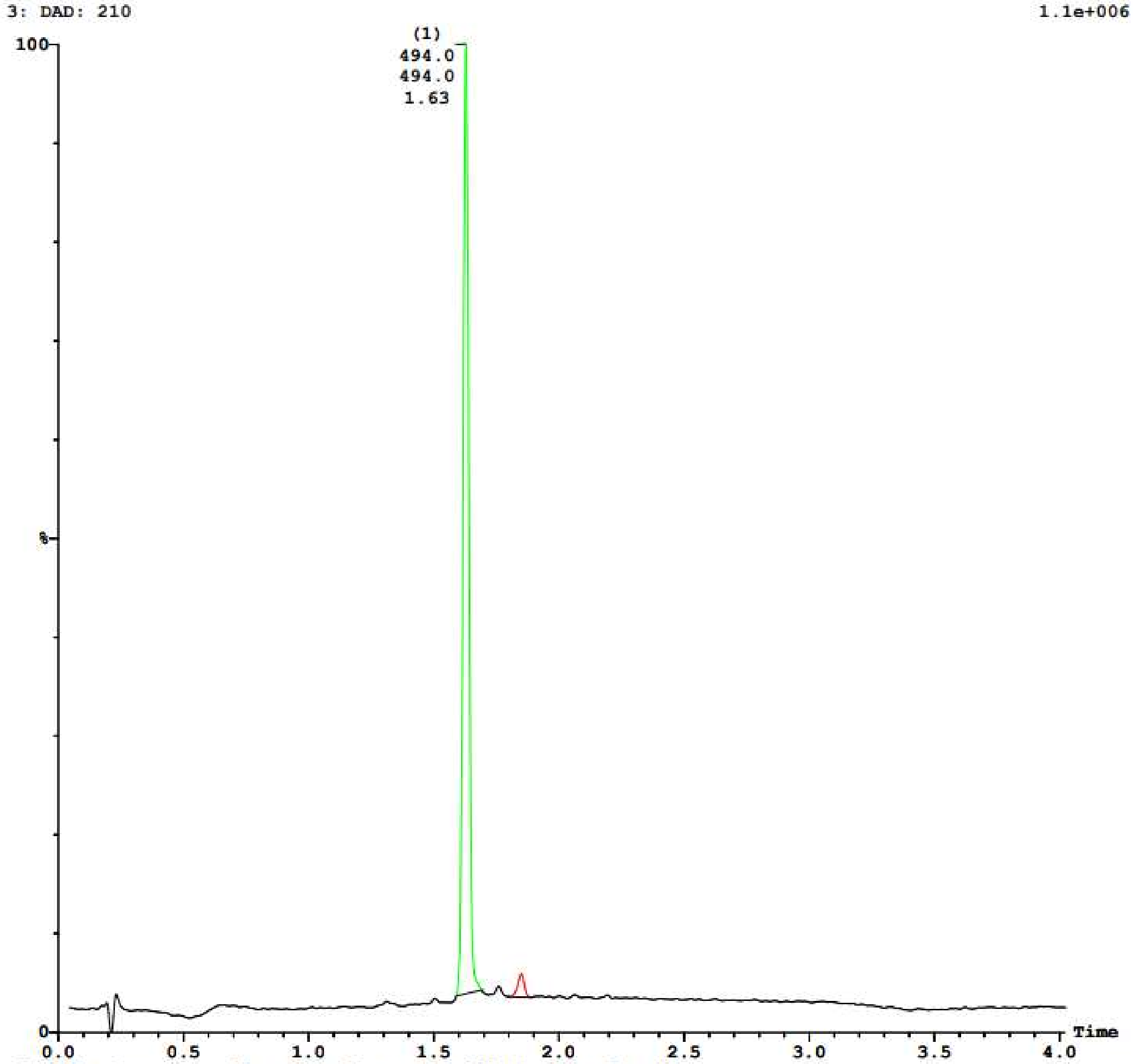


| Peak Number | Time | AreaAbs | Area %Total | Mass Found |
|---|---|---|---|---|
| 1 | 1.63 | 27736 | 97.3 | 494.0 |
| 2 | 1.85 | 772 | 2.7 | |

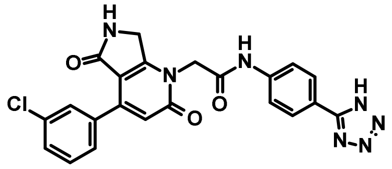

**3**

MS-MW [g/mol]: 461.1003

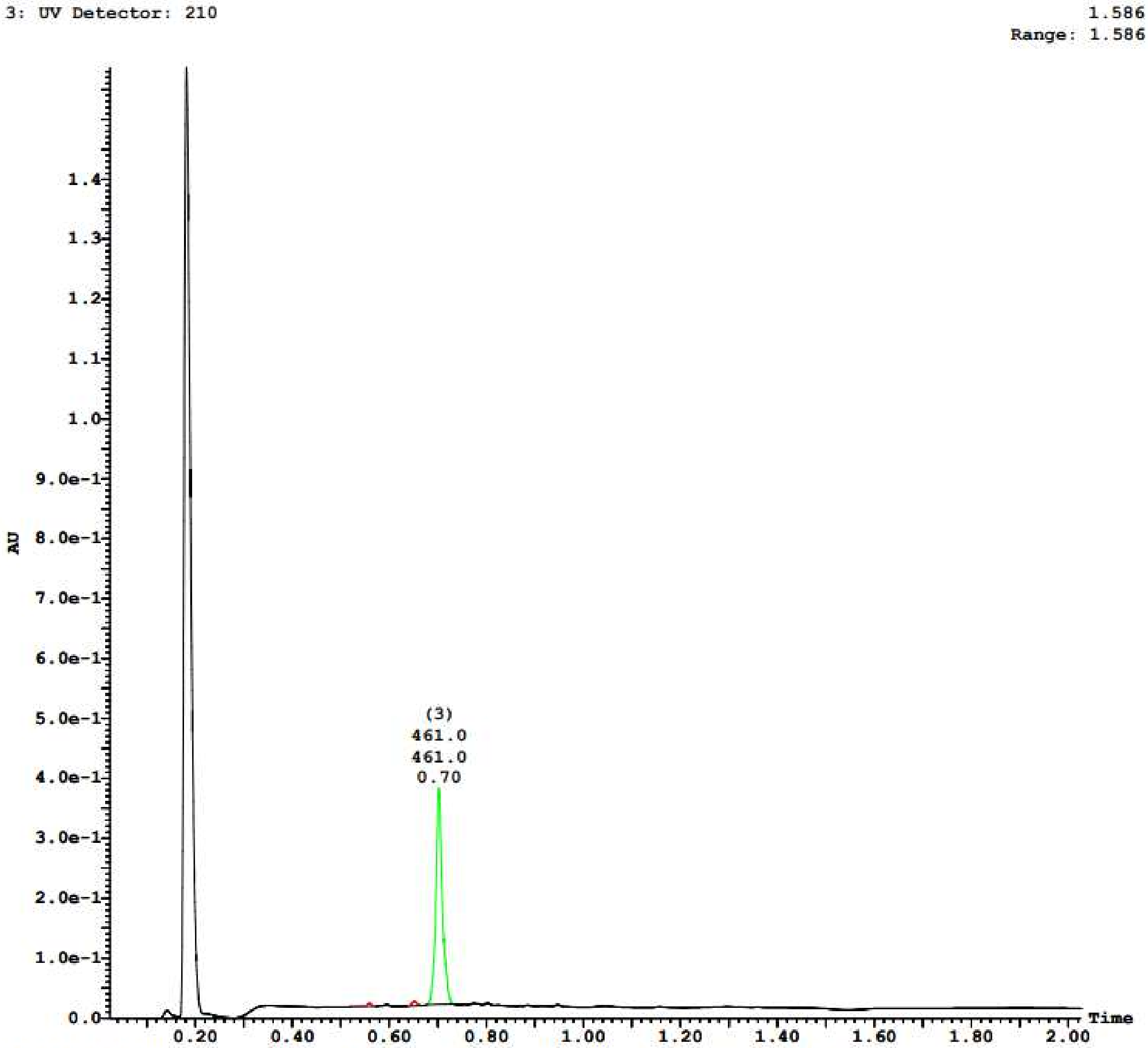


| Peak Number | Compound | Time | AreaAbs | Area %Total | Width | Height | Mass Found |
|---|---|---|---|---|---|---|---|
| 1 | | 0.56 | 64 | 1.11 | 0 | 4947 | |
| 2 | | 0.65 | 94 | 1.64 | 0 | 7757 | |
| 3 | Found | 0.70 | 5576 | 97.25 | 0 | 360683 | 461.00, 461.00 |

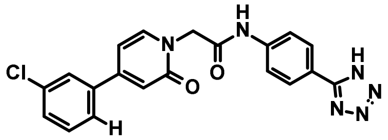

**4**

MS-MW [g/mol]: 406.0945

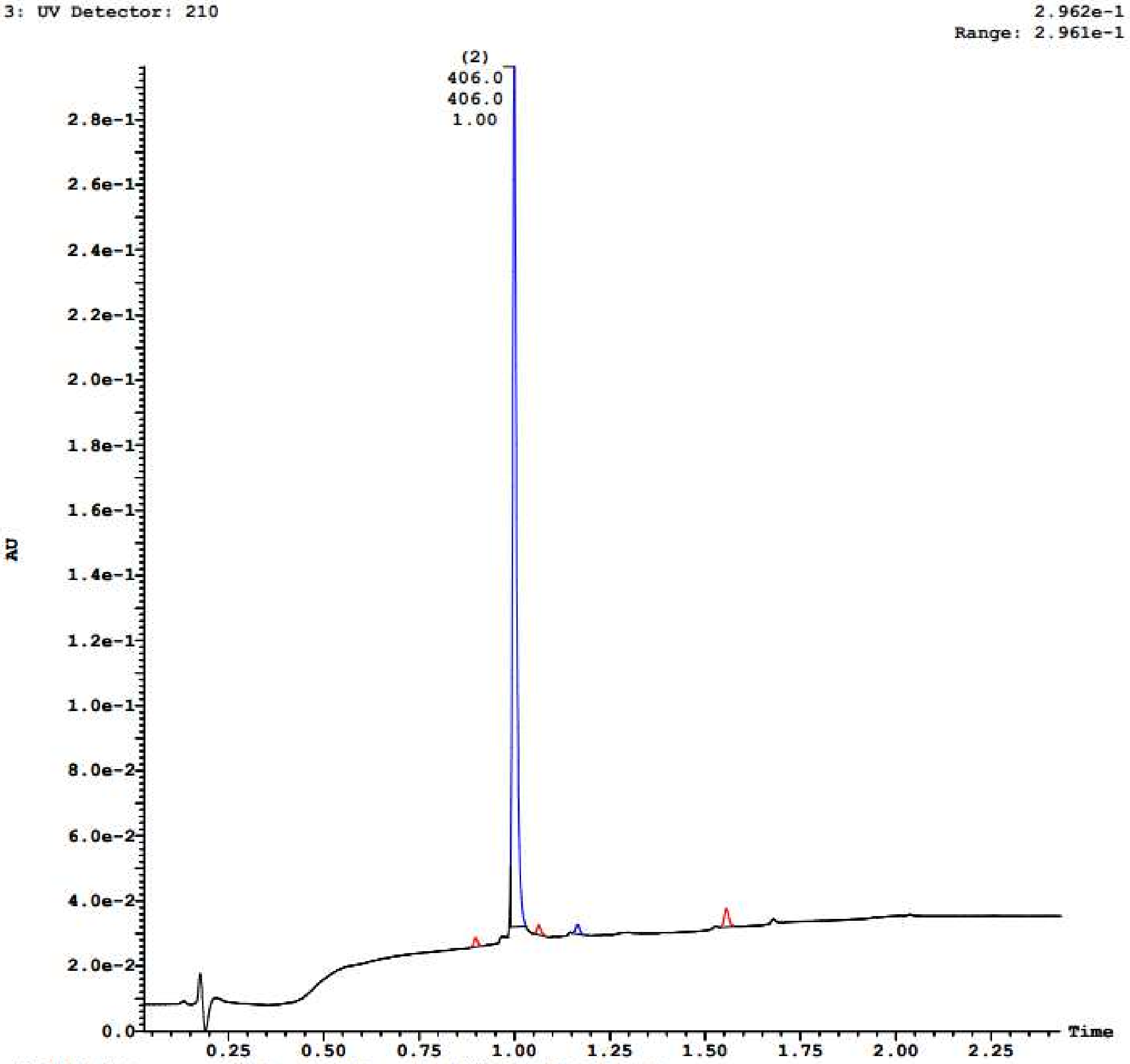


| Peak Number | Time | AreaAbs | Area %Total | Mass Found |
|---|---|---|---|---|
| 1 | 0.90 | 33 | 0.97 | |
| 2 | 1.00 | 3214 | 94.52 | 406.00, 406.00 |
| 3 | 1.06 | 38 | 1.12 | |
| 4 | 1.16 | 37 | 1.08 | 406.00 |
| 5 | 1.56 | 78 | 2.31 | |

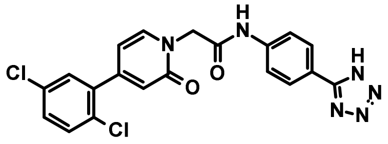

**5**

MS-MW [g/mol]: 440.0555

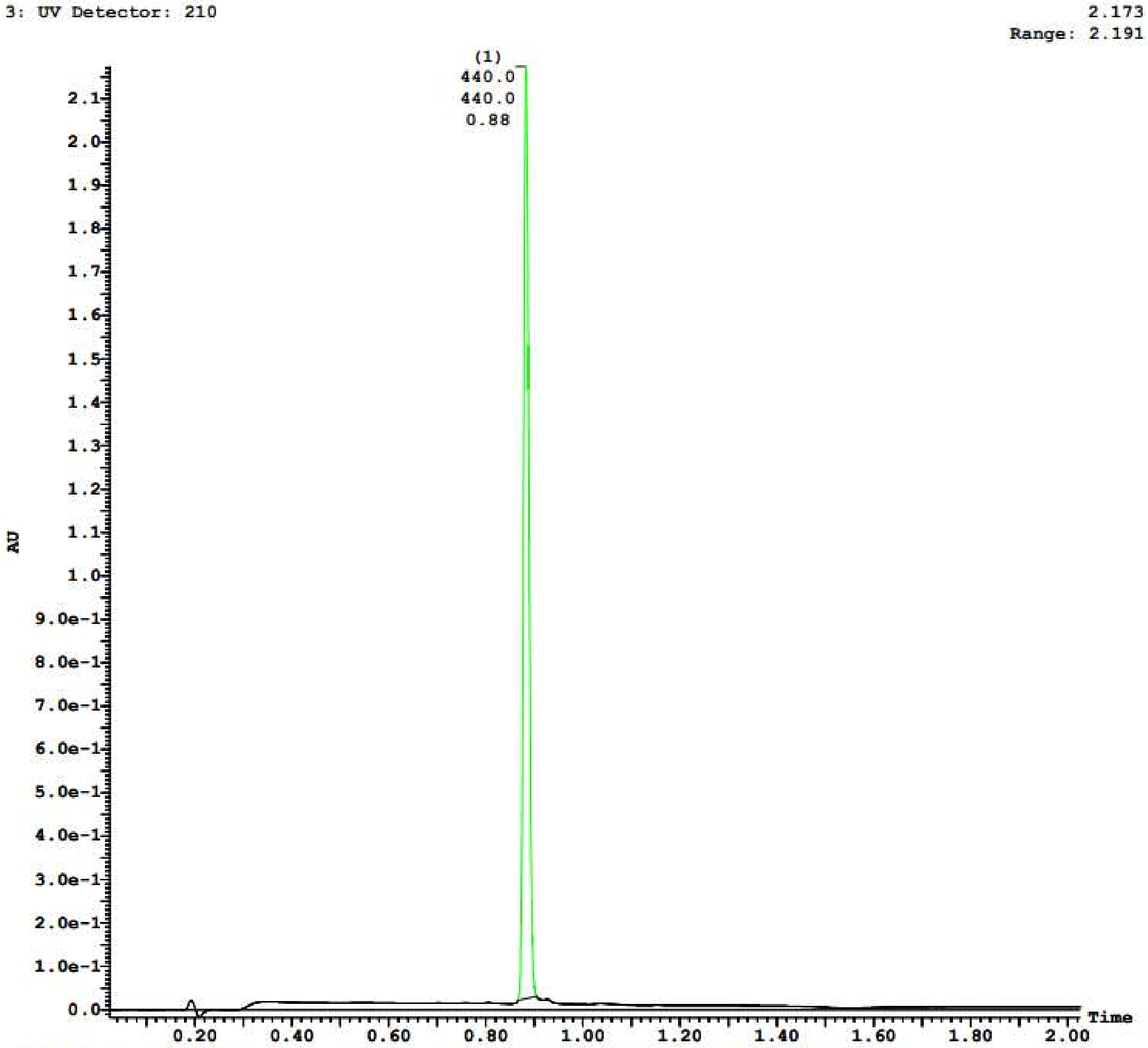


| Peak Number | Compound | Time | AreaAbs | Area %Total | Width | Height | Mass Found |
|---|---|---|---|---|---|---|---|
| 1 | Found | 0.88 | 27562 | 100.00 | 0 | 2147587 | 440.00, 440.00 |

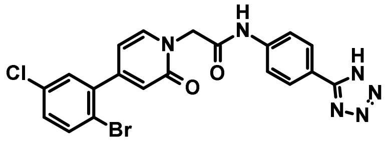


**6**

MS-MW [g/mol]: 484.0050

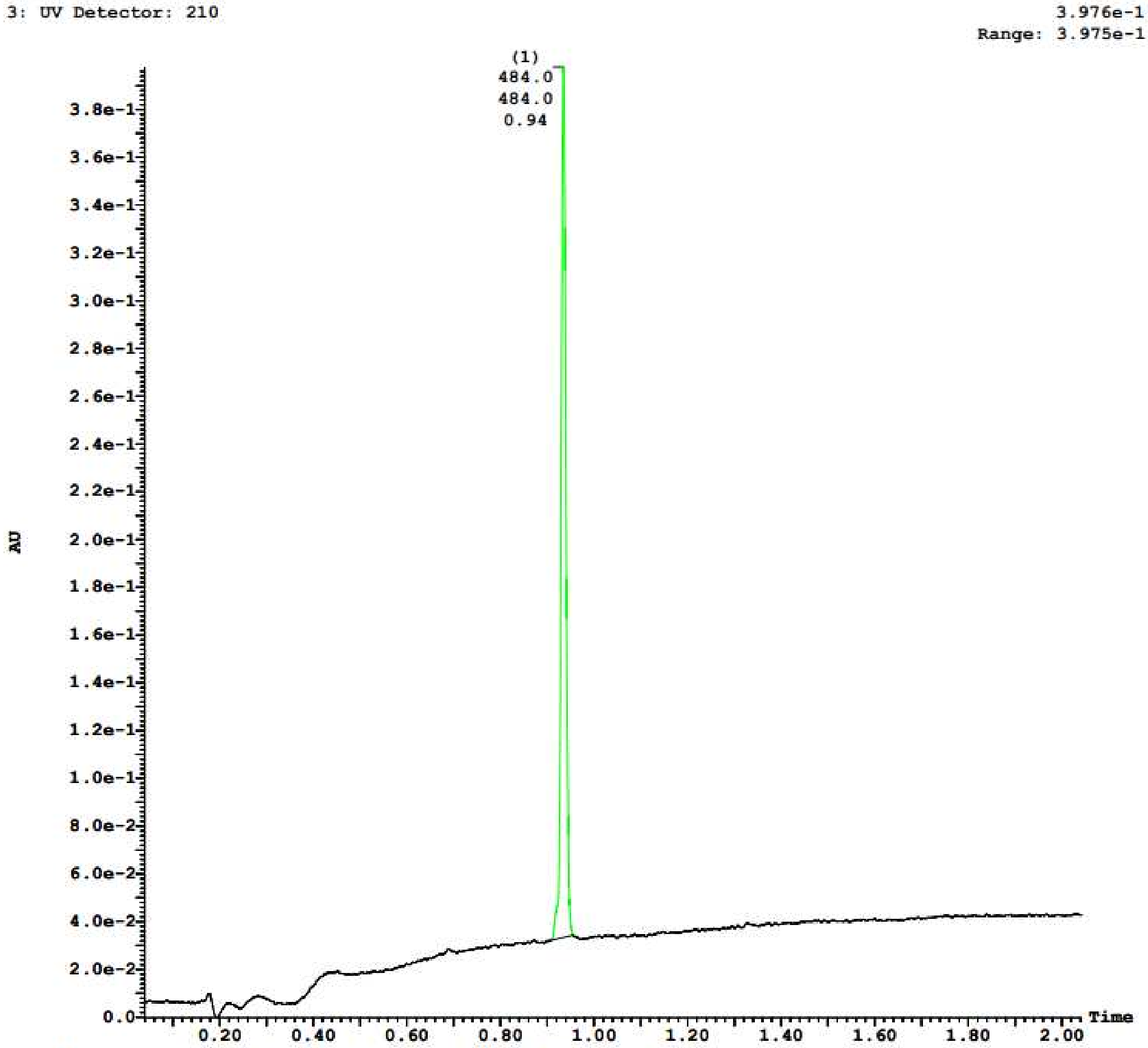


| Peak Number | Time | AreaAbs | Area %Total | Mass Found |
|---|---|---|---|---|
| 1 | 0.94 | 4294 | 100.00 | 484.00, 484.00 |

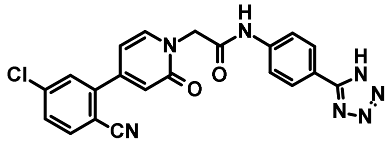

**7**

MS-MW [g/mol]: 431.0898

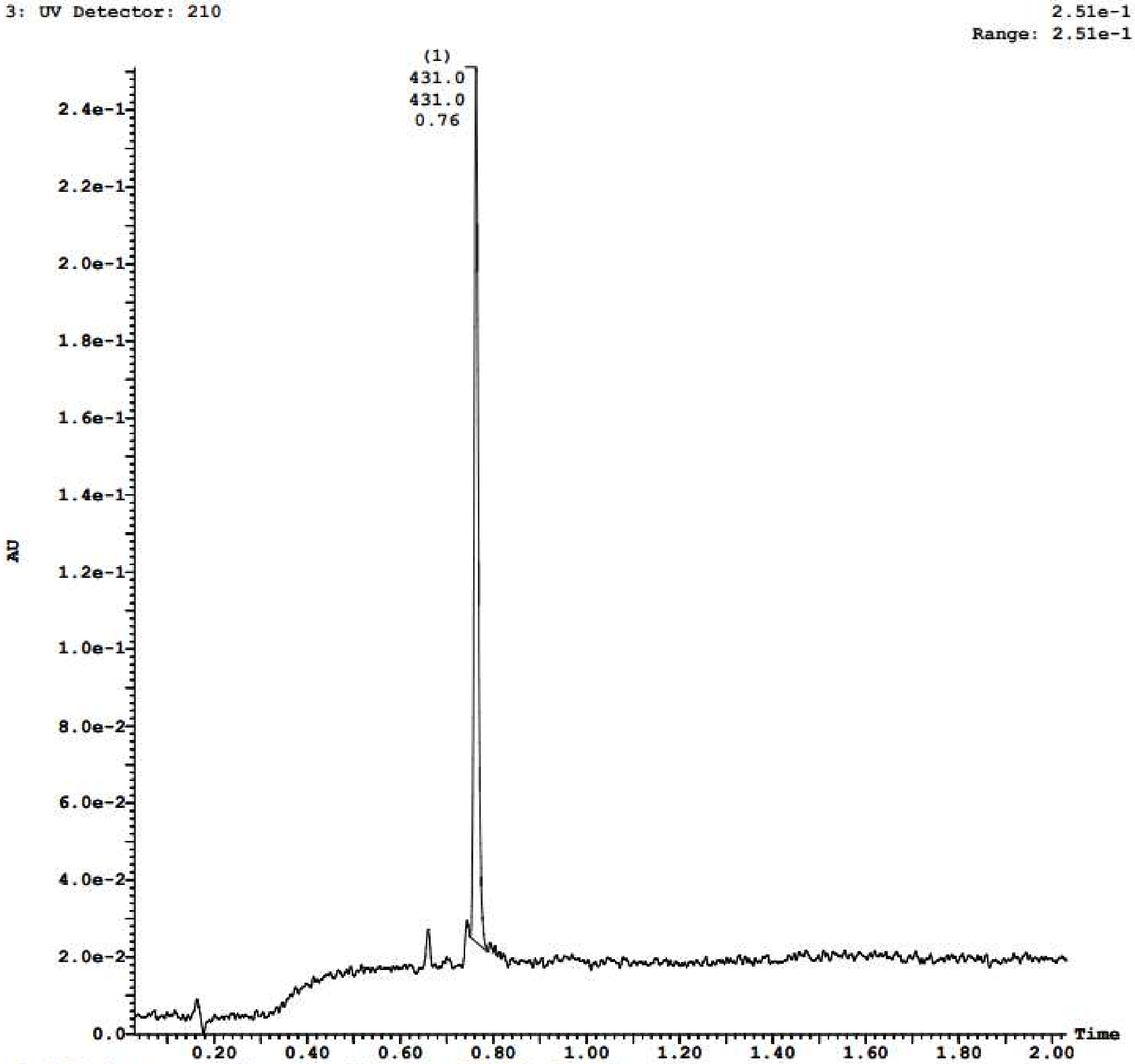


| Peak Number | Time | AreaAbs | Area %Total | Mass Found |
|---|---|---|---|---|
| 1 | 0.76 | 2352 | 100.00 | 431.00, 431.00 |

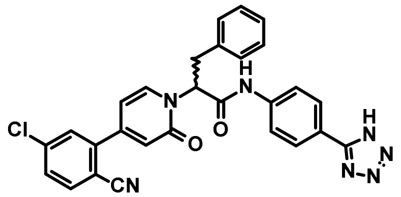

**8**

521.1367

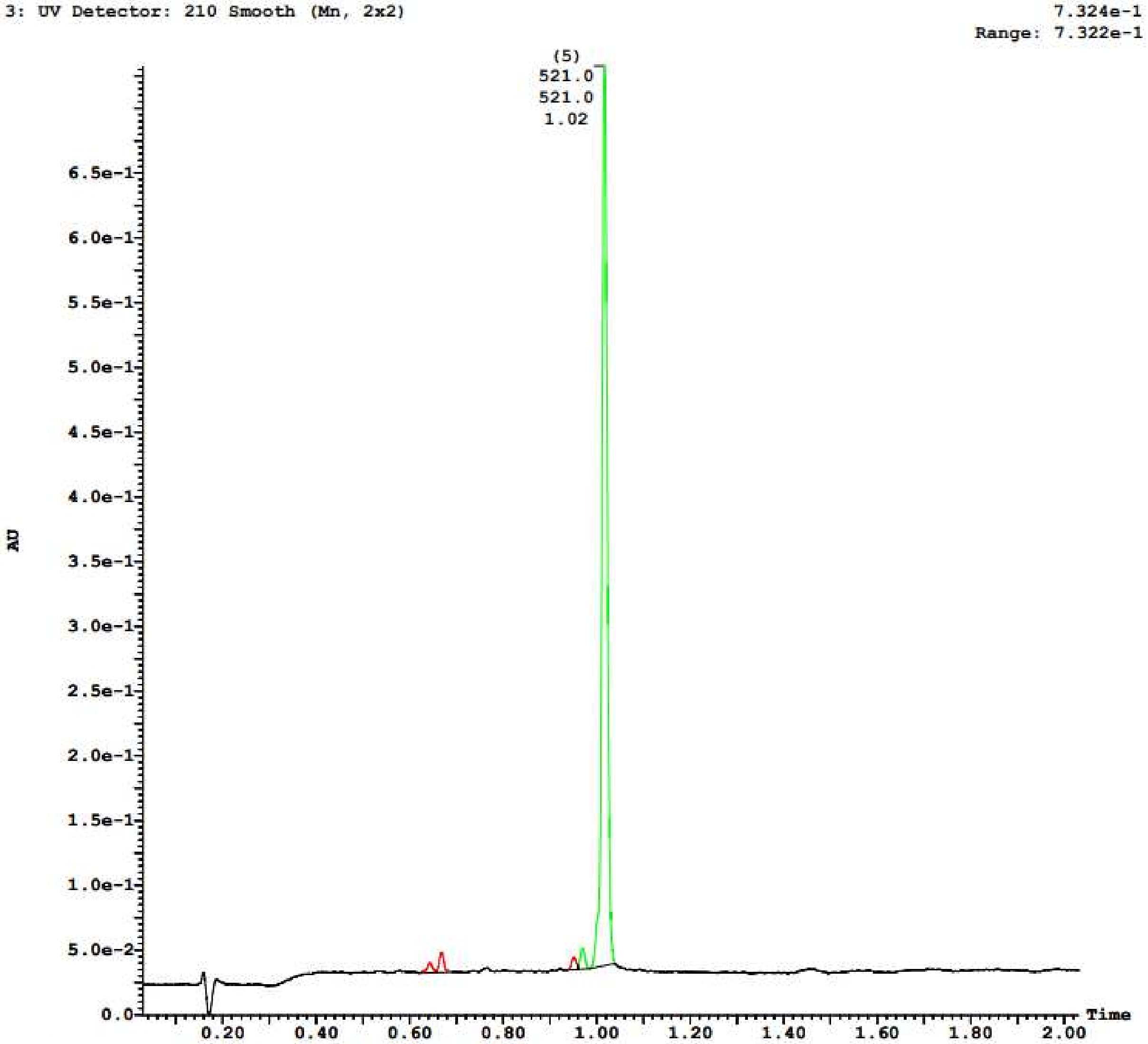


| Peak Number | Time | AreaAbs | Area %Total | Mass Found |
|---|---|---|---|---|
| 1 | 0.64 | 107 | 1.09 | |
| 2 | 0.67 | 177 | 1.81 | |
| 3 | 0.95 | 113 | 1.15 | |
| 4 | 0.97 | 191 | 1.95 | 521.00, 521.00 |
| 5 | 1.02 | 9221 | 94.00 | 521.00, 521.00 |

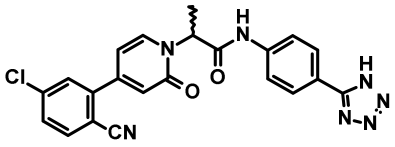


**9**

MS-MW [g/mol]: 445.1054

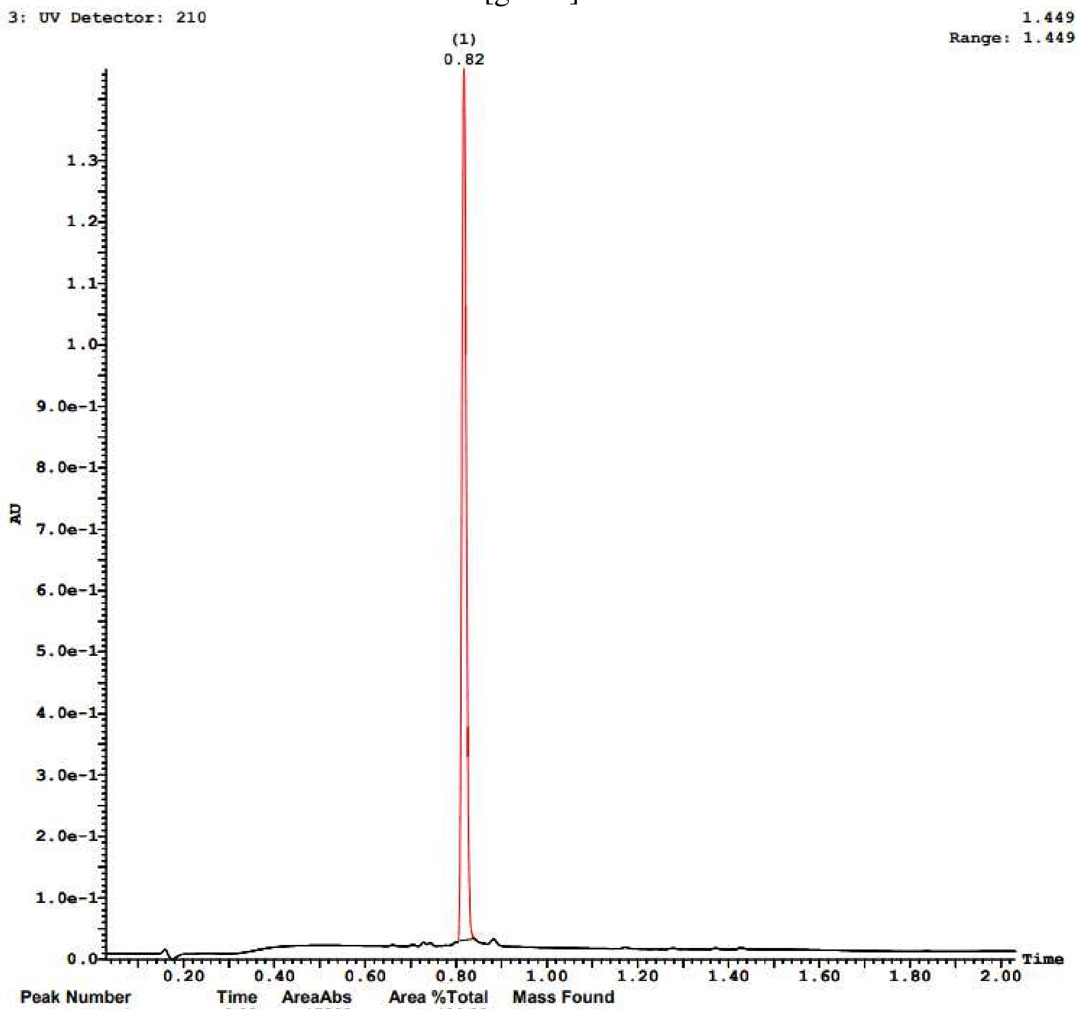


| Peak Number | Time | AreaAbs | Area %Total | Mass Found |
|---|---|---|---|---|
| 1 | 0.82 | 15666 | 100.00 | |

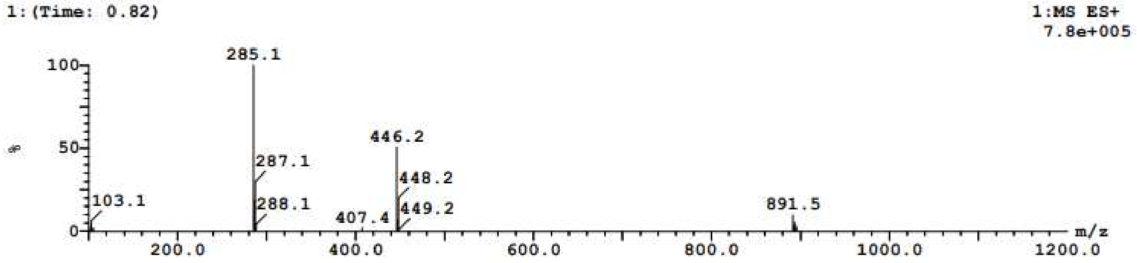


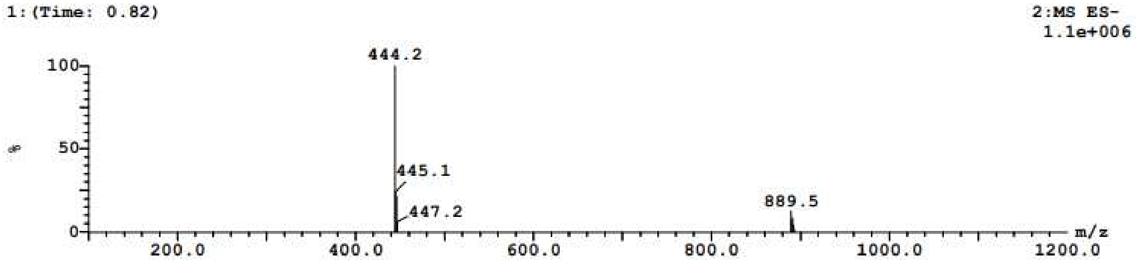

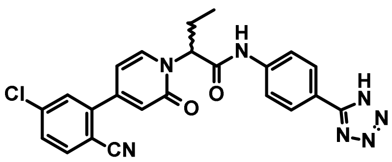

**10**

MS-MW [g/mol]: 459.1211

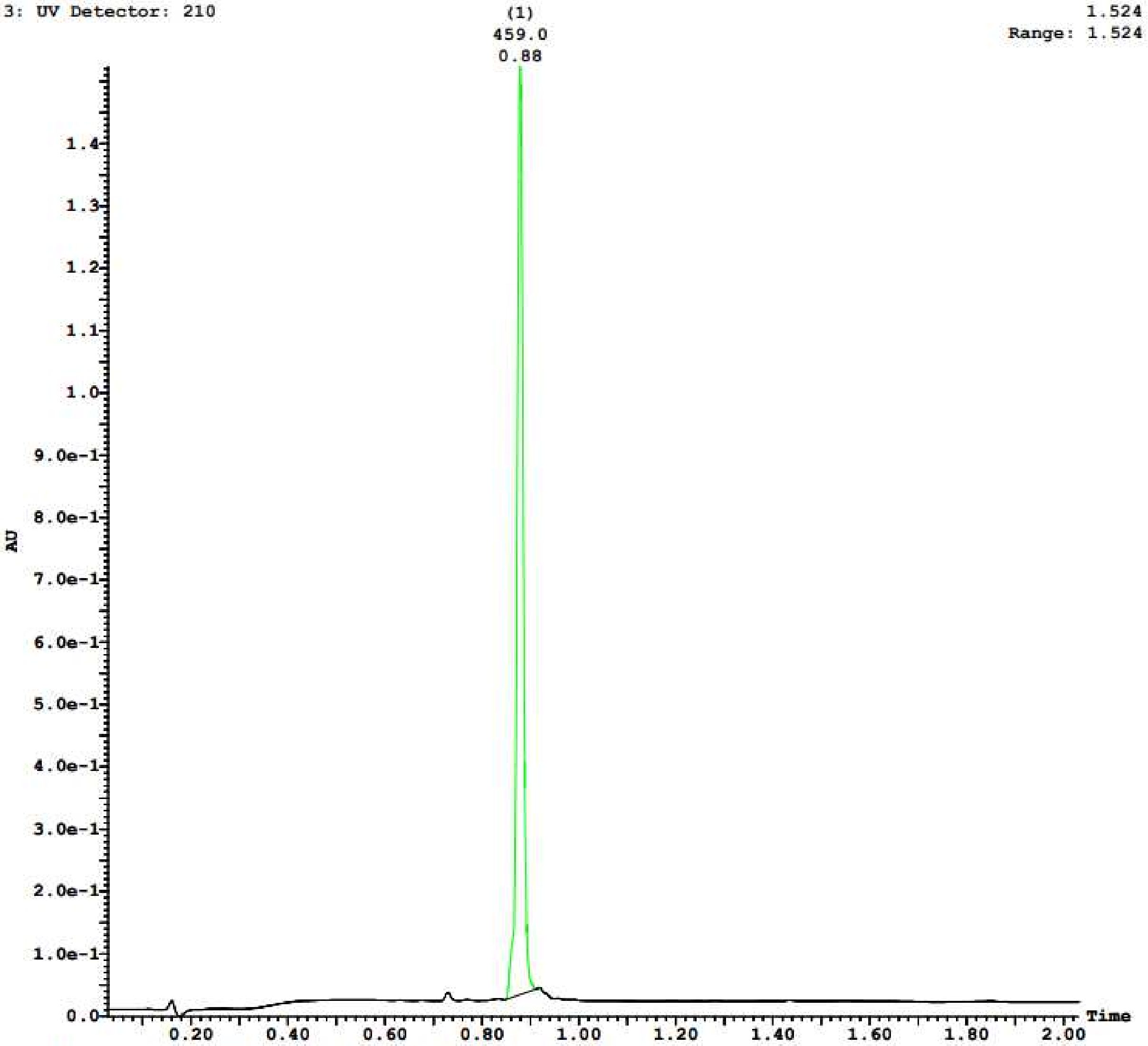


| Peak Number | Time | AreaAbs | Area %Total | Mass Found |
|---|---|---|---|---|
| 1 | 0.88 | 21771 | 100.00 | 459.00 |

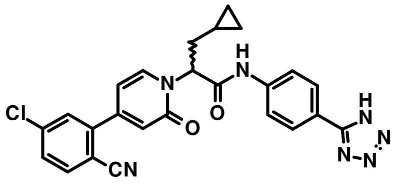


**11**

MS-MW [g/mol]: 485.1367

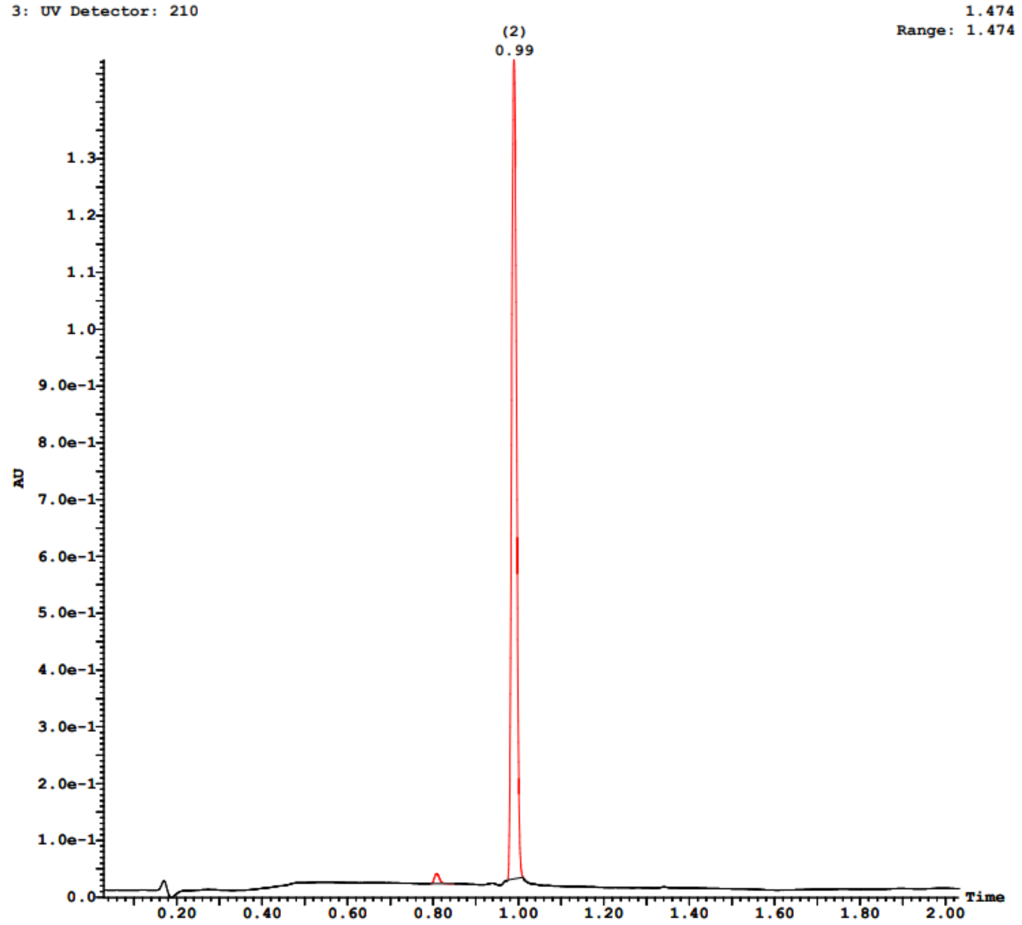


| Peak Number | Time | AreaAbs | Area %Total | Mass Found |
|---|---|---|---|---|
| 1 | 0.81 | 260 | 1.34 | |
| 2 | 0.99 | 19149 | 98.66 | |

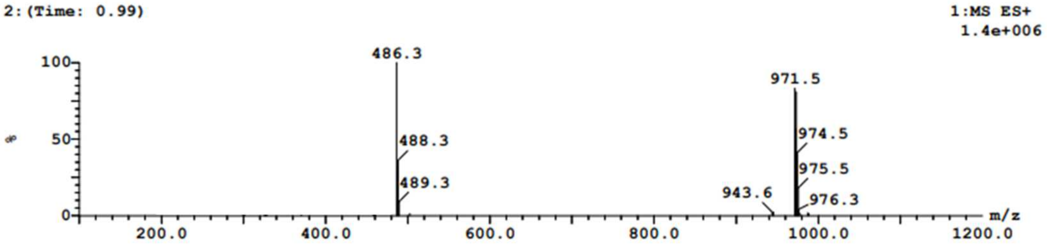


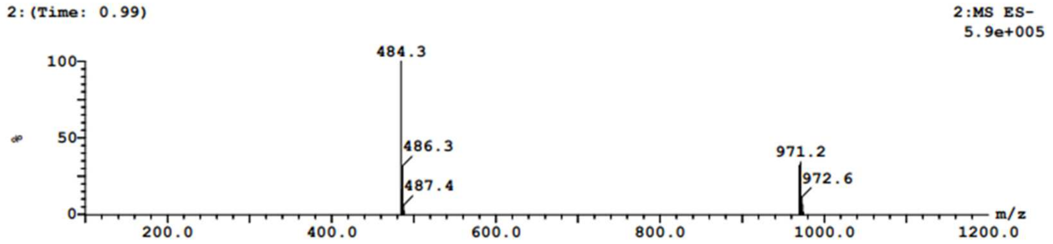

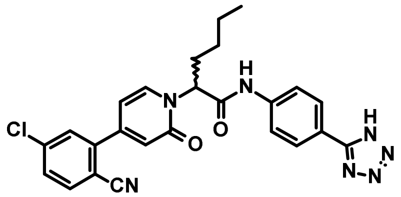


**12**

MS-MW [g/mol]: 487.1524

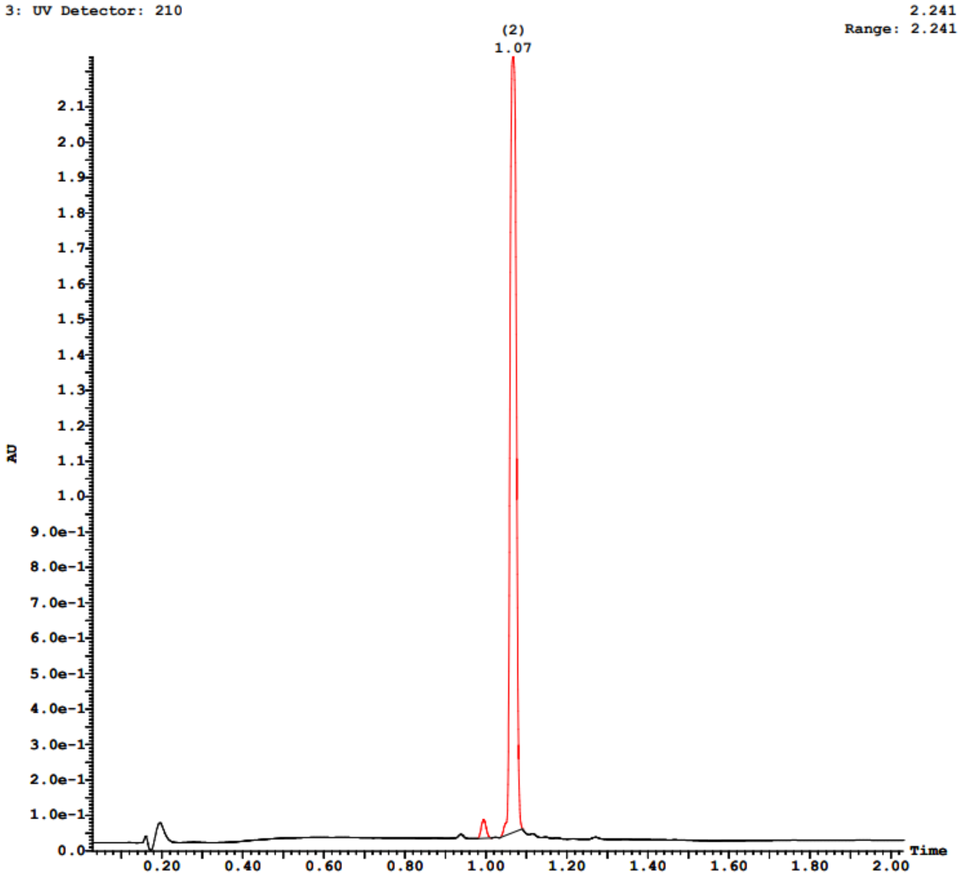


| Peak Number | Time | AreaAbs | Area %Total | Mass Found |
|---|---|---|---|---|
| 1 | 0.99 | 746 | 1.89 | |
| 2 | 1.07 | 38821 | 98.11 | |

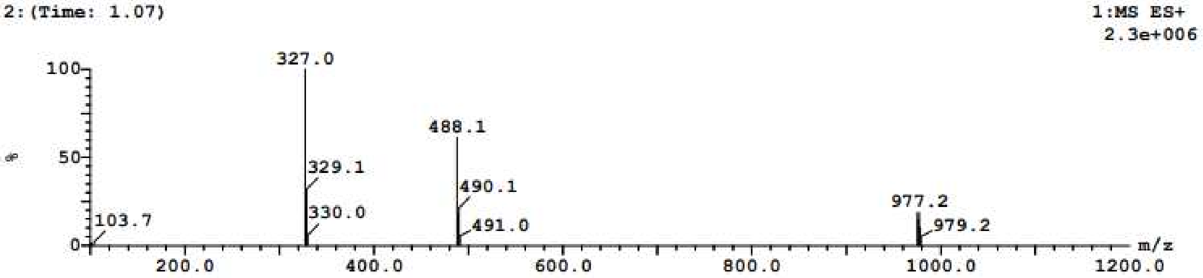


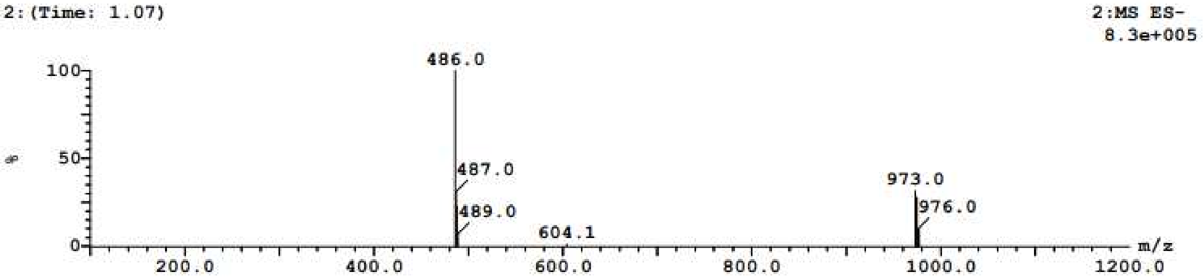

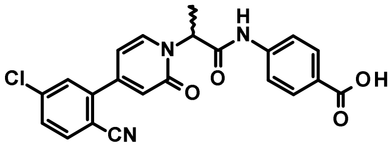

**13**

MS-MW [g/mol]: 421.0829

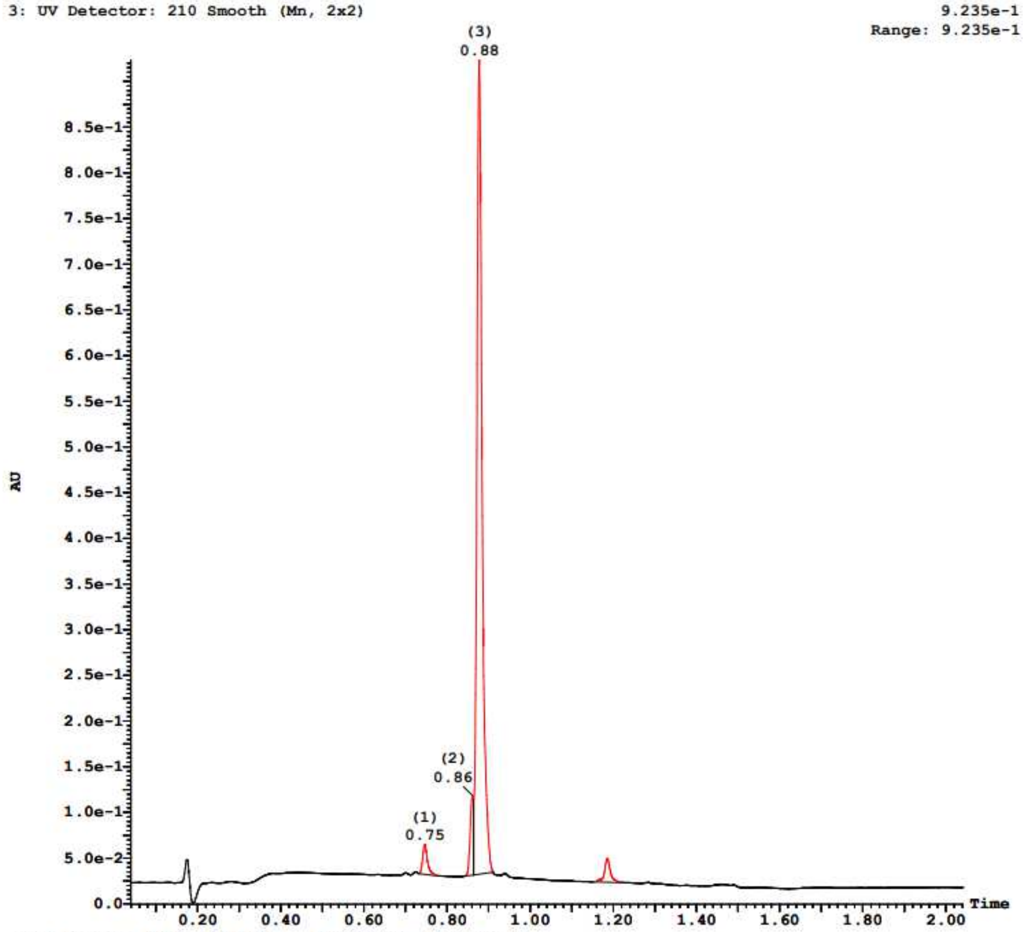


| Peak Number | Time | AreaAbs | Area %Total | Mass Found |
|---|---|---|---|---|
| 1 | 0.75 | 437 | 2.94 | |
| 2 | 0.86 | 760 | 5.10 | |
| 3 | 0.88 | 13242 | 88.96 | |
| 4 | 1.19 | 446 | 3.00 | |

| Peak ID | Time |
|---|---|
| 3 | 0.88 |

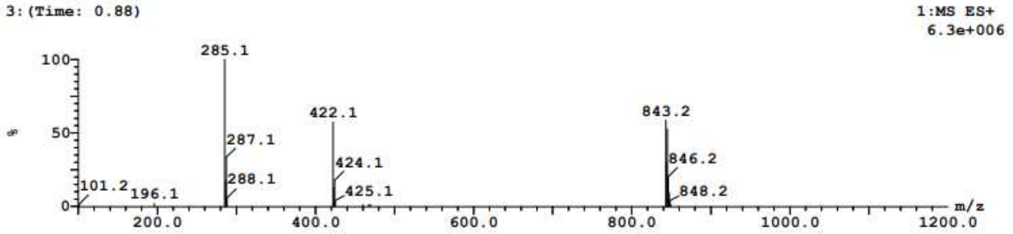


| Peak ID | Time |
|---|---|
| 3 | 0.88 |

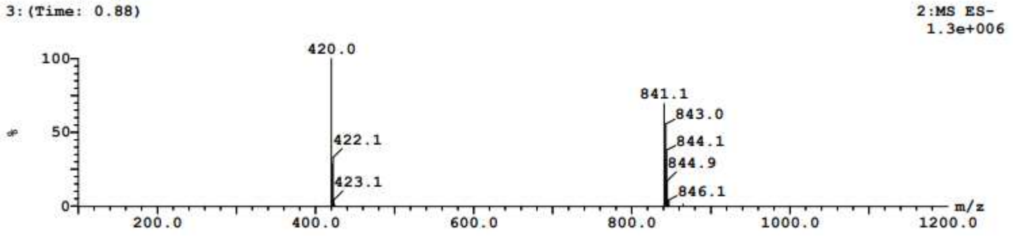

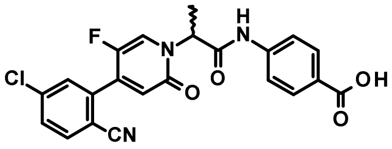

**14**

MS-MW [g/mol]: 439.0735

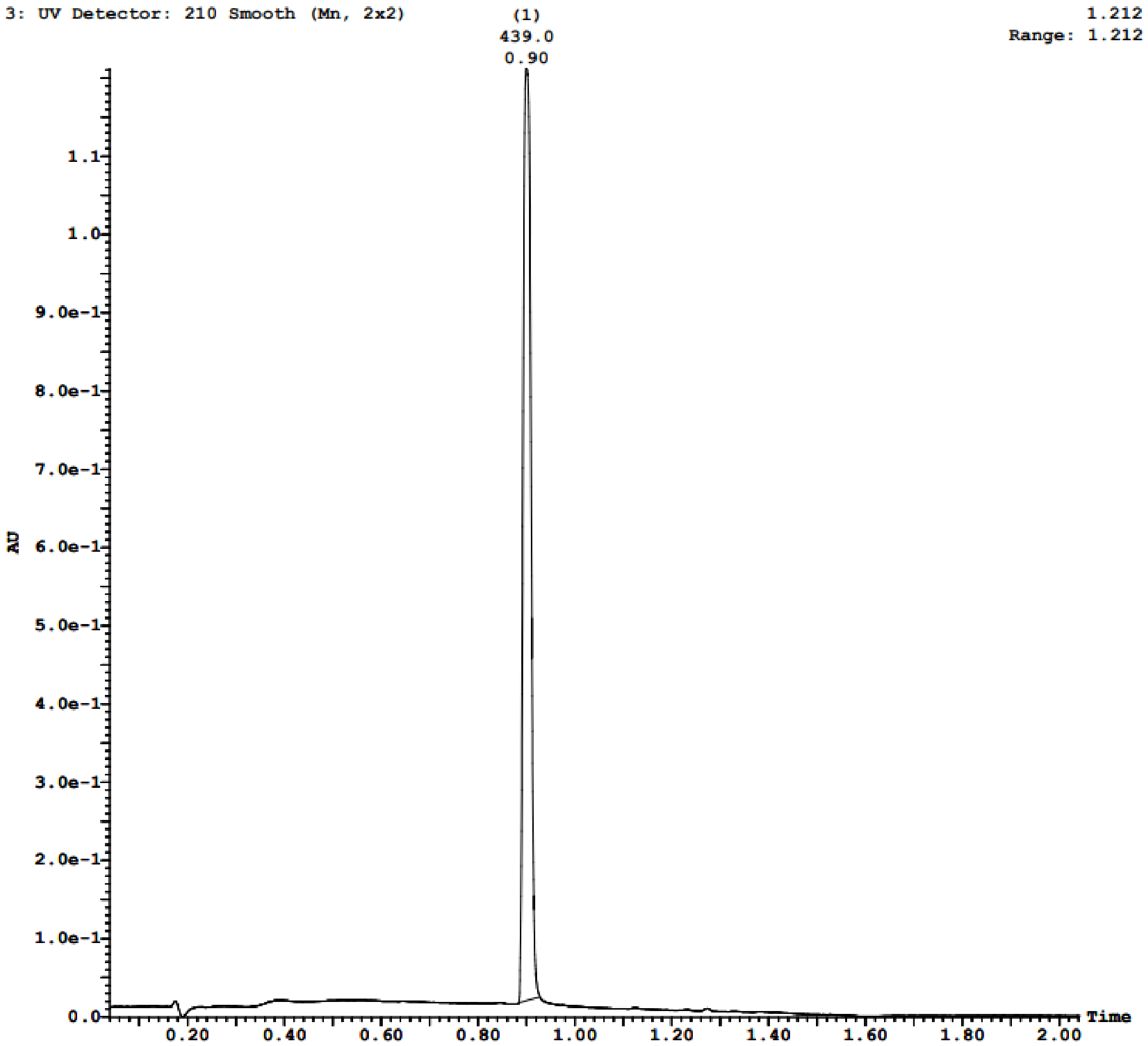


| Peak Number | Time | AreaAbs | Area %Total | Mass Found |
|---|---|---|---|---|
| 1 | 0.90 | 21992 | 100.00 | 439.00 |

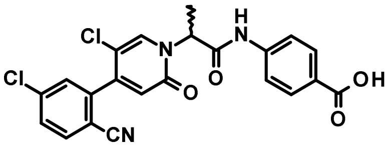

**15**

MS-MW [g/mol]: 455.0440

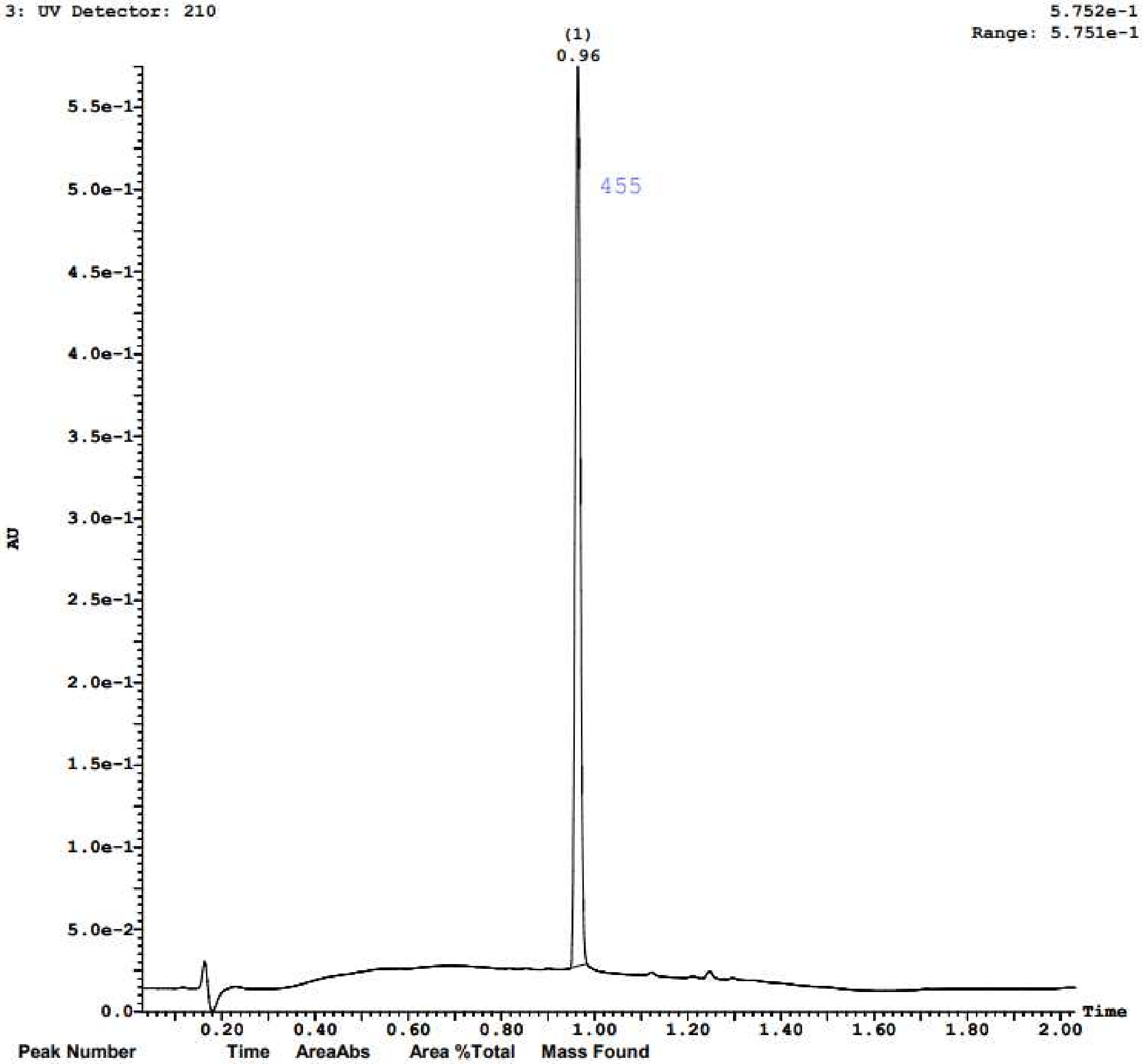


| Peak Number | Time | AreaAbs | Area %Total | Mass Found |
|---|---|---|---|---|
| 1 | 0.96 | 7055 | 100.00 | |

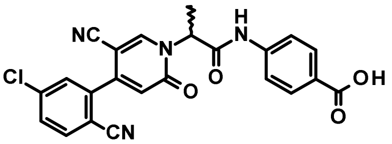


**16**

MS-MW [g/mol]: 446.0782

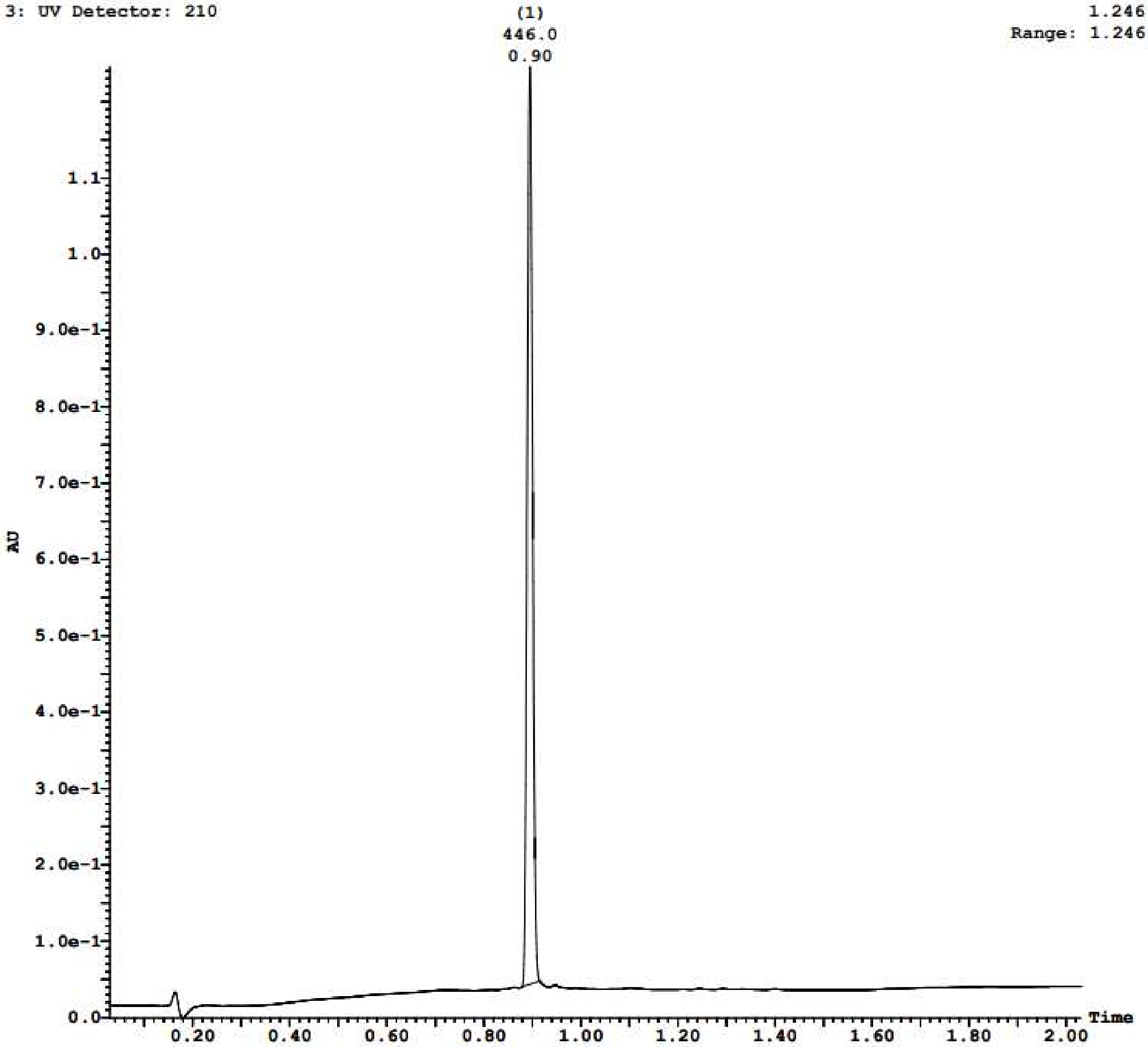


| Peak Number | Time | AreaAbs | Area %Total | Mass Found |
|---|---|---|---|---|
| 1 | 0.89 | 15518 | 100.00 | 446.00 |

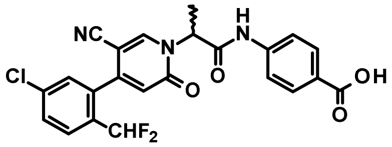


**17**

MS-MW [g/mol]: 471.0797

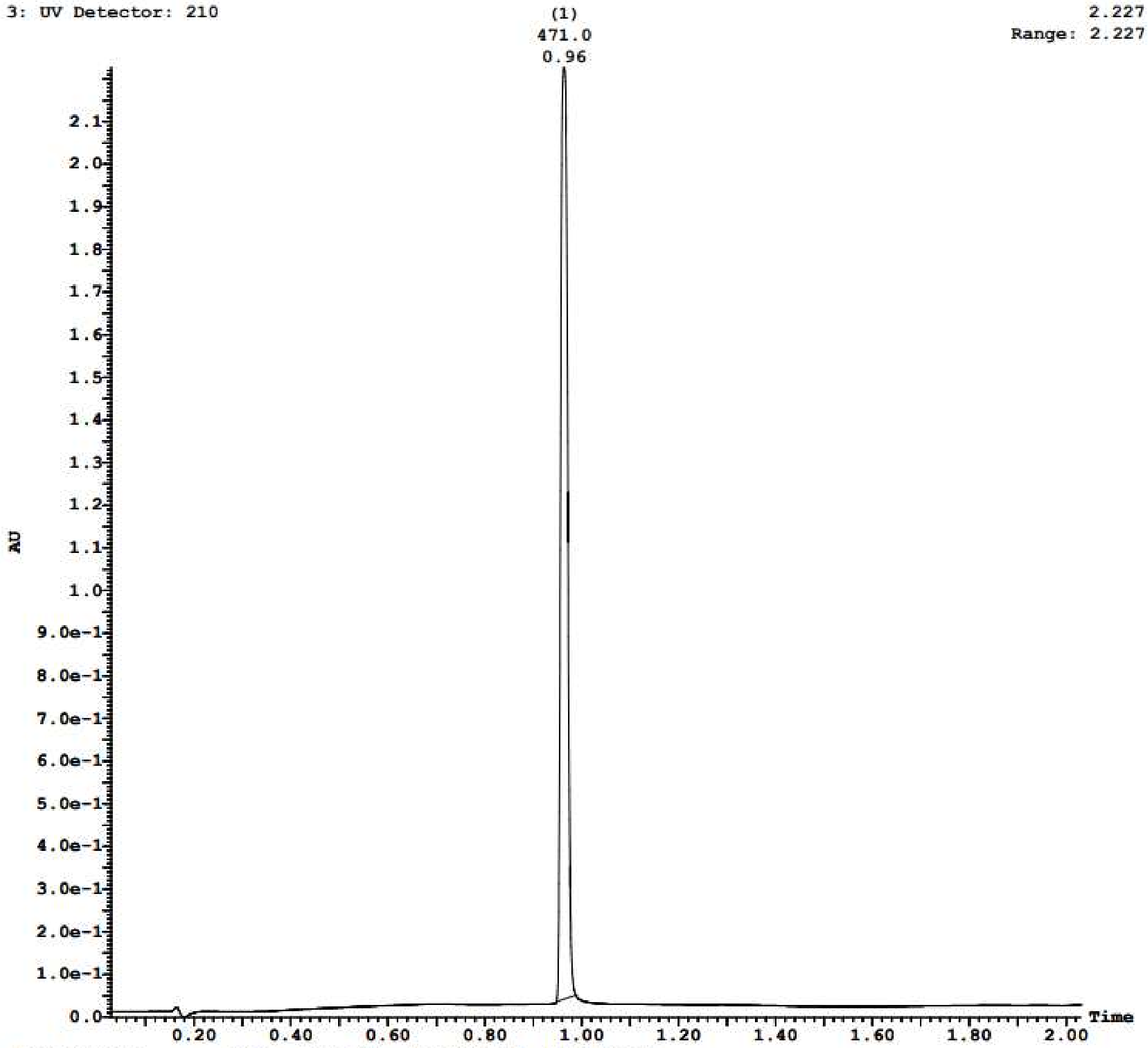


| Peak Number | Time | AreaAbs | Area %Total | Mass Found |
|---|---|---|---|---|
| 1 | 0.96 | 35967 | 100.00 | 471.00 |

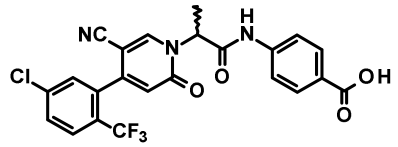


**18**

MS-MW [g/mol]: 489.0703

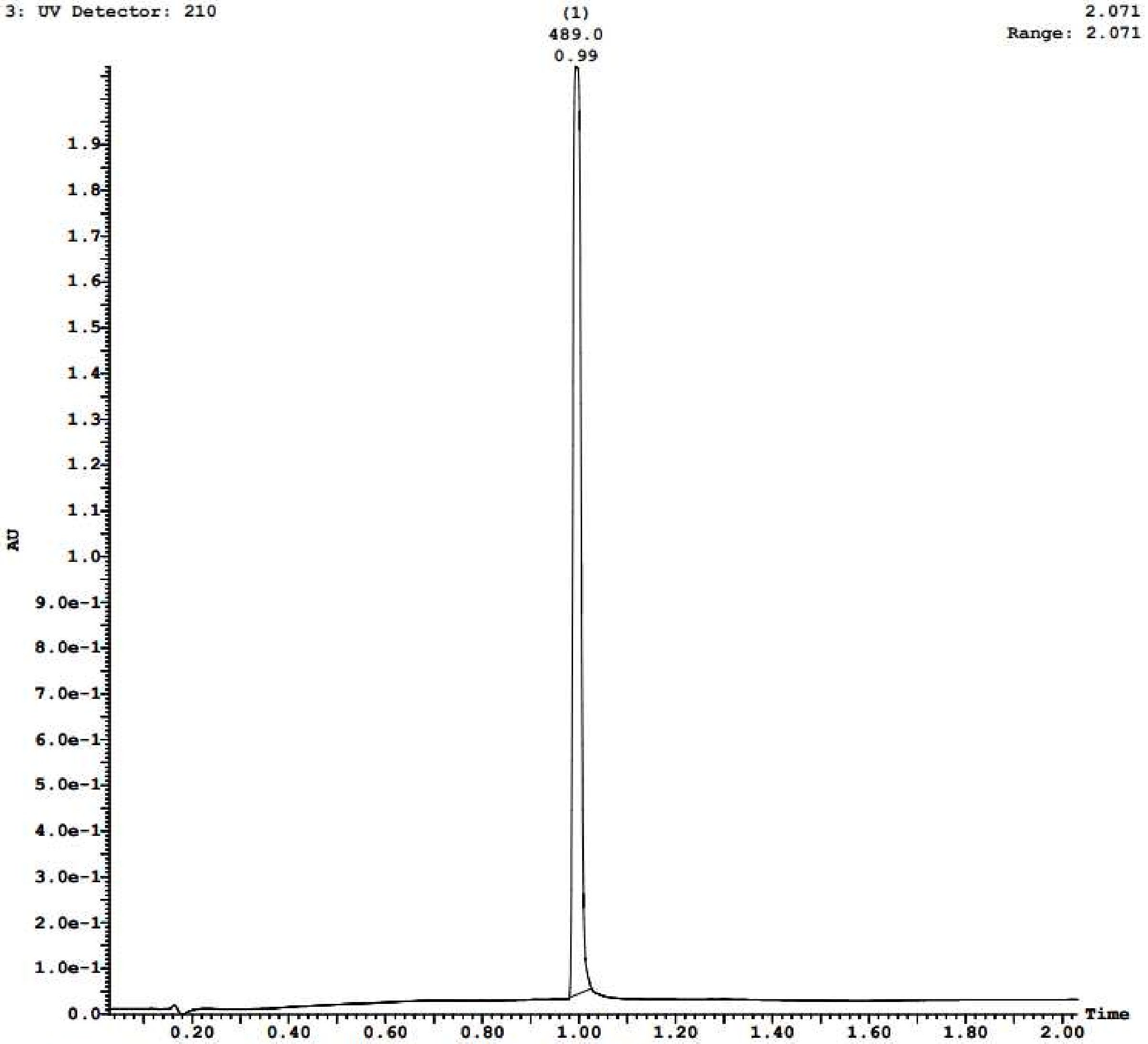


| Peak Number | Time | AreaAbs | Area %Total | Mass Found |
|---|---|---|---|---|
| 1 | 0.99 | 37964 | 100.00 | 489.00 |

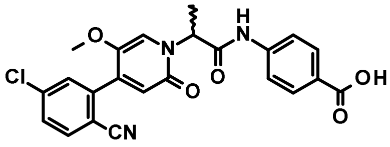


**19**

MS-MW [g/mol]: 451.0935

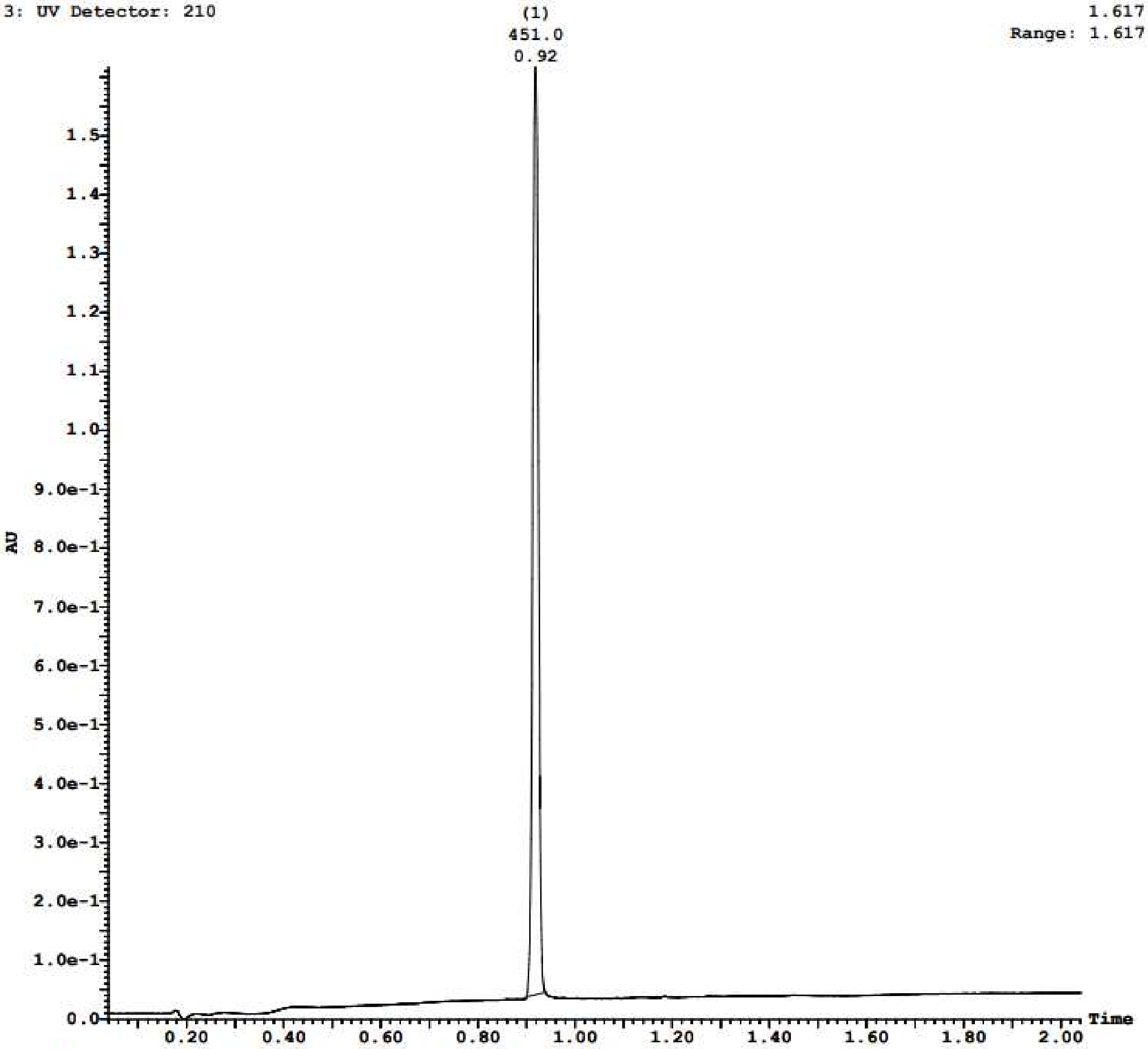


| Peak Number | Time | AreaAbs | Area %Total | Mass Found |
|---|---|---|---|---|
| 1 | 0.92 | 21642 | 100.00 | 451.00 |

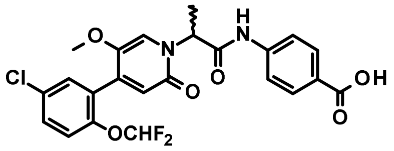


**20**

MS-MW [g/mol]: 492.0900

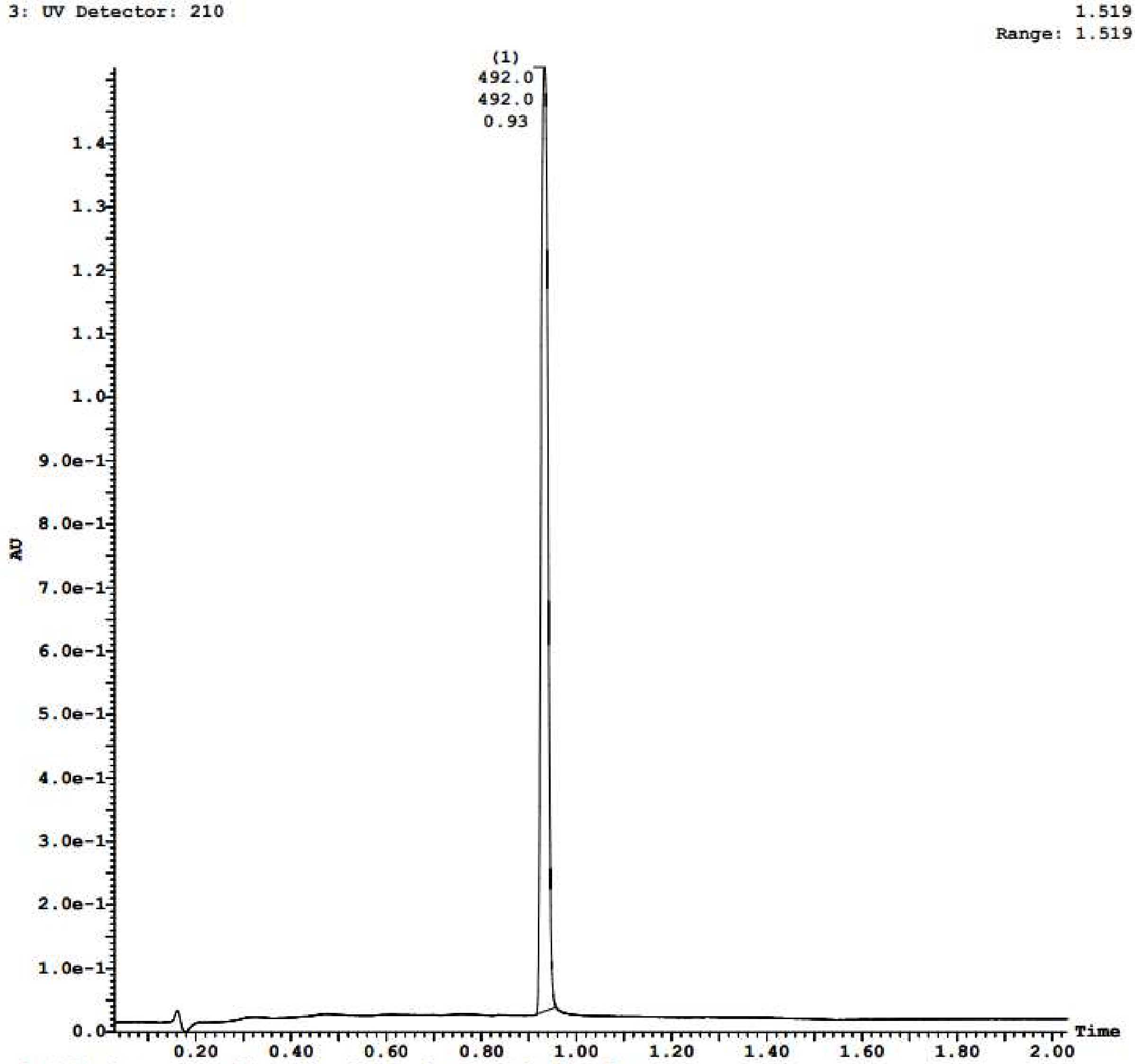


| Peak Number | Time | AreaAbs | Area %Total | Mass Found |
|---|---|---|---|---|
| 1 | 0.93 | 24867 | 100.00 | 492.00, 492.00 |

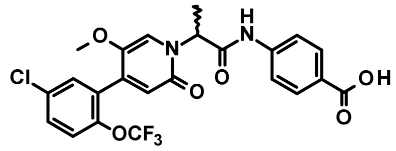

**21**

MS-MW [g/mol]: 510.0805

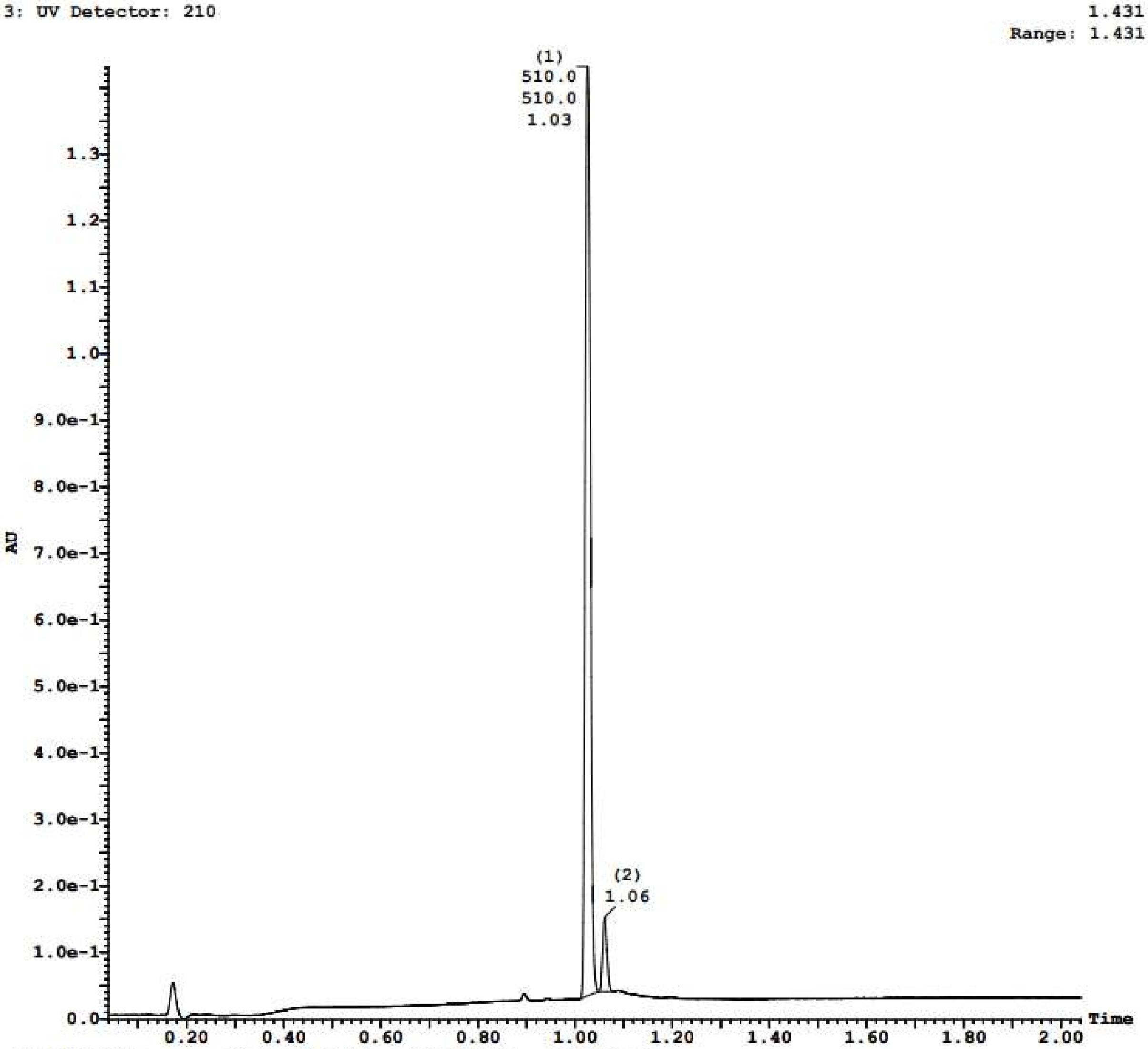


| Peak Number | Time | AreaAbs | Area %Total | Mass Found |
|---|---|---|---|---|
| 1 | 1.03 | 17278 | 93.65 | 510.00, 510.00 |
| 2 | 1.06 | 1172 | 6.35 | |

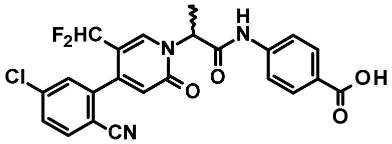


**22**

MS-MW [g/mol]: 471.0797

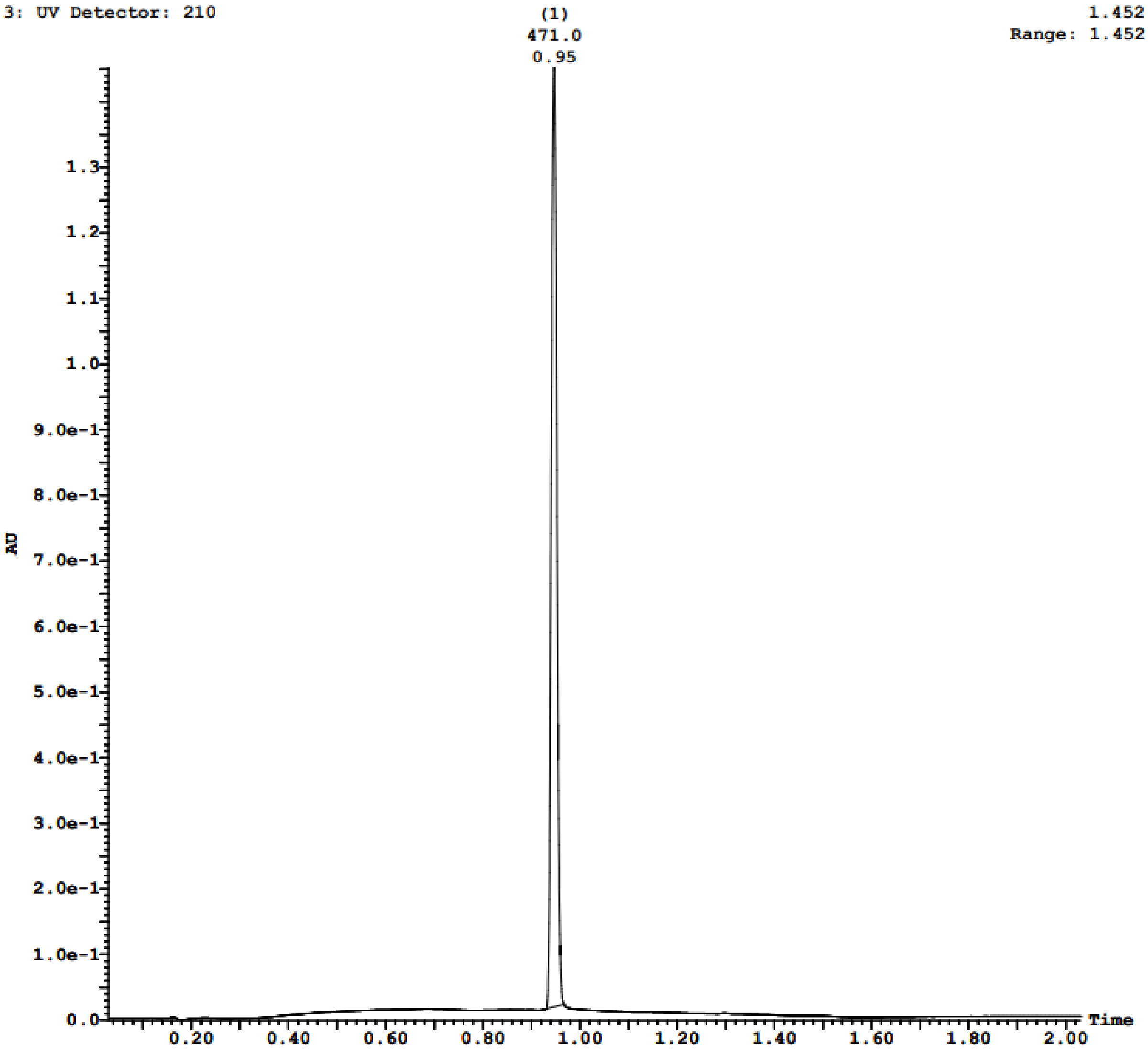


| Peak Number | Time | AreaAbs | Area %Total | Mass Found |
|---|---|---|---|---|
| 1 | 0.95 | 18892 | 100.00 | 471.00 |

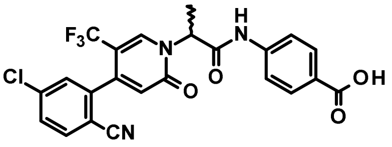


**23**

MS-MW [g/mol]: 489.0703

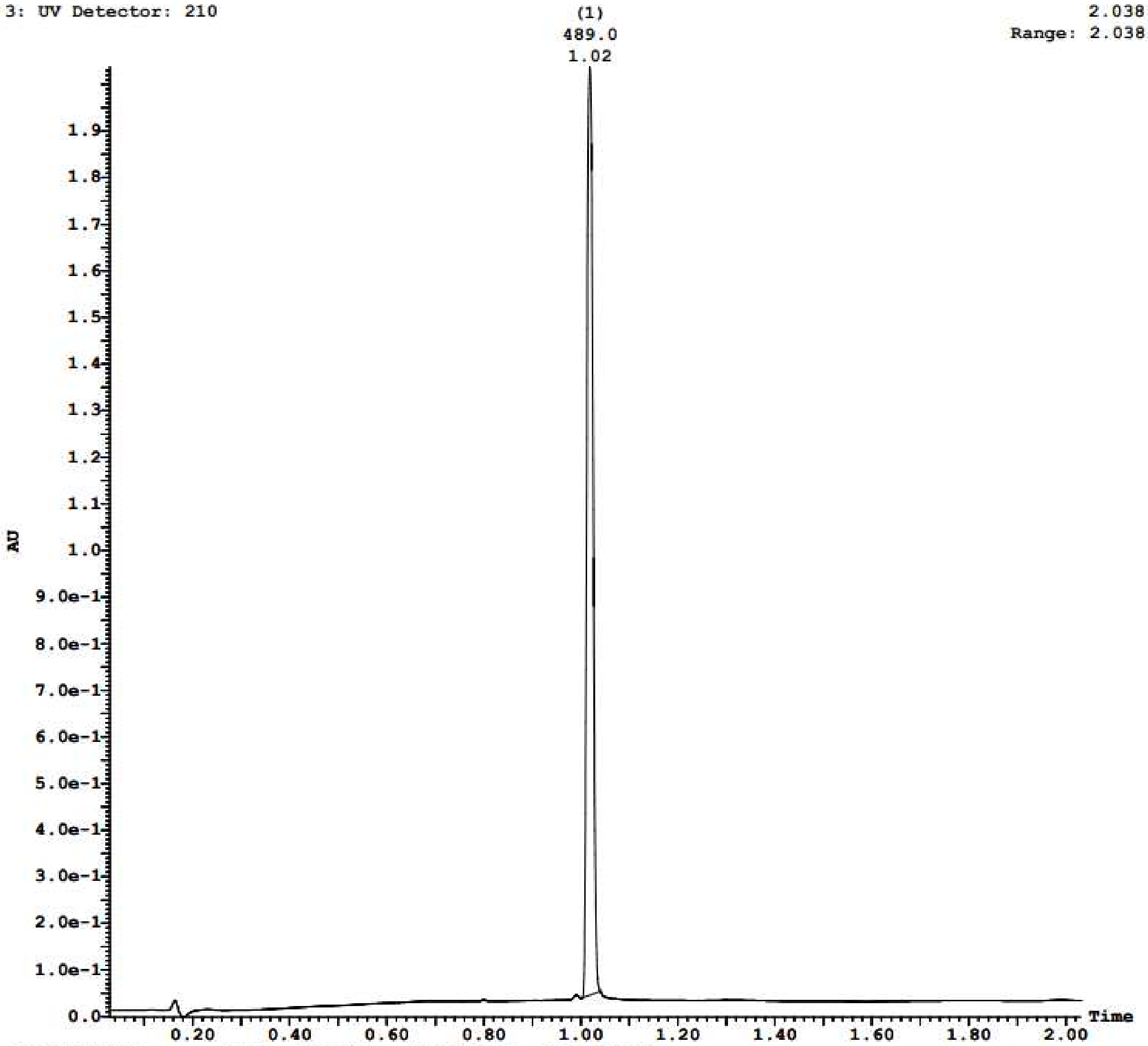


| Peak Number | Time | AreaAbs | Area %Total | Mass Found |
|---|---|---|---|---|
| 1 | 1.02 | 28903 | 100.00 | 489.00 |

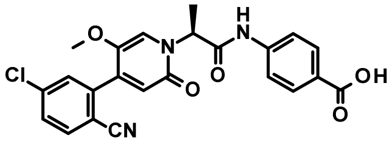


**24**

MS-MW [g/mol]: 451.0935

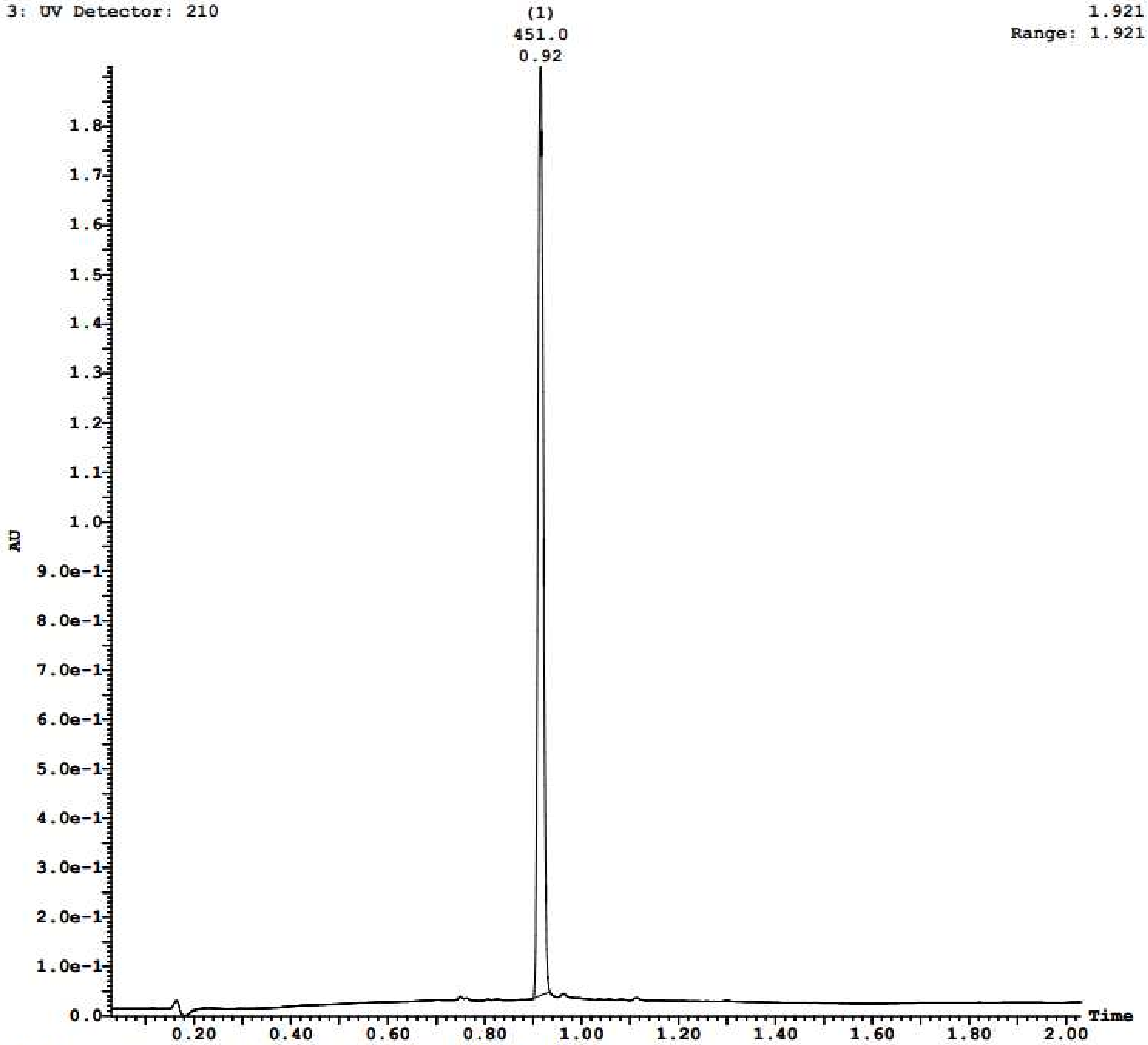


| Peak Number | Time | AreaAbs | Area %Total | Mass Found |
|---|---|---|---|---|
| 1 | 0.92 | 24306 | 100.00 | 451.00 |

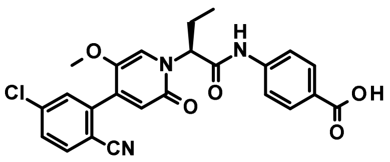

**25**

MS-MW [g/mol]: 465.1091

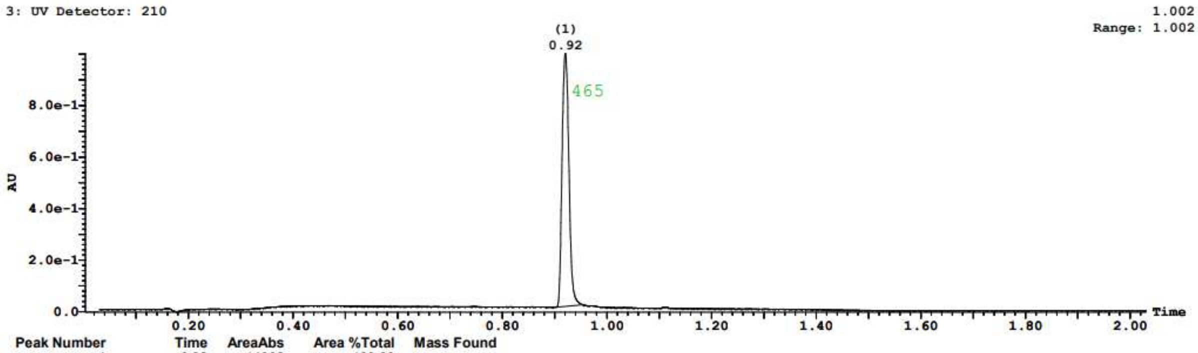


| Peak Number | Time | AreaAbs | Area %Total | Mass Found |
|---|---|---|---|---|
| 1 | 0.92 | 14902 | 100.00 | |

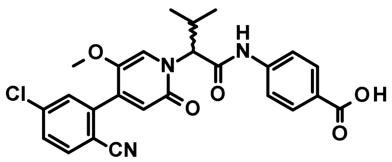

**26**

MS-MW [g/mol]: 479.1248

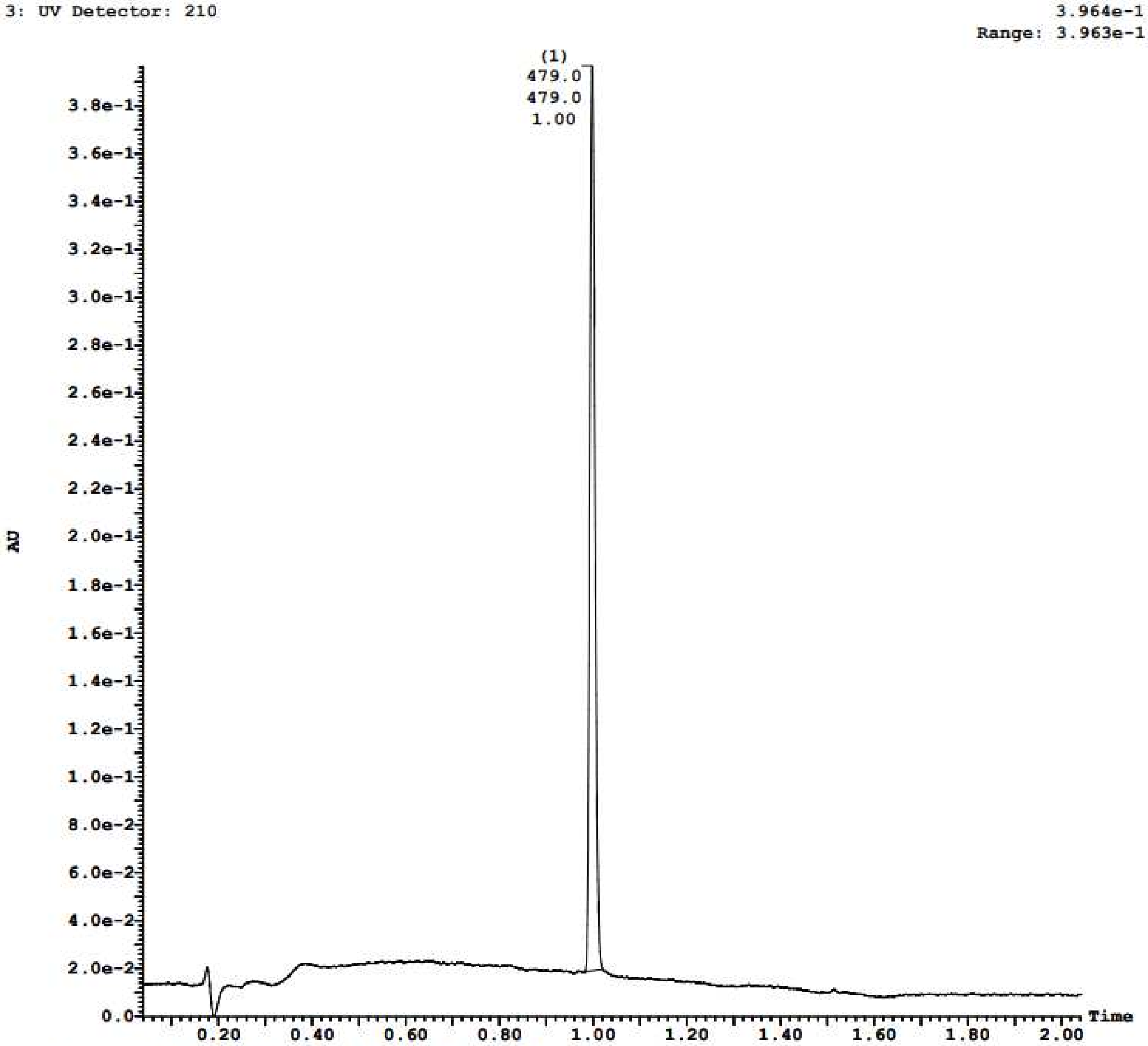


| Peak Number | Time | AreaAbs | Area %Total | Mass Found |
|---|---|---|---|---|
| 1 | 1.00 | 4818 | 100.00 | 479.00, 479.00 |

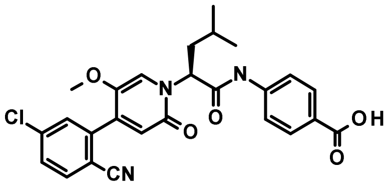

**27**

MS-MW [g/mol]: 493.1404

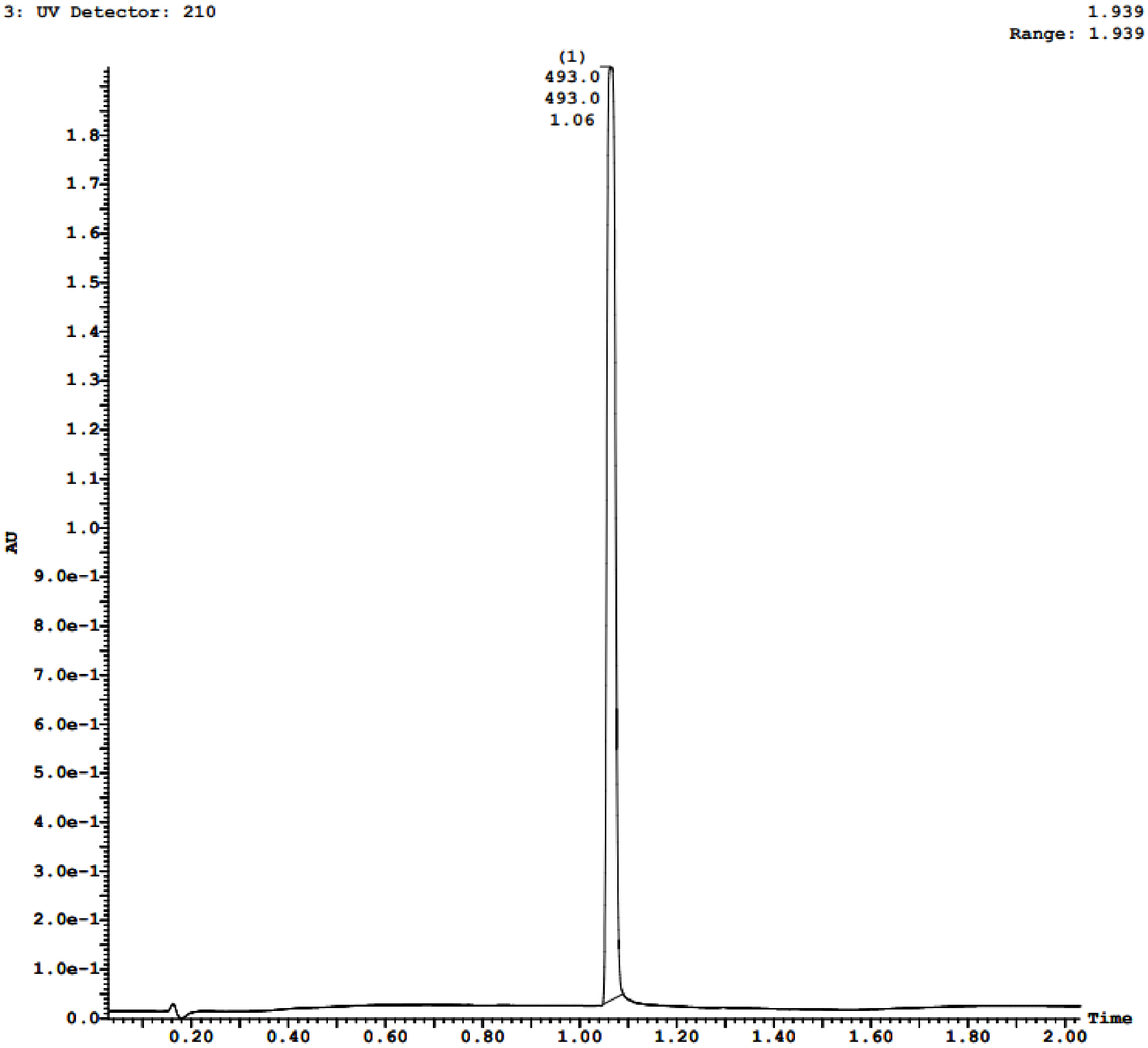


| Peak Number | Time | AreaAbs | Area %Total | Mass Found |
|---|---|---|---|---|
| 1 | 1.06 | 37793 | 100.00 | 493.00, 493.00 |

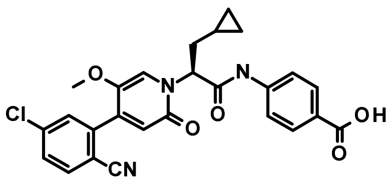

**28**

MS-MW [g/mol]: 491.1248

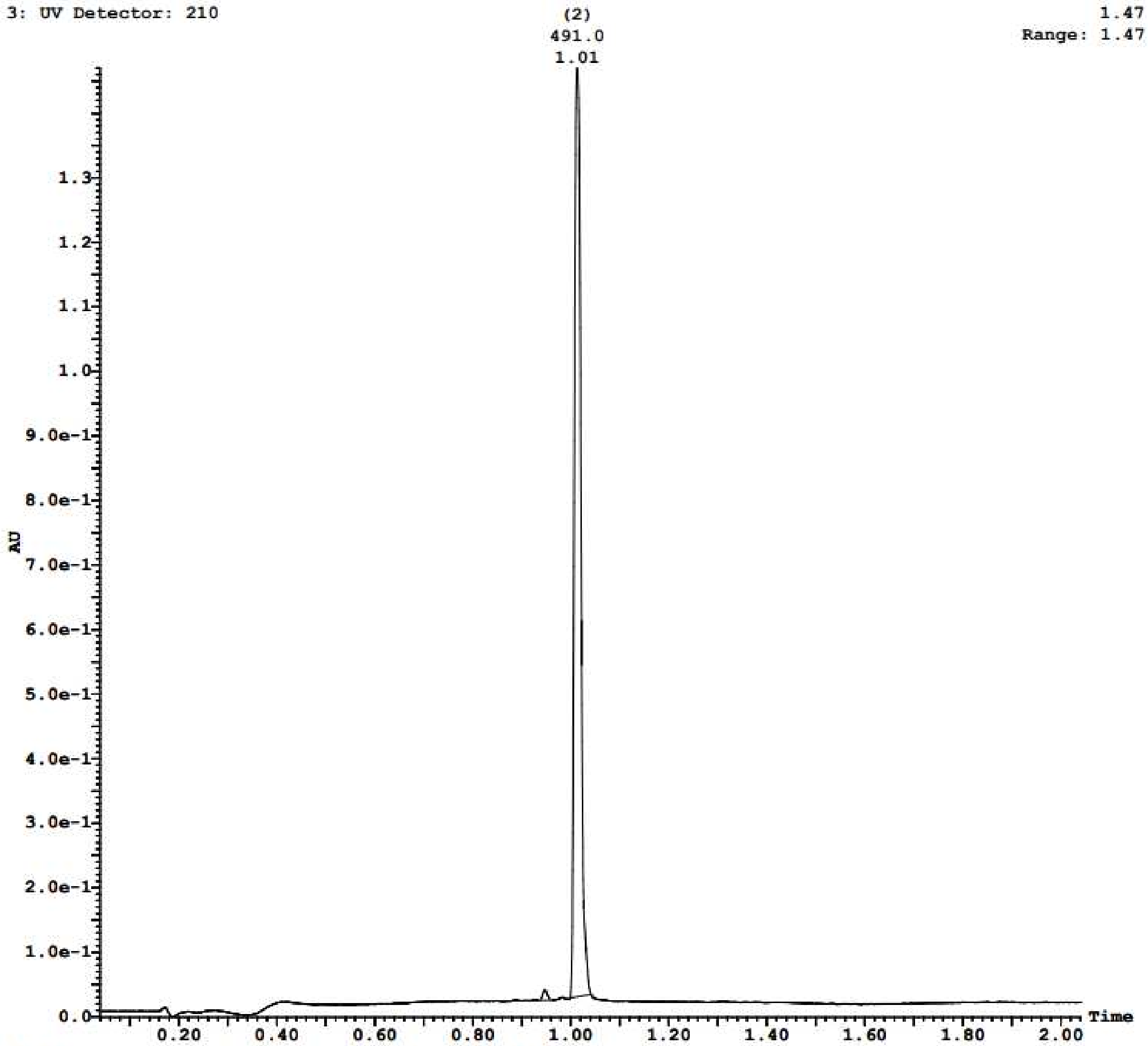


| Peak Number | Time | AreaAbs | Area %Total | Mass Found |
|---|---|---|---|---|
| 1 | 0.95 | 175 | 0.79 | |
| 2 | 1.01 | 21891 | 99.21 | 491.00 |

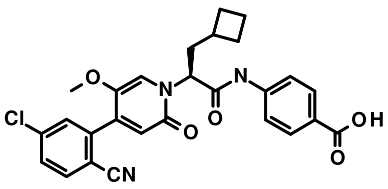

**29**

MS-MW [g/mol]: 505.1404

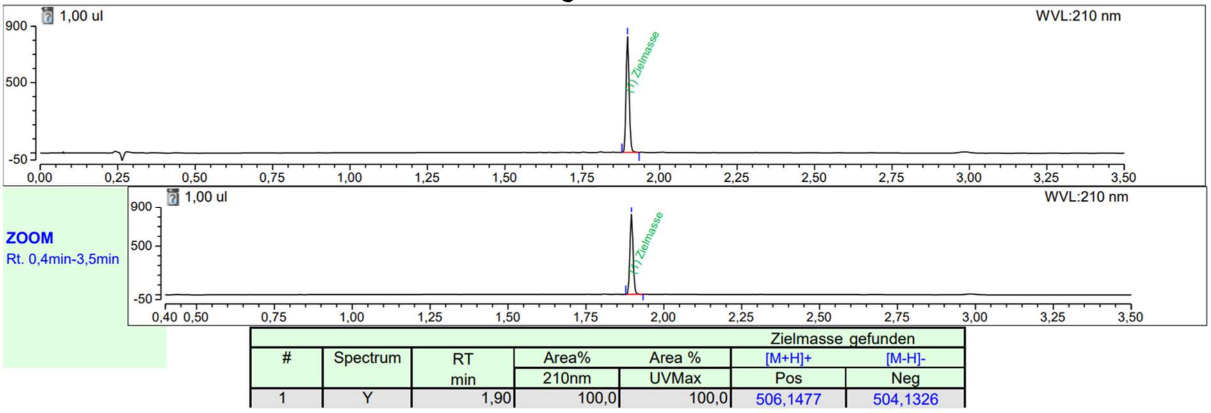


| # | Spectrum | RT | Area% | Area % | Zielmasse gefunden | |
|---|---|---|---|---|---|---|
| | | | | | [M+H]+ | [M-H]- |
| | | min | 210nm | UVMax | Pos | Neg |
| 1 | Y | 1,90 | 100,0 | 100,0 | 506,1477 | 504,1326 |

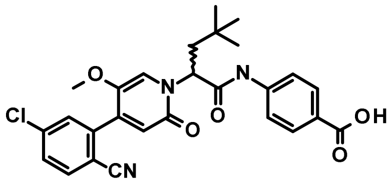


**30**

MS-MW [g/mol]: 507.1561

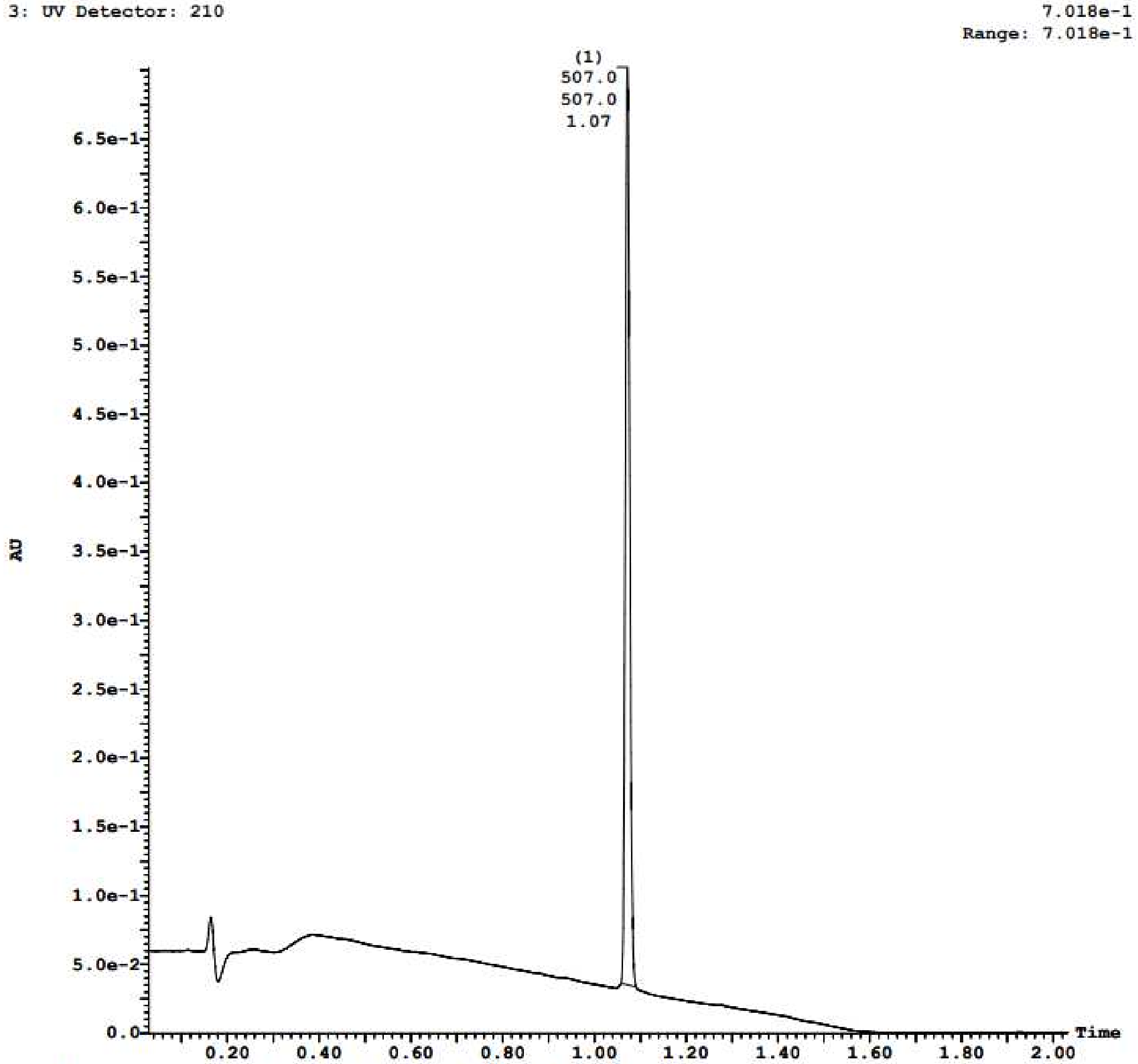


| Peak Number | Time | AreaAbs | Area %Total | Mass Found |
|---|---|---|---|---|
| 1 | 1.07 | 7546 | 100.00 | 507.00, 507.00 |

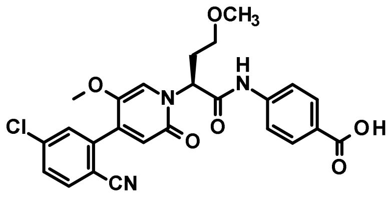


**31**

MS-MW [g/mol]: 495.1197

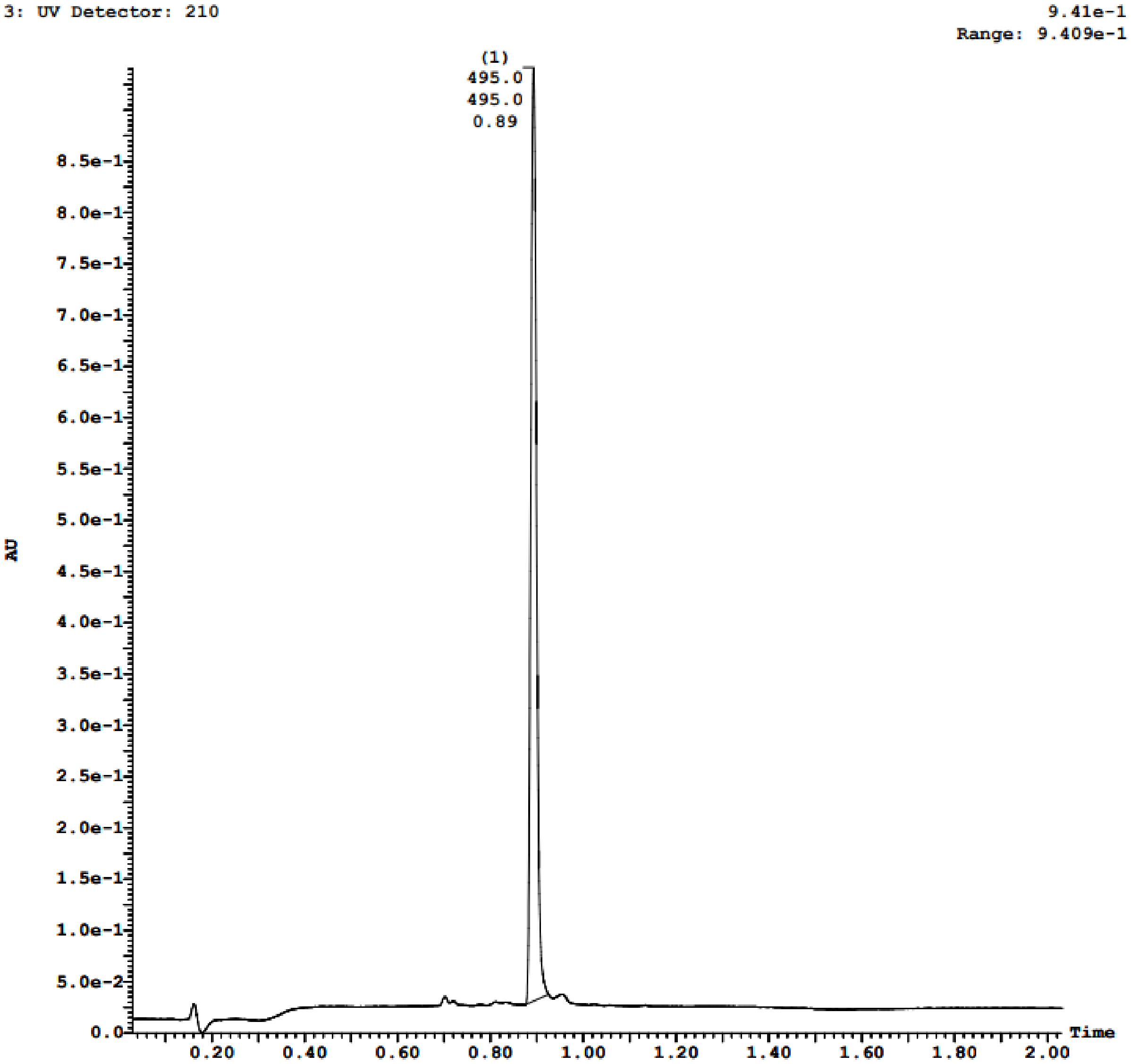


| Peak Number | Time | AreaAbs | Area %Total | Mass Found |
|---|---|---|---|---|
| 1 | 0.89 | 13082 | 100.00 | 495.00, 495.00 |

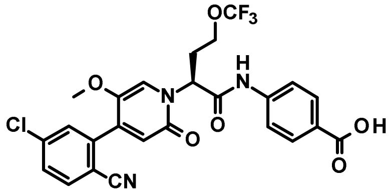

**32**

MS-MW [g/mol]: 549.0914

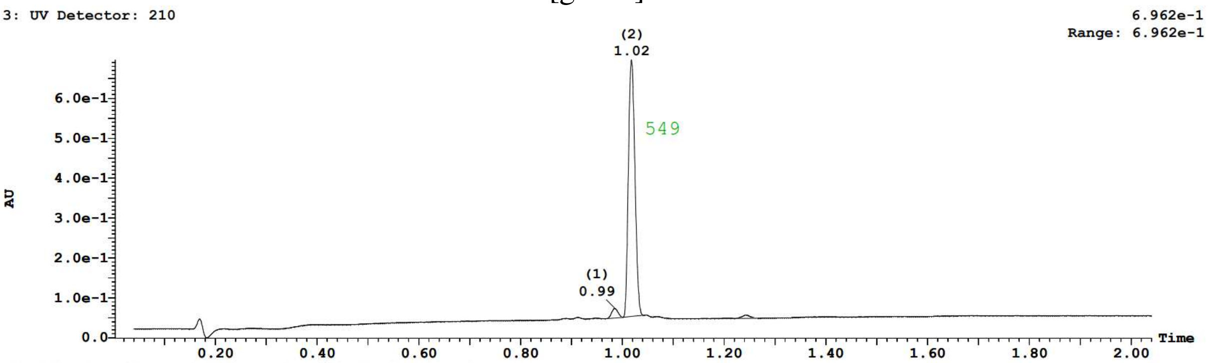


| Peak Number | Time | AreaAbs | Area %Total | Mass Found |
|---|---|---|---|---|
| 1 | 0.99 | 346 | 3.43 | |
| 2 | 1.02 | 9575 | 95.05 | |
| 3 | 1.24 | 153 | 1.52 | |

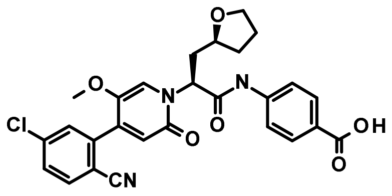

**33**

MS-MW [g/mol]: 521.1354

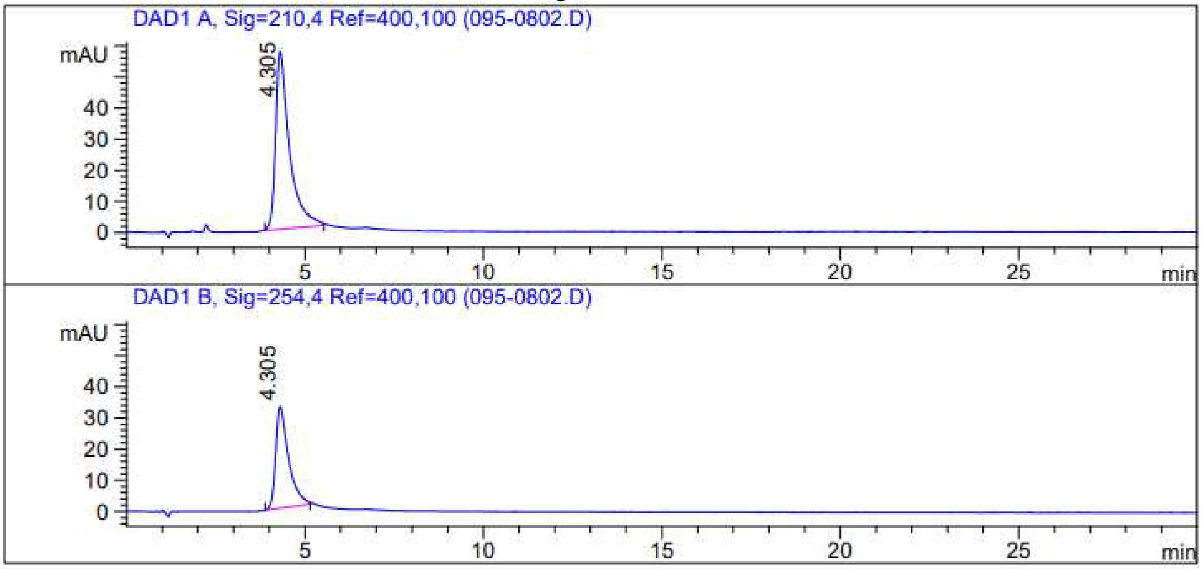


Report:

| # Peak | RetTime [Min] | Area [mAU*s] | Area % [%] | Height [mAU] | Height [%] | Width [Min] | Signal Desc. |
|---|---|---|---|---|---|---|---|
| 1 | 4.305 | 818.40 | 100.00 | 32.63 | 100.00 | 0.36 | DAD1 B, Sig=254 |
| 2 | 4.305 | 1545.42 | 100.00 | 57.06 | 100.00 | 0.39 | DAD1 A, Sig=210 |

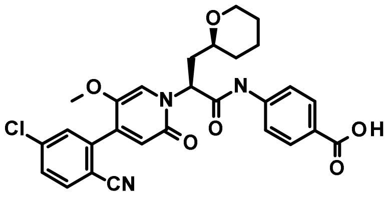


**34**

MS-MW [g/mol]: 535.1510

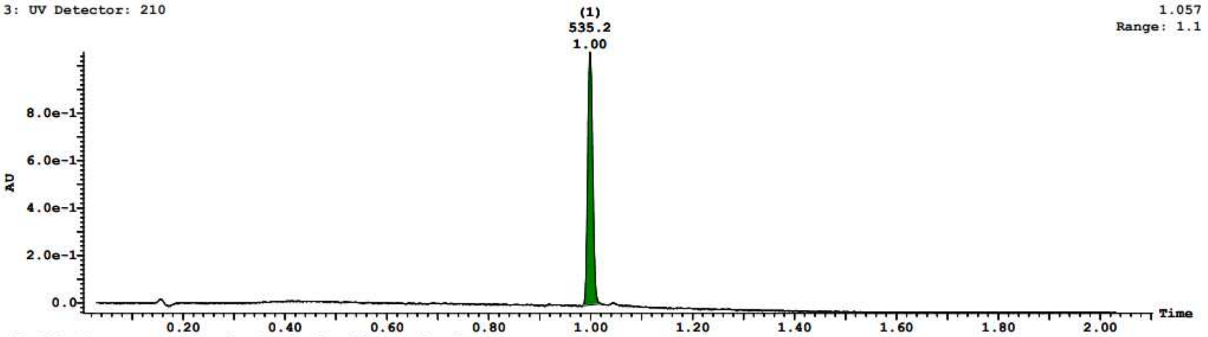


| Peak Number | Time | AreaAbs | Area %Total | Mass Found |
|---|---|---|---|---|
| 1 | 1.00 | 12039 | 100.00 | 535.15 |

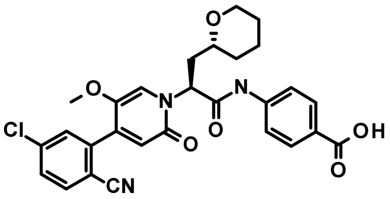


**35**

MS-MW [g/mol]: 535.1510

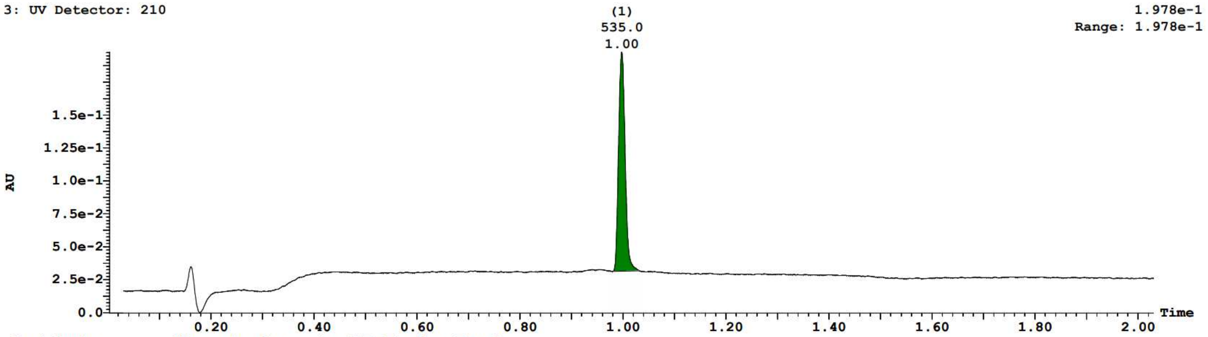


| Peak Number | Time | AreaAbs | Area %Total | Mass Found |
|---|---|---|---|---|
| 1 | 1.00 | 2464 | 100.00 | 535.00 |

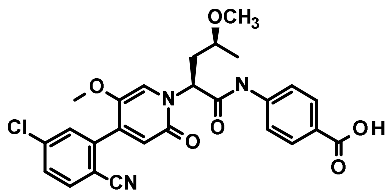


**36**

MS-MW [g/mol]: 509.1354

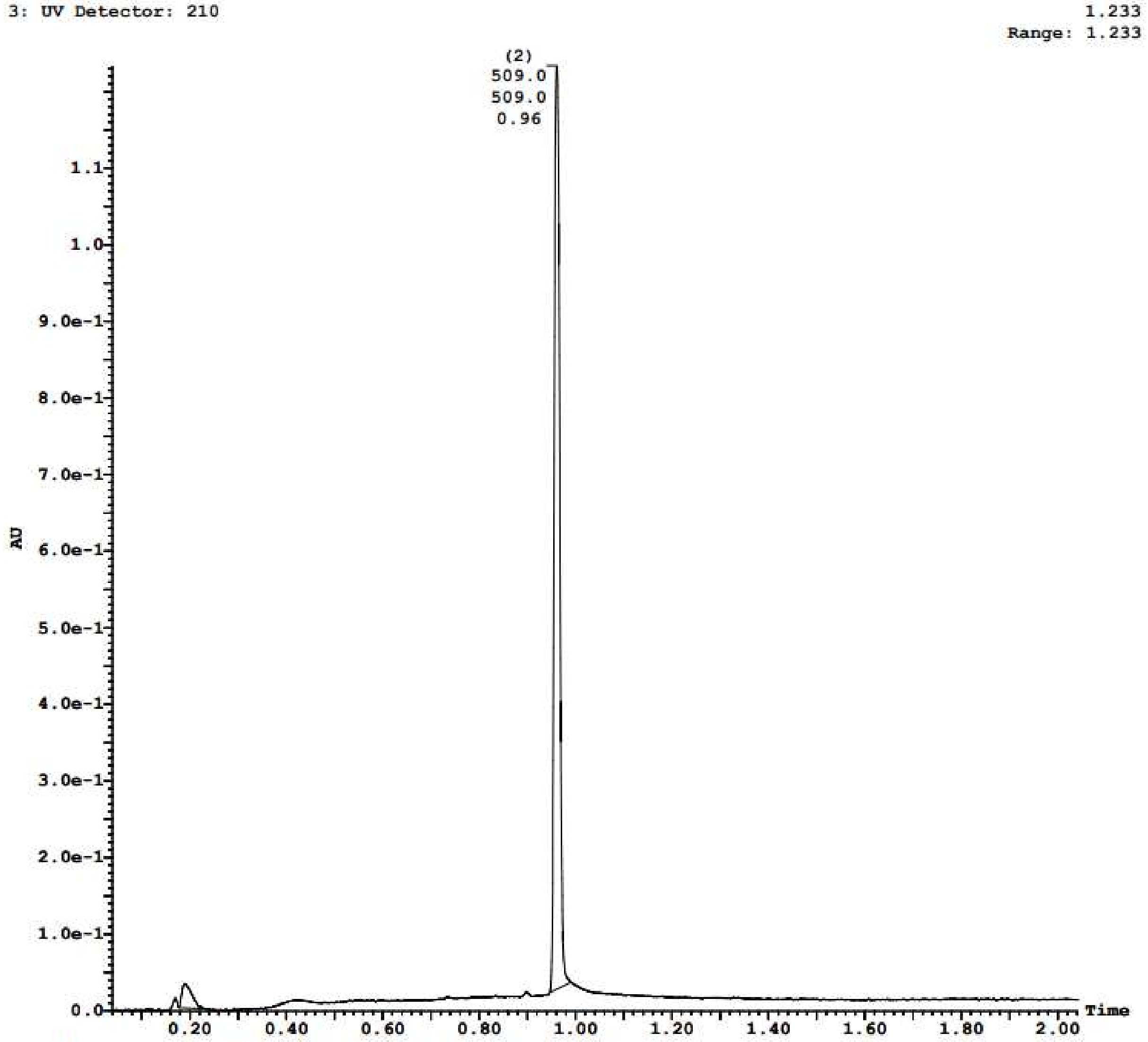


| Peak Number | Time | AreaAbs | Area %Total | Mass Found |
|---|---|---|---|---|
| 1 | 0.19 | 753 | 4.54 | |
| 2 | 0.96 | 15832 | 95.46 | 509.00, 509.00 |

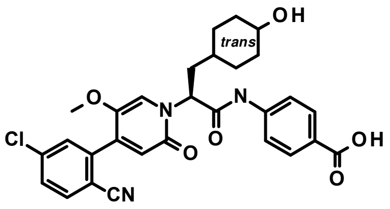


**37**

MS-MW [g/mol]: 549.1667

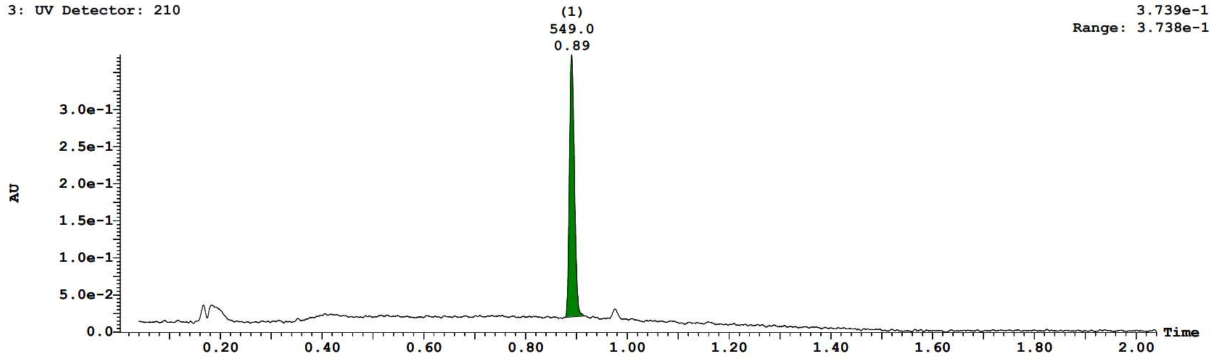


| Peak Number | Time | AreaAbs | Area %Total | Mass Found |
|---|---|---|---|---|
| 1 | 0.89 | 4060 | 100.00 | 549.00 |

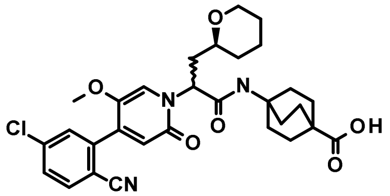


**38**

MS-MW [g/mol]: 567.2136

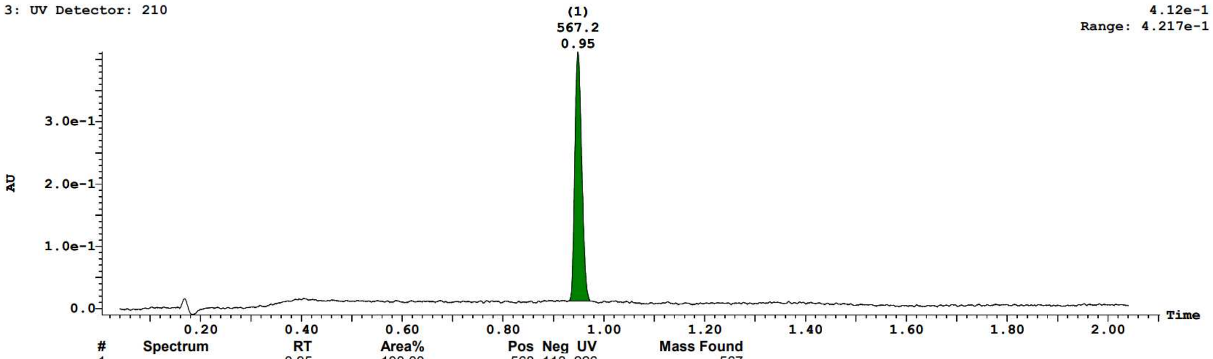


| # | Spectrum | RT | Area% | Pos Neg UV | Mass Found |
|---|---|---|---|---|---|
| 1 | --- | 0.95 | 100.00 | 568, 113, 226 | 567 |

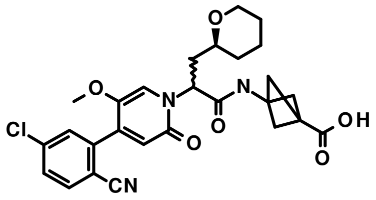


**39**

MS-MW [g/mol]: 525.1667

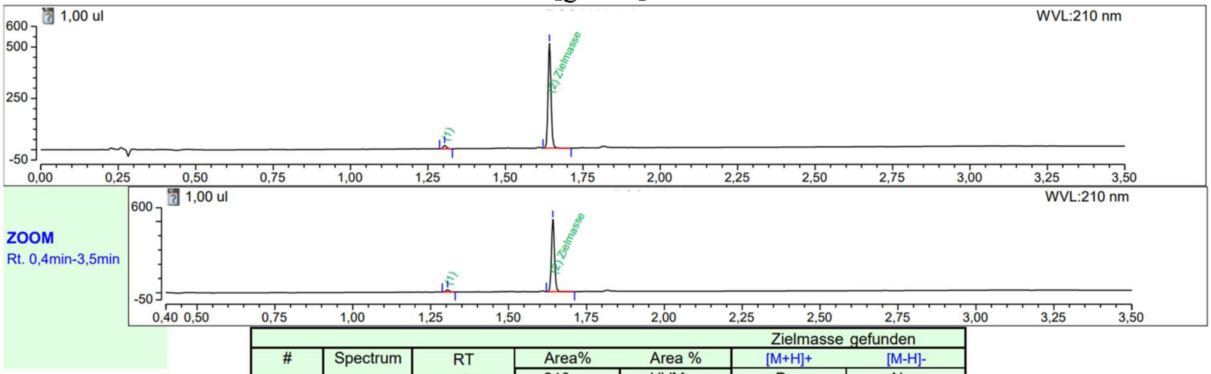


| # | Spectrum | RT min | Area% 210nm | Area % UVMax | Zielmasse gefunden [M+H]+ Pos | [M-H]- Neg |
|---|---|---|---|---|---|---|
| 1 | Y | 1,30 | 3,4 | 2,9 | | |
| 2 | Y | 1,64 | 96,6 | 97,1 | 526,1740 | 524,1594 |

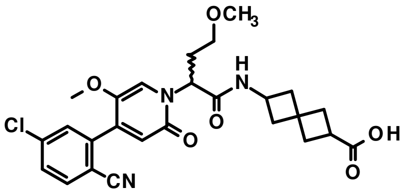


**40**

MS-MW [g/mol]: 513.1667

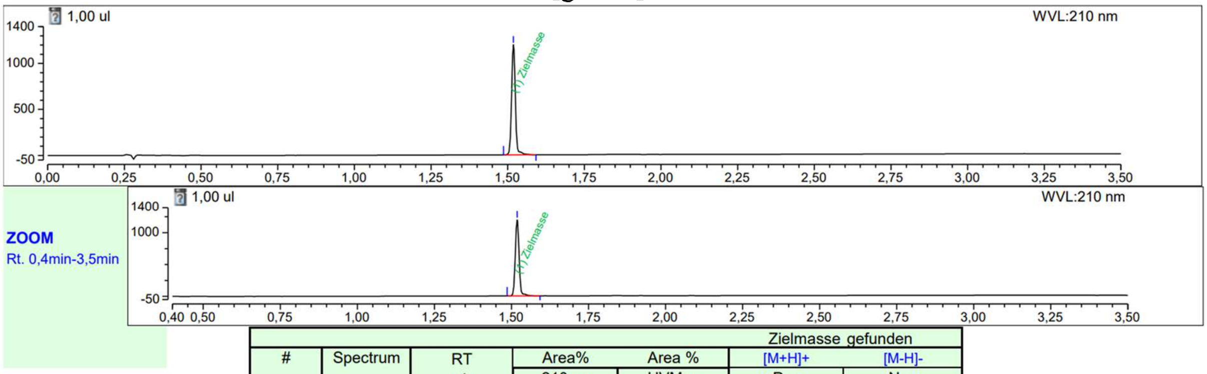


| # | Spectrum | RT min | Area% 210nm | Area % UVMax | Zielmasse gefunden [M+H]+ Pos | [M-H]- Neg |
|---|---|---|---|---|---|---|
| 1 | Y | 1,52 | 100,0 | 100,0 | 514,1740 | 512,1594 |

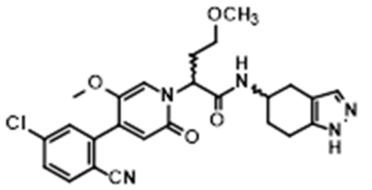

**41**

MS-MW [g/mol]: 495.1673

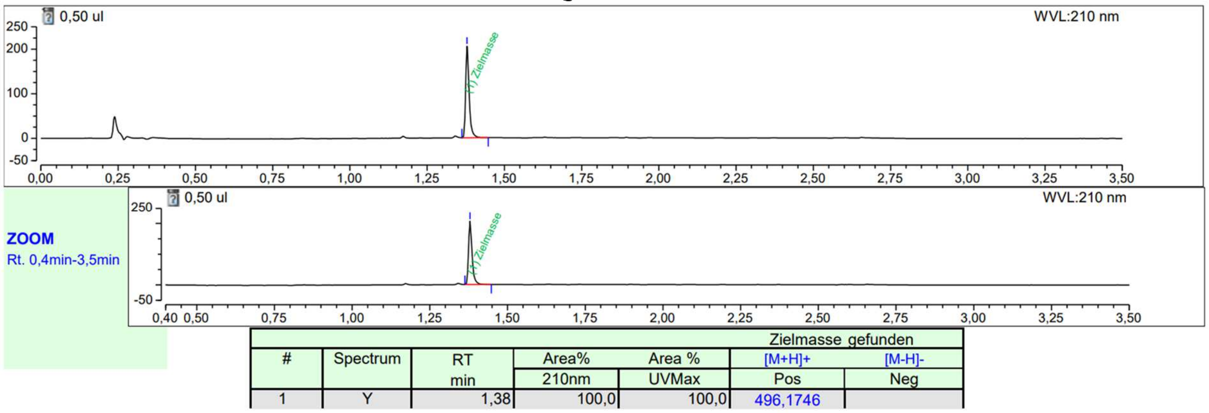


| # | Spectrum | RT min | Area% 210nm | Area % UVMax | Zielmasse gefunden [M+H]+ Pos | [M-H]- Neg |
|---|---|---|---|---|---|---|
| 1 | Y | 1,38 | 100,0 | 100,0 | 496,1746 | |

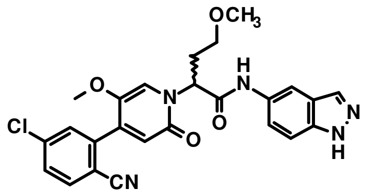

**42**

MS-MW [g/mol]: 491.1360

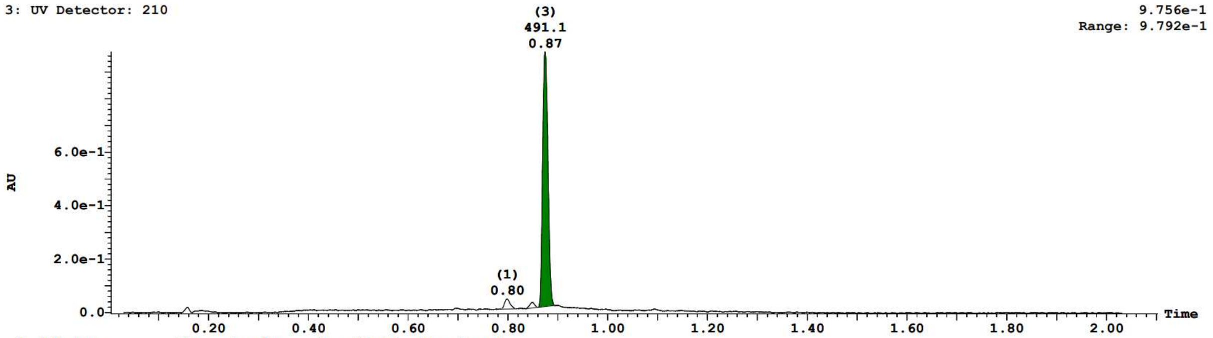


| Peak Number | Time | AreaAbs | Area %Total | Mass Found |
|---|---|---|---|---|
| 1 | 0.80 | 495 | 3.92 | |
| 2 | 0.85 | 234 | 1.85 | |
| 3 | 0.87 | 11913 | 94.23 | 491.14 |

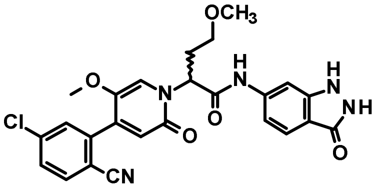

**43**

MS-MW [g/mol]: 507.1309

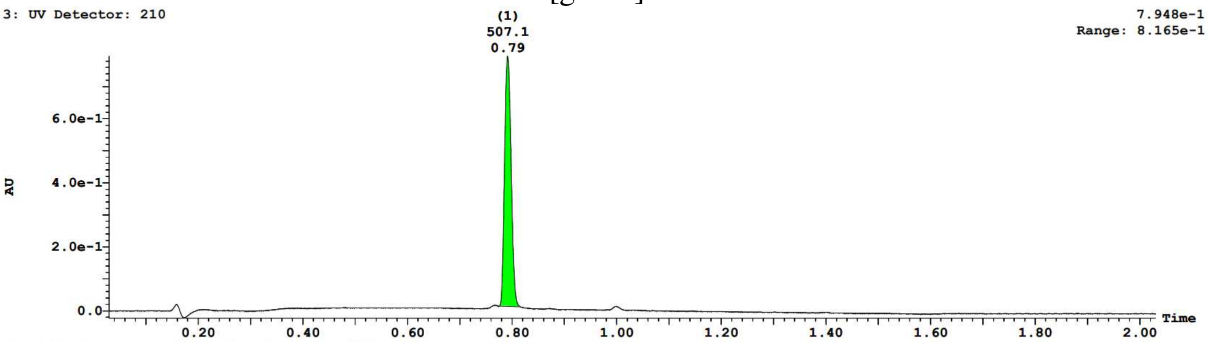


| Peak Number | Time | AreaAbs | Area %Total | Mass Found |
|---|---|---|---|---|
| 1 | 0.79 | 11324 | 100.00 | 507.13 |

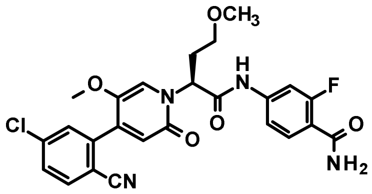

**44**

MS-MW [g/mol]: 512.1263

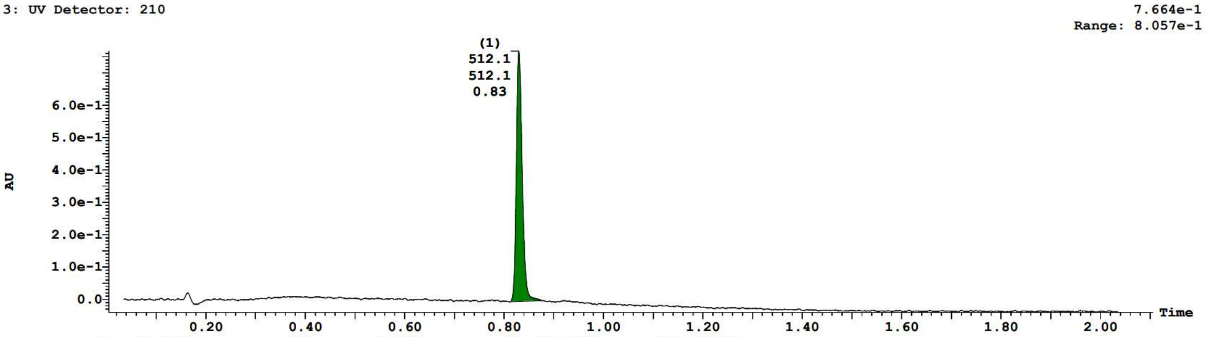


| # | Spectrum | RT | Area% | Pos Neg UV | Mass Found |
|---|---|---|---|---|---|
| 1 | --- | 0.83 | 100.00 | 513, 511, 227 | 512, 512 |

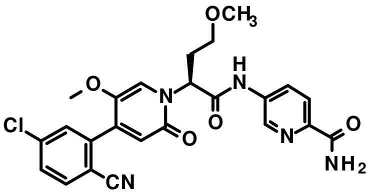


**45**

MS-MW [g/mol]: 495.1309

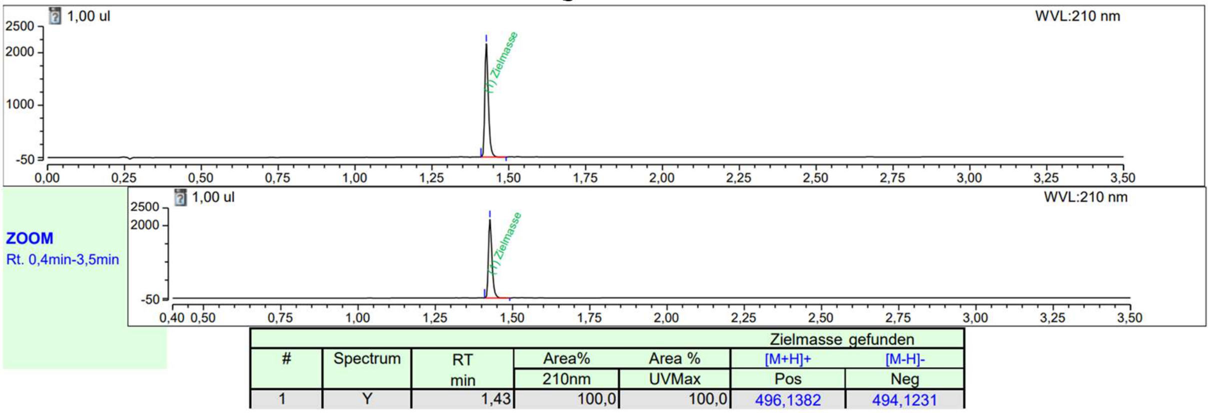


| # | Spectrum | RT min | Area% 210nm | Area % UVMax | Zielmasse gefunden [M+H]+ Pos | [M-H]- Neg |
|---|---|---|---|---|---|---|
| 1 | Y | 1,43 | 100,0 | 100,0 | 496,1382 | 494,1231 |

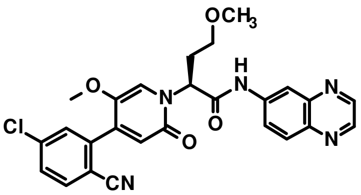


**46**

MS-MW [g/mol]: 503.1360

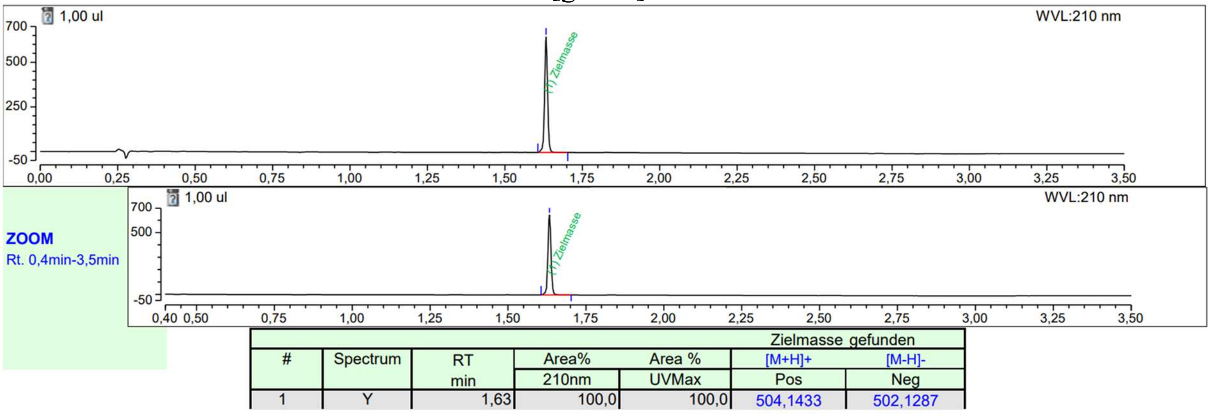


| # | Spectrum | RT min | Area% 210nm | Area % UVMax | Zielmasse gefunden [M+H]+ Pos | [M-H]- Neg |
|---|---|---|---|---|---|---|
| 1 | Y | 1,63 | 100,0 | 100,0 | 504,1433 | 502,1287 |

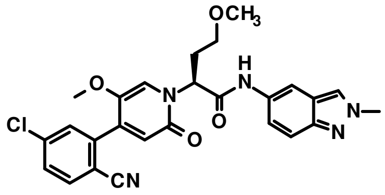

**47**

MS-MW [g/mol]: 505.1517

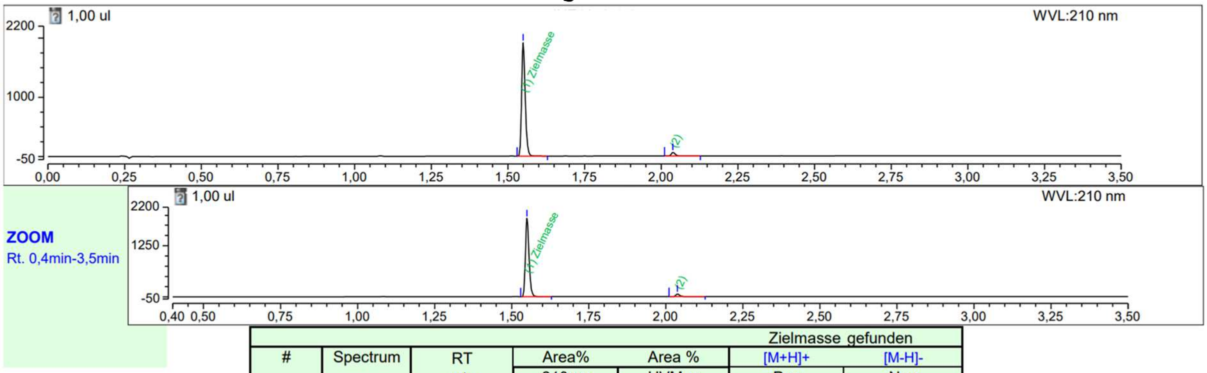


| # | Spectrum | RT min | Area% 210nm | Area % UVMax | Zielmasse gefunden [M+H]+ Pos | [M-H]- Neg |
|---|---|---|---|---|---|---|
| 1 | Y | 1,55 | 96,6 | 99,5 | | 504,1439 |
| 2 | Y | 2,04 | 3,4 | n.a. | | |

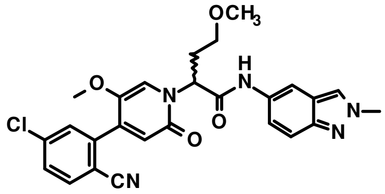

**48**

MS-MW [g/mol]: 505.1517

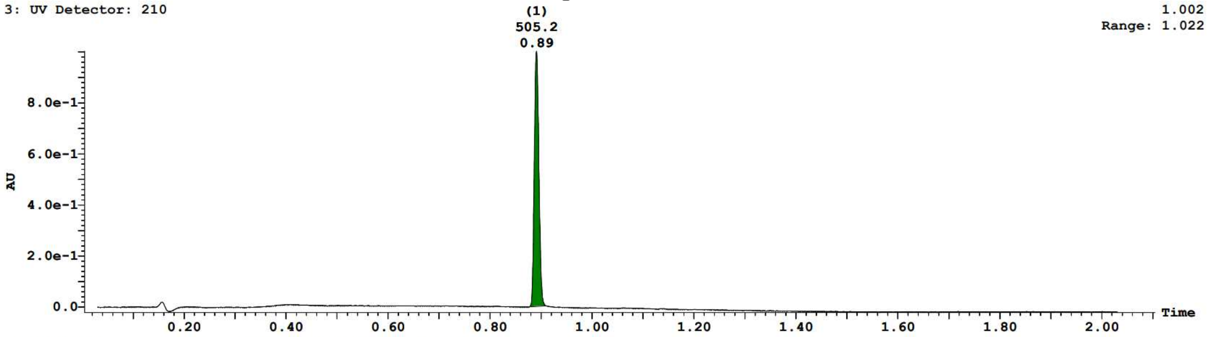


| Peak Number | Time | AreaAbs | Area %Total | Mass Found |
|---|---|---|---|---|
| 1 | 0.89 | 10460 | 100.00 | 505.15 |

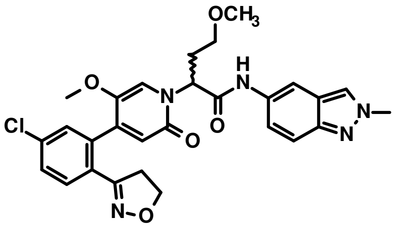


**49**

MS-MW [g/mol]: 549.1779

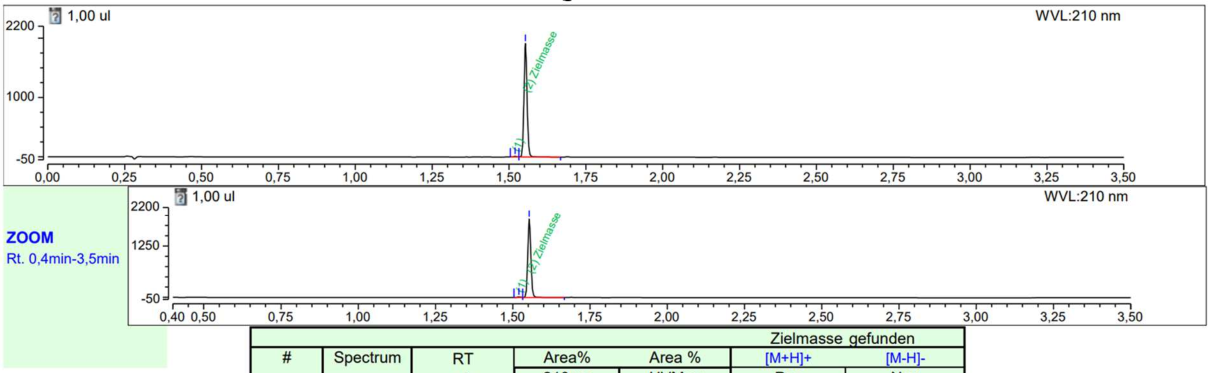


| # | Spectrum | RT min | Area% 210nm | Area % UVMax | Zielmasse gefunden [M+H]+ Pos | Zielmasse gefunden [M-H]- Neg |
|---|---|---|---|---|---|---|
| 1 | Y | 1,52 | 0,6 | 0,6 | | |
| 2 | Y | 1,55 | 99,4 | 99,4 | 550,1852 | 548,1706 |

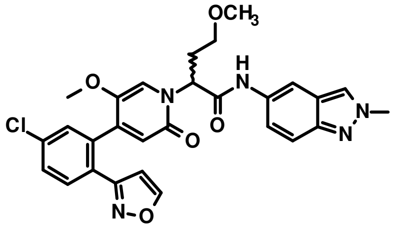


**50**

MS-MW [g/mol]: 547.1622

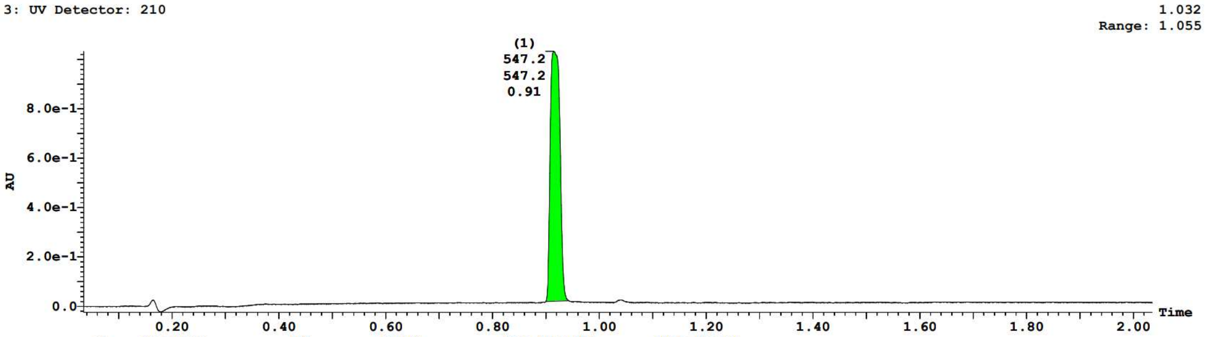


| # | Spectrum | RT | Area% | Pos Neg UV | Mass Found |
|---|---|---|---|---|---|
| 1 | --- | 0.91 | 100.00 | 548, 546, 247 | 547, 547 |

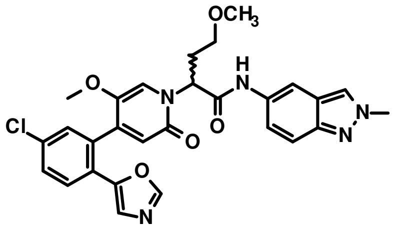


**51**

MS-MW [g/mol]: 547.1622

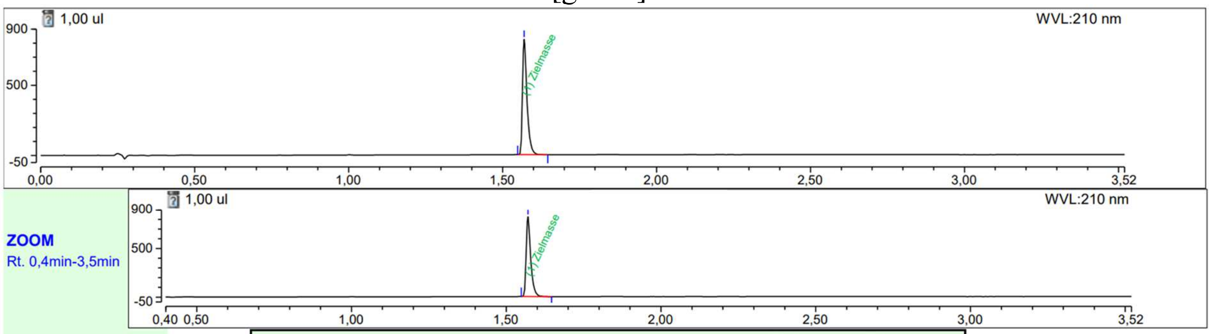


| # | Spectrum | RT | Area% | Area % | Zielmasse gefunden | |
|---|---|---|---|---|---|---|
| | | | | | [M+H]+ | [M-H]- |
| | | min | 210nm | UVMax | Pos | Neg |
| 1 | Y | 1,57 | 100,0 | 100,0 | 548,1695 | 546,1549 |

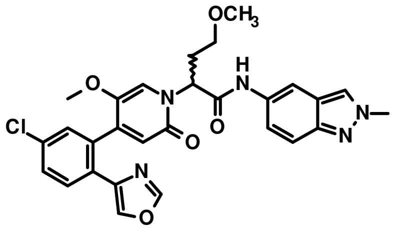


**52**

MS-MW [g/mol]: 547.1622

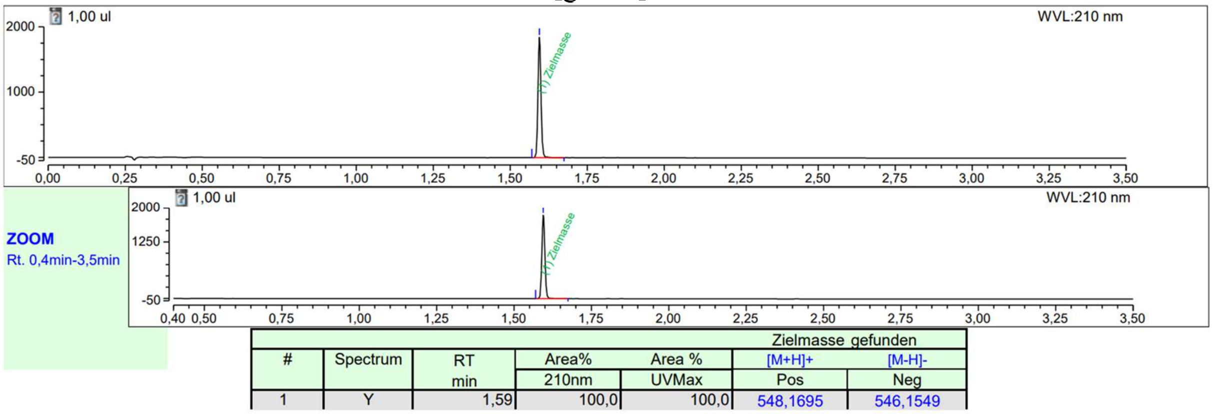


| # | Spectrum | RT | Area% | Area % | Zielmasse gefunden | |
|---|---|---|---|---|---|---|
| | | | | | [M+H]+ | [M-H]- |
| | | min | 210nm | UVMax | Pos | Neg |
| 1 | Y | 1,59 | 100,0 | 100,0 | 548,1695 | 546,1549 |

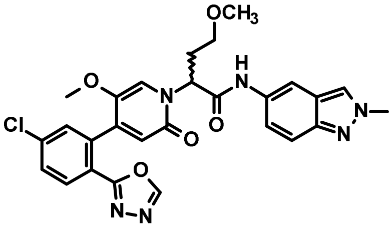


**53**

MS-MW [g/mol]: 548.1575

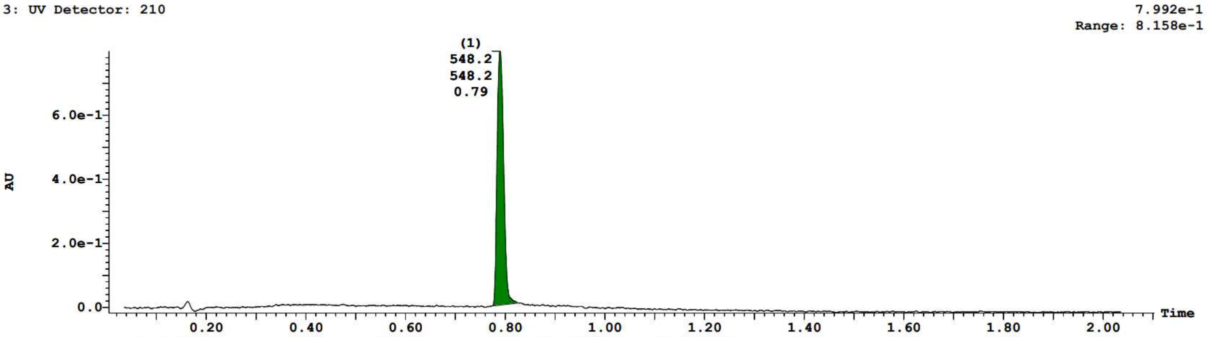


| # | Spectrum | RT | Area% | Pos Neg UV | Mass Found |
|---|---|---|---|---|---|
| 1 | --- | 0.79 | 100.00 | 549, 547, 239 | 548, 548 |

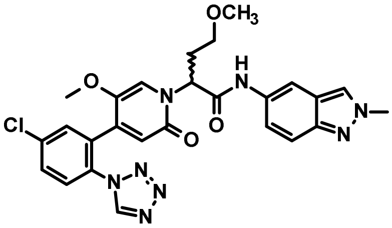


**54**

MS-MW [g/mol]: 548.1687

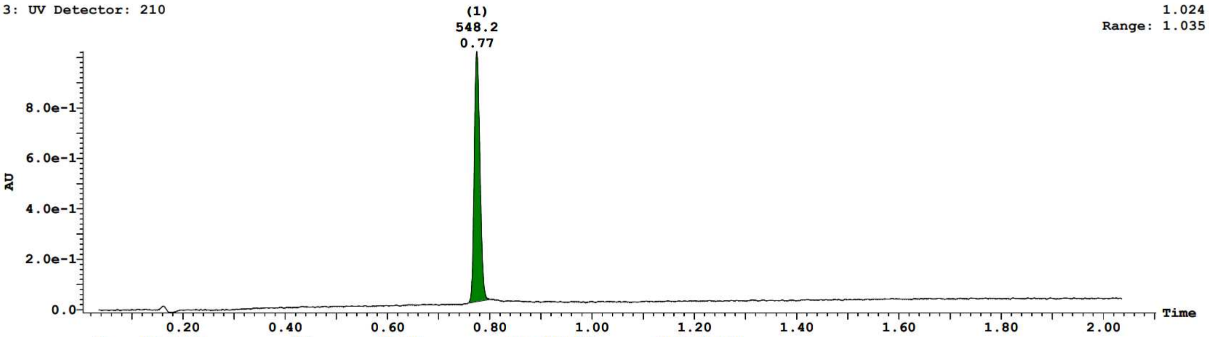


| # | Spectrum | RT | Area% | Pos Neg UV | Mass Found |
|---|---|---|---|---|---|
| 1 | --- | 0.77 | 100.00 | 402, 113, 240 | 548 |

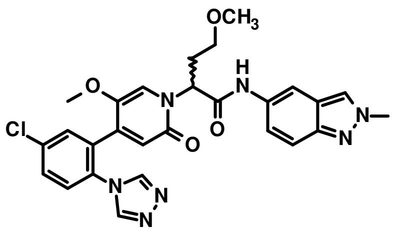

**55**

MS-MW [g/mol]: 547.1735

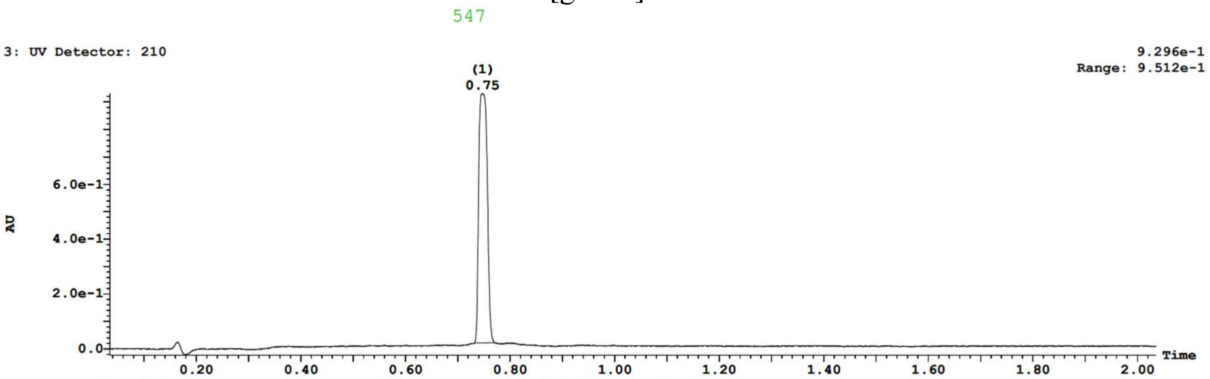


| # | Spectrum | RT | Area% | Pos Neg UV | Mass Found |
|---|---|---|---|---|---|
| 1 | --- | 0.75 | 100.00 | 275, 546, 249 | |

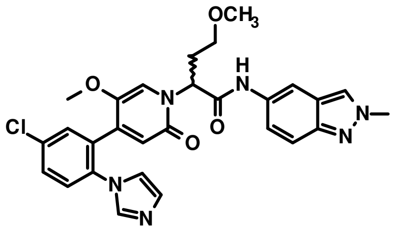

**56**

MS-MW [g/mol]: 546.1782

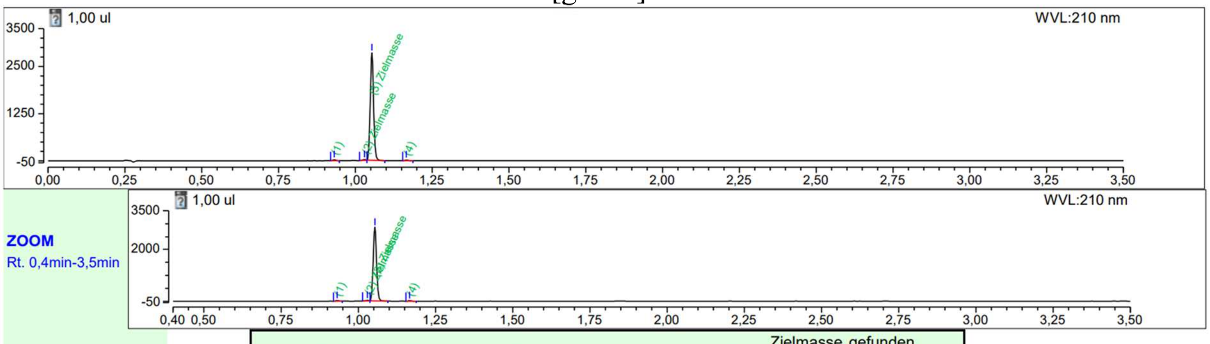


| # | Spectrum | RT min | Area% 210nm | Area % UVMax | Zielmasse gefunden [M+H]+ Pos | [M-H]- Neg |
|---|---|---|---|---|---|---|
| 1 | Y | 0,93 | 0,8 | 0,7 | | |
| 2 | Y | 1,03 | 0,6 | 0,6 | | 545,1709 |
| 3 | Y | 1,05 | 98,0 | 98,2 | 547,1855 | 545,1709 |
| 4 | Y | 1,17 | 0,6 | 0,5 | | |

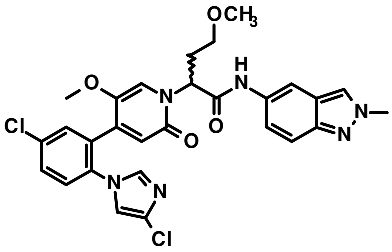


**57**

MS-MW [g/mol]: 580.1393

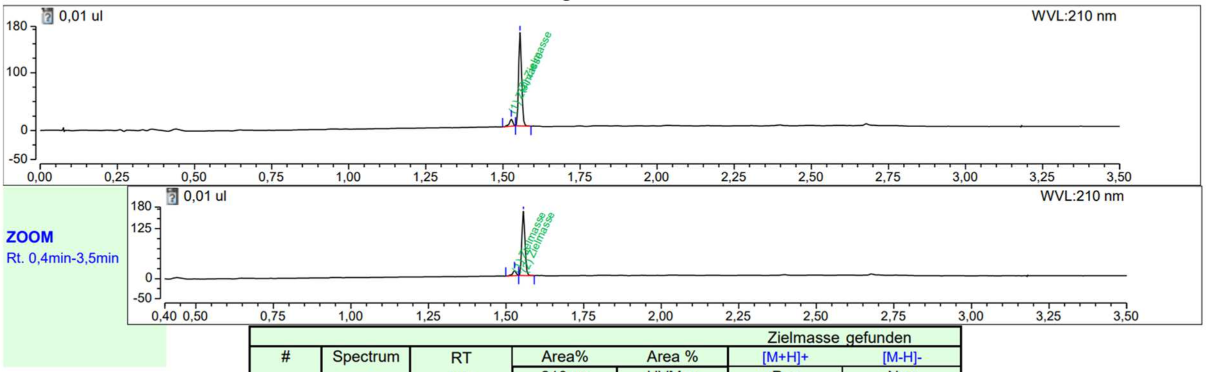


| # | Spectrum | RT min | Area% 210nm | Area % UVMax | Zielmasse gefunden [M+H]+ Pos | [M-H]- Neg |
|---|---|---|---|---|---|---|
| 1 | Y | 1,53 | 8,1 | 8,0 | 581,1466 | |
| 2 | Y | 1,56 | 91,9 | 92,0 | 581,1466 | 579,1315 |

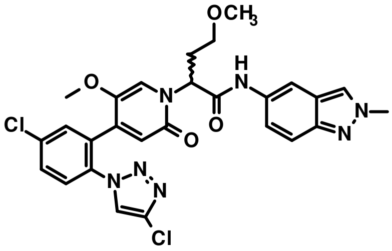


**58**

MS-MW [g/mol]: 581.1345

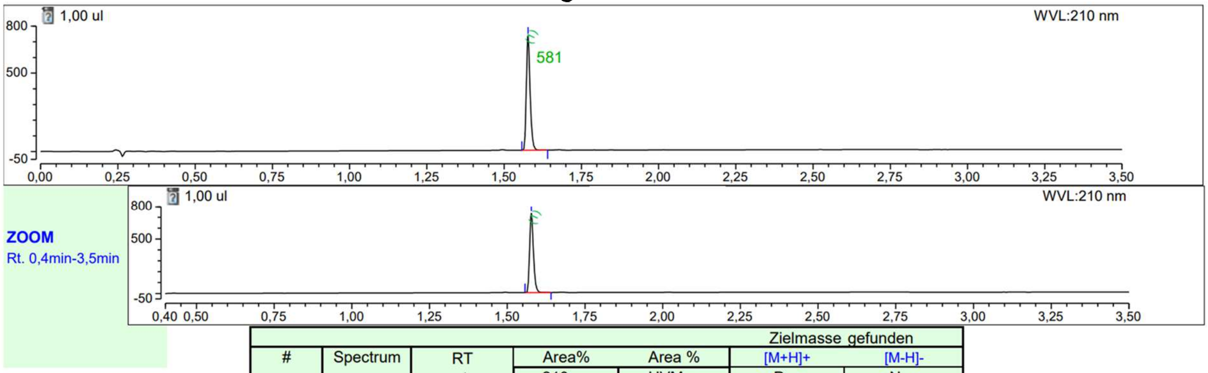


| # | Spectrum | RT min | Area% 210nm | Area % UVMax | Zielmasse gefunden [M+H]+ Pos | [M-H]- Neg |
|---|---|---|---|---|---|---|
| 1 | Y | 1,58 | 100,0 | 100,0 | | |

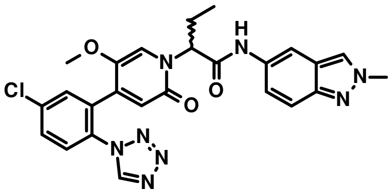

**59**

MS-MW [g/mol]: 518.1582

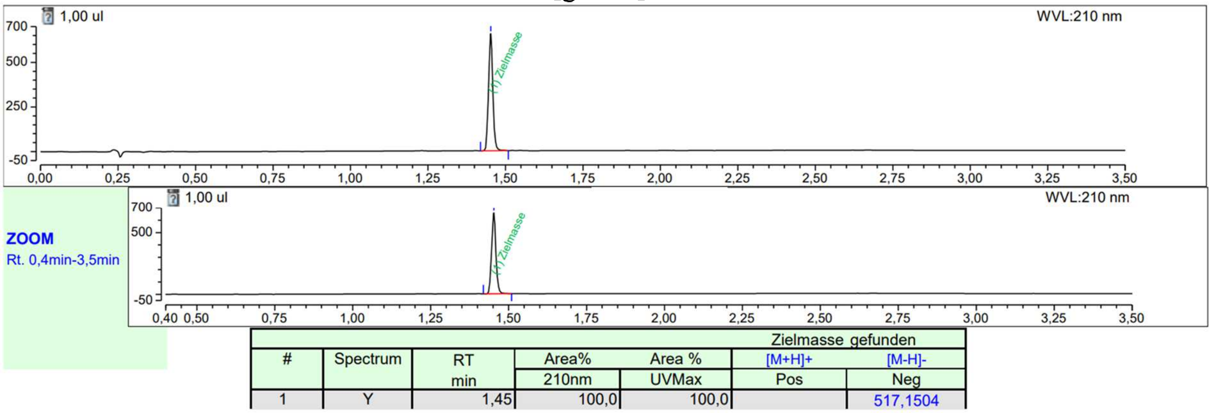


| # | Spectrum | RT min | Area% 210nm | Area % UVMax | Zielmasse gefunden [M+H]+ Pos | [M-H]- Neg |
|---|---|---|---|---|---|---|
| 1 | Y | 1,45 | 100,0 | 100,0 | | 517,1504 |

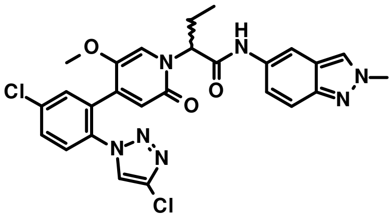

**60**

MS-MW [g/mol]: 551.1239

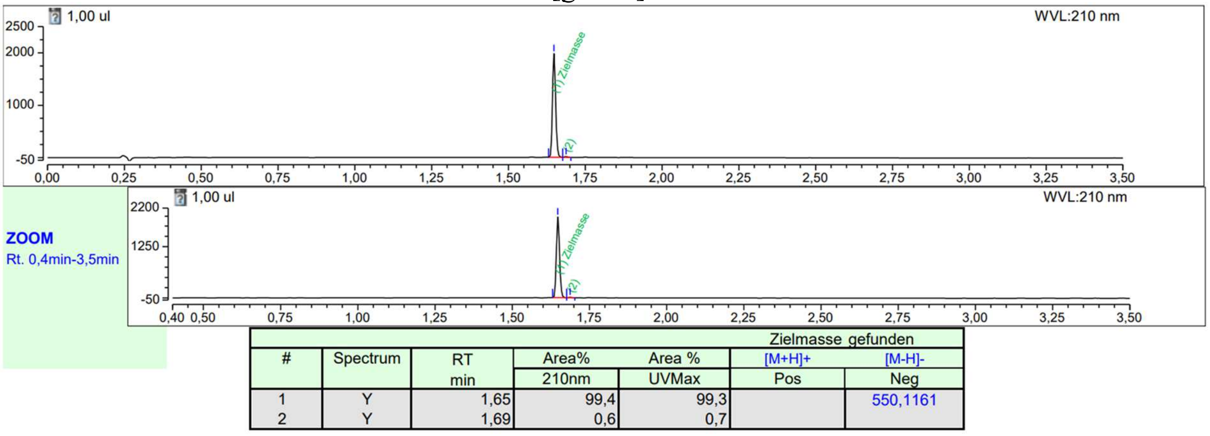


| # | Spectrum | RT min | Area% 210nm | Area % UVMax | Zielmasse gefunden [M+H]+ Pos | [M-H]- Neg |
|---|---|---|---|---|---|---|
| 1 | Y | 1,65 | 99,4 | 99,3 | | 550,1161 |
| 2 | Y | 1,69 | 0,6 | 0,7 | | |

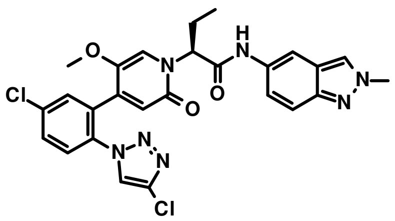

**61**

MS-MW [g/mol]: 551.1239

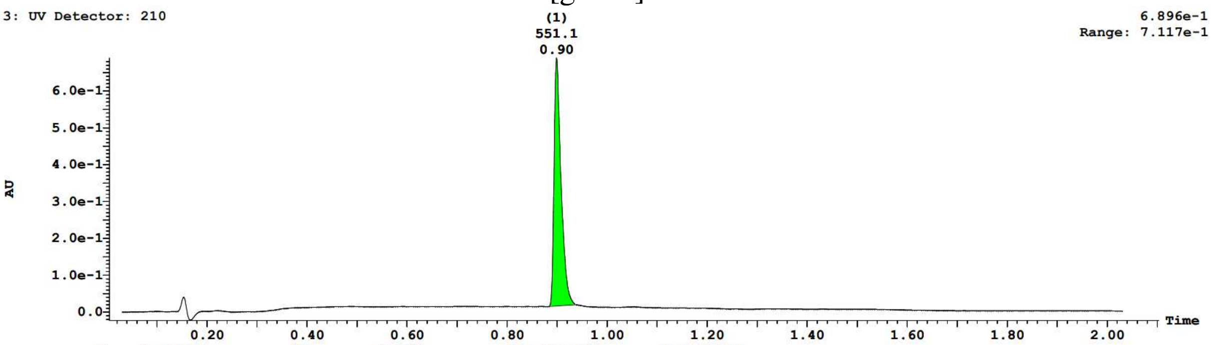


| # | Spectrum | RT | Area% | Pos Neg UV | Mass Found |
|---|---|---|---|---|---|
| 1 | --- | 0.90 | 100.00 | 405, 550, 227 | 551 |

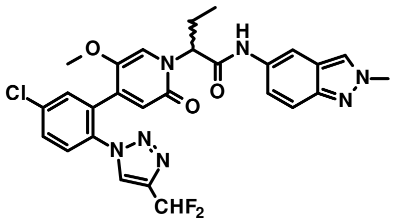

**62**

MS-MW [g/mol]: 567.1597

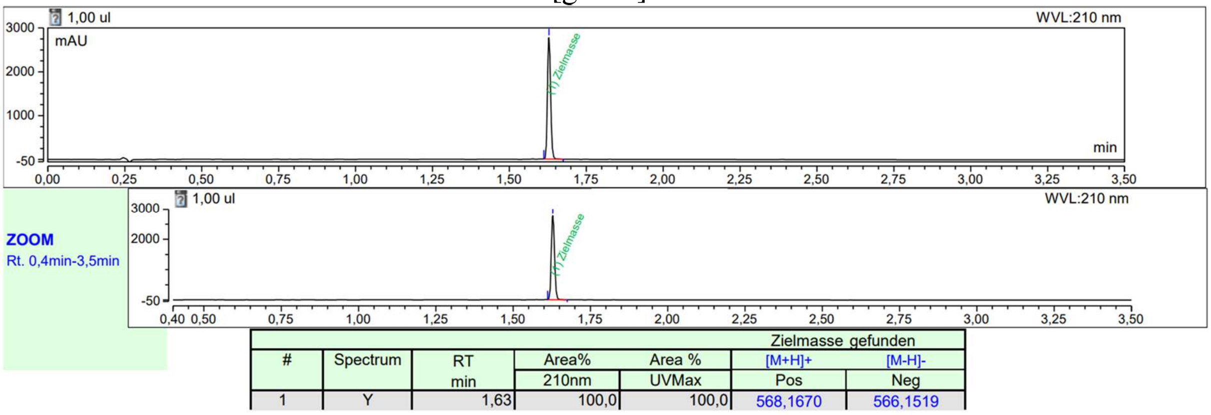


| # | Spectrum | RT min | Area% 210nm | Area % UVMax | Zielmasse gefunden [M+H]+ Pos | [M-H]- Neg |
|---|---|---|---|---|---|---|
| 1 | Y | 1,63 | 100,0 | 100,0 | 568,1670 | 566,1519 |

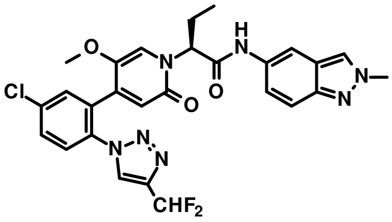


**63**

MS-MW [g/mol]: 567.1597

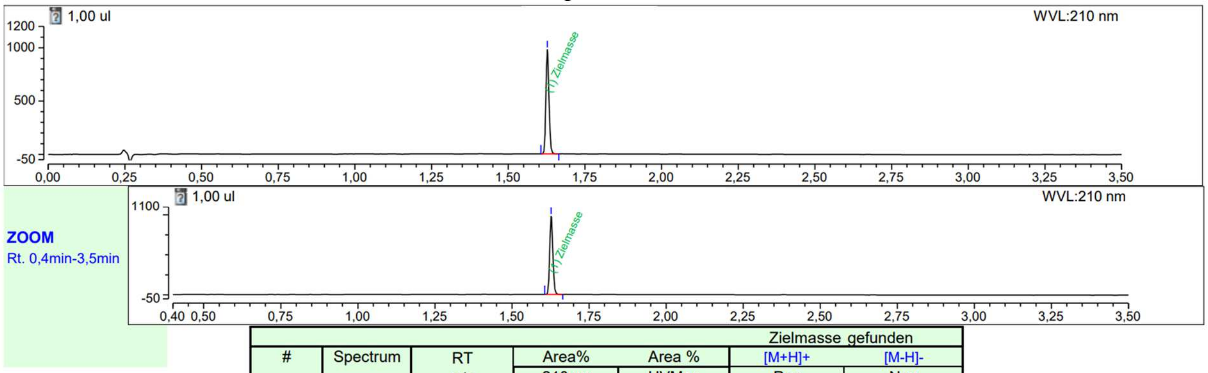


| # | Spectrum | RT | Area% | Area % | Zielmasse gefunden | |
|---|---|---|---|---|---|---|
| | | | | | [M+H]+ | [M-H]- |
| | | min | 210nm | UVMax | Pos | Neg |
| 1 | Y | 1,63 | 100,0 | 100,0 | | 566,1519 |

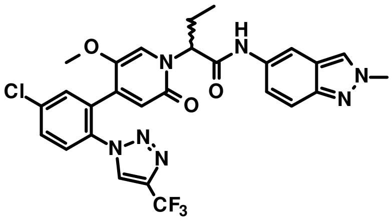


**64**

MS-MW [g/mol]: 585.1503

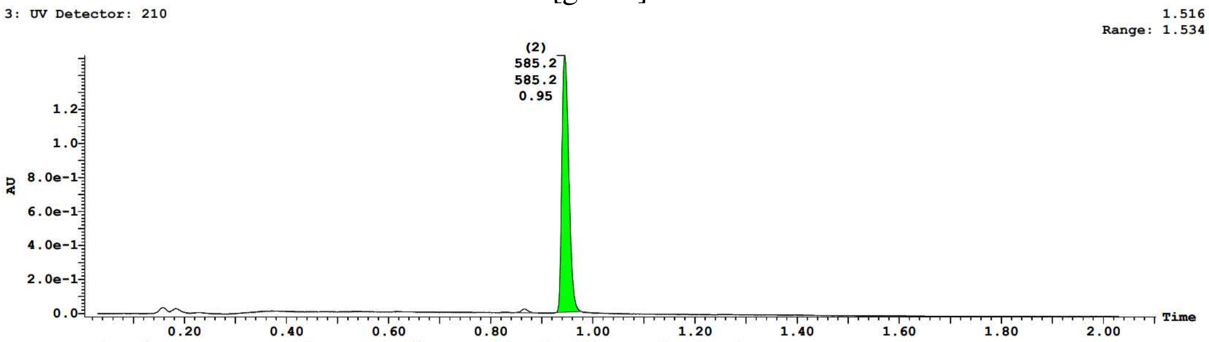


| # | Spectrum | RT | Area% | Pos Neg UV | Mass Found |
|---|---|---|---|---|---|
| 1 | --- | 0.87 | 1.41 | 695, 693, 223 | |
| 2 | --- | 0.94 | 98.59 | 439, 584, 234 | 585, 585 |

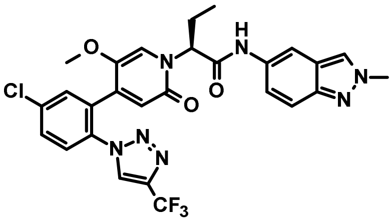


**65**

MS-MW [g/mol]: 585.1503

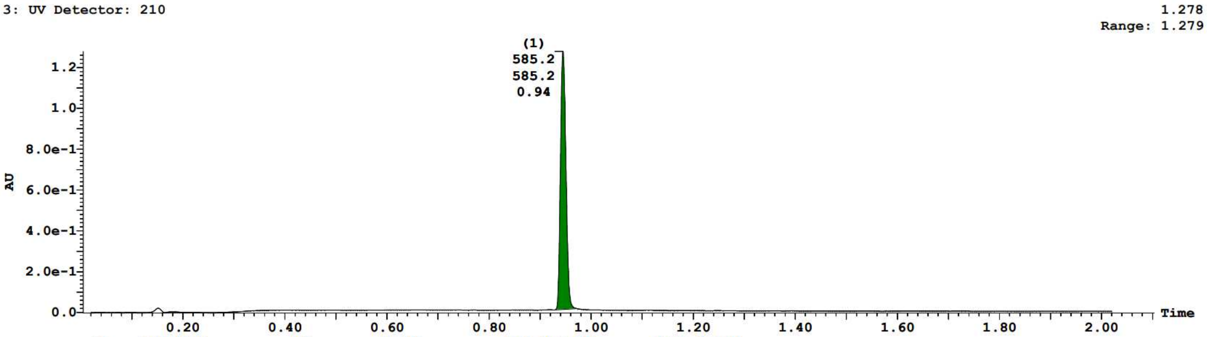


| # | Spectrum | RT | Area% | Pos Neg UV | Mass Found |
|---|---|---|---|---|---|
| 1 | --- | 0.94 | 100.00 | 586, 584, 233 | 585, 585 |

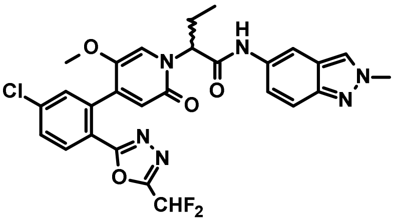


**66**

MS-MW [g/mol]: 568.1437

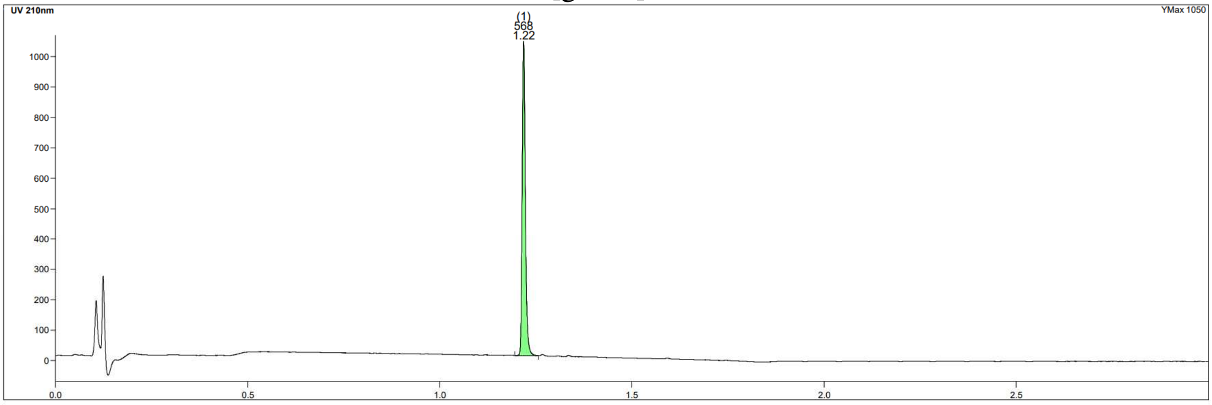


| # | Spectrum | RT | Area% | | Base Mass | |
|---|---|---|---|---|---|---|
| | | min | 210nm | UVMax | Pos | Neg |
| 1 | Y | 1.22 | 100 | 100 | 422 | 567 |

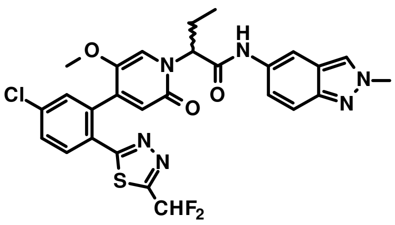


**67**

MS-MW [g/mol]: 584.1209

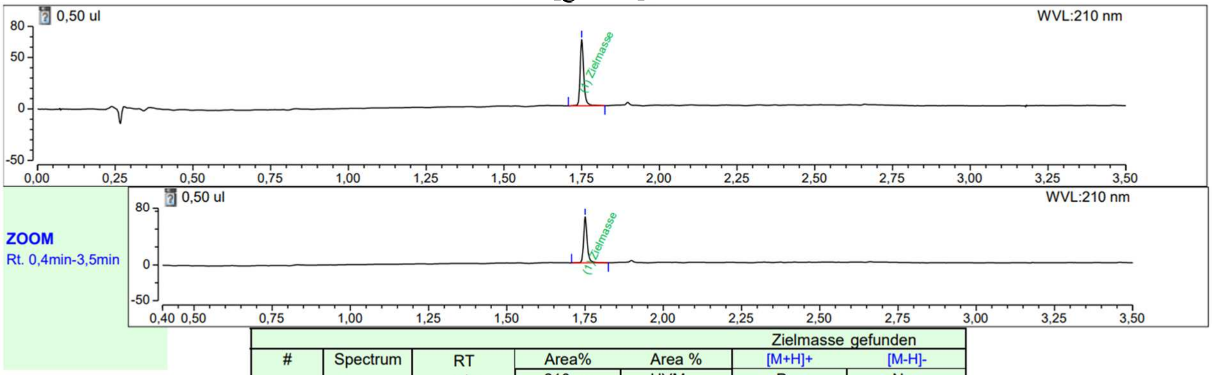


| # | Spectrum | RT | Area% | Area % | Zielmasse gefunden | |
|---|---|---|---|---|---|---|
| | | | | | [M+H]+ | [M-H]- |
| | | min | 210nm | UVMax | Pos | Neg |
| 1 | Y | 1,75 | 100,0 | 100,0 | 585,1282 | 583,1131 |

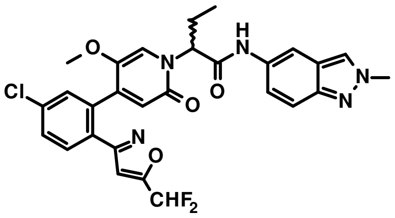


**68**

MS-MW [g/mol]: 567.1485

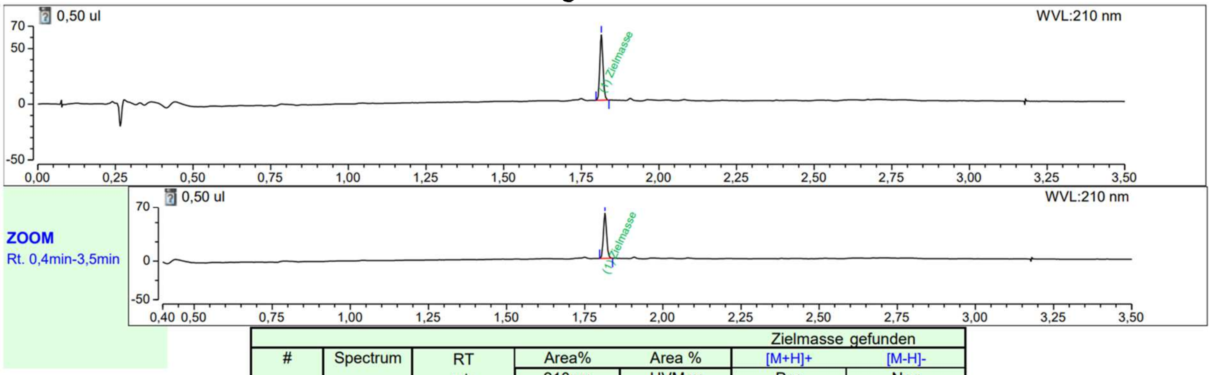


| # | Spectrum | RT | Area% | Area % | Zielmasse gefunden | |
|---|---|---|---|---|---|---|
| | | | | | [M+H]+ | [M-H]- |
| | | min | 210nm | UVMax | Pos | Neg |
| 1 | Y | 1,81 | 100,0 | 100,0 | 568,1558 | 566,1407 |

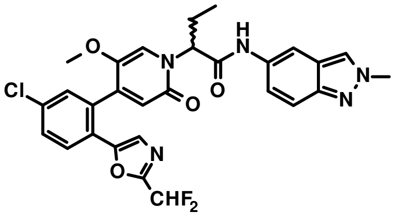

**69**

MS-MW [g/mol]: 567.1485

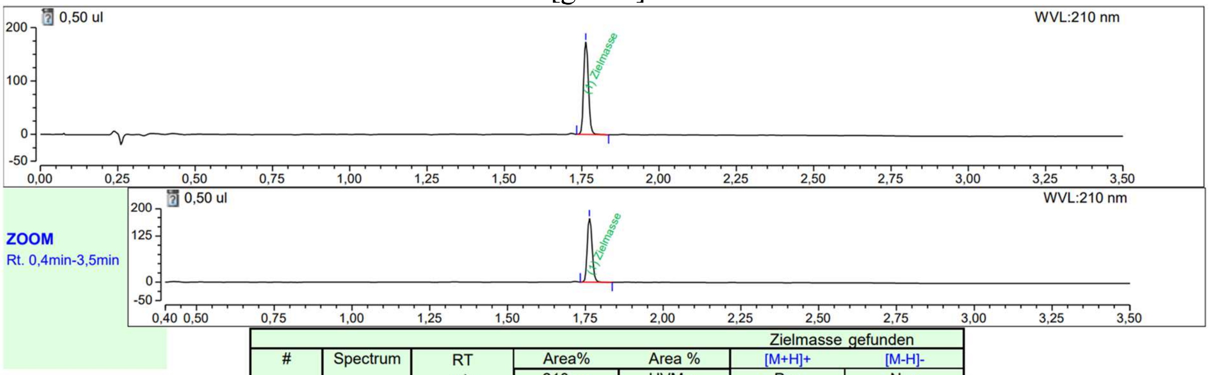


| # | Spectrum | RT | Area% | Area % | Zielmasse gefunden | |
|---|---|---|---|---|---|---|
| | | | | | [M+H]+ | [M-H]- |
| | | min | 210nm | UVMax | Pos | Neg |
| 1 | Y | 1,76 | 100,0 | 100,0 | 568,1558 | 566,1407 |

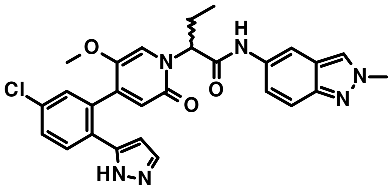

**70**

MS-MW [g/mol]: 516.1677

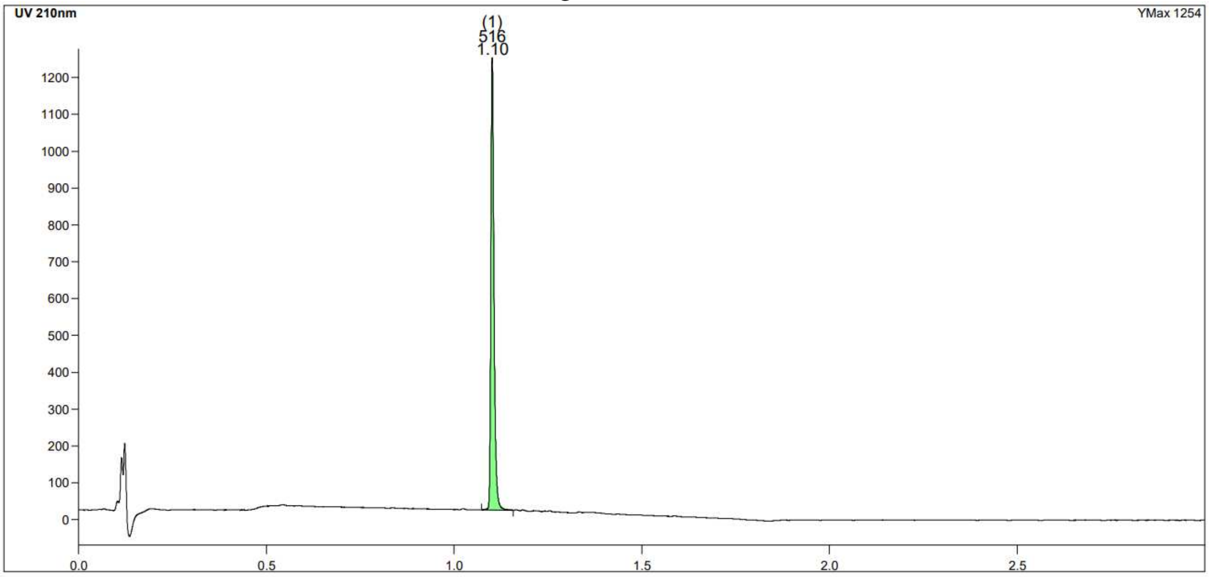


| # | Spectrum | RT | Area% | | Base Mass | |
|---|---|---|---|---|---|---|
| | | min | 210nm | UVMax | Pos | Neg |
| 1 | Y | 1.10 | 100 | 100 | 370 | 515 |

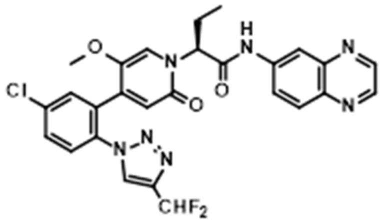

**71**

MS-MW [g/mol]: 565.1441

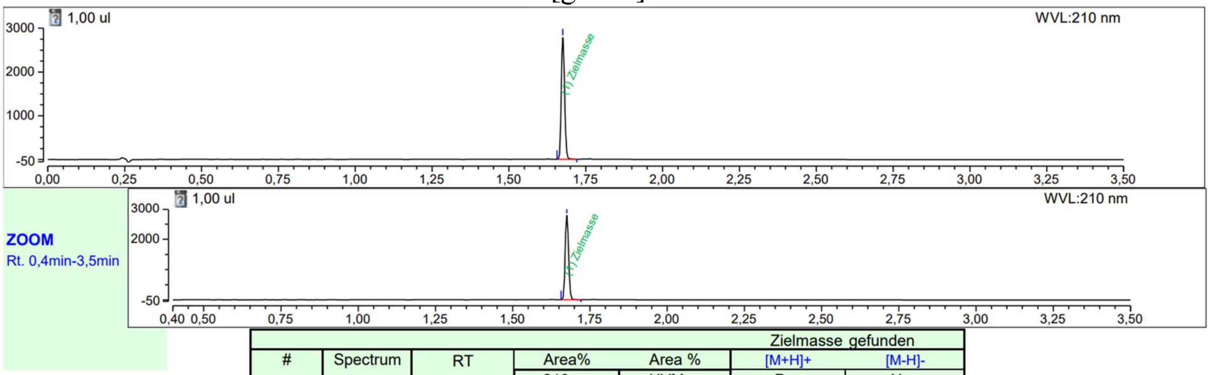


| # | Spectrum | RT min | Area% 210nm | Area % UVMax | Zielmasse gefunden [M+H]+ Pos | [M-H]- Neg |
|---|---|---|---|---|---|---|
| 1 | Y | 1,68 | 100,0 | 100,0 | 566,1514 | 564,1363 |

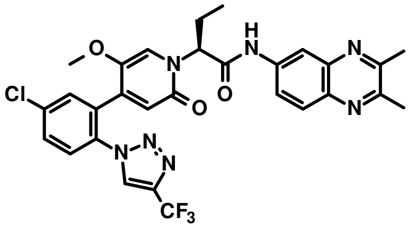

**72**

MS-MW [g/mol]: 611.1660

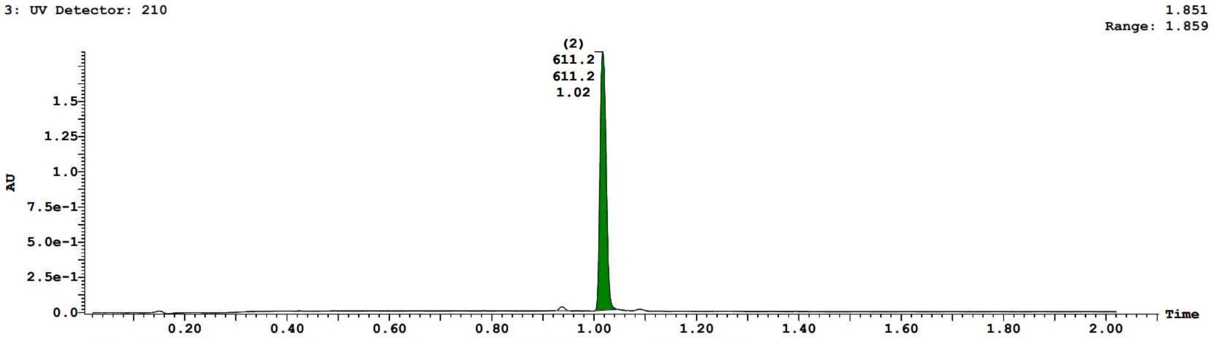


| # | Spectrum | RT | Area% | Pos Neg UV | Mass Found |
|---|---|---|---|---|---|
| 1 | --- | 0.94 | 1.38 | 101, 113, 217 | |
| 2 | --- | 1.02 | 98.62 | 612, 610, 253 | 611, 611 |

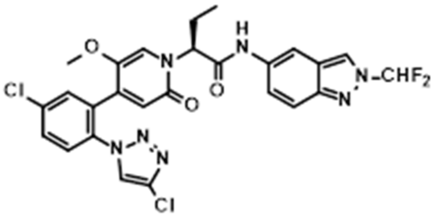


**73**

MS-MW [g/mol]: 587.1051

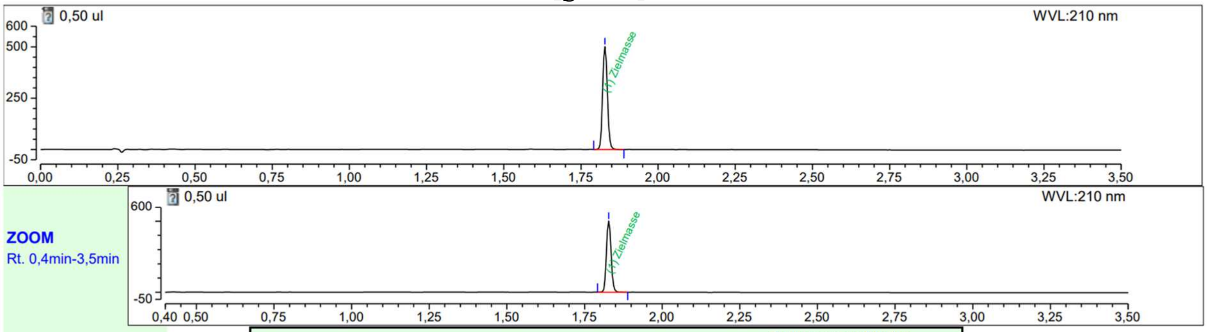


| # | Spectrum | RT min | Area% 210nm | Area % UVMax | Zielmasse gefunden [M+H]+ Pos | [M-H]- Neg |
|---|---|---|---|---|---|---|
| 1 | Y | 1,83 | 100,0 | 100,0 | | 586,0973 |

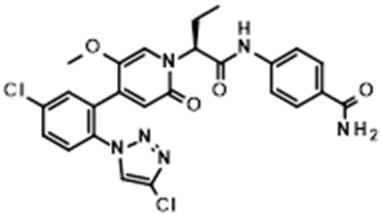


**74**

MS-MW [g/mol]: 540.1080

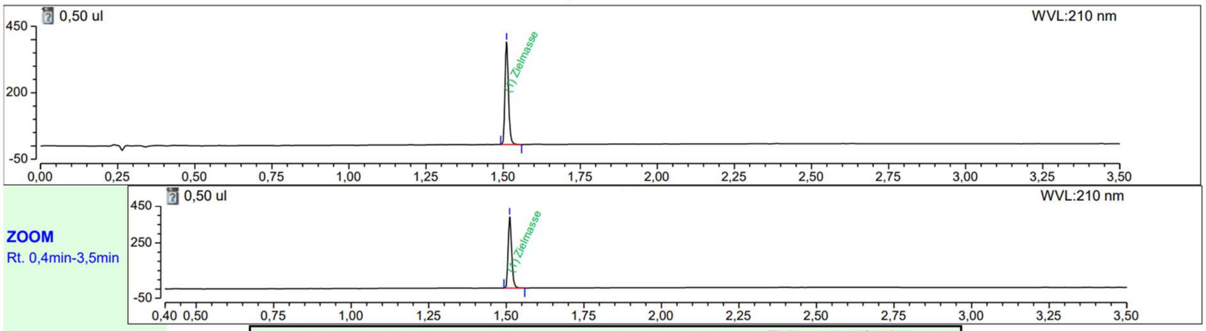


| # | Spectrum | RT min | Area% 210nm | Area % UVMax | Zielmasse gefunden [M+H]+ Pos | [M-H]- Neg |
|---|---|---|---|---|---|---|
| 1 | Y | 1,51 | 100,0 | 100,0 | 541,1153 | 539,1002 |

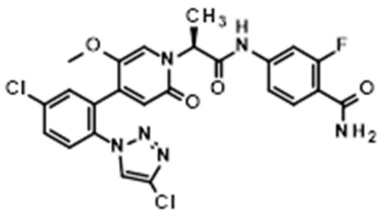

**75**

MS-MW [g/mol]: 544.0829

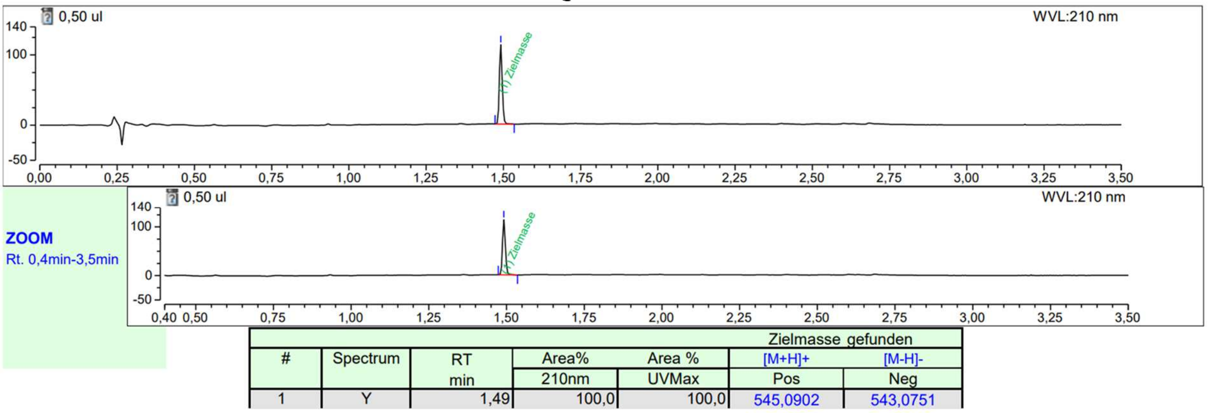


| # | Spectrum | RT min | Area% 210nm | Area % UVMax | Zielmasse gefunden [M+H]+ Pos | [M-H]- Neg |
|---|---|---|---|---|---|---|
| 1 | Y | 1,49 | 100,0 | 100,0 | 545,0902 | 543,0751 |

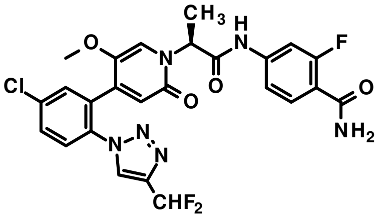

**76**

MS-MW [g/mol]: 560.1187

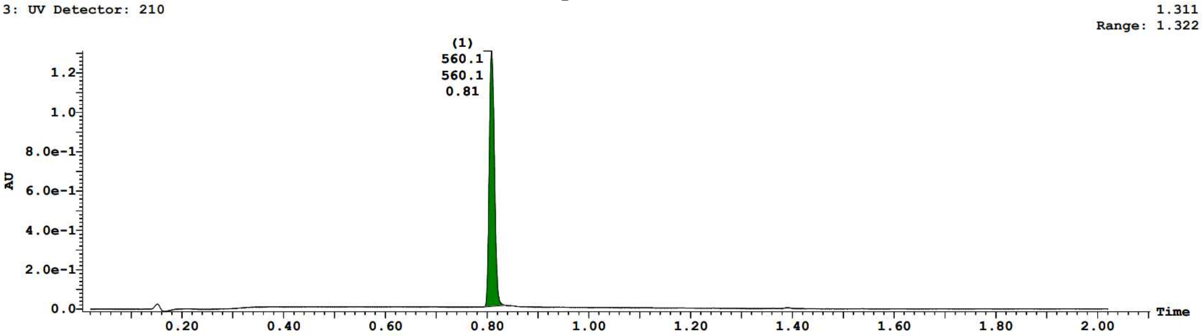


| # | Spectrum | RT | Area% | Pos Neg UV | Mass Found |
|---|---|---|---|---|---|
| 1 | --- | 0.81 | 100.00 | 561, 559, 208 | 560, 560 |

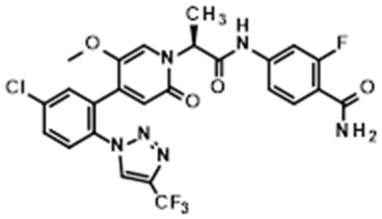

**77**

MS-MW [g/mol]: 578.1092

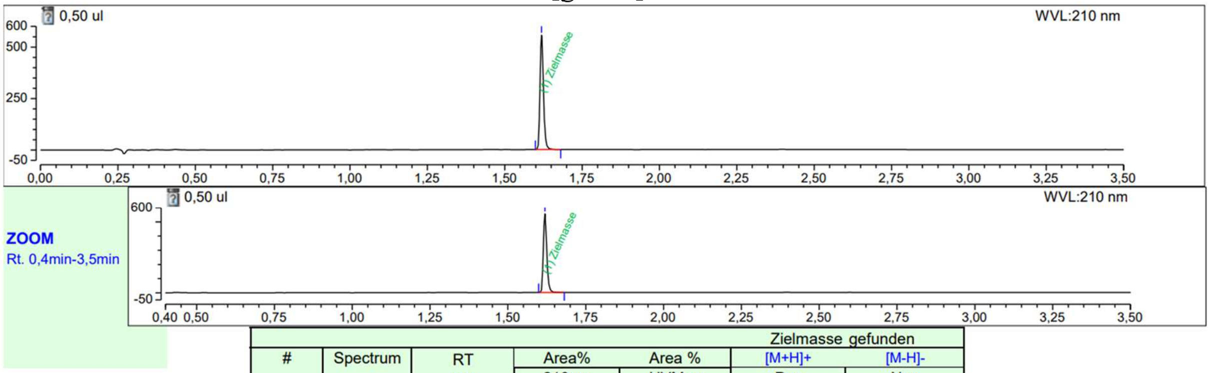


| | | | | | Zielmasse gefunden | |
|---|---|---|---|---|---|---|
| # | Spectrum | RT min | Area% 210nm | Area % UVMax | [M+H]+ Pos | [M-H]- Neg |
| 1 | Y | 1,62 | 100,0 | 100,0 | 579,1165 | |

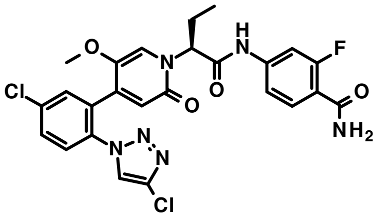

**78**

MS-MW [g/mol]: 558.0985

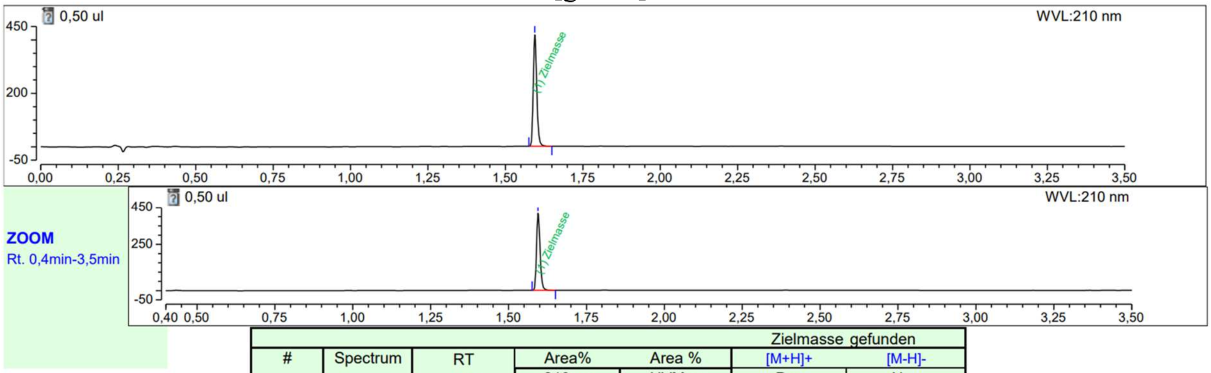


| | | | | | Zielmasse gefunden | |
|---|---|---|---|---|---|---|
| # | Spectrum | RT min | Area% 210nm | Area % UVMax | [M+H]+ Pos | [M-H]- Neg |
| 1 | Y | 1,59 | 100,0 | 100,0 | 559,1058 | |

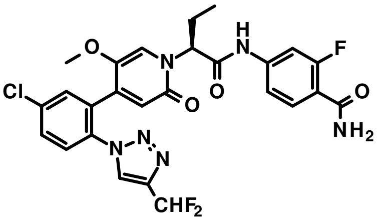


**79**

MS-MW [g/mol]: 574.1343

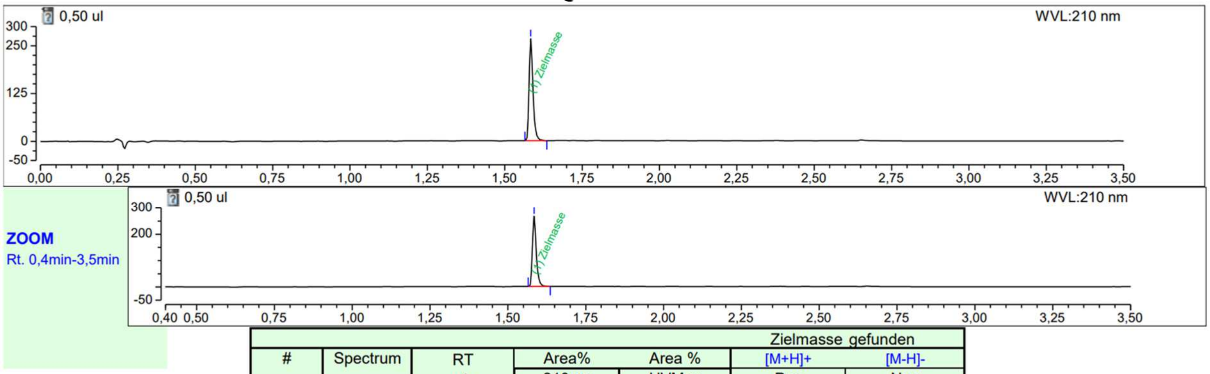


| # | Spectrum | RT min | Area% 210nm | Area % UVMax | Zielmasse gefunden [M+H]+ Pos | Zielmasse gefunden [M-H]- Neg |
|---|---|---|---|---|---|---|
| 1 | Y | 1,58 | 100,0 | 100,0 | 575,1416 | 573,1265 |

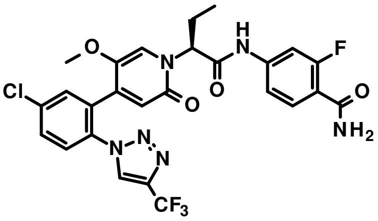


**80**

MS-MW [g/mol]: 592.1249

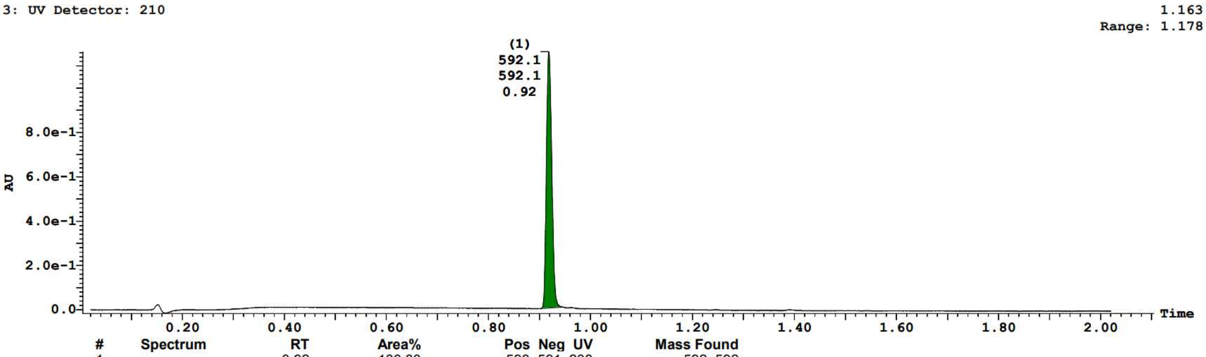


| # | Spectrum | RT | Area% | Pos Neg UV | Mass Found |
|---|---|---|---|---|---|
| 1 | --- | 0.92 | 100.00 | 593, 591, 208 | 592, 592 |

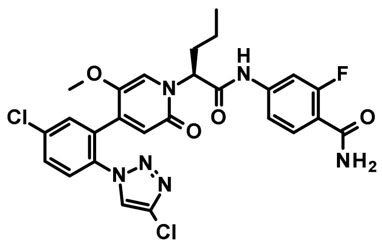


**81**

MS-MW [g/mol]: 572.1142

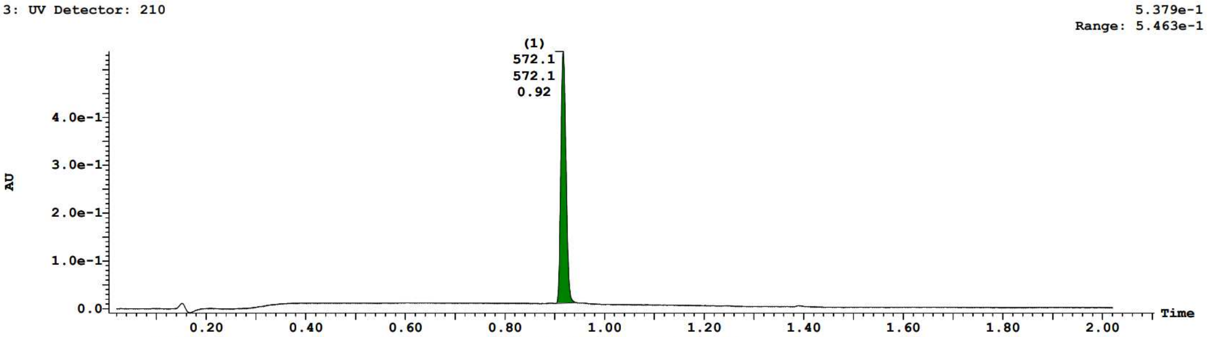


| # | Spectrum | RT | Area% | Pos Neg UV | Mass Found |
|---|---|---|---|---|---|
| 1 | --- | 0.92 | 100.00 | 573, 571, 210 | 572, 572 |

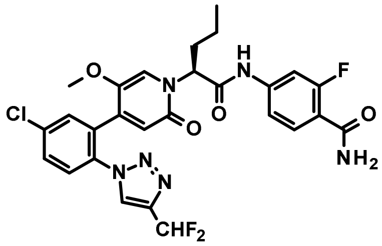


**82**

MS-MW [g/mol]: 588.1500

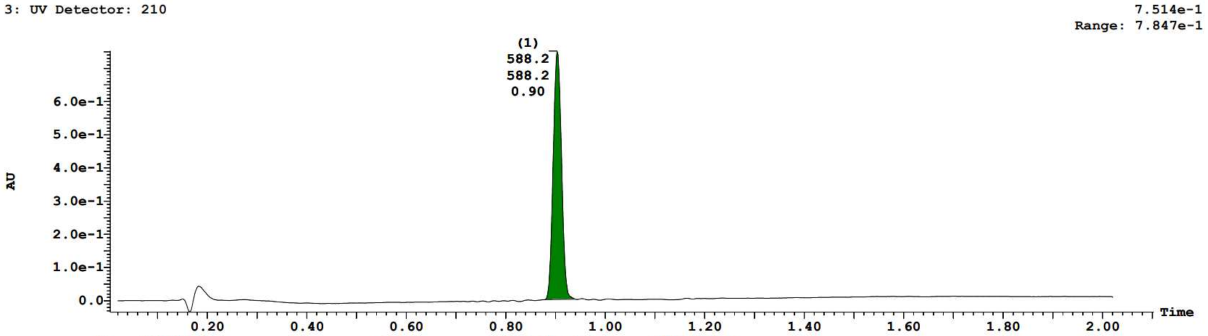


| # | Spectrum | RT | Area% | Pos Neg UV | Mass Found |
|---|---|---|---|---|---|
| 1 | --- | 0.90 | 100.00 | 589, 587, 210 | 588, 588 |

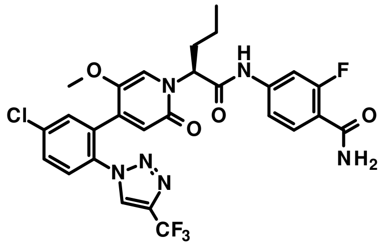

**83**

MS-MW [g/mol]: 606.1405

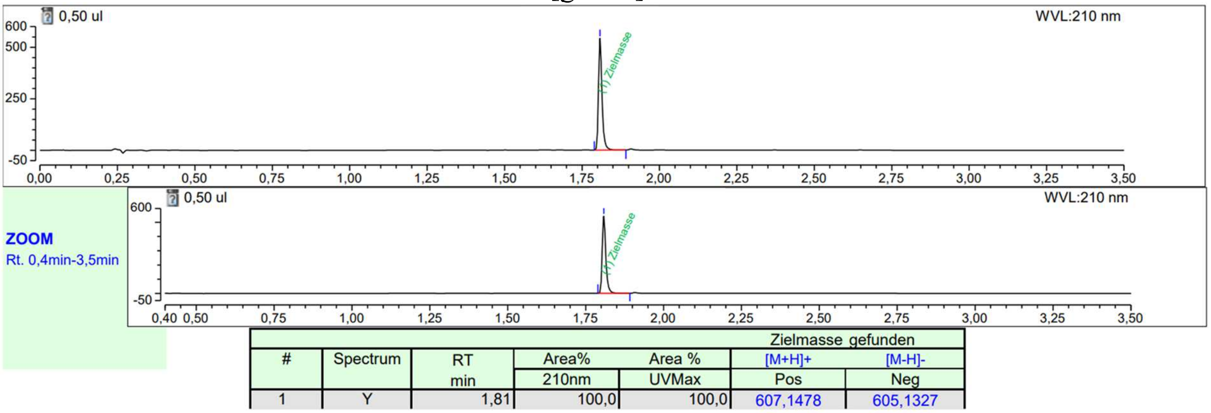


| # | Spectrum | RT min | Area% 210nm | Area % UVMax | Zielmasse gefunden [M+H]+ Pos | Zielmasse gefunden [M-H]- Neg |
|---|---|---|---|---|---|---|
| 1 | Y | 1,81 | 100,0 | 100,0 | 607,1478 | 605,1327 |

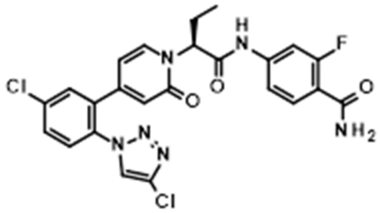

**84**

MS-MW [g/mol]: 528.0880

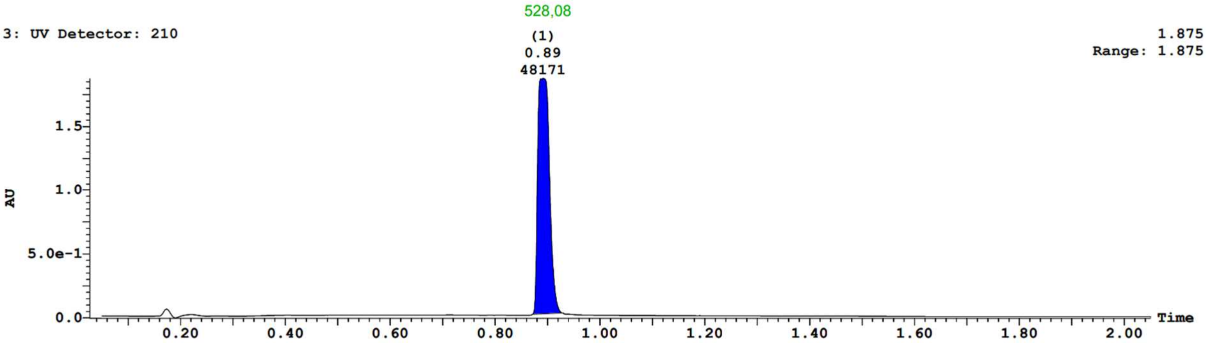


| # | Spectrum | RT | Area% | Pos | Neg | UV | Mass Found |
|---|---|---|---|---|---|---|---|
| 1 | --- | 0.89 | 100.00 | | | 375, 252 | |

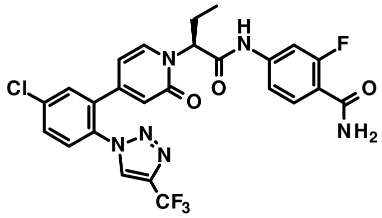


**85**

MS-MW [g/mol]: 562.1143

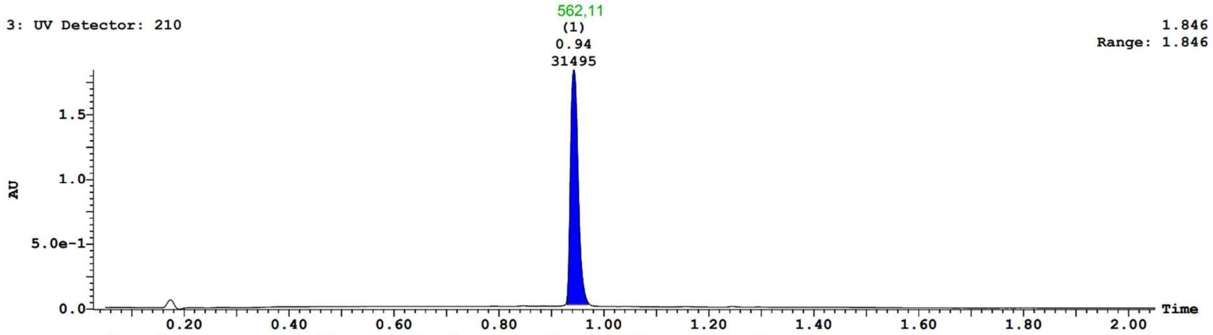


| # | Spectrum | RT | Area% | Pos | Neg | UV | Mass Found |
|---|---|---|---|---|---|---|---|
| 1 | --- | 0.94 | 100.00 | | | 409, 222 | |

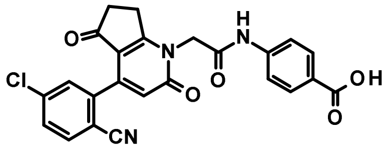

**355**

MS-MW [g/mol]: 461.0778

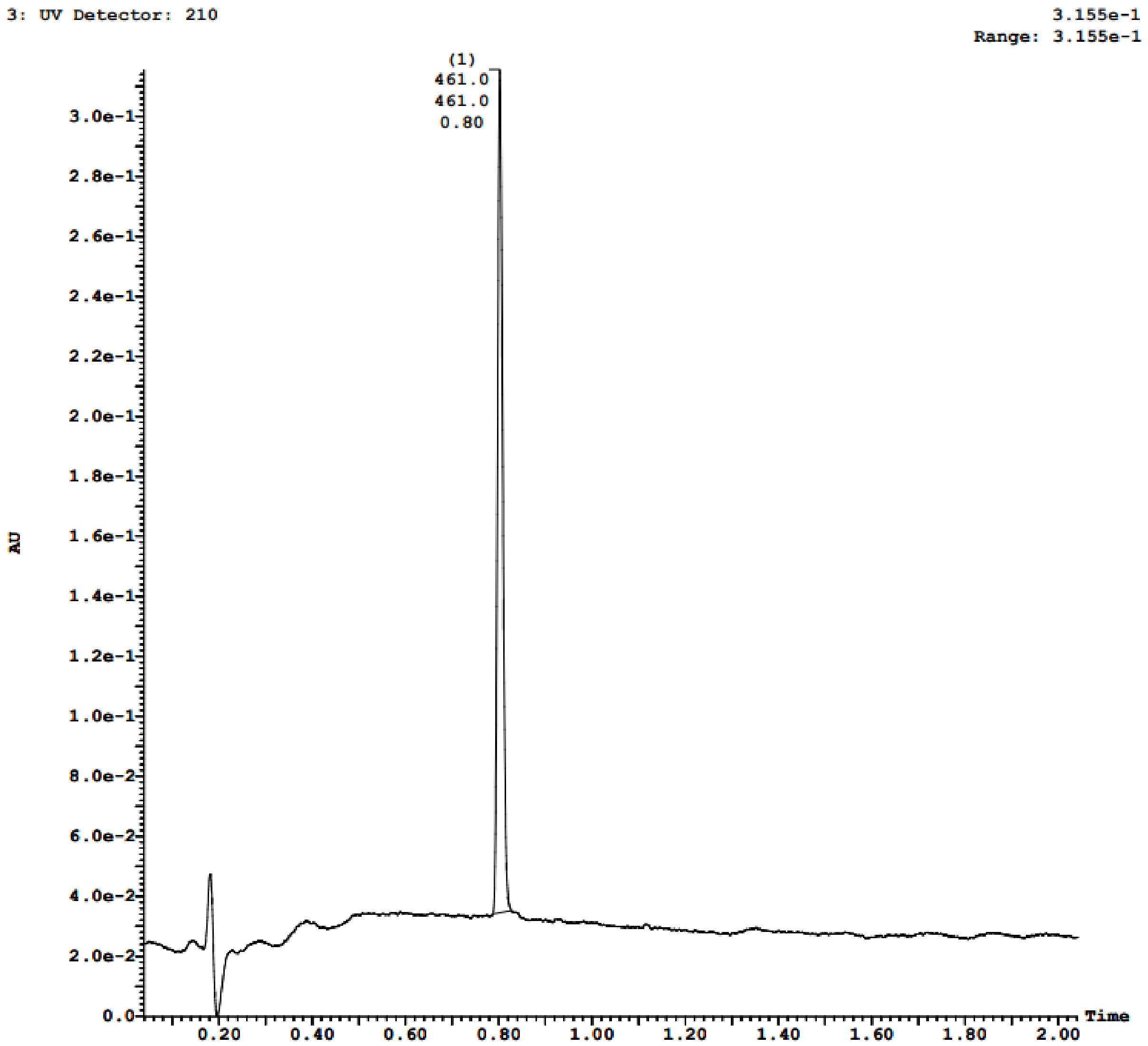


| Peak Number | Time | AreaAbs | Area %Total | Mass Found |
|---|---|---|---|---|
| 1 | 0.80 | 3631 | 100.00 | 461.00, 461.00 |

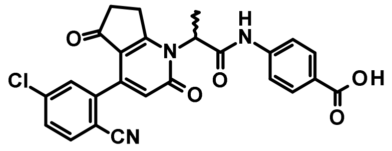

**362**

MS-MW [g/mol]: 475.0935

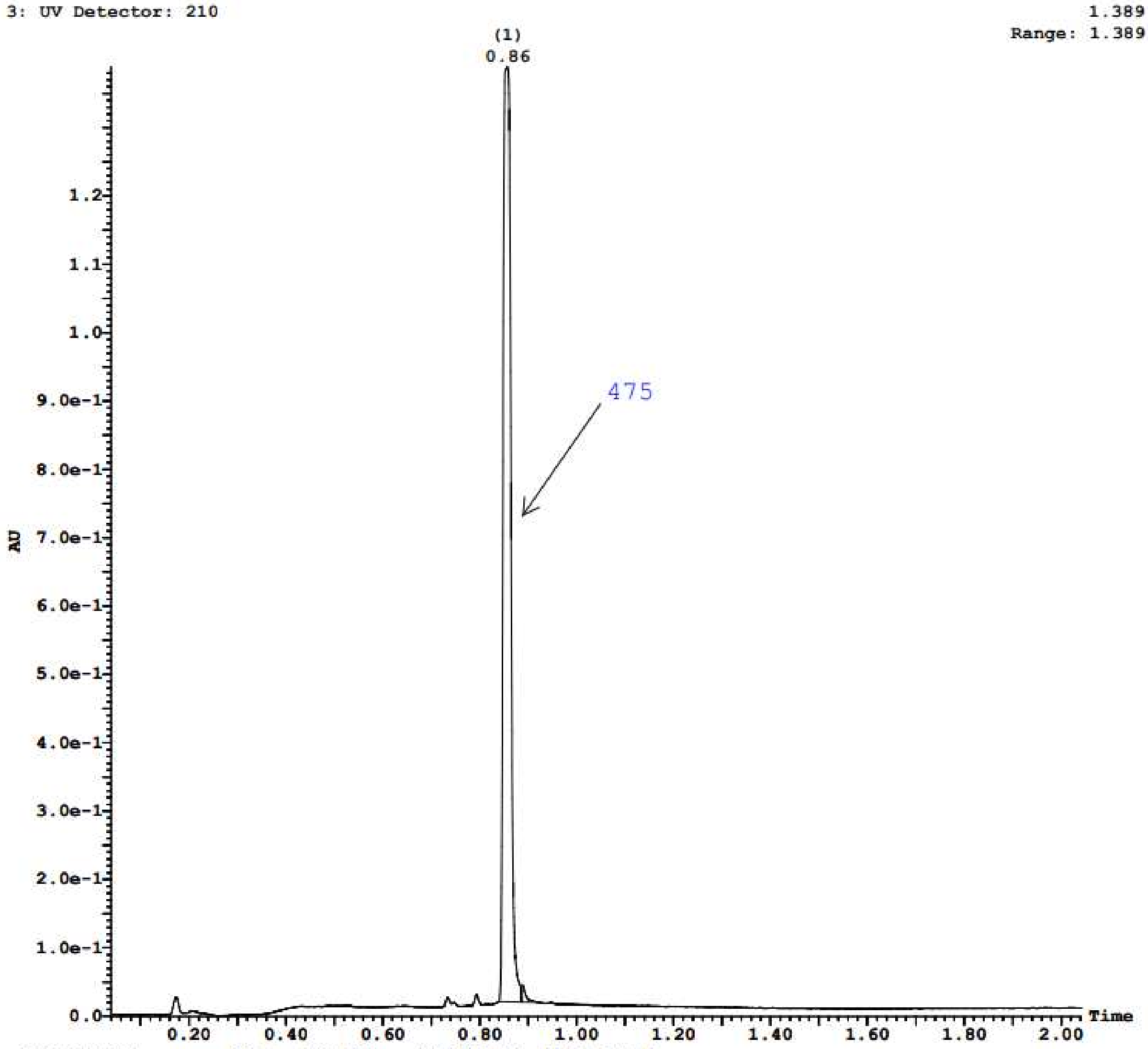


| Peak Number | Time | AreaAbs | Area %Total | Mass Found |
|---|---|---|---|---|
| 1 | 0.86 | 25037 | 98.98 | |
| 2 | 0.89 | 258 | 1.02 | |

## (E) Molecular Formula String Spreadsheet of Compounds 1-85, 355, and 262.

| Compd | SMILES | $IC_{50}$ FXIa [M] |
|---|---|---|
| **1** | NCC1CCC(CC1)C(=O)N[C@@H](Cc2ccccc2)C(=O)Nc3ccc(cc3)c4nnn[nH]4 | 8.8 E-9 |
| **2** | Nc1n[nH]c2cc(ccc12)c3nc(CNC(=O)\C=C\c4cc(Cl)ccc4n5cnnn5)[nH]c3Cl | 4.2 E-9 |
| **3** | Clc1cccc(c1)C2=CC(=O)N(CC(=O)Nc3ccc(cc3)c4nnn[nH]4)C5=C2C(=O)NC5 | 9.3 E-7 |
| **4** | Clc1cccc(c1)C2=CC(=O)N(CC(=O)Nc3ccc(cc3)c4nnn[nH]4)C=C2 | >5.0 E-5 |
| **5** | Clc1ccc(Cl)c(c1)C2=CC(=O)N(CC(=O)Nc3ccc(cc3)c4nnn[nH]4)C=C2 | 1.9 E-6 |
| **6** | Clc1ccc(Br)c(c1)C2=CC(=O)N(CC(=O)Nc3ccc(cc3)c4nnn[nH]4)C=C2 | 2.5 E-6 |
| **7** | Clc1ccc(C#N)c(c1)C2=CC(=O)N(CC(=O)Nc3ccc(cc3)c4nnn[nH]4)C=C2 | 5.6 E-7 |
| **8** | CCNCC.Clc1ccc(C#N)c(c1)C2=CC(=O)N(C=C2)C(Cc3ccccc3)C(=O)Nc4ccc(cc4)c5nnn[nH]5 | 7.6 E-9 |
| **9** | CC(N1C=CC(=CC1=O)c2cc(Cl)ccc2C#N)C(=O)Nc3ccc(cc3)c4nnn[nH]4 | 5.3 E-8 |
| **10** | CCC(N1C=CC(=CC1=O)c2cc(Cl)ccc2C#N)C(=O)Nc3ccc(cc3)c4nnn[nH]4 | 5.9 E-8 |
| **11** | Clc1ccc(C#N)c(c1)C2=CC(=O)N(C=C2)C(CC3CC3)C(=O)Nc4ccc(cc4)c5nnn[nH]5 | 3.9 E-8 |
| **12** | CCCCC(N1C=CC(=CC1=O)c2cc(Cl)ccc2C#N)C(=O)Nc3ccc(cc3)c4nnn[nH]4 | 1.5 E-7 |
| **13** | CC(N1C=CC(=CC1=O)c2cc(Cl)ccc2C#N)C(=O)Nc3ccc(cc3)C(=O)O | 1.7 E-7 |
| **14** | CC(N1C=C(F)C(=CC1=O)c2cc(Cl)ccc2C#N)C(=O)Nc3ccc(cc3)C(=O)O | 7.4 E-8 |
| **15** | CC(N1C=C(Cl)C(=CC1=O)c2cc(Cl)ccc2C#N)C(=O)Nc3ccc(cc3)C(=O)O | 2.7 E-8 |
| **16** | CC(N1C=C(C#N)C(=CC1=O)c2cc(Cl)ccc2C#N)C(=O)Nc3ccc(cc3)C(=O)O | 4.2 E-8 |
| **17** | CC(N1C=C(C#N)C(=CC1=O)c2cc(Cl)ccc2C(F)F)C(=O)Nc3ccc(cc3)C(=O)O | 4.3 E-8 |
| **18** | CC(N1C=C(C#N)C(=CC1=O)c2cc(Cl)ccc2C(F)(F)F)C(=O)Nc3ccc(cc3)C(=O)O | 4.7 E-7 |
| **19** | COC1=CN(C(C)C(=O)Nc2ccc(cc2)C(=O)O)C(=O)C=C1c3cc(Cl)ccc3C#N | 5.9 E-9 |
| **20** | COC1=CN(C(C)C(=O)Nc2ccc(cc2)C(=O)O)C(=O)C=C1c3cc(Cl)ccc3OC(F)F | 1.0 E-8 |
| **21** | COC1=CN(C(C)C(=O)Nc2ccc(cc2)C(=O)O)C(=O)C=C1c3cc(Cl)ccc3OC(F)(F)F | 4.6 E-8 |
| **22** | CC(N1C=C(C(F)F)C(=CC1=O)c2cc(Cl)ccc2C#N)C(=O)Nc3ccc(cc3)C(=O)O | 6.3 E-7 |
| **23** | CC(N1C=C(C(=CC1=O)c2cc(Cl)ccc2C#N)C(F)(F)F)C(=O)Nc3ccc(cc3)C(=O)O | 3.5 E-6 |
| **24** | COC1=CN([C@@H](C)C(=O)Nc2ccc(cc2)C(=O)O)C(=O)C=C1c3cc(Cl)ccc3C#N | 2.0 E-9 |
| **25** | CC[C@H](N1C=C(OC)C(=CC1=O)c2cc(Cl)ccc2C#N)C(=O)Nc3ccc(cc3)C(=O)O | 1.8 E-9 |
| **26** | COC1=CN(C(C(C)C)C(=O)Nc2ccc(cc2)C(=O)O)C(=O)C=C1c3cc(Cl)ccc3C#N | 1.2 E-7 |

| | | |
|---|---|---|
| **27** | COC1=CN([C@@H](CC(C)C)C(=O)Nc2ccc(cc2)C(=O)O)C(=O)C=C1c3cc(Cl)ccc3C#N | 4.7 E-9 |
| **28** | COC1=CN([C@@H](CC2CC2)C(=O)Nc3ccc(cc3)C(=O)O)C(=O)C=C1c4cc(Cl)ccc4C#N | 1.6 E-9 |
| **29** | COC1=CN([C@@H](CC2CCC2)C(=O)Nc3ccc(cc3)C(=O)O)C(=O)C=C1c4cc(Cl)ccc4C#N | 1.0 E-9 |
| **30** | COC1=CN(C(CC(C)(C)C)C(=O)Nc2ccc(cc2)C(=O)O)C(=O)C=C1c3cc(Cl)ccc3C#N | 3.2 E-8 |
| **31** | COCC[C@H](N1C=C(OC)C(=CC1=O)c2cc(Cl)ccc2C#N)C(=O)Nc3ccc(cc3)C(=O)O | 1.3 E-9 |
| **32** | COC1=CN([C@@H](CCOC(F)(F)F)C(=O)Nc2ccc(cc2)C(=O)O)C(=O)C=C1c3cc(Cl)ccc3C#N | 1.0 E-9 |
| **33** | COC1=CN([C@@H](C[C@@H]2CCCO2)C(=O)Nc3ccc(cc3)C(=O)O)C(=O)C=C1c4cc(Cl)ccc4C#N | 1.3 E-9 |
| **34** | COC1=CN([C@@H](C[C@@H]2CCCCO2)C(=O)Nc3ccc(cc3)C(=O)O)C(=O)C=C1c4cc(Cl)ccc4C#N | 5.0 E-10 |
| **35** | COC1=CN([C@@H](C[C@H]2CCCCO2)C(=O)Nc3ccc(cc3)C(=O)O)C(=O)C=C1c4cc(Cl)ccc4C#N | 1.4 E-9 |
| **36** | CO[C@@H](C)C[C@H](N1C=C(OC)C(=CC1=O)c2cc(Cl)ccc2C#N)C(=O)Nc3ccc(cc3)C(=O)O | 7.0 E-10 |
| **37** | COC1=CN([C@@H](CC2CCC(O)CC2)C(=O)Nc3ccc(cc3)C(=O)O)C(=O)C=C1c4cc(Cl)ccc4C#N | 3.0 E-10 |
| **38** | COC1=CN(C(C[C@@H]2CCCCO2)C(=O)NC34CCC(CC3)(CC4)C(=O)O)C(=O)C=C1c5cc(Cl)ccc5C#N | 1.5 E-7 |
| **39** | COC1=CN(C(C[C@@H]2CCCCO2)C(=O)NC34CC(C3)(C4)C(=O)O)C(=O)C=C1c5cc(Cl)ccc5C#N | 6.7 E-8 |
| **40** | COCCC(N1C=C(OC)C(=CC1=O)c2cc(Cl)ccc2C#N)C(=O)NC3CC4(C3)CC(C4)C(=O)O | 1.2 E-7 |
| **41** | COCCC(N1C=C(OC)C(=CC1=O)c2cc(Cl)ccc2C#N)C(=O)NC3CCc4[nH]ncc4C3 | 5.0 E-6 |
| **42** | COCCC(N1C=C(OC)C(=CC1=O)c2cc(Cl)ccc2C#N)C(=O)Nc3ccc4[nH]ncc4c3 | 6.8 E-9 |
| **43** | COCCC(N1C=C(OC)C(=CC1=O)c2cc(Cl)ccc2C#N)C(=O)Nc3ccc4C(=O)NNc4c3 | 2.2 E-9 |
| **44** | COCC[C@H](N1C=C(OC)C(=CC1=O)c2cc(Cl)ccc2C#N)C(=O)Nc3ccc(C(=O)N)c(F)c3 | 3.8 E-9 |
| **45** | COCC[C@H](N1C=C(OC)C(=CC1=O)c2cc(Cl)ccc2C#N)C(=O)Nc3ccc(nc3)C(=O)N | 5.7 E-9 |
| **46** | COCC[C@H](N1C=C(OC)C(=CC1=O)c2cc(Cl)ccc2C#N)C(=O)Nc3ccc4nccnc4c3 | 2.4 E-9 |
| **47** | COCC[C@H](N1C=C(OC)C(=CC1=O)c2cc(Cl)ccc2C#N)C(=O)Nc3ccc4nn(C)cc4c3 | 6.4 E-9 |
| **48** | COCCC(N1C=C(OC)C(=CC1=O)c2cc(Cl)ccc2C#N)C(=O)Nc3ccc4nn(C)cc4c3 | 4.3 E-9 |
| **49** | COCCC(N1C=C(OC)C(=CC1=O)c2cc(Cl)ccc2C3=NOCC3)C(=O)Nc4ccc5nn(C)cc5c4 | 4.5 E-9 |
| **50** | COCCC(N1C=C(OC)C(=CC1=O)c2cc(Cl)ccc2c3ccon3)C(=O)Nc4ccc5nn(C)cc5c4 | 2.3 E-9 |
| **51** | COCCC(N1C=C(OC)C(=CC1=O)c2cc(Cl)ccc2c3ocnc3)C(=O)Nc4ccc5nn(C)cc5c4 | 2.9 E-9 |
| **52** | COCCC(N1C=C(OC)C(=CC1=O)c2cc(Cl)ccc2c3cocn3)C(=O)Nc4ccc5nn(C)cc5c4 | 3.9 E-8 |
| **53** | COCCC(N1C=C(OC)C(=CC1=O)c2cc(Cl)ccc2c3ocnn3)C(=O)Nc4ccc5nn(C)cc5c4 | 4.0 E-9 |
| **54** | COCCC(N1C=C(OC)C(=CC1=O)c2cc(Cl)ccc2n3cnnn3)C(=O)Nc4ccc5nn(C)cc5c4 | 2.6 E-10 |
| **55** | COCCC(N1C=C(OC)C(=CC1=O)c2cc(Cl)ccc2n3cnnc3)C(=O)Nc4ccc5nn(C)cc5c4 | 4.8 E-9 |

| | | |
|---|---|---|
| **56** | COCCC(N1C=C(OC)C(=CC1=O)c2cc(Cl)ccc2n3ccnc3)C(=O)Nc4ccc5nn(C)cc5c4 | 1.8 E-9 |
| **57** | COCCC(N1C=C(OC)C(=CC1=O)c2cc(Cl)ccc2n3cnc(Cl)c3)C(=O)Nc4ccc5nn(C)cc5c4 | 3.4 E-9 |
| **58** | COCCC(N1C=C(OC)C(=CC1=O)c2cc(Cl)ccc2n3cc(Cl)nn3)C(=O)Nc4ccc5nn(C)cc5c4 | 9.6 E-10 |
| **59** | CCC(N1C=C(OC)C(=CC1=O)c2cc(Cl)ccc2n3cnnn3)C(=O)Nc4ccc5nn(C)cc5c4 | 8.2 E-10 |
| **60** | CCC(N1C=C(OC)C(=CC1=O)c2cc(Cl)ccc2n3cc(Cl)nn3)C(=O)Nc4ccc5nn(C)cc5c4 | 1.4 E-9 |
| **61** | CC[C@H](N1C=C(OC)C(=CC1=O)c2cc(Cl)ccc2n3cc(Cl)nn3)C(=O)Nc4ccc5nn(C)cc5c4 | 7.8 E-10 |
| **62** | CCC(N1C=C(OC)C(=CC1=O)c2cc(Cl)ccc2n3cc(nn3)C(F)F)C(=O)Nc4ccc5nn(C)cc5c4 | 1.9 E-9 |
| **63** | CC[C@H](N1C=C(OC)C(=CC1=O)c2cc(Cl)ccc2n3cc(nn3)C(F)F)C(=O)Nc4ccc5nn(C)cc5c4 | 8.6 E-10 |
| **64** | CCC(N1C=C(OC)C(=CC1=O)c2cc(Cl)ccc2n3cc(nn3)C(F)(F)F)C(=O)Nc4ccc5nn(C)cc5c4 | 1.1 E-9 |
| **65** | CC[C@H](N1C=C(OC)C(=CC1=O)c2cc(Cl)ccc2n3cc(nn3)C(F)(F)F)C(=O)Nc4ccc5nn(C)cc5c4 | 7.9 E-10 |
| **66** | CCC(N1C=C(OC)C(=CC1=O)c2cc(Cl)ccc2c3oc(nn3)C(F)F)C(=O)Nc4ccc5nn(C)cc5c4 | 8.1 E-9 |
| **67** | CCC(N1C=C(OC)C(=CC1=O)c2cc(Cl)ccc2c3nnc(s3)C(F)F)C(=O)Nc4ccc5nn(C)cc5c4 | 5.9 E-9 |
| **68** | CCC(N1C=C(OC)C(=CC1=O)c2cc(Cl)ccc2c3cc(on3)C(F)F)C(=O)Nc4ccc5nn(C)cc5c4 | 4.2 E-9 |
| **69** | CCC(N1C=C(OC)C(=CC1=O)c2cc(Cl)ccc2c3oc(nc3)C(F)F)C(=O)Nc4ccc5nn(C)cc5c4 | 7.6 E-8 |
| **70** | CCC(N1C=C(OC)C(=CC1=O)c2cc(Cl)ccc2c3ccn[nH]3)C(=O)Nc4ccc5nn(C)cc5c4 | 2.8 E-8 |
| **71** | CC[C@H](N1C=C(OC)C(=CC1=O)c2cc(Cl)ccc2n3cc(nn3)C(F)F)C(=O)Nc4ccc5nccnc5c4 | 7.0 E-10 |
| **72** | CC[C@H](N1C=C(OC)C(=CC1=O)c2cc(Cl)ccc2n3cc(nn3)C(F)(F)F)C(=O)Nc4ccc5nc(C)c(C)nc5c4 | 8.9 E-10 |
| **73** | CC[C@H](N1C=C(OC)C(=CC1=O)c2cc(Cl)ccc2n3cc(Cl)nn3)C(=O)Nc4ccc5nn(cc5c4)C(F)F | 9.8 E-10 |
| **74** | CC[C@H](N1C=C(OC)C(=CC1=O)c2cc(Cl)ccc2n3cc(Cl)nn3)C(=O)Nc4ccc(cc4)C(=O)N | 8.1 E-10 |
| **75** | COC1=CN([C@@H](C)C(=O)Nc2ccc(C(=O)N)c(F)c2)C(=O)C=C1c3cc(Cl)ccc3n4cc(Cl)nn4 | 6.4 E-10 |
| **76** | COC1=CN([C@@H](C)C(=O)Nc2ccc(C(=O)N)c(F)c2)C(=O)C=C1c3cc(Cl)ccc3n4cc(nn4)C(F)F | 9.8 E-10 |
| **77** | COC1=CN([C@@H](C)C(=O)Nc2ccc(C(=O)N)c(F)c2)C(=O)C=C1c3cc(Cl)ccc3n4cc(nn4)C(F)(F)F | 7. E-10 |
| **78** | CC[C@H](N1C=C(OC)C(=CC1=O)c2cc(Cl)ccc2n3cc(Cl)nn3)C(=O)Nc4ccc(C(=O)N)c(F)c4 | 8.9 E-10 |
| **79** | CC[C@H](N1C=C(OC)C(=CC1=O)c2cc(Cl)ccc2n3cc(nn3)C(F)F)C(=O)Nc4ccc(C(=O)N)c(F)c4 | 1.4 E-9 |
| **80** | CC[C@H](N1C=C(OC)C(=CC1=O)c2cc(Cl)ccc2n3cc(nn3)C(F)(F)F)C(=O)Nc4ccc(C(=O)N)c(F)c4 | 9.2 E-10 (1.0 E-9)[1] |
| **81** | CCC[C@H](N1C=C(OC)C(=CC1=O)c2cc(Cl)ccc2n3cc(Cl)nn3)C(=O)Nc4ccc(C(=O)N)c(F)c4 | 1.7 E-9 |
| **82** | CCC[C@H](N1C=C(OC)C(=CC1=O)c2cc(Cl)ccc2n3cc(nn3)C(F)F)C(=O)Nc4ccc(C(=O)N)c(F)c4 | 3.1 E-9 |
| **83** | CCC[C@H](N1C=C(OC)C(=CC1=O)c2cc(Cl)ccc2n3cc(nn3)C(F)(F)F)C(=O)Nc4ccc(C(=O)N)c(F)c4 | 1.6 E-9 |
| **84** | CC[C@H](N1C=CC(=CC1=O)c2cc(Cl)ccc2n3cc(Cl)nn3)C(=O)Nc4ccc(C(=O)N)c(F)c4 | 1.8 E-8 |

| | | |
|---|---|---|
| **85** | CC[C@H](N1C=CC(=CC1=O)c2cc(Cl)ccc2n3cc(nn3)C(F)(F)F)C(=O)Nc4ccc(C(=O)N)c(F)c4 | 2.1 E-8 |
| **355** | OC(=O)c1ccc(NC(=O)CN2C(=O)C=C(C3=C2CCC3=O)c4cc(Cl)ccc4C#N)cc1 | 6.0 E-8 |
| **362** | CC(N1C(=O)C=C(C2=C1CCC2=O)c3cc(Cl)ccc3C#N)C(=O)Nc4ccc(cc4)C(=O)O | 3.4 E-7 |

[1] in depth evaluation

## REFERENCES


(1) Halgren, T. A. Identifying and Characterizing Binding Sites and Assessing Druggability. *J. Chem. Inf. Model.* **2009**, *49*, 377−389.

(2) Sastry, G. M.; Adzhigirey, M.; Day, T.; Annabhimoju, R.; Sherman, W. Protein and Ligand Preparation: Parameters, Protocols, and Influence on Virtual Screening Enrichments. *J. Comput. Aided Mol. Des.* **2013**, *27*, 221−234.

(3) Abel, R.; Young, T.; Farid, R.; Berne, B. J.; Friesner, R. A. Role of the Active-Site Solvent in the Thermodynamics of Factor Xa Ligand Binding. *J. Am. Chem. Soc.* **2008**, *130*, 2817−2831.

(4) Young, T.; Abel, R.; Kim, B.; Berne, B. J.; Friesner, R. A. Motifs for Molecular Recognition Exploiting Hydrophobic Enclosure in Protein-Ligand Binding. *Proc. Natl. Acad. Sci. USA* **2007**, *104*, 808−813.

(5) Schneckener, S.; Grimbs, S.; Hey, J.; Menz, S.; Osmers, M.; Schaper, S.; Hillisch, A.; Göller, A. H. Prediction of Oral Bioavailability in Rats: Transferring Insights from In Vitro Correlations to (Deep) Machine Learning Models Using In Silico Model Outputs and Chemical Structure Parameters. *J. Chem. Inf. Model.* **2019**, *59*, 4893−4905.

(6) Ertl, P.; Rohde, B.; Selzer, P. Fast Calculation of Molecular Polar Surface Area as a Sum of Fragment-Based Contributions and its Application to the Prediction of Drug Transport Properties. *J. Med. Chem.* **2000**, *43*, 3714−3717.

(7) Lobell, M.; Hendrix, M.; Hinzen, B.; Keldenich, J.; Meier, H.; Schmeck, C.; Schohe-Loop, R.; Wunberg, T.; Hillisch, A. In Silico ADMET Traffic Lights as a Tool for the Prioritization of HTS Hits. *ChemMedChem* **2006**, *1*, 1229−1236.

(8) Friesner, R. A.; Banks, J. L.; Murphy, R. B.; Halgren, T. A.; Klicic, J. J.; Mainz, D. T.; Repasky, M. P.; Knoll, E. H.; Shelley, M.; Perry, J. K.; Shaw, D. E.; Francis, P.; Shenkin, P. S. Glide: A New Approach for Rapid, Accurate Docking and Scoring. 1. Method and Assessment of Docking Accuracy. *J. Med. Chem.* **2004**, *47*, 1739−1749.

(9) Shivakumar, D.; Williams, J.; Wu, Y.; Damm, W.; Shelley, J.; Sherman, W. Prediction of Absolute Solvation Free Energies Using Molecular Dynamics Free Energy Perturbation and the OPLS Force Field. *J. Chem. Theory Comput.* **2010**, *6*, 1509–1519.

(10) Madhavi Sastry, G.; Adzhigirey, M.; Day, T.; Annabhimoju, R.; Sherman, W. Protein and Ligand Preparation: Parameters, Protocols, and Influence on Virtual Screening Enrichments. *J. Comput. Aided Mol. Des.* **2013**, *27*, 221–234.

(11) Schrödinger Release 2015-1: Protein Preparation Wizard; Epik, Schrödinger, LLC, New York, NY, **2015**; Impact, Schrödinger, LLC, New York, NY; Prime, Schrödinger, LLC, New York, NY, **2015**.

(12) Jacobson, M. P.; Pincus, D. L.; Rapp, C. S.; Day, T. J. F.; Honig, B.; Shaw, D. E.; Friesner, R. A. A Hierarchical Approach to All-Atom Protein Loop Prediction. *PROTEINS: Structure, Function, and Bioinformatics* **2004**, *55*, 351–367.

(13) Wang, L.; Wu, Y.; Deng, Y.; Kim, B.; Pierce, L.; Krilov, G.; Lupyan, D.; Robinson, S.; Dahlgren, M. K.; Greenwood, J.; Romero, D. L.; Masse, C.; Knight, J. L.; Steinbrecher, T.; Beuming, T.; Damm, W.; Harder, E.; Sherman, W.; Brewer, M.; Wester, R.; Murcko, M.; Frye, L.; Farid, R.; Lin, T.; Mobley, D. L.; Jorgensen, W. L.; Berne, B. J.; Friesner, R. A.; Abel, R. Accurate and Reliable Prediction of Relative Ligand Binding Potency in Prospective Drug Discovery by Way of a Modern Free-Energy Calculation Protocol and Force Field. *J. Am. Chem. Soc.* **2015**, *137*, 2695−2703.

(14) Lu, C.; Wu, C.; Ghoreishi, D.; Chen, W.; Wang, L.; Damm, W.; Ross, G. A.; Dahlgren, M. K.; Russell, E.; Von Bargen, C. D.; Abel, R.; Friesner, R. A.; Harder, E. D. OPLS4: Improving Force Field Accuracy on Challenging Regimes of Chemical Space. *J. Chem. Theory Comput.* **2021**, *17*, 4291–4300.

(15) Minor, W.; Cymborowski, M.; Otwinowski, Z.; Chruszcz, M. HKL-3000: The Integration of Data Reduction and Structure Solution – from Diffraction Images to an Initial Model in Minutes. *Acta Cryst.* **2006**, *D62*, 859−866.

(16) Kabsch, W. XDS. *Acta Cryst.* **2010**, *D66*, 125−132.

(17) Read, R. J. Pushing the Boundaries of Molecular Replacement with Maximum Likelihood. *Acta Cryst.* **2001**, *D57*, 1373−1382.

(18) Winn, M. D.; Murshudov, G. N.; Papiz, M. Z. Macromolecular TLS Refinement in REFMAC at Moderate Resolutions. *Methods in Enzymology* **2003**, *374*, 300−321.

(19) Schüttelkopf, A. W.; van Aalten, D. M. PRODRG: a tool for high-throughput crystallography of protein-ligand complexes. *Acta Cryst.* **2004**, *D60*, 1355–1363.

(20) Emsley, P.; Lohkamp, B.; Scott, W. G.; Cowtan, K. Features and development of Coot. *Acta Cryst.* **2010**, *D66*, 486–501.

(21) Artursson, P.; Karlsson, J. Correlation between oral drug absorption in humans and apparent drug permeability coefficients in human intestinal epithelial (Caco-2) cells. *Biochem Biophys Res Commun.* **1991**, *175*, 880–885.

(22) Walsky, R. L.; Obach, R. S. Validated assays for human cytochrome P450 activities. *Drug Metab. Dispos.* **2004**, *32*, 647−660.

(23) Obach, R. S.; Walsky, R. L.; Venkatakrishnan, K. Mechanism-based inactivation of human cytochrome P450 enzymes and the prediction of drug-drug interactions. *Drug Metab. Dispos.* **2007**, *35*, 246−255.
